# Discovery and Preliminary Structure-Activity Investigation of 3-Substituted-1*H*-imidazol-5-yl-1*H*-indoles with In Vitro Activity towards Methicillin-Resistant *Staphylococcus aureus*

**DOI:** 10.3390/antibiotics11101450

**Published:** 2022-10-21

**Authors:** Steven A. Li, Rebecca J. Zheng, Kenneth Sue, Marie-Lise Bourguet-Kondracki, Azza Troudi, Jean Michel Brunel, Brent R. Copp, Melissa M. Cadelis

**Affiliations:** 1 School of Chemical Sciences, The University of Auckland, Private Bag 92019, Auckland 1142, New Zealand; 2 Laboratoire Molécules de Communication et Adaptation des Micro-Organismes, UMR 7245 CNRS, Muséum National d’Histoire Naturelle, 57 rue Cuvier (C.P. 54), 75005 Paris, France; 3 UMR MD1 “Membranes et Cibles Thérapeutiques”, U1261 INSERM, Faculté de Pharmacie, Aix-Marseille Universite, 27 bd Jean Moulin, 13385 Marseille, France; 4 School of Medical Sciences, The University of Auckland, Private Bag 92019, Auckland 1142, New Zealand

**Keywords:** MRSA, antimicrobial, imidazole, antifungal

## Abstract

Antibiotics have been the cornerstone of modern medicine saving lives by virtue of being able to cure infectious diseases and to prevent infections in those who are immune compromised. Their intense use has led to a surging increase in the incidence of antibiotic-resistant bacteria resulting in a desperate need for antibiotics with new mechanisms of action. As part of our search for new antimicrobials we have screened an in-house library of compounds and identified two 3-substituted-1*H*-imidazol-5-yl-1*H*-indoles as weak growth inhibitors (MIC 16 µg/mL) against methicillin-resistant *Staphylococcus aureus* (MRSA). An extensive library of analogues was prepared using the Van Leusen three-component reaction, biological evaluation of which led to the identification of two analogues (**26** and **32**) with favorable anti-MRSA activity (MIC ≤ 0.25 µg/mL) which also lacked cytotoxic or hemolytic properties. The screening campaign also identified two derivatives, a phenethyl-indole-imidazole **57** and a 5-phenyl-1*H*-imidazole **111** that were non-toxic selective antifungals towards *Cryptococcus neoformans*. These results have identified 3-substituted-1*H*-imidazol-5-yl-1*H*-indoles and 5-phenyl-1*H*-imidazoles as new structural scaffolds for further investigation as anti-MRSA and anti-*C. neoformans* agents, respectively.

## 1. Introduction

New Zealand has a growing problem with *Staphylococcus aureus* infection, experiencing higher levels of incidence than other developed countries, with incidences of *S. aureus*-related hospitalizations highest in the under-five and over 75 year age groups [1,2,3,4,5,6,7]. Over the ten-year period of 2001–2011, the incidence of methicillin-susceptible *S. aureus* infections increased to 361 per 100,000, with MRSA infection (covering non-multidrug resistant MRSA and multidrug resistant MRSA) accounting for an additional 12% of cases. In the case of children under the age of 15, annual average hospitalization rates for *S. aureus* skin and soft tissue infection (SSTI) increased to 522 per 100,000 population (2011), with sub-population analysis identifying Māori and Pacific children with rates of 1488 and 1215 per 100,000 population, respectively [1,2,3,4,5,6,7].

Front-line treatments for SSTI in New Zealand are the topical antimicrobials fusidic acid and mupirocin. During the 1993-2012 timeframe, community prescribing rates for fusidic acid increased dramatically (likely due to the drug becoming a Government-subsidized treatment option) and this was matched with a corresponding increase in fusidic acid-resistant MRSA [8,9]. Unfortunately, the widespread, and largely unregulated, use of this valuable topical antimicrobial resource will ultimately lead to the need for more treatment options.

**Figure 1:**
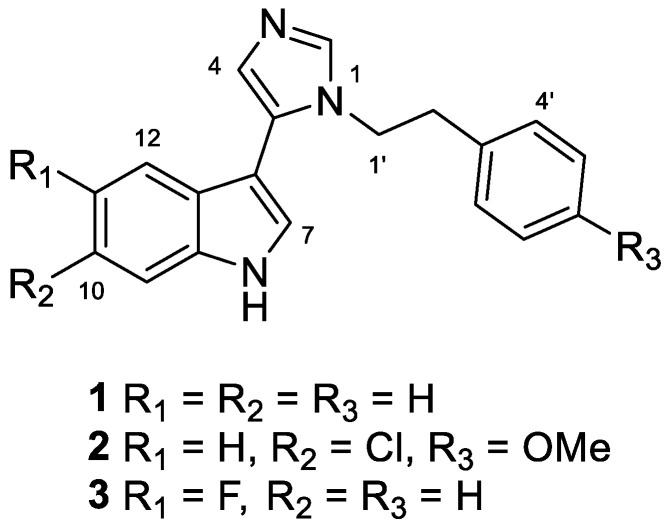
Structures of indole-imidazole compounds **1**–**3**.

We have recently screened an in-house library of natural products and synthetic compounds against a panel of bacterial and fungal pathogens [10,11] which led to the discovery of a compound class (Figure 1) that exhibited activity exclusively against methicillin-resistant *Staphylococcus aureus* (MRSA). Of the compounds screened, 3-substituted-1*H*-imidazol-5-yl-1*H*-indole (“indole-imidazole”) compounds **1** and **2** were found to weakly inhibit the growth of MRSA with MIC of 16 µg/mL (Table 1) while a closely related 5-fluoro analogue **3** (Supplementary Figures S1–S3) was devoid of activity.

Similarly functionalized 1*H*-imidazol-5-yl-1*H*-indoles have been recently reported as active towards crop-related fungi [12] and as 5-HT_7_ serotonin receptor agonists [13]. Variants with increased substitution such as the marine natural products topsentins [14,15] and nortopsentins [16,17] have been shown to exhibit cytotoxic, antiviral and antifungal activities while analogues of the natural product meridianin exhibit antibiotic adjuvant properties [18]. Additionally, 1-benzenesulfonyl-1*H*-indoles have been reported as 5-HT_6_ receptor agonists [19]. With this relatively limited literature on 3-substituted 1*H*-imidazol-5-yl-1*H*-indoles, we undertook a study to further explore the antimicrobial properties of this class of molecule by preparing a more diverse array of analogues that explored the influence of the imidazole *N*-substituent and indole halogen substitution on biological activity. Herein we report on the synthesis and biological evaluation of a diverse set of analogues of hit compounds **1** and **2**. 

## 2. Results and Discussion

A set of 39 new analogues were prepared (**4**–**42**) (Figure 2 and Supplementary Materials Figures S4–S42) utilizing the Van Leusen three-component reaction [20]. This two-step, one-pot reaction starts with in situ generation of an imine by condensation of an indole-3-carbaldehyde with an amine, followed by reaction with *p-*toluenesulfonylmethyl isocyanide (TosMIC) and K_2_CO_3_ at 60 °C for 24 h (Scheme 1 and Scheme 2).

**Figure 2:**
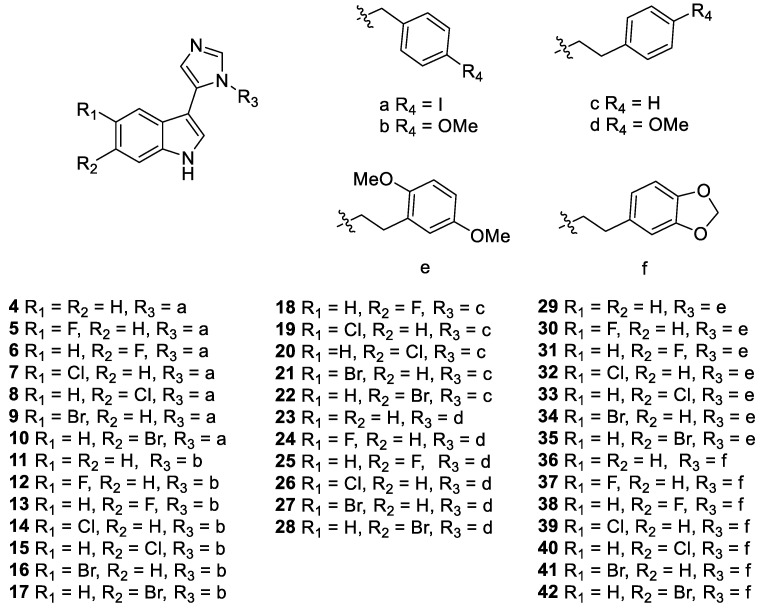
Structures of substituted indole-imidazoles **4**–**42**.

The set of analogues included the indole fragment bearing 5- and 6- halogen substituents of bromine, chlorine, and fluorine, as well as an unsubstituted indole. The differently substituted indoles offer an opportunity to probe the effect of the electronics of the indole ring against antimicrobial activity. Six amines were used to introduce variation at the R_3_ position (Figure 2). These included oxygenated amines such as *p*-methoxybenzylamine, *p*-methoxyphenethylamine, 2,5-dimethoxyphenethylamine and homopiperonylamine probe for hydrogen bond interactions. The phenethylamines explore an increased alkyl chain length between the aromatic group of the amine and the imidazole linker while *p*-iodobenzylamine can be used to directly compare with *p*-methoxybenzylamine the difference in bioactivity in the presence of a bulky halogen.

The antimicrobial activities of compounds **4**–**42** were determined against a panel of bacterial (*Staphylococcus aureus*, methicillin-resistant *S. aureus*, *Pseudomonas aeruginosa*, *Escherichia coli*, *Klebsiella pneumoniae* and *Acinetobacter baumannii*) and two fungal (*Candida albicans* and *Cryptococcus neoformans*) pathogens. The results are summarized in Table 1. Antimicrobial selectivity was observed towards the Gram-positive bacteria *S. aureus* and *S. aureus* MRSA, with MIC values ranging from ≤0.25 µg/mL to >200 µg/mL. None of the compounds exhibited activity against the Gram-negative bacteria *P. aeruginosa*, *E. coli*, *A. baumannii* or *K. pneumoniae* or the fungus *C. albicans* at a single dose of 32 µg/mL. Of the compound set, only **9** exhibited (weak, MIC 16 µg/mL) activity towards the fungus *C. neoformans*.

The more active anti-MRSA compounds were found to contain halogen substitution on the indole, with the 5- position analogues typically being more active than their 6- substituted counterparts, and the presence of an *N*-phenethyl substituent on the imidazole ring, e.g., **26** (MIC ≤ 0.25 µg/mL), **27** (8 µg/mL) and **32** (4 µg/mL). The set of compounds were also evaluated for cytotoxicity against a human embryonic kidney cell line (HEK293) and for hemolytic activity towards human red blood cells (Table 1). Of the compounds tested, none exhibited hemolytic activity at a single dose of 32 µg/mL but quite a few were identified as exhibiting some degree of cytotoxicity. Of note however was the lack of cytotoxicity observed for the two more active anti-MRSA compounds **26** and **32**.

**Table 1:** Antimicrobial (MIC, µg/mL), cytotoxic (µg/mL) and hemolytic (µg/mL) activities of selected compounds.

Compound	MIC (µg/mL)			HEK293 CC_50_ (µg/mL) ^d^	HC_10_ (µg/mL) ^e^
	*S. a*^a^	MRSA ^b^	*C. n*^c^		
**1**	>200	16	>32	19.3	>32
**2**	>200	16	32	22.5	>32
**6**	6.25	>32	32	15.6	>32
**9**	>200	16	16	5.1	>32
**11**	>200	32	>32	25.6	>32
**17**	25	32	32	11.1	>32
**18**	25	32	>32	16.3^c^	>32^c^
**19**	12.5	16	>32	17.3	>32
**21**	6.25	16	32	10.6	>32
**22**	12.5	16	32	12.7	>32
**23**	100	32	>32	24.5	>32
**25**	100	16	>32	27.0	>32
**26**	>200	≤0.25	>32	>32	>32
**27**	12.5	8	>32	16.0	>32
**28**	12.5	32	32	15.6	>32
**29**	>200	32	>32	11.5	>32
**32**	100	4	>32	>32	>32
**34**	25	16	>32	6.2	>32
**42**	12.5	16	32	16.1	>32
**57**	>200	>32	≤0.25	>32	>32
**111**	50	16	≤0.25	>32	>32

^a^*Staphylococcus aureus* ATCC25923 with streptomycin (MIC 12.5 μg/mL) and chloramphenicol (MIC 0.5–1 μg/mL) used as positive controls and values presented as the mean (*n* = 3); ^b^ *Staphylococcus aureus* ATCC43300 (MRSA) with vancomycin (MIC 1 μg/mL) used as a positive control and values presented as the mean (*n* = 2); ^c^ *Cryptococcus neoformans* ATCC208821 with fluconazole (MIC 8 μg/mL) as a positive control and values presented as the mean (*n* = 2); ^d^ Concentration of compound at 50% cytotoxicity on HEK293 human embryonic kidney cells and values presented as the mean (*n* = 2). Tamoxifen was the positive control (IC_50_ 9 μg/mL); ^e^ Concentration of compound at 10% hemolytic activity on human red blood cells and values presented as the mean (*n* = 2). Melittin was the positive control (HC_10_ 2.7 μg/mL).

A further, more-expansive set of analogues was prepared (Figure 3 and Figure 4) that explored the influence of 4- and 7- halogen or 5/6-methoxy substituted indoles bearing benzyl, 2-phenethyl or 3-phenylpropyl (compounds **43**–**72**) or indolemethylene or indole-ethyl (**73**–**88**) and *n*-pentyl (**89**–**96**) side chains. In addition, analogues **97**–**102** were prepared that retained a common 5-chloroindole fragment, identified as being associated with activity in set 1, but with a wider variation in R_3_ fragment (Figure 3), while analogues **103**–**116** explored replacement of the indole fragment with a phenyl or *p*-methoxyphenyl group (Figure 4). A total of 74 compounds (**43**–**116**) were prepared in this library (Figures S43–S116). 

Analogues **43**–**116** were evaluated for biological activity in the same manner as the earlier compound sets. Surprisingly, from the 74 analogues tested, none were considered active towards MRSA (MIC 32 µg/mL or greater). This result suggests that the structural requirements for activity of the indole-imidazole scaffold towards MRSA is very precise/narrow, with halogenation on 5- position of the indole and a methoxy phenethyl sidechain, making it difficult to identify ways forward in further optimizing the structure. Of note from this set of compounds was the observation of potent antifungal activity against *C. neoformans* (MIC values of ≤0.25 µg/mL) for both 6-methoxy-phenethyl-indole-imidazole **57** and 5-phenyl-1*H*-imidazole **111** (Table 1): the lack of cytotoxicity and hemolytic activity for these two compounds identifies them as selective hits and worthy of further investigation. 

Currently, the mechanism of antimicrobial action of this compound class is unknown. Thus, we sought to investigate the mechanism of action of this compound class against *S. aureus* using compound **6**, which exhibited strong activity against *S. aureus*. In a preliminary investigation, compound **6** exhibited only very weak membrane depolarization of *S. aureus*, suggesting the cellular target in bacteria is not membrane-related. Further investigation to improve the antimicrobial and antifungal activity and identify the mechanism of action of these compounds is underway.

## 3. Materials and Methods

### 3.1. General Experimental Procedures

High-resolution mass spectra were recorded using a MicrOTOF-QII mass spectrometer (Bruker Daltonics, Bremen, Germany). Melting points were determined on a Reichert-Hofler block and are uncorrected. Infra-red spectra were recorded on a Perkin Elmer Spectrum 100 Fourier Transform infrared spectrometer equipped with a universal ATR accessory. NMR spectra were recorded using a Bruker Avance 400 MHz or Avance III-HD 500 spectrometer operating at 400 or 500 MHz for ^1^H nuclei and 100 or 125 MHz for ^13^C nuclei. Proto-deutero solvents signals were use as internal references (DMSO-*d*_6_: δ_H_ 2.50, δ_C_ 39.52; CDCl_3_: δ_H_ 7.26, δ_C_ 77.16). For ^1^H NMR, the data are quoted as position (δ), relative integral, multiplicity (s = singlet, d = doublet, t = triplet, m = multiplet, dd = doublet of doublets, ddd = doublet of doublet of doublets, tt = triplet of triplets, br = broad), coupling constant (*J*, Hz), and assignment to the atom. The ^13^C NMR data are quoted as position (δ), and assignment to the atom. Standard Bruker pulse sequences were utilized. Pressurized (flash) column chromatography was carried out with Kieselgel 60 0.063–0.200 mesh (Merck, Darmstadt, Germany). Analytical thin layer chromatography (TLC) was carried out on 0.2 mm thick plates of Kieselgel F_254_ or DC-Kieselgel 60 RP-18 F_254_S (Merck). All samples were determined to >95% purity.

### 3.2. Synthesis

#### 3.2.1. General Procedure

A solution of aldehyde (1 eq.) and amine (1 eq.) in DMF (1 mL) was stirred for 3 h under N_2_ atmosphere. To the stirred solution was added solid K_2_CO_3_ (1 eq.) and *p*-toluenesulfonylmethyl isocyanide (1 eq.) before heating to 60 °C for 18 h. The reaction mixture was cooled to room temperature and quenched with H_2_O (30 mL). The aqueous layer was extracted with EtOAc (30 mL), and the organic layer was washed with H_2_O (3 × 30 mL) then brine (3 × 30 mL), dried over anhydrous MgSO_4_ and concentrated under reduced pressure. 

#### 3.2.2. 3-(1-Phenethyl-1*H*-imidazol-5-yl)-1*H*-indole (**1**) 

Using the general procedure, reaction of 1*H*-indole-3-carbaldehyde (0.145 g, 1.0 mmol) with phenethylamine (0.12 mL, 1.0 mmol), K_2_CO_3_ (0.138 g, 1.0 mmol) and *p*-toluenesulfonylmethyl isocyanide (0.195 g, 1.0 mmol) in DMF (1 mL) followed by purification using silica gel column chromatography (1%–10% CH_2_Cl_2_/MeOH, 99:1→9:1) afforded the title compound as a brown solid (0.135 g, 47%). *R_f_ =* 0.60 (CH_2_Cl_2_/MeOH, 9:1); m.p 69–70 °C; IR (ATR) ν_max_ 3350, 2355, 1641, 1511, 1298, 907, 841, 742 cm^−1^; ^1^H NMR (400 MHz, DMSO-*d*_6_) δ 11.47 (1H, s, NH-8), 7.63 (1H, s, H-2), 7.52–7.47 (3H, m, H-7, H-9, H-12), 7.24–7.17 (2H, m, 2H-5′), 7.17–7.15 (2H, m, H-10, H-6′), 7.07 (1H, dd, *J =* 7.3, 7.3 Hz, H-11), 7.01 (1H, s, H-4), 7.00 (2H, d, *J =* 7.3 Hz, 2H-4′), 4.23 (2H, t, *J* = 7.4 Hz, H_2_-1′), 2.86 (2H, t, *J* = 7.4 Hz, H_2_-2′); ^13^C NMR (100 MHz, DMSO-*d*_6_) δ 138.3 (C-8a), 138.0 (C-3′), 136.5 (C-2), 128.9 (2C-4′), 128.7 (2C-5′), 127.4 (C-6′), 127.0 (C-12a), 126.8 (C-4), 126.4 (C-5), 124.8 (C-7), 122.1 (C-10), 120.0 (C-11), 119.1 (C-12), 112.2 (C-9), 104.0 (C-6), 46.4 (C-1′), 36.6 (C-2′); (+)-HRESIMS *m/z* 288.1497 [M+H]^+^ (calcd for C_19_H_18_N_3_, 288.1495) 

#### 3.2.3. 6-Chloro-3-(1-(4-methoxyphenethyl)-1*H*-imidazol-5-yl)-1*H*-indole (**2**) 

Using the general procedure, reaction of 6-chloro-1*H*-indole-3-carbaldehyde (0.179 g, 1.0 mmol) with 4-methoxyphenethylamine (0.15 mL, 1.0 mmol), K_2_CO_3_ (0.138 g, 1.0 mmol) and *p*-toluenesulfonylmethyl isocyanide (0.195 g, 1.0 mmol) in DMF (1 mL) followed by purification using silica gel column chromatography (1%–10% CH_2_Cl_2_/MeOH, 99:1→9:1) afforded the title compound as a yellow solid (0.214 g, 61%). *R_f_* = 0.57 (CH_2_Cl_2_/MeOH, 9:1); m.p 165–166 °C; IR (ATR) ν_max_ 2777, 1611, 1511, 1449, 1245, 1112, 909, 827, 810 cm^−1^; ^1^H NMR (400 MHz, DMSO*-d*_6_) δ 11.55 (1H, br s, NH-8), 7.60 (1H, d, *J* = 1.0 Hz, H-2), 7.51 (1H, d, *J* = 2.7 Hz, H-7), 7.49 (1H, d, *J* = 2.0 Hz, H-9), 7.46 (1H, d, *J* = 8.6 Hz, H-12), 7.06 (1H, dd, *J* = 8.6, 2.0 Hz, H-11), 6.98 (1H, d, *J* = 1.0 Hz, H-4), 6.89 (2H, d, *J* = 8.8 Hz, 2H-4′), 6.76 (2H, d, *J =* 8.8 Hz, 2H-5′), 4.16 (2H, t, *J* = 7.3 Hz, H_2_-1′), 3.68 (3H, s, OMe), 2.77 (2H, t, *J* = 7.3 Hz, H_2_-2′); ^13^C NMR (100 MHz, DMSO-*d*_6_) δ 157.8 (C-6′), 137.8 (C-2), 136.4 (C-8a), 129.8 (C-3′), 129.5 (2C-4′), 127.5 (C-4), 126.4 (C-10), 125.3 (C-12a), 125.2 (C-7), 125.1 (C-5), 120.2 (C-12), 119.9 (C-11), 113.7 (2C-5′), 111.4 (C-9), 104.2 (C-6), 54.9 (OMe), 46.1 (C-1′), 35.3 (C-2′); (+)-HRESIMS *m/z* 374.1033 [M+Na]^+^ (calcd for C_20_H_18_^35^ClN_3_NaO, 374.1031), *m/z* 376.0999 [M+Na]^+^ (calcd for C_20_H_18_^37^ClN_3_NaO, 374.1008). 

#### 3.2.4. 5-Fluoro-3-(1-phenethyl-1*H*-imidazol-5-yl)-1*H*-indole (**3**) 

Using the general procedure, reaction of 5-fluoro-1*H*-indole-3-carbaldehyde (0.163 g, 1.0 mmol) with phenethylamine (0.12 mL, 1.0 mmol), K_2_CO_3_ (0.138 g, 1.0 mmol) and *p*-toluenesulfonylmethyl isocyanide (0.195 g, 1.0 mmol) in DMF (1 mL) followed by purification using silica gel column chromatography (1%–10% CH_2_Cl_2_/MeOH, 99:1→9:1) afforded the title compound as a brown solid (0.210 g, 69%). *R_f_* = 0.63 (CH_2_Cl_2_/MeOH, 9:1); m.p 127–130 °C; IR (ATR) ν_max_ 3053, 1581, 1473, 1216, 1106, 932, 848, 786 cm^−1^; ^1^H NMR (400 MHz, DMSO*-d*_6_) δ 11.54 (1H, br s, NH-8), 7.62 (1H, d, *J* = 1.0 Hz, H-2), 7.56 (1H, d, *J* = 2.7 Hz, H-7), 7.46 (1H, dd, *J* = 8.8, 4.5 Hz, H-9), 7.24–7.18 (2H, m, 2H-5′), 7.18–7.15 (2H, m, H-12, H-6′), 7.04-6.98 (4H, m, H-4, H-10, 2H-4′), 4.20 (2H, t, *J* = 7.4 H, H_2_-1′), 2.84 (2H, t, *J* = 7.4 Hz, H_2_-2′); ^13^C NMR (100 MHz, DMSO-*d*_6_), δ 157.4 (d, ^1^*J*_CF_ = 231.8 Hz, C-11), 138.0 (C-3′), 137.7 (C-2), 132.7 (C-8a), 128.5 (2C-4′), 128.3 (2C-5′), 127.4 (C-6′), 126.8 (C-12a), 126.4 (C-4/C-7), 126.3 (C-4/C-7), 125.2 (C-5), 112.9 (d, ^3^*J*_CF_ = 9.5 Hz, C-9), 109.9 (d, ^2^*J*_CF_ = 25.5, C-10), 104.0 (d, ^4^*J*_CF_ = 4.8 Hz, C-6), 103.4 (d, ^2^*J*_CF_ = 24.7, C-12), 45.8 (C-1′), 36.2 (C-2′); (+)-HRESIMS *m/z* 328.1215 [M+Na]^+^ (calcd for C_19_H_16_FN_3_Na, 328.1220). 

#### 3.2.5. 3-(1-(4-Iodobenzyl)-1*H*-imidazol-5-yl)-1*H*-indole (**4**) 

Using the general procedure, reaction of 1*H*-indole-3-carbaldehyde (0.145 g, 1.0 mmol) with 4- iodobenzylamine (0.233 g, 1.0 mmol), K_2_CO_3_ (0.138 g, 1.0 mmol) and *p*-toluenesulfonylmethyl isocyanide (0.195 g, 1.0 mmol) in DMF (1 mL) followed by purification by trituration from CH_2_Cl_2_ afforded the title compound as a pale yellow solid (0.023 g, 6%). *R_f_* = 0.77 (CH_2_Cl_2_/MeOH, 9:1); m.p > 200 °C; IR (ATR) ν_max_ 2981, 1611, 1450, 1403, 1230, 1118, 917, 795 cm^−1^; ^1^H NMR (400 MHz, DMSO*-d*_6_) δ 11.31 (1H, br s, NH-8), 7.81 (1H, s, H-2), 7.62 (2H, d, *J* = 8.3 Hz, 2H-4′), 7.51, (1H, d, *J* = 7.6 Hz, H-12), 7.41 (1H, d, *J* = 7.6 Hz, H-9), 7.25 (1H, d, *J* = 2.4 Hz, H-7), 7.14 (1H, ddd, *J* = 14.9, 7.6, 0.8 Hz, H-10), 7.12 (1H, s, H-4), 7.05 (1H, ddd, *J* = 14.9, 7.6, 0.8 Hz, H-11), 6.75 (2H, d, *J* = 8.3 Hz, 2H-3′), 5.25 (2H, s, H_2_-1′); ^13^C NMR (100 MHz, DMSO-*d*_6_) δ 138.3 (C-2), 137.9 (C-2′), 137.3 (2C-4′), 135.9 (C-8a), 128.6 (2C-3′), 127.4 (C-4), 126.3 (C-5/C-12a), 126.2 (C-5/C-12a), 123.8 (C-7), 121.7 (C-10), 119.6 (C-11), 118.8 (C-12), 111.7 (C-9), 103.4 (C-6), 93.2 (C-5′), 47.2 (C-1′); (+)-HRESIMS *m/z* 400.0305 [M+H]^+^ (calcd for C_18_H_15_IN_3_, 400.0301). 

#### 3.2.6. 5-Fluoro-3-(1-(4-iodobenzyl)-1*H*-imidazol-5-yl)-1*H*-indole (**5**) 

Using the general procedure, reaction of 5-fluoro-1*H*-indole-3-carbaldehyde (0.163 g, 1.0 mmol) with 4-iodobenzylamine (0.233 g, 1.0 mmol), K_2_CO_3_ (0.138 g, 1.0 mmol) and *p*-toluenesulfonylmethyl isocyanide (0.195 g, 1.0 mmol) in DMF (1 mL) followed by purification using silica gel column chromatography (1%–10% CH_2_Cl_2_/MeOH, 99:1→9:1) afforded the title compound as a brown solid (0.124 g, 30%). *R_f_* = 0.63 (CH_2_Cl_2_/MeOH, 9:1); m.p 187–188 °C; IR (ATR) ν_max_ 3046, 2777, 1630, 1583, 1507, 1468, 1229, 1118, 847, 794 cm^−1^; ^1^H NMR (400 MHz, DMSO*-d*_6_) δ 11.42 (1H, br s, NH-8), 7.83 (1H, d, *J* = 1.0 Hz, H-2), 7.62 (2H, d, *J* = 8.5 Hz, 2H-4′), 7.41 (1H, dd, *J* = 9.0, 4.6 Hz, H-9), 7.34 (1H, d, *J* = 2.7 Hz, H-7), 7.17 (1H, dd, *J* = 9.5, 2.4 Hz, H-12), 7.12 (1H, d, *J* = 1.0 Hz, H-4), 6.98 (1H, ddd, *J* = 9.5, 9.0, 2.5 Hz, H-11), 6.74 (2H, d, *J* = 8.5 Hz, 2H-3′), 5.24 (2H, s, H_2_-1′); ^13^C NMR (100 MHz, DMSO-*d*_6_) δ 157.4 (d, ^1^*J*_CF_ = 234.0 Hz, C-11), 138.5 (C-2), 138.8 (C-2′), 137.3 (2C-4′), 132.5 (C-8a), 128.6 (2C-3′), 127.5 (C-4), 126.6 (d, ^3^*J*_CF_ = 10.4 Hz, C-12a), 125.9 (C-7), 125.8 (C-5), 112.8 (d, ^3^*J*_CF_ = 9.6 Hz, C-9), 110.0 (d, ^2^*J*_CF_ = 25.7 Hz, C-10), 103.7 (C-6), 103.5 (d, ^2^*J*_CF_ = 23.4 Hz, C-12), 93.2 (C-5′), 47.2 (C-1′); (+)-HRESIMS *m/z* 440.0024 [M+Na]^+^ (calcd for C_18_H_13_FIN_3_Na, 440.0030). 

#### 3.2.7. 6-Fluoro-3-(1-(4-iodobenzyl)-1*H*-imidazol-5-yl)-1*H*-indole (**6**) 

Using the general procedure, reaction of 6-fluoro-1*H*-indole-3-carbaldehyde (0.163 g, 1.0 mmol) with 4-iodobenzylamine (0.233 g, 1.0 mmol), K_2_CO_3_ (0.138 g, 1.0 mmol) and *p*-toluenesulfonylmethyl isocyanide (0.195 g, 1.0 mmol) in DMF (1 mL) followed by purification by trituration from CH_2_Cl_2_ afforded the title compound as a light brown powder (0.119 g, 29%). *R_f_ =* 0.74 (CH_2_Cl_2_/MeOH, 9:1); m.p 191–192 °C; IR (ATR) ν_max_ 3081, 2996, 2807, 1627, 1508, 1458, 1231, 1115, 837, 793 cm^−1^; ^1^H NMR (400 MHz, DMSO*-d*_6_) δ 11.37 (1H, br s, NH-8), 7.82 (1H, d, *J* = 0.8 Hz, H-2), 7.62 (2H, d, *J* = 8.4 Hz, 2H-4′), 7.48 (1H, dd, *J* = 8.8, 5.4 Hz, H-12), 7.26 (1H, d, *J* = 2.5 Hz, H-7), 7.18 (1H, dd, *J* = 10.0, 2.5 Hz, H-9), 7.13 (1H, d, *J* = 0.8 Hz, H-4), 6.89 (1H, ddd, *J* = 9.8, 8.8, 2.5 Hz, H-11), 6.74 (2H, d, *J* = 8.4 Hz, 2H-3′), 5.24 (2H, s, H_2_-1′); ^13^C NMR (100 MHz, DMSO-*d*_6_) δ 159.0 (d, ^1^*J*_CF_ = 235.0 Hz, C-10), 138.4 (C-2), 137.8 (C-2′), 137.3 (2C-4′), 135.7 (d, ^3^*J*_CF_ = 12.7 Hz, C-8a), 128.6 (2C-3′), 127.6 (C-4), 125.8 (C-5), 124.3 (C-7), 123.1 (C-12a),119.9 (d, ^3^*J*_CF_ = 10.6 Hz, C-12), 108.1 (d, ^2^*J*_CF_ = 24.7 Hz, C-11), 103.7 (C-6), 97.6 (d, ^2^*J*_CF_ = 25.3, C-9), 93.2 (C-5′), 47.2 (C-1′); (+)-HRESIMS *m/z* 440.0030 [M+Na]^+^ (calcd for C_18_H_13_FIN_3_Na, 440.0023). 

#### 3.2.8. 5-Chloro-3-(1-(4-iodobenzyl)-1*H*-imidazol-5-yl)-1*H*-indole (**7**) 

Using the general procedure, reaction of 5-chloro-1*H*-indole-3-carbaldehyde (0.179 g, 1.0 mmol) with 4-iodobenzylamine (0.233 g, 1.0 mmol), K_2_CO_3_ (0.138 g, 1.0 mmol) and *p*-toluenesulfonylmethyl isocyanide (0.195 g, 1.0 mmol) in DMF (1 mL) followed by purification using silica gel column chromatography (1%–10% CH_2_Cl_2_/MeOH, 99:1→9:1) afforded the title compound as a pale yellow solid (0.033 g, 9%). *R_f_* = 0.58 (CH_2_Cl_2_/MeOH, 9:1); m.p >200 °C; IR (ATR) ν_max_ 3032, 2917, 2849, 1461, 1404, 1115, 1007, 886, 802, 792, 757 cm^−1^; ^1^H NMR (400 MHz, DMSO*-d*_6_) δ 11.52 (1H, br s, NH-8), 7.85 (1H, br s, H-2), 7.61 (2H, d, *J* = 8.3 Hz, 2H-4′), 7.42 (1H, d, *J* = 8.6 Hz, H-9), 7.37 (1H, d, *J* = 2.1 Hz, H-12), 7.36 (1H, d, *J* = 2.6 Hz, H-7), 7.13 (1H, dd, *J* = 8.6, 2.1 Hz, H-10), *7*.12 (1H, br s, H-4), 6.73 (2H, d, *J* = 8.3 Hz, 2H-3′), 5.22 (2H, s, H_2_-1′), ^13^C NMR (100 MHz, DMSO-*d*_6_) δ 138.6 (C-2), 137.8 (C-2′), 137.3 (2C-4′), 134.3 (C-8a), 128.6 (2C-3′), 127.8 (C-12a), 127.5 (C-4), 125.8 (C-11), 125.4 (C-5), 124.3 (C-7), 121.8 (C-10), 117.9 (C-12), 113.3 (C-9), 103.3 (C-6), 93.3 (C-5′), 47.2 (C-1′); (+)-HRESIMS *m/z* 433.9912 [M+H]^+^ (calcd for C_18_H_14_^35^ClIN_3_, 433.9915), *m/z* 435.9888 [M+H]^+^ (calcd for C_18_H_14_^37^ClIN_3_, 435.9891). 

#### 3.2.9. 6-Chloro-3-(1-(4-iodobenzyl)-1*H*-imidazol-5-yl)-1*H*-indole (**8**) 

Using the general procedure, reaction of 6-chloro-1*H*-indole-3-carbaldehyde (0.179 g, 1.0 mmol) with 4-iodobenzylamine (0.233 g, 1.0 mmol), K_2_CO_3_ (0.138 g, 1.0 mmol) and *p*-toluenesulfonylmethyl isocyanide (0.195 g, 1.0 mmol) in DMF (1 mL) followed by purification by trituration from CH_2_Cl_2_ afforded the title compound as a brown solid (0.079 g, 18%). *R_f_* = 0.68 (CH_2_Cl_2_/MeOH, 9:1); m.p > 200 °C; IR (ATR) ν_max_ 2775, 1610, 1594, 1512, 1300, 1240, 1112, 828, 796 cm^−1^; ^1^H NMR (400 MHz, DMSO*-d*_6_) δ 11.44 (1H, br s, NH-8), 7.83 (1H, d, *J* = 1.0 Hz, H-2), 7.61 (2H, d, *J* = 8.5 Hz, 2H-4′), 7.49 (1H, d, *J* = 8.5 Hz, H-12), 7.45 (1H, d, *J* = 2.0 Hz, H-9), 7.31 (1H, d, *J* = 2.7 Hz, H-7), 7.12 (1H, d, *J* = 1.0 Hz, H-4), *7*.05 (1H, dd, *J* = 8.5, 2.0 Hz, H-11), 6.74 (2H, d, *J* = 8.5 Hz, 2H-3′), 5.24 (2H, s, H_2_-1′), ^13^C NMR (100 MHz, DMSO-*d*_6_) δ 138.5 (C-2), 137.7 (C-2′), 137.3 (2C-4′), 136.2 (C-8a), 128.6 (2C-3′), 127.7 (C-4), 126.4 (C-10), 125.6 (C-12a), 125.1 (C-5), 124.9 (C-7), 120.3 (C-12), 119.9 (C-11), 111.3 (C-9), 103.8 (C-6), 93.2 (C-5′), 47.2 (C-1′); (+)-HRESIMS *m/z* 455.9728 [M+Na]^+^ (calcd for C_18_H_13_^35^ClIN_3_Na, 455.9735), *m/z* 457.9711 [M+Na]^+^ (calcd for C_18_H_13_^37^ClIN_3_Na, 457.9711). 

#### 3.2.10. 5-Bromo-3-(1-(4-iodobenzyl)-1*H*-imidazol-5-yl)-1*H*-indole (**9**) 

Using the general procedure, reaction of 5-bromo-1*H*-indole-3-carbaldehyde (0.224 g, 1.0 mmol) with 4-iodobenzylamine (0.233 g, 1.0 mmol), K_2_CO_3_ (0.138 g, 1.0 mmol) and *p*-toluenesulfonylmethyl isocyanide (0.195 g, 1.0 mmol) in DMF (1 mL) followed by purification by trituration from CH_2_Cl_2_ afforded the title compound as an orange solid (0.014 g, 3%). *R_f_* = 0.51 (CH_2_Cl_2_/MeOH, 9:1); m.p > 200 °C; IR (ATR) ν_max_ 3031, 283, 2720, 1623, 1588, 1456, 1238, 1116, 879, 792 cm^−1^; ^1^H NMR (400 MHz, DMSO*-d*_6_) δ 11.53 (1H, br s, NH-8), 7.85 (1H, d, *J* = 1.0 Hz, H-2), 7.61 (2H, d, *J* = 8.5 Hz, 2H-4′), 7.48 (1H, d, *J* = 2.0 Hz, H-12), 7.38 (1H, d, *J* = 8.5 Hz, H-9), 7.34 (1H, d, *J* = 1.0 Hz, H-7), 7.24 (1H, dd, *J* = 8.5, 2.0 Hz, H-10), 7.11 (1H, d, *J* = 1.0 Hz, H-4), 6.73 (2H, d, *J* = 8.5 Hz, 2H-3′), 5.21 (2H, d, H_2_-1′); ^13^C NMR (100 MHz, DMSO-*d*_6_) δ 138.6 (C-2), 137.7 (C-2′), 137.2 (2C-4′), 134.5 (C-8a), 128.5 (2C-3′), 128.2 (C-4), 127.9 (C-12a), 125.7 (C-5), 124.2 (C-10), 120.9 (C-12), 113.8 (C-9), 112.2 (C-11), 103.1 (C-6), 93.3 (C-5′), 47.2 (C-1′); (+)-HRESIMS *m/z* 477.9411 [M+H]^+^ (calcd for C_18_H_14_^79^BrIN_3_, 477.9411), *m/z* 479.9391 [M+H]^+^ (calcd for C_18_H_14_^81^BrIN_3_, 479.9392). 

#### 3.2.11. 6-Bromo-3-(1-(4-iodobenzyl)-1*H*-imidazol-5-yl)-1*H*-indole (**10**) 

Using the general procedure, reaction of 6-bromo-1*H*-indole-3-carbaldehyde (0.224 g, 1.0 mmol) with 4-iodobenzylamine (0.233 g, 1.0 mmol), K_2_CO_3_ (0.138 g, 1.0 mmol) and *p*-toluenesulfonylmethyl isocyanide (0.195 g, 1.0 mmol) in DMF (1 mL) followed by purification by trituration from CH_2_Cl_2_ afforded the title compound as a light brown powder (0.177 g, 37%). *R_f_* = 0.71 (CH_2_Cl_2_/MeOH, 9:1); m.p > 200 °C; IR (ATR) ν_max_ 2831, 1621, 1589, 1484, 1456, 1432, 1225, 1116, 982, 794 cm^−1^; ^1^H NMR (400 MHz, DMSO*-d*_6_) δ 11.44 (1H, br s, NH-8), 7.83 (1H, d, *J* = 0.8 Hz, H-2), 7.61 (2H, d, *J* = 8.3 Hz, 2H-4′), 7.60 (1H, d, *J* = 1.8 Hz, H-9), 7.44 (1H, d, *J* = 8.7 Hz, H-12), 7.30 (1H, d, *J* = 2.5 Hz, H-7), 7.16 (1H, dd, *J* = 8.6, 1.8 Hz, H-11), 7.12 (1H, d, *J* = 0.8 Hz, H-4), 6.73 (2H, d, *J* = 8.3 Hz, 2H-3′), 5.24 (2H, s, H_2_-1′); ^13^C NMR (100 MHz, DMSO-*d*_6_) δ 138.5 (C-2′), 137.7 (C-2), 137.2 (2C-4′), 136.7 (C-8a), 128.6 (2C-3′), 127.7 (C-4), 125.5 (C-5), 125.3 (C-12a), 124.8 (C-7), 122.5 (C-11), 120.6 (C-12), 114.4 (C-10), 114.3 (C-9), 103.8 (C-6), 93.2 (C-5′), 47.2 (C-1′); (+)-HRESIMS *m/z* 499.9211 [M+Na]^+^ (calcd for C_18_H_13_^79^BrIN_3_Na, 499.9230), *m/z* 501.9211 [M+Na]^+^ (calcd for for C_18_H_13_^81^BrIN_3_Na, 501.9211). 

#### 3.2.12. 3-(1-(4-Methoxybenzyl)-1*H*-imidazol-5-yl)-1*H*-indole (**11**) 

Using the general procedure, reaction of 1*H*-indole-3-carbaldehyde (0.145 g, 1.0 mmol) with 4- methoxybenzylamine (0.13 mL, 1.0 mmol), K_2_CO_3_ (0.138 g, 1.0 mmol) and *p*-toluenesulfonylmethyl isocyanide (0.195 g, 1.0 mmol) in DMF (1 mL) followed by purification using silica gel column chromatography (1–10% CH_2_Cl_2_/MeOH, 99:1→9:1) afforded the title compound as a brown solid (0.043 g, 14%). *R_f_ =* 0.42 (CH_2_Cl_2_/MeOH, 9:1); m.p 159–160 °C; IR (ATR) ν_max_ 3411, 2959, 1640, 1512, 1298, 1255, 1030, 909, 842, 784 cm^−1^; ^1^H NMR (400 MHz, DMSO-*d*_6_) δ 11.37 (1H, br s, NH-8), 7.78 (1H, d, *J* = 0.9 Hz, H-2), 7.54 (1H, d, *J* = 8.0 Hz, H-12), 7.43 (1H, d, *J* = 8.0 Hz, H-9), 7.32 (1H, d, *J* = 2.7 Hz, H-7), 7.15 (1H, ddd, *J* = 8.0, 7.5, 1.0 Hz, H-10), 7.11 (1H, d, *J* = 0.9 Hz, H-4), 7.06 (1H, ddd, *J* = 8.0, 7.5, 1.0 Hz, H-11), 6.92 (2H, d, *J* = 8.7 Hz, 2H-3′), 6.82 (2H, d, *J* = 8.7 Hz, 2H-4′), 5.20 (2H, s, H_2_-1′), 3.68 (3H, s, OMe); ^13^C NMR (100 MHz, DMSO-*d*_6_) δ 158.5 (C-5′), 138.1 (C-2), 136.0 (C-8a), 129.8 (C-2′), 127.9 (2C-3′), 127.2 (C-4, C-5), 126.3 (C-12a), 123.9 (C-7), 121.7 (C-10), 119.6 (C-11), 118.9 (C-12), 114.0 (2C-4′), 111.8 (C-9), 103.7 (C-6), 55.0 (OMe), 47.3 (C-1′); (+)-HRESIMS *m/z* 304.1436 [M+H]^+^ (calcd for C_19_H_18_N_3_O, 304.1444). 

#### 3.2.13. 5-Fluoro-3-(1-(4-methoxybenzyl)-1*H*-imidazol-5-yl)-1*H*-indole (**12**) 

Using the general procedure, reaction of 5-fluoro-1*H*-indole-3-carbaldehyde (0.163 g, 1.0 mmol) with 4-methoxybenzylamine (0.13 mL, 1.0 mmol), K_2_CO_3_ (0.138 g, 1.0 mmol) and *p*-toluenesulfonylmethyl isocyanide (0.195 g, 1.0 mmol) in DMF (1 mL) followed by purification using silica gel column chromatography (1–10% CH_2_Cl_2_/MeOH, 99:1→9:1) afforded the title compound as a yellow powder (0.090 g, 28%). *R_f_* = 0.54 (CH_2_Cl_2_/MeOH, 9:1); m.p 181–182 °C; IR (ATR) ν_max_ 3155, 2835, 2661m 1610, 1583, 1509, 1347, 1240, 1032, 970, 837, 790 cm^−1^; ^1^H NMR (400 MHz, DMSO-*d*_6_) δ 11.39 (1H, br s, NH-8), 7.77 (1H, d, *J* = 1.0 Hz, H-2), 7.49 (1H, dd, *J* = 8.9, 5.5 Hz, H-9), 7.31 (1H, d, *J* = 2.5 Hz, H-7), 7.20 (1H, dd, *J* = 10.0, 2.5 Hz, H-12), 7.10 (1H, d, *J* = 1.0 Hz, H-4), 6.93–6.88 (3H, m, H-10, 2H-3′), 6.82 (2H, d, *J* = 8.8 Hz, 2H-4′), 5.18 (2H, s, H_2_-1′), 3.68 (3H, s, OMe); ^13^C NMR (100 MHz, DMSO-*d*_6_) δ 158.9 (d, ^1^*J*_CF_ = 236.3 Hz, C-11), 158.5 (C-5′), 138.2 (C-2), 135.8 (d, ^3^*J*_CF_ = 12.5 Hz, C-12a), 129.7 (C-2′), 127.8 (2C-3′), 127.4 (C-4), 125.8 (C-5), 124.4 (C-7), 123.1 (C-8a), 120.0 (d, ^3^*J*_CF_ = 10.1 Hz, C-9), 113.9 (2C-4′), 108.1 (d, ^2^*J*_CF_ = 24.6 Hz, C-10), 103.9 (C-6), 97.7 (d, ^2^*J*_CF_ = 25.3 Hz, C-12), 55.0 (OMe), 47.2 (C-1′); (+)-HRESIMS *m/z* 322.1350 [M+H]^+^ (calcd for C_19_H_17_FN_3_O, 322.1350). 

#### 3.2.14. 6-Fluoro-3-(1-(4-methoxybenzyl)-1*H*-imidazol-5-yl)-1*H*-indole (**13**) 

Using the general procedure, reaction of 6-fluoro-1*H*-indole-3-carbaldehyde (0.163 g, 1.0 mmol) with 4-methoxybenzylamine (0.13 mL, 1.0 mmol), K_2_CO_3_ (0.138 g, 1.0 mmol) and *p*-toluenesulfonylmethyl isocyanide (0.195 g, 1.0 mmol) in DMF (1 mL) followed by purification using silica gel column chromatography (1–10% CH_2_Cl_2_/MeOH, 99:1→9:1) afforded the title compound as a brown solid (0.224 g, 70%). *R_f_* =0.22 (CH_2_Cl_2_/MeOH, 9:1); m.p 181–182 °C; IR (ATR) ν_max_ 3045, 2927, 2834, 1739, 1628, 1584, 1510, 1348, 1232, 1032, 837, 771 cm^−1^; ^1^H NMR (400 MHz, DMSO*-d*_6_) δ 11.44 (1H, br s, NH-8), 7.80 (1H, d, *J* = 0.63 Hz, H-2), 7.51 (1H, dd, *J* = 8.8, 5.6 Hz, H-12), 7.33 (1H, d, *J* = 2.5 Hz, H-7), 7.22 (1H, dd, *J* = 9.9, 2.3 Hz, H-9), 7.13 (1H, d, *J* = 0.63 Hz, H-4), 6.94–6.89 (3H, m, H-11, 2H-3′), 6.82 (2H, d, *J* = 8.6 Hz, 2H-4′), 5.20 (2H, s, H_2_-1′), 3.68 (3H, s, OMe); ^13^C NMR (100 MHz, DMSO-*d*_6_) δ 159 (d, ^1^*J*_CF_ = 235.7 Hz, C-10), 158.5 (C-5′), 138.2 (C-2), 135.9 (d, ^3^*J*_CF_ = 12.9 Hz, C-8a), 129.7 (C-2′), 127.8 (2C-3′), 127.4 (C-4), 125.8 (C-5), 124.4 (C-7), 123.3 (C-12a), 120.1 (d, ^3^*J*_CF_ = 10.3 Hz, C-12), 113.9 (2C-4′), 108.2 (d, ^2^*J*_CF_ = 24.4 Hz, C-11), 103.9 (C-6), 97.7 (d, ^2^*J*_CF_ = 25.6 Hz, C-9), 54.9 (OMe), 47.3 (C-1′); (+)-HRESIMS *m/z* 322.1348 [M+H]^+^ (calcd for C_19_H_17_FN_3_O, 322.1350). 

#### 3.2.15. 5-Chloro-3-(1-(4-methoxybenzyl)-1*H*-imidazol-5-yl)-1*H*-indole (**14**) 

Using the general procedure, reaction of 5-chloro-1*H*-indole-3-carbaldehyde (0.179 g, 1.0 mmol) with 4-methoxybenzylamine (0.13 mL, 1.0 mmol), K_2_CO_3_ (0.138 g, 1.0 mmol) and *p*-toluenesulfonylmethyl isocyanide (0.195 g, 1.0 mmol) in DMF (1 mL) followed by purification by trituration from CH_2_Cl_2_ afforded the title compound as a pale brown solid (0.156 g, 46%). *R_f_* = 0.49 (CH_2_Cl_2_/MeOH, 9:1); m.p 193–194 °C; IR (ATR) ν_max_ 3073, 3018, 2843, 1609, 1510, 1458, 1433, 1360, 1246, 1117, 1030, 893, 807, 764 cm^−1^; ^1^H NMR (400 MHz, DMSO*-d*_6_) δ 11.56 (1H, br s, NH-8), 7.80 (1H, d, *J* = 0.9 Hz, H-2), 7.44 (1H, d, *J* = 8.7 Hz, H-9), 7.40 (1H, d, *J* = 2.9 Hz, H-7), 7.39 (1H, d, *J* = 2.2 Hz, H-12), 7.14 (1H, dd, *J* = 8.7, 2.2 Hz, H-10), 7.10 (1H, d, *J* = 0.9 Hz, H-4), 6.89 (2H, d, *J* = 8.5 Hz, 2H-3′), 6.81 (2H, d, *J* = 8.5 Hz, 2H-4′), 5.17 (2H, s, H_2_-1′), 3.68 (3H, s, OMe); ^13^C NMR (100 MHz, DMSO-*d*_6_) δ 158.5 (C-5′), 138.3 (C-2), 134.4 (C-8a), 129.6 (C-2′), 127.8 (2C-3′), 127.6 (C-4), 127.4 (C-12a), 125.8 (C-7), 125.4 (C-5), 124.3 (C-11), 121.7 (C-10), 113.9 (2C-4′), 113.3 (C-9), 103.5 (C-6), 55.0 (OMe), 47.2 (C-1′); (+)-HRESIMS *m/z* 338.1056 [M+H]^+^ (calcd for C_19_H_17_^35^ClN_3_O, 388.1055), *m/z* 340.1039 [M+H]^+^ (calcd for C_19_H_17_^37^ClN_3_O, 340.1031). 

#### 3.2.16. 6-Chloro-3-(1-(4-methoxybenzyl)-1*H*-imidazol-5-yl)-1*H*-indole (**15**) 

Using the general procedure, reaction of 6-chloro-1*H*-indole-3-carbaldehyde (0.179 g, 1.0 mmol) with 4-methoxybenzylamine (0.13 mL, 1.0 mmol), K_2_CO_3_ (0.138 g, 1.0 mmol) and *p*-toluenesulfonylmethyl isocyanide (0.195 g, 1.0 mmol) in DMF (1 mL) followed by purification using silica gel column chromatography (1–10% CH_2_Cl_2_/MeOH, 99:1→9:1) afforded the title compound as a brown solid (0.183 g, 54%). *R_f_* = 0.64 (CH_2_Cl_2_/MeOH, 9:1); m.p 178–179 °C; IR (ATR) ν_max_ 3080, 2835, 1612, 1588, 1512, 1456, 1248, 1230, 918, 795 cm^−1^; ^1^H NMR (400 MHz, DMSO*-d*_6_) δ 11.46 (1H, br s, NH-8), 7.78 (1H, d, *J* = 0.9 Hz, H-2), 7.50 (1H, d, *J* = 8.7 Hz, H-12), 7.46 (1H, d, *J* = 2.0 Hz, H-9), 7.36 (1H, d, *J* = 2.5 Hz, H-7), 7.10 (1H, d, *J* = 0.9 Hz, H-4), 7.05 (1H, dd, *J* = 8.7, 2.0 Hz, H-11), 6.90 (2H, d, *J* = 9.0 Hz, 2H-3′), 6.81 (2H, d, *J* = 9.0 Hz, 2H-4′), 5.18 (2H, s, H_2_-1′), 3.68 (3H, s, OMe); ^13^C NMR (100 MHz, DMSO-*d*_6_) δ 158.5 (C-5′), 138.3 (C-11), 136.3 (C-8a), 129.7 (C-2′), 127.9 (2C-3′), 127.5 (C-4), 126.4 (C-10), 125.6 (C-5), 125.1 (C-12a), 124.9 (C-7), 120.3 (C-12), 119.9 (C-11), 114.0 (2C-4′), 111.3 (C-9), 104.0 (C-6), 55.0 (OMe), 47.3 (C-1′); (+)-HRESIMS *m/z* 388.1050 [M+H]^+^ (calcd for C_19_H_17_^35^ClN_3_O, 388.1055), *m/z* 340.1033 [M+H]^+^ (calcd for C_19_H_17_^37^ClN_3_O, 340.1031). 

#### 3.2.17. 5-Bromo-3-(1-(4-methoxybenzyl)-1*H*-imidazol-5-yl)-1*H*-indole (**16**) 

Using the general procedure, reaction of 5-bromo-1*H*-indole-3-carbaldehyde (0.224 g, 1.0 mmol) with 4-methoxybenzylamine (0.13 mL, 1.0 mmol), K_2_CO_3_ (0.138 g, 1.0 mmol) and *p*-toluenesulfonylmethyl isocyanide (0.195 g, 1.0 mmol) in DMF (1 mL) followed by purification using silica gel column chromatography (1–10% CH_2_Cl_2_/MeOH, 99:1→9:1) afforded the title compound as a white powder (0.025 g, 7%). *R_f_* = 0.66 (CH_2_Cl_2_/MeOH, 9:1); m.p 197–198 °C; IR (ATR) ν_max_ 2999, 2837, 1610, 1509, 1454, 1248, 1106, 915, 803 cm^−1^; ^1^H NMR (400 MHz, DMSO-*d*_6_) δ11.55 (1H, br s, NH-8), 7.80 (1H, d, *J* = 1.0 Hz, H-2), 7.51 (1H, d, *J* = 1.9 Hz, H-12), 7.39 (1H, d, *J* = 8.7 Hz, H-9), 7.38 (1H, s, H-7), 7.25 (1H, dd, *J* = 8.7, 1.9 Hz, H-10), 7.08 (1H, d, *J* = 1.0 Hz, H-4), 6.89 (2H, d, *J* = 8.7 Hz, 2H-3′), 6.80 (2H, d, *J* = 8.7 Hz, 2H-4′), 5.16 (2H, s, H_2_-1′), 3.70 (3H, s, OMe); ^13^C NMR (100 MHz, DMSO-*d*_6_) δ 158.5 (C-5′), 138.3 (C-2), 134.6 (C-8a), 129.6 (C-2′), 128.3 (C-12a), 127.9 (2C-3′), 127.7 (C-4), 125.7 (C-7), 125.3 (C-5), 124.2 (C-10), 121.0 (C-12), 113.9 (2C-4′), 113.8 (C-9), 112.2 (C-11), 103.4 (C-6), 55.0 (OMe), 47.2 (C-1′); (+)-HRESIMS *m/z* 404.0356 [M+Na]^+^ (calcd for C_19_H_16_^79^BrN_3_NaO, 404.0369), *m/z* 406.0322 [M+Na]^+^ (calcd for C_19_H_16_^81^BrN_3_NaO, 406.0350).

#### 3.2.18. 6-Bromo-3-(1-(4-methoxybenzyl)-1*H*-imidazol-5-yl)-1*H*-indole (**17**) 

Using the general procedure, reaction of 6-bromo-1*H*-indole-3-carbaldehyde (0.224 g, 1.0 mmol) with 4-methoxybenzylamine (0.13 mL, 1.0 mmol), K_2_CO_3_ (0.138 g, 1.0 mmol) and *p*-toluenesulfonylmethyl isocyanide (0.195 g, 1.0 mmol) in DMF (1 mL) followed by purification by trituration from CH_2_Cl_2_ afforded the title compound as a light brown powder (0.120 g, 32%). *R_f_* = 0.65 (CH_2_Cl_2_/MeOH, 9:1); m.p 188–189 °C; IR (ATR) ν_max_ 3081, 3023, 2834, 2646, 1612, 1588, 1213, 1454, 1224, 1249, 1225, 842, 788 cm^−1^; ^1^H NMR (400 MHz, DMSO*-d*_6_) δ 11.47 (1H, br s, NH-8), 7.78 (1H, s, H-2), 7.60 (1H, d, *J* = 1.8 Hz, H-9), 7.45 (1H, d, *J* = 8.6 Hz, H-12), 7.35 (1H, d, *J* = 2.6 Hz, H-7), 7.17 (1H, dd, *J* = 8.6, 1.8 Hz, H-11), 7.10 (1H, s, H-4), 6.90 (2H, d, *J* = 8.7 Hz, 2H-3′), 6.81 (2H, d, *J* = 8.7 Hz, 2H-4′), 5.18 (2H, s, H_2_-1′), 3.68 (3H, s, OMe); ^13^C NMR (100 MHz, DMSO-*d*_6_) δ 158.4 (C-5′), 138.3 (C-2), 136.7 (C-8a), 129.6 (C-2′), 127.8 (2C-3′), 127.5 (C-4), 125.5 (C-5), 125.4 (C-12a), 124.9 (C-7), 122.4 (C-11), 120.7 (C-12), 114.4 (C-10), 114.3 (C-9), 113.9 (2C-4′), 104.0 (C-6), 55.0 (OMe), 47.2 (C-1′); (+)-HRESIMS *m/z* 404.0369 [M+Na]^+^ (calcd for C_19_H_16_^79^BrN_3_NaO, 404.0357), *m/z* 406.0332 [M+Na]^+^ (calcd for C_19_H_16_^81^BrN_3_NaO, 406.0350). 

#### 3.2.19. 6-Fluoro-3-(1-phenethyl-1*H*-imidazol-5-yl)-1*H*-indole (**18**) 

Using the general procedure, reaction of 6-fluoro-1*H*-indole-3-carbaldehyde (163.0 mg, 1.0 mmol) with phenethylamine (0.12 mL, 1.0 mmol), K_2_CO_3_ (0.138 g, 1.0 mmol) and *p*-toluenesulfonylmethyl isocyanide (0.195 g, 1.0 mmol) in DMF (1 mL) followed by purification using silica gel column chromatography (1–10% CH_2_Cl_2_/MeOH, 99:1→9:1) afforded the title compound as a brown oil (0.053 g, 17%). *R_f_* = 0.63 (CH_2_Cl_2_/MeOH, 9:1); IR (ATR) ν_max_ 3083, 2901, 1628, 1588, 1506, 1452, 1344, 1228, 1149, 1112, 800, 751 cm^−1^; ^1^H NMR (400 MHz, DMSO*-d*_6_) δ 11.49 (1H, br s, NH-8), 7.60 (1H, s, H-2), 7.48 (1H, d, *J* = 2.5 Hz, H-7), 7.46 (1H, dd, *J* = 8.8, 5.3 Hz, H-12), 7.25–7.19 (3H, m, H-9, 2H-5′), 7.19–7.16 (1H, m, H-6′), 7.01 (1H, s, H-4), 6.99 (2H, dd, *J* = 6.8, 1.5 Hz, 2H-4′), 6.92 (1H, ddd, *J* = 9.9, 8.8, 2.3 Hz, H-11), 4.20 (2H, t, *J* = 7.5 Hz, H_2_-1′), 2.83 (2H, t, *J* = 7.5 Hz, H_2_-2′); ^13^C NMR (100 MHz, DMSO-*d*_6_) δ 158.9 (d, ^1^*J*_CF_ = 235.9, C-10), 138.0 (C-3′), 137.8 (C-2), 135.8 (d, ^3^*J*_CF_ = 12.6 Hz, C-8a), 128.5 (2C-4′), 128.3 (2C-5′), 127.4 (C-4), 126.4 (C-6′), 125.3 (C-5), 124.8 (C-7), 123.3 (C-12a), 119.9 (d, ^3^*J*_CF_ = 10.8 Hz, C-12), 108.1 (d, ^2^*J*_CF_ = 24.6, C-11), 104.0 (C-6), 97.7 (d, ^2^*J*_CF_ = 25.3, C-9), 45.8 (C-1′), 36.2 (C-2′); (+)-HRESIMS *m/z* 306.1390 [M+Na]^+^ (calcd for C_19_H_29_FNaO, 306.1395).

#### 3.2.20. 5-Chloro-3-(1-phenethyl-1*H*-imidazol-5-yl)-1*H*-indole (**19**) 

Using the general procedure, reaction of 5-chloro-1*H*-indole-3-carbaldehyde (0.179 g, 1.0 mmol) with phenethylamine (0.12 mL, 1.0 mmol), K_2_CO_3_ (0.138 g, 1.0 mmol) and *p*-toluenesulfonylmethyl isocyanide (0.195 g, 1.0 mmol) in DMF (1 mL) followed by purification by trituration from CH_2_Cl_2_ afforded the title compound as a pale yellow powder (0.149 g, 46%). *R_f_* = 0.62 (CH_2_Cl_2_/MeOH, 9:1); m.p > 200 °C; IR (ATR) ν_max_ 3016, 2831, 1628, 1481, 1436, 1227, 1111, 921, 886, 793 cm^−1^; ^1^H NMR (400 MHz, DMSO*-d*_6_) δ 11.63 (1H, br s, NH-8), 7.63 (1H, s, H-2), 7.56, (1H, d, *J* = 2.5 Hz, H-7), 7.48 (1H, d, *J* = 8.3 Hz, H-9), 7.42 (1H, d, *J =* 1.8 Hz, H-12), 7.23– 7.19 (3H, m, 2H-5′, H-6′), 7.19–7.15 (1H, m, H-10), 6.99–6.97 (3H, m, H-4, 2H-4′), 4.20 (2H, t, *J* = 7.6 Hz, H_2_-1′), 2.83 (2H, t, *J* = 7.6 Hz, H_2_-2′); ^13^C NMR (100 MHz, DMSO-*d*_6_) δ 137.9 (C-3′), 137.8 (C-2), 134.4 (C-8a), 128.5 (2C-5′), 128.3 (2C-4′), 127.7 (C-12a), 127.5 (C-7), 126.4 (C-6′), 126.1 (C-4), 124.9 (C-5), 124.3 (C-11), 121.7 (C-10), 117.8 (C-12), 113.4 (C-9), 103.6 (C-6), 45.7 (C-1′), 36.2 (C-2′); (+)-HRESIMS *m/z* 344.0925 [M+Na]^+^ (calcd for C_19_H_16_^35^ClN_3_Na, 344.0925), *m/z* 346.0893 [M+Na]^+^ (calcd for C_19_H_16_^37^ClN_3_Na, 346.0893). 

#### 3.2.21. 6-Chloro-3-(1-phenethyl-1*H*-imidazol-5-yl)-1*H*-indole (**20**) 

Using the general procedure, reaction of 6-chloro-1*H*-indole-3-carbaldehyde (0.179 g, 1.0 mmol) with phenethylamine (0.12 mL, 1.0 mmol), K_2_CO_3_ (0.138 g, 1.0 mmol) and *p*-toluenesulfonylmethyl isocyanide (0.195 g, 1.0 mmol) in DMF (1 mL) followed by purification using silica gel column chromatography (1–10% CH_2_Cl_2_/MeOH, 99:1→9:1) afforded the title compound as a dark brown solid (0.145 g, 45%). *R_f_* = 0.69 (CH_2_Cl_2_/MeOH, 9:1); m.p 91–92 °C; IR (ATR) ν_max_ 3136, 2829, 2629, 1610, 1509, 1440, 1241, 1131, 823, 793 cm-1; ^1^H NMR (400 MHz, DMSO*-d*_6_) δ 11.56 (1H, br s, NH-8), 7.61 (1H, s, H-2), 7.52 (1H, d, *J* = 2.6 Hz, H-7), 7.50 (1H, d, *J* = 1.9 Hz, H-9), 7.47 (1H, d, *J* = 8.7 Hz, H-12), 7.24–7.20 (2H, m, 2H-5′), 7.20–7.16 (1H, m, H-6′), 7.07 (1H, dd, *J* = 8.9, 1.9 Hz, H-11), 7.00–6.99 (3H, m, 2H-4′, H-4), 4.20 (2H, t, *J* = 7.4 Hz, H_2_-1′), 2.83 (2H, t, *J* = 7.6 Hz, H_2_-2′); ^13^C NMR (100 MHz, DMSO-*d*_6_) δ 138.0 (C-3′), 137.8 (C-2), 136.4 (C-8a), 128.5 (2C-4′), 128.3 (2C-5′), 127.5 (C-4), 126.4 (C-6′, C-10), 125.4 (C-12a), 125.3 (C-7), 125.1 (C-5), 120.2 (C-12), 119.9 (C-11), 104.1 (C-6), 45.8 (C-1′), 36.2 (C-2′); (+)-HRESIMS *m/z* 322.1114 [M+H]^+^ (calcd for C_19_H_17_^35^ClN_3_, 322.1106), *m/z* 324.1085 [M+H]^+^ (calcd for C_19_H_17_^37^ClN_3_, 324.1082). 

#### 3.2.22. 5-Bromo-3-(1-phenethyl-1*H*-imidazol-5-yl)-1*H*-indole (**21**) 

Using the general procedure, reaction of 5-bromo-1*H*-indole-3-carbaldehyde (0.224 g, 1.0 mmol) with phenethylamine (0.12 mL, 1.0 mmol), K_2_CO_3_ (0.138 g, 1.0 mmol) and *p*-toluenesulfonylmethyl isocyanide (0.195 g, 1.0 mmol) in DMF (1 mL) followed by purification using silica gel column chromatography (1–10% CH_2_Cl_2_/MeOH, 99:1→9:1) afforded the title compound as a brown solid (0.092 g, 25%). *R_f_* = 0.41 (CH_2_Cl_2_/MeOH, 9:1); m.p 110–111 °C; IR (ATR) ν_max_ 3404, 2921, 2256, 1627, 1507, 1453, 816, 791 cm^−1^; ^1^H NMR (400 MHz, DMSO-*d*_6_) δ 11.70 (1H, s, NH-8), 7.65 (1H, s, H-2), 7.59 (1H, d, *J* = 1.6 Hz, H-12), 7.55 (1H, d, *J* = 1.9 Hz, H-7), 7.45 (1H, d, *J* = 8.5 Hz, H-9), 7.28 (1H, dd, *J* = 8.5, 1.6 Hz, H-10), 7.22–7.15 (3H, m, 2H-5′, H-6′), 7.02 (1H, s, H-4), 6.98 (2H, d, *J* = 7.4 Hz, 2H-4′), 4.20 (2H, t, *J* = 7.2 Hz, H_2_-1′), 2.84 (2H, t, *J* = 7.2 Hz, H_2_-2′); ^13^C NMR (100 MHz, DMSO-*d*_6_) δ 137.9 (C-2/C-3), 137.8 (C-2/C-3′), 134.7 (C-8a), 128.5 (2C-4′), 128.4 (C-12a), 128.3 (2C-5′), 127.6 (C-4), 126.4 (C-6′), 126.0 (C-7), 124.9 (C-5), 124.2 (C-10), 121.0 (C-12), 113.8 (C-9), 112.2 (C-11), 103.5 (C-6), 45.8 (C-1′), 36.2 (C-2′); (+)-HRESIMS *m/z* 366.0599 [M+H]^+^ (calcd for C_19_H_17_^79^BrN_3_, 366.0600), *m/z* 368.0576 [M+H]^+^ (calcd for C_19_H_17_^81^BrN_3_, 368.0576). 

#### 3.2.23. 6-Bromo-3-(1-phenethyl-1*H*-imidazol-5-yl)-1*H*-indole (**22**) 

Using the general procedure, reaction of 6-bromo-1*H*-indole-3-carbaldehyde (0.224 g, 1.0 mmol) with phenethylamine (0.12 mL, 1.0 mmol), K_2_CO_3_ (0.138 g, 1.0 mmol) and *p*-toluenesulfonylmethyl isocyanide (0.195 g, 1.0 mmol) in DMF (1 mL) followed by purification using silica gel column chromatography (1–10% CH_2_Cl_2_/MeOH, 99:1→9:1) afforded the title compound as an brown oil (0.158 g, 43%). *R_f_* = 0.56 (CH_2_Cl_2_/MeOH, 9:1); IR (ATR) ν_max_ 3086, 2247, 1595, 1453, 1332, 1108, 894, 727 cm^−1^; ^1^H NMR (400 MHz, DMSO*-d*_6_) δ 11.56 (1H, br s, NH-8), 7.64 (1H, d, *J* = 1.4 Hz, H-9), 7.61 (1H, d, *J* = 0.7 Hz, H-2), 7.51 (1H, d, *J* = 2.7 Hz, H-7), 7.43 (1H, d, *J* = 8.5 Hz, H-12), 7.24–7.19 (2H, m, 2H-4′), 7.18–7.16 (2H, m, H-11, H-6′), 7.00–6.98 (3H, m, H-4, 2H-5′), 4.20 (2H, t, *J* = 7.2 Hz, H_2_-1′), 2.82 (2H, *J* = 7.2 Hz, H_2_-2′); ^13^C NMR (100 MHz, DMSO-*d*_6_) δ 138.0 (C-2), 137.8 (C-3′), 136.8 (C-8a), 128.5 (2C-4′), 128.3 (2C-5′), 127.5 (C-7), 126.4 (C-9), 125.6 (C-5), 125.2 (C-4), 125.0 (C-12a), 122.5 (C-11), 120.6 (C-12), 114.4 (C-10), 114.3 (C-6′), 104.1 (C-6), 45.8 (C-1′), 36.1 (C-2′); (+)-HRESIMS *m/z* 388.0422 [M+Na]^+^ (calcd for C_19_H_16_^79^BrN_3_Na, 388.0420), *m/z* 390.0400 [M+Na]^+^ (calcd for C_19_H_16_^81^BrN_3_Na, 390.0401). 

#### 3.2.24. 3-(1-(4-Methoxyphenethyl)-1*H*-imidazol-5-yl)-1*H*-indole (**23**) 

Using the general procedure, reaction of 1*H*-indole-3-carbaldehyde (0.145 g, 1.0 mmol) with 4-methoxy-phenethylamine (0.15 mL, 1.0 mmol), K_2_CO_3_ (0.138 g, 1.0 mmol) and *p*-toluenesulfonylmethyl isocyanide (0.195 g, 1.0 mmol) in DMF (1 mL) followed by purification using silica gel column chromatography (1–10% CH_2_Cl_2_/MeOH, 99:1→9:1) afforded the title compound as a brown solid (0.146 g, 46%). *R_f_ =* 0.59 (CH_2_Cl_2_/MeOH, 9:1); m.p 158–159 °C; IR (ATR) ν_max_ 3402, 2835, 1612, 1511, 1244, 1025, 916, 746 cm^−1^; ^1^H NMR (400 MHz, DMSO-*d*_6_) δ 11.46 (1H, s, NH-8), 7.60 (1H, s, H-2), 7.51–7.46 (3H, m, H-7, H-9, H-12), 7.17 (1H, ddd, *J* = 7.4, 7.4, 1.1 Hz, H-10), 7.07 (1H, ddd, *J* = 7.4, 7.4, 0.9 Hz, H-11), 7.00 (1H, s, H-4), 6.90 (2H, d, *J =* 8.7 Hz, 2H-4′), 6.77, (2H, d, *J* = 8.7 Hz, 2H-5′), 4.17 (2H, t, *J* = 7.5 Hz, H_2_-1′), 3.68 (3H, s, OMe), 2.78 (2H, t, *J* = 7.5 Hz, H_2_-2′); ^13^C NMR (100 MHz, DMSO-*d*_6_) δ 157.8 (C-6′), 137.6 (C-2), 136.0 (C-8a), 129.8 (C-3′), 129.5 (2C-4′), 127.2 (C-4), 126.5 (C-12a), 125.7 (C-5), 124.2 (C-7), 121.7 (C-10), 119.5 (C-11), 118.8 (C-12), 113.7 (2C-5′), 111.8 (C-9), 103.8 (C-6), 54.9 (OMe), 46.1 (C-1′), 35.3 (C-2′); (+)-HRESIMS *m/z* 318.1599 [M+H]^+^ (calcd for C_20_H_20_N_3_O, 318.1601).

#### 3.2.25. 5-Fluoro-3-(1-(4-methoxyphenethyl)-1*H*-imidazol-5-yl)-1*H*-indole (**24**) 

Using the general procedure, reaction of 5-fluoro-1*H*-indole-3-carbaldehyde (0.163 g, 1.0 mmol) with 4- methoxyphenethylamine (0.15 mL, 1.0 mmol), K_2_CO_3_ (0.138 g, 1.0 mmol) and *p*-toluenesulfonylmethyl isocyanide (0.195 g, 1.0 mmol) in DMF (1 mL) followed by purification using silica gel column chromatography (1–10% CH_2_Cl_2_/MeOH, 99:1→9:1) afforded the title compound as a yellow solid (0.255 g, 76%). *R_f_* = 0.70 (CH_2_Cl_2_/MeOH, 9:1); m.p 152–153 °C; IR (ATR) ν_max_ 3137, 2993, 2833, 1632, 1509, 1464, 1237, 1114, 849, 794 cm^−1^; ^1^H NMR (400 MHz, DMSO-*d*_6_) δ 11.53 (1H, br s, NH-8), 7.60 (1H, d, *J* = 1.0 Hz, H-2), 7.55 (1H, d, *J* = 2.4 Hz, H-7), 7.45 (1H, dd, *J* = 9.0, 4.7 Hz, H-9), 7.16 (1H, dd, *J* = 9.5, 2.7 Hz, H-12), 7.01 (1H, ddd, *J* = 9.5, 9.0, 2.7 Hz, H-10), 6.98 (1H, s, H-4), 6.89 (2H, d, *J* = 8.9 Hz, 2H-4′), 6.76 (2H, d, *J* = 8.9 Hz, 2H-5′), 4.15 (2H, t, *J* = 7.2 Hz, H_2_-1′), 3.68 (3H, s, OMe), 2.77 (2H, t, *J* = 7.2 Hz, H_2_-2′); ^13^C NMR (100 MHz, DMSO-*d*_6_) δ 157.8 (C-6′), 157.4 (d, ^1^*J*_CF_ = 230.8 Hz, C-11), 137.7 (C-2), 132.6 (C-8a), 129.8 (C-3′), 129.5 (2C-4′), 127.3 (C-4), 126.8 (d. ^3^*J*_CF_ = 10.5 Hz, C-12a), 126.2 (C-7), 125.2 (C-5), 113.7 (2C-5′), 112.9 (d, ^3^*J*_CF_ = 10.4 Hz, C-9), 109.9 (d, ^2^*J*_CF_ = 26.4 Hz, C-10), 104.1 (d, ^4^*J*_CF_ = 5.0 Hz, C-6), 103.4 (d, ^2^*J*_CF_ = 23.9 Hz, C-12), 54.9 (OMe), 46.0 (C-1′), 35.3 (C-2′); (+)-HRESIMS *m/z* 358.1334 [M+Na]^+^ (calcd for C_20_H_18_FN_3_NaO, 358.1326). 

#### 3.2.26. 6-Fluoro-3-(1-(4-methoxyphenethyl)-1*H*-imidazol-5-yl)-1*H*-indole (**25**) 

Using the general procedure, reaction of 6-fluoro-1*H*-indole-3-carbaldehyde (0.163 g, 1.0 mmol) with 4-methoxyphenethylamine (0.15 mL, 1.0 mmol), K_2_CO_3_ (0.138 g, 1.0 mmol) and *p*-toluenesulfonylmethyl isocyanide (0.195 g, 1.0 mmol) in DMF (1 mL) followed by purification using silica gel column chromatography (1–10% CH_2_Cl_2_/MeOH, 99:1→9:1) afforded the title compound as a brown oil (0.050 g, 15%). *R_f_* = 0.60 (CH_2_Cl_2_/MeOH, 9:1); IR (ATR) ν_max_ 3083, 2929, 2254, 1627, 1612, 1509, 1453, 1241, 1026, 820, 795 cm^−1^; ^1^H NMR (400 MHz, DMSO*-d*_6_) δ 11.49 (1H, br s, NH-8), 7.60 (1H, s, H-2), 7.48 (1H, d, *J* = 1.5 Hz, H-7), 7.46 (1H, dd, *J* = 8.5, 5.3 Hz, H-12), 7.24 (1H, d, *J* = 10.0 Hz, H-9), 7.00 (1H, s, H-4), 6.93 (1H, d, *J* = 8.5 Hz, H-11), 6.90 (2H, d, *J* = 8.3 Hz, 2H-4′), 6.77 (2H, d, *J* = 8.3 Hz, 2H-5′), 4.16 (2H, t, *J* = 7.4 Hz, H_2_-1′), 3.68 (3H, s, OMe), 2.77 (2H, t, *J* = 7.4 Hz, H_2_-2′); ^13^C NMR (100 MHz, DMSO-*d*_6_) δ 159.0 (d, ^1^*J*_CF_ = 229.0 Hz, C-10), 157.8 (C-6′), 137.8 (C-2), 135.9 (d, ^3^*J*_CF_ = 12.7 Hz, C-8a), 129.8 (C-3′), 129.5 (2C-4′), 127.4 (C-4), 125.3 (C-5), 124.7 (C-7), 123.4 (C-12a), 119.9 (d, ^3^*J*_CF_ = 10.2 Hz, C-12), 113.7 (2C-5′), 108.1 (d, ^2^*J*_CF_ = 25.0 Hz, C-11), 104.1 (C-6), 97.7 (d, ^2^*J*_CF_ = 25.5 Hz, H-9), 54.9 (OMe), 46.1 (C-1′), 35.3 (C-2′); (+)-HRESIMS *m/z* 358.1336 [M+Na]^+^ (calcd for C_20_H_18_FN_3_NaO, 358.1326). 

#### 3.2.27. 5-Chloro-3-(1-(4-methoxyphenethyl)-1*H*-imidazol-5-yl)-1*H*-indole (**26**) 

Using the general procedure, reaction of 5-chloro-1*H*-indole-3-carbaldehyde (0.179 g, 1.0 mmol) with 4-methoxyphenethylamine (0.15 mL, 1.0 mmol), K_2_CO_3_ (0.138 g, 1.0 mmol) and *p*-toluenesulfonylmethyl isocyanide (0.195 g, 1.0 mmol) in DMF (1 mL) followed by purification using silica gel column chromatography (1–10% CH_2_Cl_2_/MeOH, 99:1→9:1) afforded the title as a brown oil compound (0.038 g, 7%). *R_f_* = 0.56 (CH_2_Cl_2_/MeOH, 9:1); IR (ATR) ν_max_ 3136, 2253, 1656, 1612, 1511, 1459, 1244, 1110, 1024, 894, 797 cm^−1^; ^1^H NMR (400 MHz, DMSO*-d*_6_) δ 11.63 (1H, br s, NH-8), 7.61 (1H, d, *J* = 1.0 Hz, H-2), 7.55 (1H, d, *J* = 2.9 Hz, H-7), 7.48 (1H, d, *J* = 8.5 Hz, H-9), 7.42 (1H, d, *J* = 2.0 Hz, H-12), 7.16 (1H, dd, *J* = 8.5. 2.0 Hz, H-10), 6.99 (1H, d, *J* = 1.0 Hz, H-4), 6.89 (2H, d, *J* = 8.7 Hz, 2H-4′), 6.75 (2H, *J* = 8.7 Hz, 2H-5′), 4.15 (2H, t, *J* = 7.5 Hz, H_2_-1′), 3.68 (3H, s, OMe), 2.77 (2H, d, *J* = 7.5 Hz, H_2_-2′); ^13^C NMR (100 MHz, DMSO-*d*_6_), δ 157.8 (C-6′), 137.8 (C-2), 134.5 (C-8a), 129.8 (C-3′), 129.5 (2C-4′), 127.7 (C-4), 127.5 (C-12a), 126.1 (C-7), 125.0 (C-5), 124.3 (C-11), 121.7 (C-10), 117.9 (C-12), 113.7 (2C-5′), 113.4 (C-9), 103.7 (C-6), 54.9 (OMe), 46.0 (C-1′), 35.4 (C-2′); (+)-HRESIMS *m/z* 374.1021 [M+Na]^+^, (calcd for C_20_H_18_^35^ClN_3_NaO, 374.1031), *m/z* 376.1002 [M+Na]^+^, (calcd for C_20_H_18_^37^ClN_3_NaO, 376.1008). 

#### 3.2.28. 5-Bromo-3-(1-(4-methoxyphenethyl)-1*H*-imidazol-5-yl)-1*H*-indole (**27**) 

Using the general procedure, reaction of 5-bromo-1*H*-indole-3-carbaldehyde (0.224 g, 1.0 mmol) with 4-methoxyphenethylamine (0.15 mL, 1.0 mmol), K_2_CO_3_ (0.138 g, 1.0 mmol) and *p*-toluenesulfonylmethyl isocyanide (0.195 g, 1.0 mmol) in DMF (1 mL) followed by purification using silica gel column chromatography (1–10% CH_2_Cl_2_/MeOH, 99:1→9:1) afforded the title compound as a brown solid (0.192 g, 49%). *R_f_* = 0.76 (CH_2_Cl_2_/MeOH, 9:1); m.p 157–158 °C; IR (ATR) ν_max_ 3136, 2830, 1611, 1587, 1509, 1453, 1241, 1132, 1032, 824, 793 cm^−1^; ^1^H NMR (400 MHz, DMSO*-d*_6_) δ 11.64 (1H, br s, NH-8), 7.62 (1H, d, *J* = 1.0 Hz, H-2), 7.56 (1H, d, *J* = 2.0 Hz, H-12), 7.53 (1H, d, *J* = 2.7 Hz, H-7), 7.43 (1H, d, *J* = 8.8 Hz, H-9), 7.28 (1H, dd, *J* = 8.8, 2.0 Hz, H-10), 6.98 (1H, d, *J* = 1.0 Hz, H-4), 6.89 (2H, d, *J* = 8.4 Hz, 2H-4′), 6.76 (2H, d, *J* = 8.4 Hz, 2H-5′), 4.15 (2H, t, *J* = 7.3 Hz, H_2_-1′), 3.68 (3H, s, OMe), 2.76 (2H, t, *J* = 7.3 Hz, H_2_-2′); ^13^C NMR (100 MHz, DMSO-*d*_6_) δ 157.8 (C-6′), 137.8 (C-2), 134.6 (C-8a), 129.7 (C-3′), 129.5 (2C-4′), 128.4 (C-12a), 127.5 (C-4), 125.9 (C-7), 124.9 (C-5), 124.2 (C-10), 120.8 (C-12), 113.8 (C-9), 113.7 (2C-5′), 112.2 (C-11), 103.5 (C-6), 54.9 (OMe), 46.0 (C-1′), 35.3 (C-2′); (+)-HRESIMS *m/z* 418.0513 [M+Na]^+^ (calcd for C_20_H_18_^79^BrN_3_NaO, 418.0525), *m/z* 420.0491 [M+Na]^+^ (calcd for C_20_H_18_^81^BrN_3_NaO, 420.0507). 

#### 3.2.29. 6-Bromo-3-(1-(4-methoxyphenethyl)-1*H*-imidazol-5-yl)-1*H*-indole (**28**) 

Using the general procedure, reaction of 6-bromo-1*H*-indole-3-carbaldehyde (0.224 g, 1.0 mmol) with 4-methoxyphenethylamine (0.15 mL, 1.0 mmol), K_2_CO_3_ (0.138 g, 1.0 mmol) and *p*-toluenesulfonylmethyl isocyanide (0.195 g, 1.0 mmol) in DMF (1 mL) followed by purification using silica gel column chromatography (1–10% CH_2_Cl_2_/MeOH, 99:1→9:1) afforded the title compound as a yellow solid (0.187 g, 47%). *R_f_* = 0.40 (CH_2_Cl_2_/MeOH, 9:1); m.p 184–185 °C; IR (ATR) ν_max_ 2775, 1610, 1511, 1440, 1243, 1112, 827, 806 cm^−1^; ^1^H NMR (400 MHz, DMSO*-d*_6_) δ 11.55 (1H, br s, NH-8), 7.64 (1H, d, *J* = 1.8 Hz, H-9), 7.60 (1H, d, *J* = 1.0 Hz, H-2), 7.50 (1H, d, *J* = 2.6 Hz, H-7), 7.42 (1H, d, *J* = 8.6 Hz, H-12), 7.18 (1H, dd, *J* = 8.6, 1.8 Hz, H-11), 6.97 (1H, d, *J* = 1.0 Hz, H-4), 6.89 (2H, d, *J* = 8.3 Hz, 2H-4′), 6.76 (2H, d, *J* = 8.3 Hz, 2H-5′), 4.15 (2H, t, *J* = 7.3 Hz, H_2_-1′), 3.68 (3H, s, OMe), 2.75 (2H, t, *J* = 7.3 Hz, H_2_-2′); ^13^C NMR (100 MHz, DMSO-*d*_6_) δ 157.8 (C-6′), 137.8 (C-2), 136.8 (C-8a), 129.7 (C-3′), 129.4 (2C-4′), 127.4 (C-4), 125.5 (C-12a), 125.1 (C-7), 125.0 (C-5), 122.4 (C-11), 120.6 (C-12), 114.4 (C-9), 114.3 (C-10), 113.6 (2C-5′), 104.1 (C-6), 54.9 (OMe), 46.0 (C-1′), 35.3 (C-2′); (+)-HRESIMS *m/z* 396.0718 [M+H]^+^ (calcd for C_20_H_19_^79^BrN_3_O, 396.0706), *m/z* 398.0700 [M+H]^+^ (calcd for C_20_H_19_^81^BrN_3_O, 398.0688). 

#### 3.2.30. 3-(1-(2,5-Dimethoxyphenethyl)-1*H*-imidazol-5-yl)-1*H*-indole (**29**) 

Using the general procedure, reaction of 1*H*-indole-3-carbaldehyde (0.145 g, 1.0 mmol) with 2,5-dimethoxyphenethylamine (0.17 mL, 1.0 mmol), K_2_CO_3_ (0.138 g, 1.0 mmol) and *p*-toluenesulfonylmethyl isocyanide (0.195 g, 1.0 mmol) in DMF (1 mL) followed by purification using silica gel column chromatography (1–10% CH_2_Cl_2_/MeOH, 99:1→9:1) afforded the title compound as a brown oil (0.138 g, 40%). *R_f_* = 0.38 (CH_2_Cl_2_/MeOH, 9:1); IR (ATR) ν_max_ 3401, 2916, 1644, 1592, 1498, 1223, 823, 796 cm^−1^; ^1^H NMR (400 MHz, DMSO-*d*_6_) δ 11.49 (1H, br s, NH-8), 7.65 (1H, s, H-2), 7.51 (1H, d, *J* = 7.8 Hz, H-12), 7.51 (1H, s, H-7), 7.48 (1H, d, *J* = 7.8 Hz, H-9), 7.17 (1H, ddd, *J* = 14.9, 7.8, 1.1 Hz, H-10), 7.07 (1H, ddd, *J* = 14.9, 7.8, 1.1 Hz, H-11), 7.01 (1H, d, *J* = 0.9 Hz, H-4), 6.79 (1H, d, *J* = 8.7 Hz, H-5′), 6.70 (1H, dd, *J* = 8.7, 2.9 Hz, H-6′), 6.53 (1H, d, *J* = 2.9 Hz, H-8′), 4.16 (2H, t, *J* = 7.4 Hz, H_2_-1′), 3.62 (3H, s, 7′-OMe), 3.58 (3H, s, 4′-OMe), 2.82 (2H, t, *J* = 7.4 Hz, H_2_-2′); ^13^C NMR (100 MHz, DMSO-*d*_6_) δ 153.1 (C-7′), 151.3 (C-4′), 137.6 (C-2), 136.1 (C-8a), 127.0 (C-4), 126.7 (C-3′), 126.5 (C-12a), 125.9 (C-5), 124.1 (C-7), 121.6 (C-10), 119.5 (C-11), 119.0 (C-12), 116.3 (C-8′), 112.1 (C-6′), 111.7 (C-9), 111.5 (C-5′), 103.8 (C-6), 55.5 (4′-OMe), 55.2 (7′-OMe), 44.3 (C-1′), 31.9 (C-2′); (+)-HRESIMS *m/z* 348.1712 [M+H]^+^ (calcd for C_21_H_22_N_3_O_2_, 348.1707). 

#### 3.2.31. 3-(1-(2,5-Dimethoxyphenethyl)-1*H*-imidazol-5-yl)-5-fluoro-1*H*-indole (**30**) 

Using the general procedure, reaction of 5-fluoro-1*H*-indole-3-carbaldehyde (0.163 g, 1.0 mmol) with 2,5-dimethoxyphenethylamine (0.17 mL, 1.0 mmol), K_2_CO_3_ (0.138 g, 1.0 mmol) and *p*-toluenesulfonylmethyl isocyanide (0.195 g, 1.0 mmol) in DMF (1 mL) followed by purification using silica gel column chromatography (1–10% CH_2_Cl_2_/MeOH, 99:1→9:1) afforded the title compound as a brown solid (0.171 g, 47%). *R_f_* = 0.43 (CH_2_Cl_2_/MeOH, 9:1); m.p 150–151 °C; IR (ATR) ν_max_ 3027, 230, 1610, 1499, 1454, 1222, 1130, 1032, 821, 791 cm^−1^; ^1^H NMR (400 MHz, DMSO-*d*_6_) δ 11.56 (1H, br s, NH-8), 7.65 (1H, s, H-2), 7.56 (1H, d, *J* = 2.6 Hz, H-7), 7.46 (1H, dd, *J* = 8.9, 4.7 Hz, H-9), 7.16 (1H, dd, *J* = 9.8, 2.3 Hz, H-12), 7.01 (1H, ddd, *J* = 9.8, 8.9, 2.5 Hz, H-10), 6.98 (1H, s, H-4), 6.77 (1H, d, *J* = 8.8 Hz, H-5′), 6.68 (1H, dd, *J* = 8.8, 2.9 Hz, H-6′), 6.51 (1H, d, *J* = 2.9 Hz, H-8′), 4.14 (2H, t, *J* = 7.7 Hz, H_2_-1′), 3.62 (3H, s, 7′-OMe), 3.56 (3H, s, 4′-OMe), 2.80 (2H, t, *J* = 7.7 Hz, H_2_-2′); ^13^C NMR (100 MHz, DMSO-*d*_6_) δ 157.4 (d, ^1^*J*_FC_ = 236.3, C-11), 152.9 (C-7′), 151.2 (C-4′), 137.7 (C-2), 132.7 (C-8a), 127.1 (C-3′), 126.8 (d, ^3^*J*_FC_ = 10.0, C-12a), 126.6 (C-4), 126.1 (C-7), 125.4 (C-5), 116.3 (C-8′), 112.8 (d, ^3^*J*_FC_ = 9.8 Hz, C-9), 112.1 (C-6′), 111.5 (C-5′), 109.8 (d, ^2^*J*_FC_ = 26.4, C-10), 104.1 (d, ^4^*J*_FC_ = 4.7 Hz, C-6), 103.5 (d, ^2^*J*_FC_ = 24.7, C-12), 55.5 (4′-OMe), 55.2 (7′-OMe), 44.2 (C-1′), 32.0 (C-2′); (+)-HRESIMS *m/z* 388.1433 [M+Na]^+^ (calcd for C_21_H_20_FN_3_NaO_2_, 388.1432). 

#### 3.2.32. 3-(1-(2,5-Dimethoxyphenethyl)-1*H*-imidazol-5-yl)-6-fluoro-1*H*-indole (**31**) 

Using the general procedure, reaction of 6-fluoro-1*H*-indole-3-carbaldehyde (0.163 g, 1.0 mmol) with 2,5-dimethoxyphenethylamine (0.17 mL, 1.0 mmol), K_2_CO_3_ (0.138 g, 1.0 mmol) and *p*-toluenesulfonylmethyl isocyanide (0.195 g, 1.0 mmol) in DMF (1 mL) followed by purification using silica gel column chromatography (1%–10% CH_2_Cl_2_/MeOH, 99:1→9:1) afforded the title compound as an orange solid (0.156 g, 43%). *R_f_* = 0.60 (CH_2_Cl_2_/MeOH, 9:1); m.p 130–131 °C; IR (ATR) ν_max_ 2990, 2827, 1632, 1605, 1590, 1499, 1217, 1122, 834, 788 cm^−1^; ^1^H NMR (400 MHz, DMSO*-d*_6_) δ 11.52 (1H, br s, NH-8), 7.65 (1H, d, *J* = 1.1 Hz, H-2), 7.49 (1H, d, *J* = 2.6 Hz, H-7), 7.46 (1H, dd, *J* = 8.6, 5.5 Hz, H-12), 7.23 (1H, dd, *J* = 9.9, 2.3 Hz, H-9), 6.99 (1H, d, *J* = 1.1 Hz, H-4), 6.92 (1H, ddd, *J* = 11.9, 8.6, 2.3 Hz, H-11), 6.80 (1H, d, *J* = 9.0 Hz, H-5′), 6.70 (1H, dd, *J* = 9.0, 3.1 Hz, H-6′), 6.51 (1H, d, *J* = 3.1 Hz, H-8′), 4.14 (2H, t, *J* = 7.3 Hz, H_2_-1′), 3.62 (3H, s, 7′-OMe), 3.58 (3H, s, 4′-OMe), 2.80 (2H, t, *J* = 7.3 Hz, H_2_-2′); ^13^C NMR (100 MHz, DMSO-*d*_6_) δ 159.0 (d, ^1^*J*_FC_ = 242.0, C-10), 152.8 (C-7′), 151.2 (C-4′), 137.7 (C- 2), 135.8 (d, ^3^*J*_FC_ = 12.7, C-8a), 127.1 (C-4), 126.8 (C-3′), 124.5 (C-5), 123.3 (C-12a), 120.0 (d, ^3^*J*_FC_ = 11.4 Hz, C-12), 116.2 (C-8′), 112.1 (C-6′), 108.0 (d, ^2^*J*_FC_ = 23.9 Hz, C-11), 104.0 (C-6), 97.6 (d, ^2^*J*_FC_ = 26.0, C-9), 55.5 (4′-OMe), 55.2 (7′-OMe), 44.2 (C-1′), 31.9 (C-2′); (+)-HRESIMS *m/z* 388.1432 [M+Na]^+^ (calcd for C_21_H_20_FN_3_NaO_2_, 388.1418). 

#### 3.2.33. 5-Chloro-3-(1-(2,5-dimethoxyphenethyl)-1*H*-imidazol-5-yl)-1*H*-indole (**32**) 

Using the general procedure, reaction of 5-chloro-1*H*-indole-3-carbaldehyde (0.179 g, 1.0 mmol) with 2,5-dimethoxyphenethylamine (0.17 mL, 1.0 mmol), K_2_CO_3_ (0.138 g, 1.0 mmol) and *p*-toluenesulfonylmethyl isocyanide (0.195 g, 1.0 mmol) in DMF (1 mL) followed by purification using silica gel column chromatography (1–10% CH_2_Cl_2_/MeOH, 99:1→9:1) afforded the title compound as a brown oil (0.198 g, 52%). *R_f_* = 0.49 (CH_2_Cl_2_/MeOH, 9:1); IR (ATR) ν_max_ 3116, 2833, 1592, 1500, 1457, 1222, 110, 1046, 89, 796 cm^−1^; ^1^H NMR (400 MHz, DMSO-*d*_6_) δ 11.66 (1H, br s, NH-8), 7.66 (1H, d, *J* = 0.8 Hz, H-2), 7.55 (1H, d, *J* = 1.5 Hz, H-7), 7.48 (1H, d, *J* = 8.6 Hz, H-9), 7.40 (1H, d, *J* = 2.0 Hz, H-12), 7.16 (1H, dd, *J* = 8.6, 2.0 Hz, H-10), 6.98 (1H, s, H-4), 6.75 (1H, d, *J* = 8.7 Hz, H-5′), 6.68 (1H, dd, *J* = 8.7, 2.9 Hz, H-6′), 6.49 (1H, d, *J* = 2.9 Hz, H-8′), 4.14 (2H, t, *J* = 7.6 Hz, H_2_-1′), 3.62 (3H, s, 7′-OMe), 3.55 (3H, s, 4′-OMe), 2.78 (2H, t, *J* = 7.6 Hz, H_2_-2′); ^13^C NMR (100 MHz, DMSO-*d*_6_), δ 152.8 (C-7′), 151.2 (C-4′), 137.8 (C-2), 134.5 (C-8a), 127.7 (C-12a), 127.3 (C-7), 126.6 (C-3′), 125.9 (C-4), 125.1 (C-5), 124.2 (C-11), 121.6 (C-10), 117.9 (C-12), 116.2 (C-8′), 113.3 (C-9), 112.1 (C-6′), 111.4 (C-5′), 103.6 (C-6), 55.4 (4′-OMe), 55.2 (7′-OMe), 44.2 (C-1′), 32.0 (C-2′); (+)-HRESIMS *m/z* 404.1129 [M+Na]^+^ (calcd for C_21_H_20_^35^ClN_3_NaO_2_, 404.1136), *m/*z 406.1107 [M+Na]^+^ (calcd for C_21_H_20_^37^ClN_3_NaO_2_, 406.1115). 

#### 3.2.34. 6-Chloro-3-(1-(2,5-dimethoxyphenethyl)-1*H*-imidazol-5-yl)-1*H*-indole (**33**) 

Using the general procedure, reaction of 6-chloro-1*H*-indole-3-carbaldehyde (0.179 g, 1.0 mmol) with 2,5-dimethoxyphenethylamine (0.17 mL, 1.0 mmol), K_2_CO_3_ (0.138 g, 1.0 mmol) and *p*-toluenesulfonylmethyl isocyanide (0.195 g, 1.0 mmol) in DMF (1 mL) followed by purification by trituration from CH_2_Cl_2_ afforded the title compound as a pale yellow solid (0.044 g, 12%). *R_f_* = 0.57 (CH_2_Cl_2_/MeOH, 9:1); m.p 80–81 °C; IR (ATR) ν_max_ 3534, 3157, 2966, 1501, 1457, 1220, 1024, 872, 789, 715 cm^−1^; ^1^H NMR (400 MHz, DMSO*-d*_6_) δ 11.58 (1H, br s, NH-8), 7.66 (1H, d, *J* = 0.6 Hz, H-2), 7.53 (1H, d, *J* = 2.5 Hz, H-7), 7.50 (1H, d, *J* = 2.0 Hz, H-9), 7.47 (1H, d, *J* = 8.5 Hz, H-12), 7.07 (1H, dd, *J* = 8.5, 2.0 Hz, H-10), 6.99 (1H, d, *J* = 0.6 Hz, H-4), 6.79 (1H, d, *J* = 9.0 Hz, H-5′), 6.69 (1H, dd, *J* = 9.0, 3.0 Hz, H-6′), 6.50 (1H, d, *J* = 3.0 Hz, H-8′), 4.14 (2H, t, *J* = 7.5 Hz, H_2_-1′), 3.62 (3H, s, 7′-OMe), 3.57 (3H, s, 4′-OMe), 2.79 (2H, t, *J* = 7.5 Hz, H_2_-2′); ^13^C NMR (100 MHz, DMSO-*d*_6_) δ 152.9 (C-7′), 151.2 (C-4′), 137.8 (C-2), 136.4 (C-8a), 127.2 (C-4), 126.6 (C-3′), 126.3 (C-10), 125.3 (C-12a), 125.2 (C-5), 125.1 (C-7), 120.3 (C-12), 119.8 (C-11), 116.3 (C-8′), 112.1 (C-6′), 111.5 (C-5′), 111.3 (C-9), 104.1 (C-6), 55.5 (4′-OMe), 55.2 (7′-OMe), 44.3 (C-1′), 31.9 (C-2′); (+)-HRESIMS *m/z* 382.1305 [M+H]^+^ (calcd for C_21_H_21_^35^ClN_3_O_2_, 382.1317), *m/*z 384.1280 [M+H]^+^ (calcd for C_21_H_21_^37^ClN_3_O_2_, 384.1295). 

#### 3.2.35. 5-Bromo-3(1-(2,5-dimethoxyphenethyl)-1*H*-imidazol-5-yl)-1*H*-indole (**34**) 

Using the general procedure, reaction of 5-bromo-1*H*-indole-3-carbaldehyde (0.224 g, 1.0 mmol) with 2,5-dimethoxyphenethylamine (0.17 mL, 1.0 mmol), K_2_CO_3_ (0.138 g, 1.0 mmol) and *p*-toluenesulfonylmethyl isocyanide (0.195 g, 1.0 mmol) in DMF (1 mL) followed by purification by trituration from CH_2_Cl_2_ afforded the title compound as a light brown powder (0.010 g, 3%). *R_f_* = 0.45 (CH_2_Cl_2_/MeOH, 9:1); m.p 141–142 °C; IR (ATR) ν_max_ 3124, 2836, 2161, 1619, 1593, 1495, 1455, 1226, 1121, 1030, 918, 884, 782 cm^−1^; ^1^H NMR (400 MHz, DMSO*-d*_6_) δ 11.66 (1H, br s, NH-8), 7.65 (1H, d, *J* = 0.9 Hz, H-2), 7.54 (1H, s, H-12), 7.53 (1H, s, H-7), 7.43 (1H, d, *J* = 8.6 Hz, H-9), 7.27 (1H, dd, *J* = 8.6, 1.9 Hz, H-10), 6.98 (1H, d, *J* = 0.9 Hz, H-4), 6.75 (1H, d, *J* = 8.6 Hz, H-5′), 6.68 (1H, dd, *J* = 8.6, 2.9 Hz, H-6′), 6.48 (1H, d, *J* = 2.9 Hz, H-8′), 4.14 (2H, t, *J* = 7.7 Hz, H_2_-1′), 3.62 (3H, s, 4′-OMe), 3.54 (3H, s, 7′-OMe), 2.77 (2H, t, *J* = 7.7 Hz, H_2_-2′); ^13^C NMR (100 MHz, DMSO-*d*_6_) δ 152.8 (C-7′), 151.1 (C-4′), 137.8 (C-2), 134.7 (C-8a), 128.4 (C-12a), 127.3 (C-4), 125.7 (C-7), 125.0 (C-5), 124.1 (C-10), 120.9 (C-12), 116.2 (C-8′), 113.7 (C-9), 112.1 (C-11), 112.0 (C-6′), 111.4 (C-5′), 103.5 (C-6), 55.4 (7′-OMe), 55.2 (4′-OMe), 44.1 (C-1′), 32.0 (C-2′); (+)-HRESIMS *m/z* 426.0820 [M+H]^+^ (calcd for C_21_H_21_^79^BrN_3_O_2_, 426.0812), *m/z* 428.0801 [M+H]^+^ (calcd for C_21_H_21_^81^BrN_3_O_2_, 428.0794). 

#### 3.2.36. 6-Bromo-3-(1-(2,5-dimethoxyphenethyl)-1*H*-imidazol-5-yl)-1*H*-indole (**35**) 

Using the general procedure, reaction of 6-bromo-1*H*-indole-3-carbaldehyde (0.224 g, 1.0 mmol) with 2,5-dimethoxyphenethylamine (0.17 mL, 1.0 mmol), K_2_CO_3_ (0.138 g, 1.0 mmol) and *p*-toluenesulfonylmethyl isocyanide (0.195 g, 1.0 mmol) in DMF (1 mL) followed by purification by trituration from CH_2_Cl_2_ afforded the title compound as a pale white powder (0.088 g, 21%). *R_f_* = 0.71 (CH_2_Cl_2_/MeOH, 9:1); m.p 133–134 °C; IR (ATR) ν_max_ 2100, 1590, 1497, 1457, 1269, 1219, 795 cm^−1^; ^1^H NMR (400 MHz, DMSO*-d*_6_) δ 11.59 (1H, br s, NH-8), 7.65 (1H, d, *J* = 1.0 Hz, H-2), 7.64 (1H, d, *J* = 1.7 Hz, H-9), 7.51 (1H, d, *J* = 2.1 Hz, H-6), 7.42 (1H, d, *J* = 8.5 Hz, H-12), 7.19 (1H, dd, *J* = 8.5, 1.7 Hz, H-11), 6.98 (1H, d, *J* = 1.0 Hz, H-4), 6.79 (1H, d, *J* = 8.8 Hz, H-5′), 6.69 (1H, dd, *J* = 8.8, 3.0 Hz, H-6′), 6.50 (1H, d, *J* = 3.0 Hz, H-8′), 4.14 (2H, t, *J* = 7.7 Hz, H_2_-1′), 3.62 (3H, s, 7′-OMe), 3.57 (3H, s, 4′-OMe), 2.78 (2H, t, *J* = 7.7 Hz, H_2_-2′); ^13^C NMR (100 MHz, DMSO-*d*_6_) δ 152.8 (C-7′), 151.2 (C-4′), 137.8 (C-2), 136.8 (C-8a), 127.2 (C-4), 126.6 (C-5), 125.5 (C-12a), 125.1 (C-3′), 125.0 (C-7), 122.3 (C-11), 120.6 (C-12), 116.2 (C-8′), 114.3 (C-10), 114.2 (C-9), 112.1 (C-6′), 111.5 (C-5′), 104.1 (C-6), 55.5 (4′-OMe), 55.2 (7′-OMe), 44.2 (C-1′), 31.9 (C-2′); (+)-HRESIMS *m/z* 448.0622 [M+Na]^+^ (calcd for C_21_H_20_^79^BrN_3_NaO_2_, 448.0631), *m/z* 450.0613 [M+Na]^+^ (calcd for C_21_H_20_^81^BrN_3_NaO_2_, 450.0613). 

#### 3.2.37. 3-(1-(2-(Benzo[*d*][1,3]dioxol-5-yl)ethyl)-1*H*-imidazol-5-yl)-1*H*-indole (**36**) 

Using the general procedure, reaction of 1*H*-indole-3-carbaldehyde (0.145 g, 1.0 mmol) with homopiperonylamine (0.13 mL, 1.0 mmol), K_2_CO_3_ (0.138 g, 1.0 mmol) and *p*-toluenesulfonylmethyl isocyanide (0.195 g, 1.0 mmol) in DMF (1 mL) followed by purification using silica gel column chromatography (1–10% CH_2_Cl_2_/MeOH, 99:1→9:1) afforded the title compound as white crystals (0.075 g, 46%). *R_f_* = 0.51 (CH_2_Cl_2_/MeOH, 9:1); m.p 195–196 °C; IR (ATR) ν_max_ 2862, 1626, 1500, 1487, 1246, 1117, 819, 799, 737 cm^−1^; ^1^H NMR (400 MHz, DMSO-*d*_6_) δ 11.41 (1H, br s, NH-8), 7.59 (1H, d, *J* = 1.0 Hz, H-2), 7.49 (1H, s, H-7), 7.47 (1H, d, *J* = 7.4 Hz, H-12), 7.45 (1H, d, *J* = 7.4 Hz, H-9), 7.17 (1H, ddd, *J* = 7.4, 7.4, 1.0 Hz, H-10), 7.06 (1H, ddd, *J* = 7.4, 7.4, 1.0 Hz, H-11), 6.97 (1H, d, *J* = 1.0 Hz, H- 4), 6.73 (1H, d, *J* = 8.1 Hz, H-8′), 6.55 (1H, d, *J* =1.7 Hz, H-4′), 6.43 (1H, dd, *J* = 8.1, 1.7 Hz, H-9′), 5.93 (2H, s, H_2_-6′), 4.16 (2H, t, *J* = 7.6 Hz, H_2_-1′), 2.74 (2H, t, *J* = 7.6 Hz, H_2_-2′); ^13^C NMR (100 MHz, DMSO-*d*_6_) δ 147.1 (C-4a′), 145.5 (C-7a′), 137.6 (C-2), 136.0 (C-8a), 131.7 (C-3′), 127.3 (C-4), 126.5 (C-12a), 125.7 (C-5), 124.2 (C-7), 121.7 (C-10), 121.5 (C-9′), 119.5 (C-11), 118.8 (C-12), 111.8 (C-9), 108.8 (C-4′), 108.0 (C-8′), 103.8 (C-6), 100.7 (C-6′), 46.0 (C-1′), 35.9 (C-2′); (+)-HRESIMS *m/z* 332.1398 [M+H]^+^ (calcd for C_20_H_18_N_3_O_2_, 332.1394) 

#### 3.2.38. 3-(1-(2-(Benzo[*d*][1,3]dioxol-5-yl)ethyl)-1*H*-imidazol-5-yl)-5-fluoro-1*H*-indole (**37**) 

Using the general procedure, reaction of 5-fluoro-1*H*-indole-3-carbaldehyde (0.163 g, 1.0 mmol) with homopiperonylamine (0.13 mL, 1.0 mmol), K_2_CO_3_ (0.138 g, 1.0 mmol) and *p*-toluenesulfonylmethyl isocyanide (0.195 g, 1.0 mmol) in DMF (1 mL) followed by purification using silica gel column chromatography (1%–10% CH_2_Cl_2_/MeOH, 99:1→9:1) afforded the title compound as a pale yellow powder (0.137 g, 39%). *R_f_* = 0.49 (CH_2_Cl_2_/MeOH, 9:1); m.p > 200 °C; IR (ATR) ν_max_ 3137, 3105, 2834, 2784, 1583, 1502, 1238, 1115, 1033, 940, 807, 789 cm^−1^; ^1^H NMR (400 MHz, DMSO-*d*_6_) δ 11.53 (1H, br s, NH-8), 7.62 (1H, s, H-2), 7.56 (1H, d, *J* = 2.6 Hz, H-7), 7.45 (1H, dd, *J* = 8.9, 4.6 Hz, H-9), 7.15 (1H, dd, *J* = 9.8, 2.5 Hz, H-12), 7.00 (1H, ddd, *J* = 9.3, 8.9, 2.5 Hz, H-10), 6.97 (1H, s, H-4), 6.72 (1H, s, *J* = 8.1 Hz, H-8′), 6.54 (1H, d, *J* = 1.3 Hz, H-4′), 6.42 (1H, dd, *J* = 8.1, 1.3 Hz, H-9′), 5.92 (2H, s, H_2_-6′), 4.16 (2H, t, *J* = 7.3 Hz, H_2_-1′), 2.75 (2H, t, *J* = 7.3 Hz, H_2_-2′); ^13^C NMR (100 MHz, DMSO-*d*_6_) δ 157.4 (d, ^1^*J*_CF_ = 230.6 Hz, C-11), 147.1 (C-4a′), 145.7 (C-7a′), 137.7 (C-2), 132.6 (C-8a), 131.7 (C-3′), 127.4 (C-7), 126.8 (d, ^3^*J*_CF_ = 10.2 Hz, C-12a), 126.2 (C-4), 125.3 (C-5), 121.5 (C-9′), 112.9 (d, ^3^*J*_CF_ = 9.5 Hz, C-9), 109.9 (d, ^2^*J*_CF_ = 26.0 Hz, C-10), 108.8 (C-4′), 108.0 (C-8′), 104.1 (d, ^4^*J*_CF_ = 4.8 Hz, C-6), 103.4 (d, ^2^*J*_CF_ = 23.5 Hz, H-12), 100.7 (C-6′), 45.9 (C-1′), 35.9 (C-2′); (+)-HRESIMS *m/z* 350.1307 [M+H]^+^ (calcd for C_20_H_17_FN_3_O_2_, 350.1299). 

#### 3.2.39. 3-(1-(2-(Benzo[*d*][1,3]dioxol-5-yl)ethyl)-1*H*-imidazol-5-yl)-6-fluoro-1*H*-indole (**38**) 

Using the general procedure, reaction of 6-fluoro-1*H*-indole-3-carbaldehyde (0.163 g, 1.0 mmol) with homopiperonylamine (0.13 mL, 1.0 mmol), K_2_CO_3_ (0.138 g, 1.0 mmol) and *p*-toluenesulfonylmethyl isocyanide (0.195 g, 1.0 mmol) in DMF (1 mL) followed by purification by trituration from CH_2_Cl_2_ afforded the title compound as an pale white powder (0.089 g, 26%). *R_f_* = 0.69 (CH_2_Cl_2_/MeOH, 9:1); m.p 188–189 °C; IR (ATR) ν_max_ 2785, 1605, 1490, 1445, 1502, 1345, 1248,806, 796 cm^−1^; ^1^H NMR (400 MHz, DMSO*-d*_6_) δ 11.48 (1H, br s, NH-8), 7.61 (1H, s, H-2), 7.49 (1H, d, *J* = 2.5 Hz, H-7), 7.45 (1H, dd, *J* = 8.8, 5.5 Hz, H-12), 7.23 (1H, dd, *J* = 10.0, 2.5 Hz, H-9), 6.98 (1H, d, *J* = 1.1 Hz, H-4), 6.92 (1H, ddd, *J* = 12.2, 8.5, 2.2 Hz, H-11), 6.72 (1H, d, *J* = 7.9 Hz, H-8′), 6.55 (1H, d, *J* = 1.6 Hz, H-4′), 6.42 (1H, dd, *J* = 7.9, 1.6 Hz, H-9′), 5.93 (2H, s, H_2_-6′), 4.16 (2H, t, *J* = 7.2 Hz, H_2_-1′), 2.75 (2H, t, *J* = 7.2 Hz, H_2_-2′); ^13^C NMR (100 MHz, DMSO-*d*_6_) δ 159.0 (d, ^1^*J*_CF_ = 235.3 Hz, C-10), 147.1 (C-4a′), 145.7 (C-7a′), 137.7 (C-2), 135.8 (C-8a), 131.6 (C-3′), 127.3 (C-4), 125.3 (C-5), 124.7 (C-7), 123.3 (C-12a), 121.5 (C-9′), 119.9 (d, ^3^*J*_CF_ = 10.3 Hz, C-12), 108.8 (C-4′), 108.0 (d, ^2^*J*_CF_ = 25.2, C-11, C-8′), 104.0 (C-6), 100.6 (C-6′), 97.7 (d, ^2^*J*_CF_ = 25.2, C-9), 46.0 (C-1′), 35.9 (C-2′); (+)-HRESIMS *m/z* 350.1299 [M+H]^+^ (calcd for C_20_H_17_FN_3_O_2_, 350.1287). 

#### 3.2.40. 3-(1-(2-(Benzo[*d*][1,3]dioxol-5-yl)ethyl)-1*H*-imidazol-5-yl)-5-chloro-1*H*-indole (**39**) 

Using the general procedure, reaction of 5-chloro-1*H*-indole-3-carbaldehyde (0.179 g, 1.0 mmol) with homopiperonylamine (0.13 mL, 1.0 mmol), K_2_CO_3_ (0.138 g, 1.0 mmol) and *p*-toluenesulfonylmethyl isocyanide (0.195 g, 1.0 mmol) in DMF (1 mL) followed by purification by trituration from EtOAc afforded the title compound as a white powder (0.146 g, 40%). *R_f_* = 0.62 (CH_2_Cl_2_/MeOH, 9:1); m.p > 200 °C; IR (ATR) ν_max_ 3137, 2784, 1632, 1584, 1509, 1463, 1439, 1239, 1123, 1114, 940, 794 cm^−1^; ^1^H NMR (400 MHz, DMSO*-d*_6_) δ 11.62 (1H, br s, NH-8), 7.63 (1H, s, H-2), 7.57 (1H, d, *J* = 2.5 Hz, H-7), 7.47 (1H, d, *J* = 8.5 Hz, H-9), 7.41 (1H, d, *J* = 2.0 Hz, H-12), 7.16 (1H, dd, *J* = 8.5, 2.0 Hz, H-10), 6.98 (1H, s, H-4), 6.71 (1H, d, *J* = 8.0 Hz, H-8′), 6.53 (1H, d, *J* = 1.5 Hz, H-4′), 6.41 (1H, dd, *J* = 8.0, 1.5 Hz, H-9′), 5.92 (2H, s, H_2_-6′), 4.16 (2H, t, *J* = 7.2 Hz, H_2_-1′), 2.74 (2H, t, *J* = 7.2 Hz, H_2_-2′), ^13^C NMR (100 MHz, DMSO-*d*_6_) δ 147.1 (C-4a′), 145.7 (C-7a′), 137.8 (C-2), 134.4 (C-8a), 131.6 (C-3′), 127.7 (C-12a), 127.5 (C-4), 126.0 (C-7), 124.9 (C-5), 124.2 (C-11), 121.7 (C-9′), 121.4 (C-10), 117.8 (C-12), 113.4 (C-9), 108.9 (C-4′), 108.0 (C-8′), 103.6 (C-6), 100.6 (C-6′), 45.9 (C-1′), 35.9 (C-2′); (+)-HRESIMS *m/z* 388.0812 [M+Na]^+^ (calcd for C_20_H_16_^35^ClN_3_NaO_2_, 388.0823), *m/z* 390.0794 [M+Na]^+^ (calcd for C_20_H_16_^37^ClN_3_NaO_2_, 390.0801). 

#### 3.2.41. 3-(1-(2-(Benzo[*d*][1,3]dioxol-5-yl)ethyl)-1*H*-imidazol-5-yl)-6-chloro-1*H*-indole (**40**) 

Using the general procedure, reaction of 6-chloro-1*H*-indole-3-carbaldehyde (0.179 g, 1.0 mmol) with homopiperonylamine (0.13 mL, 1.0 mmol), K_2_CO_3_ (0.138 g, 1.0 mmol) and *p*-toluenesulfonylmethyl isocyanide (0.195 g, 1.0 mmol) in DMF (1 mL) followed by purification by trituration from CH_2_Cl_2_ afforded the title compound as a light brown solid (0.106 g, 29%). *R_f_* = 0.49 (CH_2_Cl_2_/MeOH, 9:1); m.p 180–181 °C; IR (ATR) ν_max_ 3014, 2900, 1737, 1504, 1487, 1251, 1042, 910, 809 cm^−1^; ^1^H NMR (400 MHz, DMSO*-d*_6_) δ 11.55 (1H, br s, NH-8), 7.61 (1H, d, *J* = 0.8 Hz, H-2), 7.54 (1H, d, *J* = 2.6 Hz, H-7), 7.49 (1H, d, *J* = 2.0 Hz, H-9), 7.46 (1H, d, *J* = 8.5 Hz, H-12), 7.07 (1H, dd, *J* = 8.5, 2.0 Hz, H-11), 6.98 (1H, s, H-4), 6.72 (1H, d, *J* = 7.8 Hz, H-8′), 6.54 (1H, d, *J* = 2.0 Hz, H-4′), 6.41 (1H, dd, *J* = 7.8, 2.0 Hz, H-9′), 5.92 (2H, s, H_2_-6′), 4.16 (2H, t, *J* = 7.1 Hz, H_2_-1′), 2.74 (2H, t, *J* = 7.1 Hz, H_2_-2′); ^13^C NMR (100 MHz, DMSO-*d*_6_) δ 147.1 (C-4a′), 145.7 (C-7a′), 137.8 (C-2), 136.3 (C-8a), 131.6 (C-3′), 127.5 (C-4), 126.4 (C-10), 125.3 (C-12a), 125.2 (C-7), 125.1 (C-5), 121.5 (C-9′), 120.2 (C-12), 119.9 (C-11), 111.3 (C-9), 108.8 (C-4′), 108.0 (C-8′), 104.1 (C-6), 100.7 (C-6′), 46.0 (C-1′), 35.9 (C-2′); (+)-HRESIMS *m/z* 388.0836 [M+Na]^+^ (calcd for C_20_H_16_^35^ClN_3_NaO_2_, 388.0823), *m/z* 390.0820 [M+Na]^+^ (calcd for C_20_H_16_^37^ClN_3_NaO_2_, 390.0801). 

#### 3.2.42. 3-(1-(2-(Benzo[*d*][1,3]dioxol-5-yl)ethyl)-1*H*-imidazol-5-yl)-5-bromo-1*H*-indole (**41**) 

Using the general procedure, reaction of 5-bromo-1*H*-indole-3-carbaldehyde (0.224 g, 1.0 mmol) with homopiperonylamine (0.13 mL, 1.0 mmol), K_2_CO_3_ (0.138 g, 1.0 mmol) and *p*-toluenesulfonylmethyl isocyanide (0.195 g, 1.0 mmol) in DMF (1 mL) followed by purification using silica gel column chromatography (1–10% CH_2_Cl_2_/MeOH, 99:1→9:1) afforded the title compound as a brown solid (0.164 g, 40%). *R_f_* = 0.41 (CH_2_Cl_2_/MeOH, 9:1); m.p 176–177 °C; IR (ATR) ν_max_ 3150, 2854, 1499, 1456, 1241, 865, 795 cm^−1^; ^1^H NMR (400 MHz, DMSO-*d*_6_) δ 11.65 (1H, br s, NH-8), 7.63 (1H, d, *J* = 1.0 Hz, H-2), 7.55 (1H, d, *J* = 1.6 Hz, H-7), 7.55 (1H, d, *J* = 1.6 Hz, H-12), 7.43 (1H, d, *J* = 8.5 Hz, H-9), 7.27 (1H, dd, *J* = 8.5, 1.6 Hz, H-10), 6.98 (1H, d, *J* = 1.0 Hz, H-4), 6.70 (1H, d, *J* = 8.2 Hz, H-8′), 6.53 (1H, d, *J* = 1.6 Hz, H-4′), 6.40 (1H, dd, *J* = 8.2, 1.6 Hz, H-9′), 5.92 (2H, s, H_2_-6′), 4.15 (2H, t, *J* = 7.3 Hz, H_2_-1′), 2.74 (2H, t, *J* = 7.3 Hz, H_2_-2′); ^13^C NMR (100 MHz, DMSO-*d*_6_) δ 147.1 (C-4a′), 145.7 (C-7a′), 137.8 (C-2), 134.7 (C-8a), 131.6 (C-3′), 128.4 (C-12a), 127.5 (C-4), 125.8 (C-7), 124.9 (C-5), 124.2 (C-10), 121.4 (C-9′), 120.8 (C-12), 113.8 (C-9), 112.2 (C-11), 108.8 (C-4′), 108.1 (C-8′), 103.5 (C-6), 100.6 (C-6′), 45.9 (C-1′), 35.9 (C-2′); (+)-HRESIMS *m/z* 432.0327 [M+Na]^+^ (calcd for C_20_H_16_^79^BrN_3_NaO_2_, 432.0318), *m/z* 434.0304 [M+Na]^+^ (calcd for C_20_H_16_^81^BrN_3_NaO_2_, 434.0300). 

#### 3.2.43. 3-(1-(2-(Benzo[*d*][1,3]dioxol-5-yl)ethyl)-1*H*-imidazol-5-yl)-6-bromo-1*H*-indole (**42**) 

Using the general procedure, reaction of 6-bromo-1*H*-indole-3-carbaldehyde (0.224 g, 1.0 mmol) with homopiperonylamine (0.13 mL, 1.0 mmol), K_2_CO_3_ (0.138 g, 1.0 mmol) and *p*-toluenesulfonylmethyl isocyanide (0.195 g, 1.0 mmol) in DMF (1 mL) followed by purification using silica gel column chromatography (1–10% CH_2_Cl_2_/MeOH, 99:1→9:1) afforded the title compound as a light brown powder (0.230 g, 56%). *R_f_* = 0.62 (CH_2_Cl_2_/MeOH, 9:1); m.p 194–195 °C; IR (ATR) ν_max_ 2781, 1596, 1504, 1487, 1253, 1110, 1042, 845, 806, 815 cm^−1^; ^1^H NMR (400 MHz, DMSO*-d*_6_) δ 11.55 (1H, br s, NH-8), 7.63 (1H, d, *J* = 1.8 Hz, H-9), 7.61 (1H, d, *J* = 1.0 Hz, H-2), 7.52 (1H, d, *J* = 2.6 Hz, H-7), 7.41 (1H, d, *J* = 8.5 Hz, H-12), 7.18 (1H, dd, *J* = 8.5, 1.8 Hz, H-11), 6.97 (1H, d, *J* = 1.0 Hz, H-4), 6.72 (1H, d, *J* = 7.7 Hz, H-8′), 6.54 (1H, d, *J* = 1.5 Hz, H-4′), 6.41 (1H, dd, *J* = 7.7, 1.5 Hz, H-9′), 5.92 (2H, s, H_2_-6′), 4.16 (2H, t, *J* = 7.3 Hz, H_2_-1′), 2.73 (2H, t, *J* = 7.3 Hz, H_2_-2′); ^13^C NMR (100 MHz, DMSO-*d*_6_) δ 147.1 (C-4a′), 145.7 (C-7a′), 137.8 (C-2), 136.8 (C-8a), 131.6 (C-3′), 127.5 (C-4), 125.5 (C-5), 125.1 (C-7), 125.0 (C-12a), 122.4 (C-11), 121.5 (C-9′), 120.6 (C-12), 114.4 (C-10), 114.3 (C-9), 108.8 (C-4′), 108.0 (C-8′), 100.7 (C-6′), 46.0 (C-1′), 35.9 (C-2′); (+)-HRESIMS *m/z* 432.0311 [M+Na]^+^ (calcd for C_20_H_16_^79^BrN_3_NaO_2_, 432.0318), *m/z* 434.0287 [M+Na]^+^ (calcd for C_20_H_16_^81^BrN_3_NaO_2_, 434.0300). 

#### 3.2.44. 3-(1-Benzyl-1*H*-imidazol-5-yl)-4-fluoro-1*H*-indole (**43**) 

Using the general procedure, reaction of 4-fluoro-1*H*-indole-3-carbaldehyde (0.028 g, 0.172 mmol) with benzylamine (19 µL, 0.172 mmol), K_2_CO_3_ (0.024 g, 0.172 mmol) and *p*-toluenesulfonylmethyl isocyanide (0.034 g, 0.172 mmol) followed by purification using silica gel column chromatography (CH_2_Cl_2_:MeOH, 1:0→9:1) afforded the title compound as a brown solid (0.028 g, 56%). R*_f_ =* 0.50 (CH_2_Cl_2_:MeOH, 9:1); m.p. 177–178 °C; IR (ATR) ν_max_ 3115, 2932, 2858, 1497, 1227, 1109, 716 cm^−1^; ^1^H NMR (400 MHz, CDCl_3_) δ 9.08 (1H, br s, NH-8), 7.57 (1H, d, *J* = 1.0 Hz, H-2), 7.25–7.18 (4H, m, H-9, 2H-4′, H-5′), 7.15 (1H, ddd, *J* = 7.8, 7.8, 4.8 Hz, H-10), 7.14 (1H, br s, H-4), 7.04 (1H, d, *J* = 2.5 Hz, H-7), 7.00–6.96 (2H, m, 2H-3′), 6.82 (1H, ddd, *J* = 11.0, 7.5, 1.0 Hz, H-11), 5.10 (2H, s, H_2_-1′); ^13^C NMR (100 MHz, CDCl_3_) δ 156.6 (d, ^1^*J*_CF_ = 247.7 Hz, C-12), 139.0 (d, ^3^*J*_CF_ = 10.2 Hz, C-8a), 138.0 (C-2), 137.0 (C-2′), 129.2 (C-4), 128.8 (2C-4′), 127.9 (C-5′), 127.3 (2C-3′), 126.6 (C-5), 125.3 (C-7), 123.3 (d, ^3^*J*_CF_ = 8.0 Hz, C-10), 116.2 (d, ^2^*J*_CF_ = 18.9 Hz, C-12a), 107.8 (d, ^4^*J*_CF_ = 3.8 Hz, C-8a), 105.8 (d, ^2^*J*_CF_ = 19.1 Hz, C-11), 102.8 (C-6), 49.0 (C-1′); (–)-HRESIMS *m/z* 290.1095 [M−H]^–^ (calcd for C_18_H_13_FN_3_, 290.1099). 

#### 3.2.45. 3-(1-Benzyl-1*H*-imidazol-5-yl)-7-fluoro-1*H*-indole (**44**) 

Using the general procedure, reaction of 7-fluoro-1*H*-indole-3-carbaldehyde (0.028 g, 0.172 mmol) with benzylamine (19 µL, 0.172 mmol), K_2_CO_3_ (0.024 g, 0.172 mmol) and *p*-toluenesulfonylmethyl isocyanide (0.034 g, 0.172 mmol) followed by purification using silica gel column chromatography (CH_2_Cl_2_:MeOH, 1:0→9:1) afforded the title compound as a yellow solid (0.019 g, 38%). R*_f_*= 0.49 (CH_2_Cl_2_:MeOH, 9:1); m.p. 179–180 °C; IR (ATR) ν_max_ 3074, 2917, 2849, 1454, 1234, 1110, 733 cm^−1^; ^1^H NMR (500 MHz, CDCl_3_) δ 8.93 (1H, br s, NH-8), 7.65 (1H, br s, H-2), 7.35 (1H, d, *J* = 7.9 Hz, H-12), 7.31–7.25 (4H, m, H-4, 2H-4′, H-5′), 7.07 (1H, ddd, *J* = 7.9, 7.9, 4.9 Hz, H-11), 7.01–6.97 (2H, m, 2H-3′), 6.96 (1H, dd, *J* = 11.0, 7.9 Hz, H-10), 5.13 (2H, s, H_2_-1′); ^13^C NMR (125 MHz, CDCl_3_) δ 149.7 (d, ^1^*J*_CF_ = 244.2 Hz, C-9), 138.4 (C-2), 137.2 (C-2′), 131.0 (d, ^3^*J*_CF_ = 4.8 Hz, C-12a), 129.07 (C-4), 129.05 (2C-4′), 128.0 (C-5′), 126.7 (2C-3′), 126.0 (C-5), 124.6 (d, ^2^*J*_CF_ = 13.7 Hz, C-8a), 124.4 (C-7), 121.0 (d, ^3^*J*_CF_ = 5.9 Hz, C-11), 115.4 (d, ^4^*J*_CF_ = 3.7 Hz, C-12), 107.7 (d, ^2^*J*_CF_ = 15.8 Hz, C-10), 106.1 (d, ^4^*J*_CF_ = 2.5 Hz, C-6), 48.8 (C-1′); (–)-HRESIMS *m/z* 290.1099 [M−H]^−^ (calcd for C_18_H_13_FN_3_, 290.1099). 

#### 3.2.46. 3-(1-Benzyl-1*H*-imidazol-5-yl)-4-chloro-1*H*-indole (**45**) 

Using the general procedure, reaction of 4-chloro-1*H*-indole-3-carbaldehyde (0.029 g, 0.162 mmol) with benzylamine (18 µL, 0.162 mmol), K_2_CO_3_ (0.023 g, 0.162 mmol) and *p*-toluenesulfonylmethyl isocyanide (0.032 g, 0.162 mmol) followed by purification using silica gel column chromatography (CH_2_Cl_2_:MeOH, 1:0→9:1) afforded the title compound as a light brown solid (0.020 g, 40%). R*_f_*(CH_2_Cl_2_:MeOH, 9:1) 0.40; m.p. 198–199 °C; IR (ATR) ν_max_ 3110, 2924, 2856, 1486, 1108, 777 cm^−1^; ^1^H NMR (400 MHz, CDCl_3_) δ 9.70 (1H, br s, NH-8), 7.60 (1H, d, *J* = 1.0 Hz, H-2), 7.36–7.31 (1H, m, H-9), 7.24–7.18 (3H, m, 2H-4′, H-5′), 7.15–7.10 (2H, m, H-10, H-11), 7.10 (1H, d, *J* = 1.0 Hz, H-4), 7.09 (1H, d, *J* = 2.6 Hz, H-2), 6.96–6.92 (2H, m, 2H-3′), 4.98 (2H, s, H_2_-1′); ^13^C NMR (100 MHz, CDCl_3_) δ 137.5 (C-8a), 137.4 (C-2), 136.8 (C-2′), 129.8 (C-4), 128.7 (2C-4′), 127.9 (C-5′), 127.6 (2C-3′), 127.5 (C-7), 126.3 (C-5), 125.9 (C-12), 124.8 (C-12a), 123.2 (C-10), 121.3 (C-11), 110.7 (C-9), 103.7 (C-6), 49.3 (C-1′); (–)-HRESIMS *m/z* 306.0809 [M−H]^–^ (calcd for C_18_H_13_^35^ClN_3_, 306.0803), *m/z* 308.0782 [M−H]^−^ (calcd for C_18_H_13_^37^ClN_3_, 308.0779). 

#### 3.2.47. 3-(1-Benzyl-1*H*-imidazol-5-yl)-5-chloro-1*H*-indole (**46**) 

Using the general procedure, reaction of 5-chloro-1*H*-indole-3-carbaldehyde (0.029 g, 0.162 mmol) with benzylamine (18 µL, 0.162 mmol), K_2_CO_3_ (0.023 g, 0.162 mmol) and *p*-toluenesulfonylmethyl isocyanide (0.032 g, 0.162 mmol) followed by purification using silica gel column chromatography (EtOAc) afforded the title compound as a light brown solid (0.020 g, 40%). R*_f_* = 0.49 (CH_2_Cl_2_:MeOH, 9:1); m.p. 78–79 °C; IR (ATR) ν_max_ 3121, 2923, 2855, 1455, 1109, 800 cm^−1^; ^1^H NMR (400 MHz, CDCl_3_) δ 8.63 (1H, br s, NH-8), 7.65 (1H, d, *J* = 1.0 Hz, H-2), 7.50 (1H, d, *J* = 2.0 Hz, H-12), 7.32 (1H, d, *J* = 8.8 Hz, H-9), 7.31–7.26 (3H, m, 2H-4′, H-5′), 7.22 (1H, d, *J* = 1.0 Hz, H-4), 7.19 (1H, dd, *J* = 8.8, 2.0 Hz, H-10), 7.00–6.95 (3H, m, H-7, 2H-3′), 5.11 (2H, s, H_2_-1′); ^13^C NMR (100 MHz, CDCl_3_) δ 138.4 (C-2), 137.1 (C-2′), 134.4 (C-8a), 129.1 (C-4), 129.0 (2C-4′), 128.5 (C-12a), 128.0 (C-5′), 126.7 (2C-3′), 126.6 (C-11), 125.9 (C-5), 125.1 (C-7), 123.4 (C-10), 119.2 (C-12), 112.5 (C-9), 105.0 (C-6), 48.8 (C-1′); (+)-HRESIMS *m/z* 308.0951 [M+H]^+^ (calcd for C_18_H_15_^35^ClN_3_, 308.0949), *m/z* 310.0916 [M+H]^+^ (calcd for C_18_H_15_^37^ClN_3_, 310.0925).

#### 3.2.48. 3-(1-Benzyl-1*H*-imidazol-5-yl)-7-chloro-1*H*-indole (**47**) 

Using the general procedure, reaction of 7-chloro-1*H*-indole-3-carbaldehyde (0.029 g, 0.162 mmol) with benzylamine (18 µL, 0.162 mmol), K_2_CO_3_ (0.023 g, 0.162 mmol) and *p*-toluenesulfonylmethyl isocyanide (0.032 g, 0.162 mmol) followed by purification using silica gel column chromatography (EtOAc) afforded the title compound as a yellow solid (0.012 g, 24%). R*_f_ =* 0.51 (CH_2_Cl_2_:MeOH, 9:1); m.p. 179–180 °C; IR (ATR) ν_max_ 3088, 2919, 2853, 1436, 1111, 736 cm^−1^; ^1^H NMR (400 MHz, CDCl_3_) δ 8.92 (1H, br s, NH-8), 7.65 (1H, d, *J* = 1.0 Hz, H-2), 7.48 (1H, dd, *J* = 8.0, 1.0 Hz, H-12), 7.32–7.22 (5H, m, H-4, H-10, 2H-4′, H-5′), 7.09 (1H, dd, *J* = 8.0, 7.4 Hz, H-11), 7.02 (1H, d, *J* = 2.0 Hz, H-7), 7.01–6.97 (2H, m, 2H-3′), 5.12 (2H, s, H_2_-1′); ^13^C NMR (100 MHz, CDCl_3_) δ 138.4 (C-2), 137.2 (C-2′), 133.4 (C-8a), 129.1 (C-4), 129.0 (2C-4′), 128.8 (C-12a), 128.0 (C-5′), 126.7 (2C-3′), 126.0 (C-5), 124.3 (C-7), 122.3 (C-10), 121.4 (C-11), 118.4 (C-12), 117.0 (C-9), 106.3 (C-6), 48.8 (C-1′); (–)-HRESIMS *m/z* 306.0814 (calcd for C_18_H_13_^35^ClN_3_, 306.0803), *m/z* 308.0791 [M−H]^−^ (calcd for C_18_H_13_^37^ClN_3_, 308.0779).

#### 3.2.49. 3-(1-Benzyl-1*H*-imidazol-5-yl)-4-bromo-1*H*-indole (**48**) 

Using the general procedure, reaction of 4-bromo-1*H*-indole-3-carbaldehyde (0.032 g, 0.142 mmol) with benzylamine (16 µL, 0.142 mmol), K_2_CO_3_ (0.020 g, 0.142 mmol) and *p*-toluenesulfonylmethyl isocyanide (0.028 g, 0.142 mmol) followed by purification using silica gel column chromatography (CH_2_Cl_2_:MeOH, 1:0→9:1) afforded the title compound as a yellow solid (0.025 g, 50%). R*_f_ =* 0.41 (CH_2_Cl_2_:MeOH, 9:1); m.p. 201–202 °C; IR (ATR) ν_max_ 3109, 3033, 2926, 2856, 1484, 1108, 775 cm^−1^; ^1^H NMR (400 MHz, CDCl_3_) δ 9.85 (1H, br s, NH-8), 7.61 (1H, d, *J* = 0.9 Hz, H-2), 7.40 (1H, dd, *J* = 7.8, 0.9 Hz, H-9), 7.32 (1H, dd, *J* = 7.8, 0.9 Hz, H-11), 7.24–7.19 (3H, m, 2H-4′, H-5′), 7.11–7.09 (2H, m, H-4, H-7), 7.07 (1H, dd, *J* = 7.8, 7.8 Hz, H-10), 6.97–6.93 (2H, m, 2H-3′), 4.95 (2H, s, H_2_-1′); ^13^C NMR (100 MHz, CDCl_3_) δ 137.3 (C-2), 137.2 (C-8a), 136.7 (C-2′), 130.4 (C-4), 128.7 (2C-4′), 127.90 (C-7/C-5′), 127.86 (C-7/C-5′), 127.6 (2C-3′), 126.3 (C-12a), 125.7 (C-5), 124.7 (C-11), 123.5 (C-10), 113.8 (C-12), 111.3 (C-9), 104.5 (C-6), 49.4 (C-1′); (–)-HRESIMS *m/z* 350.0304 [M−H]^−^ (calcd for C_18_H_13_^79^BrN_3_, 350.0298), *m/z* 352.0289 [M−H]^–^ (calcd for C_18_H_13_^81^BrN_3_, 352.0279). 

#### 3.2.50. 3-(1-Benzyl-1*H*-imidazol-5-yl)-5-methoxy-1*H*-indole (**49**) 

Using the general procedure, reaction of 5-methoxy-1*H*-indole-3-carbaldehyde (0.029 g, 0.165 mmol) with benzylamine (18 µL, 0.165 mmol), K_2_CO_3_ (0.022 g, 0.165 mmol) and *p*-toluenesulfonylmethyl isocyanide (0.032 g, 0.165 mmol) followed by purification using silica gel column chromatography (CH_2_Cl_2_:MeOH, 1:0→9:1) afforded the title compound as a brown solid (0.008 g, 16%). R*_f_*(CH_2_Cl_2_:MeOH, 9:1) 0.50; m.p. 65–66 °C; IR (ATR) ν_max_ 3124, 2956, 2930, 2859, 1482, 1115, 765 cm^−1^; ^1^H NMR (400 MHz, CDCl_3_) δ 8.43 (1H, br s, NH-8), 7.65 (1H, br s, H-2), 7.32–7.22 (5H, m, H-4, H-9, 2H-4′, H-5′), 7.01 (2H, dd, *J* = 7.9, 1.5 Hz, 2H-3′), 6.96 (2H, d, *J* = 2.4 Hz, H-7, H-12), 6.89 (1H, dd, *J* = 8.8, 2,4 Hz, H-10), 5.12 (2H, s, H_2_-1′), 3.75 (3H, s, OMe); ^13^C NMR (100 MHz, CDCl_3_) δ 155.0 (C-11), 138.2 (C-2), 137.4 (C-2′), 131.0 (C-8a), 129.0 (2C-4′), 128.7 (C-4, C-12a), 127.9 (C-5′), 126.7 (C-5, 2C-3′), 124.5 (C-7), 113.6 (C-10), 112.3 (C-9), 105.0 (C-6), 100.9 (C-12), 55.9 (OMe), 48.7 (C-1′); (–)-HRESIMS *m/z* 302.1293 [M−H]^−^ (calcd for C_19_H_16_N_3_O, 302.1299). 

#### 3.2.51. 3-(1-Benzyl-1*H*-imidazol-5-yl)-6-methoxy-1*H*-indole (**50**) 

Using the general procedure, reaction of 6-methoxy-1*H*-indole-3-carbaldehyde (0.029 g, 0.165 mmol) with benzylamine (18 µL, 0.165 mmol), K_2_CO_3_ (0.022 g, 0.165 mmol) and *p*-toluenesulfonylmethyl isocyanide (0.032 g, 0.165 mmol) followed by purification using silica gel column chromatography (CH_2_Cl_2_:MeOH, 1:0→9:1) afforded the title compound as a brown solid (0.010 g, 20%). R*_f_ =* 0.49 (CH_2_Cl_2_:MeOH, 9:1); m.p. 66–67 °C; IR (ATR) ν_max_ 3122, 2960, 2930, 2861, 1454, 1111, 764 cm^−1^; ^1^H NMR (400 MHz, CDCl_3_) δ 8.40 (1H, br s, NH-8), 7.62 (1H, br s, H-2), 7.46 (1H, d, *J* = 8.8 Hz, H-12), 7.32–7.22 (4H, m, H-4, 2H-4′, H-5′), 7.00 (2H, dd, *J* = 8.3, 1.9 Hz, 2H-3′), 6.88 (1H, d, *J* = 2.4 Hz, H-9), 6.87 (1H, d, *J* = 2.5 Hz, H-7), 6.83 (1H, dd, *J* = 8.8, 2.4 Hz, H-11), 5.14 (2H, s, H_2_-1′), 3.85 (3H, s, OMe); ^13^C NMR (100 MHz, CDCl_3_) δ 157.1 (C-10), 138.1 (C-2), 137.3 (C-2′), 136.8 (C-8a), 129.0 (2C-4′), 128.5 (C-4), 127.9 (C-5′), 126.8 (C-5), 126.7 (2C-3′), 122.4 (C-7), 121.6 (C-12a), 120.3 (C-12), 110.7 (C-11), 105.2 (C-6), 94.8 (C-9), 55.8 (OMe), 48.8 (C-1′); (–)-HRESIMS *m/z* 302.1304 [M−H]^−^ (calcd for C_19_H_16_N_3_O, 302.1299). 

#### 3.2.52. 4-Fluoro-3-(1-phenethyl-1*H*-imidazol-5-yl)-1*H*-indole (**51**) 

Using the general procedure, reaction of 4-fluoro-1*H*-indole-3-carbaldehyde (0.027 g, 0.164 mmol) was reacted with phenethylamine (21 µL, 0.164 mmol), K_2_CO_3_ (0.023 g, 0.164 mmol) and *p*-toluenesulfonylmethyl isocyanide (0.032 g, 0.164 mmol) followed by purification using silica gel column chromatography (EtOAc) afforded the title compound as a brown solid (0.049 g, 98%). R*_f_ =* 0.47 (CH_2_Cl_2_:MeOH, 9:1); m.p. 181–182 °C; IR (ATR) ν_max_ 3115, 2930, 2855, 1495, 1226, 736 cm^−1^; ^1^H NMR (400 MHz, CDCl_3_) δ 9.43 (1H, br s, NH-8), 7.45 (1H, d, *J* = 1.0 Hz, H-2), 7.23 (1H, d, *J* = 8.0 Hz, H-9), 7.21–7.15 (3H, m, 2H-5′, H-6′), 7.15 (1H, ddd, *J* = 8.0, 8.0, 5.0 Hz, H-10), 7.06 (1H, d, *J* = 1.0 Hz, H-4), 7.03 (1H, s, H-7), 6.90–6.86 (2H, m, 2H-4′), 6.81 (1H, ddd, *J* = 11.0, 8.0, 0.9 Hz, H-11), 4.15 (2H, t, *J* = 7.2 Hz, H_2_-1′), 2.86 (2H, t, *J* = 7.2 Hz, H_2_-2′); ^13^C NMR (100 MHz, CDCl_3_) δ 156.6 (d, ^1^*J*_CF_ = 248.2 Hz, C-12), 139.1 (d, ^3^*J*_CF_ = 10.6 Hz, C-8a), 137.9 (C-3′), 137.6 (C-2), 129.0 (C-4), 128.8 (2C-4′), 128.7 (2C-5′), 126.8 (C-6′), 126.1 (C-5), 125.3 (C-7), 123.2 (d, ^3^*J*_CF_ = 8.4 Hz, C-10), 116.2 (d, ^2^*J*_CF_ = 19.8 Hz, C-12a), 108.0 (d, ^4^*J*_CF_ = 3.7 Hz, C-9), 105.7 (d, ^2^*J*_CF_ = 19.2 Hz, C-11), 102.6 (C-6), 46.8 (C-1′), 37.3 (C-2′); (–)-HRESIMS *m/z* 304.1262 [M−H]^−^ (calcd for C_19_H_15_FN_3_, 304.1255).

#### 3.2.53. 7-Fluoro-3-(1-phenethyl-1*H*-imidazol-5-yl)-1*H*-indole (**52**) 

Using the general procedure, reaction of 7-fluoro-1*H*-indole-3-carbaldehyde (0.027 g, 0.164 mmol) was reacted with phenethylamine (21 µL, 0.164 mmol), K_2_CO_3_ (0.023 g, 0.164 mmol) and *p*-toluenesulfonylmethyl isocyanide (0.032 g, 0.164 mmol) followed by purification using silica gel column chromatography (EtOAc) afforded the title compound as a light brown solid (0.031 g, 62%). R*_f_* = 0.47 (CH_2_Cl_2_:MeOH, 9:1); m.p. 45–46 °C; IR (ATR) ν_max_ 3122, 2930, 2859, 1454, 1233, 783 cm^−1^; ^1^H NMR (400 MHz, CDCl_3_) δ 9.57 (1H, br s, NH-8), 7.49 (1H, d, *J* = 1.0 Hz, H-2), 7.30 (1H, d, *J* = 8.0 Hz, H-12), 7.23–7.16 (3H, m, 2H-5′, H-6′), 7.15 (1H, d, *J* = 1.0 Hz, H-4), 7.08 (1H, s, H-7), 7.07 (1H, ddd, *J* = 8.0, 8.0, 4.6 Hz, H-11), 6.97 (1H, ddd, *J* = 11.0, 8.0, 1.0 Hz, H-10), 6.91–6.87 (2H, m, 2H-4′), 4.15 (2H, t, *J* = 7.0 Hz, H_2_-1′), 2.87 (2H, t, *J* = 7.0 Hz, H_2_-2′); ^13^C NMR (100 MHz, CDCl_3_) δ 149.8 (d, ^1^*J*_CF_ = 244.9 Hz, C-9), 137.8 (C-2), 137.6 (C-3′), 131.0 (d, ^3^*J*_CF_ = 5.3 Hz, C-12a), 129.0 (C-4), 128.8 (2C-5′), 128.7 (2C-4′), 127.0 (C-6′), 125.5 (C-5), 124.71 (d, ^2^*J*_CF_ = 14.0 Hz, C-8a), 124.70 (C-7), 120.8 (d, ^3^*J*_CF_ = 6.0 Hz, C-11), 115.4 (d, ^4^*J*_CF_ = 3.2 Hz, C-12), 107.6 (d, ^2^*J*_CF_ = 16.2 Hz, C-10), 106.0 (d, ^4^*J*_CF_ = 2.9 Hz, C-6), 46.9 (C-1′), 37.6 (C-2′); (–)-HRESIMS *m/z* 304.1259 [M−H]^−^ (calcd for C_19_H_15_FN_3_, 304.1255). 

#### 3.2.54. 4-Chloro-3-(1-phenethyl-1*H*-imidazol-5-yl)-1*H*-indole (**53**) 

Using the general procedure, reaction of 4-chloro-1*H*-indole-3-carbaldehyde (0.028 g, 0.155 mmol) was reacted with phenethylamine (20 µL, 0.155 mmol), K_2_CO_3_ (0.022 g, 0.155 mmol) and *p*-toluenesulfonylmethyl isocyanide (0.030 g, 0.155 mmol) followed by purification using silica gel column chromatography (EtOAc) afforded the title compound as a light brown solid (0.034 g, 68%). R*_f_* = 0.46 (CH_2_Cl_2_:MeOH, 9:1); m.p. 73–74 °C; IR (ATR) ν_max_ 3105, 2922, 2858, 1490, 1193, 741 cm^−1^; ^1^H NMR (400 MHz, CDCl_3_) δ 9.99 (1H, br s, NH-8), 7.49 (1H, d, *J* = 1.0 Hz, H-2), 7.36 (1H, dd, *J* = 7.5, 1.5 Hz, H-8), 7.22–7.16 (3H, m, 2H-5′, H-6′), 7.14 (1H, dd, *J* = 7.5, 7.4 Hz, H-10), 7.10 (1H, dd, *J* = 7.4, 1.5 Hz, H-11), 7.05 (1H, d, *J* = 1.0 Hz, H-4), 7.03 (1H, d, *J* = 2.1 Hz, H-7), 6.88–6.84 (2H, m, 2H-4′), 4.07 (2H, t, *J* = 7.2 Hz, H_2_-1′), 2.85 (2H, t, *J* = 7.2 Hz, H_2_-2′); ^13^C NMR (100 MHz, CDCl_3_) δ 138.0 (C-3′), 137.6 (C-8a), 137.1 (C-2), 129.7 (C-4), 128.8 (2C-5′), 128.7 (2C-4′), 127.3 (C-7), 126.8 (C-6′), 125.9 (C-5, C-12), 124.8 (C-12a), 123.1 (C-10), 121.2 (C-11), 110.7 (C-9), 103.8 (C-6), 46.9 (C-1′), 37.3 (C-2′); (–)-HRESIMS *m/z* 320.0954 [M−H]^−^ (calcd for C_19_H_15_^35^ClN_3_, 320.0960), *m/z* 322.0931 [M−H]^−^ (calcd for C_19_H_15_^37^ClN_3_, 322.0936). 

#### 3.2.55. 7-Chloro-3-(1-phenethyl-1*H*-imidazol-5-yl)-1*H*-indole (**54**) 

Using the general procedure, reaction of 7-chloro-1*H*-indole-3-carbaldehyde (0.028 g, 0.155 mmol) was reacted with phenethylamine (20 µL, 0.155 mmol), K_2_CO_3_ (0.022 g, 0.155 mmol) and *p*-toluenesulfonylmethyl isocyanide (0.030 g, 0.155 mmol) followed by purification using silica gel column chromatography (EtOAc) afforded the title compound as a light brown solid (0.023 g, 46%). R*_f_ =* 0.51 (CH_2_Cl_2_:MeOH, 9:1); m.p. 47–48 °C; IR (ATR) ν_max_ 3102, 2923, 2853, 1494, 1110, 738 cm^−1^; ^1^H NMR (400 MHz, CDCl_3_) δ 9.43 (1H, br s, NH-8), 7.48 (1H, d, *J* = 1.0 Hz, H-2), 7.43 (1H, dd, *J* = 8.0, 1.0 Hz, H-12), 7.26 (1H, d, *J* = 7.6 Hz, H-10), 7.23–7.16 (3H, m, 2H-5′, H-6′), 7.14 (1H, d, *J* = 1.0 Hz, H-4), 7.10 (1H, s, H-7), 7.10 (1H, dd, *J* = 8.0, 7.6 Hz, H-11), 6.90–6.86 (2H, m, 2H-4′), 4.14 (2H, t, *J* = 7.3 Hz, H_2_-1′), 2.86 (2H, t, *J* = 7.3 Hz, H_2_-2′); ^13^C NMR (100 MHz, CDCl_3_) δ 137.8 (C-2), 137.6 (C-3′), 133.5 (C-8a), 129.1 (C-4), 128.9 (C-12a), 128.8 (2C-5′), 128.7 (2C-4′), 127.0 (C-6′), 125.4 (C-5), 124.6 (C-7), 122.2 (C-10), 121.4 (C-11), 118.3 (C-12), 117.1 (C-9), 106.3 (C-6), 46.8 (C-1′), 37.6 (C-2′); (–)-HRESIMS *m/z* 320.0959 [M−H]^−^ (calcd for C_19_H_15_^35^ClN_3_, 320.0960), *m/z* 322.0936 [M−H]^−^ (calcd for C_19_H_15_^37^ClN_3_, 322.0936). 

#### 3.2.56. 4-Bromo-3-(1-phenethyl-1*H*-imidazol-5-yl)-1*H*-indole (**55**) 

Using the general procedure, reaction of 4-bromo-1*H*-indole-3-carbaldehyde (0.031 g, 0.136 mmol) with phenethylamine (17 µL, 0.136 mmol), K_2_CO_3_ (0.019 g, 0.136 mmol) and *p*-toluenesulfonylmethyl isocyanide (0.027 g, 0.136 mmol) followed by purification using silica gel column chromatography (EtOAc) afforded the title compound as a yellow solid (0.039 g, 78%). R*_f_ =* 0.46 (CH_2_Cl_2_:MeOH, 9:1); m.p. 204–205 °C; IR (ATR) ν_max_ 3109, 2919, 2848, 1490, 1110, 743 cm^−1^; ^1^H NMR (400 MHz, CDCl_3_) δ 9.29 (1H, br s, NH-8), 7.52 (1H, d, *J* = 1.0 Hz, H-2), 7.40 (1H, dd, *J* = 8.0, 1.0 Hz, H-9), 7.30 (1H, dd, *J* = 7.6, 1.0 Hz, H-11), 7.23–7.16 (3H, m, 2H-5′, H-6′), 7.08 (1H, dd, *J* = 8.0, 7.6 Hz, H-10), 7.03 (1H, d, *J* = 1.0 Hz, H-4), 7.00 (1H, d, *J* = 2.5 Hz, H-7), 6.88–6.84 (2H, m, 2H-4′), 4.02 (2H, t, *J* = 7.0 Hz, H_2_-1′), 2.87 (2H, t, *J* = 7.0 Hz, H_2_-2′); ^13^C NMR (100 MHz, CDCl_3_) δ 138.1 (C-3′), 137.1 (C-8a), 137.0 (C-2), 130.3 (C-4), 128.8 (2C-5′), 128.7 (2C-4′), 127.4 (C-7), 126.8 (C-6′), 126.2 (C-12a), 125.1 (C-5), 124.8 (C-11), 123.6 (C-10), 113.9 (C-12), 111.1 (C-9), 105.0 (C-6), 46.9 (C-1′), 37.4 (C-2′); (–)-HRESIMS *m/z* 364.0452 [M−H]^−^ (calcd for C_19_H_15_^79^BrN_3_, 364.0455), *m/z* 366.0436 [M−H]^−^ (calcd for C_19_H_15_^81^BrN_3_, 366.0436). 

#### 3.2.57. 5-Methoxy-3-(1-phenethyl-1*H*-imidazol-5-yl)-1*H*-indole (**56**) 

Using the general procedure, reaction of 5-methoxy-1*H*-indole-3-carbaldehyde (0.028 g, 0.157 mmol) with phenethylamine (20 µL, 0.157 mmol), K_2_CO_3_ (0.023 g, 0.157 mmol) and *p*-toluenesulfonylmethyl isocyanide (0.031 g, 0.157 mmol) followed by purification using silica gel column chromatography (EtOAc) afforded the title compound as a brown solid (0.014 g, 28%). R*_f_ =* 0.49 (CH_2_Cl_2_:MeOH, 9:1); m.p. 51–52 °C; IR (ATR) ν_max_ 3181, 2933, 2833, 1480, 1247, 812 cm^−1^; ^1^H NMR (400 MHz, CDCl_3_) δ 8.57 (1H, br s, NH-8), 7.46 (1H, d, *J* = 1.0 Hz, H-2), 7.33 (1H, d, *J* = 8.6 Hz, H-9), 7.24–7.17 (3H, m, 2H-5′, H-6′), 7.14 (1H, br s, H-4), 7.04 (1H, d, *J* = 2.5 Hz, H-7), 6.97 (1H, d, *J* = 2.5 Hz, H-12), 6.92 (1H, dd, *J* = 8.6, 2.5 Hz, H-10), 6.91–6.89 (2H, m, 2H-4′), 4.15 (2H, t, *J* = 7.3 Hz, H_2_-1′), 3.81 (3H, s, OMe), 2.88 (2H, t, *J* = 7.3 Hz, H_2_-2′); ^13^C NMR (100 MHz, CDCl_3_) δ 155.0 (C-11), 137.74 (C-3′), 137.69 (C-2), 131.1 (C-8a), 128.9 (C-4), 128.79 (2C-5′), 128.76 (2C-4′), 128.0 (C-12a), 126.9 (C-6′), 126.0 (C-5), 124.4 (C-7), 113.5 (C-10), 112.3 (C-9), 105.2 (C-6), 101.0 (C-12), 56.0 (OMe), 46.8 (C-1′), 37.7 (C-2′); (–)-HRESIMS *m/z* 316.1452 [M−H]^−^ (calcd for C_20_H_18_N_3_O, 316.1455). 

#### 3.2.58. 6-Methoxy-3-(1-phenethyl-1*H*-imidazol-5-yl)-1*H*-indole (**57**) 

Using the general procedure, reaction of 6-methoxy-1*H*-indole-3-carbaldehyde (0.028 g, 0.157 mmol) with phenethylamine (20 µL, 0.157 mmol), K_2_CO_3_ (0.023 g, 0.157 mmol) and *p*-toluenesulfonylmethyl isocyanide (0.031 g, 0.157 mmol) followed by purification using silica gel column chromatography (EtOAc) afforded the title compound as a brown solid (0.015 g, 30%). R*_f_ =* 0.49 (CH_2_Cl_2_:MeOH, 9:1); m.p. 48–49 °C; IR (ATR) ν_max_ 3209, 2918, 2835, 1513, 1247, 812 cm^−1^; ^1^H NMR (400 MHz, CDCl_3_) δ 8.65 (1H, br s, NH-8), 7.44 (1H, d, *J* = 1.0 Hz, H-2), 7.41 (1H, d, *J* = 8.7 Hz, H-12), 7.24–7.17 (3H, m, 2H-5′, H-6′), 7.12 (1H, d, *J* = 1.0 Hz, H-4), 6.98 (1H, d, *J* = 2.5 Hz, H-7), 6.92 (1H, d, *J* = 2.5 Hz, H-9), 6.92–6.89 (2H, m, 2H-4′), 6.84 (1H, dd, *J* = 8.7, 2.5 Hz, H-11), 4.15 (2H, t, *J* = 7.0 Hz, H_2_-1′), 3.86 (3H, s, OMe), 2.87 (2H, t, *J* = 7.0 Hz, H_2_-2′); ^13^C NMR (100 MHz, CDCl_3_) δ 157.1 (C-10), 137.7 (C-3′), 137.6 (C-2), 136.9 (C-8a), 128.82 (C-4), 128.77 (2C-4′, 2C-5′), 126.9 (C-6′), 126.1 (C-5), 122.5 (C-7), 121.7 (C-12a), 120.3 (C-12), 110.7 (C-11), 105.4 (C-6), 94.9 (C-9), 55.8 (OMe), 46.9 (C-1′), 37.6 (C-2′); (–)-HRESIMS *m/z* 316.1448 [M−H]^−^ (calcd for C_20_H_18_N_3_O, 316.1455). 

#### 3.2.59. 5-Chloro-3-(1-(3-phenylpropyl)-1*H*-imidazol-5-yl)-1*H*-indole (**58**) 

Using the general procedure, reaction of 5-chloro-1*H*-indole-3-carbaldehyde (0.050 g, 0.278 mmol) with phenylpropylamine (0.040 mL, 0.278 mmol), K_2_CO_3_ (0.038 g, 0.278 mmol) and *p*-toluenesulfonylmethyl isocyanide (0.054 g, 0.278 mmol) in DMF (1 mL) followed by purification using silica gel column chromatography (EtOAc) afforded the title compound as a white oil (0.012 g, 13%). *R_f_ =* 0.08 (EtOAc); IR (ATR) ν_max_ 3027, 2929, 1603, 1456, 1226, 1110, 922, 889, 800, 750 cm^−1^; ^1^H NMR (400 MHz, DMSO-*d*_6_) δ 11.59 (1H, s, NH-8), 7.79 (1H, s, H-2), 7.55 (1H, d, *J* = 8.5 Hz, H-7), 7.47 (1H, d, *J* = 8.5 Hz, H-9), 7.43 (1H, d, *J* = 2.1 Hz, H-12), 7.19–7.09 (4H, m, H-10, 2H-6′, H-7′), 7.02–7.00 (3H, m, H-4, 2H-5′), 3.97 (2H, t, *J* = 7.5 Hz, H_2_-1′), 2.43 (2H, t, *J* = 7.5 Hz, H_2_-3′), 1.84 (2H, tt, *J* = 7.5, 7.5 Hz, H_2_-2′); ^13^C NMR (100 MHz, DMSO-*d*_6_) δ 140.8 (C-4′), 137.9 (C-2), 134.5 (C-8a), 128.2 (2C-6′), 128.0 (2C-5′), 127.8 (C-12a), 127.6 (C-4), 126.1 (C-7), 125.8 (C-5, C-7′), 124.3 (C-11), 121.7 (C-10), 117.8 (C-12), 113.4 (C-9), 103.6 (C-6), 44.1 (C-1′), 31.9 (C-3′), 31.7 (C-2′); (+)-HRESIMS *m/z* 336.1261 [M+H]^+^ (calcd for C_20_H_19_ClN_3_, 336.1262).

#### 3.2.60. 4-Fluoro-3-(1-(4-methoxybenzyl)-1*H*-imidazol-5-yl)-1*H*-indole (**59**) 

Using the general procedure, reaction of 4-fluoro-1*H*-indole-3-carbaldehyde (0.025 g, 0.156 mmol) with *p*-methoxybenzylamine (20 µL, 0.156 mmol), K_2_CO_3_ (0.022 g, 0.156 mmol) and *p*-toluenesulfonylmethyl isocyanide (0.030 g, 0.156 mmol) followed by purification using silica gel column chromatography (CH_2_Cl_2_:MeOH, 1:0→9:1) afforded the title compound as a brown solid (0.036 g, 72%). R*_f_* = 0.51 (CH_2_Cl_2_:MeOH, 9:1); m.p. 66–67 °C; IR (ATR) ν_max_ 3120, 2930, 2841, 1513, 1250, 736 cm^−1^; ^1^H NMR (400 MHz, CDCl_3_) δ 9.40 (1H, br s, NH-8), 7.55 (1H, d, *J* = 1.0 Hz, H-2), 7.22 (1H, d, *J* = 8.0 Hz, H-9), 7.14 (1H, ddd, *J* = 8.0, 7.4, 4.5 Hz, H-10), 7.12 (1H, br s, H-4), 7.06 (1H, d, *J* = 2.5 Hz, H-7), 6.95–6.90 (2H, m, 2H-3′), 6.81 (1H, ddd, *J* = 10.9, 7.4, 1.0 Hz, H-11), 6.79–6.74 (2H, m, 2H-4′), 5.00 (2H, s, H_2_-1′), 3.75 (3H, s, OMe); ^13^C NMR (100 MHz, CDCl_3_) δ 159.3 (C-5′), 156.6 (d, ^1^*J*_CF_ = 247.6 Hz, C-12), 139.1 (d, ^2^*J*_CF_ = 10.7 Hz, C-8a), 137.7 (C-2), 128.9 (2C-3′), 128.72 (C-2′), 128.70 (C-4), 126.6 (C-5), 125.4 (C-7), 123.3 (d, ^3^*J*_CF_ = 8.0 Hz, C-10), 116.2 (d, ^2^*J*_CF_ = 19.0 Hz, C-12a), 114.2 (2C-4′), 108.0 (d, ^4^*J*_CF_ = 3.8 Hz, C-9), 105.7 (d, ^2^*J*_CF_ = 19.5 Hz, C-11), 102.5 (C-6), 55.4 (OMe), 48.6 (C-1′); (–)-HRESIMS *m/z* 320.1210 [M−H]^−^ (calcd for C_19_H_15_FN_3_O, 320.1205). 

#### 3.2.61. 7-Fluoro-3-(1-(4-methoxybenzyl)-1*H*-imidazol-5-yl)-1*H*-indole (**60**) 

Using the general procedure, reaction of 7-fluoro-1*H*-indole-3-carbaldehyde (0.025 g, 0.156 mmol) with *p*-methoxybenzylamine (20 µL, 0.156 mmol), K_2_CO_3_ (0.022 g, 0.156 mmol) and *p*-toluenesulfonylmethyl isocyanide (0.030 g, 0.156 mmol) followed by purification using silica gel column chromatography (CH_2_Cl_2_:MeOH, 1:0→9:1) afforded the title compound as a brown solid (0.018 g, 36%). R*_f_* = 0.56 (CH_2_Cl_2_:MeOH, 9:1); m.p. 179–180 °C; IR (ATR) ν_max_ 3122, 2918, 2851, 1514, 1250, 785 cm^−1^; ^1^H NMR (400 MHz, CDCl_3_) δ 8.68 (1H, br s, NH-8), 7.64 (1H, d, *J* = 1.0 Hz, H-2), 7.33 (1H, d, *J* = 8.0 Hz, H-12), 7.23 (1H, d, *J* = 1.0 Hz, H-4), 7.07 (1H, ddd, *J* = 8.0, 8.0, 5.0 Hz, H-11), 7.03 (1H, d, *J* = 2.5 Hz, H-7), 6.96 (1H, ddd, *J* = 10.9, 8.0, 1.0 Hz, H-10), 6.94–6.90 (2H, m, 2H-3′), 6.83–6.78 (2H, m, 2H-4′), 5.05 (2H, s, H_2_-1′), 3.77 (3H, s, OMe); ^13^C NMR (100 MHz, CDCl_3_) δ 159.4 (C-5′), 149.7 (d, ^1^*J*_CF_ = 244.6 Hz, C-10), 138.1 (C-2), 130.9 (d, ^2^*J*_CF_ = 5.3 Hz, C-8a), 128.9 (C-9), 128.8 (C-4), 128.2 (2C-3′), 126.0 (C-5), 124.6 (d, ^2^*J*_CF_ = 13.7 Hz, C-12a), 124.5 (C-7), 121.0 (d, ^3^*J*_CF_ = 6.3 Hz, C-11), 115.5 (d, ^4^*J*_CF_ = 3.5 Hz, C-12), 114.4 (2C-4′), 107.7 (d, ^2^*J*_CF_ = 15.9 Hz, C-10), 106.1 (d, ^4^*J*_CF_ = 2.5 Hz, C-6), 55.4 (OMe), 48.5 (C-1′); (–)-HRESIMS *m/z* 320.1205 [M−H]^−^ (calcd for C_19_H_15_FN_3_O, 320.1205). 

#### 3.2.62. 4-Chloro-3-(1-(4-methoxybenzyl)-1*H*-imidazol-5-yl)-1*H*-indole (**61**) 

Using the general procedure, reaction of 4-chloro-1*H*-indole-3-carbaldehyde (0.027 g, 0.148 mmol) with *p*-methoxybenzylamine (19 µL, 0.148 mmol), K_2_CO_3_ (0.020 g, 0.148 mmol) and *p*-toluenesulfonylmethyl isocyanide (0.029 g, 0.148 mmol) followed by purification using silica gel column chromatography (CH_2_Cl_2_:MeOH, 1:0→9:1) afforded the title compound as a brown solid (0.034 g, 68%). R*_f_* = 0.57 (CH_2_Cl_2_:MeOH, 9:1); m.p. 74–75 °C; IR (ATR) ν_max_ 3115, 2925, 2838, 1514, 1251, 740 cm^−1^; ^1^H NMR (400 MHz, CDCl_3_) δ 9.93 (1H, br s, NH-8), 7.56 (1H, d, *J* = 1.0 Hz, H-2), 7.38–7.35 (1H, m, H-9), 7.14–7.10 (3H, m, H-7, H-10, H-11), 7.09 (1H, d, *J* = 1.0 Hz, H-4), 6.91–6.87 (2H, m, 2H-3′), 6.77–6.72 (2H, m, 2H-4′), 4.89 (2H, s, H_2_-1′), 3.74 (3H, s, OMe); ^13^C NMR (100 MHz, CDCl_3_) δ 159.3 (C-5′), 137.6 (C-8a), 137.1 (C-2), 129.4 (C-4), 129.2 (2C-3′), 128.5 (C-2′), 127.5 (C-7), 126.3 (C-5), 125.8 (C-12), 124.8 (C-12a), 123.1 (C-10), 121.2 (C-11), 114.1 (2C-4′), 110.8 (C-9), 103.6 (C-6), 55.4 (OMe), 48.9 (C-1′); (–)-HRESIMS *m/z* 336.0899 [M−H]^−^ (calcd for C_19_H_15_^35^ClN_3_O, 336.0909), *m/z* 338.0879 [M−H]^−^ (calcd for C_19_H_15_^37^ClN_3_O, 338.0886). 

#### 3.2.63. 7-Chloro-3-(1-(4-methoxybenzyl)-1*H*-imidazol-5-yl)-1*H*-indole (**62**) 

Using the general procedure, reaction of 7-chloro-1*H*-indole-3-carbaldehyde (0.027 g, 0.148 mmol) with *p*-methoxybenzylamine (19 µL, 0.148 mmol), K_2_CO_3_ (0.020 g, 0.148 mmol) and *p*-toluenesulfonylmethyl isocyanide (0.029 g, 0.148 mmol) followed by purification using silica gel column chromatography (EtOAc) afforded the title compound as a white solid (0.011 g, 22%). R*_f_* = 0.56 (CH_2_Cl_2_:MeOH, 9:1); m.p. 220–221 °C; IR (ATR) ν_max_ 2920, 2853, 1514, 1250, 1116, 743 cm^−1^; ^1^H NMR (400 MHz, CDCl_3_) δ 8.56 (1H, br s, NH-8), 7.64 (1H, br s, H-2), 7.48 (1H, d, *J* = 8.0 Hz, H-12), 7.26 (1H, dd, *J* = 7.8, 0.9 Hz, H-10), 7.23 (1H, d, *J* = 1.0 Hz, H-4), 7.10 (1H, dd, *J* = 8.0, 7.8 Hz, H-11), 7.05 (1H, d, *J* = 2.5 Hz, H-2), 6.95–6.90 (2H, m, 2H-3′), 6.84–6.79 (2H, m, 2H-4′), 5.05 (2H, s, H_2_-1′), 3.78 (3H, s, OMe); ^13^C NMR (100 MHz, CDCl_3_) δ 159.4 (C-5′), 138.3 (C-2), 133.4 (C-8a), 129.2 (C-4), 129.0 (C-12a/C-2′), 128.8 (C-12a/C-2′), 128.2 (2C-3′), 125.8 (C-5), 124.3 (C-7), 122.3 (C-10), 121.5 (C-11), 118.5 (C-12), 117.0 (C-9), 114.4 (2C-4′), 106.6 (C-6), 55.4 (OMe), 48.4 (C-1′); (–)-HRESIMS *m/z* 336.0904 [M−H]^−^ (calcd for C_19_H_15_^35^ClN_3_O, 336.0909), *m/z* 338.0876 [M−H]^−^ (calcd for C_19_H_15_^37^ClN_3_O, 338.0886). 

#### 3.2.64. 4-Bromo-3-(1-(4-methoxybenzyl)-1*H*-imidazol-5-yl)-1*H*-indole (**63**) 

Using the general procedure, reaction of 4-bromo-1*H*-indole-3-carbaldehyde (0.029 g, 0.131 mmol) with *p*-methoxybenzylamine (17 µL, 0.131 mmol), K_2_CO_3_ (0.018 g, 0.131 mmol) and *p*-toluenesulfonylmethyl isocyanide (0.026 g, 0.131 mmol) followed by purification using silica gel column chromatography (CH_2_Cl_2_:MeOH, 1:0→9:1) afforded the title compound as a brown solid (0.023 g, 46%). R*_f_* = 0.50 (CH_2_Cl_2_:MeOH, 9:1); m.p. 92–93 °C; IR (ATR) ν_max_ 3128, 2920, 2853, 1514, 1250, 1176, 740 cm^−1^; ^1^H NMR (400 MHz, CDCl_3_) δ 9.74 (1H, br s, NH-8), 7.59 (1H, d, *J* = 1.0 Hz, H-2), 7.42 (1H, dd, *J* = 8.0, 1.0 Hz, H-9), 7.32 (1H, dd, *J* = 7.6, 1.0 Hz, H-11), 7.13 (1H, d, *J* = 2.5 Hz, H-7), 7.08 (1H, d, *J* = 1.0 Hz, H-4), 7.07 (1H, dd, *J* = 8.0, 7.6 Hz, H-10), 6.93–6.87 (2H, m, 2H-3′), 6.77–6.73 (2H, m, 2H-4′), 4.86 (2H, s, H_2_-1′), 3.75 (3H, s, OMe); ^13^C NMR (100 MHz, CDCl_3_) δ 159.4 (C-5′), 137.2 (C-8a), 137.0 (C-2), 129.9 (C-4), 129.3 (2C-3′), 128.4 (C-2′), 127.8 (C-7), 126.2 (C-12a), 125.7 (C-5), 124.7 (C-11), 123.5 (C-10), 114.1 (2C-4′), 113.8 (C-12), 111.3 (C-9), 104.4 (C-6), 55.4 (OMe), 49.1 (C-1′); (–)-HRESIMS *m/z* 380.0408 [M−H]^−^ (calcd for C_19_H_15_^79^BrN_3_O, 380.0404), *m/z* 382.0389 [M−H]^−^ (calcd for C_19_H_15_^81^BrN_3_O, 382.0385). 

#### 3.2.65. 5-Methoxy-3-(1-(4-methoxybenzyl)-1*H*-imidazol-5-yl)-1*H*-indole (**64**) 

Using the general procedure, reaction of 5-methoxy-1*H*-indole-3-carbaldehyde (0.026 g, 0.150 mmol) with *p*-methoxybenzylamine (20 µL, 0.150 mmol), K_2_CO_3_ (0.021 g, 0.150 mmol) and *p*-toluenesulfonylmethyl isocyanide (0.029 g, 0.150 mmol) followed by purification using silica gel column chromatography (CH_2_Cl_2_:MeOH, 1:0→9:1) afforded the title compound as a brown solid (0.009 g, 18%). R*_f_ =* 0.54 (CH_2_Cl_2_:MeOH, 9:1); m.p. 67–68 °C; IR (ATR) ν_max_ 3316, 2929, 2838, 1250, 809 cm^−1^; ^1^H NMR (400 MHz, CDCl_3_) δ 8.44 (1H, br s, NH-8), 7.62 (1H, d, *J* = 1.0 Hz, H-2), 7.30 (1H, d, *J* = 8.7 Hz, H-9), 7.22 (1H, d, *J* = 1.0 Hz, H-4), 7.00 (1H, d, *J* = 2.5 Hz, H-7), 6.96 (1H, d, *J* = 2.5 Hz, H-12), 6.95–6.91 (2H, m, 2H-3′), 6.90 (1H, dd, *J* = 8.7, 2.5 Hz, H-10), 6.83–6.78 (2H, m, 2H-4′), 5.04 (2H, s, H_2_-1′), 3.77 (6H, s, 11-OMe, 5′-OMe); ^13^C NMR (100 MHz, CDCl_3_) δ 159.3 (C-5′), 155.0 (C-11), 138.0 (C-2), 131.0 (C-8a), 129.2 (C-2′), 128.6 (C-4), 128.3 (2C-3′), 127.9 (C-12a), 126.6 (C-5), 124.6 (C-7), 114.4 (2C-4′), 113.5 (C-10), 112.3 (C-9), 105.0 (C-6), 100.9 (C-12), 55.9 (11-OMe), 55.4 (5′-OMe), 48.3 (C-1′); (–)-HRESIMS *m/z* 332.1398 [M−H]^−^ (calcd for C_20_H_18_N_3_O_2_, 332.1405). 

#### 3.2.66. 6-Methoxy-3-(1-(4-methoxybenzyl)-1*H*-imidazol-5-yl)-1*H*-indole (**65**) 

Using the general procedure, reaction of 6-methoxy-1*H*-indole-3-carbaldehyde (0.026 g, 0.150 mmol) with *p*-methoxybenzylamine (20 µL, 0.150 mmol), K_2_CO_3_ (0.021 g, 0.150 mmol) and *p*-toluenesulfonylmethyl isocyanide (0.029 g, 0.150 mmol) followed by purification using silica gel column chromatography (CH_2_Cl_2_:MeOH, 1:0→9:1) afforded the title compound as a brown solid (0.008 g, 16%). R*_f_* = 0.54 (CH_2_Cl_2_:MeOH, 9:1); m.p. 65–66 °C; IR (ATR) ν_max_ 3332, 2917, 2848, 1612, 1250, 816 cm^−1^; ^1^H NMR (400 MHz, CDCl_3_) δ 8.41 (1H, br s, NH-8), 7.59 (1H, d, *J* = 1.0 Hz, H-2), 7.45 (1H, d, *J* = 8.8 Hz, H-12), 7.21 (1H, d, *J* = 1.0 Hz, H-4), 6.96–6.91 (2H, m, 2H-3′), 6.91 (1H, d, *J* = 2.5 Hz, H-7), 6.89 (1H, d, *J* = 2.2 Hz, H-9), 6.83 (1H, dd, *J* = 8.8, 2.2 Hz, H-11), 6.82–6.78 (2H, m, 2H-4′), 5.05 (2H, s, H_2_-1′), 3.85 (3H, s, 10-OMe), 3.77 (3H, s, 5′-OMe); ^13^C NMR (100 MHz, CDCl_3_) δ 159.3 (C-5′), 157.1 (C-10), 137.9 (C-2), 136.9 (C-8a), 129.1 (C-2′), 128.33 (C-4), 128.29 (2C-3′), 126.7 (C-5), 122.5 (C-7), 121.6 (C-12a), 120.4 (C-12), 114.4 (2C-4′), 110.7 (C-11), 105.2 (C-6), 94.9 (C-9), 55.8 (10-OMe), 55.4 (5′-OMe), 48.4 (C-1′); (–)-HRESIMS *m/z* 332.1400 [M−H]^−^ (calcd for C_20_H_18_N_3_O_2_, 332.1405). 

#### 3.2.67. 4-Fluoro-3-(1-(4-methoxyphenethyl)-1*H*-imidazol-5-yl)-1*H*-indole (**66**) 

Using the general procedure, reaction of 4-fluoro-1*H*-indole-3-carbaldehyde (0.024 g, 0.149 mmol) was reacted with *p*-methoxyphenethylamine hydrochloride (0.028 g, 0.149 mmol), K_2_CO_3_ (0.020 g, 0.149 mmol) and *p*-toluenesulfonylmethyl isocyanide (0.029 g, 0.149 mmol) followed by purification using silica gel column chromatography (EtOAc) afforded the title compound as a brown solid (0.047 g, 94%). R*_f_* = 0.39 (CH_2_Cl_2_:MeOH, 9:1); m.p. 56–57 °C; IR (ATR) ν_max_ 3116, 2929, 2851, 1512, 1247, 781 cm^−1^; ^1^H NMR (400 MHz, CDCl_3_) δ 10.23 (1H, br s, NH-8), 7.43 (1H, d, *J* = 1.0 Hz, H-2), 7.23 (1H, d, *J* = 8.0 Hz, H-9), 7.14 (1H, ddd, *J* = 8.0, 8.0, 5.0 Hz, H-10), 7.08 (1H, br s, H-4), 7.08 (1H, d, *J* = 2.5 Hz, H-7), 6.81–6.76 (2H, m, 2H-4′), 6.79 (1H, dd, *J* = 10.8, 8.0 Hz, H-11), 6.74–6.69 (2H, m, 2H-5′), 4.12 (2H, t, *J* = 7.0 Hz, H_2_-1′), 3.72 (3H, s, OMe), 2.79 (2H, t, *J* = 7.0 Hz, H_2_-2′); ^13^C NMR (100 MHz, CDCl_3_) δ 158.5 (C-6′), 156.5 (d, ^1^*J*_CF_ = 247.0 Hz, C-12), 139.4 (d, ^3^*J*_CF_ = 11.0 Hz, C-8a), 137.5 (C-2), 129.9 (C-3′), 129.7 (2C-4′), 128.8 (C-4), 126.4 (C-5), 125.5 (C-7), 123.0 (d, ^3^*J*_CF_ = 7.9 Hz, C-10), 116.1 (d, ^2^*J*_CF_ = 18.8 Hz, C-12a), 114.1 (2C-5′), 108.1 (d, ^4^*J*_CF_ = 3.8 Hz, C-9), 105.5 (d, ^2^*J*_CF_ = 19.2 Hz, C-11), 102.2 (C-6), 55.3 (OMe), 47.0 (C-1′), 36.3 (C-2′); (–)-HRESIMS *m/z* 334.1360 [M−H]^−^ (calcd for C_20_H_17_FN_3_O, 334.1361). 

#### 3.2.68. 7-Fluoro-3-(1-(4-methoxyphenethyl)-1*H*-imidazol-5-yl)-1*H*-indole (**67**) 

Using the general procedure, reaction of 7-fluoro-1*H*-indole-3-carbaldehyde (0.024 g, 0.149 mmol) was reacted with *p*-methoxyphenethylamine hydrochloride (0.028 g, 0.149 mmol), K_2_CO_3_ (0.020 g, 0.149 mmol) and *p*-toluenesulfonylmethyl isocyanide (0.029 g, 0.149 mmol) followed by purification using silica gel column chromatography (EtOAc) afforded the title compound as a brown solid (0.009 g, 18%). R*_f_* = 0.43 (CH_2_Cl_2_:MeOH, 9:1); m.p. 175–176 °C; IR (ATR) ν_max_ 3121, 2927, 2852, 1513, 1236, 784 cm^−1^; ^1^H NMR (400 MHz, CDCl_3_) δ 8.87 (1H, br s, NH-8), 7.48 (1H, br s, H-2), 7.29 (1H, d, *J* = 8.0 Hz, H-12), 7.13 (1H, br s, H-4), 7.09 (1H, d, *J* = 2.5 Hz, H-7), 7.07 (1H, ddd, *J* = 8.0, 8.0, 4.8 Hz, H-11), 6.98 (1H, ddd, *J* = 11.2, 8.0, 1.0 Hz, H-10), 6.82–6.77 (2H, m, 2H-4′), 6.76–6.71 (2H, m, 2H-5′), 4.11 (2H, t, *J* = 7.2 Hz, H_2_-1′), 3.75 (3H, s, OMe), 2.81 (2H, t, *J* = 7.2 Hz, H_2_-2′); ^13^C NMR (100 MHz, CDCl_3_) δ 158.6 (C-6′), 149.7 (d, ^1^*J*_CF_ = 245.1 Hz, C-9), 137.9 (C-2), 131.0 (d, ^3^*J*_CF_ = 5.3 Hz, C-12a), 129.7 (2C-4′), 129.6 (C-3′), 129.1 (C-4), 125.3 (C-5), 124.6 (d, ^2^*J*_CF_ = 14.2 Hz, C-8a), 124.4 (C-7), 121.0 (d, ^3^*J*_CF_ = 5.9 Hz, C-11), 115.5 (d, ^4^*J*_CF_ = 3.3 Hz, C-12), 114.2 (2C-5′), 107.7 (d, ^2^*J*_CF_ = 16.2 Hz, C-10), 106.4 (d, ^4^*J*_CF_ = 2.9 Hz, C-6), 55.4 (OMe), 47.1 (C-1′), 36.8 (C-2′); (–)-HRESIMS *m/z* 334.1356 [M−H]^−^ (calcd for C_20_H_17_FN_3_O, 334.1361). 

#### 3.2.69. 4-Chloro-3-(1-(4-methoxyphenethyl)-1*H*-imidazol-5-yl)-1*H*-indole (**68**) 

Using the general procedure, reaction of 4-chloro-1*H*-indole-3-carbaldehyde (0.026 g, 0.142 mmol) was reacted with *p*-methoxyphenethylamine hydrochloride (0.027 g, 0.142 mmol), K_2_CO_3_ (0.020 g, 0.142 mmol) and *p*-toluenesulfonylmethyl isocyanide (0.028 g, 0.142 mmol) followed by purification using silica gel column chromatography (EtOAc) afforded the title compound as a yellow solid (0.038 g, 76%). R*_f_ =* 0.43 (CH_2_Cl_2_:MeOH, 9:1); m.p. 76–77 °C; IR (ATR) ν_max_ 3107, 2925, 2853, 1512, 1247, 823 cm^−1^; ^1^H NMR (400 MHz, CDCl_3_) δ 10.31 (1H, br s, NH-8), 7.48 (1H, d, *J* = 1.0 Hz, H-2), 7.36 (1H, dd, *J*= 7.5, 1.5 Hz, H-9), 7.13 (1H, dd, *J* = 7.5, 7.5 Hz, H-10), 7.10 (1H, dd, *J* = 7.5, 1.5 Hz, H-11), 7.09 (1H, d, *J* = 2.5 Hz, H-7), 7.06 (1H, d, *J* = 1.0 Hz, H-4), 6.80–6.75 (2H, m, 2H-4′), 6.75–6.70 (2H, m, 2H-5′), 4.03 (2H, t, *J* = 7.1 Hz, H_2_-1′), 3.73 (3H, s, OMe), 2.79 (2H, t, *J* = 7.1 Hz, H_2_-2′); ^13^C NMR (100 MHz, CDCl_3_) δ 158.5 (C-6′), 137.7 (C-8a), 137.1 (C-2), 130.0 (C-3′), 129.8 (2C-4′), 129.6 (C-4), 127.4 (C-7), 126.0 (C-5/C-12), 125.8 (C-5/C-12), 124.8 (C-12a), 123.0 (C-10), 121.1 (C-11), 114.1 (2C-5′), 110.8 (C-9), 103.6 (C-6), 55.3 (OMe), 47.1 (C-1′), 36.4 (C-2′); (–)-HRESIMS *m/z* 350.1061 [M−H]^−^ (calcd for C_20_H_17_^35^ClN_3_O, 350.1066), *m/z* 352.1036 [M−H]^−^ (calcd for C_20_H_17_^37^ClN_3_O, 352.1043). 

#### 3.2.70. 7-Chloro-3-(1-(4-methoxyphenethyl)-1*H*-imidazol-5-yl)-1*H*-indole (**69**) 

Using the general procedure, reaction of 7-chloro-1*H*-indole-3-carbaldehyde (0.026 g, 0.142 mmol) was reacted with *p*-methoxyphenethylamine hydrochloride (0.027 g, 0.142 mmol), K_2_CO_3_ (0.020 g, 0.142 mmol) and *p*-toluenesulfonylmethyl isocyanide (0.028 g, 0.142 mmol) followed by purification using silica gel column chromatography (EtOAc) afforded the title compound as a yellow solid (0.018 g, 36%). R*_f_* = 0.49 (CH_2_Cl_2_:MeOH, 9:1); m.p. 59–60 °C; IR (ATR) ν_max_ 3112, 2921, 2835, 1512, 1247, 821 cm^−1^; ^1^H NMR (400 MHz, CDCl_3_) δ 8.95 (1H, br s, NH-8), 7.47 (1H, d, *J* = 1.0 Hz, H-2), 7.43 (1H, d, *J* = 8.0 Hz, H-12), 7.27 (1H, dd, *J* = 7.5, 1.0 Hz, H-10), 7.13 (1H, d, *J* = 1.0 Hz, H-4), 7.11 (1H, d, *J* = 2.5 Hz, H-7), 7.10 (1H, dd, *J* = 8.0, 7.5 Hz, H-11), 6.82–6.77 (2H, m, 2H-4′), 6.76–6.71 (2H, m 2H-5′), 4.11 (2H, t, *J* = 7.0 Hz, H_2_-1′), 3.75 (3H, s, OMe), 2.81 (2H, t, *J* = 7.0 Hz, H_2_-2′); ^13^C NMR (100 MHz, CDCl_3_) δ 158.6 (C-6′), 137.9 (C-2), 133.4 (C-8a), 129.7 (2C-4′), 129.6 (C-3′), 129.2 (C-4), 128.9 (C-12a), 125.3 (C-5), 124.3 (C-7), 122.3 (C-10), 121.4 (C-11), 118.4 (C-12), 117.1 (C-9), 114.2 (2C-5′), 106.7 (C-6), 55.4 (OMe), 47.0 (C-1′), 36.8 (C-2′); (–)-HRESIMS *m/z* 350.1068 [M−H]^−^ (calcd for C_20_H_17_^35^ClN_3_O, 350.1066), *m/z* 352.1050 [M−H]^−^ (calcd for C_20_H_17_^37^ClN_3_O, 352.1043). 

#### 3.2.71. 4-Bromo-3-(1-(4-methoxyphenethyl)-1*H*-imidazol-5-yl)-1*H*-indole (**70**) 

Using the general procedure, reaction of 4-bromo-1*H*-indole-3-carbaldehyde (0.028 g, 0.126 mmol) was reacted with *p*-methoxyphenethylamine hydrochloride (0.024 g, 0.126 mmol), K_2_CO_3_ (0.018 g, 0.126 mmol) and *p*-toluenesulfonylmethyl isocyanide (0.025 g, 0.126 mmol) followed by purification using silica gel column chromatography (EtOAc) afforded the title compound as a yellow solid (0.042 g, 84%). R*_f_* = 0.44 (CH_2_Cl_2_:MeOH, 9:1); m.p. 83–84 °C; IR (ATR) ν_max_ 3107, 2927, 2833, 1513, 1247, 822 cm^−1^; ^1^H NMR (400 MHz, CDCl_3_) δ 9.82 (1H, br s, NH-8), 7.50 (1H, d, *J* = 1.0 Hz, H-2), 7.41 (1H, dd, *J* = 8.1, 1.0 Hz, H-9), 7.30 (1H, dd, *J* = 7.7, 1.0 Hz, H-11), 7.08 (1H, d, *J* = 2.5 Hz, H-7), 7.07 (1H, dd, *J* = 8.1, 7.7 Hz, H-10), 7.05 (1H, d, *J* = 1.0 Hz, H-4), 6.80–6.75 (2H, m, 2H-4′), 6.75–6.70 (2H, m, 2H-5′), 4.00 (2H, t, *J* = 7.1 Hz, H_2_-1′), 3.74 (3H, s, OMe), 2.80 (2H, *J* = 7.1 Hz, H_2_-2′); ^13^C NMR (100 MHz, CDCl_3_) δ 158.5 (C-6′), 137.3 (C-8a), 137.0 (C-2), 130.2 (C-4), 130.1 (C-3′), 129.8 (2C-4′), 127.6 (C-7), 126.2 (C-12a), 125.3 (C-5), 124.7 (C-11), 123.4 (C-10), 114.1 (2C-5′), 113.8 (C-12), 111.2 (C-9), 104.7 (C-6), 55.4 (OMe), 47.4 (C-1′), 36.4 (C-2′); (–)-HRESIMS *m/z* 394.0548 [M−H]^−^ (calcd for C_20_H_17_^79^BrN_3_O, 394.0560), *m/z* 396.0530 [M−H]^−^ (calcd for C_20_H_17_^81^BrN_3_O, 396.0542). 

#### 3.2.72. 5-Methoxy-3-(1-(4-methoxyphenethyl)-1*H*-imidazol-5-yl)-1*H*-indole (**71**) 

Using the general procedure, reaction of 5-methoxy-1*H*-indole-3-carbaldehyde (0.025 g, 0.144 mmol) was reacted with *p*-methoxyphenethylamine hydrochloride (0.027 g, 0.144 mmol), K_2_CO_3_ (0.020 g, 0.144 mmol) and *p*-toluenesulfonylmethyl isocyanide (0.028 g, 0.144 mmol) followed by purification using silica gel column chromatography (EtOAc) afforded the title compound as a yellow solid (0.026 g, 52%). R*_f_* = 0.44 (CH_2_Cl_2_:MeOH, 9:1); m.p. 48–49 °C; IR (ATR) ν_max_ 3221, 2932, 2835, 1454, 1248, 698 cm^−1^; ^1^H NMR (400 MHz, CDCl_3_) δ 8.85 (1H, br s, NH-8), 7.45 (1H, d, *J* = 1.0 Hz, H-2), 7.32 (1H, d, *J* = 8.5 Hz, H-9), 7.14 (1H, d, *J* = 1.0 Hz, H-4), 7.07 (1H, d, *J* = 2.5 Hz, H-7), 6.97 (1H, d, *J* = 2.5 Hz, H-12), 6.92 (1H, dd, *J* = 8.5, 2.5 Hz, H-10), 6.83–6.78 (2H, m, 2H-4′), 6.76–6.71 (2H, m, 2H-5′), 4.11 (2H, t, *J* = 7.2 Hz, H_2_-1′), 3.81 (3H, s, 11-OMe), 3.74 (3H, s, 6′-OMe), 2.81 (2H, t, *J* = 7.2 Hz, H_2_-2′); ^13^C NMR (100 MHz, CDCl_3_) δ 158.6 (C-6′), 155.0 (C-11), 137.7 (C-2), 131.2 (C-8a), 129.7 (C-3′, 2C-4′), 128.8 (C-4), 128.0 (C-12a), 126.0 (C-5), 124.5 (C-7), 114.2 (2C-5′), 113.4 (C-10), 112.4 (C-9), 105.1 (C-6), 100.9 (C-12), 56.0 (11-OMe), 55.3 (6′-OMe), 47.0 (C-1′), 36.7 (C-2′); (–)-HRESIMS *m/z* 346.1548 [M−H]^−^ (calcd for C_21_H_20_N_3_O_2_, 346.1561). 

#### 3.2.73. 6-Methoxy-3-(1-(4-methoxyphenethyl)-1*H*-imidazol-5-yl)-1*H*-indole (**72**) 

Using the general procedure, reaction of 6-methoxy-1*H*-indole-3-carbaldehyde (0.025 g, 0.144 mmol) was reacted with *p*-methoxyphenethylamine hydrochloride (0.027 g, 0.144 mmol), K_2_CO_3_ (0.020 g, 0.144 mmol) and *p*-toluenesulfonylmethyl isocyanide (0.028 g, 0.144 mmol) followed by purification using silica gel column chromatography (EtOAc) afforded the title compound as a brown solid (0.040 g, 80%). R*_f_* = 0.43 (CH_2_Cl_2_:MeOH, 9:1); m.p. 45–46 °C; IR (ATR) ν_max_ 3115, 2929, 2834, 1512, 1245, 809 cm^−1^; ^1^H NMR (400 MHz, CDCl_3_) δ 8.95 (1H, br s, NH-8), 7.42 (1H, d, *J* = 1.0 Hz, H-2), 7.41 (1H, d, *J* = 8.8 Hz, H-12), 7.13 (1H, d, *J* = 1.0 Hz, H-4), 7.00 (1H, d, *J* = 2.5 Hz, H-7), 6.92 (1H, d, *J* = 2.5 Hz, H-9), 6.84 (1H, dd, *J* = 8.8, 2.5 Hz, H-11), 6.83–6.78 (2H, m, 2H-4′), 6.76–6.71 (2H, m, 2H-5′), 4.12 (2H, t, *J* = 7.1 Hz, H_2_-1′), 3.86 (3H, s, 10-OMe), 3.73 (3H, s, 6′-OMe), 2.81 (2H, t, *J* = 7.1 Hz, H_2_-2′); ^13^C NMR (100 MHz, CDCl_3_) δ 158.5 (C-6′), 157.0 (C-10), 137.6 (C-2), 137.0 (C-8a), 129.7 (C-3′, 2C-4′), 128.6 (C-4), 126.2 (C-5), 122.5 (C-7), 121.7 (C-12a), 120.3 (C-12), 114.1 (2C-5′), 110.6 (C-11), 105.3 (C-6), 95.0 (C-9), 55.8 (10-OMe), 55.3 (6′-OMe), 47.1 (C-1′), 36.6 (C-2′); (–)-HRESIMS *m/z* 346.1554 [M−H]^−^ (calcd for C_21_H_20_N_3_O_2_, 346.1561). 

#### 3.2.74. 3-(1-((1*H*-Indol-3-yl)methyl)-1*H*-imidazol-5-yl)-4-fluoro-1*H*-indole (**73**) 

Using the general procedure, reaction of 4-fluoro-1*H*-indole-3-carbaldehyde (0.025 g, 0.151 mmol) was reacted with (1*H*-indol-3-yl)methanamine (0.022 g, 0.151 mmol), K_2_CO_3_ (0.021 g, 0.151 mmol) and *p*-toluenesulfonylmethyl isocyanide (0.030 g, 0.151 mmol) followed by purification using silica gel column chromatography (EtOAc) afforded the title compound as a brown solid (0.040 g, 80%). R*_f_* = 0.34 (CH_2_Cl_2_:MeOH, 9:1); m.p. 164–165 ℃; IR (ATR) ν_max_ 3404, 3132, 2923, 1108, 738 cm^−1^; ^1^H NMR (400 MHz, DMSO-*d*_6_) δ 11.74 (1H, br s, NH-8), 11.00 (1H, br s, NH-4′), 7.65 (1H, d, *J* = 1.0 Hz, H-2), 7.45 (1H, d, *J* = 2.5 Hz, H-7), 7.32 (2H, d, *J* = 7.9 Hz, H-9, H-11), 7.21 (1H, d, *J* = 7.9 Hz, H-8′), 7.14 (1H, ddd, *J* = 7.9, 7.9, 5.0 Hz, H-10), 7.05 (1H, ddd, *J* = 7.9, 7.0, 1.0 Hz, H-6′), 6.99 (1H, d, *J* = 2.5 Hz, H-3′), 6.90 (1H, br s, H-4), 6.90 (1H, ddd, *J* = 7.9, 7.0, 1.0 Hz, H-7′), 6.84 (1H, dd, *J* = 11.5, 7.9 Hz, H-5′), 5.23 (2H, br s, H_2_-1′); ^13^C NMR (100 MHz, DMSO-*d*_6_) δ 155.8 (d, ^1^*J*_CF_ = 244.9 Hz, C-12), 139.1 (d, ^3^*J*_CF_ = 10.8 Hz, C-8a), 137.4 (C-2), 136.1 (C-4a′), 128.0 (C-4), 126.2 (C-7), 125.93 (C-5), 125.89 (C-8a′), 124.4 (C-3′), 122.2 (d, ^3^*J*_CF_ = 7.8 Hz, C-10), 121.3 (C-6′), 118.8 (C-7′), 117.9 (C-8′), 115.3 (d, ^2^*J*_CF_ = 18.3 Hz, C-12a), 111.6 (C-5′), 110.4 (C-2′), 108.5 (d, ^4^*J*_CF_ = 3.8 Hz, C-9), 104.6 (d, ^2^*J*_CF_ = 19.5 Hz, C-11), 101.3 (C-6), 40.0 (C-1′); (–)-HRESIMS *m/z* 329.1207 [M−H]^−^ (calcd for C_20_H_14_FN_4_, 329.1208). 

#### 3.2.75. 3-(1-((1*H*-Indol-3-yl)methyl)-1*H*-imidazol-5-yl)-7-fluoro-1*H*-indole (**74**) 

Using the general procedure, reaction of 7-fluoro-1*H*-indole-3-carbaldehyde (0.025 g, 0.151 mmol) was reacted with (1*H*-indol-3-yl)methanamine (0.022 g, 0.151 mmol), K_2_CO_3_ (0.021 g, 0.151 mmol) and *p*-toluenesulfonylmethyl isocyanide (0.030 g, 0.151 mmol) followed by purification by trituration with CH_2_Cl_2_ afforded the title compound as a light brown solid (0.026 g, 52%). R*_f_* = 0.41 (CH_2_Cl_2_:MeOH, 9:1); m.p. 186–187 °C; IR (ATR) ν_max_ 3405, 3120, 2923, 1108, 741 cm^−1^; ^1^H NMR (400 MHz, DMSO-*d*_6_) δ 11.94 (1H, br s, NH-8), 11.02 (1H, br s, NH-4′), 7.73 (1H, s, H-2), 7.58 (1H, s, H-7), 7.39 (1H, d, *J* = 7.5 Hz, H-4′), 7.34 (1H, d, *J* = 8.0 Hz, H-5′), 7.28 (1H, d, *J* = 8.0 Hz, H-8′), 7.09–7.03 (2H, m, H-4, H-6′), 7.06–7.01 (1H, m, H-11), 7.03–6.96 (2H, m, H-10, H-3′), 6.92 (1H, dd, *J* = 8.0, 7.5 Hz, H-7′), 5.37 (2H, s, H_2_-1′); ^13^C NMR (100 MHz, DMSO-*d*_6_) δ 149.3 (d, ^1^*J*_FC_ = 243.7 Hz, C-9), 137.9 (C-2), 136.2 (C-4a′), 130.4 (d, ^3^*J*_CF_ = 5.9 Hz, C-12a), 127.3 (C-4), 125.8 (C-8a′), 125.6 (C-5), 125.3 (C-7), 124.2 (C-3′), 124.0 (d, ^2^*J*_CF_ = 13.9 Hz, C-8a), 121.4 (C-6′), 119.9 (d, ^3^*J*_CF_ = 6.3 Hz, C-11), 118.9 (C-7′), 118.0 (C-8′), 115.2 (d, ^4^*J*_CF_ = 3.0 Hz, C-12), 111.6 (C-5′), 110.5 (C-2′), 106.5 (d, ^2^*J*_CF_ = 16.1 Hz, C-10), 105.2 (d, ^4^*J*_CF_ = 2.2 Hz, C-6), 40.5 (C-1′); (–)-HRESIMS *m/z* 329.1205 [M−H]^−^ (calcd for C_20_H_14_FN_4_, 329.1208). 

#### 3.2.76. 3-(1-((1*H*-Indol-3-yl)methyl)-1*H*-imidazol-5-yl)-4-chloro-1*H*-indole (**75**) 

Using the general procedure, reaction of 4-chloro-1*H*-indole-3-carbaldehyde (0.026 g, 0.144 mmol) was reacted with (1*H*-indol-3-yl)methanamine (0.021 g, 0.144 mmol), K_2_CO_3_ (0.020 g, 0.144 mmol) and *p*-toluenesulfonylmethyl isocyanide (0.028 g, 0.144 mmol) followed by purification using silica gel column chromatography (EtOAc) afforded the title compound as a light brown solid (0.032 g, 64%). R*_f_ =* 0.37 (CH_2_Cl_2_:MeOH, 9:1); m.p. 195-196 °C; IR (ATR) ν_max_ 3412, 3109, 2923, 1487, 1106, 740 cm^−1^; ^1^H NMR (400 MHz, DMSO-*d*_6_) δ 11.77 (1H, d, *J* = 2.5 Hz, NH-8), 11.00 (1H, br s, NH-4′), 7.64 (1H, br s, H-2), 7.48 (1H, dd, *J* = 8.0, 1.0 Hz, H-9), 7.44 (1H, d, *J* = 2.5 Hz, H-7), 7.32 (1H, d, *J* = 8.0 Hz, H-5′), 7.20 (1H, d, *J* = 8.0 Hz, H-8′), 7.16 (1H, dd, *J* = 8.0, 7.5 Hz, H-10), 7.11 (1H, dd, *J* = 7.5, 1.0 Hz, H-11), 7.05 (1H, ddd, *J* = 8.0, 7.5, 1.0 Hz, H-6′), 6.97 (1H, d, *J* = 2.6 Hz, H-3′), 6.91 (1H, ddd, *J* = 8.0, 7.5, 1.0 Hz, H-7′), 6.84 (1H, br s, H-4), 5.06 (2H, s, H_2_-1′); ^13^C NMR (100 MHz, DMSO-*d*_6_) δ 137.6 (C-8a), 137.0 (C-2), 136.1 (C-4a′), 128.9 (C-4), 128.4 (C-7), 126.0 (C-8a′), 125.3 (C-5), 124.6 (C-3′), 124.2 (C-4/C-12a), 124.1 (C-4/C-12a), 122.4 (C-10), 121.3 (C-6′), 120.2 (C-11), 118.8 (C-7′), 117.9 (C-8′), 111.5 (C-5′), 111.3 (C-9), 110.1 (C-2′), 102.6 (C-6), 40.0 (C-1′); (–)-HRESIMS *m/z* 345.0919 [M−H]^−^ (calcd for C_20_H_14_^35^ClN_4_, 345.0912), *m/z* 347.0886 [M−H]^−^ (calcd for C_20_H_14_^37^ClN_4_, 347.0889).

#### 3.2.77. 3-(1-((1*H*-Indol-3-yl)methyl)-1*H*-imidazol-5-yl)-5-chloro-1*H*-indole (**76**) 

Using the general procedure, reaction of 5-chloro-1*H*-indole-3-carbaldehyde (0.026 g, 0.144 mmol) was reacted with (1*H*-indol-3-yl)methanamine (0.021 g, 0.144 mmol), K_2_CO_3_ (0.020 g, 0.144 mmol) and *p*-toluenesulfonylmethyl isocyanide (0.028 g, 0.144 mmol) followed by purification using silica gel column chromatography (EtOAc) afforded the title compound as a yellow solid (0.027 g, 54%). R*_f_* = 0.41 (CH_2_Cl_2_:MeOH, 9:1); m.p. 107–108 ℃; IR (ATR) ν_max_ 3404, 3227, 2929, 1660, 1458, 744 cm^−1^; ^1^H NMR (400 MHz, DMSO-*d*_6_) δ 11.62 (1H, br s, NH-8), 11.60 (1H, br s, NH-4′), 7.72 (1H, d, *J* = 1.0 Hz, H-2), 7.60 (1H, d, *J* = 2.5 Hz, H-7), 7.49 (1H, d, *J* = 2.0 Hz, H-12), 7.47 (1H, d, *J* = 8.8 Hz, H-9), 7.33 (1H, d, *J* = 8.2 Hz, H-5′), 7.27 (1H, d, *J* = 8.1 Hz, H-8′), 7.16 (1H, dd, *J* = 8.8, 2.0 Hz, H-10), 7.06 (1H, d, *J* = 1.0 Hz, H-4), 7.06 (1H, ddd, *J* = 8.2, 7.0, 1.0 Hz, H-6′), 6.98 (1H, d, *J* = 2.5 Hz, H-3′), 6.91 (1H, ddd, *J* = 8.1, 7.0, 1.0 Hz, H-7′), 5.34 (2H, s, H_2_-1′); ^13^C NMR (100 MHz, DMSO-*d*_6_) δ 137.9 (C-2), 136.1 (C-4a′), 134.5 (C-8a), 127.6 (C-12a), 127.2 (C-4), 126.0 (C-7), 125.8 (C-8a′), 125.4 (C-5), 124.3 (C-11), 124.2 (C-3′), 121.7 (C-10), 121.4 (C-6′), 118.9 (C-7′), 118.02 (C-12/C-8′), 117.99 (C-12/C-8′), 113.4 (C-9), 111.6 (C-5′), 110.5 (C-2′), 103.9 (C-6), 40.4 (C-1′); (–)-HRESIMS *m/z* 345.0910 [M−H]^−^ (calcd for C_20_H_14_^35^ClN_4_, 345.0912), *m/z* 347.0881 [M−H]^−^ (calcd for C_20_H_14_^37^ClN_4_, 347.0889).

#### 3.2.78. 3-(1-((1*H*-Indol-3-yl)methyl)-1*H*-imidazol-5-yl)-7-chloro-1*H*-indole (**77**) 

Using the general procedure, reaction of 7-chloro-1*H*-indole-3-carbaldehyde (0.026 g, 0.144 mmol) was reacted with (1*H*-indol-3-yl)methanamine (0.021 g, 0.144 mmol), K_2_CO_3_ (0.020 g, 0.144 mmol) and *p*-toluenesulfonylmethyl isocyanide (0.028 g, 0.144 mmol) followed by purification using silica gel column chromatography (EtOAc) afforded the title compound as a pale yellow solid (0.023 g, 46%). R*_f_* = 0.46 (CH_2_Cl_2_:MeOH, 9:1); m.p. 165–166 °C; IR (ATR) ν_max_ 3412, 3116, 2921, 1108, 741 cm^−1^; ^1^H NMR (400 MHz, DMSO-*d*_6_) δ 11.80 (1H, br s, NH-8), 11.01 (1H, br s, NH-4′), 7.74 (1H, d, *J* = 1.0 Hz, H-2), 7.58 (1H, s, H-7), 7.54 (1H, dd, *J* = 8.0, 1.0 Hz, H-12), 7.34 (1H, d, *J* = 8.5 Hz, H-5′), 7.29 (1H, d, *J* = 8.0 Hz, H-8′), 7.26 (1H, dd, *J* = 7.5, 1.0 Hz, H-10), 7.08 (1H, d, *J* = 1.0 Hz, H-4), 7.08 (1H, dd, *J* = 8.5, 7.0 Hz, H-11), 7.07 (1H, ddd, *J* = 8.5, 7.2, 1.0 Hz, H-6′), 6.98 (1H, d, *J* = 2.5 Hz, H-3′), 6.92 (1H, ddd, *J* = 8.0, 7.2, 1.0 Hz, H-7′), 5.37 (2H, s, H_2_-1′); ^13^C NMR (100 MHz, DMSO-*d*_6_) δ 137.9 (C-2), 136.2 (C-4a′), 132.9 (C-8a), 128.4 (C-12a), 127.3 (C-4), 125.8 (C-8a′), 125.5 (C-5), 125.4 (C-7), 124.1 (C-3′), 121.4 (C-6′), 121.3 (C-10), 120.6 (C-11), 118.9 (C-7′), 118.1 (C-12/C-8′), 118.0 (C-12/C-8′), 116.2 (C-9), 111.6 (C-5′), 110.5 (C-2′), 105.4 (C-6), 40.5 (C-1′); (–)-HRESIMS *m/z* 345.0919 [M−H]^−^ (calcd for C_20_H_14_^35^ClN_4_, 345.0912), *m/z* 347.0894 [M−H]^−^ (calcd for C_20_H_14_^37^ClN_4_, 347.0889).

#### 3.2.79. 3-(1-((1*H*-Indol-3-yl)methyl)-1*H*-imidazol-5-yl)-4-bromo-1*H*-indole (**78**) 

Using the general procedure, reaction of 4-bromo-1*H*-indole-3-carbaldehyde (0.029 g, 0.128 mmol) was reacted with (1*H*-indol-3-yl)methanamine (0.019 g, 0.128 mmol), K_2_CO_3_ (0.018 g, 0.128 mmol) and *p*-toluenesulfonylmethyl isocyanide (0.025 g, 0.128 mmol) followed by purification using silica gel column chromatography (EtOAc) afforded the title compound as a yellow solid (0.036 g, 72%). R*_f_ =* 0.37 (CH_2_Cl_2_:MeOH, 9:1); m.p. 201–202 °C; IR (ATR) ν_max_ 3410, 3110, 2922, 1489, 1107, 740 cm^−1^; ^1^H NMR (400 MHz, DMSO-*d*_6_) δ 11.78 (1H, br s, NH-8), 11.01 (1H, br s, NH-4′), 7.66 (1H, br s, H-2), 7.53 (1H, d, *J* = 7.9 Hz, H-9), 7.45 (1H, d, *J* = 2.5 Hz, H-7), 7.33 (1H, d, *J* = 8.0 Hz, H-5′), 7.28 (1H, d, *J* = 7.5 Hz, H-12), 7.23 (1H, d, *J* = 8.0 Hz, H-8′), 7.10 (1H, dd, *J* = 7.9, 7.5 Hz, H-10), 7.06 (1H, ddd, *J* = 8.0, 7.5, 1.0 Hz, H-6′), 6.98 (1H, d, *J* = 2.5 Hz, H-3′), 6.92 (1H, ddd, *J* = 8.0, 7.5, 1.0 Hz, H-7′), 6.85 (1H, br s, H-4), 5.02 (2H, s, H_2_-1′); ^13^C NMR (100 MHz, DMSO-*d*_6_) δ 137.2 (C-8a), 136.9 (C-2), 136.1 (C-4a′), 129.3 (C-4), 128.7 (C-7), 126.0 (C-8a′), 125.6 (C-12a), 124.8 (C-5), 124.6 (C-3′), 123.5 (C-11), 122.7 (C-10), 121.3 (C-6′), 118.8 (C-7′), 118.0 (C-8′), 112.5 (C-12), 111.8 (C-9), 111.5 (C-5′), 110.0 (C-2′), 103.4 (C-6), 40.1 (C-1′); (–)-HRESIMS *m/z* 389.0412 [M−H]^–^ (calcd for C_20_H_14_^79^BrN_4_, 389.0407), *m/z* 391.0394 [M−H]^–^ (calcd for C_20_H_14_^81^BrN_4_, 391.0389). 

#### 3.2.80. 3-(1-((1*H*-Indol-3-yl)methyl)-1*H*-imidazol-5-yl)-5-methoxy-1*H*-indole (**79**) 

Using the general procedure, reaction of 5-methoxy-1*H*-indole-3-carbaldehyde (0.026 g, 0.146 mmol) was reacted with (1*H*-indol-3-yl)methanamine (0.021 g, 0.146 mmol), K_2_CO_3_ (0.020 g, 0.146 mmol) and *p*-toluenesulfonylmethyl isocyanide (0.029 g, 0.146 mmol) followed by purification using silica gel column chromatography (EtOAc) afforded the title compound as a light brown solid (0.019 g, 38%). R*_f_ =* 0.37 (CH_2_Cl_2_:MeOH, 9:1); m.p. 97–98 °C; IR (ATR) ν_max_ 3402, 3116, 2926, 1481, 1212, 744 cm^−1^; ^1^H NMR (400 MHz, CDCl_3_) δ 8.57 (1H, br s, NH-8), 8.49 (1H, br s, NH-4′), 7.64 (1H, d, *J* = 1.0 Hz, H-2), 7.37 (1H, d, *J* = 7.7 Hz, H-8′), 7.35 (1H, d, *J* = 8.0 Hz, H-5′), 7.29 (1H, d, *J* = 8.9 Hz, H-9), 7.23 (1H, d, *J* = 1.0 Hz, H-4), 7.19 (1H, ddd, *J* = 8.0, 7.0, 1.0 Hz, H-6′), 7.11 (1H, d, *J* = 2.5 Hz, H-7), 7.07 (1H, ddd, *J* = 7.7, 7.0, 1.0 Hz, H-7′), 7.04 (1H, d, *J* = 2.5 Hz, H-12), 6.90 (1H, dd, *J* = 8.9, 2.5 Hz, H-10), 6.83 (1H, d, *J* = 1.0 Hz, H-3′), 5.26 (2H, s, H_2_-1′), 3.75 (3H, s, OMe); ^13^C NMR (100 MHz, CDCl_3_) δ 155.0 (C-11), 138.0 (C-2), 136.5 (C-4a′), 131.1 (C-8a), 128.4 (C-4), 127.9 (C-12a), 126.5 (C-5), 126.0 (C-8a′), 124.4 (C-7), 123.2 (C-3′), 122.8 (C-6′), 120.3 (C-7′), 118.6 (C-8′), 113.5 (C-10), 112.3 (C-9), 112.2 (C-2′), 111.6 (C-5′), 105.4 (C-6), 101.1 (C-12), 55.9 (OMe), 41.3 (C-1′); (+)-HRESIMS *m/z* 343.1540 [M+H]^+^ (calcd for C_21_H_19_N_4_O, 343.1553). 

#### 3.2.81. 3-(1-((1*H*-Indol-3-yl)methyl)-1*H*-imidazol-5-yl)-6-methoxy-1*H*-indole (**80**) 

Using the general procedure, reaction of 6-methoxy-1*H*-indole-3-carbaldehyde (0.026 g, 0.146 mmol) was reacted with (1*H*-indol-3-yl)methanamine (0.021 g, 0.146 mmol), K_2_CO_3_ (0.020 g, 0.146 mmol) and *p*-toluenesulfonylmethyl isocyanide (0.029 g, 0.146 mmol) followed by purification using silica gel column chromatography (EtOAc) afforded the title compound as a brown solid (0.018 g, 36%). R*_f =_* 0.43 (CH_2_Cl_2_:MeOH, 9:1); m.p. 95–96 °C; IR (ATR) ν_max_ 3399, 3214, 2921, 1457, 744 cm^−1^; ^1^H NMR (400 MHz, CDCl_3_) δ 8.41 (2H, br s, NH-8, NH-4′), 7.60 (1H, d, *J* = 1.0 Hz, H-2), 7.53 (1H, d, *J* = 8.6 Hz, H-12), 7.38 (1H, d, *J* = 8.0 Hz, H-8′), 7.35 (1H, d, *J* = 8.0 Hz, H-5′), 7.23 (1H, d, *J* = 1.0 Hz, H-4), 7.20 (1H, ddd, *J* = 8.0, 7.5, 1.0 Hz, H-6′), 7.08 (1H, ddd, *J* = 8.0, 7.5, 1.0 Hz, H-7′), 7.02 (1H, d, *J* = 2.5 Hz, H-7), 6.89 (1H, d, *J* = 2.5 Hz, H-9), 6.85 (1H, dd, *J* = 8.6, 2.5 Hz, H-11), 6.84 (1H, d, *J* = 2.4 Hz, H-3′), 5.27 (2H, s, H_2_-1′), 3.85 (3H, s, OMe); ^13^C NMR (100 MHz, CDCl_3_) δ 157.1 (C-10), 137.9 (C-2), 136.9 (C-8a), 136.5 (C-4a′), 128.2 (C-4), 126.6 (C-5), 126.0 (C-8a′), 123.2 (C-3′), 122.8 (C-6′), 122.3 (C-7), 121.6 (C-12a), 120.5 (C-12), 120.2 (C-7′), 118.5 (C-8′), 112.1 (C-2′), 111.6 (C-5′), 110.7 (C-11), 105.6 (C-6), 94.9 (C-9), 55.9 (OMe), 41.3 (C-1′); (+)-HRESIMS *m/z* 343.1556 [M+H]^+^ (calcd for C_21_H_19_N_4_O, 343.1553). 

#### 3.2.82. 3-(1-(2-(1*H*-Indol-3-yl)ethyl)-1*H*-imidazol-5-yl)-4-fluoro-1*H*-indole (**81**) 

Using the general procedure, reaction of 4-fluoro-1*H*-indole-3-carbaldehyde (0.024 g, 0.145 mmol) with tryptamine (0.023 g, 0.145 mmol), K_2_CO_3_ (0.020 g, 0.145 mmol) and *p*-toluenesulfonylmethyl isocyanide (0.028 g, 0.145 mmol) followed by purification using silica gel column chromatography (CH_2_Cl_2_:MeOH, 1:0→9:1) afforded the title compound as a yellow solid (0.036 g, 72%). R*_f_ =* 0.27 (CH_2_Cl_2_:MeOH, 9:1); m.p. > 230 °C; IR (ATR) ν_max_ 3409, 3138, 2927, 1227, 738 cm^−1^; ^1^H NMR (400 MHz, DMSO-*d*_6_) δ 11.76 (1H, br s, NH-8), 10.78 (1H, br s, NH-5′), 7.68 (1H, br s, H-2), 7.47 (1H, d, *J* = 2.5 Hz, H-7), 7.34 (1H, d, *J* = 8.0 Hz, H-9), 7.27 (1H, d, *J* = 8.2 Hz, H-6′), 7.14 (1H, ddd, *J* = 8.0, 8.0, 5.2 Hz, H-10), 6.99 (1H, ddd, *J* = 8.2, 6.9, 0.9 Hz, H-7′), 6.94 (1H, d, *J* = 2.2 Hz, H-4′), 6.91 (1H, br s, H-4), 6.84 (1H, d, *J* = 7.9 Hz, H-9′), 6.79 (1H, dd, *J* = 11.7, 8.0 Hz, H-11), 6.76 (1H, ddd, *J* = 7.9, 6.9, 0.9 Hz, H-8′), 4.12 (2H, t, *J* = 7.8 Hz, H_2_-1′), 2.90 (2H, t, *J* = 7.8 Hz, H_2_-2′); ^13^C NMR (100 MHz, DMSO-*d*_6_) δ 155.7 (d, ^1^*J*_CF_ = 243.9 Hz, C-12), 139.1 (d, ^3^*J*_CF_ = 10.8 Hz, C-8a), 137.5 (C-2), 136.0 (C-5a′), 128.3 (C-4), 126.7 (C-9a′), 126.4 (C-7), 125.5 (C-5), 122.9 (C-4′), 122.2 (d, ^3^*J*_CF_ = 7.7 Hz, C-10), 120.9 (C-7′), 118.2 (C-8′), 117.7 (C-9′), 115.4 (d, ^2^*J*_CF_ = 18.1 Hz, C-12a), 111.3 (C-6′), 110.3 (C-3′), 108.5 (d, ^4^*J*_CF_ = 2.9 Hz, C-9), 104.5 (d, ^2^*J*_CF_ = 19.1 Hz, C-11), 101.1 (C-6), 45.2 (C-1′), 26.5 (C-2′); (–)-HRESIMS *m/z* 343.1359 [M−H]^−^ (calcd for C_21_H_16_FN_4_, 343.1364). 

#### 3.2.83. 3-(1-(2-(1*H*-Indol-3-yl)ethyl)-1*H*-imidazol-5-yl)-7-fluoro-1*H*-indole (**82**) 

Using the general procedure, reaction of 4-fluoro-1*H*-indole-3-carbaldehyde (0.024 g, 0.145 mmol) with tryptamine (0.023 g, 0.145 mmol), K_2_CO_3_ (0.020 g, 0.145 mmol) and *p*-toluenesulfonylmethyl isocyanide (0.028 g, 0.145 mmol) followed by purification using silica gel column chromatography (CH_2_Cl_2_:MeOH, 1:0→9:1) afforded the title compound as an off-white solid (0.020 g, 40%). R*_f_* = 0.30 (CH_2_Cl_2_:MeOH, 9:1); m.p. > 230 °C; IR (ATR) ν_max_ 3417, 3135, 2927, 2856, 1234, 739 cm^−1^; ^1^H NMR (400 MHz, DMSO-*d*_6_) δ 11.97 (1H, br s, NH-8), 10.80 (1H, br s, NH-5′), 7.71 (1H, d, *J* = 1.0 Hz, H-2), 7.58 (1H, d, *J* = 2.5 Hz, H-7), 7.32–7.29 (1H, m, H-12), 7.29 (1H, d, *J* = 8.0 Hz, H-6′), 7.06–7.00 (4H, m, H-10, H-11, H-7′, H-9′), 7.02 (1H, d, *J* = 1.0 Hz, H-4), 6.96 (1H, d, *J* = 2.4 Hz, H-4′), 6.84 (1H, ddd, *J* = 8.0, 7.0, 1.0 Hz, H-8′), 4.22 (2H, t, *J* = 7.9 Hz, H_2_-1′), 2.93 (2H, t, *J* = 7.9 Hz, H_2_-2′); ^13^C NMR (100 MHz, DMSO-*d*_6_) δ 149.3 (d, ^1^*J*_CF_ = 243.9 Hz, C-9), 137.9 (C-2), 136.0 (C-5a′), 130.6 (d, ^3^*J*_CF_ = 5.9 Hz, C-12a), 127.7 (C-4), 126.8 (C-9a′), 125.6 (C-7), 125.0 (C-5), 124.0 (d, ^2^*J*_CF_ = 13.8 Hz, C-8a), 122.9 (C-4′), 120.9 (C-7′), 120.0 (d, ^3^*J*_CF_ = 6.2 Hz, C-11), 118.2 (C-8′), 117.9 (C-9′), 115.1 (d, ^4^*J*_CF_ = 2.9 Hz, C-12), 111.3 (C-6′), 110.3 (C-3′), 106.5 (d, ^2^*J*_CF_ = 15.9 Hz, C-10), 105.0 (d, ^4^*J*_CF_ = 2.4 Hz, C-6), 45.4 (C-1′), 26.6 (C-2′); (–)-HRESIMS *m/z* 343.1350 [M−H]^−^ (calcd for C_21_H_16_FN_4_, 343.1364). 

#### 3.2.84. 3-(1-(2-(1*H*-Indol-3-yl)ethyl)-1*H*-imidazol-5-yl)-4-chloro-1*H*-indole (**83**) 

Using the general procedure, reaction of 4-chloro-1*H*-indole-3-carbaldehyde (0.025 g, 0.139 mmol) with tryptamine (0.023 g, 0.139 mmol), K_2_CO_3_ (0.019 g, 0.139 mmol) and *p*-toluenesulfonylmethyl isocyanide (0.027 g, 0.139 mmol) followed by purification using silica gel column chromatography (CH_2_Cl_2_:MeOH, 1:0→9:1) afforded the title compound as a brown solid (0.022 g, 44%). R*_f_ =* 0.33 (CH_2_Cl_2_:MeOH, 9:1); m.p. > 230 °C; IR (ATR) ν_max_ 3399, 3173, 2924, 1490, 1194, 741 cm^−1^; ^1^H NMR (400 MHz, DMSO-*d*_6_) δ 11.81 (1H, d, *J* = 2.2 Hz, NH-8), 10.75 (1H, br s, NH-5′), 7.71 (1H, d, *J* = 1.0 Hz, H-2), 7.51 (1H, d, *J* = 2.2 Hz, H-7), 7.50 (1H, d, *J* = 7.9 Hz, H-9), 7.26 (1H, d, *J* = 8.2 Hz, H-6′), 7.15 (1H, dd, *J* = 7.9, 7.9 Hz, H-10), 7.05 (1H, d, *J* = 7.9 Hz, H-11), 6.97 (1H, dd, *J* = 8.2, 7.9 Hz, H-7′), 6.94 (1H, br s, H-4′), 6.88 (1H, d, *J* = 1.0 Hz, H-4), 6.69 (1H, dd, *J* = 7.9, 7.9 Hz, H-8′), 6.63 (1H, d, *J* = 7.9 Hz, H-9′), 3.99 (2H, t, *J* = 7.9 Hz, H_2_-1′), 2.89 (2H, t, *J* = 7.9 Hz, H_2_-2′); ^13^C NMR (100 MHz, DMSO-*d*_6_) δ 137.6 (C-8a), 137.2 (C-2), 136.0 (C-5a′), 129.2 (C-4), 128.4 (C-7), 126.6 (C-9a′), 125.0 (C-5), 124.2 (C-12/C-12a), 124.1 (C-12/C-12a), 122.9 (C-10), 122.4 (C-4′), 120.8 (C-7′), 120.2 (C-11), 118.1 (C-8′), 117.5 (C-9′), 111.28 (C-9/C-6′), 111.25 (C-9/C-6′), 110.2 (C-3′), 102.6 (C-6), 45.3 (C-1′), 26.6 (C-2′); (–)-HRESIMS *m/z* 359.1068 [M−H]^−^ (calcd for C_21_H_16_^35^ClN_4_, 359.1069), *m/z* 361.1046 [M−H]^−^ (calcd for C_21_H_16_^37^ClN_4_, 361.1047). 

#### 3.2.85. 3-(1-(2-(1*H*-Indol-3-yl)ethyl)-1*H*-imidazol-5-yl)-5-chloro-1*H*-indole (**84**) 

Using the general procedure, reaction of 5-chloro-1*H*-indole-3-carbaldehyde (0.025 g, 0.139 mmol) with tryptamine (0.023 g, 0.139 mmol), K_2_CO_3_ (0.019 g, 0.139 mmol) and *p*-toluenesulfonylmethyl isocyanide (0.027 g, 0.139 mmol) followed by purification using silica gel column chromatography (EtOAc) afforded the title compound as a yellow solid (0.028 g, 56%). R*_f_ =* 0.44 (CH_2_Cl_2_:MeOH, 9:1); m.p. 70–71 °C; IR (ATR) ν_max_ 3401, 3172, 2922, 1457, 744 cm^−1^; ^1^H NMR (400 MHz, DMSO-*d*_6_) δ 11.66 (1H, br s, NH-8), 10.80 (1H, br s, NH-5′), 7.70 (1H, d, *J* = 1.0 Hz, H-2), 7.60 (1H, d, *J* = 2.0 Hz, H-7), 7.50 (1H, d, *J* = 8.3 Hz, H-9), 7.45 (1H, d, *J* = 2.0 Hz, H-12), 7.29 (1H, d, *J* = 8.3 Hz, H-6′), 7.18 (1H, dd, *J* = 8.3, 2.0 Hz, H-10), 7.05 (1H, d, *J* = 7.9 Hz, H-9′), 7.04–6.99 (1H, m, H-7′), 7.01 (1H, d, *J* = 1.0 Hz, H-4), 6.97 (1H, d, *J* = 2.5 Hz, H-4′), 6.84 (1H, ddd, *J* = 7.9, 6.9, 1.0 Hz, H-8′), 4.21 (2H, t, *J* = 7.6 Hz, H_2_-1′), 2.93 (2H, t, *J* = 7.6 Hz, H_2_-2′); ^13^C NMR (100 MHz, DMSO-*d*_6_) δ 137.9 (C-2), 136.0 (C-5a′), 134.5 (C-8a), 127.8 (C-12a), 127.6 (C-4), 126.8 (C-9a′), 126.2 (C-7), 124.9 (C-5), 124.3 (C-11), 122.9 (C-4′), 121.7 (C-10), 120.9 (C-7′), 118.2 (C-8′), 117.9 (C-12, C-9′), 113.4 (C-9), 111.3 (C-6′), 110.2 (C-3′), 103.7 (C-6), 45.3 (C-1′), 26.6 (C-2′); (+)-HRESIMS *m/z* 361.1206 [M+H]^+^ (calcd for C_21_H_18_^35^ClN_4_, 361.1215), *m/z* 363.1168 [M+H]^+^ (calcd for C_21_H_18_^37^ClN_4_, 363.1192). 

#### 3.2.86. 3-(1-(2-(1*H*-Indol-3-yl)ethyl)-1*H*-imidazol-5-yl)-7-chloro-1*H*-indole (**85**)

Using the general procedure, reaction of 7-chloro-1*H*-indole-3-carbaldehyde (0.025 g, 0.139 mmol) with tryptamine (0.023 g, 0.139 mmol), K_2_CO_3_ (0.019 g, 0.139 mmol) and *p*-toluenesulfonylmethyl isocyanide (0.027 g, 0.139 mmol) followed by purification using silica gel column chromatography (CH_2_Cl_2_:MeOH, 1:0→9:1) afforded the title compound as an off-white solid (0.023 g, 46%). R*_f_ =* 0.31 (CH_2_Cl_2_:MeOH, 9:1); m.p. 226–227 °C; IR (ATR) ν_max_ 3408, 3130, 2919, 1435, 1110, 740 cm^−1^; ^1^H NMR (400 MHz, DMSO-*d*_6_) δ 11.85 (1H, br s, NH-8), 10.80 (1H, br s, NH-5′), 7.73 (1H, d, *J* = 1.0 Hz, H-2), 7.60 (1H, d, *J* = 2.4 Hz, H-7), 7.46 (1H, d, *J* = 7.9 Hz, H-12), 7.29 (1H, d, *J* = 8.0 Hz, H-6′), 7.26 (1H, dd, *J* = 7.9, 0.9 Hz, H-10), 7.07 (1H, dd, *J* = 7.9, 7.9 Hz, H-11), 7.04–7.01 (1H, m, H-7′), 7.03 (1H, d, *J* = 1.0 Hz, H-4), 7.00 (1H, d, *J* = 7.8 Hz, H-9′), 6.97 (1H, d, *J* = 2.5 Hz, H-4′), 6.84 (1H, ddd, *J* = 7.8, 6.9, 1.0 Hz, H-8′), 4.22 (2H, t, *J* = 7.5 Hz, H_2_-1′), 2.93 (2H, t, *J* = 7.5 Hz, H_2_-2′); ^13^C NMR (100 MHz, DMSO-*d*_6_) δ 137.9 (C-2), 136.0 (C-5a′), 132.9 (C-8a), 128.6 (C-12a), 127.8 (C-4), 126.7 (C-9a′), 125.7 (C-7), 124.9 (C-5), 122.9 (C-4′), 121.3 (C-10), 120.9 (C-7′), 120.6 (C-11), 118.2 (C-8′), 118.0 (C-12/C-9′), 117.9 (C-12/C-9′), 116.2 (C-9), 111.3 (C-6′), 110.2 (C-3′), 105.2 (C-6), 45.4 (C-1′), 26.6 (C-2′); (–)-HRESIMS *m/z* 359.1066 [M−H]^−^ (calcd for C_21_H_16_^35^ClN_4_, 359.1069), *m/z* 361.1049 [M−H]^−^ (calcd for C_21_H_16_^37^ClN_4_, 361.1047). 

#### 3.2.87. 3-(1-(2-(1*H*-Indol-3-yl)ethyl)-1*H*-imidazol-5-yl)-4-bromo-1*H*-indole (**86**) 

Using the general procedure, reaction of 4-bromo-1*H*-indole-3-carbaldehyde (0.028 g, 0.123 mmol) with tryptamine (0.020 g, 0.123 mmol), K_2_CO_3_ (0.017 g, 0.123 mmol) and *p*-toluenesulfonylmethyl isocyanide (0.024 g, 0.123 mmol) followed by purification using silica gel column chromatography (CH_2_Cl_2_:MeOH, 1:0→9:1) afforded the title compound as an off white solid (0.021 g, 42%). R*_f_ =* 0.34 (CH_2_Cl_2_:MeOH, 9:1); m.p. > 230 °C; IR (ATR) ν_max_ 3422, 3111, 2919, 1456, 1192, 742 cm^−1^; ^1^H NMR (400 MHz, DMSO-*d*_6_) δ 11.82 (1H, d, *J* = 2.5 Hz, NH-8), 10.75 (1H, br s, NH-5′), 7.73 (1H, br s, H-2), 7.55 (1H, dd, *J* = 7.8, 1.0 Hz, H-9), 7.52 (1H, d, *J* = 2.5 Hz, H-7), 7.25 (1H, d, *J* = 7.9 Hz, H-6′), 7.22 (1H, dd, *J* = 7.8, 1.0 Hz, H-11), 7.08 (1H, dd, *J* = 7.8, 7.8 Hz, H-10), 6.99–6.94 (2H, m, H-4′, H-7′), 6.88 (1H, d, *J* = 0.9 Hz, H-4), 6.69 (1H, ddd, *J* = 7.8, 6.9, 1.0 Hz, H-8′), 6.61 (1H, d, *J* = 7.8 Hz, H-9′), 3.95 (2H, t, *J* = 7.8 Hz, H_2_-1′), 2.90 (2H, t, *J* = 7.8 Hz, H_2_-2′); ^13^C NMR (100 MHz, DMSO-*d*_6_) δ 137.3 (C-8a), 137.1 (C-2), 136.0 (C-5a′), 129.6 (C-4), 128.7 (C-7), 126.6 (C-9a′), 125.5 (C-12a), 124.5 (C-5), 123.5 (C-11), 122.9 (C-10/C-4′), 122.7 (C-10/C-4′), 120.8 (C-7′), 118.1 (C-8′), 117.5 (C-9′), 112.5 (C-12), 111.7 (C-9), 111.3 (C-6′), 110.3 (C-3′), 103.4 (C-6), 45.5 (C-1′), 26.7 (C-2′); (–)-HRESIMS *m/z* 403.0565 [M−H]^−^ (calcd for C_21_H_16_^79^BrN_4_, 403.0564), *m/z* 405.0545 [M−H]^−^ (calcd for C_21_H_16_^81^BrN_4_, 405.0546). 

#### 3.2.88. 3-(1-(2-(1*H*-Indol-3-yl)ethyl)-1*H*-imidazol-5-yl)-5-methoxy-1*H*-indole (**87**) 

Using the general procedure, reaction of 5-methoxy-1*H*-indole-3-carbaldehyde (0.049 g, 0.280 mmol) with tryptamine (0.045 g, 0.280 mmol), K_2_CO_3_ (0.039 g, 0.280 mmol) and *p*-toluenesulfonylmethyl isocyanide (0.055 g, 0.280 mmol) followed by purification using silica gel column chromatography (CH_2_Cl_2_:MeOH, 1:0→9:1) afforded the title compound as an orange solid (0.095 g, 95%). R*_f_* = 0.36 (CH_2_Cl_2_:MeOH, 9:1); m.p. 152–153 °C; IR (ATR) ν_max_ 3411, 3115, 2929, 1481, 1211 cm^−1^; ^1^H NMR (400 MHz, CDCl_3_) δ 8.47 (1H, br s, NH-8), 8.10 (1H, br s, NH-5′), 7.48 (1H, d, *J* = 0.9 Hz, H-2), 7.32 (1H, d, *J* = 8.9 Hz, H-9), 7.30 (1H, dd, *J* = 7.9, 1.0 Hz, H-6′), 7.17–7.12 (3H, m, H-4, H-7′, H-9′), 7.03 (1H, d, *J* = 2.5 Hz, H-7), 7.02–7.00 (1H, m, H-8′), 7.00–6.98 (1H, m, H-12), 6.93 (1H, dd, *J* = 8.9, 2.5 Hz, H-10), 6.71 (1H, d, *J* = 2.5 Hz, H-4′), 4.21 (2H, t, *J* = 7.2 Hz, H_2_-1′), 3.79 (3H, s, OMe), 3.03 (2H, t, *J* = 7.2 Hz, H_2_-2′); ^13^C NMR (100 MHz, CDCl_3_) δ 155.1 (C-11), 137.9 (C-2), 136.3 (C-5a′), 131.2 (C-8a), 128.8 (C-4), 128.0 (C-12a), 127.0 (C-9a′), 126.0 (C-5), 124.5 (C-7), 122.4 (C-4′/C-7′), 122.3 (C-4′/C-7′), 119.6 (C-8′), 118.3 (C-9′), 113.5 (C-10), 112.3 (C-9), 111.7 (C-3′), 111.4 (C-6′), 105.3 (C-6), 101.0 (C-12), 56.0 (OMe), 45.9 (C-1′), 27.5 (C-2′); (–)-HRESIMS *m/z* 355.1575 [M−H]^−^ (calcd for C_22_H_19_N_4_O, 355.1564).

#### 3.2.89. 3-(1-(2-(1*H*-Indol-3-yl)ethyl)-1*H*-imidazol-5-yl)-6-methoxy-1*H*-indole (**88**) 

Using the general procedure, reaction of 6-methoxy-1*H*-indole-3-carbaldehyde (0.025 g, 0.140 mmol) with tryptamine (0.023 g, 0.140 mmol), K_2_CO_3_ (0.020 g, 0.140 mmol) and *p*-toluenesulfonylmethyl isocyanide (0.028 g, 0.140 mmol) followed by purification using silica gel column chromatography (CH_2_Cl_2_:MeOH, 1:0→9:1) afforded the title compound as brown solid (0.011 g, 22%). R*_f_ =* 0.43 (CH_2_Cl_2_:MeOH), 9:1); m.p. 81–82 °C; IR (ATR) ν_max_ 3301, 2932, 2841, 1456, 1161, 738 cm^−1^; ^1^H NMR (400 MHz, CDCl_3_) δ 8.54 (1H, br s, NH-8), 8.19 (1H, br s, NH-5′), 7.43 (1H, d, *J* = 8.8 Hz, H-12), 7.42 (1H, d, *J* = 1.5 Hz, H-2), 7.29 (1H, dd, *J* = 7.8, 1.0 Hz, H-6′), 7.19–7.13 (2H, m, H-7′, H-9′), 7.13 (1H, d, *J* = 1.5 Hz, H-4), 7.01 (1H, ddd, *J* = 7.8, 7.0, 1.0 Hz, H-8′), 6.96 (1H, d, *J* = 2.5 Hz, H-7), 6.91 (1H, d, *J* = 2.0 Hz, H-9), 6.83 (1H, dd, *J* = 8.8, 2.0 Hz, H-11), 6.67 (1H, d, *J* = 2.5 Hz, H-4′), 4.20 (2H, t, *J* = 7.2 Hz, H_2_-1′), 3.86 (3H, s, OMe), 3.00 (2H, t, *J* = 7.2 Hz, H_2_-2′); ^13^C NMR (100 MHz, CDCl_3_) δ 157.2 (C-10), 137.9 (C-2), 136.9 (C-8a), 136.3 (C-5a′), 128.8 (C-4), 127.0 (C-9a′), 126.0 (C-5), 122.5 (C-4′/C-7′), 122.4 (C-4′/C-7′), 122.33 (C-12a), 122.27 (C-7), 120.4 (C-12), 119.6 (C-8′), 118.4 (C-9′), 111.8 (C-3′), 111.4 (C-6′), 110.7 (C-11), 105.7 (C-6), 94.5 (C-9), 55.9 (OMe), 45.9 (C-1′), 27.4 (C-2′); (–)-HRESIMS *m/z* 355.1570 [M−H]^−^ (calcd for C_22_H_19_N_4_O, 355.1564). 

#### 3.2.90. 4-Fluoro-3-(1-pentyl-1*H*-imidazol-5-yl)-1*H*-indole (**89**) 

Using the general procedure, reaction of 4-fluoro-1*H*-indole-3-carbaldehyde (0.030 g, 0.184 mmol) with pentylamine (21 µL, 0.184 mmol), K_2_CO_3_ (0.026 g, 0.184 mmol) and *p*-toluenesulfonylmethyl isocyanide (0.036 g, 0.184 mmol) followed by purification using silica gel column chromatography (CH_2_Cl_2_:MeOH, 1:0→9:1) afforded the title compound as a brown solid (0.043 g, 86%). R*_f_ =* 0.44 (CH_2_Cl_2_:MeOH, 9:1); m.p. 147–148 °C; IR (ATR) ν_max_ 3118, 2955, 2930, 2859, 1228, 734 cm^−1^; ^1^H NMR (400 MHz, CDCl_3_) δ 9.32 (1H, br s, NH-8), 7.63 (1H, br s, H-2), 7.24 (1H, d, *J* = 7.9 Hz, H-9), 7.20 (1H, d, *J* = 2.5 Hz, H-7), 7.15 (1H, ddd, *J* = 7.9, 7.9, 5.0 Hz, H-10), 7.07 (1H, br s, H-4), 6.80 (1H, ddd, *J* = 11.1, 7.9, 1.0 Hz, H-11), 3.90 (2H, t, *J* = 7.2 Hz, H_2_-1′), 1.61 (2H, p, *J* = 7.2 Hz, H_2_-2′), 1.22–1.12 (4H, m, H_2_-3′, H_2_-4′), 0.78 (3H, t, *J* = 6.9 Hz, H_3_-5′); ^13^C NMR (100 MHz, CDCl_3_) δ 156.6 (d, ^1^*J*_CF_ = 248.0 Hz, C-4), 139.1 (d, ^3^*J*_CF_ = 10.7 Hz, C-8a), 137.5 (C-2), 128.9 (C-4), 126.3 (C-5), 125.2 (C-7), 123.2 (d, ^3^*J*_CF_ = 7.9 Hz, C-10), 116.3 (d, ^2^*J*_CF_ = 19.1 Hz, C-12a), 107.9 (d, ^4^*J*_CF_ = 3.7 Hz, C-9), 105.7 (d, ^2^*J*_CF_ = 19.4 Hz, C-11), 103.1 (C-6), 45.3 (C-1′), 30.4 (C-2′), 28.7 (C-3′), 22.2 (C-4′), 13.9 (C-5′); (–)-HRESIMS *m/z* 270.1406 [M−H]^−^ (calcd for C_16_H_17_FN_3_, 270.1412). 

#### 3.2.91. 7-Fluoro-3-(1-pentyl-1*H*-imidazol-5-yl)-1*H*-indole (**90**) 

Using the general procedure, reaction of 7-fluoro-1*H*-indole-3-carbaldehyde (0.030 g, 0.184 mmol) with pentylamine (21 µL, 0.184 mmol), K_2_CO_3_ (0.026 g, 0.184 mmol) and *p*-toluenesulfonylmethyl isocyanide (0.036 g, 0.184 mmol) followed by purification using silica gel column chromatography (CH_2_Cl_2_:MeOH, 1:0→9:1) afforded the title compound as a brown solid (0.030 g, 60%). R*_f_ =* 0.44 (CH_2_Cl_2_:MeOH, 9:1); m.p. 164–165 °C; IR (ATR) ν_max_ 3072, 2957, 2930, 2859, 1234, 1110, 783 cm^−1^; ^1^H NMR (400 MHz, CDCl_3_) δ 9.48 (1H, br s, NH-8), 7.68 (1H, d, *J* = 1.0 Hz, H-2), 7.30 (1H, d, *J* = 7.8 Hz, H-12), 7.29 (1H, d, *J* = 2.4 Hz, H-7), 7.16 (1H, d, *J* = 1.0 Hz, H-4), 7.07 (1H, ddd, *J* = 7.8, 7.8, 4.9 Hz, H-11), 6.97 (1H, ddd, *J* = 11.4, 7.8, 1.0 Hz, H-10), 3.91 (2H, t, *J* = 7.4 Hz, H_2_-1′), 1.63 (2H, p, *J* = 7.4 Hz, H_2_-2′), 1.23–1.13 (4H, m, H_2_-3′, H_2_-4′), 0.79 (3H, t, *J* = 6.8 Hz, H_3_-5′); ^13^C NMR (100 MHz, CDCl_3_) δ 149.8 (d, ^1^*J*_CF_ = 244.6 Hz, C-9), 137.6 (C-2), 131.1 (d, ^3^*J*_CF_ = 5.2 Hz, C-12a), 128.7 (C-4), 125.6 (C-5), 124.7 (d, ^2^*J*_CF_ = 14.0 Hz, C-8a), 124.6 (C-7), 120.8 (d, ^3^*J*_CF_ = 6.2 Hz, C-11), 115.4 (d, ^4^*J*_CF_ = 3.4 Hz, C-12), 107.6 (d, ^2^*J*_CF_ = 16.1 Hz, C-10), 106.3 (d, ^4^*J*_CF_ = 3.0 Hz, C-6), 45.5 (C-1′), 30.7 (C-2′), 28.7 (C-3′), 22.2 (C-4′), 13.9 (C-5′); (–)-HRESIMS *m/z* 270.1414 [M−H]^−^ (calcd for C_16_H_17_FN_3_, 270.1412). 

#### 3.2.92. 4-Chloro-3-(1-pentyl-1*H*-imidazol-5-yl)-1*H*-indole (**91**) 

Using the general procedure, reaction of 4-chloro-1*H*-indole-3-carbaldehyde (0.031 g, 0.174 mmol) with pentylamine (20 µL, 0.174 mmol), K_2_CO_3_ (0.024 g, 0.174 mmol) and *p*-toluenesulfonylmethyl isocyanide (0.034 g, 0.174 mmol) followed by purification by trituration with CH_2_Cl_2_ afforded the title compound as a yellow solid (0.019 g, 38%). R*_f_* = 0.43 (CH_2_Cl_2_:MeOH, 9:1); m.p. 188–189 ℃; IR (ATR) ν_max_ 3107, 2960, 2930, 2861, 1491, 1191, 739 cm^−1^; ^1^H NMR (400 MHz, CDCl_3_) δ 8.98 (1H, br s, NH-8), 7.63 (1H, br s, H-2), 7.36 (1H, dd, *J* = 7.8, 1.5 Hz, H-9), 7.26 (1H, d, *J* = 2.4 Hz, H-7), 7.16 (1H, dd, *J* = 7.8, 7.5 Hz, H-10), 7.12 (1H, dd, *J* = 7.5, 1.5 Hz, H-11), 7.04 (1H, br s, H-4), 3.80 (2H, t, *J* = 7.4 Hz, H_2_-1′), 1.58 (2H, p, *J* = 7.4 Hz, H_2_-2′), 1.22–1.12 (4H, m, H_2_-3′, H_2_-4′), 0.78 (3H, t, *J* = 7.0 Hz, H_3_-5′); ^13^C NMR (100 MHz, CDCl_3_) δ 137.4 (C-8a), 137.2 (C-2), 130.0 (C-4), 126.9 (C-7), 126.3 (C-12), 125.5 (C-5), 124.9 (C-12a), 123.3 (C-10), 121.4 (C-11), 110.5 (C-9), 104.8 (C-6), 45.5 (C-1′), 30.3 (C-2′), 28.8 (C-3′), 22.2 (C-4′), 13.9 (C-5′); (–)-HRESIMS *m/z* 286.1114 [M−H]^−^ (calcd for C_16_H_17_^35^ClN_3_, 286.1116), *m/z* 288.1095 [M−H]^−^ (calcd for C_16_H_17_^37^ClN_3_, 288.1091). 

#### 3.2.93. 5-Chloro-3-(1-pentyl-1*H*-imidazol-5-yl)-1*H*-indole (**92**) 

Using the general procedure, reaction of 5-chloro-1*H*-indole-3-carbaldehyde (0.031 g, 0.174 mmol) with pentylamine (20 µL, 0.174 mmol), K_2_CO_3_ (0.024 g, 0.174 mmol) and *p*-toluenesulfonylmethyl isocyanide (0.034 g, 0.17 mmol) followed by purification using silica gel column chromatography (EtOAc) afforded the title compound as a brown gum (0.026 g, 52%). R*_f_* = 0.45 (CH_2_Cl_2_:MeOH, 9:1); IR (ATR) ν_max_ 3129, 2929, 2873, 1458, 1109, 799 cm^−1^; ^1^H NMR (400 MHz, CDCl_3_) δ 9.84 (1H, br s, NH-8), 7.66 (1H, br s, H-2), 7.51 (1H, d, *J* = 2.0 Hz, H-12), 7.37 (1H, d, *J* = 8.5 Hz, H-9), 7.29 (1H, d, *J* = 2.4 Hz, H-7), 7.20 (1H, dd, *J* = 8.5, 2.0 Hz, H-10), 7.14 (1H, br s, H-4), 3.91 (2H, t, *J* = 7.3 Hz, H_2_-1′), 1.63 (2H, p, *J* = 7.3 Hz, H_2_-2′), 1.23–1.13 (4H, m, H_2_-3′, H_2_-4′), 0.80 (3H, t, *J* = 6.8 Hz, H_3_-5′); ^13^C NMR (100 MHz, CDCl_3_) δ 137.6 (C-2), 134.7 (C-8a), 128.7 (C-4), 128.6 (C-12a), 126.3 (C-11), 125.7 (C-5), 125.4 (C-7), 123.1 (C-10), 119.0 (C-12), 112.8 (C-9), 105.0 (C-6), 45.5 (C-1′), 30.7 (C-2′), 28.7 (C-3′), 22.2 (C-4′), 13.9 (C-5′); (+)-HRESIMS *m/z* 288.1261 [M+H]^+^ (calcd for C_16_H_19_^35^ClN_3_, 288.1262), *m/z* 290.1227 [M+H]^+^ (calcd for C_16_H_19_^37^ClN_3_, 290.1237). 

#### 3.2.94. 7-Chloro-3-(1-pentyl-1*H*-imidazol-5-yl)-1*H*-indole (**93**) 

Using the general procedure, reaction of 7-chloro-1*H*-indole-3-carbaldehyde (0.031 g, 0.174 mmol) with pentylamine (20 µL, 0.174 mmol), K_2_CO_3_ (0.024 g, 0.174 mmol) and *p*-toluenesulfonylmethyl isocyanide (0.034 g, 0.174 mmol) followed by purification using silica gel column chromatography (CH_2_Cl_2_:MeOH, 1:0→9:1) afforded the title compound as a brown solid (0.041 g, 82%). R*_f_* = 0.36 (CH_2_Cl_2_:MeOH, 9:1); m.p. 122–123 °C; IR (ATR) ν_max_ 3097, 2956, 2929, 2858, 1436, 1110, 737 cm^−1^; ^1^H NMR (400 MHz, CDCl_3_) δ 8.96 (1H, br s, NH-8), 7.64 (1H, d, *J* = 1.0 Hz, H-2), 7.45 (1H, dd, *J* = 8.0, 1.0 Hz, H-12), 7.31 (1H, d, *J* = 2.5 Hz, H-7), 7.27 (1H, dd, *J* = 8.0, 7.5 Hz, H-10), 7.14 (1H, d, *J* = 1.0 Hz, H-4), 7.11 (1H, dd, *J* = 7.5, 1.0 Hz, H-11), 3.90 (2H, t, *J* = 7.2 Hz, H_2_-1′), 1.63 (2H, p, *J* = 7.2 Hz, H_2_-2′), 1.23–1.13 (4H, m, H_2_-3′, H_2_-4′), 0.79 (3H, t, *J* = 7.0 Hz, H_3_-5′); ^13^C NMR (100 MHz, CDCl_3_) δ 137.8 (C-2), 133.5 (C-8a), 129.1 (C-4), 128.9 (C-12a), 125.4 (C-5), 124.3 (C-7), 122.3 (C-10), 121.4 (C-11), 118.4 (C-12), 117.1 (C-9), 106.8 (C-6), 45.4 (C-1′), 30.7 (C-2′), 28.7 (C-3′), 22.2 (C-4′), 13.9 (C-5′); (–)-HRESIMS *m/z* 286.1112 [M−H]^−^ (calcd for C_16_H_17_^35^ClN_3_, 286.1116), *m/z* 288.1091 [M−H]^−^ (calcd for C_16_H_17_^37^ClN_3_, 288.1091). 

#### 3.2.95. 4-Bromo-3-(1-pentyl-1*H*-imidazol-5-yl)-1*H*-indole (**94**) 

Using the general procedure, reaction of 4-bromo-1*H*-indole-3-carbaldehyde (0.034 g, 0.150 mmol) with pentylamine (17 µL, 0.150 mmol), K_2_CO_3_ (0.021 g, 0.150 mmol) and *p*-toluenesulfonylmethyl isocyanide (0.030 g, 0.150 mmol) followed by purification by trituration with CH_2_Cl_2_ afforded the title compound as a yellow solid (0.026 g, 52%). R*_f_* = 0.46 (CH_2_Cl_2_:MeOH, 9:1); m.p. 207–208 °C; IR (ATR) ν_max_ 3140, 2930, 2861, 1337, 1110, 741 cm^−1^; ^1^H NMR (400 MHz, CDCl_3_) δ 9.11 (1H, br s, NH-8), 7.64 (1H, d, *J* = 1.0 Hz, H-2), 7.42 (1H, dd, *J* = 8.3, 1.0 Hz, H-9), 7.31 (1H, dd, *J* = 7.9, 1.0 Hz, H-11), 7.29 (1H, d, *J* = 2.7 Hz, H-7), 7.08 (1H, dd, *J* = 8.3, 7.9 Hz, H-10), 7.05 (1H, d, *J* = 1.0 Hz, H-4), 3.78 (2H, t, *J* = 7.3 Hz, H_2_-1′), 1.59 (2H, p, *J* = 7.3 Hz, H_2_-2′), 1.22–1.12 (4H, m, H_2_-3′, H_2_-4′), 0.79 (3H, t, *J* = 6.8 Hz, H_3_-5′); ^13^C NMR (100 MHz, CDCl_3_) δ 137.14 (C-8a), 137.08 (C-2), 130.5 (C-4), 127.2 (C-7), 126.3 (C-12a), 125.0 (C-5), 124.8 (C-11), 123.6 (C-10), 114.1 (C-4), 111.0 (C-9), 105.5 (C-6), 45.6 (C-1′), 30.3 (C-2′), 28.9 (C-3′), 22.2 (C-4′), 13.9 (C-5′); (–)-HRESIMS *m/z* 330.0618 [M−H]^–^ (calcd for C_16_H_17_^79^BrN_3_, 330.0611), *m/z* 332.0596 [M−H]^−^ (cacld for C_16_H_17_^81^BrN_3_, 332.0592). 

#### 3.2.96. 5-Methoxy-3-(1-pentyl-1*H*-imidazol-5-yl)-1*H*-indole (**95**) 

Using the general procedure, reaction of 5-methoxy-1*H*-indole-3-carbaldehyde (0.031 g, 0.176 mmol) with pentylamine (20 µL, 0.176 mmol), K_2_CO_3_ (0.025 g, 0.176 mmol) and *p*-toluenesulfonylmethyl isocyanide (0.035 g, 0.176 mmol) followed by purification using silica gel column chromatography (CH_2_Cl_2_:MeOH, 1:0→9:1) afforded the title compound as a brown gum (0.021 g, 42%). R*_f_* = 0.33 (CH_2_Cl_2_:MeOH, 9:1); IR (ATR) ν_max_ 3183, 2957, 2858, 1485, 1212, 803 cm^−1^; ^1^H NMR (400 MHz, CDCl_3_) δ 8.97 (1H, br s, NH-8), 7.65 (1H, br s, H-2), 7.33 (1H, d, *J* = 8.8 Hz, H-9), 7.22 (1H, d, *J* = 2.5 Hz, H-7), 7.15 (1H, br s, H-4), 6.96 (1H, d, *J* = 2.4 Hz, H-12), 6.91 (1H, dd, *J* = 8.8, 2.4 Hz, H-10), 3.90 (2H, t, *J* = 7.3 Hz, H_2_-1′), 3.81 (3H, s, OMe), 1.64 (2H, p, *J* = 7.3 Hz, H_2_-2′), 1.24–1.14 (4H, m, H_2_-3′, H_2_-4′), 0.80 (3H, t, *J* = 6.8 Hz, H_3_-5′); ^13^C NMR (100 MHz, CDCl_3_) δ 154.9 (C-11), 137.4 (C-2), 131.2 (C-8a), 128.5 (C-4), 128.0 (C-12a), 126.3 (C-5), 124.5 (C-7), 113.4 (C-10), 112.4 (C-9), 105.2 (C-6), 100.9 (C-12), 56.0 (OMe), 45.4 (C-1′), 30.8 (C-2′), 28.8 (C-3′), 22.2 (C-4′), 13.9 (C-5′); (–)-HRESIMS *m/z* 282.1601 [M−H]^−^ (calcd for C_17_H_20_N_3_O, 282.1612). 

#### 3.2.97. 6-Methoxy-3-(1-pentyl-1*H*-imidazol-5-yl)-1*H*-indole (**96**) 

Using the general procedure, reaction of 6-methoxy-1*H*-indole-3-carbaldehyde (0.031 g, 0.176 mmol) with pentylamine (20 µL, 0.176 mmol), K_2_CO_3_ (0.025 g, 0.176 mmol) and *p*-toluenesulfonylmethyl isocyanide (0.035 g, 0.176 mmol) followed by purification using silica gel column chromatography (CH_2_Cl_2_:MeOH, 1:0→9:1) afforded the title compound as a brown gum (0.016 g, 32%). R*_f_* = 0.33 (CH_2_Cl_2_:MeOH, 9:1); IR (ATR) ν_max_ 3191, 2930, 2861, 1457, 1163, 805 cm^−1^; ^1^H NMR (400 MHz, CDCl_3_) δ 8.87 (1H, br s, NH-8), 7.62 (1H, d, *J* = 1.0 Hz, H-2), 7.41 (1H, d, *J* = 8.8 Hz, H-12), 7.13 (1H, br s, H-4), 7.12 (1H, d, *J* = 2.4 Hz, H-7), 6.92 (1H, d, *J* = 2.2 Hz, H-9), 6.83 (1H, dd, *J* = 8.8, 2.2 Hz, H-11), 3.91 (2H, t, *J* = 7.4 Hz, H_2_-1′), 3.85 (3H, s, OMe), 1.63 (2H, p, *J* = 7.4 Hz, H_2_-2′), 1.24–1.14 (4H, m, H_2_-3′, H_2_-4′), 0.80 (3H, t, *J* = 6.8 Hz, H_3_-5′); ^13^C NMR (100 MHz, CDCl_3_) δ 157.0 (C-10), 137.4 (C-2), 137.0 (C-8a), 128.4 (C-4), 126.3 (C-5), 122.5 (C-7), 121.8 (C-12a), 120.3 (C-12), 110.6 (C-11), 105.5 (C-6), 94.9 (C-9), 55.8 (OMe), 45.4 (C-1′), 30.7 (C-2′), 28.8 (C-3′), 22.2 (C-4′), 13.9 (C-5′); (–)-HRESIMS *m/z* 282.1603 [M−H]^−^ (calcd for C_17_H_20_N_3_O, 282.1612). 

#### 3.2.98. 3-(1-(Benzo[*d*][1,3]dioxol-5-ylmethyl)-1*H*-imidazol-5-yl)-5-chloro-1*H*-indole (**97**) 

Using the general procedure, reaction of 5-chloro-1*H*-indole-3-carbaldehyde (0.050 g, 0.278 mmol) with piperonylamine (0.035 mL, 0.278 mmol), K_2_CO_3_ (0.038 g, 0.278 mmol) and *p*-toluenesulfonylmethyl isocyanide (0.054 g, 0.278 mmol) in DMF (1 mL) followed by purification using silica gel column chromatography (EtOAc) afforded the title compound as a pale brown solid (0.040 g, 41%). *R_f_* = 0.30 (EtOAc); IR (ATR) ν_max_ 3401, 1664, 1504, 1491, 1447, 1250, 1110, 1025, 1005, 923, 894, 762 cm^−1^; ^1^H NMR (400 MHz, DMSO-*d*_6_) δ 11.56 (1H, br s, NH-8), 7.82 (1H, br s, H-2), 7.44 (1H, d, *J* = 8.6 Hz, H-9), 7.42 (1H, d, *J* = 2.7 Hz, H-7), 7.38 (1H, d, *J* = 2.0 Hz, H-12), 7.14 (1H, dd, *J* = 8.6, 2.1 Hz, H-10), 7.09 (1H, br s, H-4), 6.77 (1H, d, *J* = 8.0 Hz, H-7′), 6.52 (1H, d, *J* = 1.6 Hz, H-3′), 6.40 (1H, dd, *J* = 8.1, 1.5 Hz, H-8′), 5.94 (2H, s, H_2_-5′), 5.13 (2H, s, H_2_-1′); ^13^C NMR (100 MHz, DMSO-*d*_6_) δ 147.4 (C-3a′), 146.5 (C-6a′), 138.4 (C-2), 134.4 (C-8a), 131.6 (C-2′), 127.7 (C-12a), 127.6 (C-4), 125.9 (C-7), 125.4 (C-5), 124.3 (C-11), 121.7 (C-10), 119.9 (C-8′), 117.9 (C-12), 113.3 (C-9), 108.2 (C-7′), 107.1 (C-3′), 103.4 (C-6), 101.0 (C-5′), 47.5 (C-1′); (+)-HRESIMS *m/z* 352.0841 [M+H]^+^ (calcd for C_19_H_15_^35^ClN_3_O_2_, 352.0847), *m/z* 354.0815 [M+H]^+^ (calcd for C_19_H_15_^37^ClN_3_O_2_, 354.0824).

#### 3.2.99. 5-Chloro-3-(1-(2-(pyrrolidin-1-yl)ethyl)-1*H*-imidazol-5-yl)-1*H*-indole (**98**) 

Using the general procedure, reaction of 5-chloro-1*H*-indole-3-carbaldehyde (0.050 g, 0.278 mmol) with aminomethyl pyrrolidine (0.038 mL, 0.278 mmol), K_2_CO_3_ (0.038 g, 0.278 mmol) and *p*-toluenesulfonylmethyl isocyanide (0.054 g, 0.278 mmol) in DMF (1 mL) followed by purification using silica flash chromatography (EtOAc) afforded the title compound as an orange oil (0.048 g, 55%). *R_f_* = 0.03 (EtOAc); IR (ATR) ν_max_ 3118, 2964, 2803, 1625, 1457, 1338, 1110, 921, 894, 800 cm^−1^; ^1^H NMR (400 MHz, DMSO-*d*_6_) δ 11.63 (1H, s, NH-8), 7.78 (1H, br s, H-2), 7.63 (1H, d, *J* = 2.6 Hz, H-7), 7.47 (1H, d, *J* = 8.6 Hz, H-9), 7.44 (1H, d, *J* = 2.0 Hz, H-12), 7.16 (1H, dd, *J* = 8.6, 2.1 Hz, H-10), 6.99 (1H, br s, H-4), 4.05 (2H, t, *J* = 6.7 Hz, H_2_-1′), 2.58 (2H, t, *J* = 6.7 Hz, H_2_-2′), 2.31–2.28 (4H, m, 2H_2_-4′), 1.59–1.55 (4H, m, 2H_2_-5′); ^13^C NMR (100 MHz, DMSO-*d*_6_) δ 138.1 (C-2), 134.5 (C-8a), 127.8 (C-12a), 127.3 (C-4), 126.2 (C-7), 125.1 (C-5), 124.3 (C-11), 121.7 (C-10), 117.9 (C-12), 113.4 (C-9), 103.7 (C-6), 55.7 (C-2′), 53.4 (2C-4′), 43.6 (C-1′), 23.0 (2C-5′); (+)-HRESIMS *m/z* 315.1371 [M+H]^+^ (calcd for C_17_H_20_ClN_4_, 315.1371).

#### 3.2.100. (*E*)-5-Chloro-3-(1-(3,7-dimethylocta-2,6-dien-1-yl)-1*H*-imidazol-5-yl)-1*H*-indole (**99**) 

Using the general procedure, reaction of 5-chloro-1*H*-indole-3-carbaldehyde (0.050 g, 0.278 mmol) with geranylamine (0.051 mL, 0.278 mmol), K_2_CO_3_ (0.038 g, 0.278 mmol) and *p*-toluenesulfonylmethyl isocyanide (0.054 g, 0.278 mmol) in DMF (1 mL) followed by purification using silica gel column chromatography (EtOAc) to afford the title product as a yellow oil (0.029 g, 29%). *R_f_* = 0.62 (EtOAc); IR (ATR) ν_max_ 2918, 1625, 1458, 1377, 1281, 1220, 1109, 921, 893, 799 cm^−1^; ^1^H NMR (400 MHz, DMSO-*d*_6_) δ 11.62 (1H, s, NH-8), 7.70 (1H, br s, H-2), 7.57 (1H, d, *J* = 2.5 Hz, H-7), 7.46 (1H, d, *J* = 8.6 Hz, H-9), 7.43 (1H, d, *J* = 1.8 Hz, H-12), 7.15 (1H, dd, *J* = 8.6, 2.0 Hz, H-10), 7.03 (1H, br s, H-4), 5.17 (1H, t, *J* = 6.7 Hz, H-2′), 4.97–4.95 (1H, m, H-6′), 4.55 (2H, d, *J* = 6.7 Hz, H_2_-1′), 1.95–1.88 (4H, m, H_2_-4′, H_2_-5′), 1.58 (3H, s, H_3_-10′), 1.49 (3H, s, H_3_-8′), 1.47 (3H, s, H_3_-9′); ^13^C NMR (100 MHz, DMSO-*d*_6_) δ 139.1 (C-3′), 137.4 (C-2), 134.5 (C-8a), 131.0 (C-7′), 127.8 (C-12a), 127.5 (C-4), 126.2 (C-7), 125.1 (C-5), 124.3 (C-11), 123.7 (C-6′), 121.7 (C-10), 120.1 (C-2′), 117.9 (C-12), 113.3 (C-9), 103.7 (C-6), 42.6 (C-1′), 38.7 (C-4′), 25.6 (C-10′), 25.4 (C-5′), 17.5 (C-8′), 15.8 (C-9′); (+)-HRESIMS *m/z* 354.1735 [M+H]^+^ (calcd for C_21_H_25_^35^ClN_3_, 354.1732), *m/z* 356.1706 [M+H]^+^ (calcd for C_21_H_25_^37^ClN_3_, 356.1709).

#### 3.2.101. 6-(5-(5-Chloro-1*H*-indol-3-yl)-1*H*-imidazol-1-yl)hexan-1-ol (**100**) 

Using the general procedure, reaction of 5-chloro-1*H*-indole-3-carbaldehyde (0.050 g, 0.278 mmol) with aminohexanol (0.037 g, 0.278 mmol), K_2_CO_3_ (0.038 g, 0.278 mmol) and *p*-toluenesulfonylmethyl isocyanide (0.054 g, 0.278 mmol) in DMF (1 mL) followed by purification using silica gel column chromatography (EtOAc) to afford the title compound as a yellow oil (0.068 g, 77%). *R_f_* = 0.03 (EtOAc); IR (ATR) ν_max_ 3220, 2932, 2859, 1626, 1458, 1226, 1111, 1050, 1024, 1004, 922, 894, 801, 760 cm^−1^; ^1^H NMR (400 MHz, DMSO-*d*_6_) δ 11.65 (1H, br s, NH-8), 7.76 (1H, br s, H-2), 7.61 (1H, d, *J* = 2.5 Hz, H-7), 7.48 (1H, d, *J* = 8.6 Hz, H-9), 7.43 (1H, d, *J* = 2.0 Hz, H-12), 7.16 (1H, dd, *J* = 8.6, 2.1 Hz, H-10), 7.02 (1H, br s, H-4), 4.31 (1H, br s, OH), 3.95 (2H, t, *J* = 7.0 Hz, H_2_-1′), 3.28 (2H, t, *J* = 6.6 Hz, H_2_-6′), 1.52 (2H, tt, *J* = 7.3, 7.0 Hz, H_2_-2′), 1.27 (2H, tt, *J* = 7.0, 6.6 Hz, H_2_-5′), 1.18–1.05 (4H, m, H_2_-3′, H_2_-4′); ^13^C NMR (100 MHz, DMSO-*d*_6_) δ 137.9 (C-2), 134.5 (C-8a), 127.8 (C-12a), 127.6 (C-4), 126.0 (C-7), 125.1 (C-5), 124.3 (C-11), 121.7 (C-10), 117.9 (C-12), 113.4 (C-9), 103.8 (C-6), 60.5 (C-6′), 44.4 (C-1′), 32.3 (C-5′), 30.2 (C-2′), 25.7 (C-4′), 24.9 (C-3′); (+)-HRESIMS *m/z* 318.1365 [M+H]^+^ (calcd for C_17_H_21_ClN_3_O, 318.1368).

#### 3.2.102. *Tert*-Butyl (6-(5-(5-chloro-1*H*-indol-3-yl)-1*H*-imidazol-1-yl)hexyl)carbamate (**101**) 

Using the general procedure, reaction of 5-chloro-1*H*-indole-3-carbaldehyde (0.050 g, 0.278 mmol) with *N*-Boc-1,6-hexanediamine (0.062 mL, 0.278 mmol), K_2_CO_3_ (0.038 g, 0.278 mmol) and *p*-toluenesulfonylmethyl isocyanide (0.054 g, 0.278 mmol) in DMF (1 mL) followed by purification using silica gel column chromatography (EtOAc) to afford the title product as an orange oil (0.099 g, 85%). *R_f_* = 0.03 (EtOAc); IR (ATR) ν_max_ 3228, 2932, 2860, 1684, 1512, 1457, 1366, 1337, 1276, 1166, 1109, 1028, 894, 799, 786, 703 cm^−1^; ^1^H NMR (400 MHz, DMSO-*d*_6_) δ 11.61 (1H, d, *J* = 1.5 Hz, NH-8), 7.75 (1H, br s, H-2), 7.60 (1H, d, *J* = 2.5 Hz, H-7), 7.47 (1H, d, *J* = 1.9 Hz, H-9), 7.42 (1H, d, *J* = 1.9 Hz, H-12), 7.16 (1H, dd, *J* = 8.7, 2.1 Hz, H-10), 7.00 (1H, br s, H-7), 6.68 (1H, t, *J* = 5.4 Hz, NH-7′), 3.95 (2H, t, *J* = 7.1 Hz, H_2_-1′), 2.80 (2H, q, *J* = 6.5 Hz, H_2_-6′), 1.54–1.48 (2H, m, H_2_-2′), 1.34 (9H, s, 3H_3_-11′), 1.26–1.20 (2H, m, H_2_-5′), 1.11–1.08 (4H, m, H_2_-3′, H_2_-4′); ^13^C NMR (100 MHz, DMSO-*d*_6_) δ 155.5 (C-8′), 137.9 (C-2), 134.5 (C-8a), 127.7 (C-12a), 127.5 (C-4), 126.0 (C-7), 125.0 (C-5), 124.3 (C-11), 121.7 (C-10), 117.8 (C-12), 113.4 (C-9) 103.7 (C-6), 77.2 (C-10′), 44.3 (C-1′), 39.6 (C-6′), 30.1 (C-2′), 29.2 (C-5′), 28.2 (3C-11′), 25.7 (C-4′), 25.5 (C-3′); (+)-HRESIMS *m/z* 417.2034 [M+H]^+^ (calcd for C_22_H_30_^35^ClN_4_O_2_, 417.2041), *m/z* 419.2012 [M+H]^+^ (calcd for C_22_H_30_^37^ClN_4_O_2_, 419.2020).

#### 3.2.103. 6-(5-(5-Chloro-1*H*-indol-3-yl)-1*H*-imidazol-1-yl)hexan-1-aminium 2,2,2-trifluoroacetate (**102**) 

A solution of *tert*-butyl (6-(5-(5-chloro-1*H*-indol-3-yl)-1*H*-imidazol-1-yl)hexyl)carbamate (**101**) (0.022 g, 0.0528 mmol) in CH_2_Cl_2_:TFA (10:1, 2.2 ml) was stirred for 2 h at r.t. under N_2_ atmosphere. The resulting solution was dried under reduced pressure and subjected to C_8_ reverse-phased column chromatography eluting with 3:1 H_2_O:MeOH (+0.05% TFA) to afford the title compound as an orange oil in quantitative yield as the TFA salt. *R_f_* = 0.71 (70% MeOH/30% 1M HCl, C_18_); IR (ATR) ν_max_ 3116, 2938, 1674, 1201, 1132, 1050, 1026, 1006, 896, 831, 800, 721 cm^−1^; ^1^H NMR (400 MHz, DMSO-*d*_6_) δ 12.05 (1H, d, *J* = 1.9 Hz, NH-8), 9.27 (1H, s, H-2), 7.89 (1H, s, H-4), 7.87 (1H, d, *J* = 2.7 Hz, H-7), 7.72 (3H, br s, NH_3_-7′), 7.58 (1H, d, *J* = 1.9 Hz, H-12), 7.55 (1H, d, *J* = 8.7 Hz, H-9), 7.23 (1H, dd, *J* = 8.8, 2.0 Hz, H-10), 4.16 (2H, t, *J* = 7.3 Hz, H_2_-1′), 2.73–2.64 (2H, m, H_2_-6′), 1.66–1.59 (2H, m, H_2_-2′), 1.45–1.38 (2H, m, H_2_-5′), 1.17–1.15 (4H, m, H_2_-3′, H_2_-4′); ^13^C NMR (100 MHz, DMSO-*d*_6_) δ 135.5 (C-2), 134.5 (C-8a), 128.5 (C-5), 127.5 (C-12a), 127.3 (C-7), 125.1 (C-11), 122.4 (C-10), 118.2 (C-4), 117.7 (C-12), 113.9 (C-9), 99.4 (C-6), 46.6 (C-1′), 38.6 (C-6′), 28.7 (C-2′), 26.7 (C-5′), 25.1 (C-3′), 25.0 (C-4′); (+)-HRESIMS *m/z* 317.1526 [M+H]^+^ (calcd for C_17_H_22_ClN_4_ 317.1528).

#### 3.2.104. 1-Benzyl-5-phenyl-1*H*-imidazole (**103**) 

Using the general procedure, reaction of benzaldehyde (46 µL, 0.417 mmol) with benzylamine (48 µL, 0.417 mmol), K_2_CO_3_ (0.060 g, 0.417 mmol) and *p*-toluenesulfonylmethyl isocyanide (0.084 g, 0.417 mmol) followed by purification using silica gel column chromatography (EtOAc) afforded the title compound as an off-white solid (0.013 g, 13%). R*_f_ =* 0.60 (CH_2_Cl_2_:MeOH, 9:1); m.p. 99–100 °C; IR (ATR) ν_max_ 3373, 3084, 2919, 2851, 1109, 725 cm^−1^; ^1^H NMR (400 MHz, CDCl_3_) δ 7.56 (1H, d, *J* = 1.0 Hz, H-2), 7.39–7.31 (3H, m, 2H-8, H-9), 7.31–7.25 (5H, m, 2H-7, 2H-4′, H-5′), 7.14 (1H, d, *J* = 1.0 Hz, H-4), 7.03–6.99 (2H, m, 2H-3′), 5.15 (2H, s, H_2_-1′); ^13^C NMR (100 MHz, CDCl_3_) δ 138.8 (C-2), 136.9 (C-2′), 133.6 (C-5), 129.8 (C-6), 129.0 (2C-8, 2C-4′), 128.8 (2C-7), 128.3 (C-9/C-5′), 128.2 (C-9/C-5′), 128.1 (C-4), 126.8 (2C-3′), 48.9 (C-1′); (+)-HRESIMS *m/z* 235.1233 [M+H]^+^ (calcd for C_16_H_15_N_2_, 235.1230). 

#### 3.2.105. 1-Benzyl-5-(4-methoxyphenyl)-1*H*-imidazole (**104**) 

Using the general procedure, reaction of *p*-methoxybenzaldehyde (23 µL, 0.189 mmol) with benzylamine (21 µL, 0.189 mmol), K_2_CO_3_ (0.026 g, 0.189 mmol) and *p*-toluenesulfonylmethyl isocyanide (0.037 g, 0.189 mmol) followed by purification using silica gel column chromatography (CH_2_Cl_2_:MeOH, 1:0→9:1) afforded the title compound as a yellow solid (0.025 g, 50%). R*_f_ =* 0.60 (CH_2_Cl_2_:MeOH, 9:1); m.p. 83–84 °C; IR (ATR) ν_max_ 3351, 2920, 2851, 1251, 730 cm^−1^; ^1^H NMR (400 MHz, CDCl_3_) δ 7.54 (1H, d, *J* = 1.0 Hz, H-2), 7.33–7.24 (3H, m, 2H-4′, H-5′), 7.21–7.16 (2H, m, 2H-7), 7.07 (1H, d, *J* = 1.0 Hz, H-4), 7.02–6.98 (2H, m, 2H-3′), 6.91–6.86 (2H, m, 2H-8′), 5.10 (2H, s, H_2_-1′), 3.80 (3H, s, OMe); ^13^C NMR (100 MHz, CDCl_3_) δ 159.7 (C-9), 138.4 (C-2), 137.0 (C-2′), 133.3 (C-5), 130.5 (2C-7), 129.0 (2C-4′), 128.0 (C-5′), 127.9 (C-4), 126.8 (2C-3′), 122.1 (C-6), 114.2 (2C-8), 55.4 (OMe), 48.7 (C-1′); (+)-HRESIMS *m/z* 265.1333 [M+H]^+^ (calcd for C_17_H_17_N_2_O, 265.1335). 

#### 3.2.106. 1-Phenethyl-5-phenyl-1*H*-imidazole (**105**) 

Using the general procedure, reaction of benzaldehyde (21 µL, 0.201 mmol) was reacted with phenethylamine (25 µL, 0.201 mmol), K_2_CO_3_ (0.028 g, 0.201 mmol) and *p*-toluenesulfonylmethyl isocyanide (0.039 g, 0.201 mmol) followed by purification using silica gel column chromatography (EtOAc) afforded the title compound as a yellow oil (0.029 g, 58%). R*_f_* = 0.53 (CH_2_Cl_2_:MeOH, 9:1); IR (ATR) ν_max_ 3376, 3029, 2932, 2856, 1484, 698 cm^−1^; ^1^H NMR (400 MHz, CDCl_3_) δ 7.45–7.35 (3H, m, 2H-8, H-9), 7.38 (1H, d, *J* = 1.0 Hz, H-2), 7.33–7.28 (2H, m, 2H-7), 7.26–7.16 (3H, m, 2H-5′, H-6′), 7.04 (1H, d, *J* = 1.0 Hz, H-4), 6.95–6.90 (2H, m, 2H-4′), 4.19 (2H, t, *J* = 7.3 Hz, H_2_-1′), 2.85 (2H, t, *J* = 7.3 Hz, H_2_-2′); ^13^C NMR (100 MHz, CDCl_3_) δ 138.2 (C-2), 137.4 (C-3′), 132.9 (C-5), 130.2 (C-6), 129.0 (2C-8), 128.9 (2C-5′), 128.8 (2C-4′), 128.7 (2C-7), 128.4 (C-4), 128.2 (C-9), 127.0 (C-6′), 46.9 (C-1′), 37.5 (C-2′); (+)-HRESIMS *m/z* 249.1390 [M+H]^+^ (calcd for C_17_H_17_N_2_, 249.1386). 

#### 3.2.107. 5-(4-Methoxyphenyl)-1-phenethyl-1*H*-imidazole (**106**) 

Using the general procedure, reaction of *p*-methoxybenzaldehyde (22 µL, 0.180 mmol) was reacted with phenethylamine (23 µL, 0.180 mmol), K_2_CO_3_ (0.025 g, 0.180 mmol) and *p*-toluenesulfonylmethyl isocyanide (0.035 g, 0.180 mmol) followed by purification using silica gel column chromatography (EtOAc) afforded the title compound as a yellow oil (0.033 g, 66%). R*_f_* = 0.47 (CH_2_Cl_2_:MeOH, 9:1); IR (ATR) ν_max_ 3079, 3028, 2835, 1454, 1248, 698 cm^−1^; ^1^H NMR (400 MHz, CDCl_3_) δ 7.36 (1H, d, *J* = 1.0 Hz, H-2), 7.27–7.21 (2H, m, 2H-5′), 7.23–7.18 (3H, m, 2H-7, H-6′), 6.98 (1H, d, *J* = 1.0 Hz, H-4), 6.97–6.92 (4H, 2H-8, 2H-4′), 4.14 (2H, t, *J* = 7.3 Hz, H_2_-1′), 3.85 (3H, s, OMe), 2.85 (2H, t, *J* = 7.3 Hz, H_2_-2′); ^13^C NMR (100 MHz, CDCl_3_) δ 159.7 (C-9), 137.8 (C-2), 137.5 (C-3′), 132.7 (C-5), 130.4 (2C-7), 128.8 (2C-5′), 128.7 (2C-4′), 127.9 (C-4), 127.0 (C-6′), 122.5 (C-6), 114.3 (2C-8), 55.5 (OMe), 46.8 (C-1′), 37.5 (C-2′); (+)-HRESIMS *m/z* 279.1494 [M+H]^+^ (calcd for C_18_H_19_N_2_O, 279.1492). 

#### 3.2.108. 1-(4-Methoxybenzyl)-5-phenyl-1*H*-imidazole (**107**) 

Using the general procedure, reaction of benzaldehyde (17 µL, 0.170 mmol) with *p*-methoxybenzylamine (22 µL, 0.170 mmol), K_2_CO_3_ (0.024 g, 0.170 mmol) and *p*-toluenesulfonylmethyl isocyanide (0.033 g, 0.170 mmol) followed by purification using silica gel column chromatography (EtOAc) afforded the title compound as a clear oil (0.006 g, 13%). R*_f_* = 0.60 (CH_2_Cl_2_:MeOH, 9:1); IR (ATR) ν_max_ 3392, 2933, 2837, 1513, 1249, 769 cm^−1^; ^1^H NMR (400 MHz, CDCl_3_) δ 7.53 (1H, d, *J* = 1.0 Hz, H-2), 7.40–7.33 (3H, m, 2H-8, H-9), 7.32–7.28 (2H, m, 2H-7), 7.12 (1H, d, *J* = 1.0 Hz, H-4), 6.98–6.93 (2H, m, 2H-3′), 6.85–6.80 (2H, m, 2H-4′), 5.08 (2H, s, H_2_-1′), 3.78 (3H, s, OMe); ^13^C NMR (100 MHz, CDCl_3_) δ 159.4 (C-5′), 138.6 (C-2), 133.5 (C-5), 130.0 (C-6), 129.1 (2C-7), 128.80 (2C-8), 128.79 (C-2′), 128.4 (C-4/C-9), 128.3 (2C-3′), 128.2 (C-4/C-9), 114.4 (2C-4′), 55.4 (OMe), 48.5 (C-1′); (+)-HRESIMS *m/z* 265.1342 [M+H]^+^ (calcd for C_17_H_17_N_2_O, 265.1335). 

#### 3.2.109. 1-(4-Methoxybenzyl)-5-(4-methoxyphenyl)-1*H*-imidazole (**108**) 

Using the general procedure, reaction of *p*-methoxybenzaldehyde (21 µL, 0.170 mmol) with *p*-methoxybenzylamine (22 µL, 0.170 mmol), K_2_CO_3_ (0.024 g, 0.170 mmol) and *p*-toluenesulfonylmethyl isocyanide (0.033 g, 0.170 mmol) followed by purification using silica gel column chromatography (EtOAc) afforded the title compound as a yellow oil (0.022 g, 44%). R*_f_ =* 0.56 (CH_2_Cl_2_:MeOH, 9:1); IR (ATR) ν_max_ 3371, 2934, 2836, 1512, 1246, 818 cm^−1^; ^1^H NMR (400 MHz, CDCl_3_) δ 7.51 (1H, d, *J* = 1.0 Hz, H-2), 7.22–7.18 (2H, m, 2H-7), 7.05 (1H, d, *J* = 1.0 Hz, H-4), 6.97–6.92 (2H, m, 2H-3′), 6.92–6.88 (2H, m, 2H-8), 6.84–6.80 (2H, m, 2H-4′), 5.03 (2H, s, H_2_-1′), 3.82 (3H, s, 9-OMe), 3.78 (3H, s, 5′-OMe); ^13^C NMR (100 MHz, CDCl_3_) δ 159.7 (C-9), 159.4 (C-5′), 138.2 (C-2), 133.2 (C-5), 130.5 (2C-7), 128.9 (C-2′), 128.3 (2C-3′), 127.9 (C-4), 122.3 (C-6), 114.4 (2C-4′), 114.2 (2C-8), 55.43 (9-OMe), 55.42 (5′-OMe), 48.3 (C-1′); (+)-HRESIMS *m/z* 295.1448 [M+H]^+^ (calcd for C_18_H_19_N_2_O_2_, 295.1441). 

#### 3.2.110. 1-(4-Methoxyphenethyl)-5-phenyl-1*H*-imidazole (**109**)

Using the general procedure, reaction of benzaldehyde (36 µL, 0.358 mmol) was reacted with *p*-methoxyphenethylamine hydrochloride (0.064 g, 0.358 mmol), K_2_CO_3_ (0.050 g, 0.358 mmol) and *p*-toluenesulfonylmethyl isocyanide (0.070 g, 0.358 mmol) followed by purification using a combination of silica gel column chromatography (EtOAc) and reversed-phase C_8_ column chromatography (H_2_O:MeOH, 1:0→0:1) afforded the title compound as a yellow gum (0.005 g, 5%). R*_f_* = 0.54 (CH_2_Cl_2_:MeOH, 9:1); IR (ATR) ν_max_ 3367, 3061, 2932, 1513, 1247, 763 cm^−1^; ^1^H NMR (400 MHz, CDCl_3_) δ 7.45–7.35 (4H, m, H-2, 2H-8, H-9), 7.33–7.29 (2H, m, 2H-7), 7.05 (1H, br s, H-4), 6.86–6.80 (2H, m, 2H-4′), 6.79–6.73 (2H, m, 2H-5′), 4.16 (2H, t, *J* = 7.4 Hz, H_2_-1′), 3.77 (3H, s, OMe), 2.79 (2H, t, *J* = 7.4 Hz, H_2_-2′); ^13^C NMR (100 MHz, CDCl_3_) δ 158.7 (C-6′), 138.3 (C-2), 133.0 (C-5), 130.2 (C-6), 129.7 (2C-4′), 129.4 (C-3′), 129.0 (2C-8), 128.9 (2C-7), 128.2 (C-4, C-9), 114.3 (2C-5′), 55.4 (OMe), 47.2 (C-1′), 36.6 (C-2′); (+)-HRESIMS *m/z* 279.1499 [M+H]^+^ (calcd for C_18_H_19_N_2_O, 279.1492).

#### 3.2.111. 1-(4-Methoxyphenethyl)-5-(4-methoxyphenyl)-1*H*-imidazole (**110**) 

Using the general procedure, reaction of *p*-methoxybenzaldehyde (20 µL, 0.162 mmol) was reacted with *p*-methoxyphenethylamine hydrochloride (0.030 g, 0.162 mmol), K_2_CO_3_ (0.023 g, 0.162 mmol) and *p*-toluenesulfonylmethyl isocyanide (0.032 g, 0.162 mmol) followed by purification using silica gel column chromatography (EtOAc) afforded the title compound as a yellow gum (0.006 g, 12%). R*_f_* = 0.54 (CH_2_Cl_2_:MeOH, 9:1); IR (ATR) ν_max_ 3116, 2930, 2839, 1513, 1248, 821 cm^−1^; ^1^H NMR (400 MHz, CDCl_3_) δ 7.35 (1H, br s, H-2), 7.24–7.20 (2H, m, 2H-7), 6.98 (1H, br s, H-4), 6.97–6.94 (2H, m, 2H-8), 6.87–6.84 (2H, m, 2H-4′), 6.80–6.75 (2H, m, 2H-5′), 4.11 (2H, t, *J* = 7.0 Hz, H_2_-1′), 3.86 (3H, s, 9-OMe), 3.77 (3H, s, 6′-OMe), 2.79 (2H, t, *J* = 7.0 Hz, H_2_-2′); ^13^C NMR (100 MHz, CDCl_3_) δ 159.7 (C-9), 158.7 (C-6′), 137.9 (C-2), 132.7 (C-5), 130.4 (2C-7), 129.7 (2C-4′), 129.5 (C-3′), 128.0 (C-4), 122.6 (C-6), 114.3 (2C-8/2C-5′), 114.2 (2C-8/2C-5′), 55.5 (9-OMe), 55.4 (6′-OMe), 47.0 (C-1′), 36.7 (C-2′); (+)-HRESIMS *m/z* 309.1602 [M+H]^+^ (calcd for C_19_H_21_N_2_O_2_, 309.1598). 

#### 3.2.112. 3-((5-Phenyl-1*H*-imidazol-1-yl)methyl)-1*H*-indole (**111**) 

Using the general procedure, reaction of benzaldehyde (19 µL, 0.183 mmol) was reacted with (1*H*-indol-3-yl)methanamine (0.027 g, 0.183 mmol), K_2_CO_3_ (0.025 g, 0.183 mmol) and *p*-toluenesulfonylmethyl isocyanide (0.036 g, 0.183 mmol) followed by purification using silica gel column chromatography (EtOAc) afforded the title compound as a yellow solid (0.014 g, 28%). R*_f_* = 0.49 (CH_2_Cl_2_:MeOH, 9:1); m.p. 57–58 ℃; IR (ATR) ν_max_ 3412, 2924, 2849, 1110, 744 cm^−1^; ^1^H NMR (400 MHz, CDCl_3_) δ 8.69 (1H, br s, NH-4′), 7.56 (1H, d, *J* = 1.0 Hz, H-2), 7.44–7.39 (5H, m, 2H-7, 2H-8, H-9), 7.38–7.33 (2H, m, H-5′, H-8′), 7.20 (1H, ddd, *J* = 8.3, 7.0, 1.0 Hz, H-6′), 7.13 (1H, d, *J* = 1.0 Hz, H-4), 7.09 (1H, ddd, *J* = 8.0, 7.0, 1.0 Hz, H-7′), 6.91 (1H, d, *J* = 2.5 Hz, H-3′), 5.30 (2H, s, H_2_-1′); ^13^C NMR (100 MHz, CDCl_3_) δ 138.5 (C-2), 136.6 (C-4a′), 133.4 (C-5), 130.2 (C-6), 129.0 (2C-8), 128.9 (2C-7), 128.1 (C-4/C-9), 128.0 (C-4/C-9), 126.0 (C-8a′), 123.5 (C-3′), 122.8 (C-6′), 120.3 (C-7′), 118.4 (C-8′), 111.6 (C-5′), 111.5 (C-2′), 41.5 (C-1′); (+)-HRESIMS *m/z* 274.1339 [M+H]^+^ (calcd for C_18_H_16_N_3_, 274.1339).

#### 3.2.113. 3-((5-(4-Methoxyphenyl)-1*H*-imidazol-1-yl)methyl)-1*H*-indole (**112**) 

Using the general procedure, reaction of *p*-methoxybenzaldehyde (20 µL, 0.165 mmol) with (1*H*-indol-3-yl)methanamine (0.024 g, 0.165 mmol), K_2_CO_3_ (0.023 g, 0.165 mmol) and *p*-toluenesulfonylmethyl isocyanide (0.032 g, 0.165 mmol) in DMF (1 mL) followed by purification using silica gel column chromatography (EtOAc) afforded the title compound as a yellow solid (0.026 g, 52%). R*_f_ =* 0.47 (CH_2_Cl_2_:MeOH, 9:1); m.p. 152–153 ℃; IR (ATR) ν_max_ 3417, 3105, 2925, 1250, 744 cm^−1^; ^1^H NMR (400 MHz, CDCl_3_) δ 8.38 (1H, br s, NH-4′), 7.55 (1H, s, H-2), 7.37 (1H, dd, *J* = 8.3, 1.0 Hz, H-5′), 7.36 (1H, d, *J* = 8.3 Hz, H-8′), 7.34–7.30 (2H, m, 2H-7), 7.21 (1H, ddd, *J* = 8.3, 7.2, 1.0 Hz, H-6′), 7.10 (1H, ddd, *J* = 8.3, 7.2, 1.0 Hz, H-7′), 7.06 (1H, d, *J* = 1.0 Hz, H-4), 6.91 (1H, d, *J* = 2.5 Hz, H-3′), 5.27 (2H, s, H_2_-1′), 3.83 (3H, s, OMe); ^13^C NMR (100 MHz, CDCl_3_) δ 159.6 (C-9), 138.2 (C-2), 136.5 (C-4a′), 133.1 (C-5), 130.4 (2C-7), 127.6 (C-4), 126.0 (C-8a′), 123.3 (C-3′), 122.8 (C-6′), 122.6 (C-6), 120.3 (C-7′), 118.5 (C-8′), 114.3 (2C-8), 111.9 (C-2′), 111.6 (C-5′), 55.5 (OMe), 41.3 (C-1′); (+)-HRESIMS *m/z* 304.1444 [M+H]^+^ (calcd for C_19_H_18_N_3_O, 304.1444). 

#### 3.2.114. 3-(2-(5-Phenyl-1*H*-imidazol-1-yl)ethyl)-1*H*-indole (**113**) 

Using the general procedure, reaction of benzaldehyde (17 µL, 0.174 mmol) with tryptamine (0.028 g, 0.174 mmol), K_2_CO_3_ (0.024 g, 0.174 mmol) and *p*-toluenesulfonylmethyl isocyanide (0.034 g, 0.174 mmol) followed by purification using silica gel column chromatography (CH_2_Cl_2_:MeOH, 1:0→9:1) afforded the title compound as a brown solid (0.023 g, 46%). R*_f_ =* 0.40 (CH_2_Cl_2_:MeOH, 9:1); m.p. 155–156 ℃; IR (ATR) ν_max_ 3417, 3170, 2921, 1482, 1221, 742 cm^−1^; ^1^H NMR (400 MHz, CDCl_3_) δ 8.27 (1H, br s, NH-5′), 7.43–7.35 (4H, m, H-4, 2H-7, H-9), 7.35–7.30 (3H, m, 2H-8, H-6′), 7.28 (1H, dd, *J* = 7.8, 0.9 Hz, H-9′), 7.18 (1H, ddd, *J* = 6.9, 6.9, 0.9 Hz, H-7′), 7.06 (1H, d, *J* = 1.5 Hz, H-4), 7.06 (1H, ddd, *J* = 7.8, 6.9, 1.0 Hz, H-8′), 6.73 (1H, d, *J* = 2.2 Hz, H-4′), 4.25 (2H, t, *J* = 7.1 Hz, H_2_-1′), 3.02 (2H, t, *J* = 7.1 Hz, H_2_-2′); ^13^C NMR (100 MHz, CDCl_3_) δ 138.5 (C-2), 136.4 (C-5a′), 133.0 (C-5), 130.3 (C-6), 129.0 (2C-8), 128.9 (2C-7), 128.3 (C-4/C-9), 128.2 (C-4/C-9), 127.0 (C-9a′), 122.5 (C-7′), 122.3 (C-4′), 119.7 (C-8′), 118.3 (C-9′), 111.5 (C-3′, C-6′), 46.0 (C-1′), 27.3 (C-2′); (–)-HRESIMS *m/z* 286.1351 [M−H]^−^ (calcd for C_19_H_16_N_3_, 286.1350). 

#### 3.2.115. 3-(2-(5-(4-Methoxyphenyl)-1*H*-imidazol-1-yl)ethyl)-1*H*-indole (**114**) 

Using the general procedure, reaction of *p*-methoxybenzaldehyde (19 µL, 0.157 mmol) with tryptamine (0.025 g, 0.157 mmol), K_2_CO_3_ (0.022 g, 0.157 mmol) and *p*-toluenesulfonylmethyl isocyanide (0.031 g, 0.157 mmol) followed by purification using silica gel column chromatography (CH_2_Cl_2_:MeOH, 1:0→9:1) afforded the title compound as a yellow solid (0.030 g, 60%). R*_f_* = 0.40 (CH_2_Cl_2_:MeOH, 9:1); m.p. 169–170 °C; IR (ATR) ν_max_ 3412, 3163, 2929, 1488, 1250, 744 cm^−1^; ^1^H NMR (400 MHz, CDCl_3_) δ 8.31 (1H, br s, NH-5′), 7.36 (1H, d, *J* = 1.0 Hz, H-2), 7.33 (1H, d, *J* = 8.3 Hz, H-6′), 7.28 (1H, d, *J* = 8.3 Hz, H-9′), 7.22 (2H, d, *J* = 8.8 Hz, 2H-7), 7.18 (1H, ddd, *J* = 8.3, 7.0, 1.0 Hz, H-7′), 7.06 (1H, ddd, *J* = 8.3, 7.0, 1.0 Hz, H-8′), 7.00 (1H, d, *J* = 1.0 Hz, H-4), 6.92 (2H, d, *J* = 8.8 Hz, 2H-8), 6.73 (1H, d, *J* = 2.5 Hz, H-4′), 4.21 (2H, t, *J* = 7.2 Hz, H_2_-1′), 3.85 (3H, s, OMe), 3.01 (2H, t, *J* = 7.2 Hz, H_2_-2′); ^13^C NMR (100 MHz, CDCl_3_) δ 159.7 (C-9), 138.0 (C-2), 136.4 (C-5a′), 132.8 (C-5), 130.5 (2C-7), 127.8 (C-4), 127.0 (C-9a′), 122.6 (C-6), 122.5 (C-4′/C-7′), 122.3 (C-4′/C-7′), 119.6 (C-8′), 118.3 (C-9′), 114.3 (2C-8), 111.51 (C-3′), 111.47 (C-6′), 55.5 (OMe), 45.8 (C-1′), 27.4 (C-2′); (–)-HRESIMS *m/z* 316.1452 [M−H]^−^ (calcd for C_20_H_18_N_3_O, 316.1455). 

#### 3.2.116. 1-Pentyl-5-phenyl-1*H*-imidazole (**115**) 

Using the general procedure, reaction of benzaldehyde (24 µL, 0.233 mmol) with pentylamine (27 µL, 0.233 mmol), K_2_CO_3_ (0.032 g, 0.233 mmol) and *p*-toluenesulfonylmethyl isocyanide (0.046 g, 0.233 mmol) followed by purification using silica gel column chromatography (EtOAc) afforded the title compound as a yellow gum (0.015 g, 30%). R*_f_ =* 0.53 (CH_2_Cl_2_:MeOH, 9:1); IR (ATR) ν_max_ 3221, 2957, 2930, 2859, 1481, 1114, 764 cm^−1^; ^1^H NMR (400 MHz, CDCl_3_) δ 7.55 (1H, br s, H-2), 7.46–7.40 (2H, m, 2H-8), 7.40–7.36 (3H, m, 2H-7, H-9), 7.06 (1H, br s, H-4), 3.95 (2H, t, *J* = 7.3 Hz, H_2_-1′), 1.62 (2H, p, *J* = 7.3 Hz, H_2_-2′), 1.28–1.12 (4H, m, H_2_-3′, H_2_-4′), 0.82 (3H, t, *J* = 6.9 Hz, H_3_-5′); ^13^C NMR (100 MHz, CDCl_3_) δ 138.2 (C-2), 133.1 (C-5), 130.4 (C-6), 128.9 (2C-8), 128.8 (2C-7), 128.3 (C-4), 128.1 (C-9), 45.5 (C-1′), 30.7 (C-2′), 28.7 (C-3′), 22.1 (C-4′), 13.9 (C-5′); (+)-HRESIMS *m/z* 215.1549 [M+H]^+^ (calcd for C_14_H_19_N_2_, 215.1543). 

#### 3.2.117. 5-(4-Methoxyphenyl)-1-pentyl-1*H*-imidazole (**116**) 

Using the general procedure, reaction of *p*-methoxybenzaldehyde (25 µL, 0.205 mmol) with pentylamine (24 µL, 0.205 mmol), K_2_CO_3_ (0.028 g, 0.205 mmol) and *p*-toluenesulfonylmethyl isocyanide (0.040 g, 0.205 mmol) followed by purification using silica gel column chromatography (CH_2_Cl_2_:MeOH, 1:0→9:1) afforded the title compound as a yellow gum (0.028 g, 56%). R*_f_ =* 0.54 (CH_2_Cl_2_:MeOH, 9:1); IR (ATR) ν_max_ 3237, 2957, 2930, 2860, 1482, 1249, 763 cm^−1^; ^1^H NMR (400 MHz, CDCl_3_) δ 7.52 (1H, br s, H-2), 7.28–7.25 (2H, m, 2H-7), 6.99 (1H, br s, H-4), 6.98–6.93 (2H, m, 2H-8), 3.90 (2H, t, *J* = 7.2 Hz, H_2_-1′), 3.85 (3H, s, OMe), 1.61 (2H, p, *J* = 7.2 Hz, H_2_-2′), 1.28–1.12 (2H, m, H_2_-3′, H_2_-4′), 0.83 (3H, t, *J* = 6.8 Hz, H_3_-5′); ^13^C NMR (100 MHz, CDCl_3_) δ 159.5 (C-9), 137.7 (C-2), 132.8 (C-5), 130.3 (C-7), 127.8 (C-4), 122.6 (C-6), 114.2 (C-8), 55.4 (OMe), 45.3 (C-1′), 30.7 (C-2′), 28.7 (C-3′), 22.1 (C-4′), 13.9 (C-5′); (+)-HRESIMS *m/z* 245.1653 [M+H]^+^ (calcd for C_15_H_21_N_2_O, 245.1648). 

### 3.3. Biological Evaluation

#### 3.3.1. Antimicrobial Assays 

The susceptibility of *S. aureus* (ATCC25923) to antibiotics and compounds was determined in microplates using the standard broth dilution method in accordance with the recommendations of the Comité de l’AntibioGramme de la Société Française de Microbiologie (CA-SFM) as previously described. Briefly, the minimal inhibitory concentrations (MICs) were determined with an inoculum of 10^5^ CFU in 200 µL of Mueller-Hinton broth (MHB) containing two-fold serial dilutions of each drug. The MIC was defined as the lowest concentration of drug that completely inhibited visible growth after incubation for 18 h at 37 °C. To determine all MICs, the measurements were independently repeated in triplicate [21]. 

Additional antimicrobial evaluation against *Staphylococcus aureus* MRSA (ATCC43300), *Pseudomonas aeruginosa* (ATCC27853), *Escherichia coli* (ATCC25922), *Klebsiella pneumoniae* (ATCC700603), *Acinetobacter baumannii* (ATCC19606), *Candida albicans* (ATCC90028), and *Cryptococcus neoformans* (ATCC208821) was undertaken at the Community for Open Antimicrobial Drug Discovery at The University of Queensland (Australia) according to their standard protocols [22]. For antimicrobial assays, the tested strains were cultured in either Luria broth (LB) (In Vitro Technologies, USB75852, Noble Park North, Australia), nutrient broth (NB) (Becton Dickson, 234000, Macquarie Park, Australia), or cation-adjusted MHB at 37 °C overnight. A sample of culture was then diluted 40-fold in fresh MHB and incubated at 37 °C for 1.5−2 h. The compounds were serially diluted 2-fold across the wells of 96-well plates (Corning 3641, Tewksbury, MA, USA, nonbinding surface), with compound concentrations ranging from 0.015 to 64 μg/mL, plated in duplicate. The resultant mid log phase cultures were diluted to the final concentration of 1 × 10^6^ CFU/mL; then, 50 μL was added to each well of the compound containing plates giving a final compound concentration range of 0.008 to 32 μg/mL and a cell density of 5 × 10^5^ CFU/mL. All plates were then covered and incubated at 37 °C for 18 h. Resazurin was added at 0.001% final concentration to each well and incubated for 2 h before MICs were read by eye.

For the antifungal assay, fungi strains were cultured for 3 days on yeast extract-peptone dextrose (YPD) agar at 30 °C. A yeast suspension of 1 × 10^6^ to 5 × 10^6^ CFU/mL was prepared from five colonies. These stock suspensions were diluted with yeast nitrogen base (YNB) (Becton Dickinson, 233520) broth to a final concentration of 2.5 × 10^3^ CFU/mL. The compounds were serially diluted 2-fold across the wells of 96-well plates (Corning 3641, nonbinding surface), with compound concentrations ranging from 0.015 to 64 μg/mL and final volumes of 50 μL, plated in duplicate. Then, 50 μL of the fungi suspension that was previously prepared in YNB broth to the final concentration of 2.5 × 10^3^ CFU/mL was added to each well of the compound-containing plates, giving a final compound concentration range of 0.008 to 32 μg/mL. Plates were covered and incubated at 35 °C for 36 h without shaking. *C. albicans* MICs were determined by measuring the absorbance at OD_530_. For *C. neoformans*, resazurin was added at 0.006% final concentration to each well and incubated for a further 3 h before MICs were determined by measuring the absorbance at OD_570–600_.

Colistin and vancomycin were used as positive bacterial inhibitor standards for Gram-negative and Gram-positive bacteria, respectively. Fluconazole was used as a positive fungal inhibitor standard for *C. albicans* and *C. neoformans*. The antibiotics were provided in 4 concentrations, with 2 above and 2 below its MIC value, and plated into the first 8 wells of column 23 of the 384-well NBS plates. The quality control (QC) of the assays was determined by the antimicrobial controls and the Z′-factor (using positive and negative controls). Each plate was deemed to fulfil the quality criteria (pass QC), if the Z′-factor was above 0.4, and the antimicrobial standards showed full range of activity, with full growth inhibition at their highest concentration, and no growth inhibition at their lowest concentration.

#### 3.3.2. Cytotoxicity Assays

For the HEK293 cytotoxicity assay, cells were counted manually in a Neubauer hemocytometer and plated at a density of 5000 cells/well into each well of the 384-well plates containing the 25× (2 μL) concentrated compounds. The medium used was Dulbecco’s modified eagle medium (DMEM) supplemented with 10% fetal bovine serum (FBS). Cells were incubated together with the compounds for 20 h at 37 °C, 5% CO_2_. To measure cytotoxicity, 5 μL (equals 100 μM final) of resazurin was added to each well after incubation, and incubated for further 3 h at 37 °C with 5% CO_2_. After final incubation fluorescence intensity was measured as Fex 560/10 nm, em 590/10 nm (F_560/590_) using a Tecan M1000 Pro monochromator plate reader. CC_50_ values (concentration at 50% cytotoxicity) were calculated by normalizing the fluorescence readout, with 74 μg/mL tamoxifen as negative control (0%) and normal cell growth as positive control (100%). The concentration-dependent percentage cytotoxicity was fitted to a dose response function (using Pipeline Pilot) and CC_50_ values determined [22]. 

#### 3.3.3. Hemolytic Assay 

Human whole blood was washed three times with 3 volumes of 0.9% NaCl and then resuspended in same to a concentration of 0.5 × 10^8^ cells/mL, as determined by manual cell count in a Neubauer hemocytometer. The washed cells were then added to the 384-well compound-containing plates for a final volume of 50 μL. After a 10 min shake on a plate shaker the plates were then incubated for 1 h at 37 °C. After incubation, the plates were centrifuged at 1000 g for 10 min to pellet cells and debris, 25 μL of the supernatant was then transferred to a polystyrene 384-well assay plate. Hemolysis was determined by measuring the supernatant absorbance at 405 mm (OD_405_). The absorbance was measured using a Tecan M1000 Pro monochromator plate reader. HC_10_ and HC_50_ (concentration at 10% and 50% hemolysis, respectively) were calculated by curve fitting the inhibition values vs. log(concentration) using a sigmoidal dose–response function with variable fitting values for top, bottom and slope [22]. 

#### 3.3.4. Membrane Depolarization Assay

*S. aureus* (ATCC25923) was grown in MH II broth for 24 h at 37 °C. After reaching an OD_600 nm_ of 0.5, cells were centrifuged (3600× *g* for 20 min at 20 °C) and washed twice with buffered sucrose solution (250 mM), magnesium sulfate solution (25 mM) and Hepes (5 mM) (pH = 7.2). The fluorescent dye 3,3′-diethylthiacarbocyanine iodide DiSC_3_(5) was added to a final concentration of 5 µM and was incubated with the suspensions for 5 min at 37 °C to allow the dye incorporation into the polarized membranes. 10 µL of compound was then added to 90 µL of the fluorescent suspensions at different concentrations ranging from 200 µM to 25 µM. Fluorescence measurements were recorded for 20 minutes (excitation wavelength 622 nm, emission wavelength 690 nm).

The difference in the relative fluorescence values (RFU) from the control containing only buffer and the control containing bacteria treated only with cetyltrimethylammonium bromide (CTAB 1%) is taken as the maximum level of depolarization. Assays were performed in three independent experiments. Blank determined by using no compound. Maximum RFU determined by using CTAB 0.1%

Equation used:

$$\frac{\left(\mathrm{RFU}\;\mathrm{of}\;\mathrm{the}\;\mathrm{compound}-\mathrm{RFU}\;\mathrm{of}\;\mathrm{the}\;\mathrm{blank}\right)\times 100}{\mathrm{RFU}\;\mathrm{of}\;\mathrm{the}\;\mathrm{maximum}\;}$$

## 4. Conclusions

In a bid to combat the surging increase of antibiotic-resistant bacteria a screening programme of a library of natural products, semi-synthetics and synthetic compounds was initiated targeting the a subset of the ESKAPE pathogens. Following our discovery of weak anti-MRSA activity associated with two 3-substituted-1*H*-imidazol-5-yl-1*H*-indoles we have synthesized a further 99 analogues as well as 14 examples of structurally related 5-phenyl-1*H*-imidazoles. Surprisingly few examples from the extended set of analogues exhibited antimicrobial properties, with only two examples (**26** and **32**) identified as being promising non-toxic selective anti-MRSA compounds. Of note was the identification of two analogues, **57** (a phenethyl-indole-imidazole) and **111** (a 5-phenyl-1*H*-imidazole) as being non-toxic selective antifungals towards *C. neoformans*. Further elaboration of these anti-MRSA and antifungal compounds will be the subject of future research.

## Figures and Tables

**Scheme 1:**
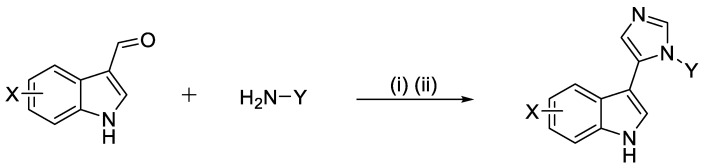
Synthetic route to 3-substituted-1H-imidazol-5-yl-1H-indoles **4**–**42**. Reagents and conditions: (i) DMF, 3 h, then (ii) TOSMIC, K2CO3, 60 °C, 24 h.

**Scheme 2:**
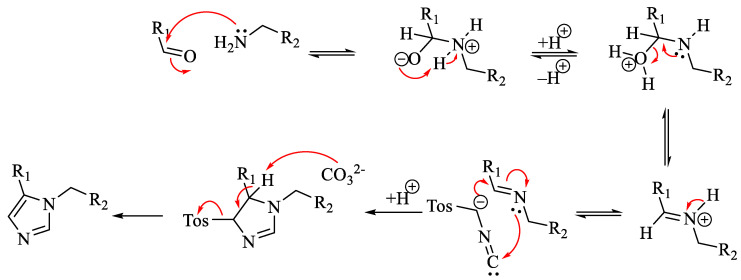
Proposed reaction mechanism of Van Leusen three-component reaction.

**Figure 3:**
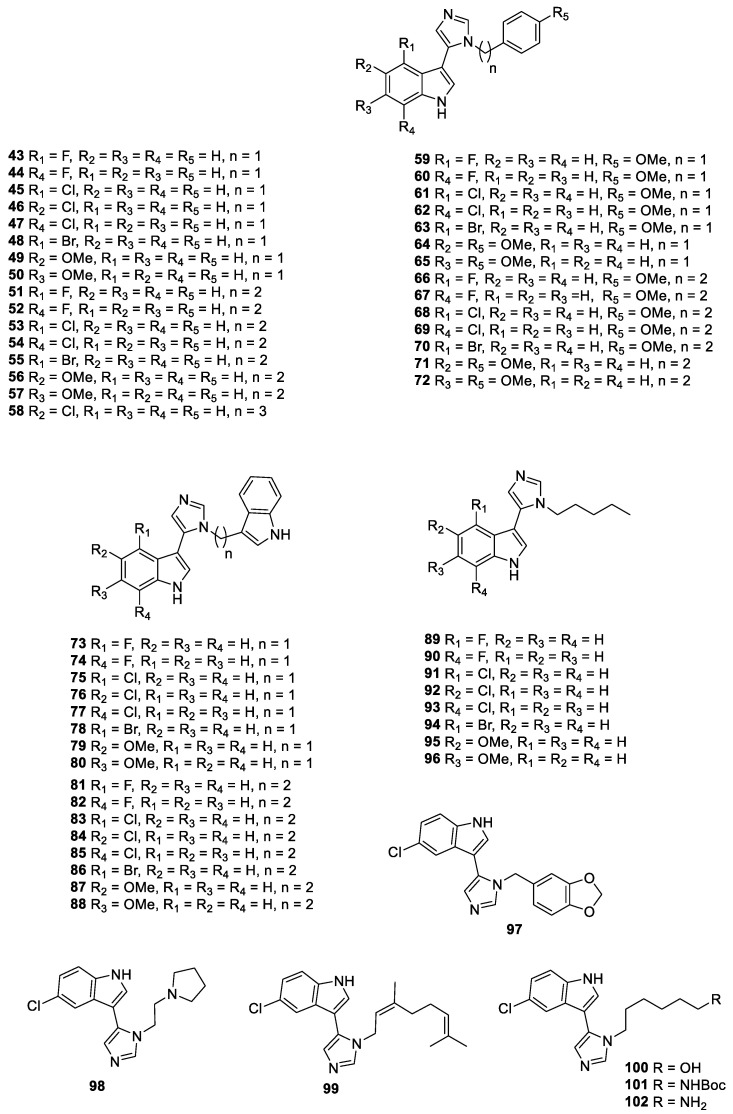
Structures of substituted indole-imidazoles **43**–**102**.

**Figure 4:**
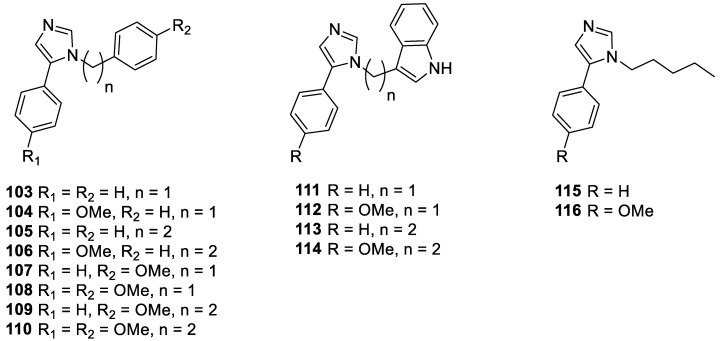
Structures of substituted 5-phenyl-1*H*-imidazoles **103**–**116**.

## Data Availability

Not applicable.

## Supplementary Materials

The following supporting information can be downloaded at: https://www.mdpi.com/article/10.3390/antibiotics11101450/s1, Figures S1–S116: NMR spectra of compounds **1**–**116**.
