# Supplementary material for: Discovery and Preliminary Structure-Activity Investigation of 3-Substituted-1H-imidazol-5-yl-1H-indoles with In Vitro Activity towards Methicillin-Resistant Staphylococcus aureus
Source: Antibiotics (Basel). 2022 Oct 21;11(10):1450. doi: 10.3390/antibiotics11101450 (PMC9598367; doi:10.3390/antibiotics11101450)

Supporting Information

Discovery and preliminary structure-activity investigation of 3-substituted-1*H*-imidazol-5-yl-1*H*-indoles with *in vitro* activity towards methicillin-resistant *Staphylococcus aureus*

Steven A. Li[1], Rebecca J. Zheng[1], Kenneth Sue[1], Marie-Lise Bourguet-Kondracki[2], Azza Troudi[3], Jean Michel Brunel[3], Brent R. Copp[1] and Melissa M. Cadelis[1,4,*]

[1] School of Chemical Sciences, The University of Auckland, Private Bag 92019, Auckland 1142, New Zealand

[2] Laboratoire Molécules de Communication et Adaptation des Micro-organismes, UMR 7245 CNRS, Muséum National d'Histoire Naturelle, 57 rue Cuvier (C.P. 54), 75005 Paris, France

[3] UMR MD1 "Membranes et Cibles Thérapeutiques", U1261 INSERM, Faculté de Pharmacie, Aix-Marseille Universite, 27 bd Jean Moulin, 13385 Marseille, France

[4] School of Medical Sciences, The University of Auckland, Private Bag 92019, Auckland 1142, New Zealand

*Correspondence: m.cadelis@auckland.ac.nz

Contents

Figure S1. 3-(1-Phenethyl-1*H*-imidazol-5-yl)-1*H*-indole (**1**)	**S4**
Figure S2. 6-Chloro-3-(1-(4-methoxyphenethyl)-1*H*-imidazol-5-yl)-1*H*-indole (**2**)	**S5**
Figure S3. 5-Fluoro-3-(1-phenethyl-1*H*-imidazol-5-yl)-1*H*-indole (**3**)	**S6**
Figure S4. 3-(1-(4-Iodobenzyl)-1*H*-imidazol-5-yl)-1*H*-indole (**4**)	**S7**
Figure S5. 5-Fluoro-3-(1-(4-iodobenzyl)-1*H*-imidazol-5-yl)-1*H*-indole (**5**)	**S8**
Figure S6. 6-Fluoro-3-(1-(4-iodobenzyl)-1*H*-imidazol-5-yl)-1*H*-indole (**6**)	**S9**
Figure S7. 5-Chloro-3-(1-(4-iodobenzyl)-1*H*-imidazol-5-yl)-1*H*-indole (**7**)	**S10**
Figure S8. 6-Chloro-3-(1-(4-iodobenzyl)-1*H*-imidazol-5-yl)-1*H*-indole (**8**)	**S11**
Figure S9. 5-Bromo-3-(1-(4-iodobenzyl)-1*H*-imidazol-5-yl)-1*H*-indole (**9**)	**S12**
Figure S10. 6-Bromo-3-(1-(4-iodobenzyl)-1*H*-imidazol-5-yl)-1*H*-indole (**10**)	**S13**
Figure S11. 3-(1-(4-Methoxybenzyl)-1*H*-imidazol-5-yl)-1*H*-indole (**11**)	**S14**
Figure S12. 5-Fluoro-3-(1-(4-methoxybenzyl)-1*H*-imidazol-5-yl)-1*H*-indole (**12**)	**S15**
Figure S13. 6-Fluoro-3-(1-(4-methoxybenzyl)-1*H*-imidazol-5-yl)-1*H*-indole (**13**)	**S16**
Figure S14. 5-Chloro-3-(1-(4-methoxybenzyl)-1*H*-imidazol-5-yl)-1*H*-indole (**14**)	**S17**
Figure S15. 6-Chloro-3-(1-(4-methoxybenzyl)-1*H*-imidazol-5-yl)-1*H*-indole (**15**)	**S18**
Figure S16. 5-Bromo-3-(1-(4-methoxybenzyl)-1*H*-imidazol-5-yl)-1*H*-indole (**16**)	**S19**
Figure S17. 6-Bromo-3-(1-(4-methoxybenzyl)-1*H*-imidazol-5-yl)-1*H*-indole (**17**)	**S20**
Figure S18. 6-Fluoro-3-(1-phenethyl-1*H*-imidazol-5-yl)-1*H*-indole (**18**)	**S21**
Figure S19. 5-Chloro-3-(1-phenethyl-1*H*-imidazol-5-yl)-1*H*-indole (**19**)	**S22**
Figure S20. 6-Chloro-3-(1-phenethyl-1*H*-imidazol-5-yl)-1*H*-indole (**20**)	**S23**
Figure S21. 5-Bromo-3-(1-phenethyl-1*H*-imidazol-5-yl)-1*H*-indole (**21**)	**S24**
Figure S22. 6-Bromo-3-(1-phenethyl-1*H*-imidazol-5-yl)-1*H*-indole (**22**)	**S25**
Figure S23. 3-(1-(4-Methoxyphenethyl)-1*H*-imidazol-5-yl)-1*H*-indole (**23**)	**S26**
Figure S24. 5-Fluoro-3-(1-(4-methoxyphenethyl)-1*H*-imidazol-5-yl)-1*H*-indole (**24**)	**S27**
Figure S25. 6-Fluoro-3-(1-(4-methoxyphenethyl)-1*H*-imidazol-5-yl)-1*H*-indole (**25**)	**S28**
Figure S26. 5-Chloro-3-(1-(4-methoxyphenethyl)-1*H*-imidazol-5-yl)-1*H*-indole (**26**)	**S29**
Figure S27. 5-Bromo-3-(1-(4-methoxyphenethyl)-1*H*-imidazol-5-yl)-1*H*-indole (**27**)	**S30**
Figure S28. 6-Bromo-3-(1-(4-methoxyphenethyl)-1*H*-imidazol-5-yl)-1*H*-indole (**28**)	**S31**
Figure S29. 3-(1-(2,5-Dimethoxyphenethyl)-1*H*-imidazol-5-yl)-1*H*-indole (**29**)	**S32**

Figure S30. 3-(1-(2,5-Dimethoxyphenethyl)-1*H*-imidazol-5-yl)-5-fluoro-1*H*-indole (**30**)	**S33**
Figure S31. 3-(1-(2,5-Dimethoxyphenethyl)-1*H*-imidazol-5-yl)-6-fluoro-1*H*-indole (**31**)	**S34**
Figure S32. 5-Chloro-3-(1-(2,5-dimethoxyphenethyl)-1*H*-imidazol-5-yl)-1*H*-indole (**32**)	**S35**
Figure S33. 6-Chloro-3-(1-(2,5-dimethoxyphenethyl)-1*H*-imidazol-5-yl)-1*H*-indole (**33**)	**S36**
Figure S34. 5-Bromo-3-(1-(2,5-dimethoxyphenethyl)-1*H*-imidazol-5-yl)-1*H*-indole (**34**)	**S37**
Figure S35. 6-Bromo-3-(1-(2,5-dimethoxyphenethyl)-1*H*-imidazol-5-yl)-1*H*-indole (**35**)	**S38**
Figure S36. 3-(1-(2-(Benzo[*d*][1,3]dioxol-5-yl)ethyl)-1*H*-imidazol-5-yl)-1*H*-indole (**36**)	**S39**
Figure S37. 3-(1-(2-(Benzo[*d*][1,3]dioxol-5-yl)ethyl)-1*H*-imidazol-5-yl)-5-fluoro-1*H*-indole (**37**)	**S40**
Figure S38. 3-(1-(2-(Benzo[*d*][1,3]dioxol-5-yl)ethyl)-1*H*-imidazol-5-yl)-6-fluoro-1*H*-indole (**38**)	**S41**
Figure S39. 3-(1-(2-(Benzo[*d*][1,3]dioxol-5-yl)ethyl)-1*H*-imidazol-5-yl)-5-chloro-1*H*-indole (**39**)	**S42**
Figure S40. 3-(1-(2-(Benzo[*d*][1,3]dioxol-5-yl)ethyl)-1*H*-imidazol-5-yl)-6-chloro-1*H*-indole (**40**)	**S43**
Figure S41. 3-(1-(2-(Benzo[*d*][1,3]dioxol-5-yl)ethyl)-1*H*-imidazol-5-yl)-5-bromo-1*H*-indole (**41**)	**S44**
Figure S42. 3-(1-(2-(Benzo[*d*][1,3]dioxol-5-yl)ethyl)-1*H*-imidazol-5-yl)-6-bromo-1*H*-indole (**42**)	**S45**
**Figure S43.** 3-(1-Benzyl-1*H*-imidazol-5-yl)-4-fluoro-1*H*-indole (**43**)	**S46**
Figure S44. 3-(1-Benzyl-1*H*-imidazol-5-yl)-7-fluoro-1*H*-indole (**44**)	**S47**
Figure S45. 3-(1-Benzyl-1*H*-imidazol-5-yl)-4-chloro-1*H*-indole (**45**)	**S48**
Figure S46. 3-(1-Benzyl-1*H*-imidazol-5-yl)-5-chloro-1*H*-indole (**46**)	**S49**
Figure S47. 3-(1-Benzyl-1*H*-imidazol-5-yl)-7-chloro-1*H*-indole (**47**)	**S50**
Figure S48. 3-(1-Benzyl-1*H*-imidazol-5-yl)-4-bromo-1*H*-indole (**48**)	**S51**
Figure S49. 3-(1-Benzyl-1*H*-imidazol-5-yl)-5-methoxy-1*H*-indole (**49**)	**S52**
Figure S50. 3-(1-Benzyl-1*H*-imidazol-5-yl)-6-methoxy-1*H*-indole (**50**)	**S53**
Figure S51. 4-Fluoro-3-(1-phenethyl-1*H*-imidazol-5-yl)-1*H*-indole (**51**)	**S54**
Figure S52. 7-Fluoro-3-(1-phenethyl-1*H*-imidazol-5-yl)-1*H*-indole (**52**)	**S55**
Figure S53. 4-Chloro-3-(1-phenethyl-1*H*-imidazol-5-yl)-1*H*-indole (**53**)	**S56**
Figure S54. 7-Chloro-3-(1-phenethyl-1*H*-imidazol-5-yl)-1*H*-indole (**54**)	**S57**
Figure S55. 4-Bromo-3-(1-phenethyl-1*H*-imidazol-5-yl)-1*H*-indole (**55**)	**S58**
Figure S56. 5-Methoxy-3-(1-phenethyl-1*H*-imidazol-5-yl)-1*H*-indole (**56**)	**S59**
Figure S57. 6-Methoxy-3-(1-phenethyl-1*H*-imidazol-5-yl)-1*H*-indole (**57**)	**S60**
Figure S58. 5-Chloro-3-(1-(3-phenylpropyl)-1*H*-imidazol-5-yl)-1*H*-indole (**58**)	**S61**
Figure S59. 4-Fluoro-3-(1-(4-methoxybenzyl)-1*H*-imidazol-5-yl)-1*H*-indole (**59**)	**S62**
Figure S60. 7-Fluoro-3-(1-(4-methoxybenzyl)-1*H*-imidazol-5-yl)-1*H*-indole (**60**)	**S63**
Figure S61. 4-Chloro-3-(1-(4-methoxybenzyl)-1*H*-imidazol-5-yl)-1*H*-indole (**61**)	**S64**
Figure S62. 7-Chloro-3-(1-(4-methoxybenzyl)-1*H*-imidazol-5-yl)-1*H*-indole (**62**)	**S65**
Figure S63. 4-Bromo-3-(1-(4-methoxybenzyl)-1*H*-imidazol-5-yl)-1*H*-indole (**63**)	**S66**
Figure S64. 5-Methoxy-3-(1-(4-methoxybenzyl)-1*H*-imidazol-5-yl)-1*H*-indole (**64**)	**S67**
Figure S65. 6-Methoxy-3-(1-(4-methoxybenzyl)-1*H*-imidazol-5-yl)-1*H*-indole (**65**)	**S68**
Figure S66. 4-Fluoro-3-(1-(4-methoxyphenethyl)-1*H*-imidazol-5-yl)-1*H*-indole (**66**)	**S69**
Figure S67. 7-Fluoro-3-(1-(4-methoxyphenethyl)-1*H*-imidazol-5-yl)-1*H*-indole (**67**)	**S70**
Figure S68. 4-Chloro-3-(1-(4-methoxyphenethyl)-1*H*-imidazol-5-yl)-1*H*-indole (**68**)	**S71**
Figure S69. 7-Chloro-3-(1-(4-methoxyphenethyl)-1*H*-imidazol-5-yl)-1*H*-indole (**69**)	**S72**
Figure S70. 4-Bromo-3-(1-(4-methoxyphenethyl)-1*H*-imidazol-5-yl)-1*H*-indole (**70**)	**S73**
Figure S71. 5-Methoxy-3-(1-(4-methoxyphenethyl)-1*H*-imidazol-5-yl)-1*H*-indole (**71**)	**S74**
Figure S72. 6-Methoxy-3-(1-(4-methoxyphenethyl)-1*H*-imidazol-5-yl)-1*H*-indole (**72**)	**S75**
Figure S73. 3-(1-((1*H*-Indol-3-yl)methyl)-1*H*-imidazol-5-yl)-4-fluoro-1*H*-indole (**73**)	**S76**

Figure S74. 3-(1-((1*H*-Indol-3-yl)methyl)-1*H*-imidazol-5-yl)-7-fluoro-1*H*-indole (**74**)	**S77**
Figure S75. 3-(1-((1*H*-Indol-3-yl)methyl)-1*H*-imidazol-5-yl)-4-chloro-1*H*-indole (**75**)	**S78**
Figure S76. 3-(1-((1*H*-Indol-3-yl)methyl)-1*H*-imidazol-5-yl)-5-chloro-1*H*-indole (**76**)	**S79**
Figure S77. 3-(1-((1*H*-Indol-3-yl)methyl)-1*H*-imidazol-5-yl)-7-chloro-1*H*-indole (**77**)	**S80**
Figure S78. 3-(1-((1*H*-Indol-3-yl)methyl)-1*H*-imidazol-5-yl)-4-bromo-1*H*-indole (**78**)	**S81**
Figure S79. 3-(1-((1*H*-Indol-3-yl)methyl)-1*H*-imidazol-5-yl)-5-methoxy-1*H*-indole (**79**)	**S82**
Figure S80. 3-(1-((1*H*-Indol-3-yl)methyl)-1*H*-imidazol-5-yl)-6-methoxy-1*H*-indole (**80**)	**S83**
Figure S81. 3-(1-(2-(1*H*-Indol-3-yl)ethyl)-1*H*-imidazol-5-yl)-4-fluoro-1*H*-indole (**81**)	**S84**
Figure S82. 3-(1-(2-(1*H*-Indol-3-yl)ethyl)-1*H*-imidazol-5-yl)-7-fluoro-1*H*-indole (**82**)	**S85**
Figure S83. 3-(1-(2-(1*H*-Indol-3-yl)ethyl)-1*H*-imidazol-5-yl)-4-chloro-1*H*-indole (**83**)	**S86**
Figure S84. 3-(1-(2-(1*H*-Indol-3-yl)ethyl)-1*H*-imidazol-5-yl)-5-chloro-1*H*-indole (**84**)	**S87**
Figure S85. 3-(1-(2-(1*H*-Indol-3-yl)ethyl)-1*H*-imidazol-5-yl)-7-chloro-1*H*-indole (**85**)	**S88**
Figure S86. 3-(1-(2-(1*H*-Indol-3-yl)ethyl)-1*H*-imidazol-5-yl)-4-bromo-1*H*-indole (**86**)	**S89**
Figure S87. 3-(1-(2-(1*H*-Indol-3-yl)ethyl)-1*H*-imidazol-5-yl)-5-methoxy-1*H*-indole (**87**)	**S90**
Figure S88. 3-(1-(2-(1*H*-Indol-3-yl)ethyl)-1*H*-imidazol-5-yl)-6-methoxy-1*H*-indole (**88**)	**S91**
Figure S89. 4-Fluoro-3-(1-pentyl-1*H*-imidazol-5-yl)-1*H*-indole (**89**)	**S92**
Figure S90. 7-Fluoro-3-(1-pentyl-1*H*-imidazol-5-yl)-1*H*-indole (**90**)	**S93**
Figure S91. 4-Chloro-3-(1-pentyl-1*H*-imidazol-5-yl)-1*H*-indole (**91**)	**S94**
Figure S92. 5-Chloro-3-(1-pentyl-1*H*-imidazol-5-yl)-1*H*-indole (**92**)	**S95**
Figure S93. 7-Chloro-3-(1-pentyl-1*H*-imidazol-5-yl)-1*H*-indole (**93**)	**S96**
Figure S94. 4-Bromo-3-(1-pentyl-1*H*-imidazol-5-yl)-1*H*-indole (**94**)	**S97**
Figure S95. 5-Methoxy-3-(1-pentyl-1*H*-imidazol-5-yl)-1*H*-indole (**95**)	**S98**
Figure S96. 6-Methoxy-3-(1-pentyl-1*H*-imidazol-5-yl)-1*H*-indole (**96**)	**S99**
Figure S97. 3-(1-(Benzo[*d*][1,3]dioxol-5-ylmethyl)-1*H*-imidazol-5-yl)-5-chloro-1*H*-indole (**97**)	**S100**
Figure S98. 5-Chloro-3-(1-(2-(pyrrolidin-1-yl)ethyl)-1*H*-imidazol-5-yl)-1*H*-indole (**98**)	**S101**
Figure S99. (*E*)-5-Chloro-3-(1-(3,7-dimethylocta-2,6-dien-1-yl)-1*H*-imidazol-5-yl)-1*H*-indole (**99**)	**S102**
Figure S100. 6-(5-(5-Chloro-1*H*-indol-3-yl)-1*H*-imidazol-1-yl)hexan-1-ol (**100**)	**S103**
Figure S101. *tert*-Butyl (6-(5-(5-chloro-1*H*-indol-3-yl)-1*H*-imidazol-1-yl)hexyl)carbamate (**101**)	**S104**
Figure S102. 6-(5-(5-Chloro-1*H*-indol-3-yl)-1*H*-imidazol-1-yl)hexan-1-aminium 2,2,2-trifluoroacetate (**102**)	**S105**
Figure S103. 1-Benzyl-5-phenyl-1*H*-imidazole (**103**)	**S106**
Figure S104. 1-Benzyl-5-(4-methoxyphenyl)-1*H*-imidazole (**104**)	**S107**
Figure S105. 1-Phenethyl-5-phenyl-1*H*-imidazole (**105**)	**S108**
Figure S106. 5-(4-Methoxyphenyl)-1-phenethyl-1*H*-imidazole (**106**)	**S109**
Figure S107. 1-(4-Methoxybenzyl)-5-phenyl-1*H*-imidazole (**107**)	**S110**
Figure S108. 1-(4-Methoxybenzyl)-5-(4-methoxyphenyl)-1*H*-imidazole (**108**)	**S111**
Figure S109. 1-(4-Methoxyphenethyl)-5-phenyl-1*H*-imidazole (**109**)	**S112**
Figure S110. 1-(4-Methoxyphenethyl)-5-(4-methoxyphenyl)-1*H*-imidazole (**110**)	**S113**
Figure S111. 3-((5-Phenyl-1*H*-imidazol-1-yl)methyl)-1*H*-indole (**111**)	**S114**
Figure S112. 3-((5-(4-Methoxyphenyl)-1*H*-imidazol-1-yl)methyl)-1*H*-indole (**112**)	**S115**
Figure S113. 3-(2-(5-Phenyl-1*H*-imidazol-1-yl)ethyl)-1*H*-indole (**113**)	**S116**
Figure S114. 3-(2-(5-(4-Methoxyphenyl)-1*H*-imidazol-1-yl)ethyl)-1*H*-indole (**114**)	**S117**
Figure S115. 1-Pentyl-5-phenyl-1*H*-imidazole (**115**)	**S118**
Figure S116. 5-(4-Methoxyphenyl)-1-pentyl-1*H*-imidazole (**116**)	**S119**

Figure S1. 3-(1-Phenethyl-1*H*-imidazol-5-yl)-1*H*-indole (**1**)

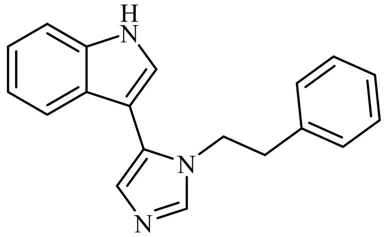

^{1}H NMR (DMSO-d_6, 400 MHz):

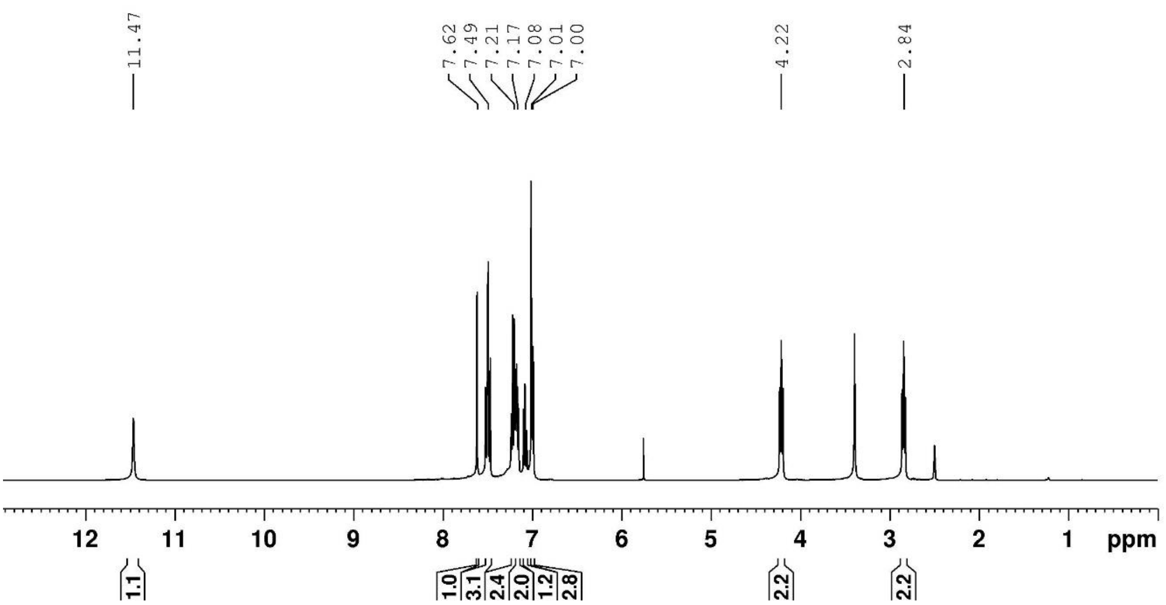

^{13}C NMR (DMSO-d_6, 100 MHz):

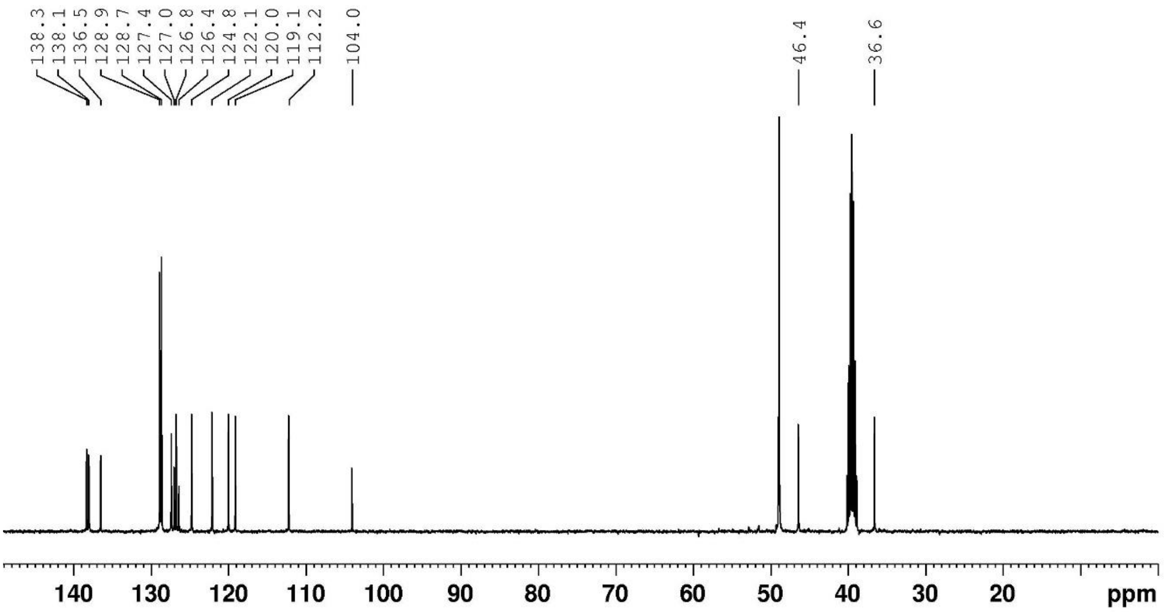

Figure S2. 6-Chloro-3-(1-(4-methoxyphenethyl)-1*H*-imidazol-5-yl)-1*H*-indole (**2**)

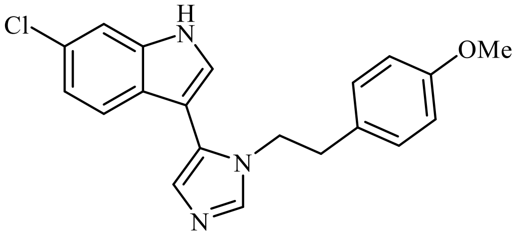

^{1}H NMR (DMSO-d_6, 400 MHz):

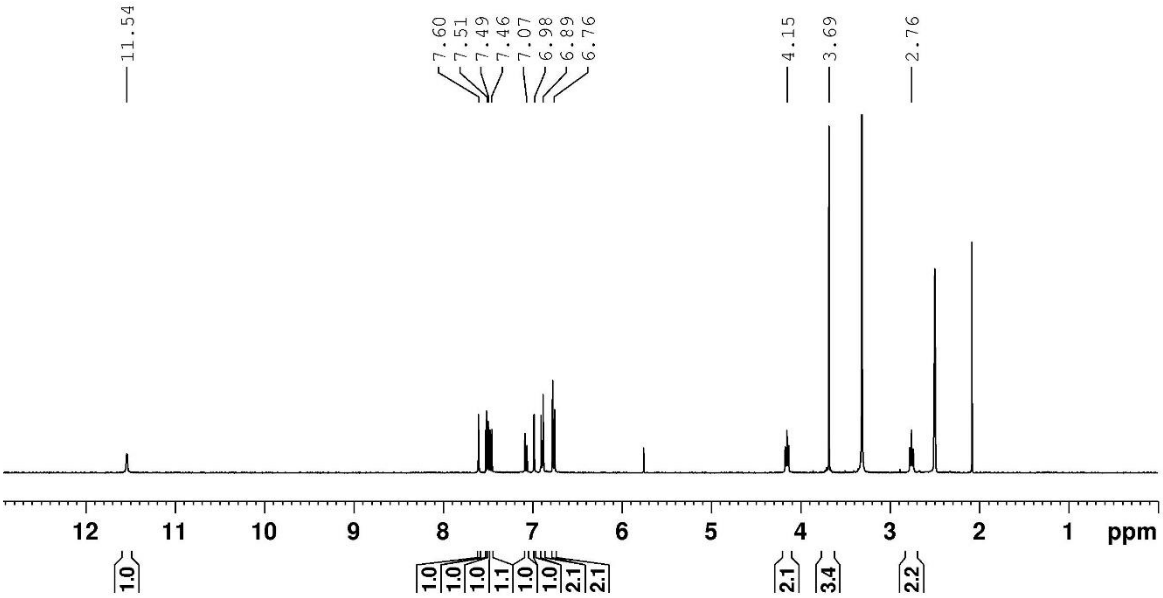

^{13}C NMR (DMSO-d_6, 100 MHz):

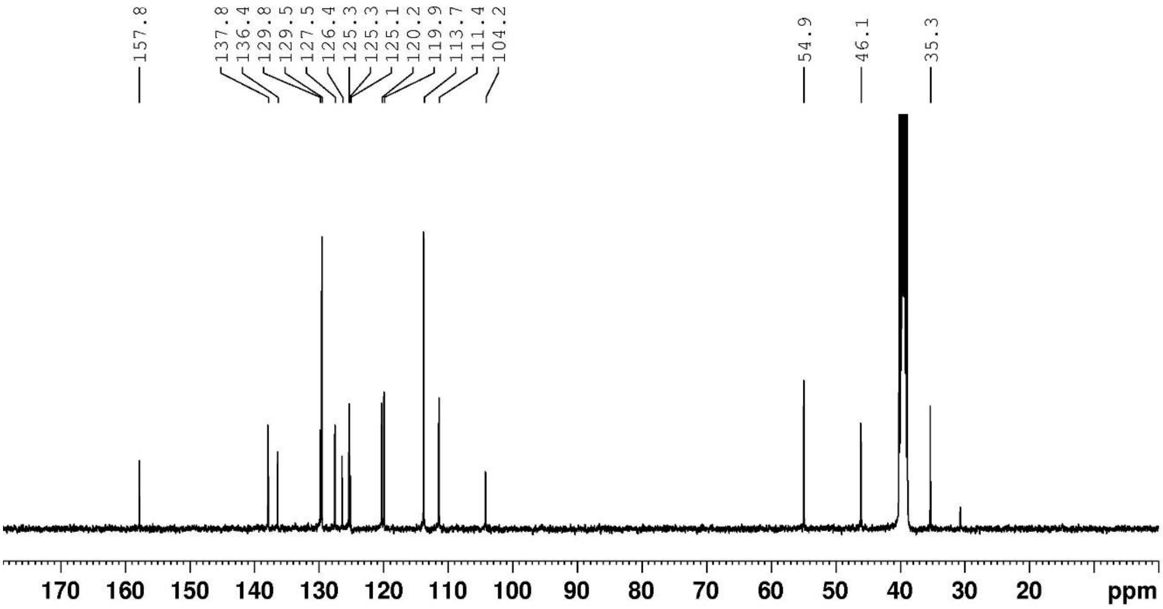

Figure S3. 5-Fluoro-3-(1-phenethyl-1*H*-imidazol-5-yl)-1*H*-indole (**3**)

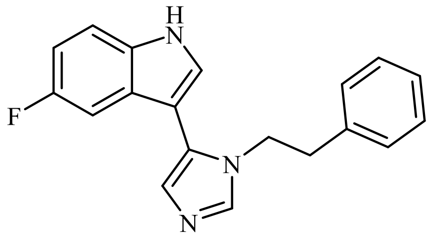

^{1}H NMR (DMSO-d_6, 400 MHz):

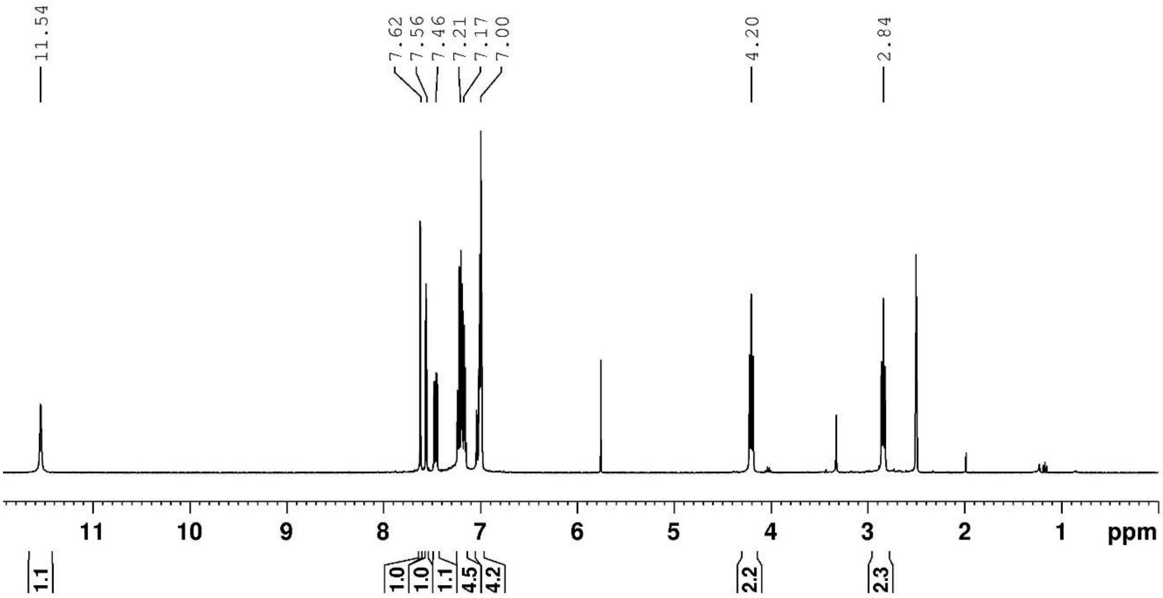

^{13}C NMR (DMSO-d_6, 100 MHz):

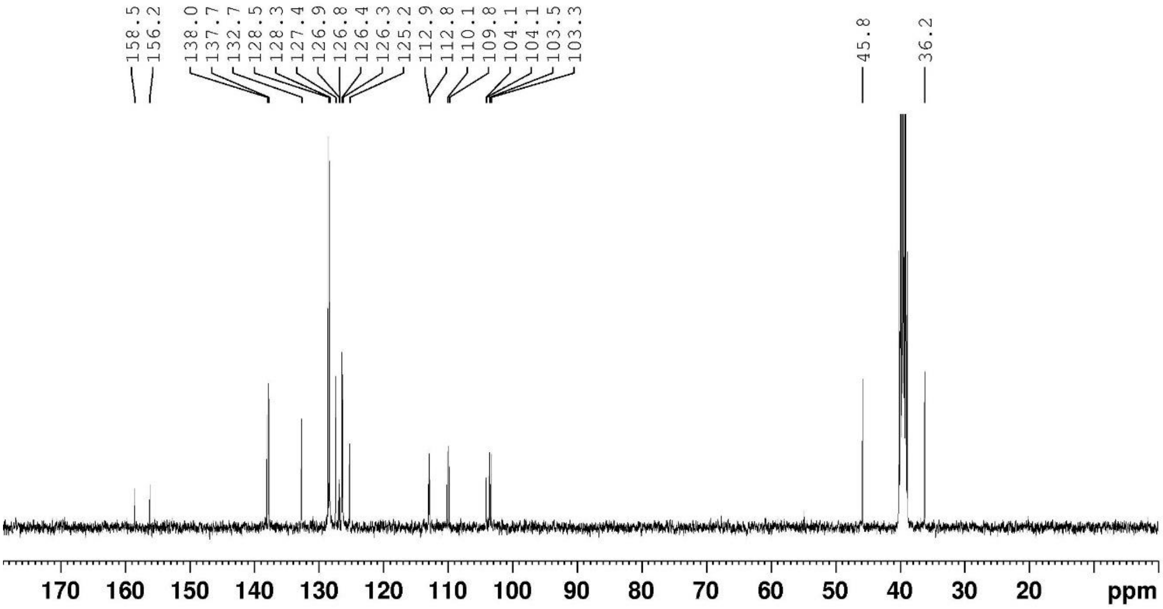

Figure S4. 3-(1-(4-Iodobenzyl)-1*H*-imidazol-5-yl)-1*H*-indole (**4**)

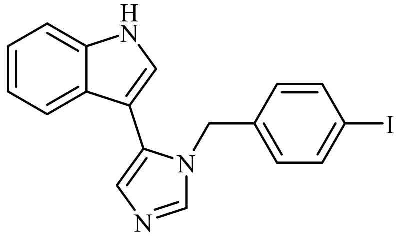

^{1}H NMR (DMSO-d_6, 400 MHz):

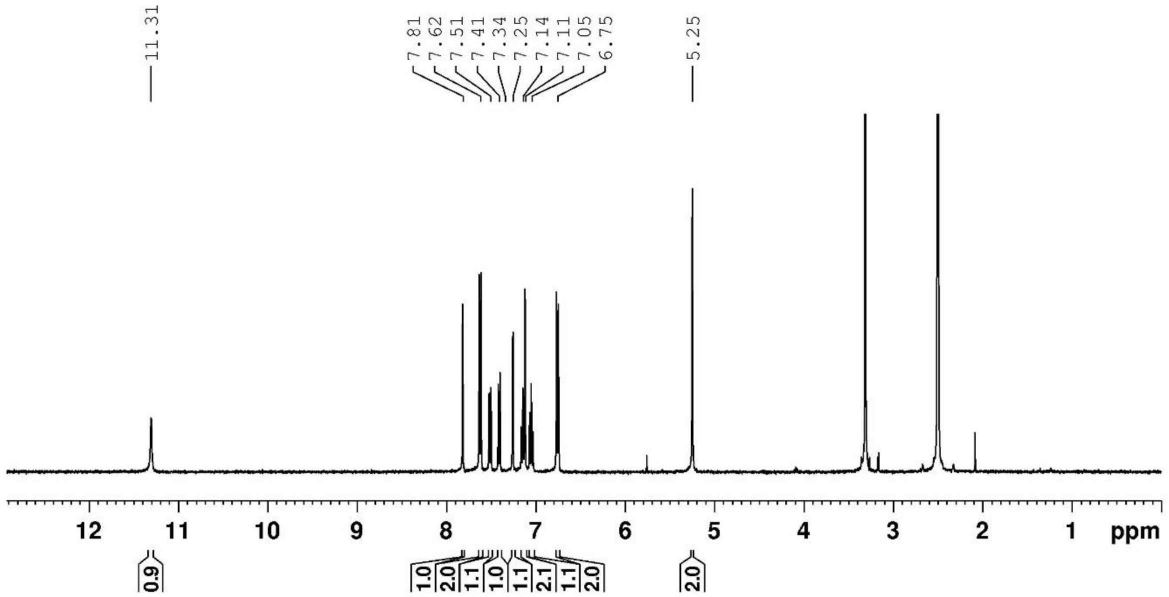

^{13}C NMR (DMSO-d_6, 100 MHz):

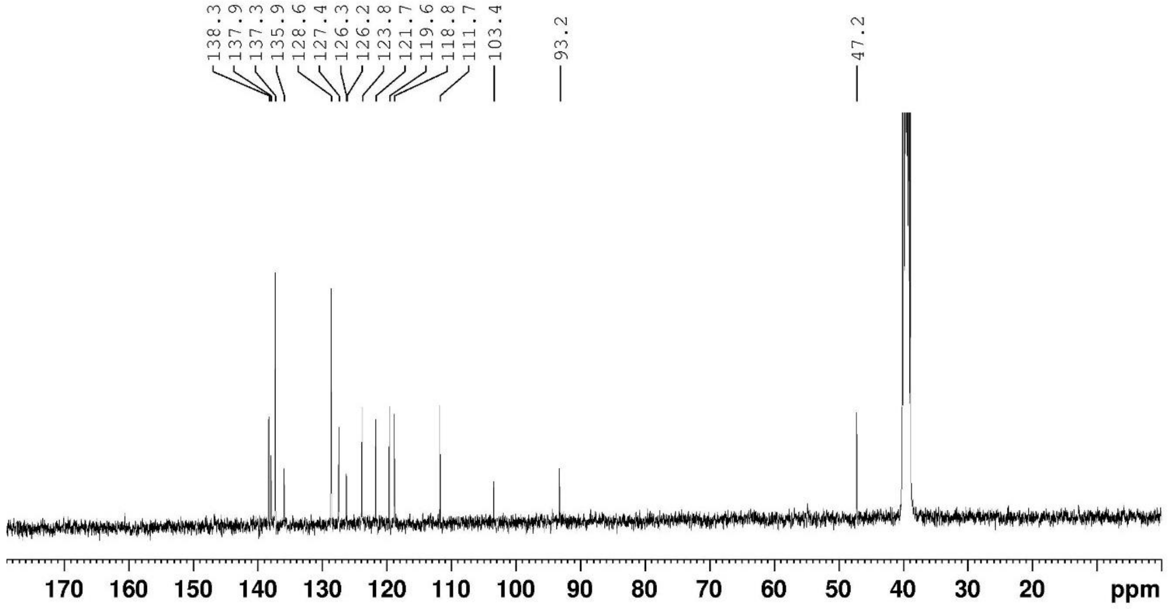

Figure S5. 5-Fluoro-3-(1-(4-iodobenzyl)-1*H*-imidazol-5-yl)-1*H*-indole (**5**)

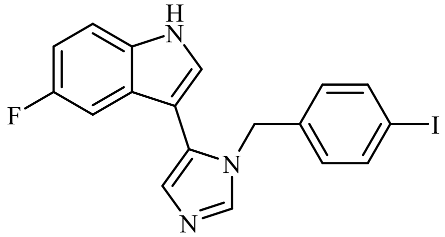

^{1}H NMR (DMSO-d_6, 400 MHz):

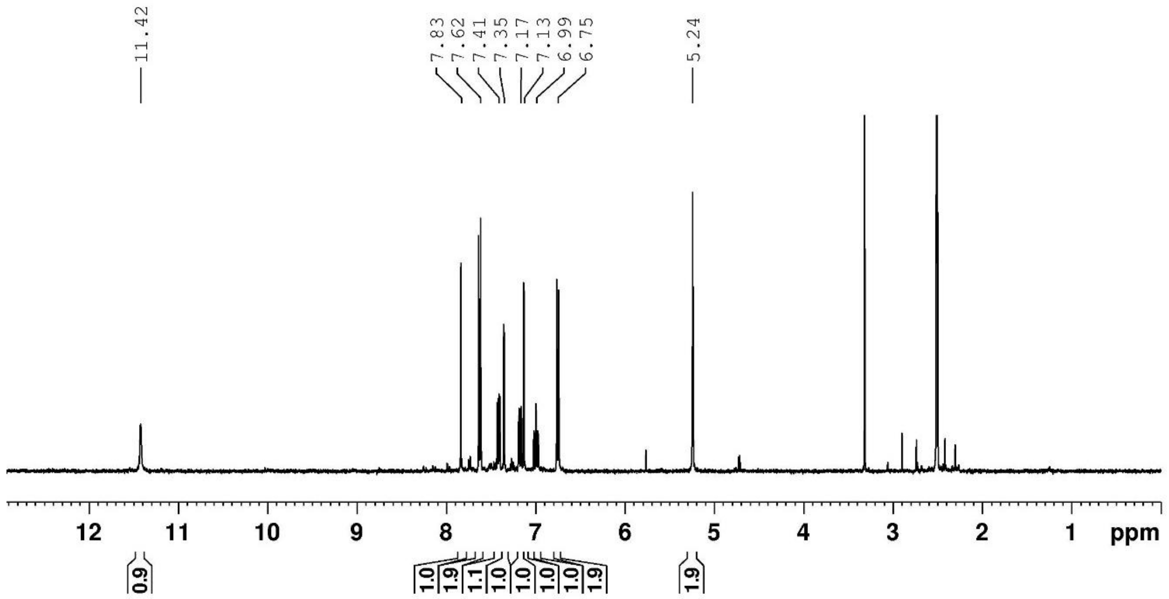

^{13}C NMR (DMSO-d_6, 100 MHz):

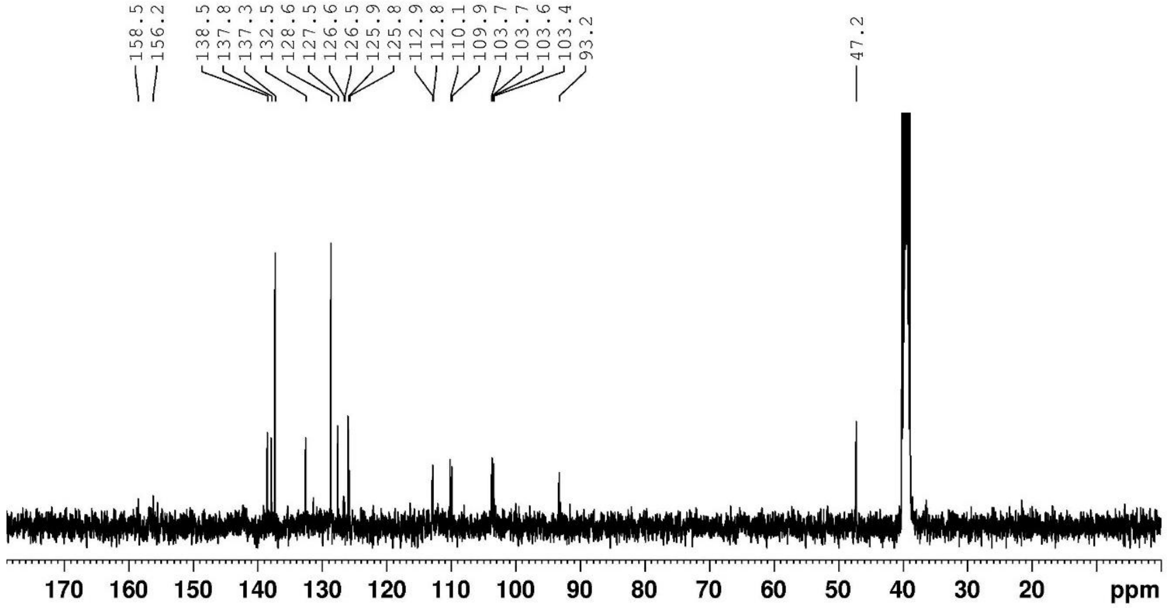

Figure S6. 6-Fluoro-3-(1-(4-iodobenzyl)-1*H*-imidazol-5-yl)-1*H*-indole (**6**)

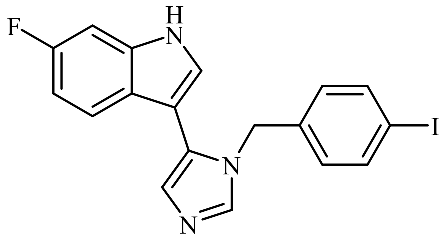

^{1}H NMR (DMSO-d_6, 400 MHz):

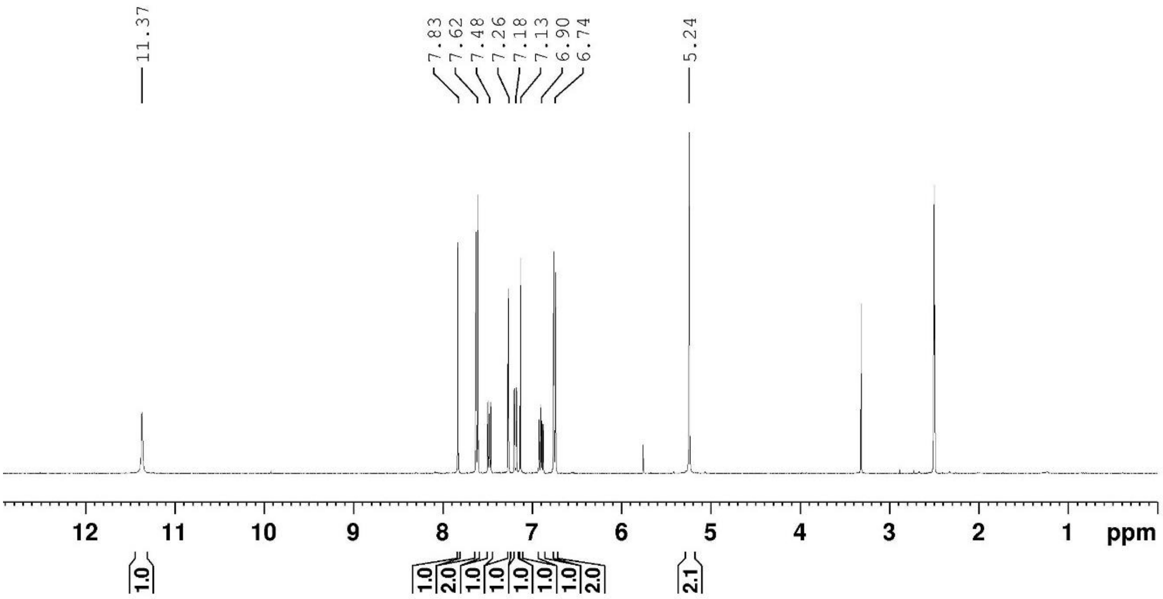

^{13}C NMR (DMSO-d_6, 100 MHz):

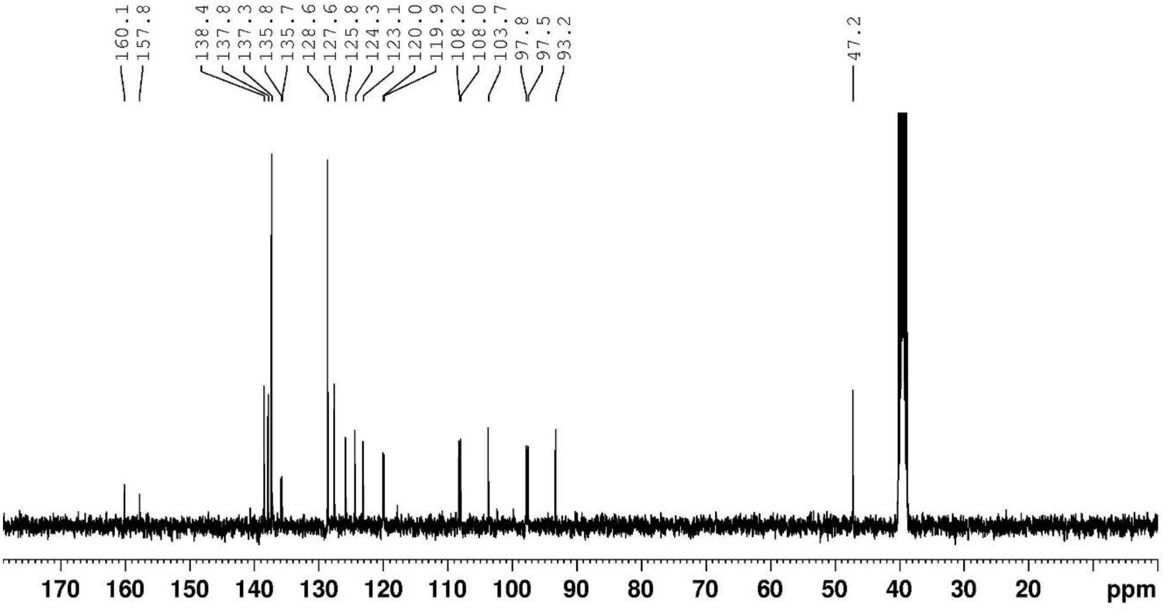

Figure S7. 5-Chloro-3-(1-(4-iodobenzyl)-1*H*-imidazol-5-yl)-1*H*-indole (**7**)

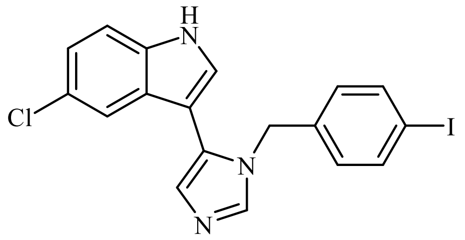

^{1}H NMR (DMSO-d_6, 400 MHz):

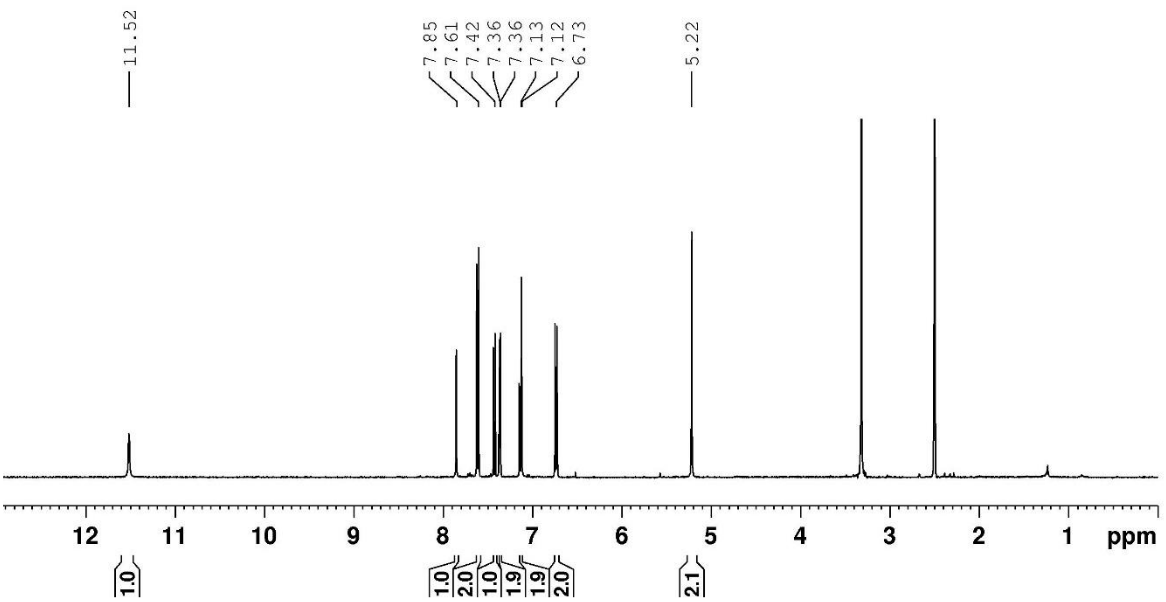

^{13}C NMR (DMSO-d_6, 100 MHz):

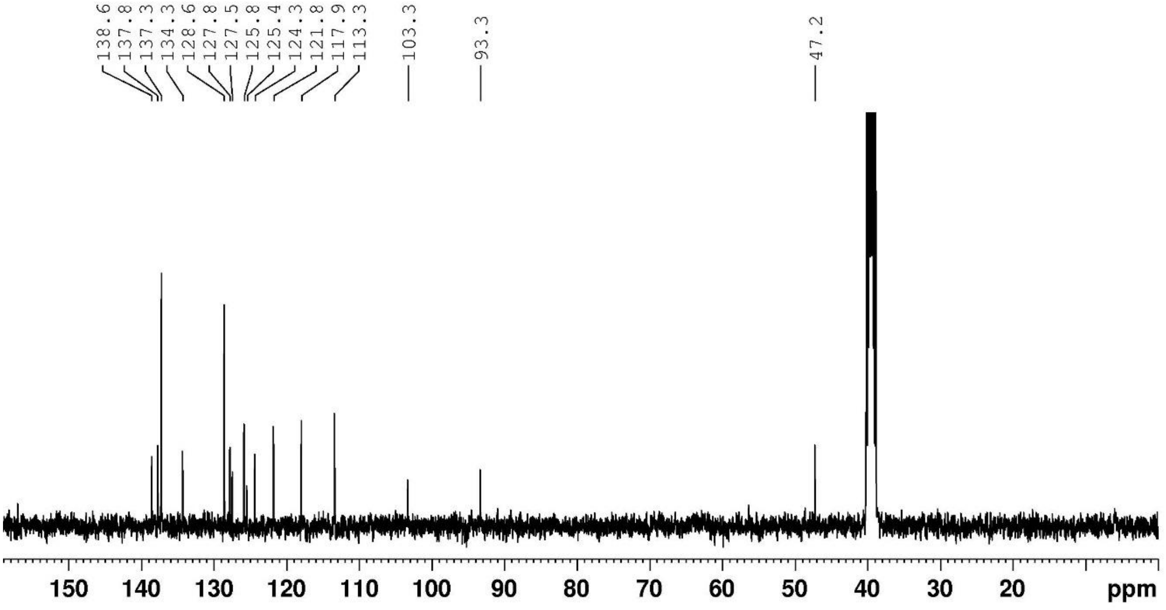

Figure S8. 6-Chloro-3-(1-(4-iodobenzyl)-1*H*-imidazol-5-yl)-1*H*-indole (**8**)

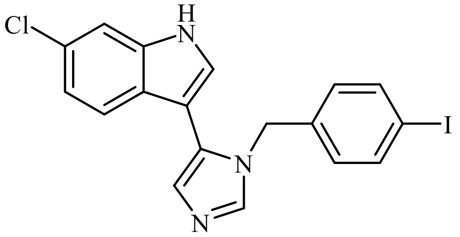

^{1}H NMR (DMSO-d_6, 400 MHz):

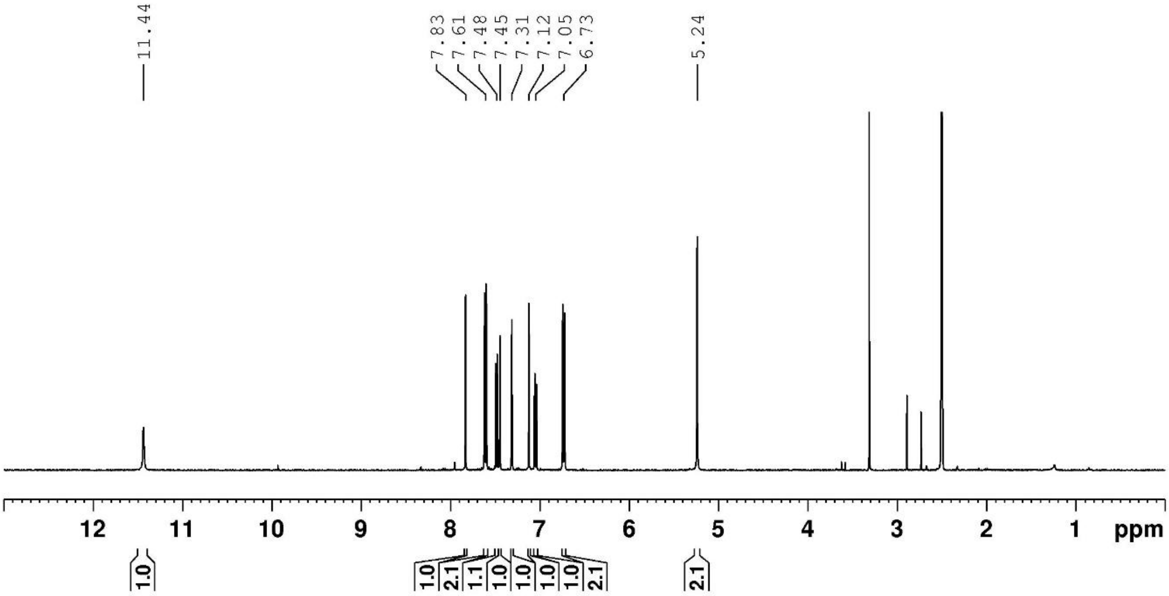

^{13}C NMR (DMSO-d_6, 100 MHz):

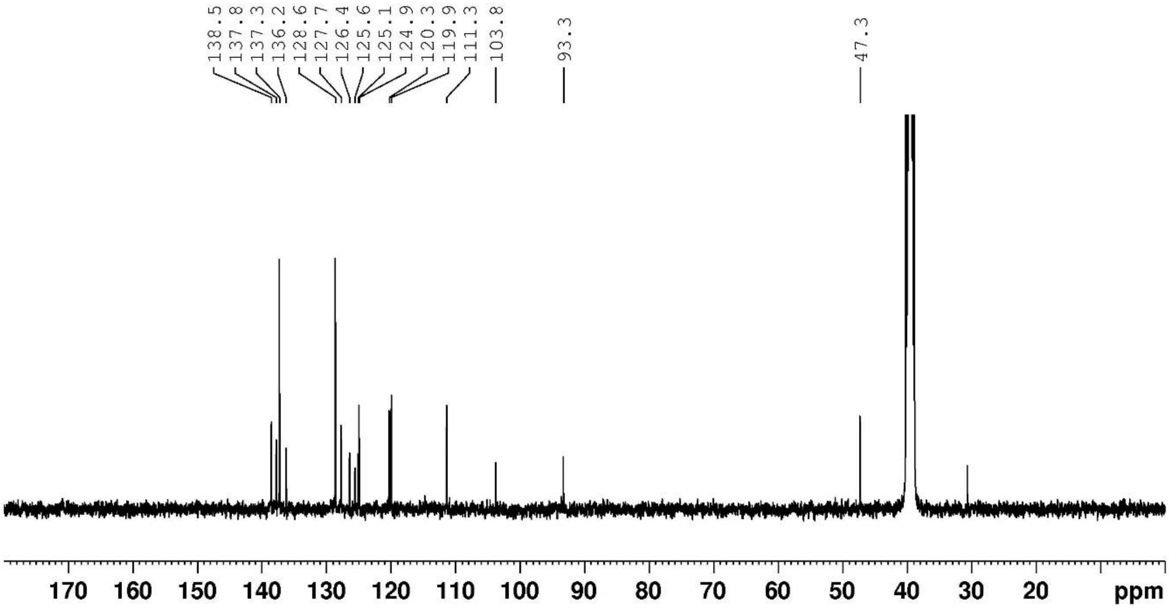

Figure S9. 5-Bromo-3-(1-(4-iodobenzyl)-1*H*-imidazol-5-yl)-1*H*-indole (**9**)

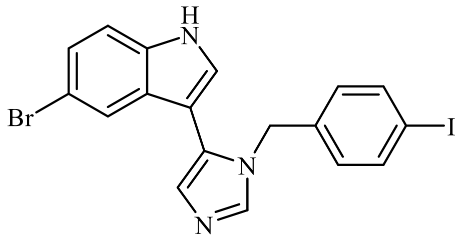

^{1}H NMR (DMSO-d_6, 400 MHz):

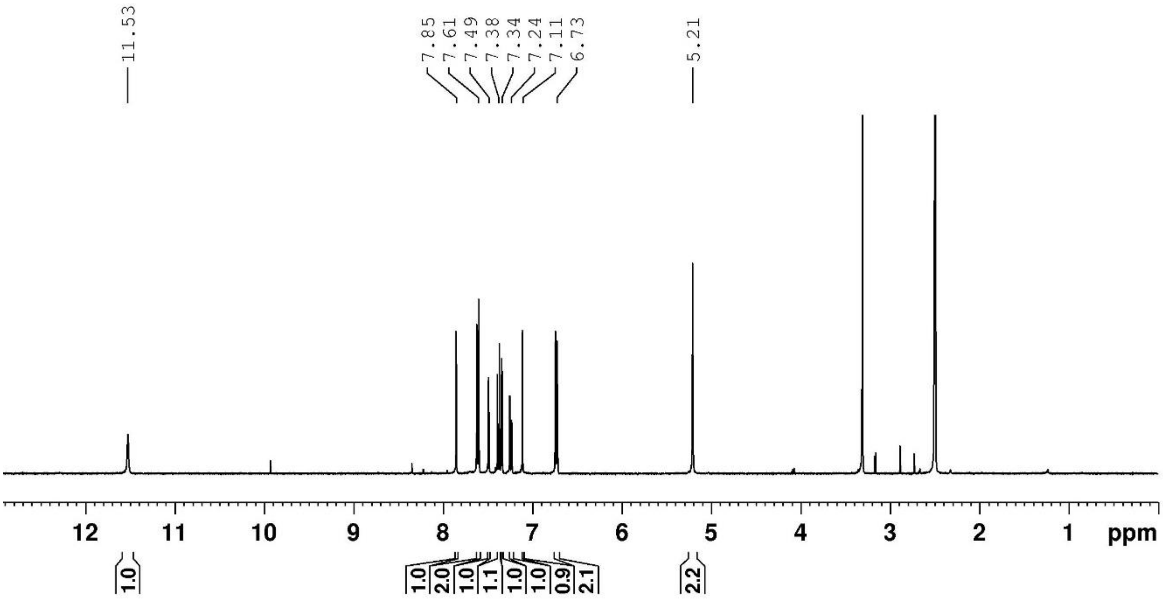

^{13}C NMR (DMSO-d_6, 100 MHz):

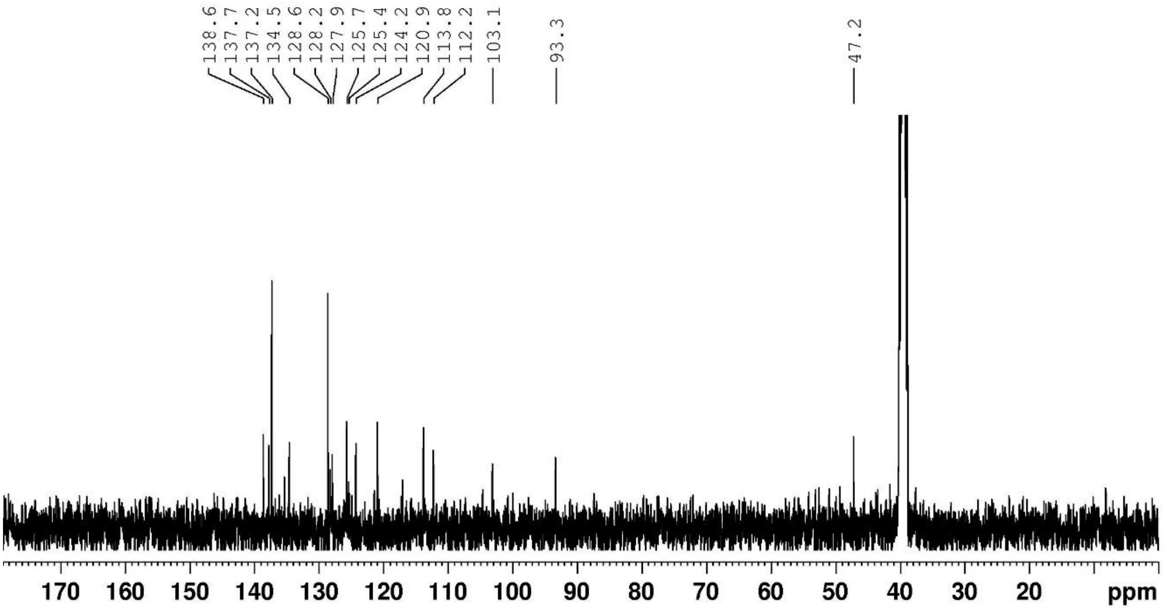

Figure S10. 6-Bromo-3-(1-(4-iodobenzyl)-1*H*-imidazol-5-yl)-1*H*-indole (**10**)

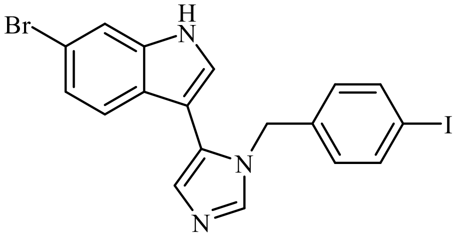

^{1}H NMR (DMSO-d_6, 400 MHz):

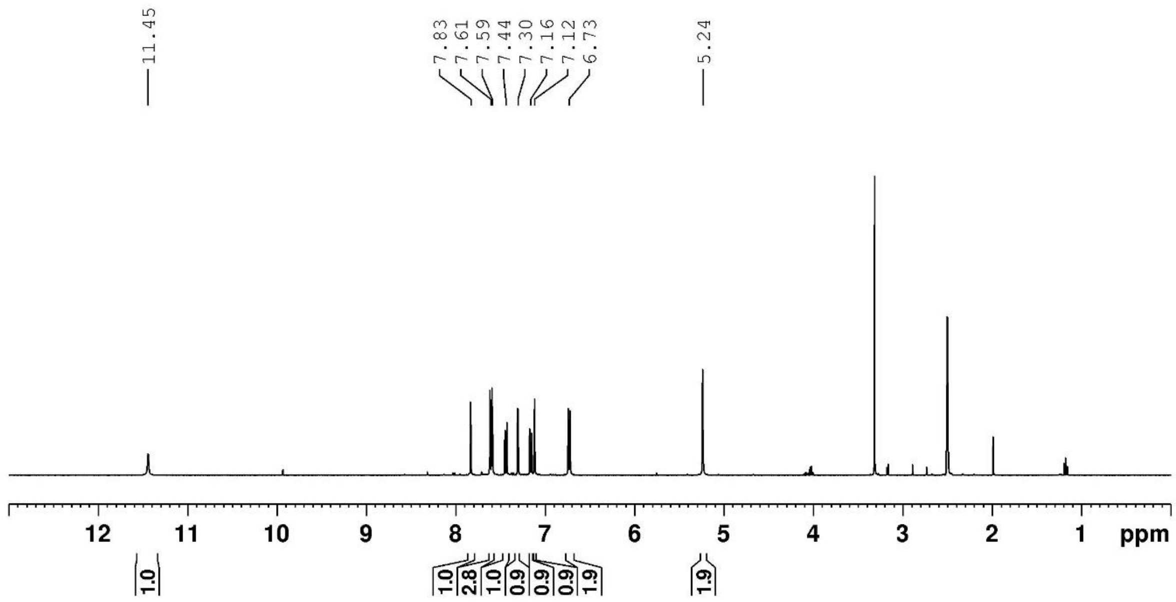

^{13}C NMR (DMSO-d_6, 100 MHz):

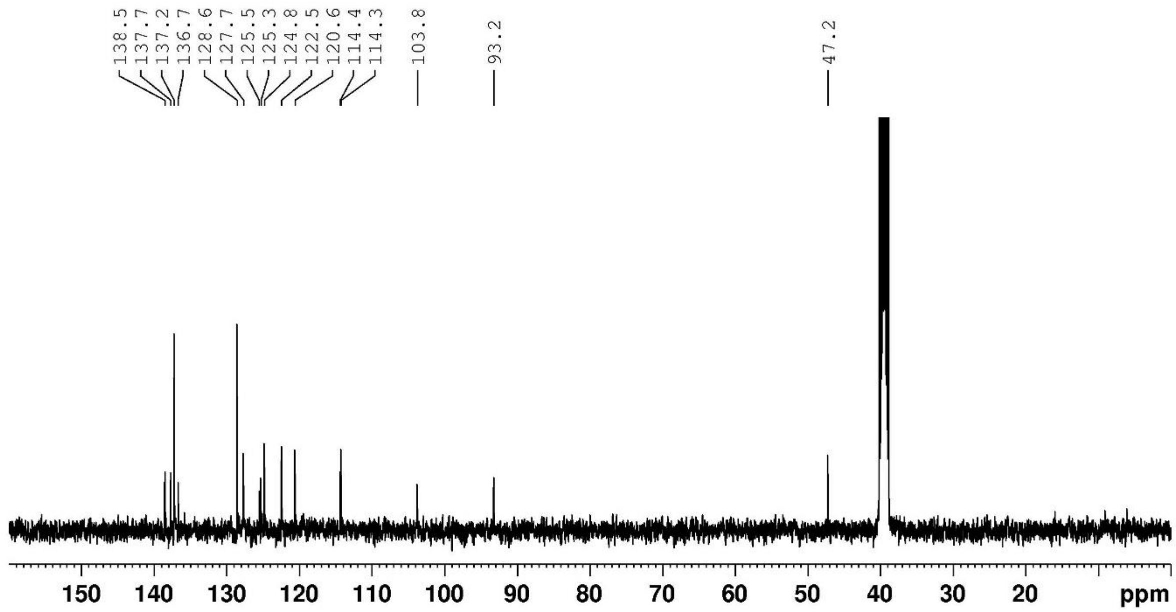

Figure S11. 3-(1-(4-Methoxybenzyl)-1*H*-imidazol-5-yl)-1*H*-indole (**11**)

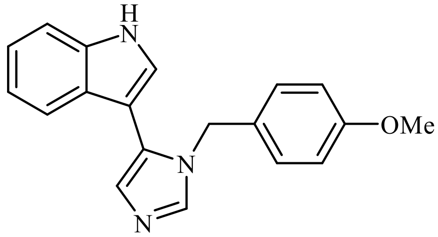

^{1}H NMR (DMSO-d_6, 400 MHz):

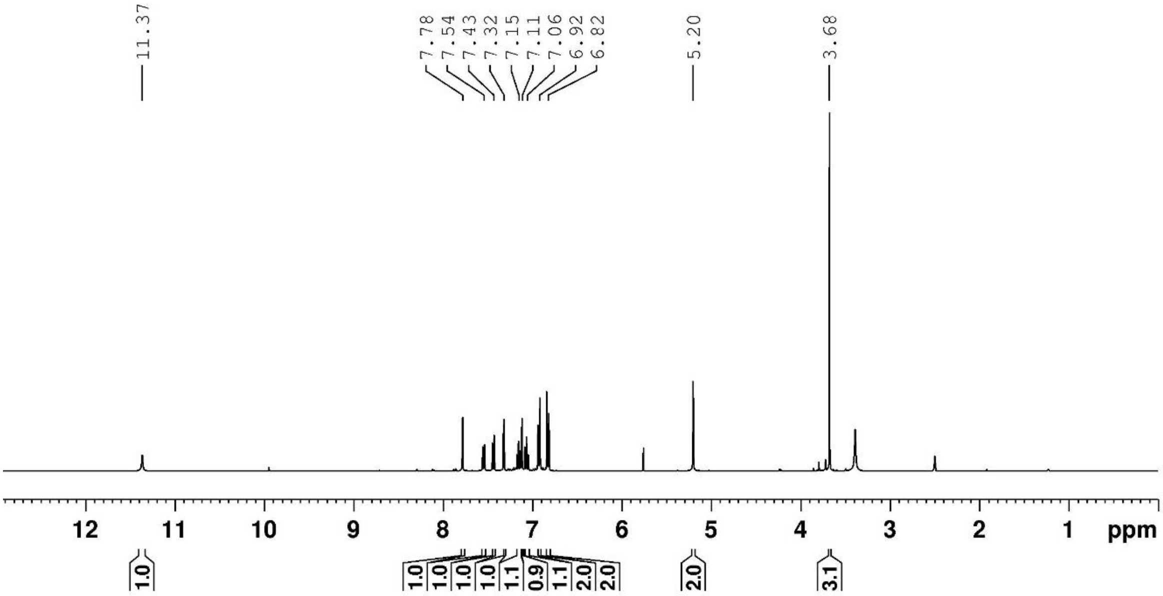

^{13}C NMR (DMSO-d_6, 100 MHz):

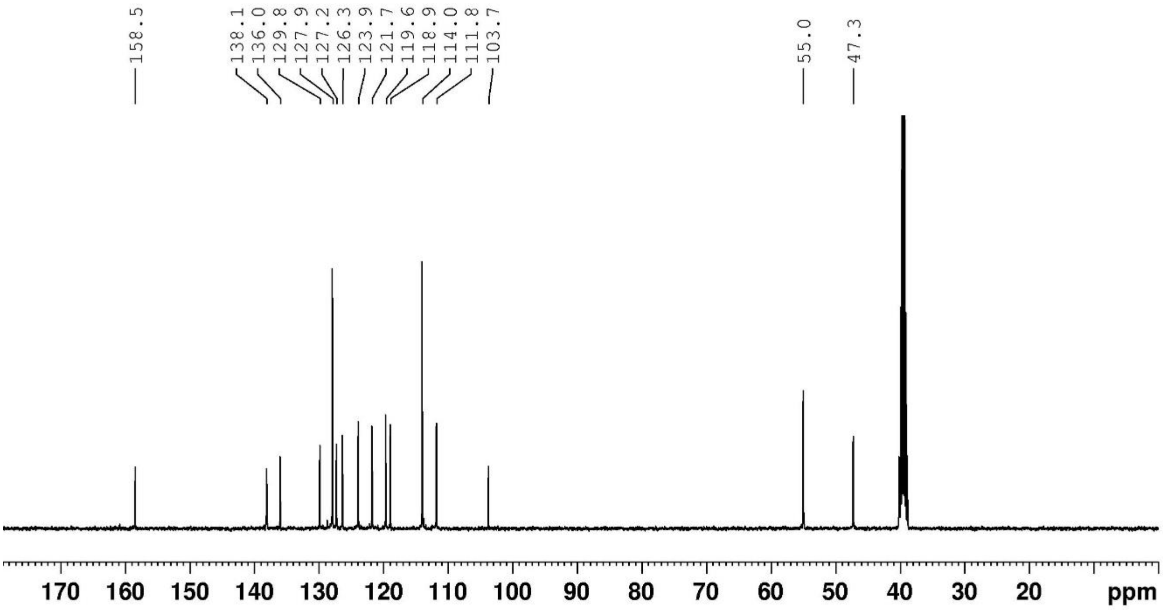

Figure S12. 5-Fluoro-3-(1-(4-methoxybenzyl)-1*H*-imidazol-5-yl)-1*H*-indole (**12**)

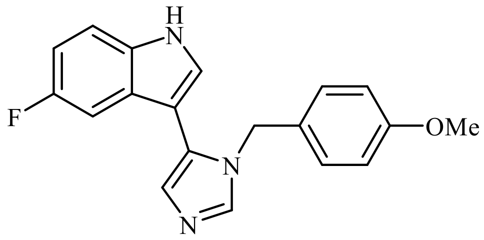

^{1}H NMR (DMSO-d_6, 400 MHz):

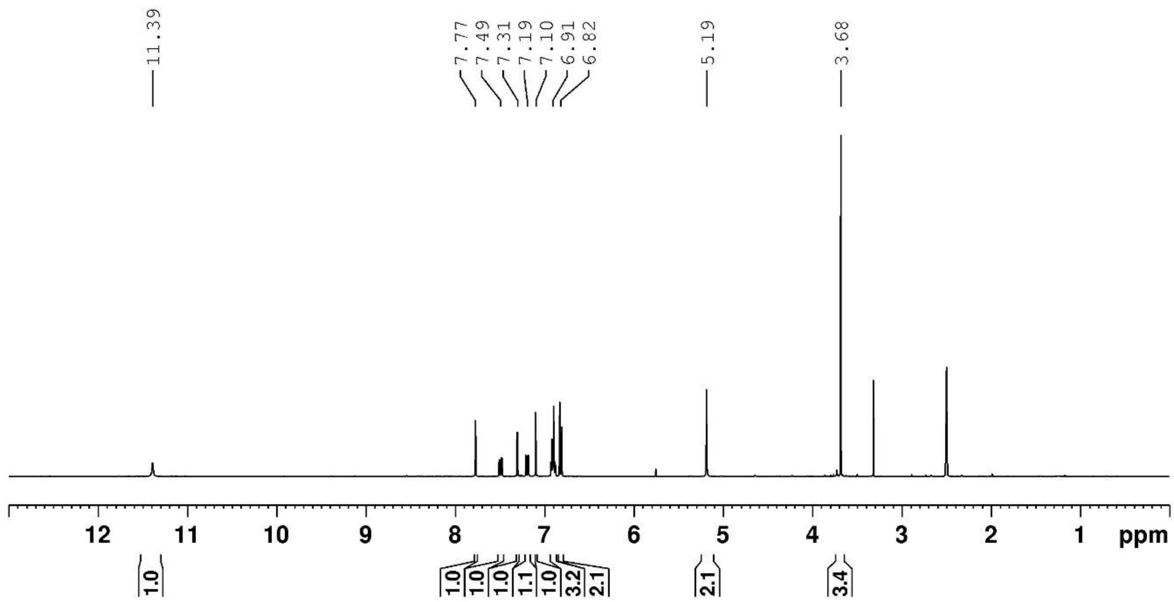

^{13}C NMR (DMSO-d_6, 100 MHz):

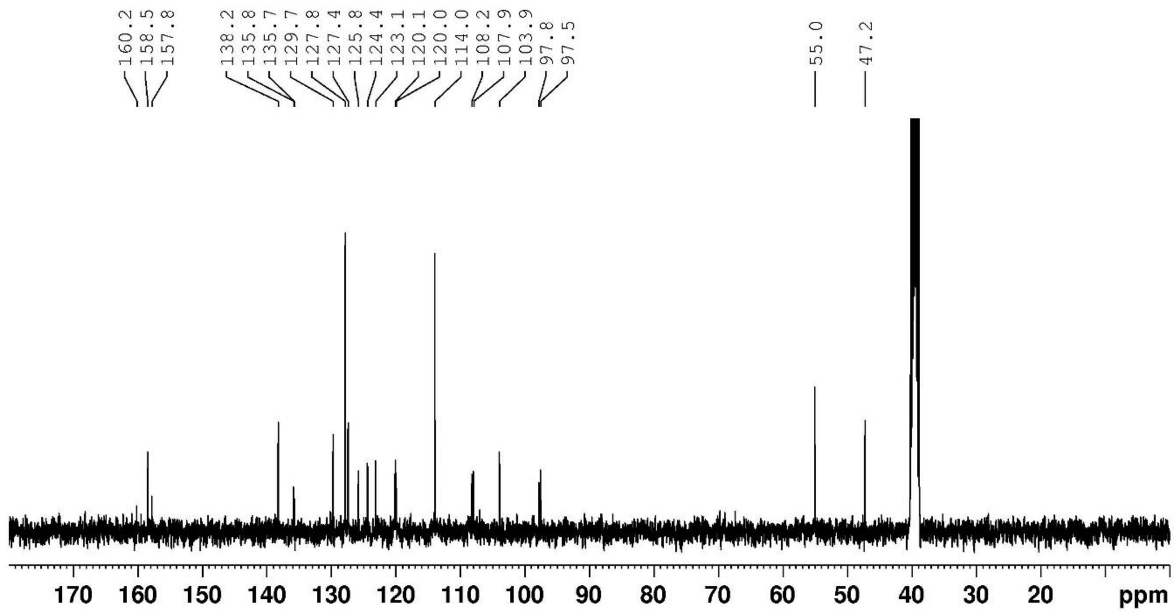

Figure S13. 6-Fluoro-3-(1-(4-methoxybenzyl)-1*H*-imidazol-5-yl)-1*H*-indole (**13**)

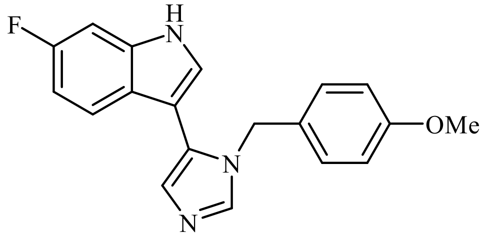

^{1}H NMR (DMSO-d_6, 400 MHz):

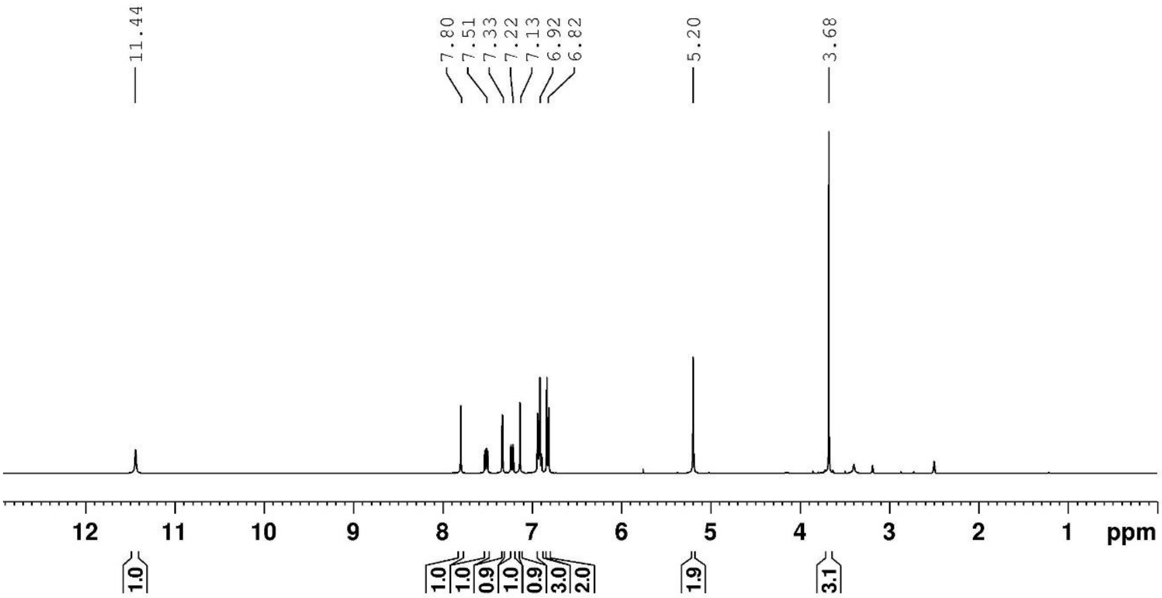

^{13}C NMR (DMSO-d_6, 100 MHz):

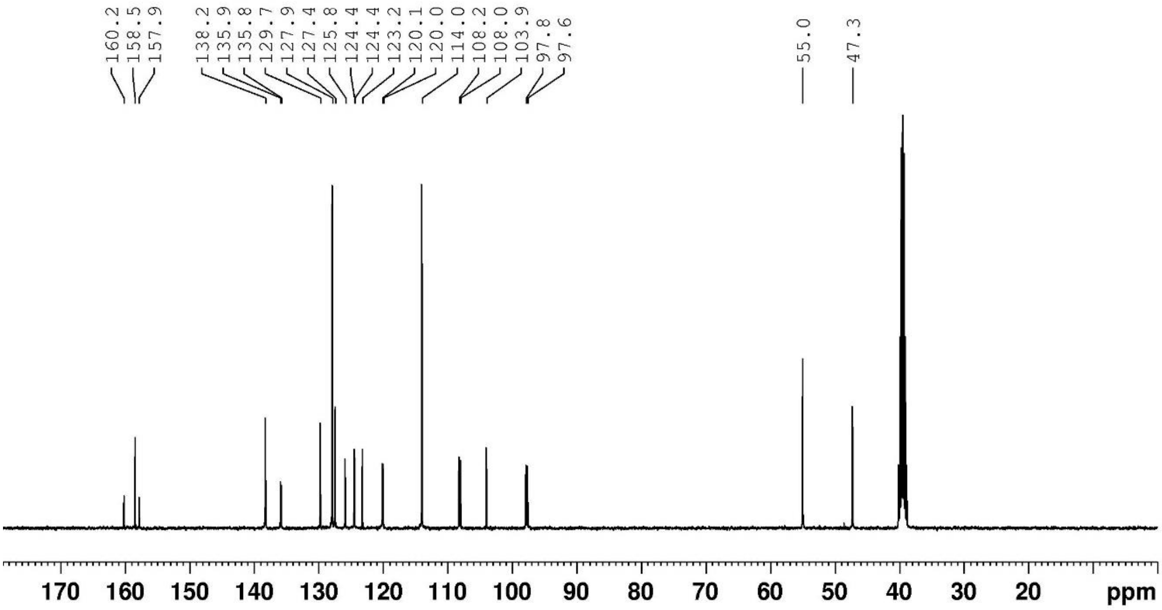

Figure S14. 5-Chloro-3-(1-(4-methoxybenzyl)-1*H*-imidazol-5-yl)-1*H*-indole (**14**)

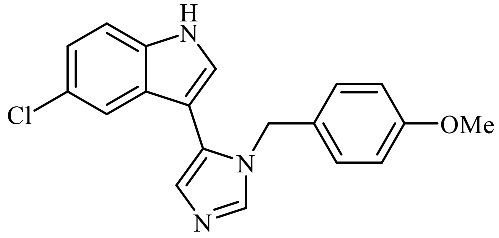

^{1}H NMR (DMSO-d_6, 400 MHz):

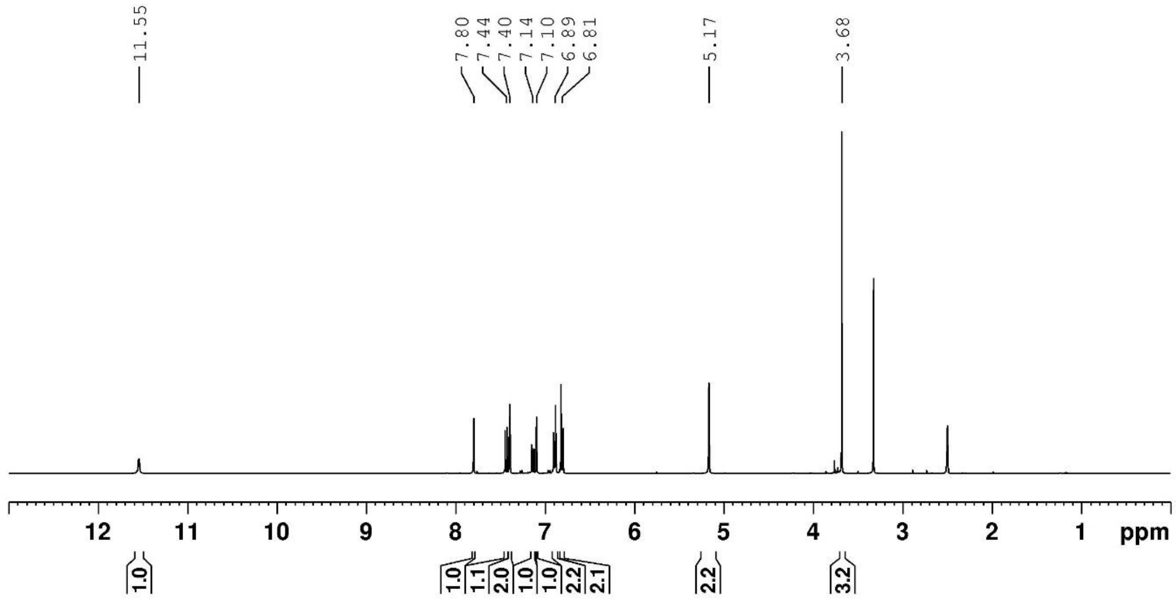

^{13}C NMR (DMSO-d_6, 100 MHz):

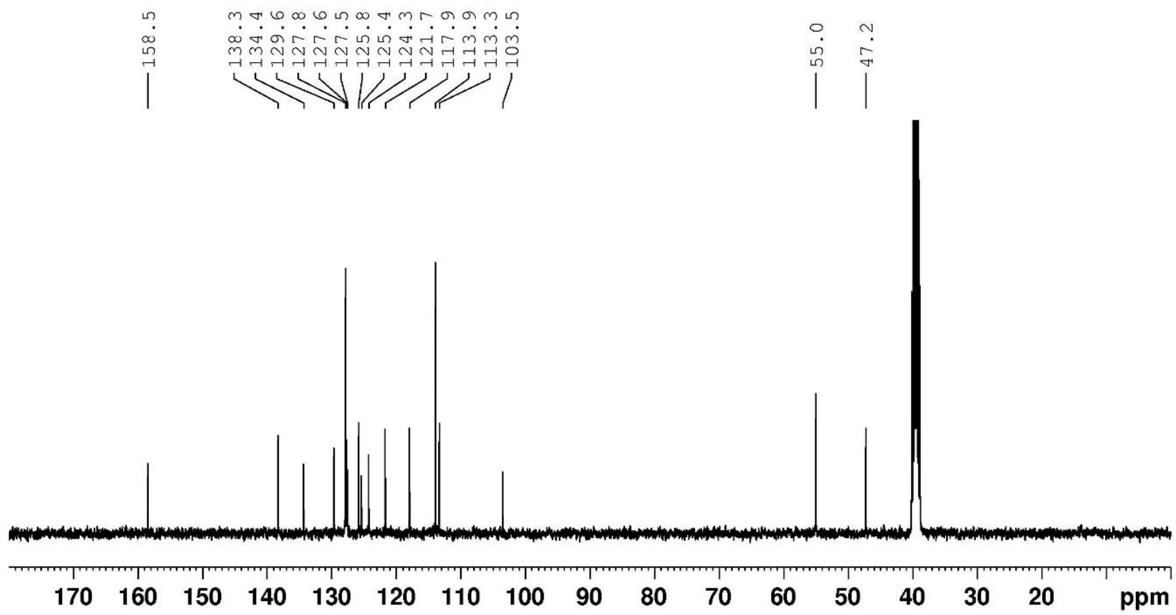

Figure S15. 6-Chloro-3-(1-(4-methoxybenzyl)-1*H*-imidazol-5-yl)-1*H*-indole (**15**)

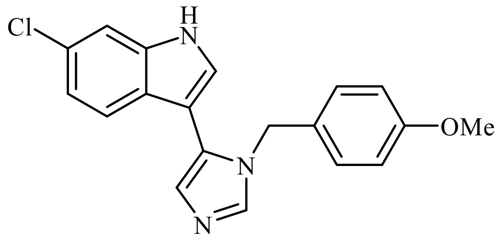

^{1}H NMR (DMSO-d_6, 400 MHz):

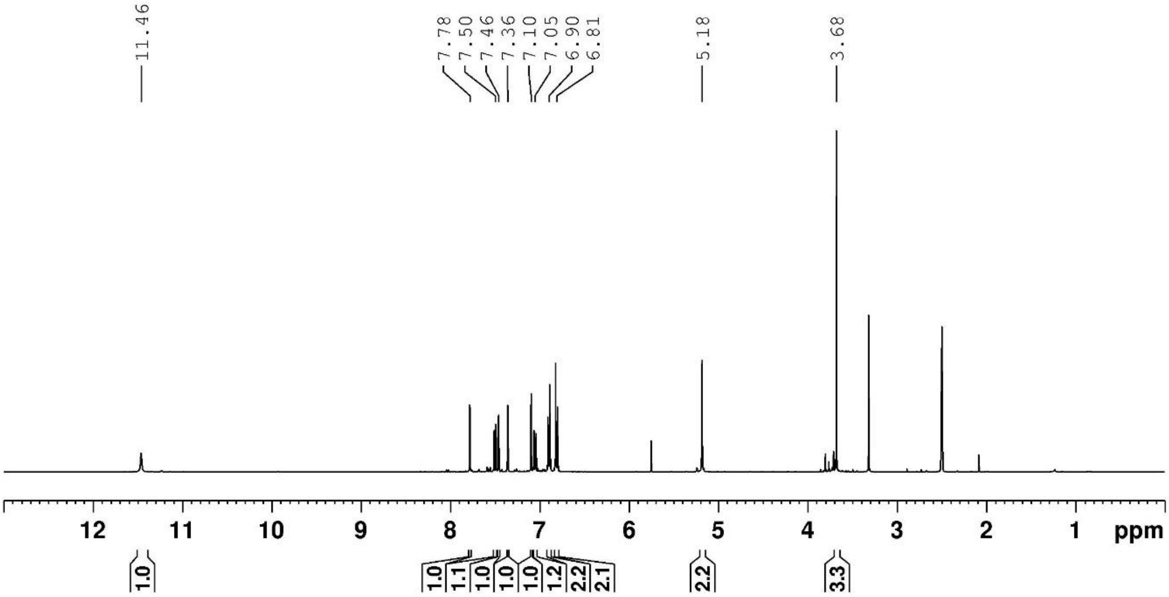

^{13}C NMR (DMSO-d_6, 100 MHz):

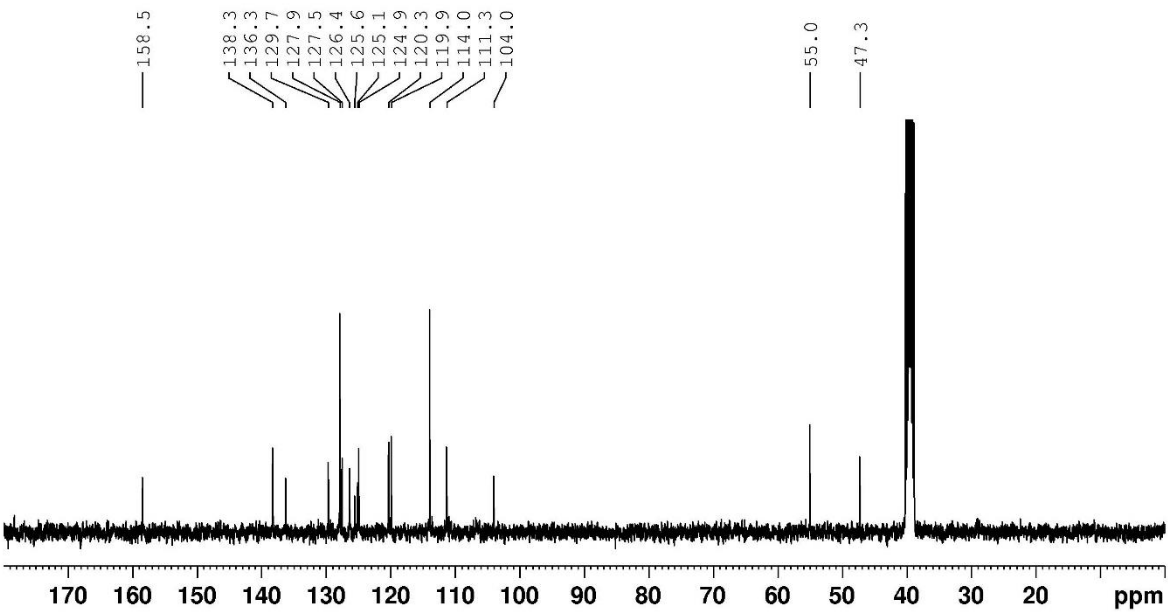

Figure S16. 5-Bromo-3-(1-(4-methoxybenzyl)-1*H*-imidazol-5-yl)-1*H*-indole (**16**)

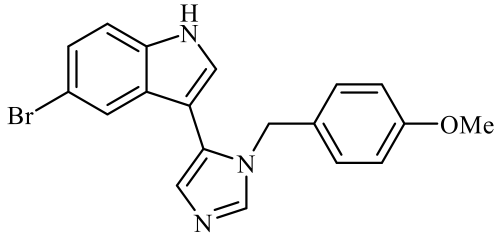

^{1}H NMR (DMSO-d_6, 400 MHz):

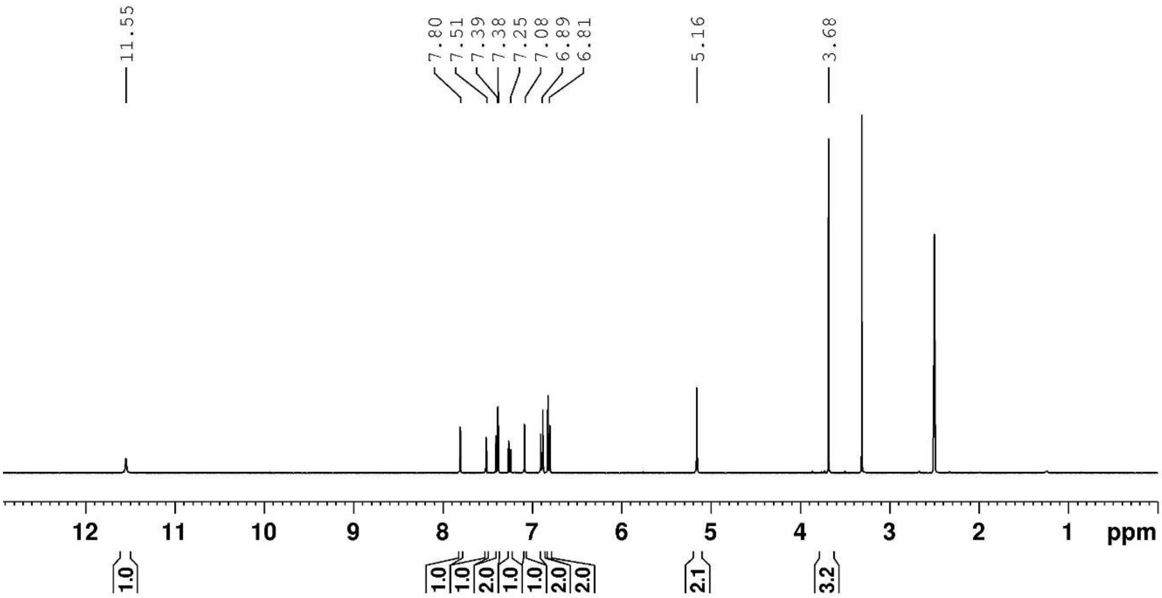

^{13}C NMR (DMSO-d_6, 100 MHz):

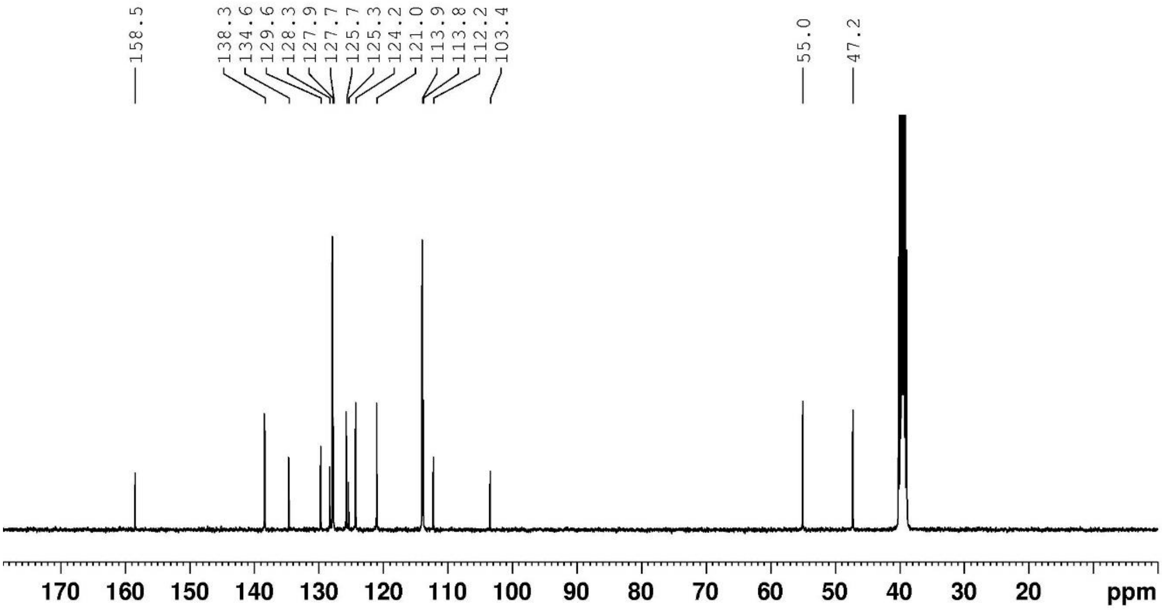

Figure S17. 6-Bromo-3-(1-(4-methoxybenzyl)-1*H*-imidazol-5-yl)-1*H*-indole (**17**)

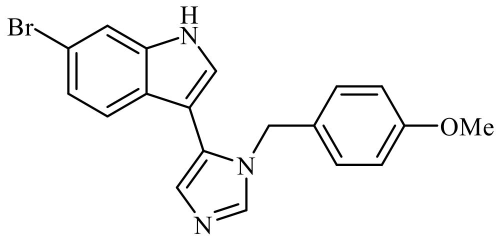

^{1}H NMR (DMSO-d_6, 400 MHz):

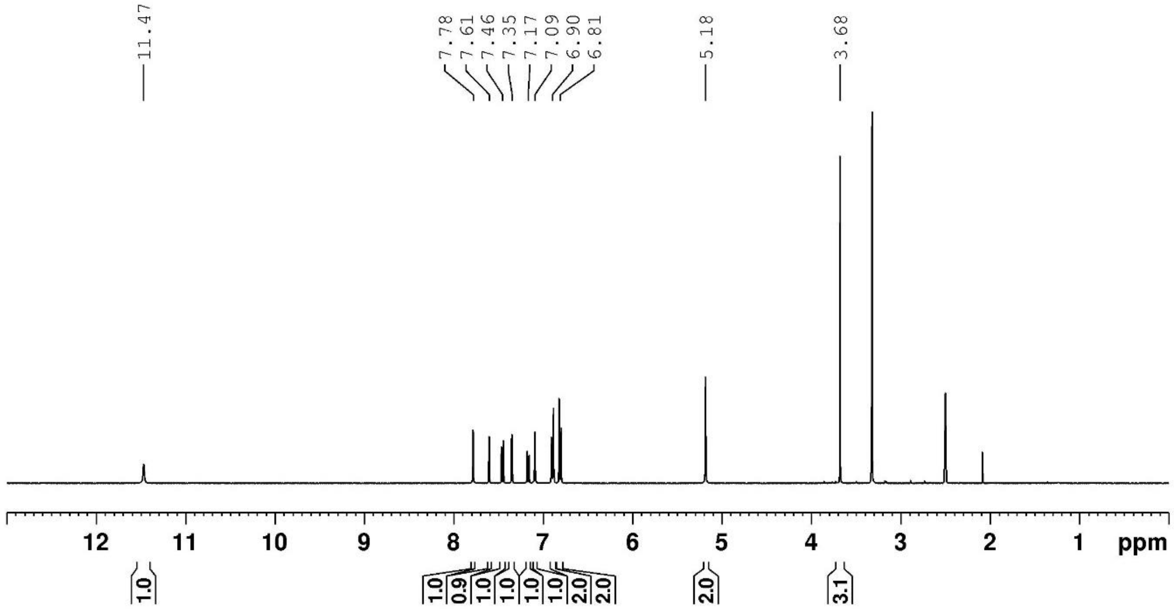

^{13}C NMR (DMSO-d_6, 100 MHz):

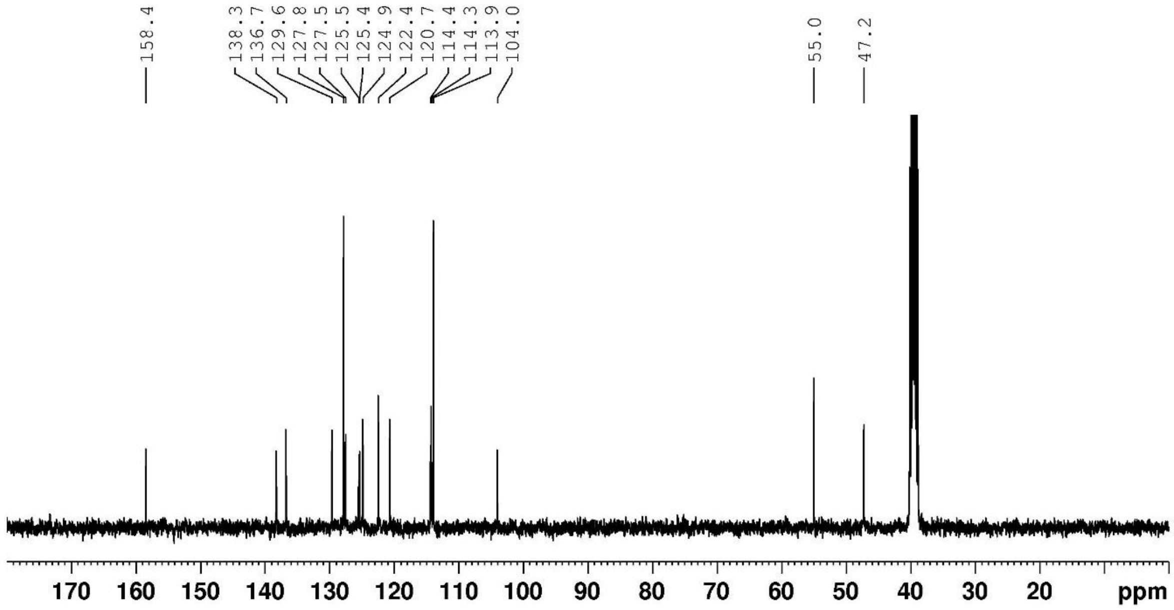

Figure S18. 6-Fluoro-3-(1-phenethyl-1*H*-imidazol-5-yl)-1*H*-indole (**18**)

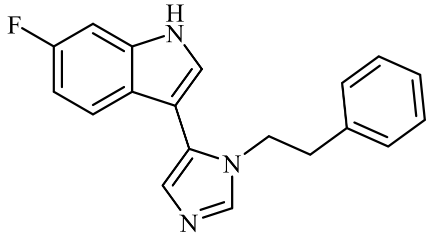

^{1}H NMR (DMSO-d_6, 400 MHz):

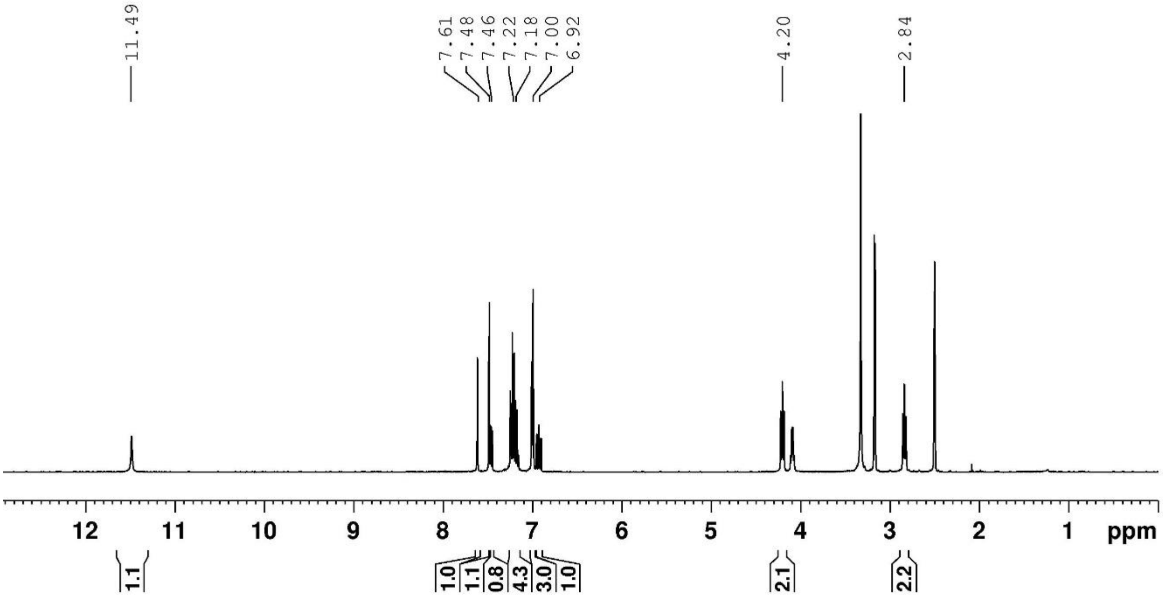

^{13}C NMR (DMSO-d_6, 100 MHz):

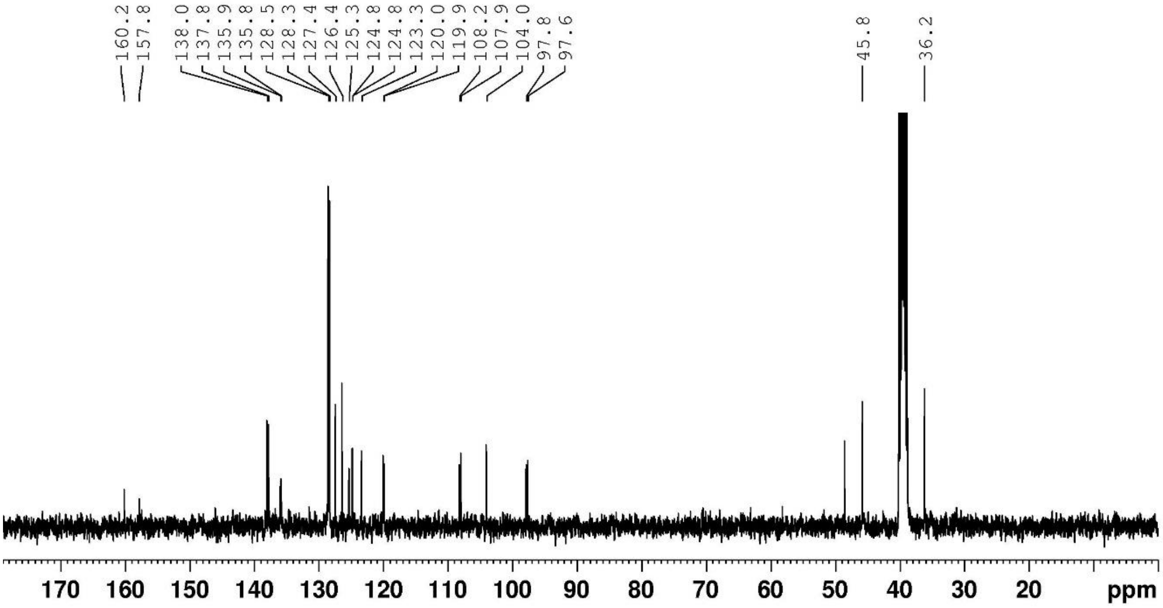

Figure S19. 5-Chloro-3-(1-phenethyl-1*H*-imidazol-5-yl)-1*H*-indole (**19**)

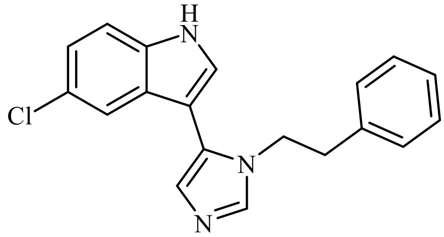

^{1}H NMR (DMSO-d_6, 400 MHz):

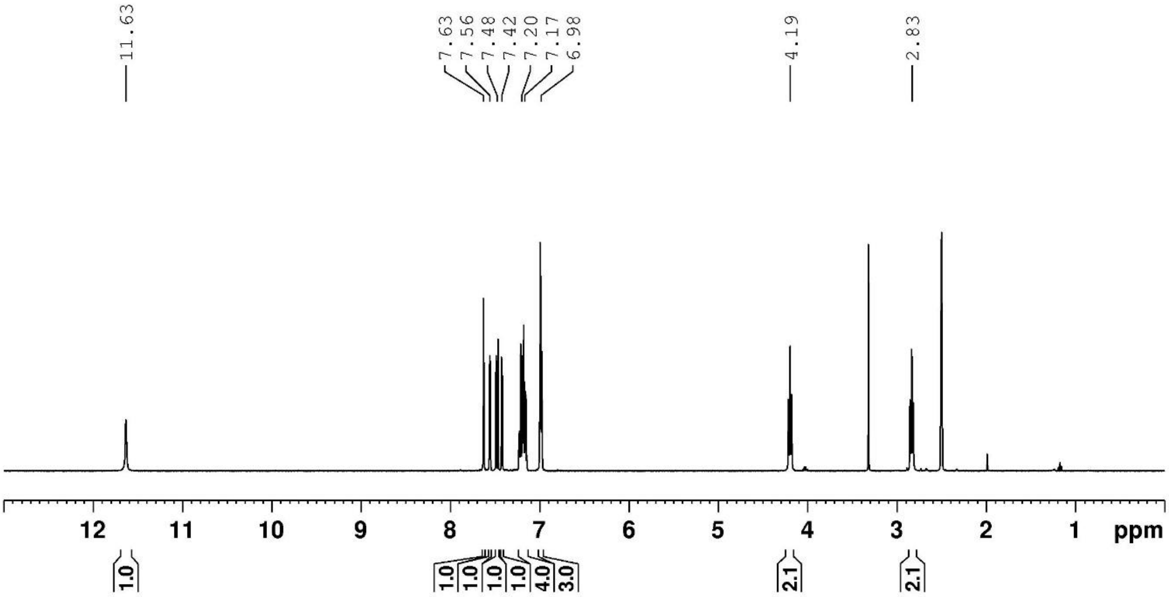

^{13}C NMR (DMSO-d_6, 100 MHz):

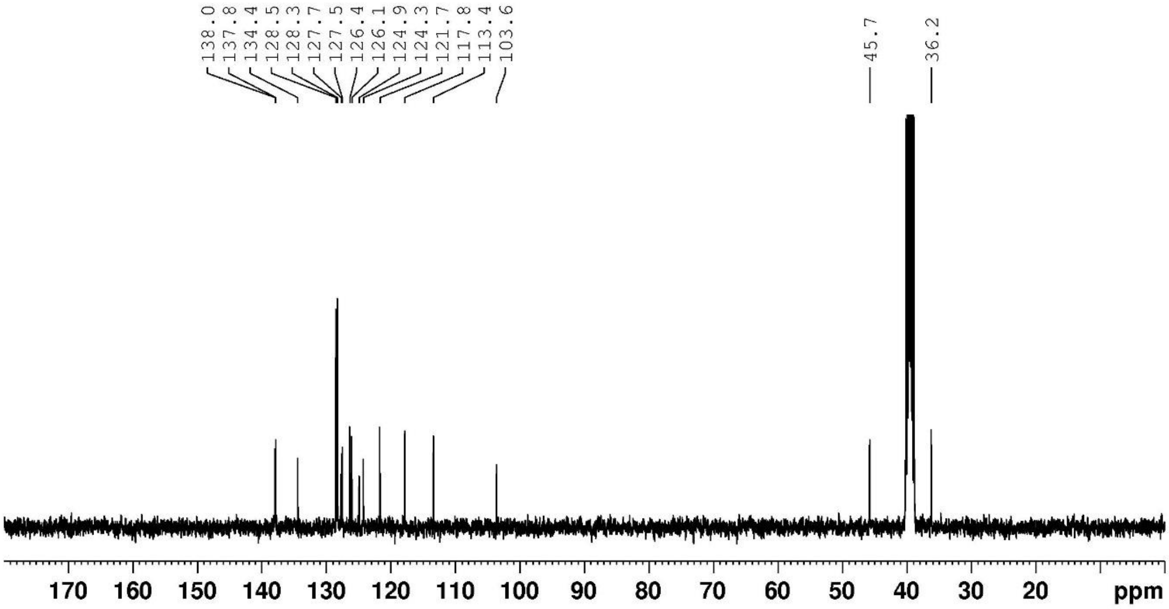

Figure S20. 6-Chloro-3-(1-phenethyl-1*H*-imidazol-5-yl)-1*H*-indole (**20**)

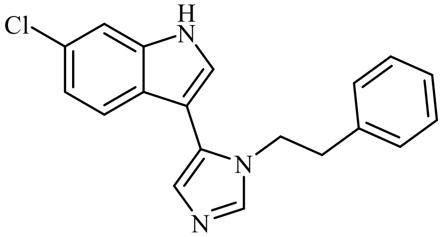

^{1}H NMR (DMSO-*d*6, 400 MHz):

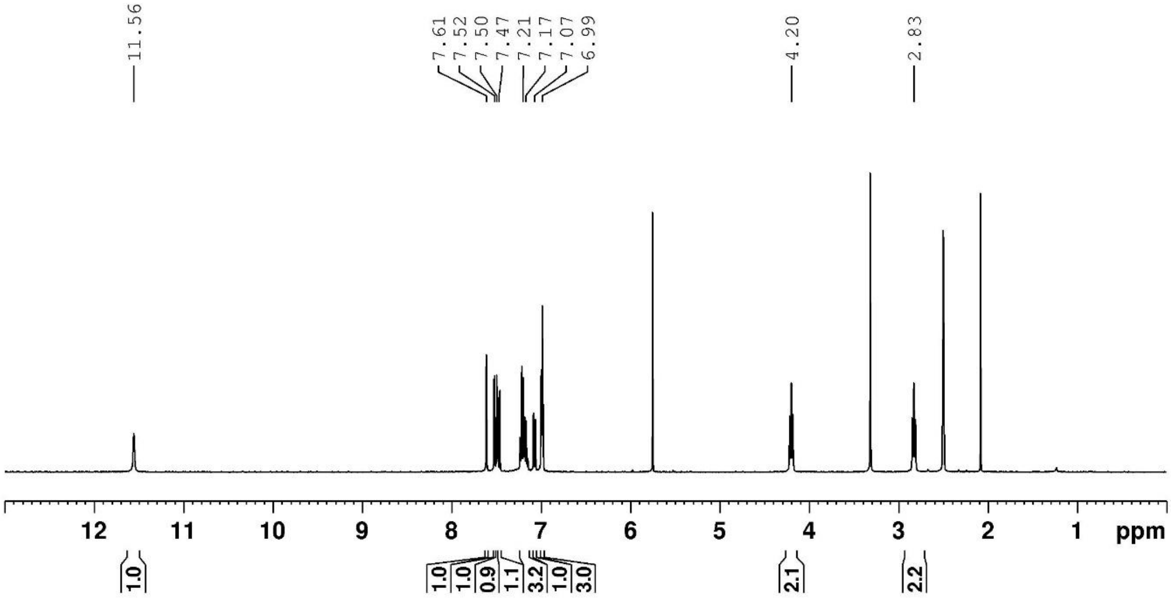

^{13}C NMR (DMSO-*d*6, 100 MHz):

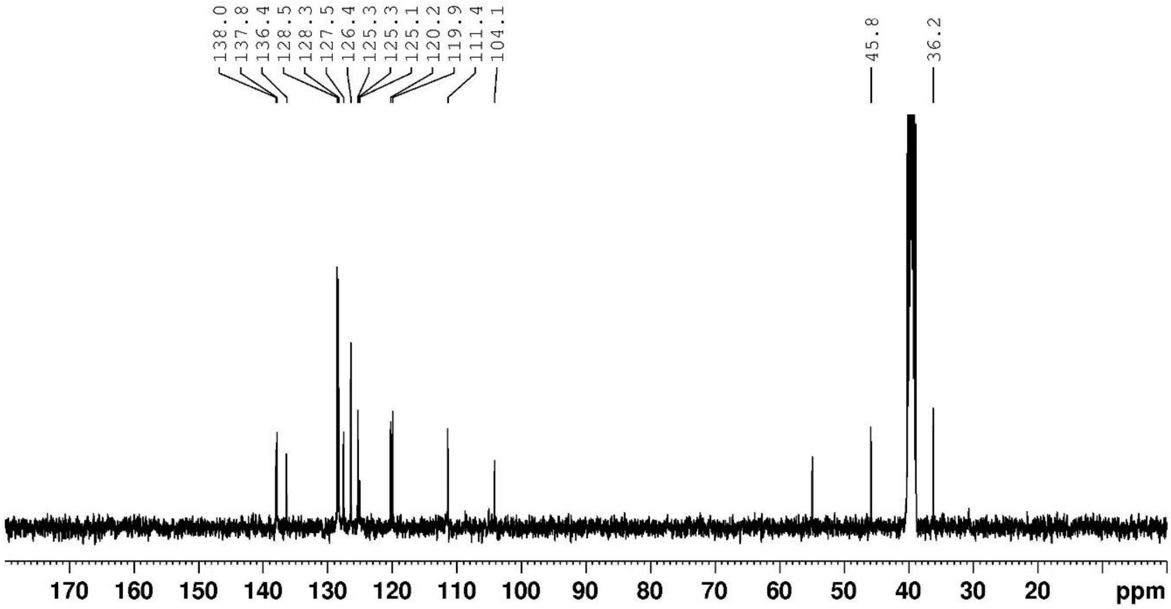

Figure S21. 5-Bromo-3-(1-phenethyl-1*H*-imidazol-5-yl)-1*H*-indole (**21**)

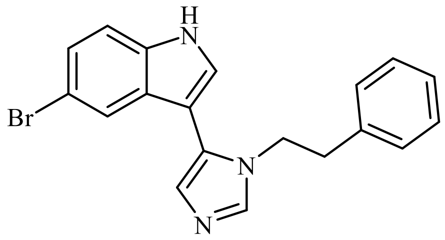

^{1}H NMR (DMSO-d_6, 400 MHz):

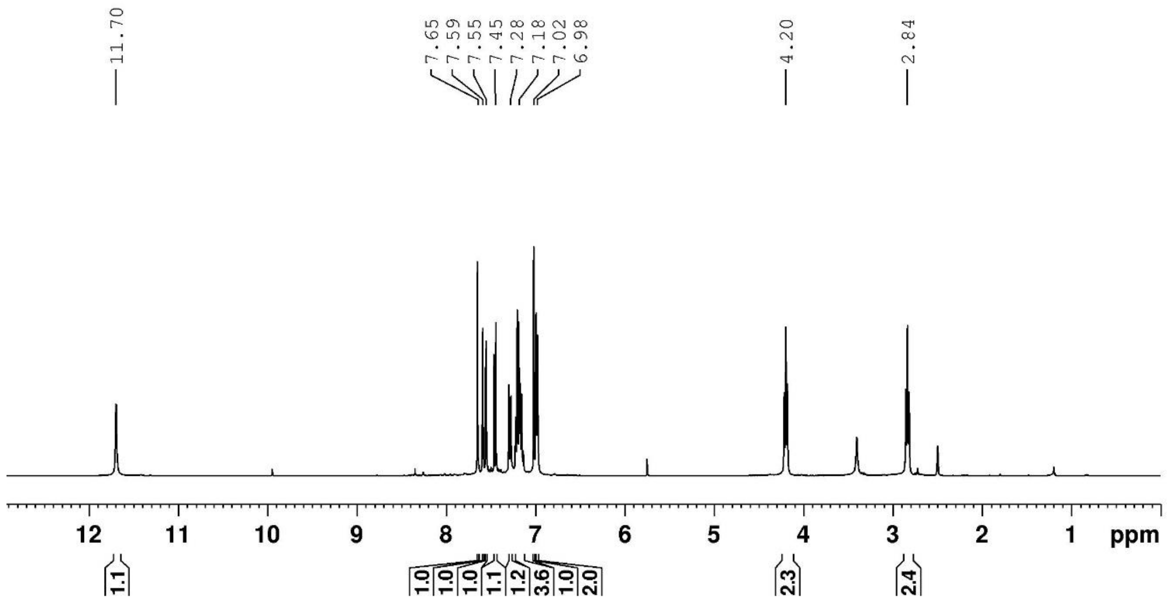

^{13}C NMR (DMSO-d_6, 100 MHz):

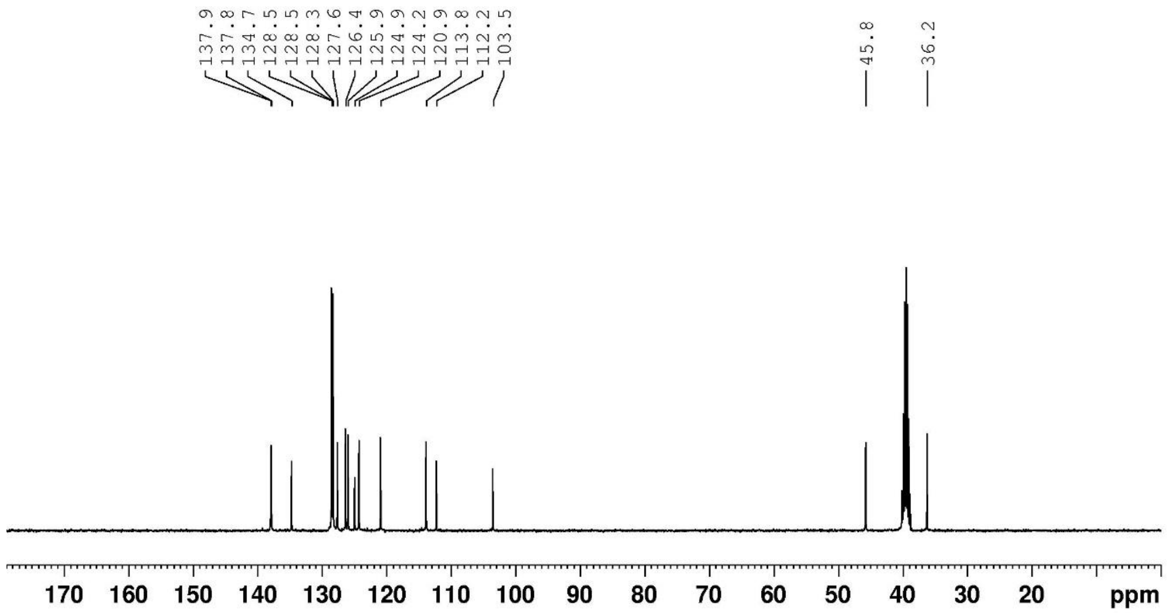

Figure S22. 6-Bromo-3-(1-phenethyl-1*H*-imidazol-5-yl)-1*H*-indole (**22**)

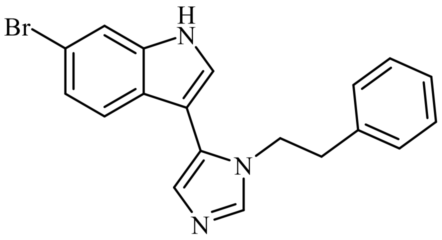

^{1}H NMR (DMSO-d_6, 400 MHz):

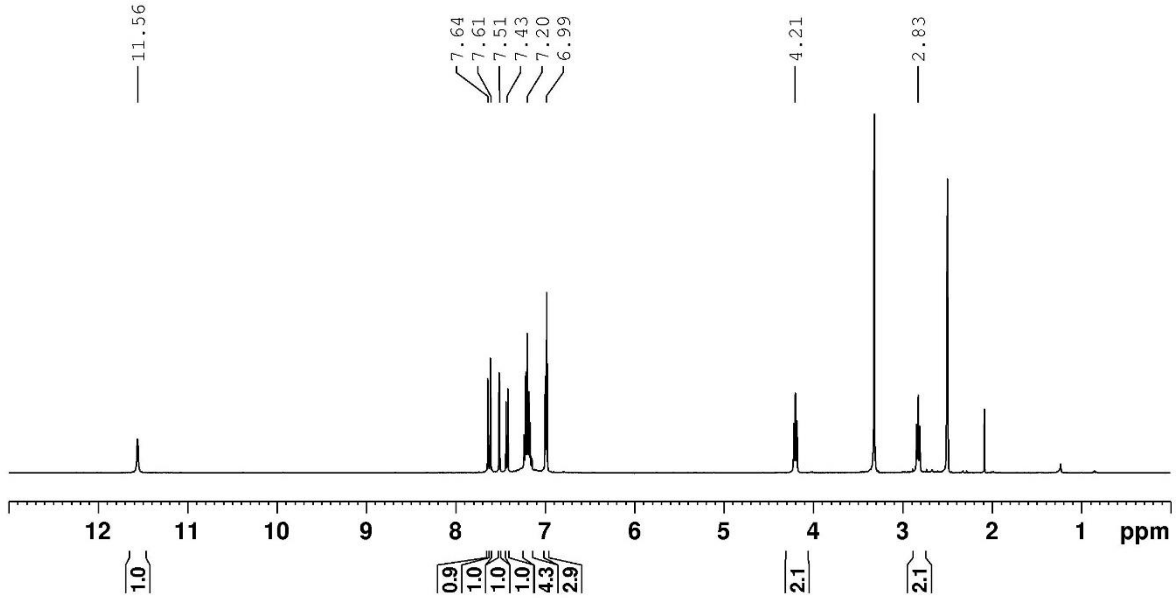

^{13}C NMR (DMSO-d_6, 100 MHz):

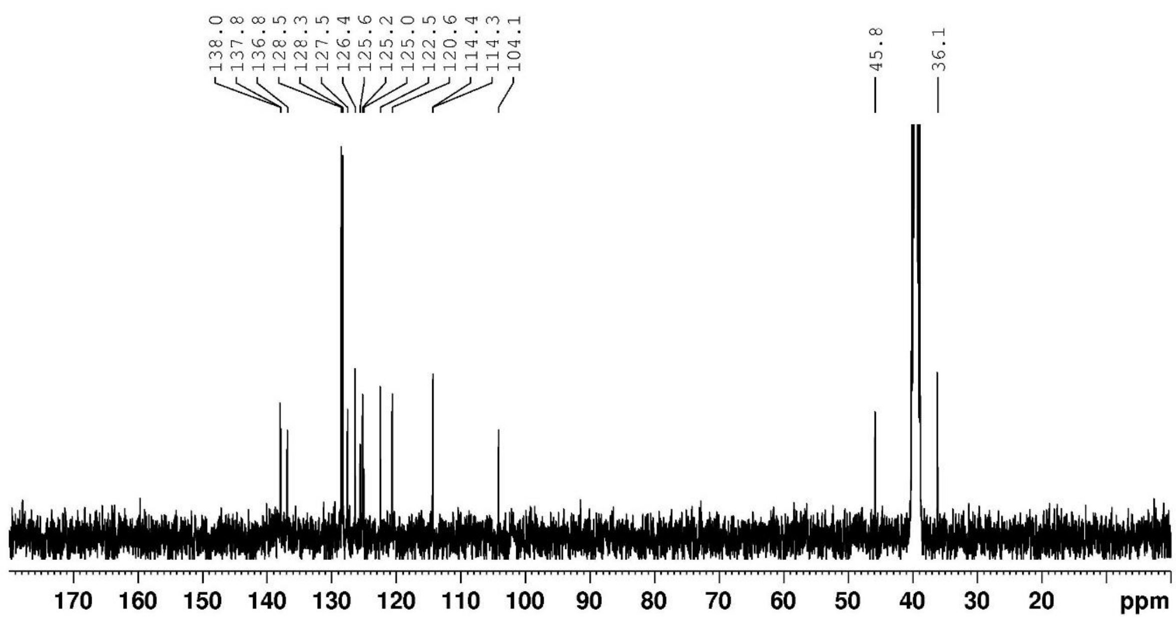

Figure S23. 3-(1-(4-Methoxyphenethyl)-1*H*-imidazol-5-yl)-1*H*-indole (**23**)

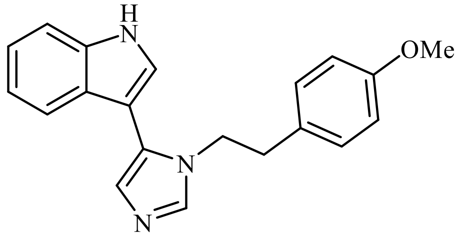

^{1}H NMR (DMSO-d_6, 400 MHz):

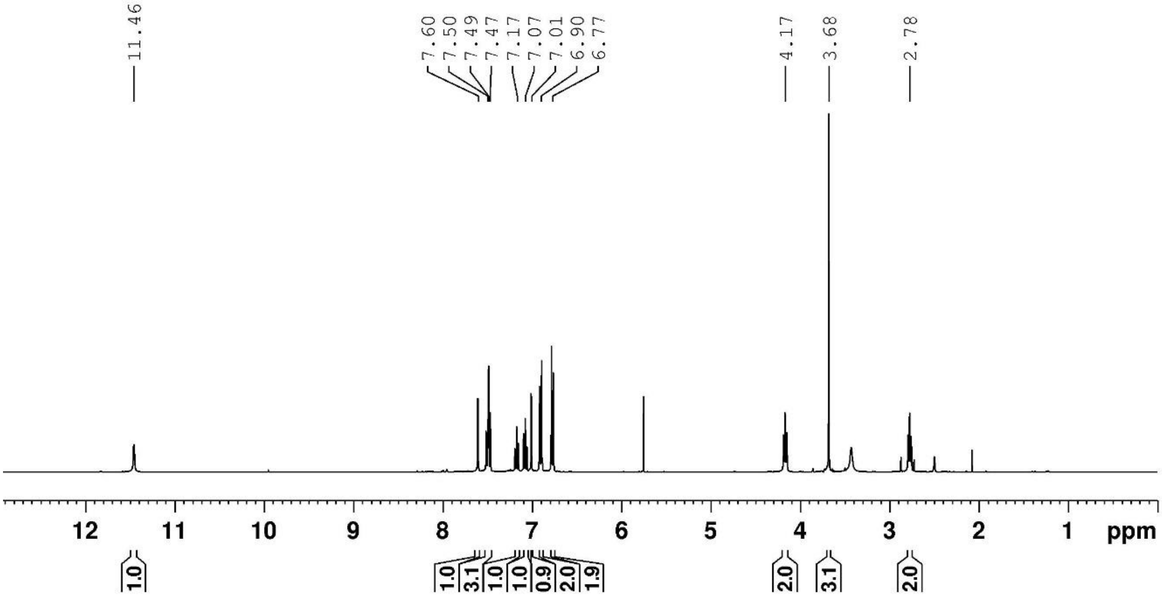

^{13}C NMR (DMSO-d_6, 100 MHz):

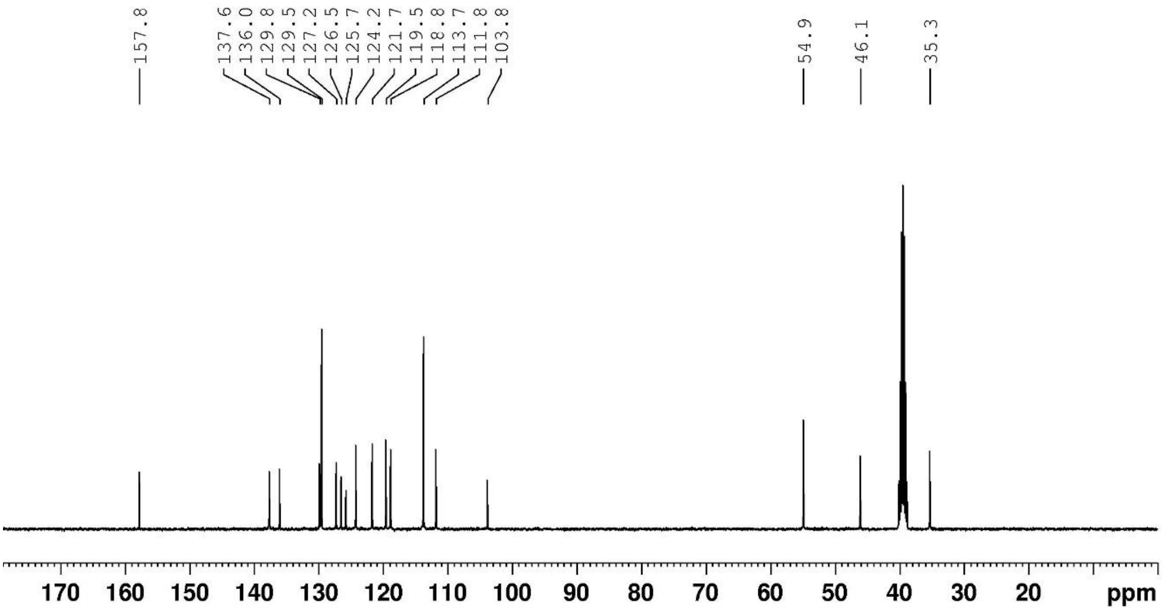

Figure S24. 5-Fluoro-3-(1-(4-methoxyphenethyl)-1*H*-imidazol-5-yl)-1*H*-indole (**24**)

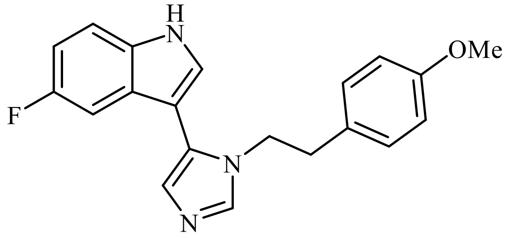

^{1}H NMR (DMSO-d_6, 400 MHz):

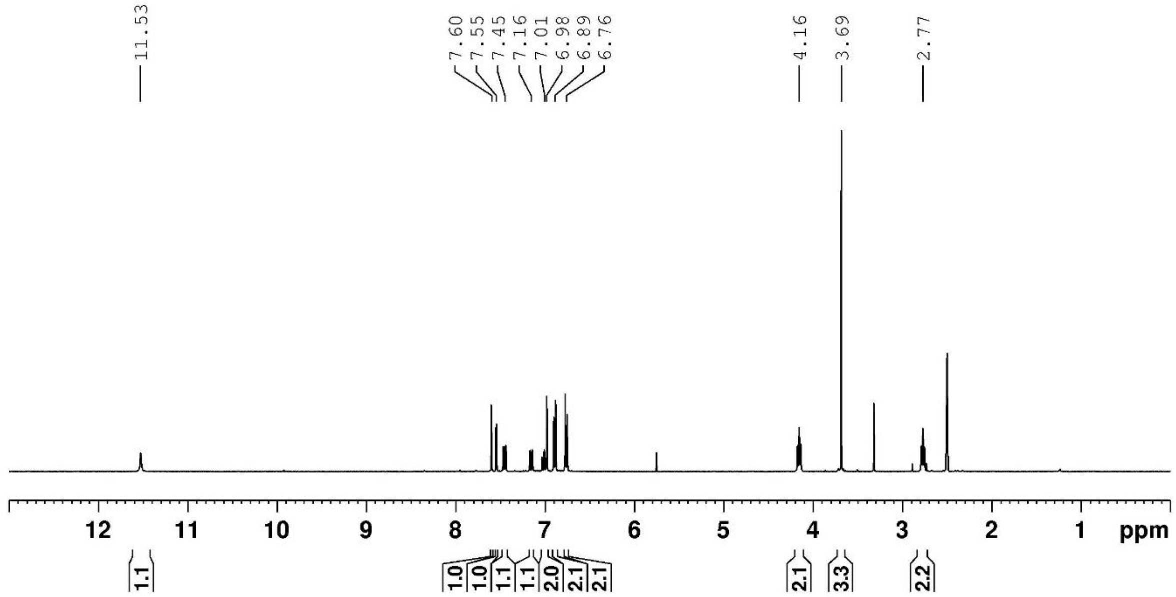

^{13}C NMR (DMSO-d_6, 100 MHz):

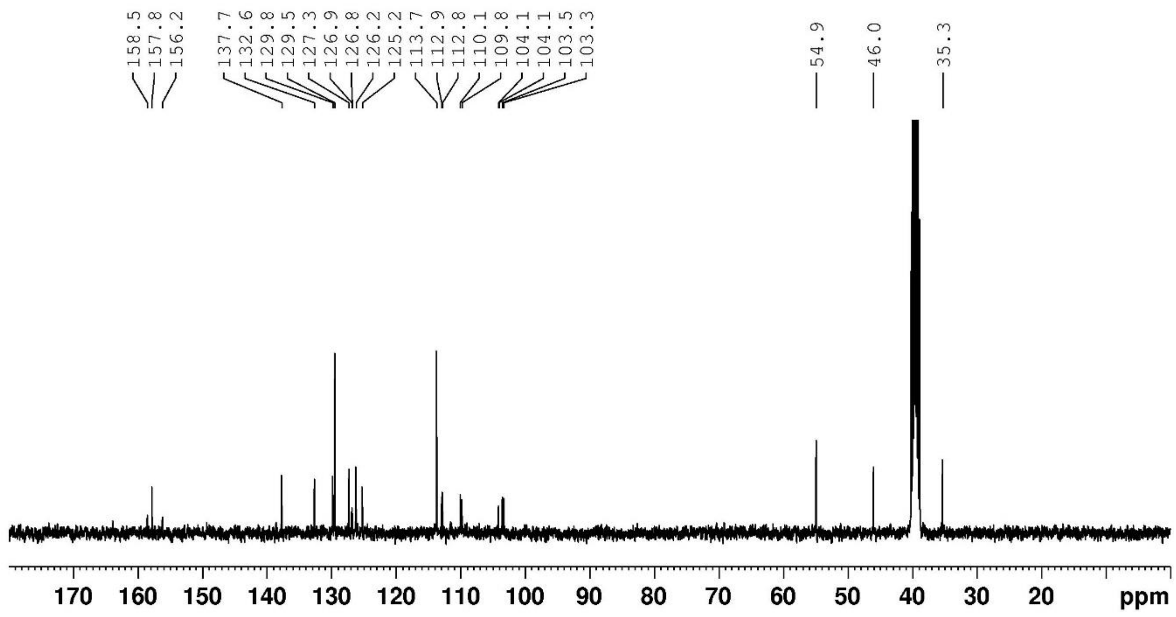

Figure S25. 6-Fluoro-3-(1-(4-methoxyphenethyl)-1*H*-imidazol-5-yl)-1*H*-indole (**25**)

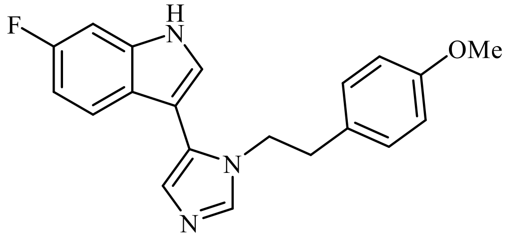

^{1}H NMR (DMSO-d_6, 400 MHz):

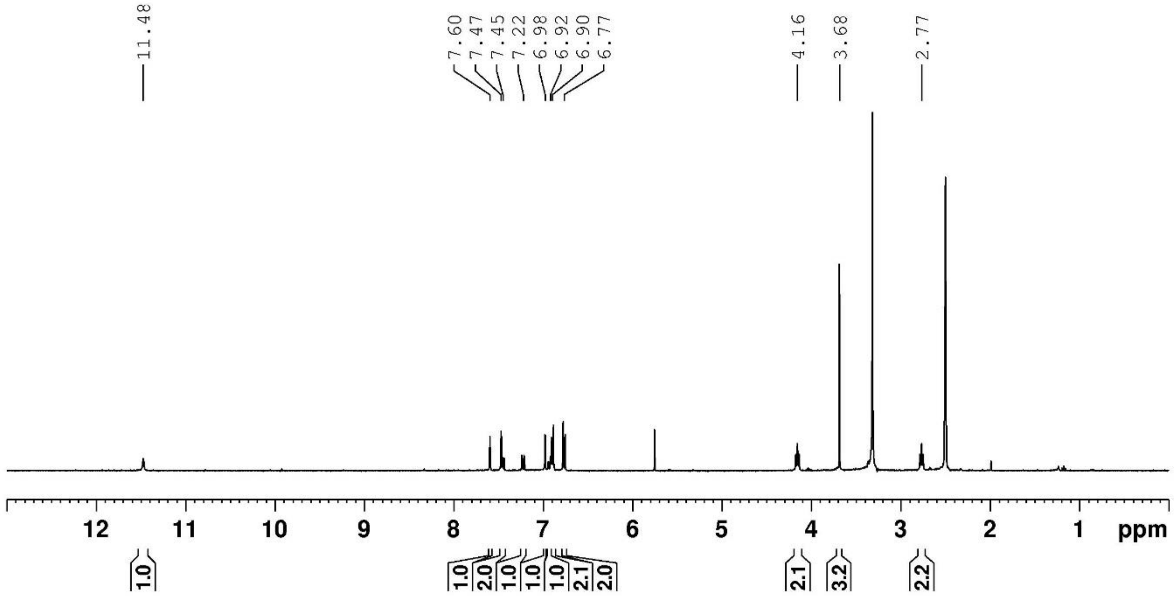

^{13}C NMR (DMSO-d_6, 100 MHz):

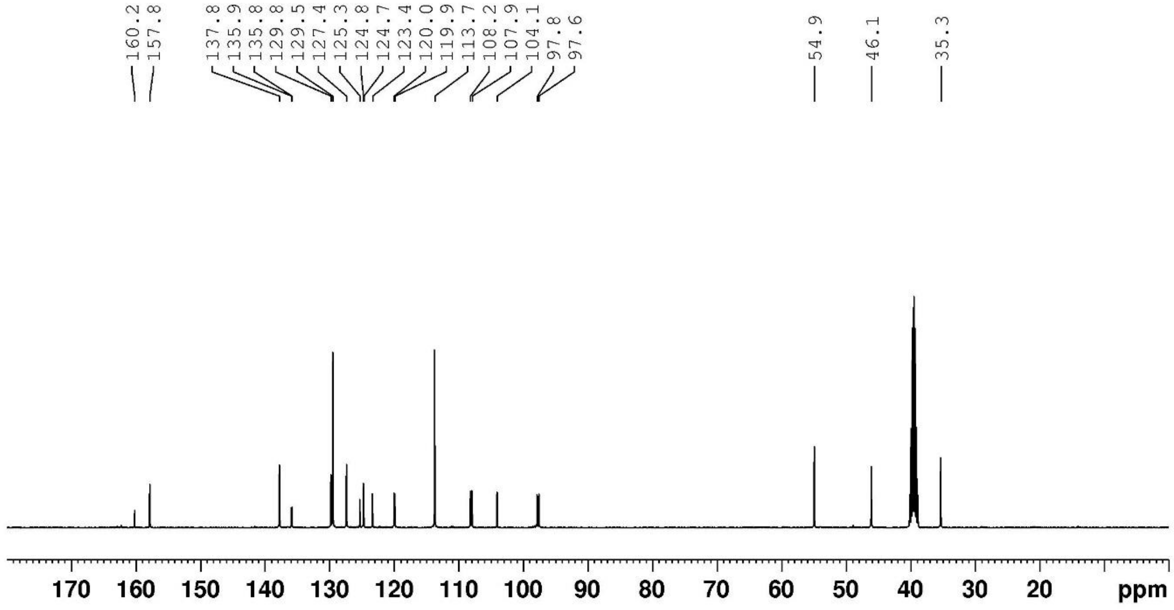

Figure S26. 5-Chloro-3-(1-(4-methoxyphenethyl)-1*H*-imidazol-5-yl)-1*H*-indole (**26**)

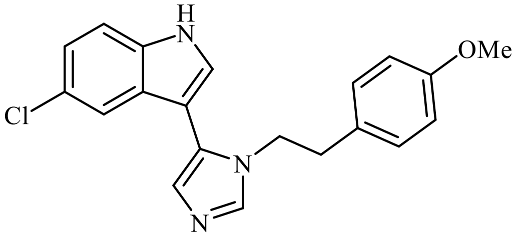

^{1}H NMR (DMSO-d_6, 400 MHz):

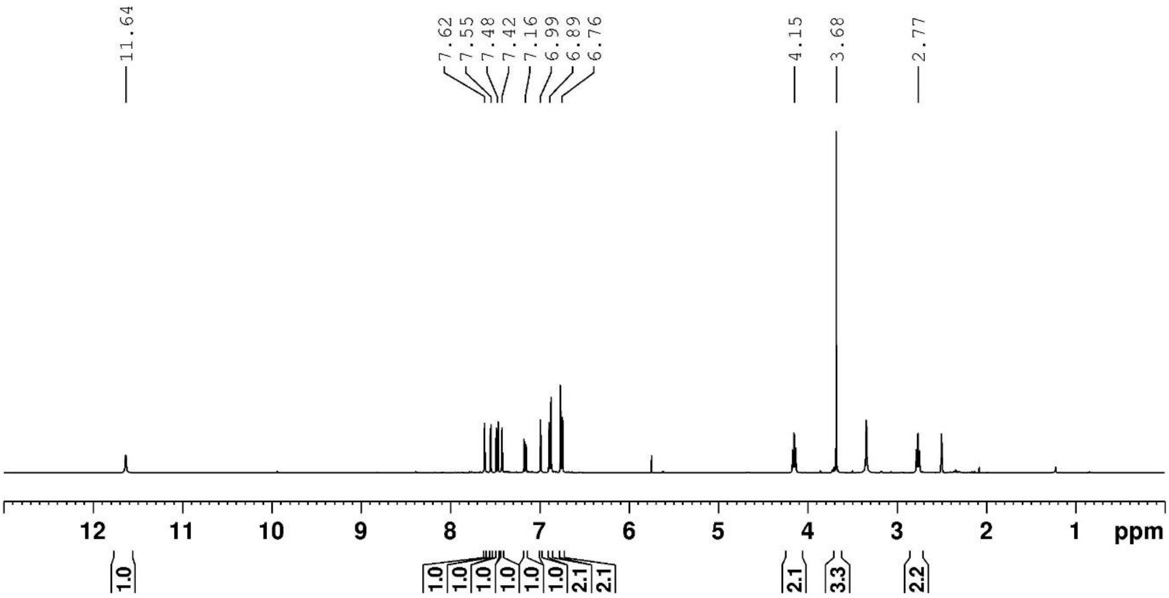

^{13}C NMR (DMSO-d_6, 100 MHz):

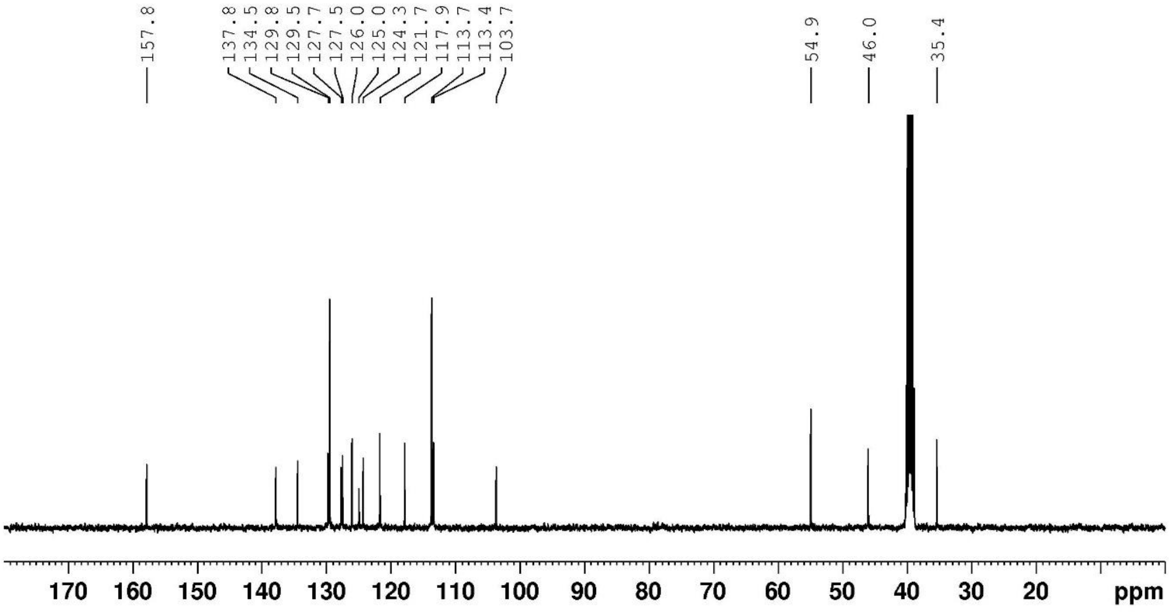

Figure S27. 5-Bromo-3-(1-(4-methoxyphenethyl)-1*H*-imidazol-5-yl)-1*H*-indole (**27**)

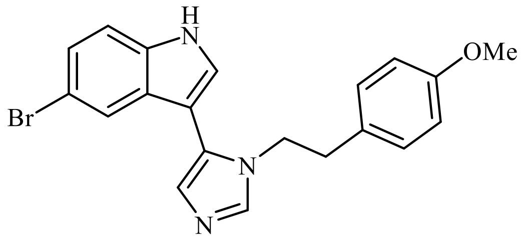

^{1}H NMR (DMSO-d_6, 400 MHz):

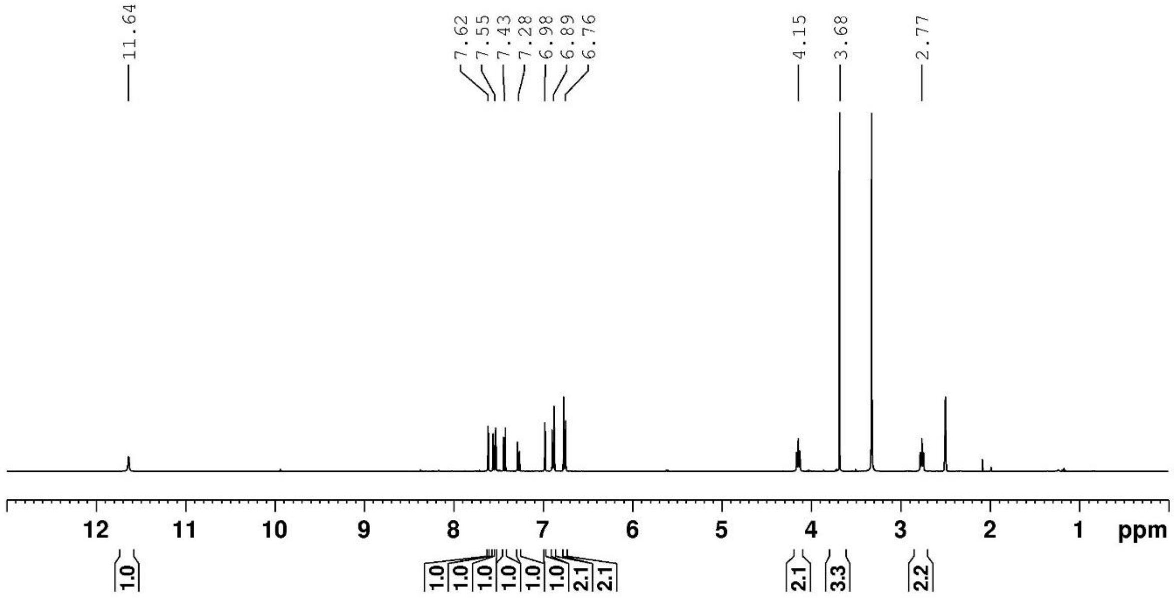

^{13}C NMR (DMSO-d_6, 100 MHz):

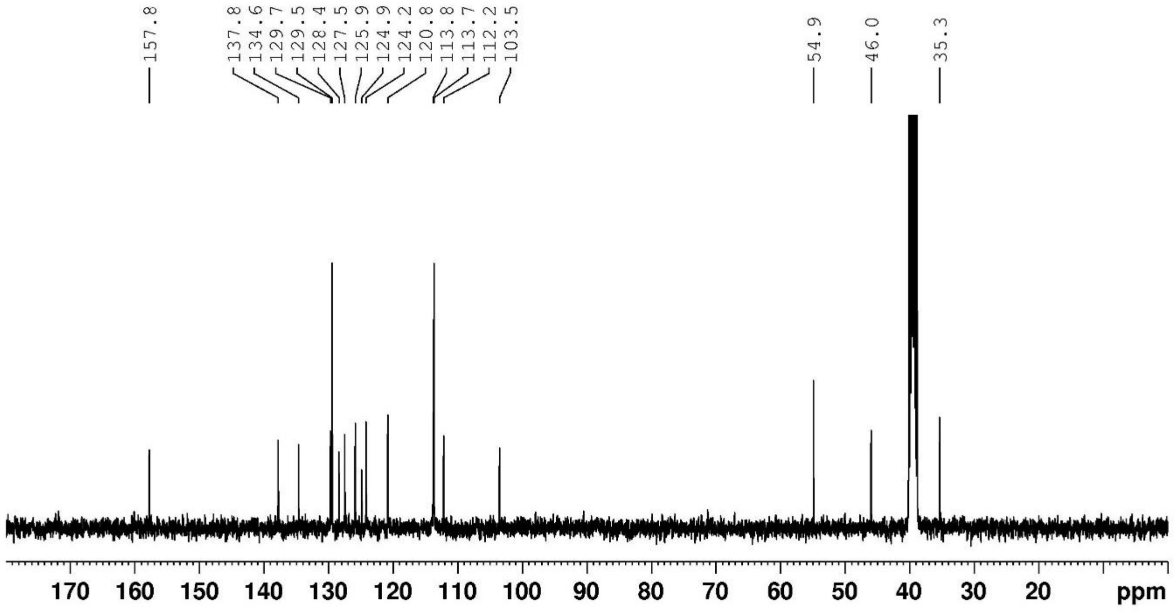

Figure S28. 6-Bromo-3-(1-(4-methoxyphenethyl)-1*H*-imidazol-5-yl)-1*H*-indole (**28**)

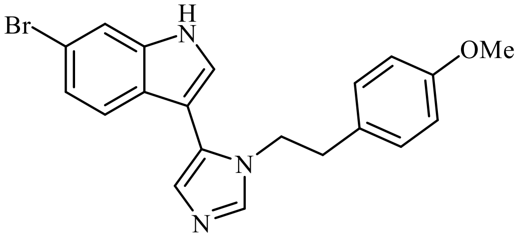

^{1}H NMR (DMSO-d_6, 400 MHz):

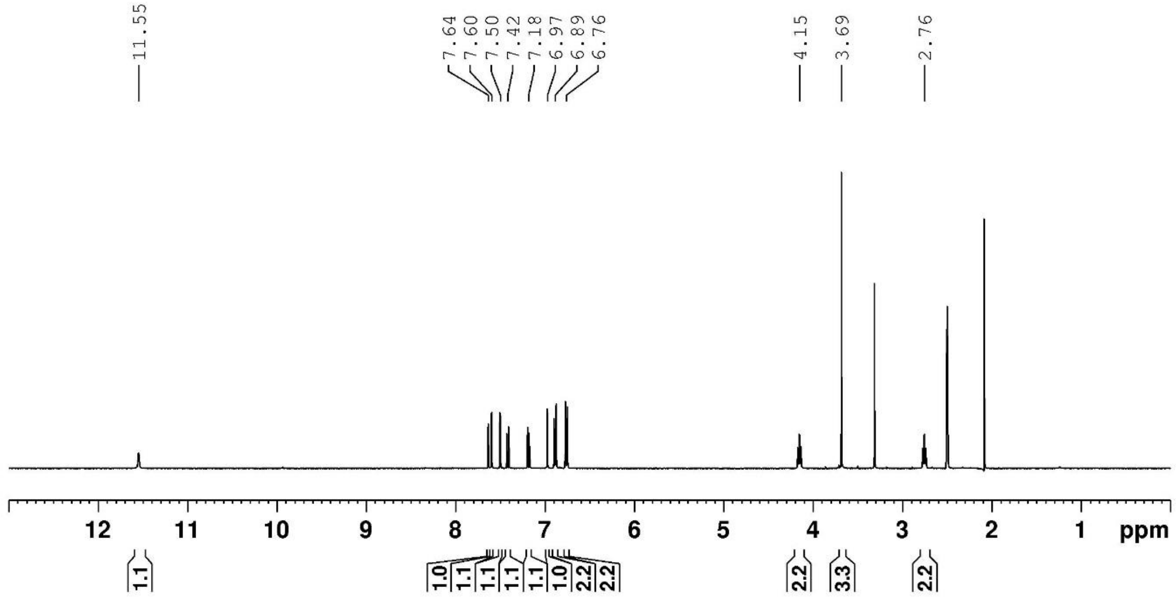

^{13}C NMR (DMSO-d_6, 100 MHz):

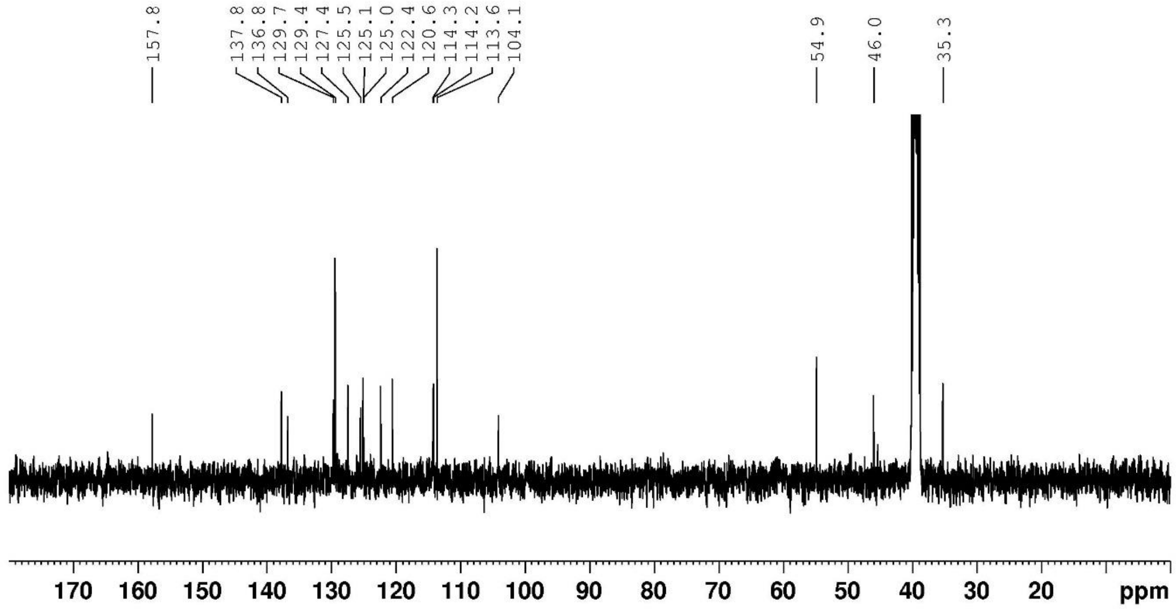

Figure S29. 3-(1-(2,5-Dimethoxyphenethyl)-1*H*-imidazol-5-yl)-1*H*-indole (**29**)

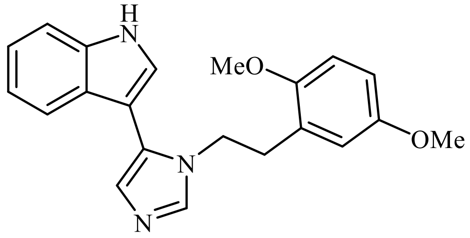

^{1}H NMR (DMSO-d_6, 400 MHz):

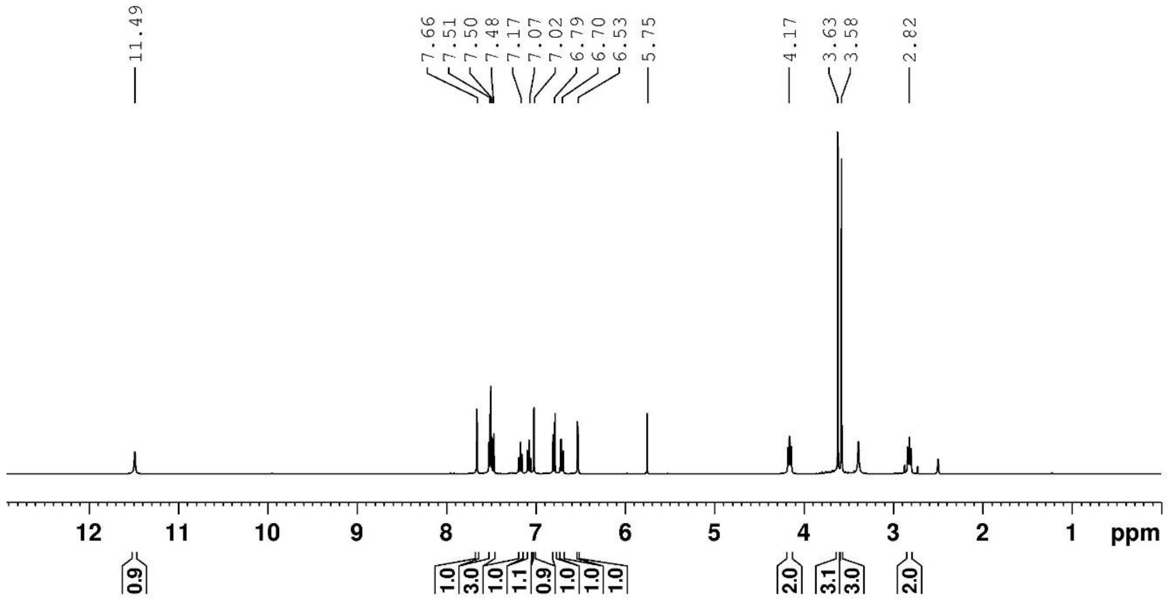

^{13}C NMR (DMSO-d_6, 100 MHz):

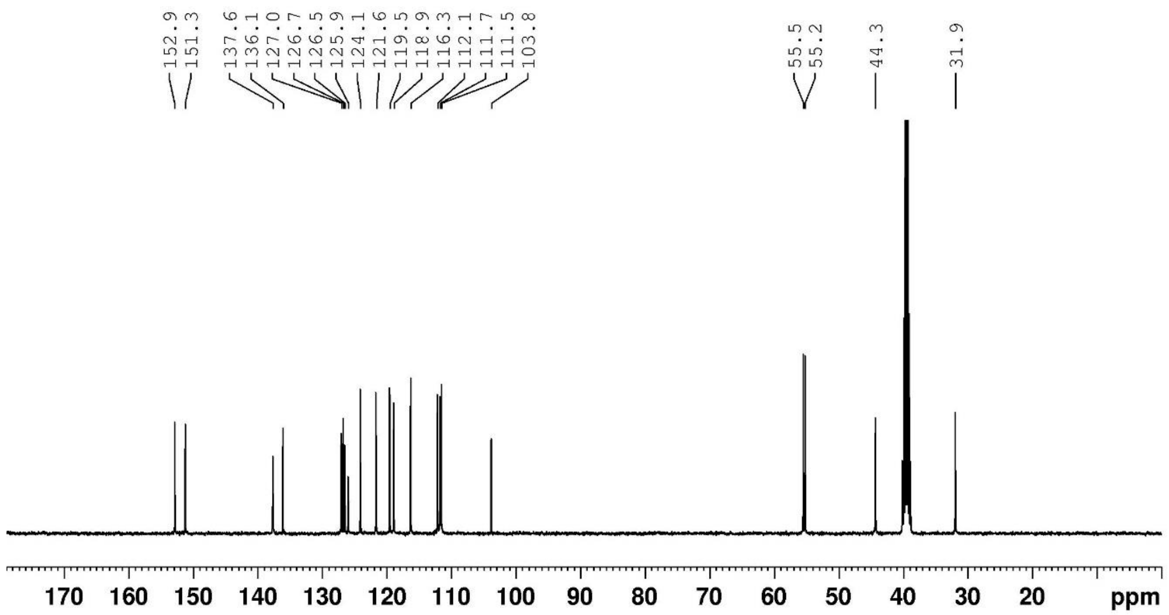

Figure S30. 3-(1-(2,5-Dimethoxyphenethyl)-1*H*-imidazol-5-yl)-5-fluoro-1*H*-indole (**30**)

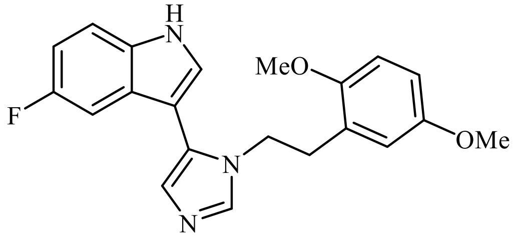

^{1}H NMR (DMSO-d_6, 400 MHz):

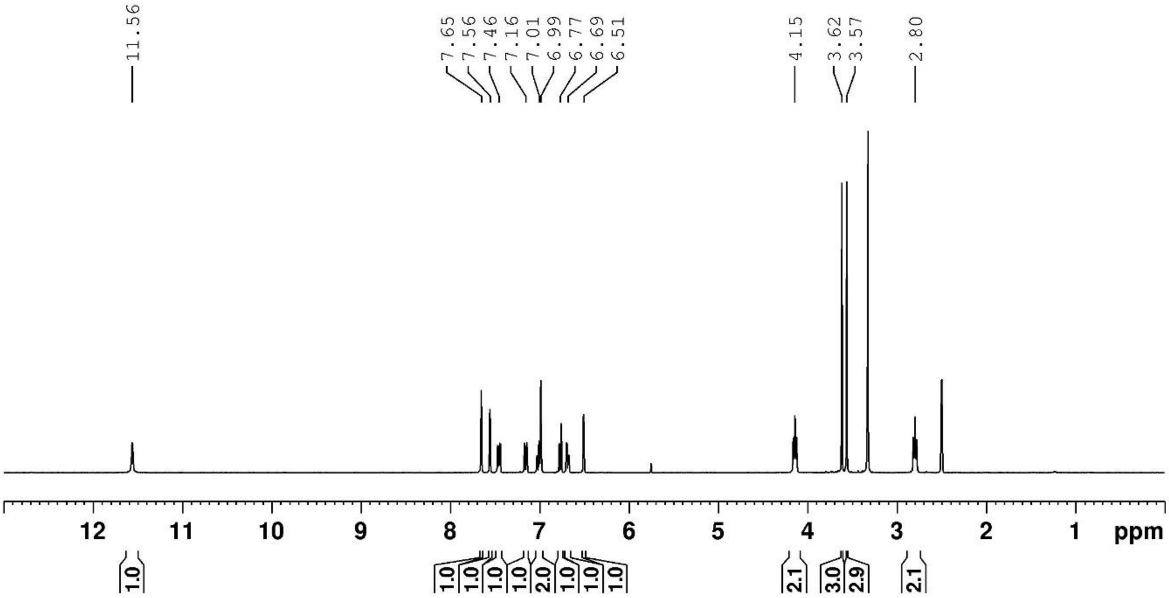

^{13}C NMR (DMSO-d_6, 100 MHz):

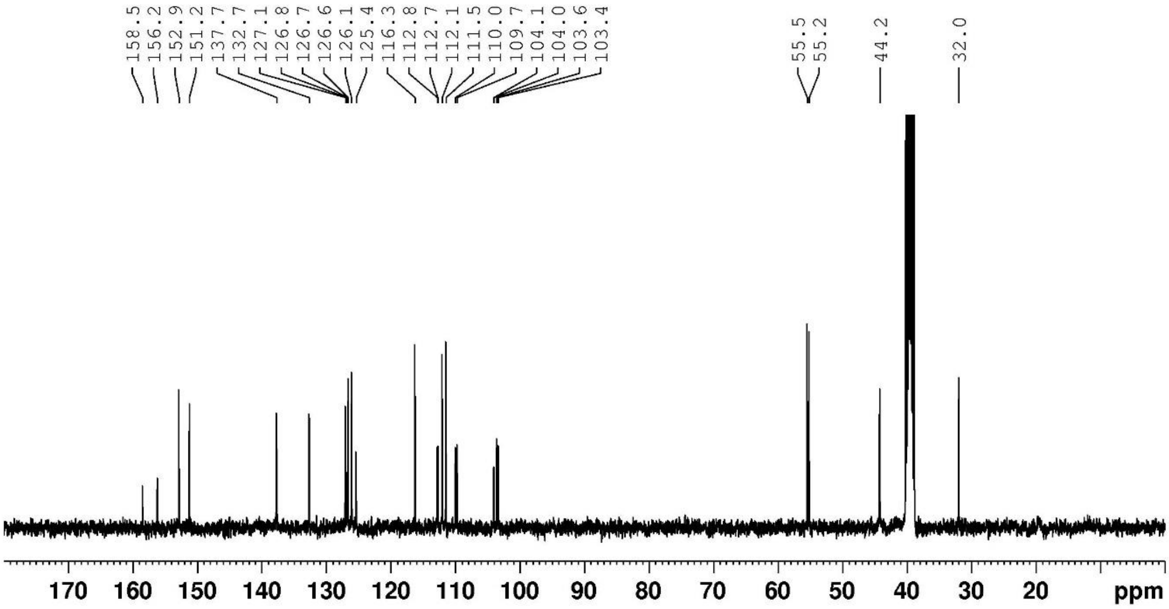

Figure S31. 3-(1-(2,5-Dimethoxyphenethyl)-1*H*-imidazol-5-yl)-6-fluoro-1*H*-indole (**31**)

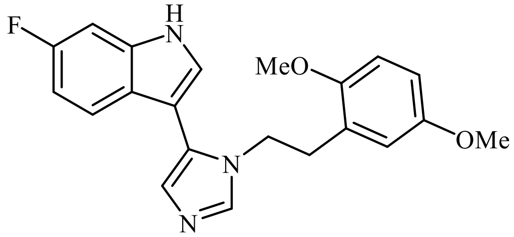

^{1}H NMR (DMSO-d_6, 400 MHz):

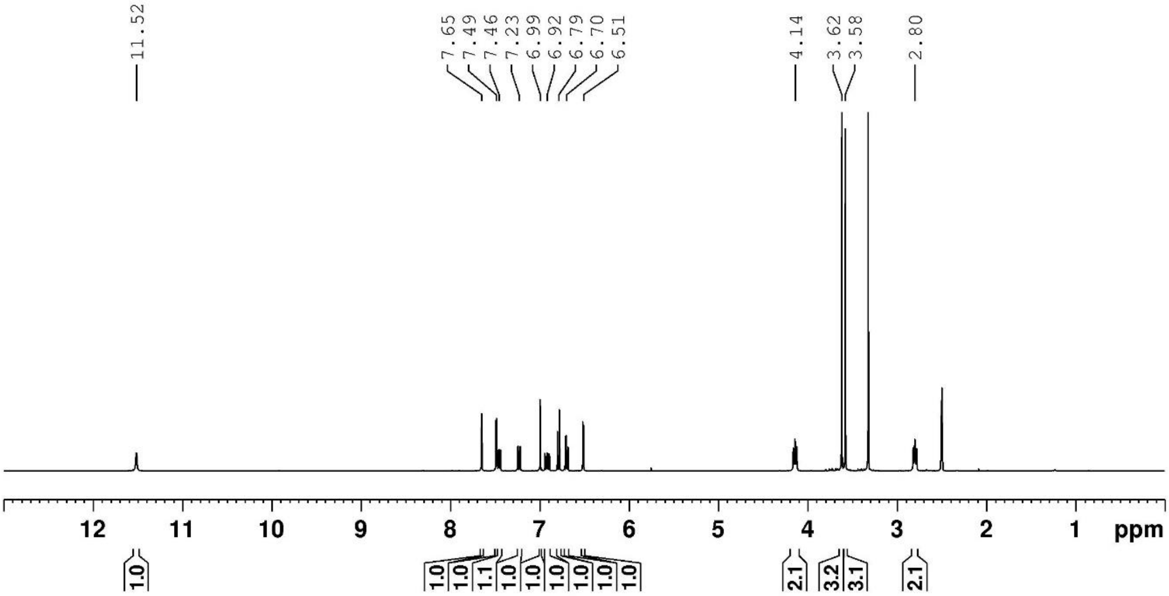

^{13}C NMR (DMSO-d_6, 100 MHz):

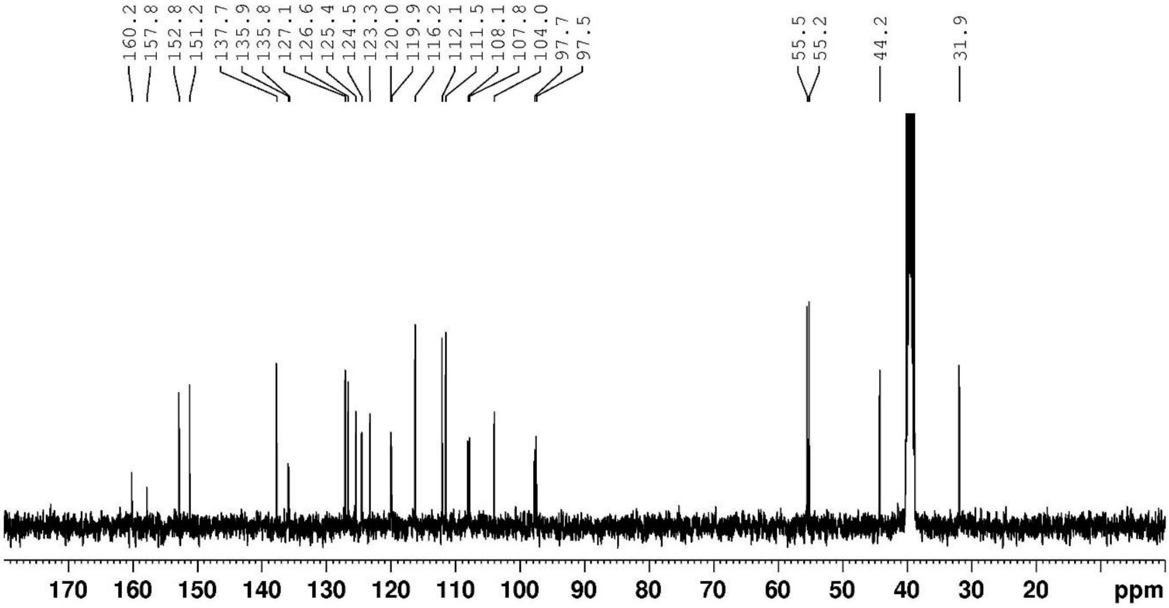

Figure S32. 5-Chloro-3-(1-(2,5-dimethoxyphenethyl)-1*H*-imidazol-5-yl)-1*H*-indole (**32**)

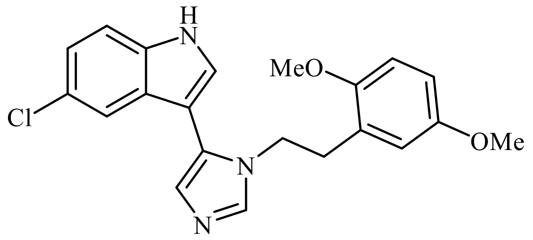

^{1}H NMR (DMSO-d_6, 400 MHz):

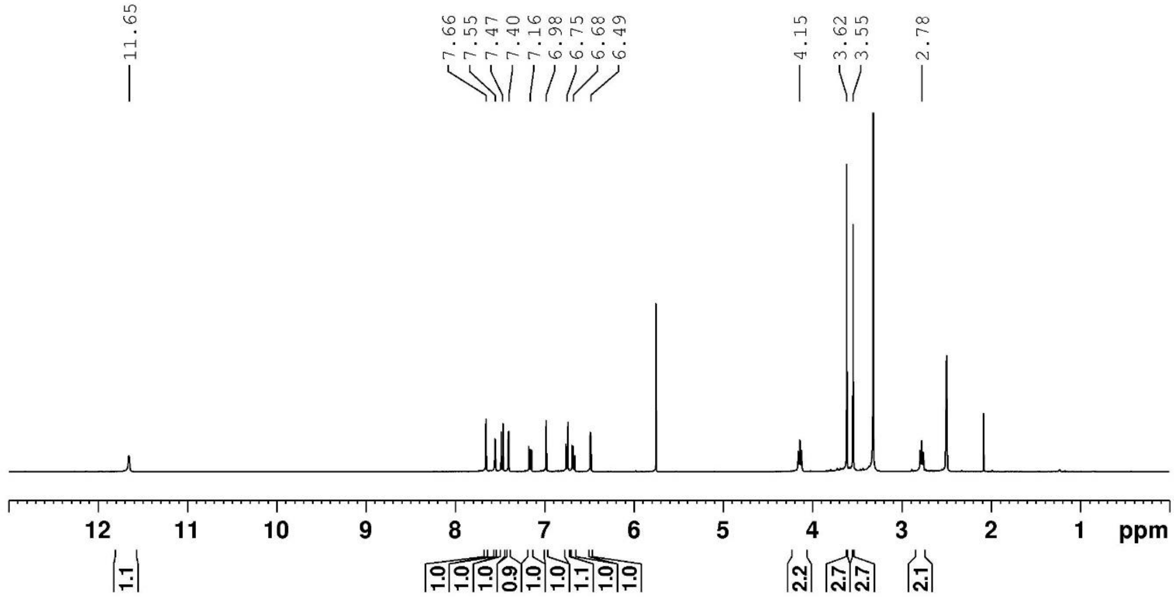

^{13}C NMR (DMSO-d_6, 100 MHz):

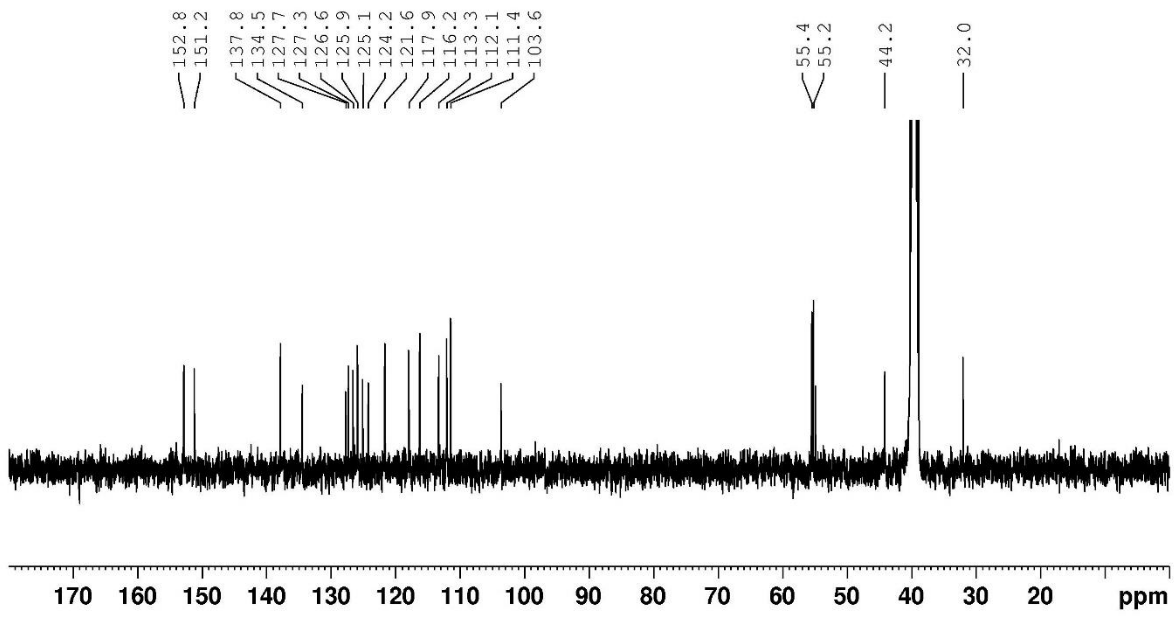

Figure S33. 6-Chloro-3-(1-(2,5-dimethoxyphenethyl)-1*H*-imidazol-5-yl)-1*H*-indole (**33**)

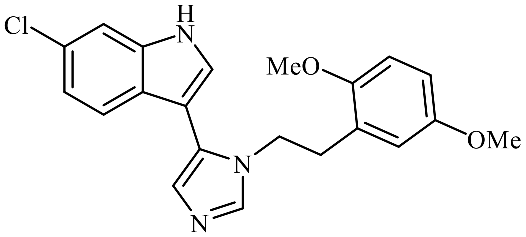

^{1}H NMR (DMSO-d_6, 400 MHz):

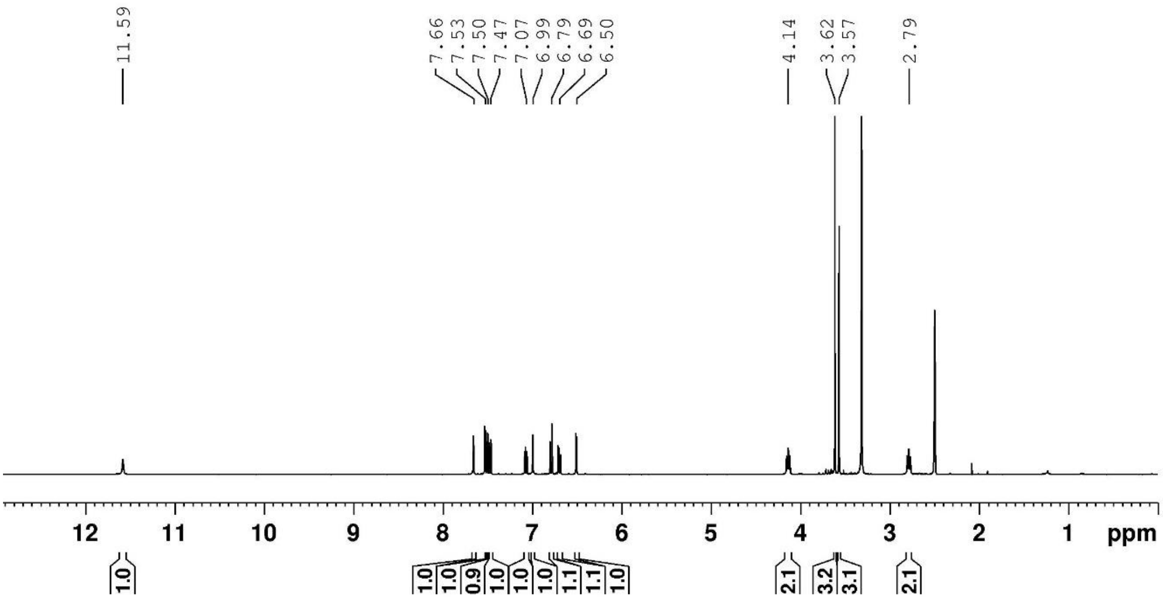

^{13}C NMR (DMSO-d_6, 100 MHz):

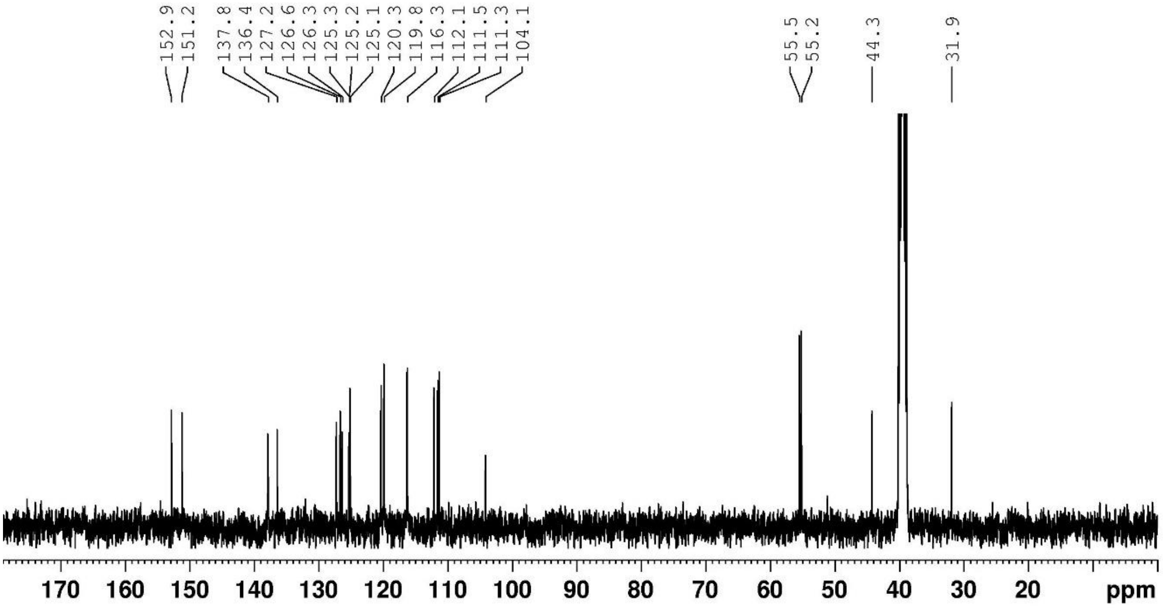

Figure S34. 5-Bromo-3-(1-(2,5-dimethoxyphenethyl)-1*H*-imidazol-5-yl)-1*H*-indole (**34**)

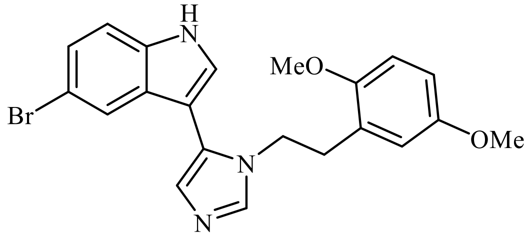

^{1}H NMR (DMSO-d_6, 400 MHz):

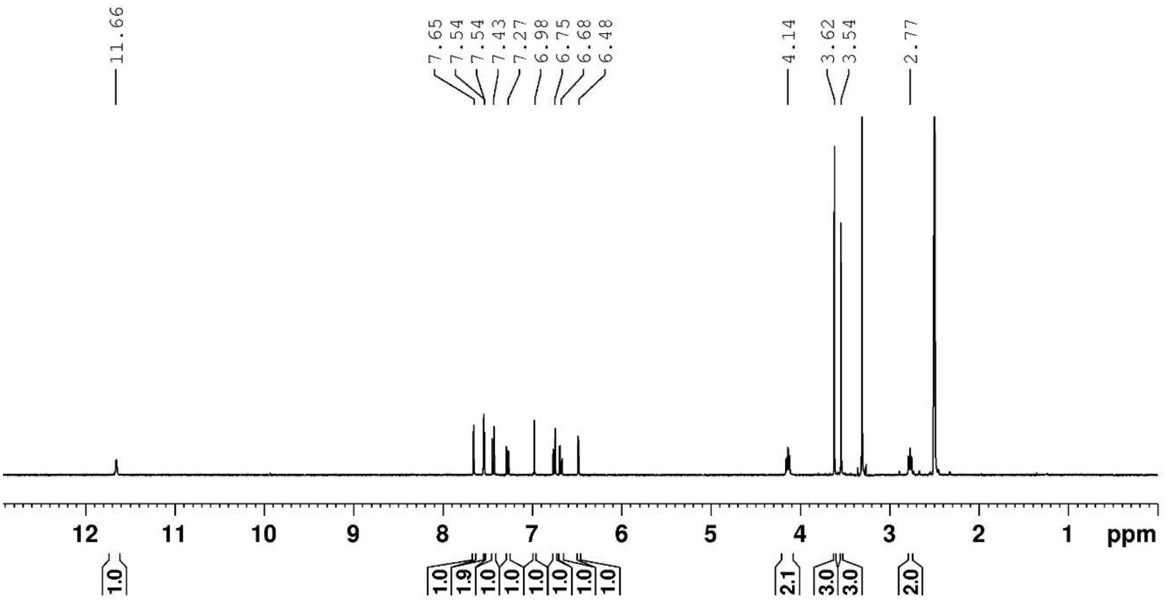

^{13}C NMR (DMSO-d_6, 100 MHz):

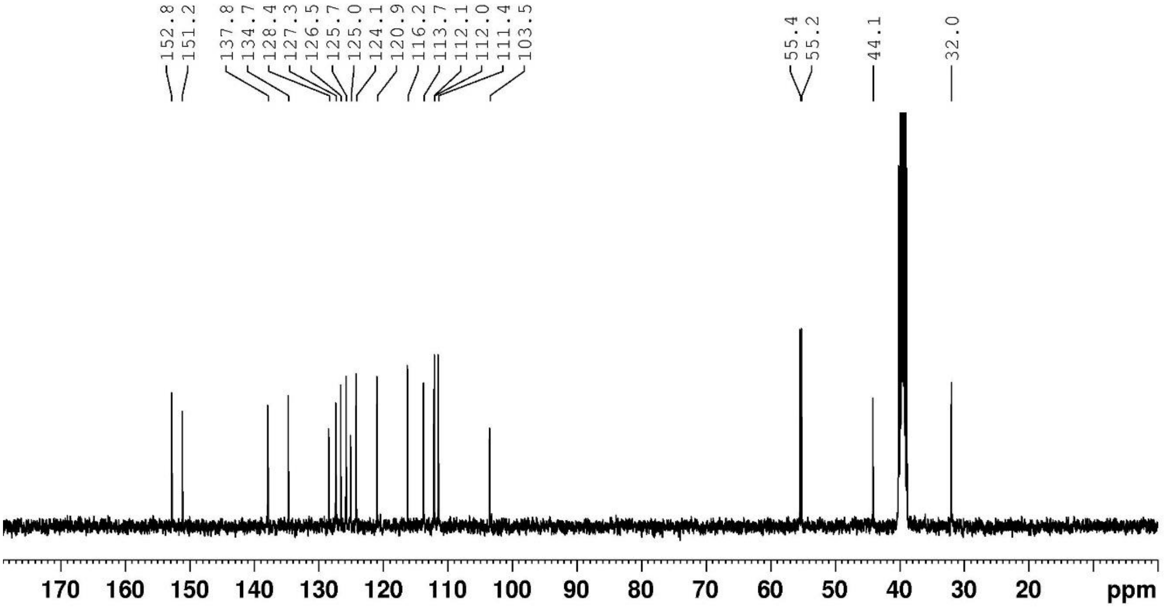

Figure S35. 6-Bromo-3-(1-(2,5-dimethoxyphenethyl)-1*H*-imidazol-5-yl)-1*H*-indole (**35**)

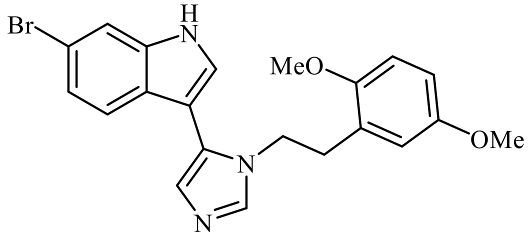

^{1}H NMR (DMSO-d_6, 400 MHz):

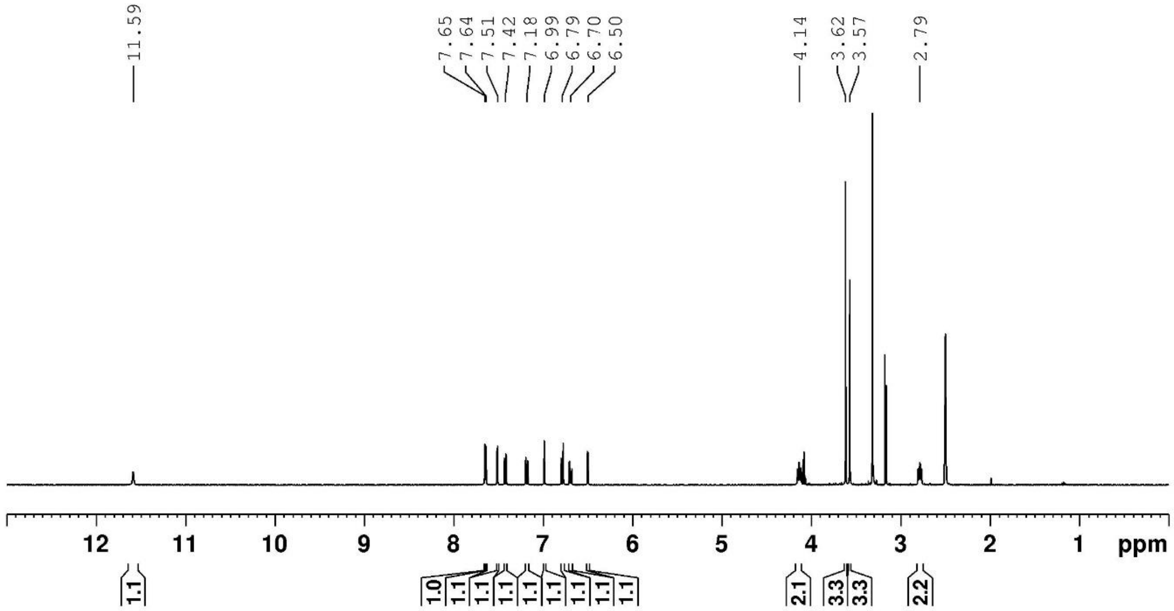

^{13}C NMR (DMSO-d_6, 100 MHz):

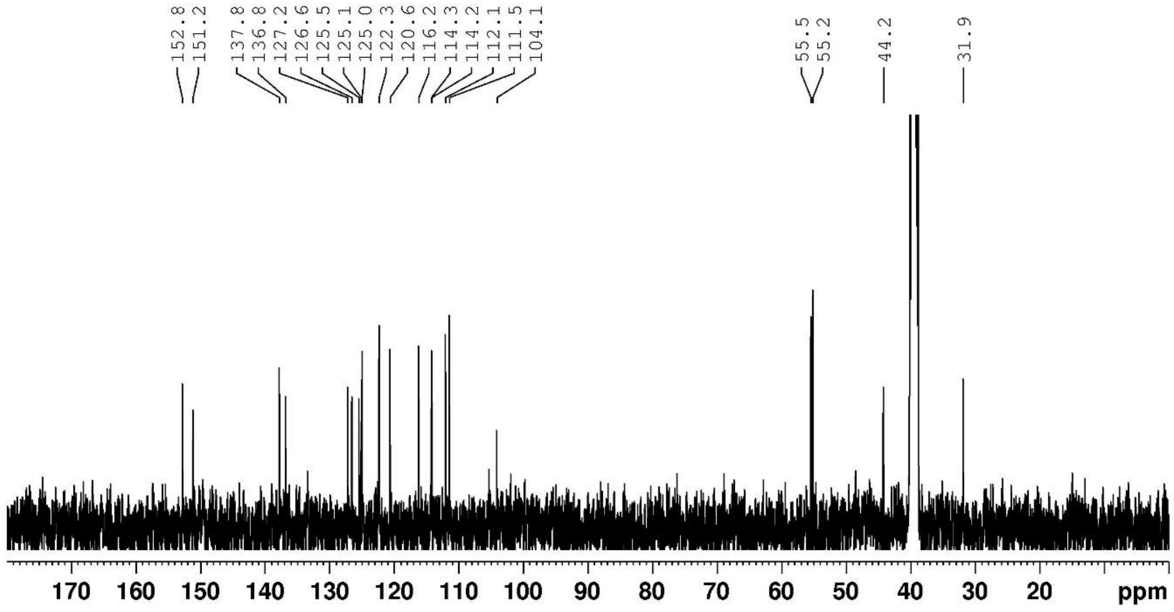

Figure S36. 3-(1-(2-(Benzo[*d*][1,3]dioxol-5-yl)ethyl)-1*H*-imidazol-5-yl)-1*H*-indole (**36**)

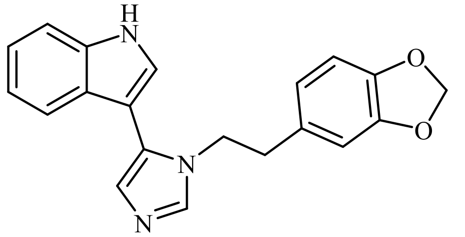

^{1}H NMR (DMSO-d_6, 400 MHz):

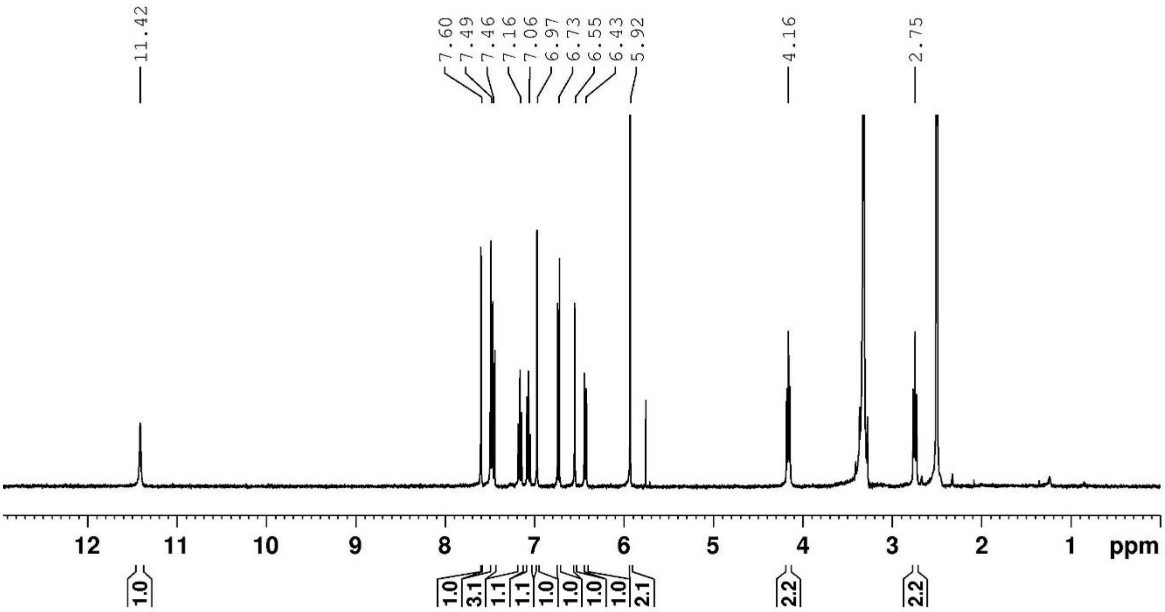

^{13}C NMR (DMSO-d_6, 100 MHz):

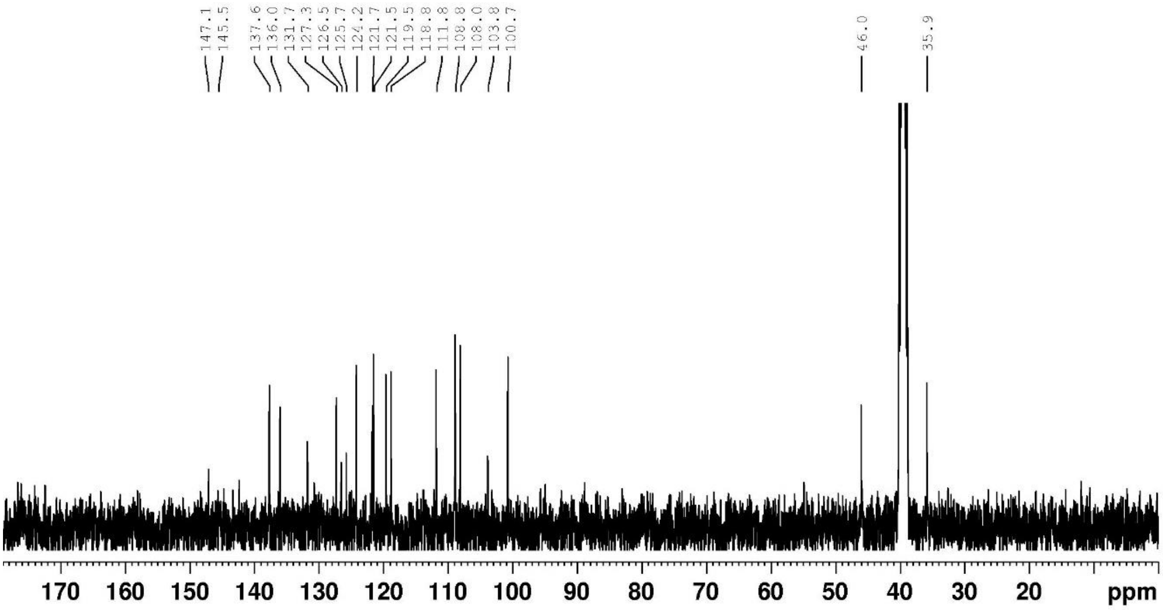

Figure S37. 3-(1-(2-(Benzo[*d*][1,3]dioxol-5-yl)ethyl)-1*H*-imidazol-5-yl)-5-fluoro-1*H*-indole (**37**)

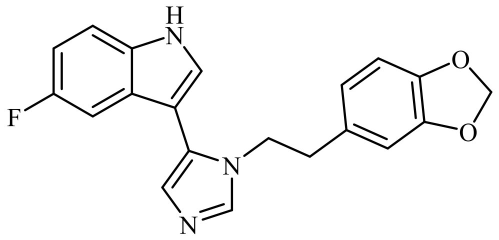

^{1}H NMR (DMSO-d_6, 400 MHz):

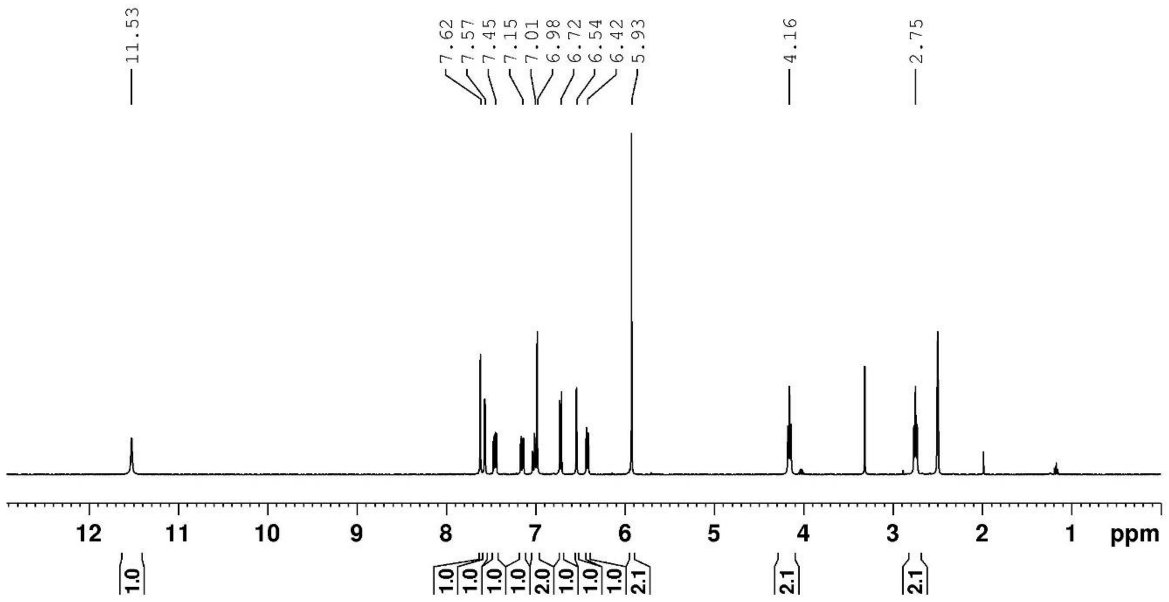

^{13}C NMR (DMSO-d_6, 100 MHz):

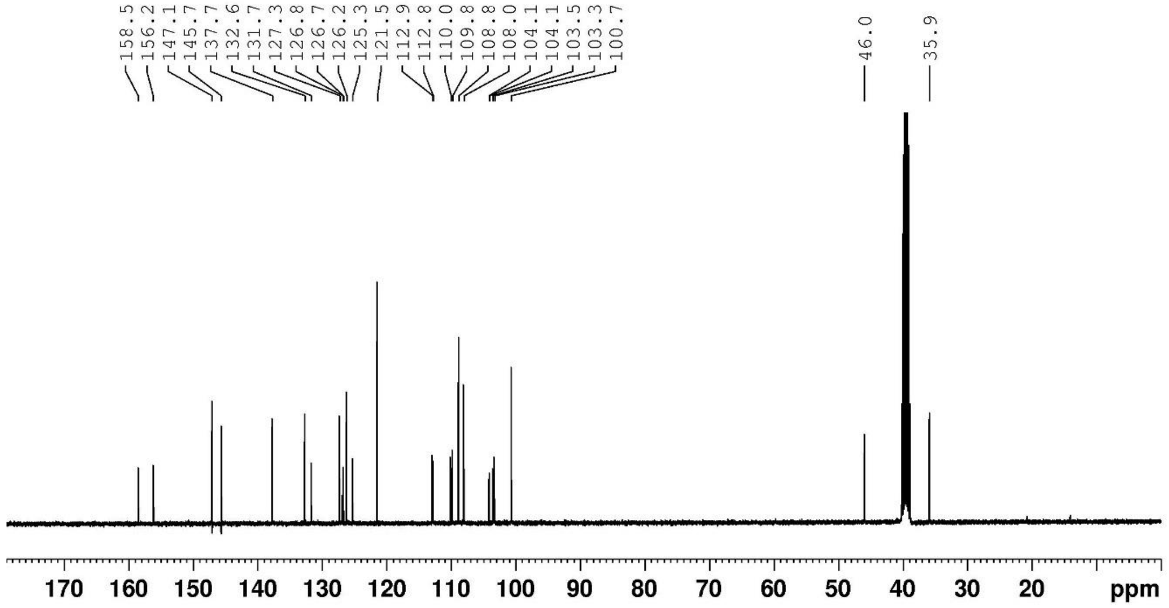

Figure S38. 3-(1-(2-(Benzo[*d*][1,3]dioxol-5-yl)ethyl)-1*H*-imidazol-5-yl)-6-fluoro-1*H*-indole (**38**)

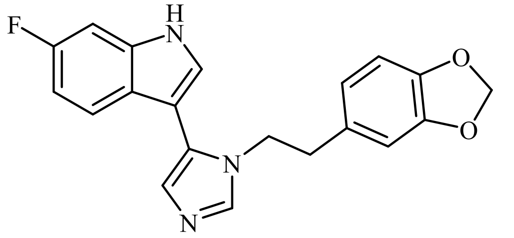

^{1}H NMR (DMSO-d_6, 400 MHz):

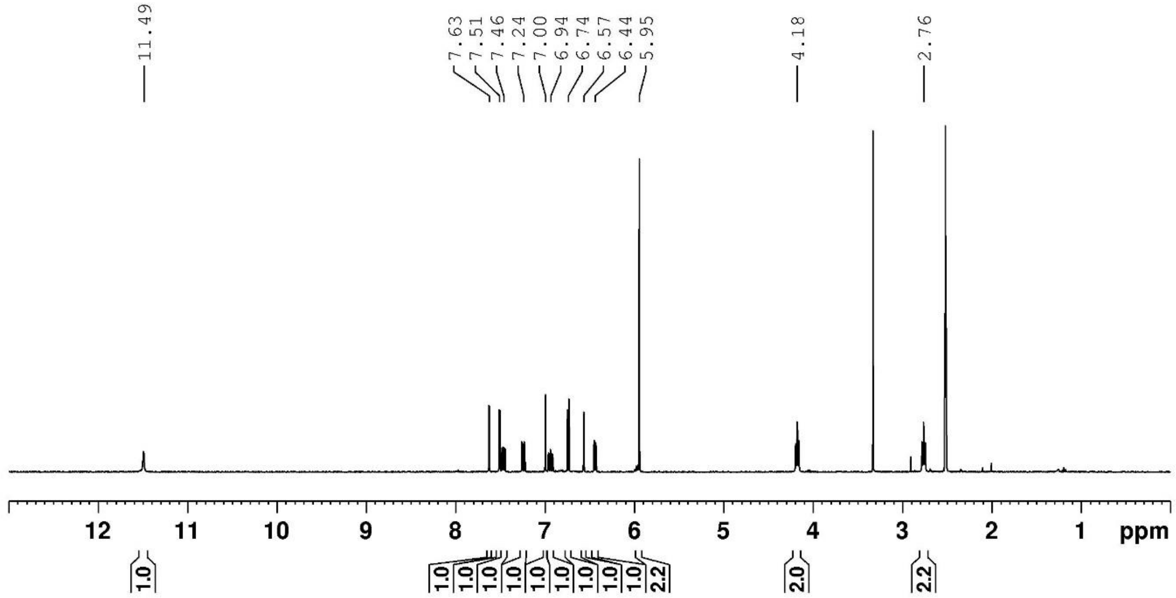

^{13}C NMR (DMSO-d_6, 100 MHz):

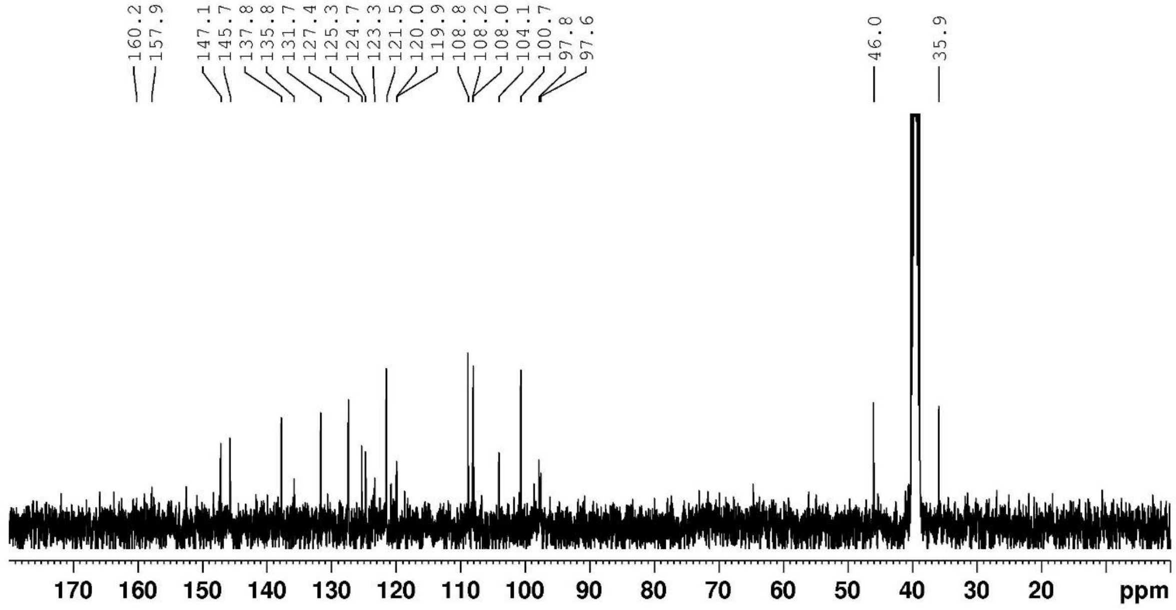

Figure S39. 3-(1-(2-(Benzo[*d*][1,3]dioxol-5-yl)ethyl)-1*H*-imidazol-5-yl)-5-chloro-1*H*-indole (**39**)

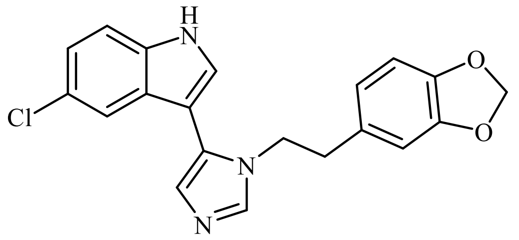

^{1}H NMR (DMSO-d_6, 400 MHz):

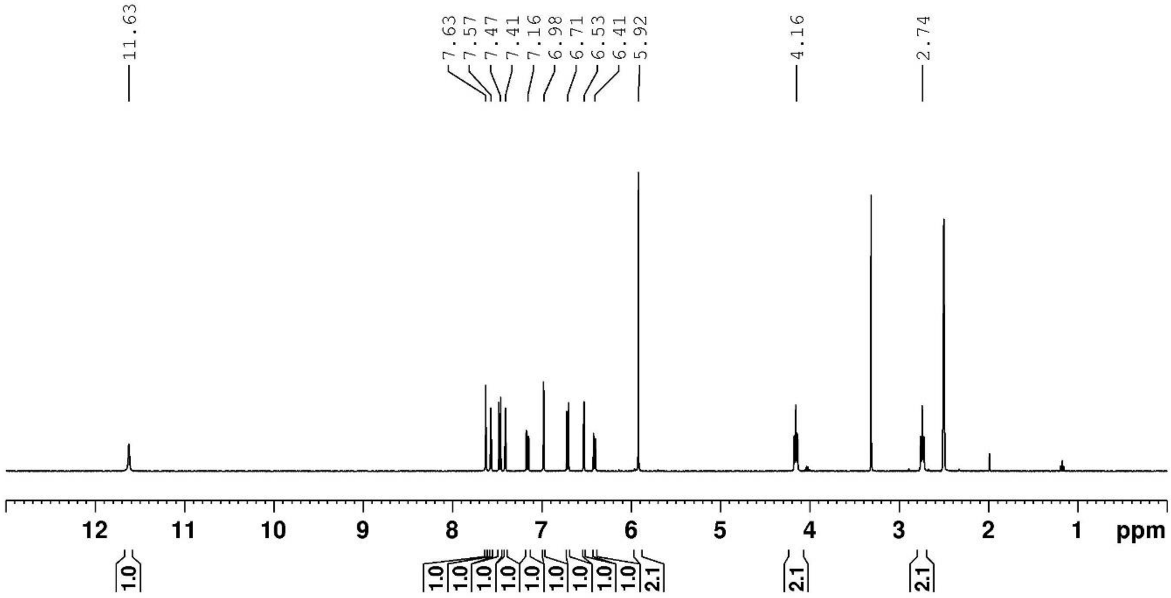

^{13}C NMR (DMSO-d_6, 100 MHz):

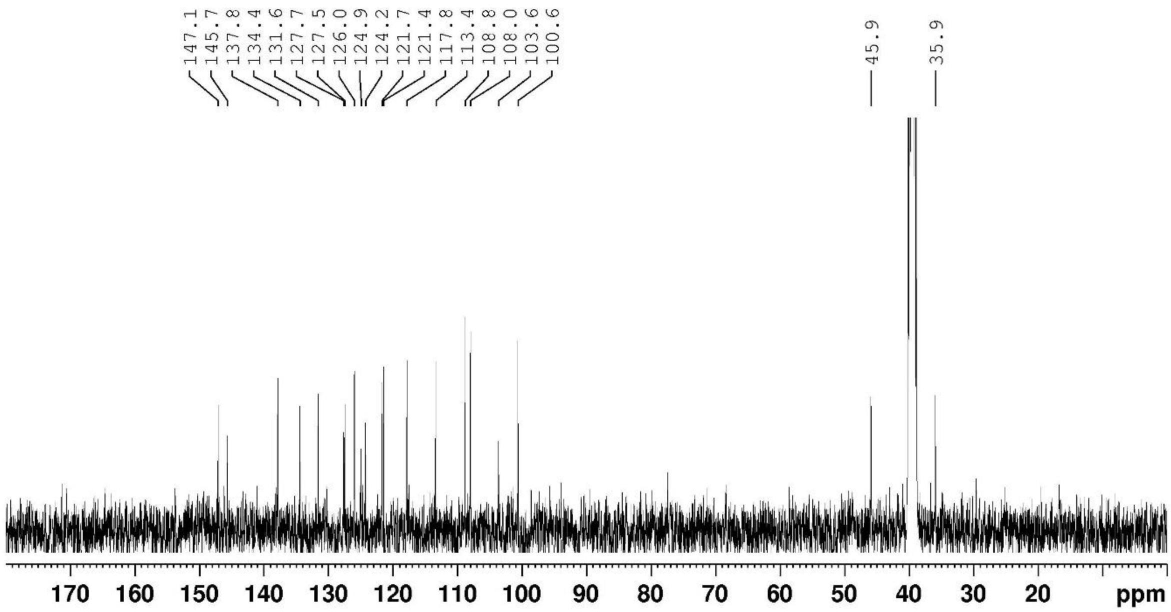

Figure S40. 3-(1-(2-(Benzo[*d*][1,3]dioxol-5-yl)ethyl)-1*H*-imidazol-5-yl)-6-chloro-1*H*-indole (**40**)

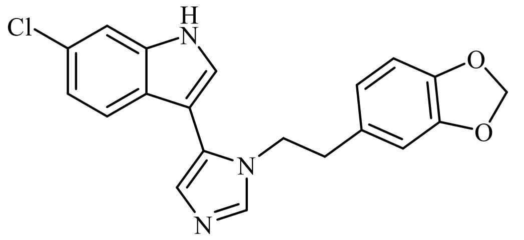

^{1}H NMR (DMSO-d_6, 400 MHz):

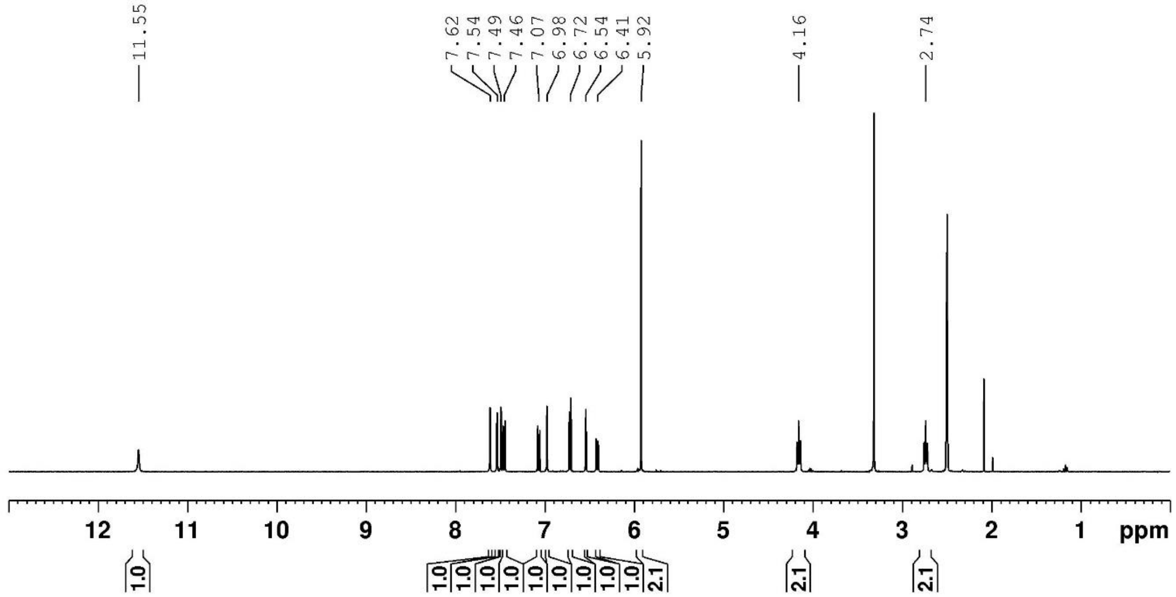

^{13}C NMR (DMSO-d_6, 100 MHz):

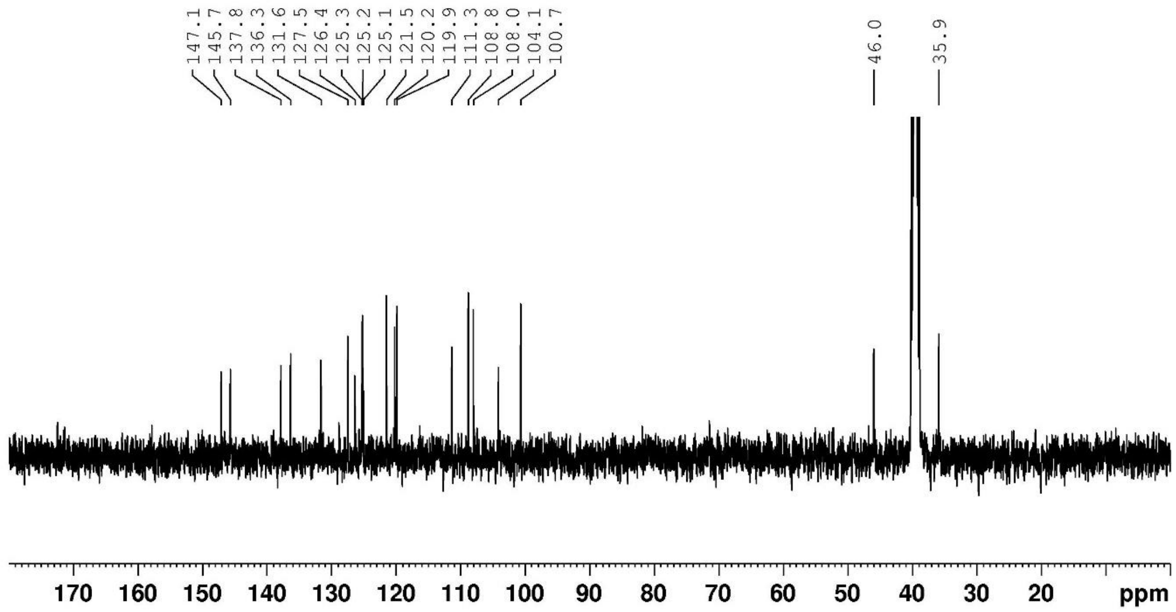

Figure S41. 3-(1-(2-(Benzo[*d*][1,3]dioxol-5-yl)ethyl)-1*H*-imidazol-5-yl)-5-bromo-1*H*-indole (**41**)

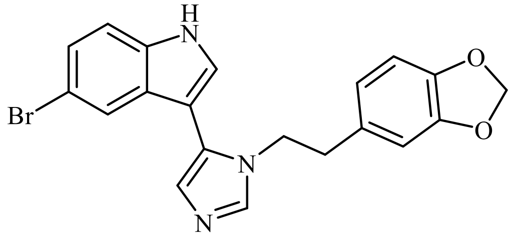

^{1}H NMR (DMSO-d_6, 400 MHz):

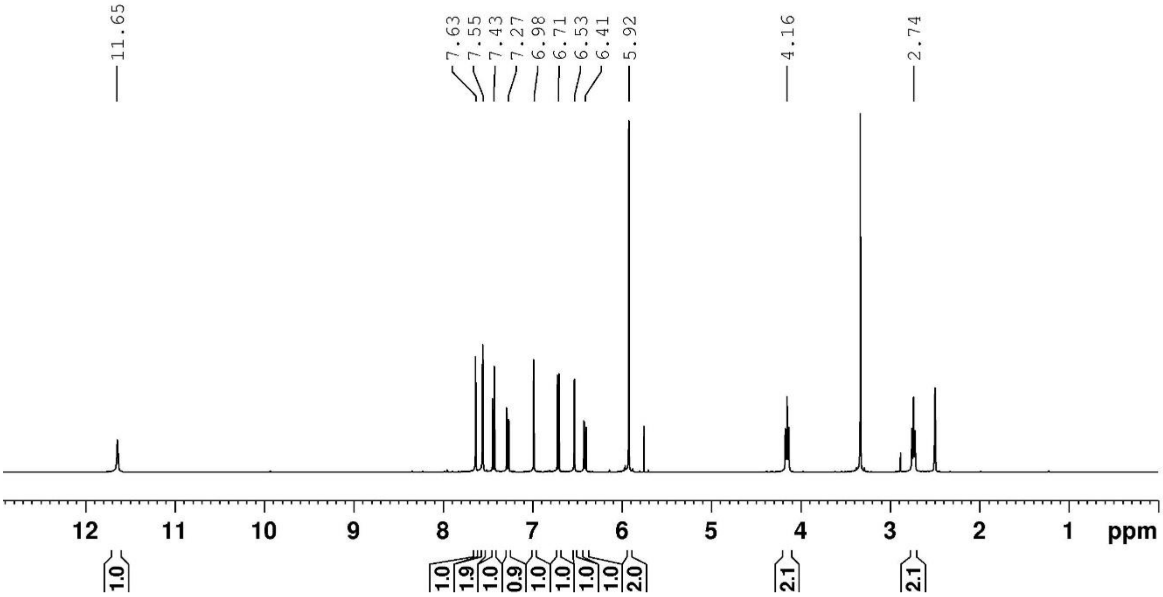

^{13}C NMR (DMSO-d_6, 100 MHz):

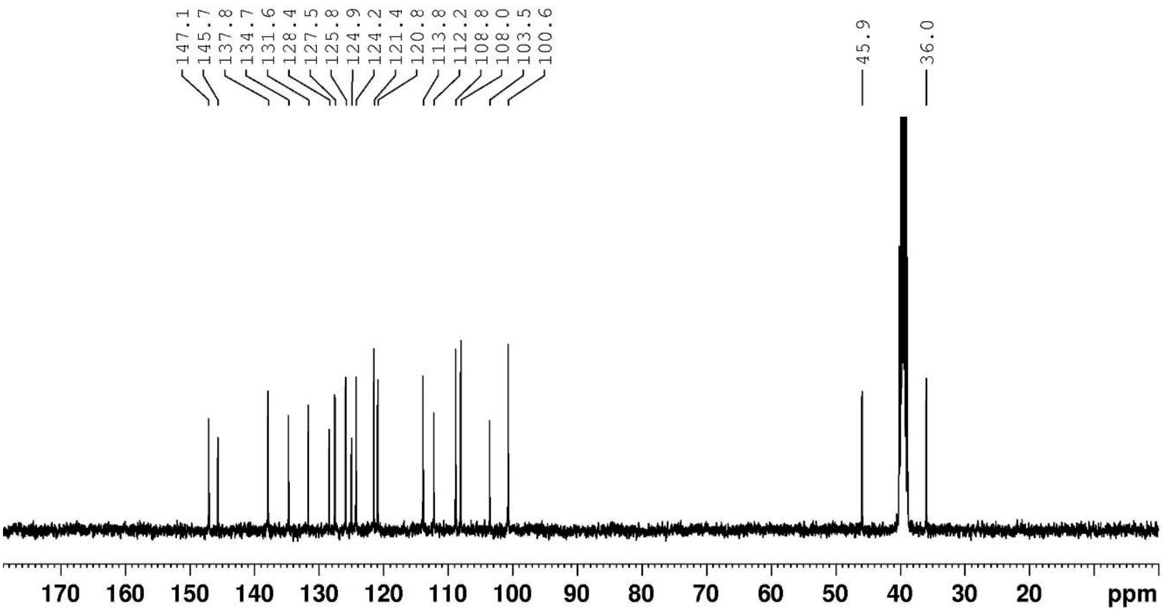

Figure S42. 3-(1-(2-(Benzo[*d*][1,3]dioxol-5-yl)ethyl)-1*H*-imidazol-5-yl)-6-bromo-1*H*-indole (**42**)

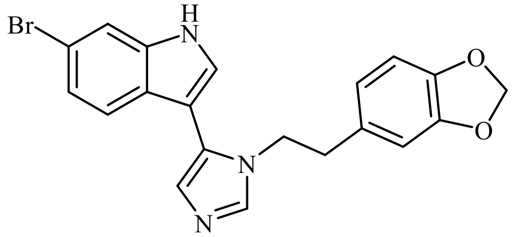

^{1}H NMR (DMSO-d_6, 400 MHz):

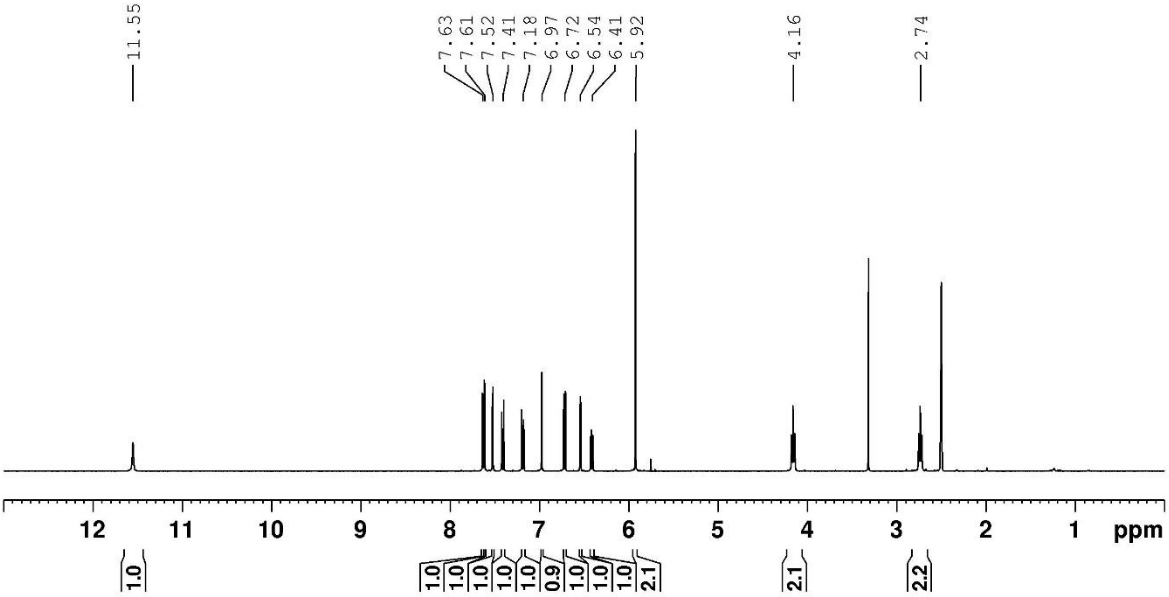

^{13}C NMR (DMSO-d_6, 100 MHz):

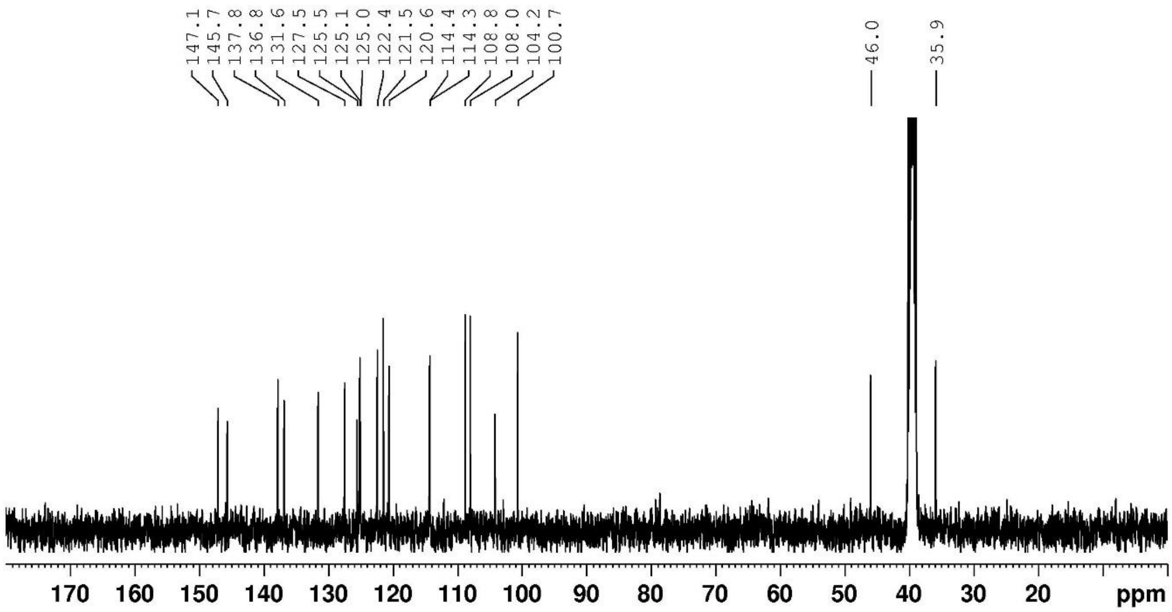

Figure S43. 3-(1-Benzyl-1*H*-imidazol-5-yl)-4-fluoro-1*H*-indole (**43**)

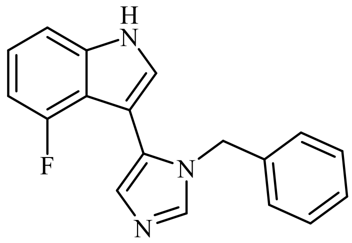

^{1}H NMR ($CDCl_3$, 400 MHz):

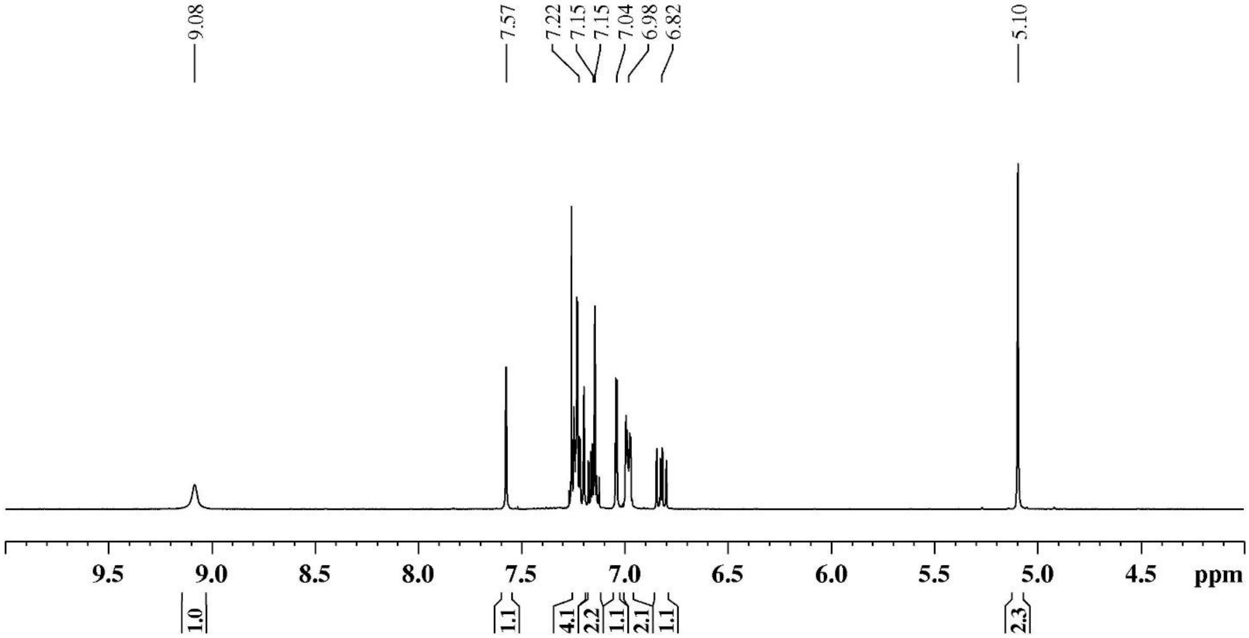

^{13}C NMR ($CDCl_3$, 100 MHz):

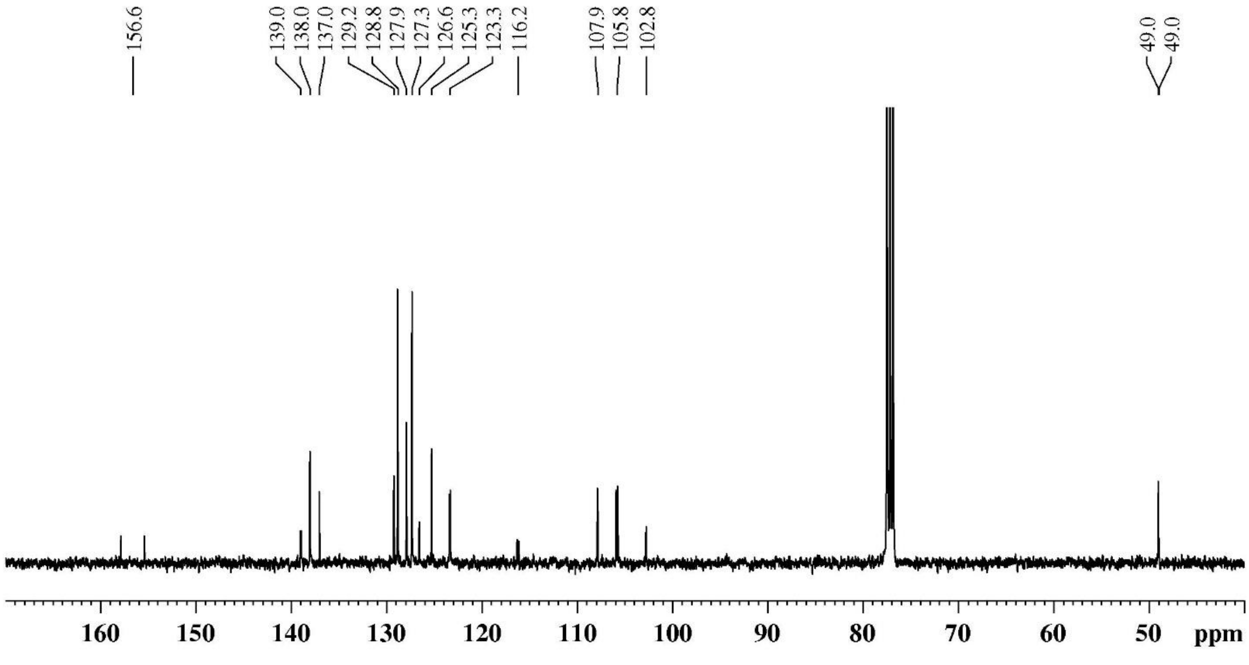

Figure S44. 3-(1-Benzyl-1*H*-imidazol-5-yl)-7-fluoro-1*H*-indole (**44**)

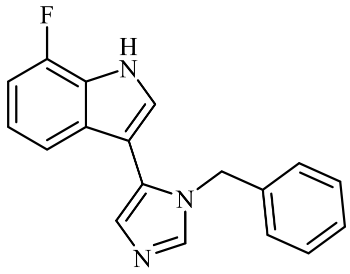

^{1}H NMR ($CDCl_3$, 500 MHz):

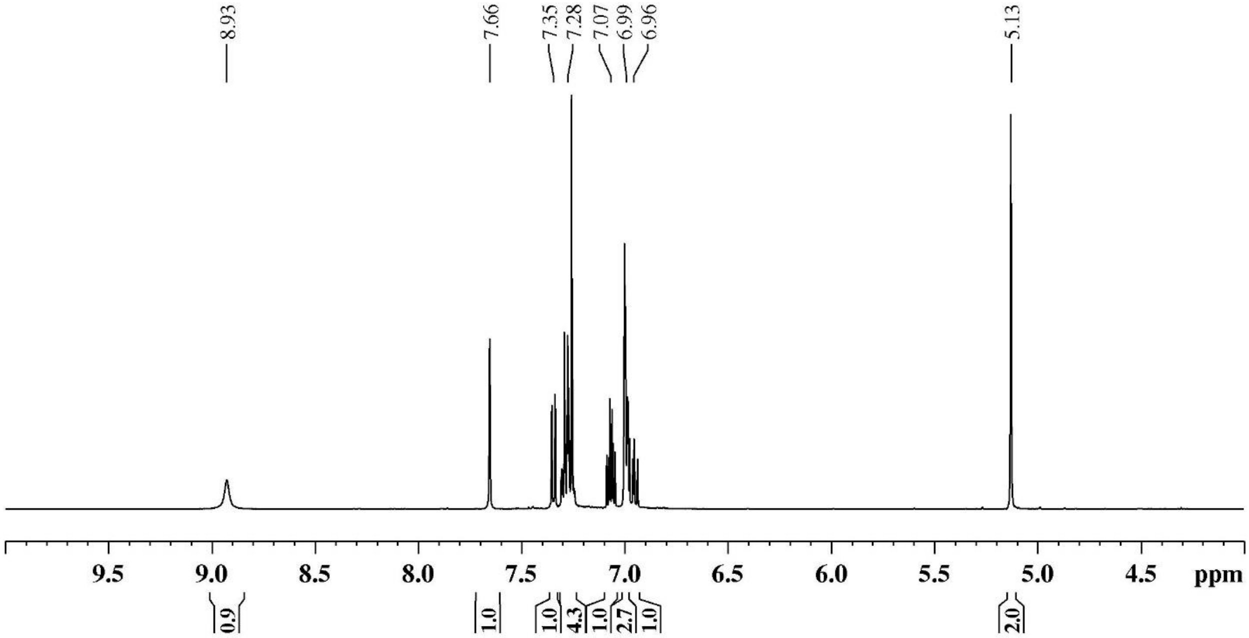

^{13}C NMR ($CDCl_3$, 125 MHz):

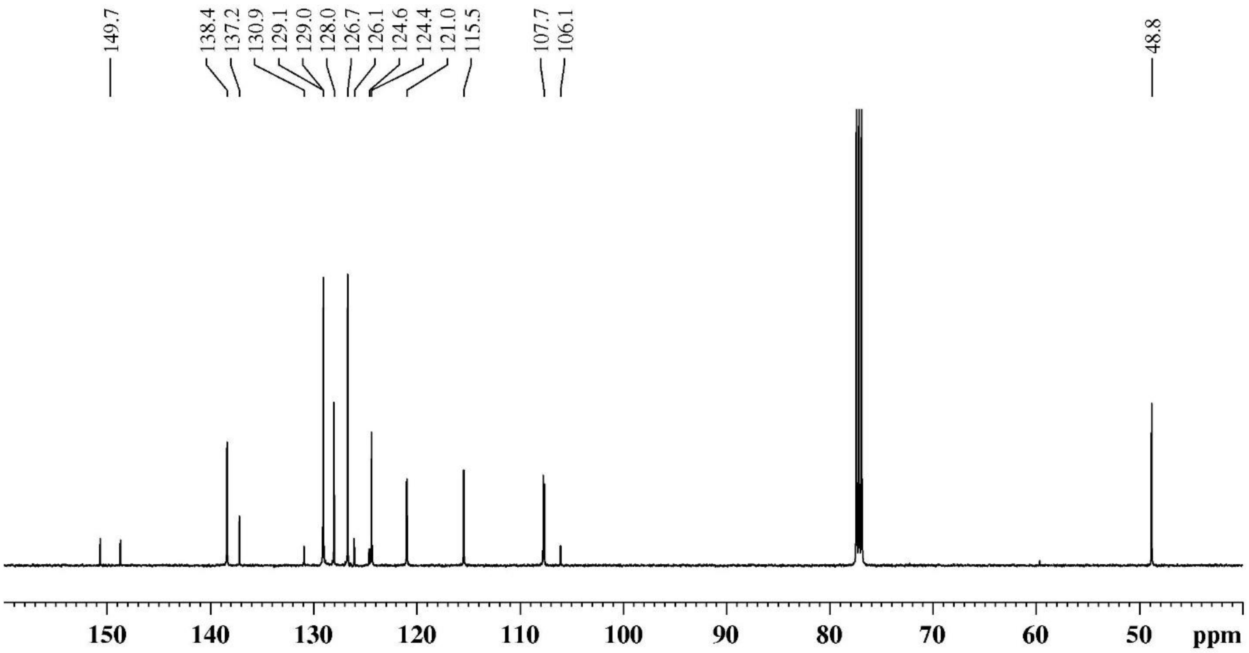

Figure S45. 3-(1-Benzyl-1*H*-imidazol-5-yl)-4-chloro-1*H*-indole (**45**)

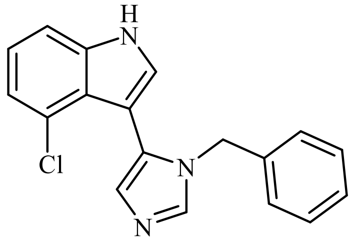

^{1}H NMR ($CDCl_3$, 400 MHz):

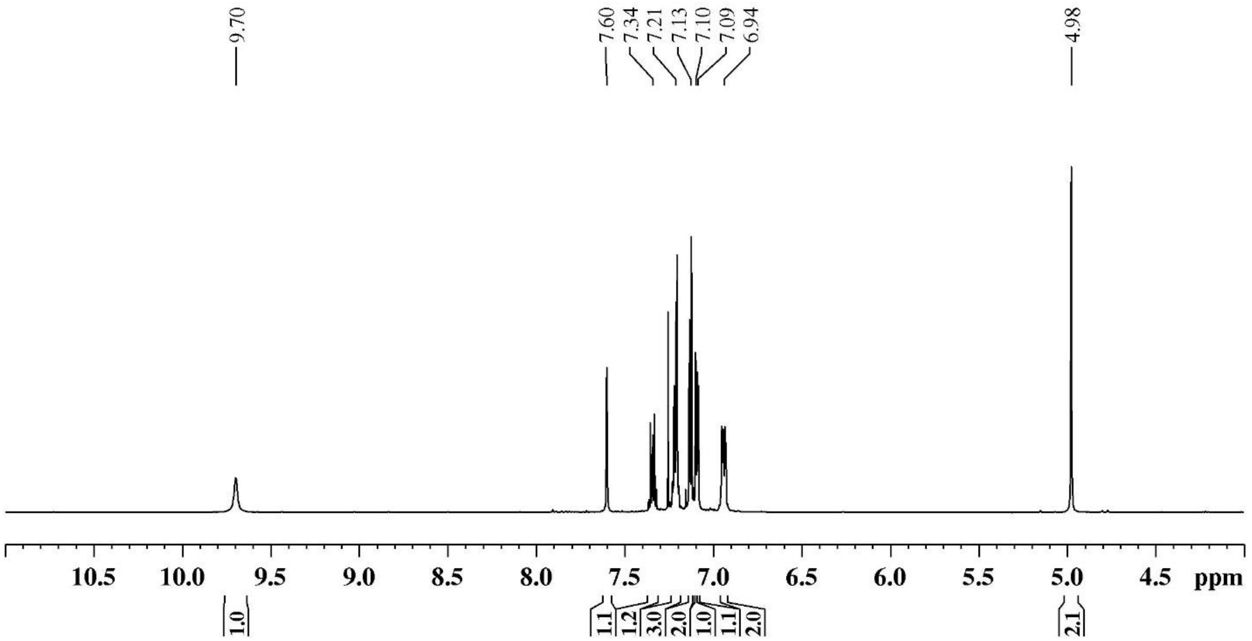

^{13}C NMR ($CDCl_3$, 100 MHz):

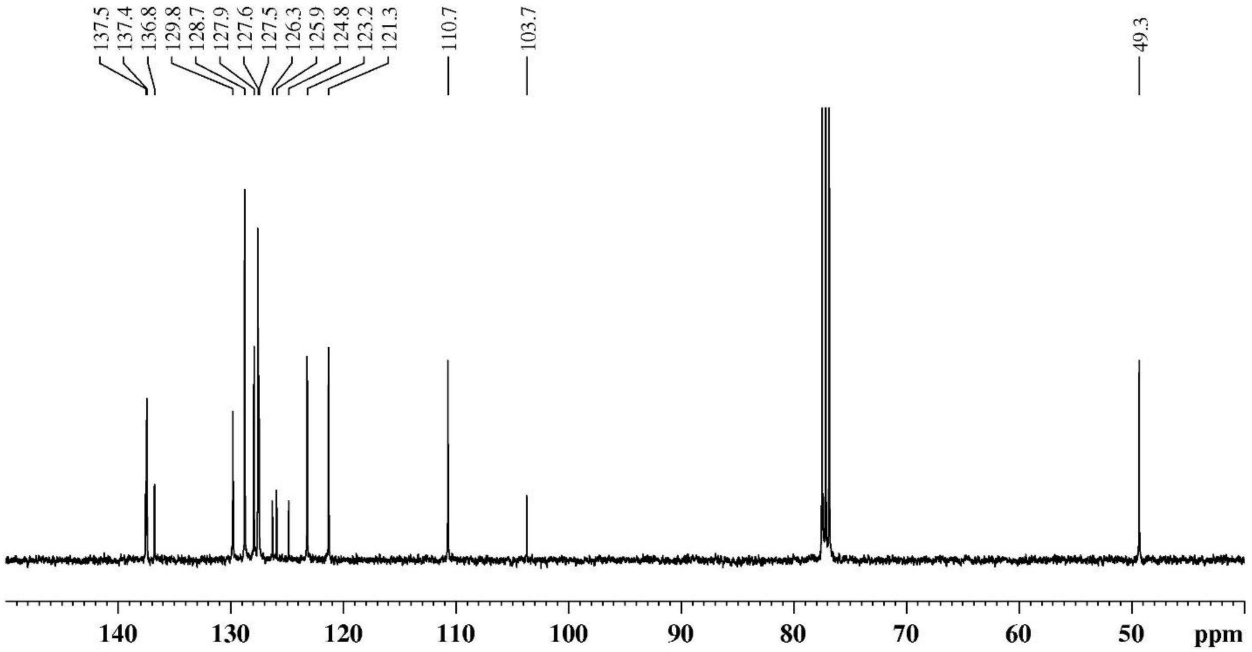

Figure S46. 3-(1-Benzyl-1*H*-imidazol-5-yl)-5-chloro-1*H*-indole (**46**)

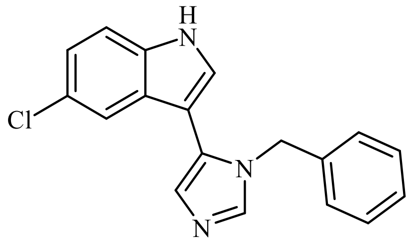

^{1}H NMR ($CDCl_3$, 400 MHz):

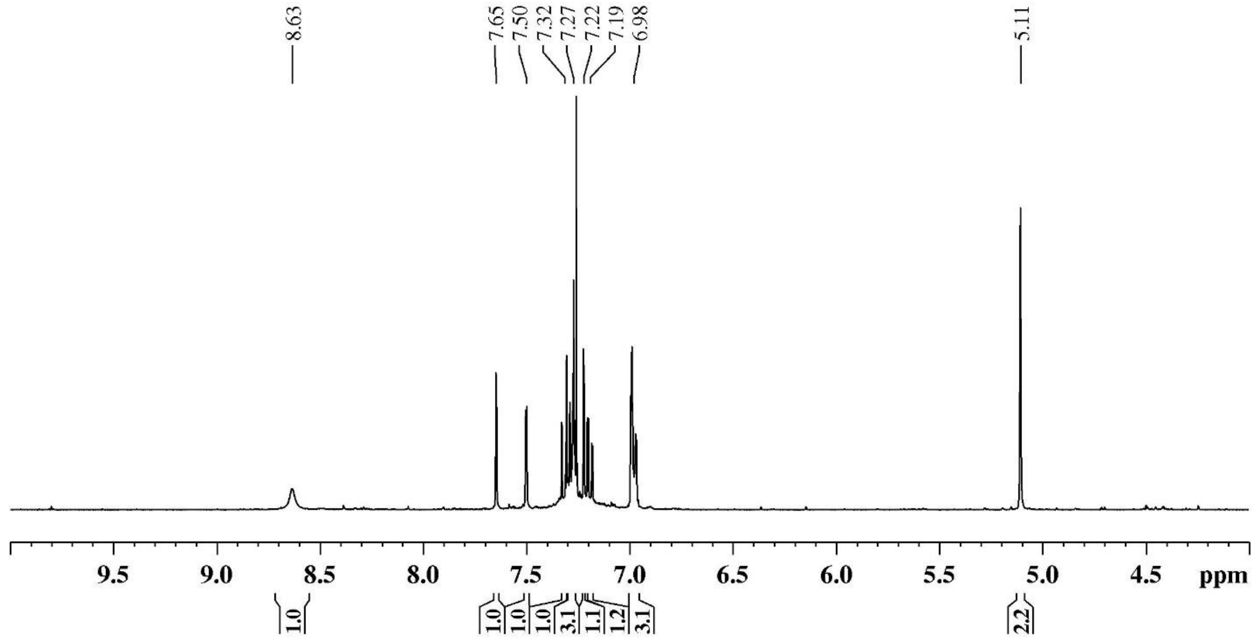

^{13}C NMR ($CDCl_3$, 100 MHz):

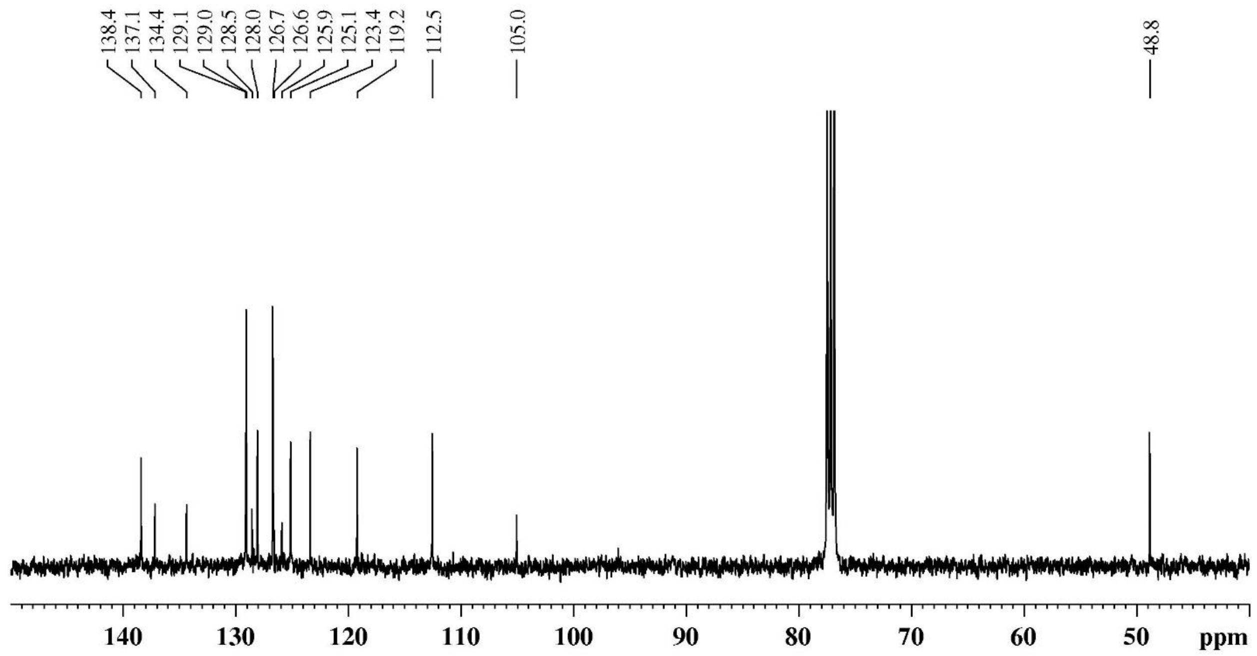

Figure S47. 3-(1-Benzyl-1*H*-imidazol-5-yl)-7-chloro-1*H*-indole (**47**)

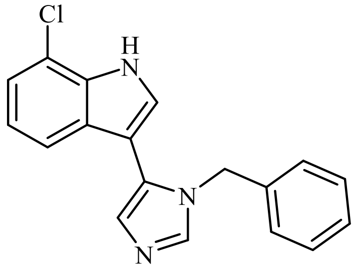

^{1}H NMR ($CDCl_3$, 400 MHz):

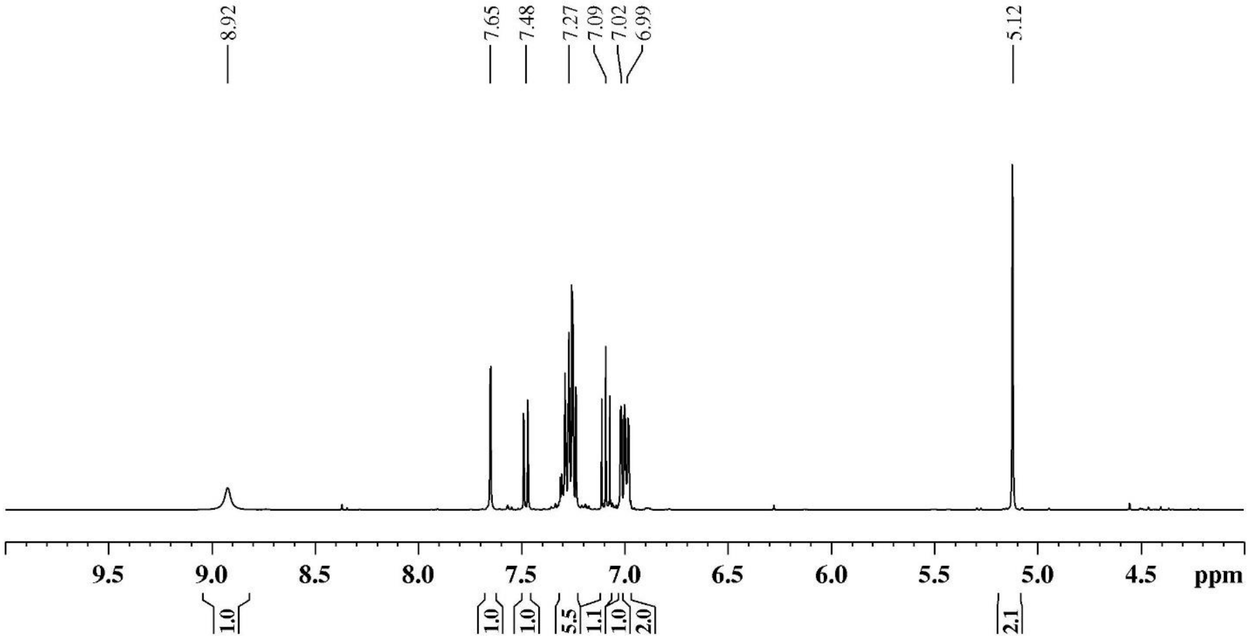

^{13}C NMR ($CDCl_3$, 100 MHz):

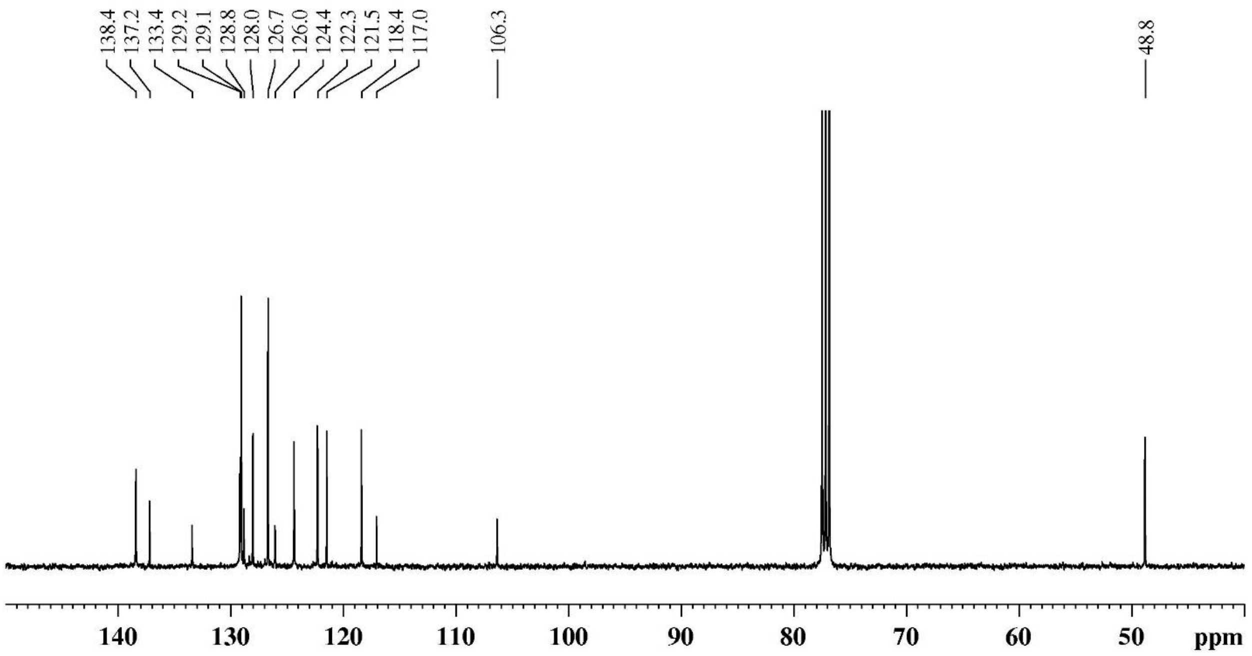

Figure S48. 3-(1-Benzyl-1*H*-imidazol-5-yl)-4-bromo-1*H*-indole (**48**)

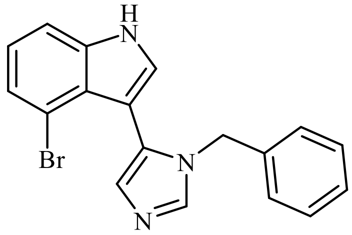

^{1}H NMR ($CDCl_3$, 400 MHz):

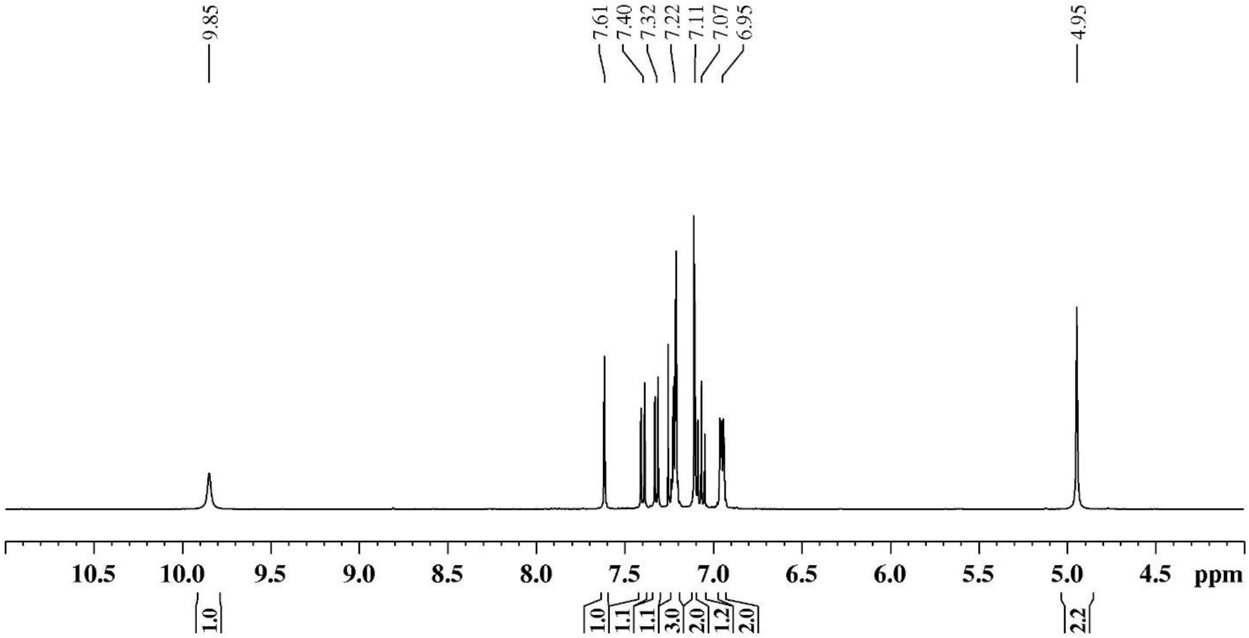

^{13}C NMR ($CDCl_3$, 100 MHz):

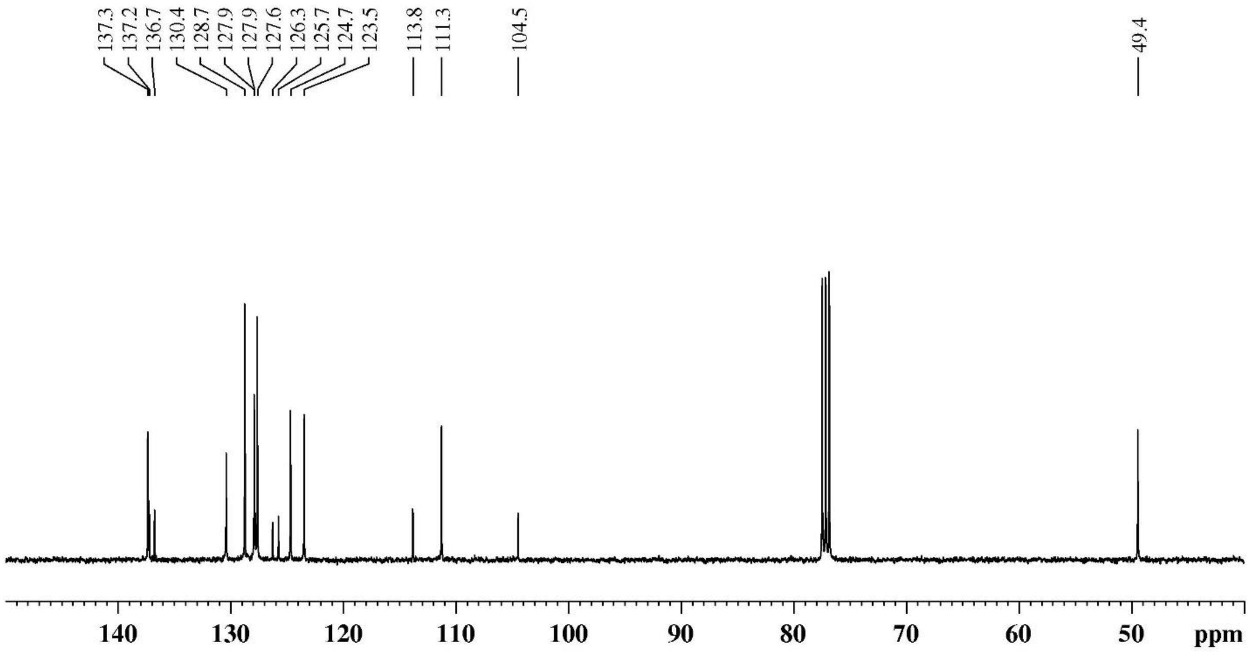

Figure S49. 3-(1-Benzyl-1*H*-imidazol-5-yl)-5-methoxy-1*H*-indole (**49**)

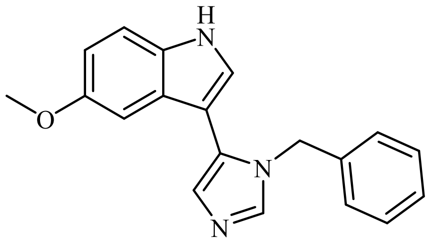

^{1}H NMR ($CDCl_3$, 400 MHz):

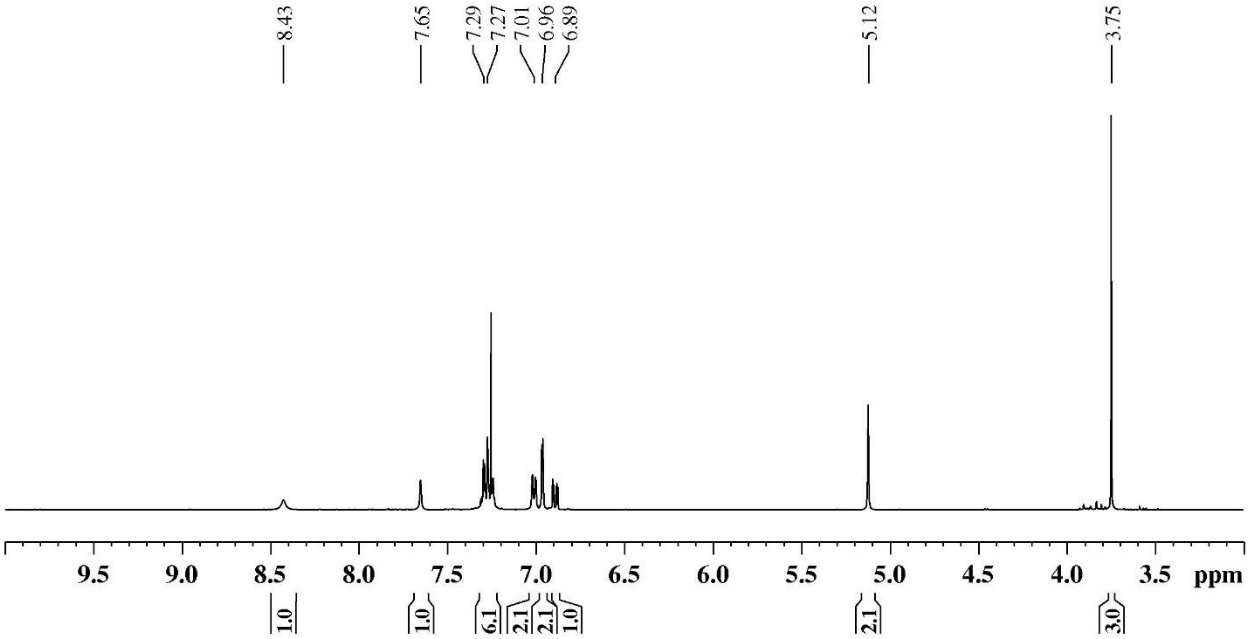

^{13}C NMR ($CDCl_3$, 100 MHz):

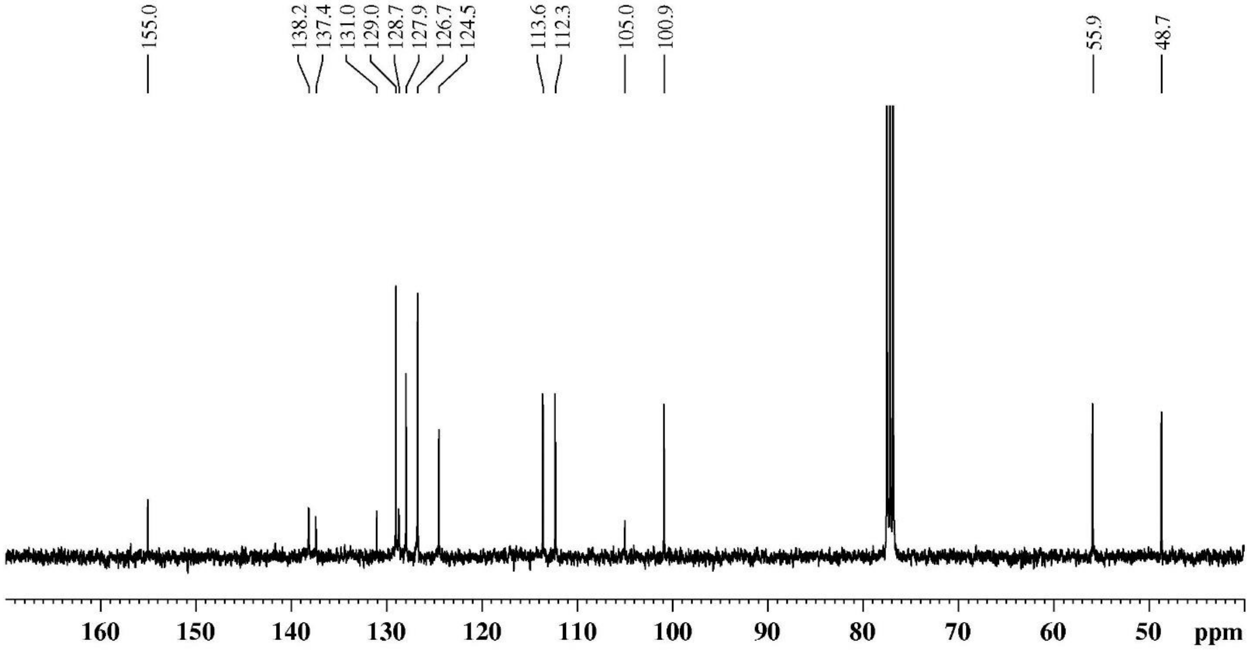

Figure S50. 3-(1-Benzyl-1*H*-imidazol-5-yl)-6-methoxy-1*H*-indole (**50**)

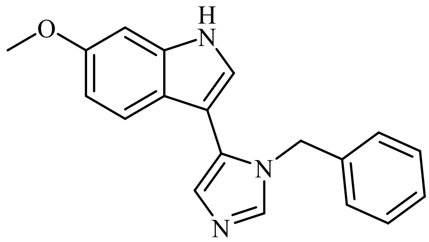

^{1}H NMR ($CDCl_3$, 400 MHz):

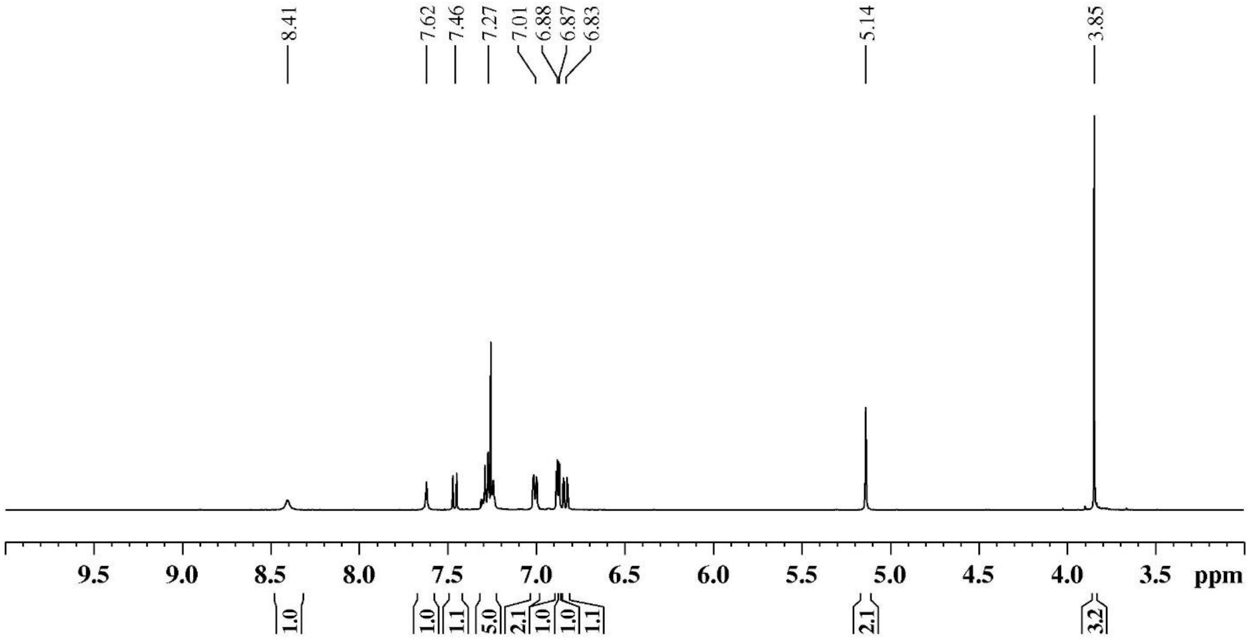

^{13}C NMR ($CDCl_3$, 100 MHz):

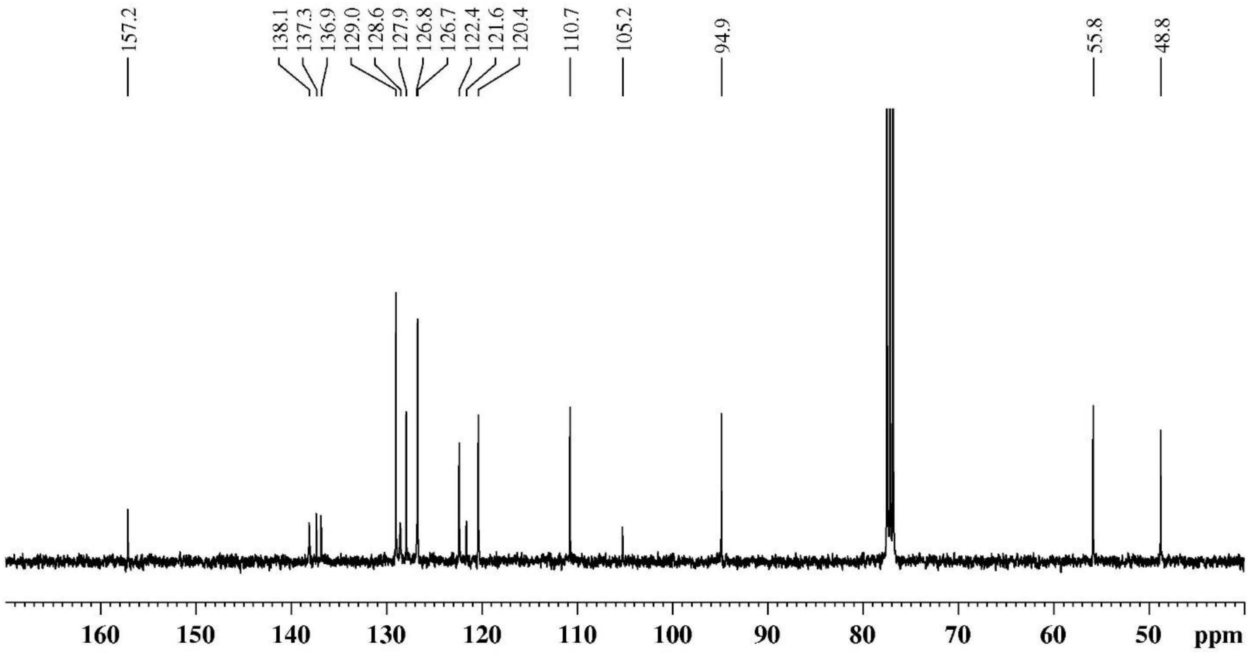

Figure S51. 4-Fluoro-3-(1-phenethyl-1*H*-imidazol-5-yl)-1*H*-indole (**51**)

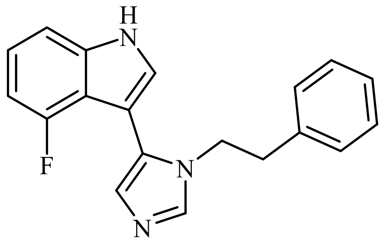

^{1}H NMR ($CDCl_3$, 400 MHz):

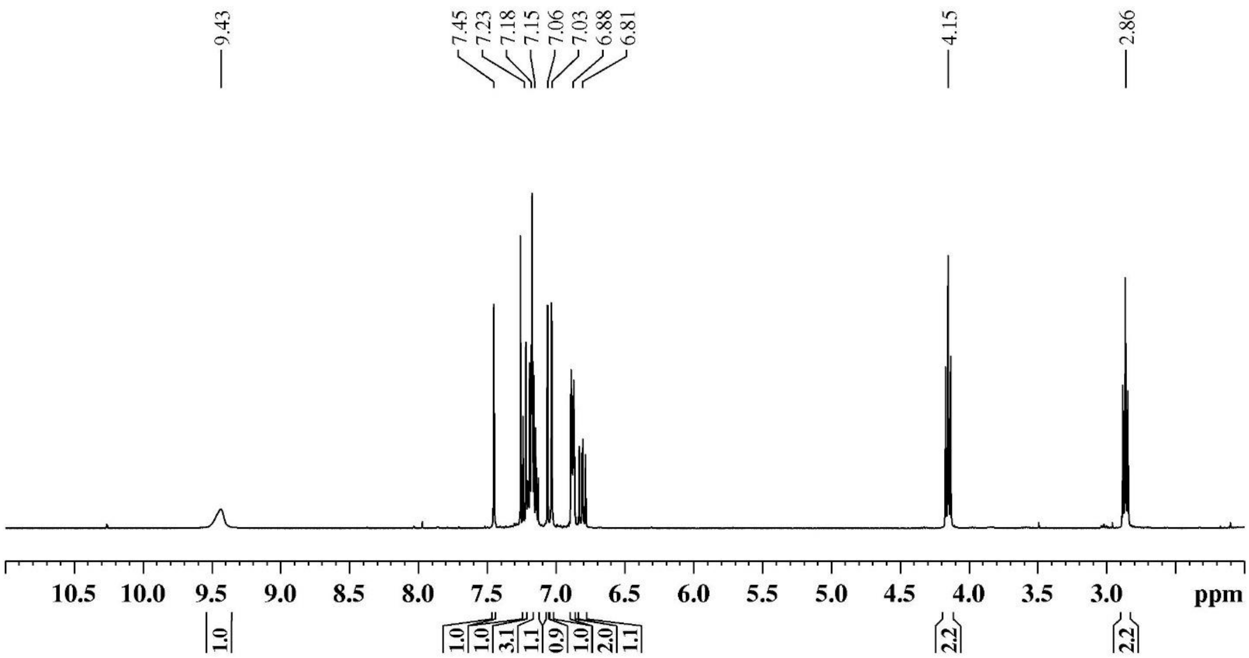

^{13}C NMR ($CDCl_3$, 100 MHz):

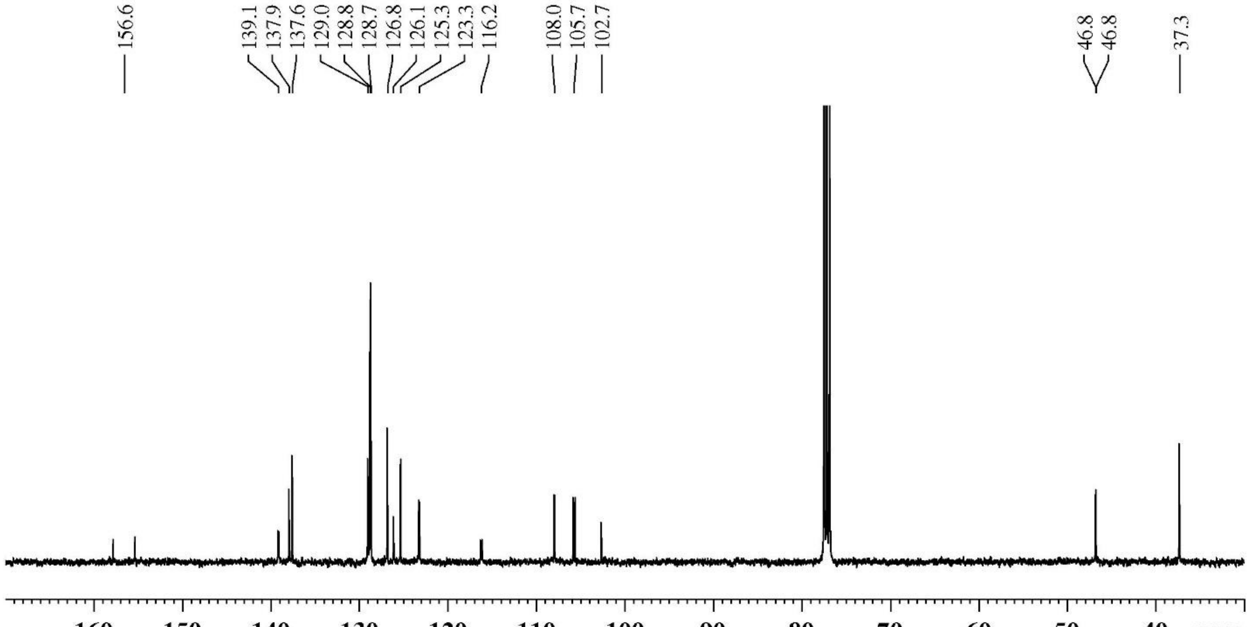

Figure S52. 7-Fluoro-3-(1-phenethyl-1*H*-imidazol-5-yl)-1*H*-indole (**52**)

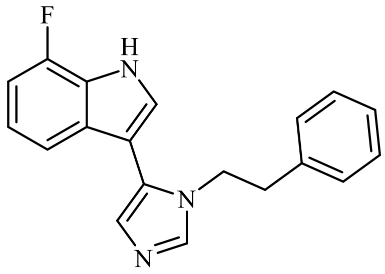

^1H NMR ($CDCl_3$, 400 MHz):

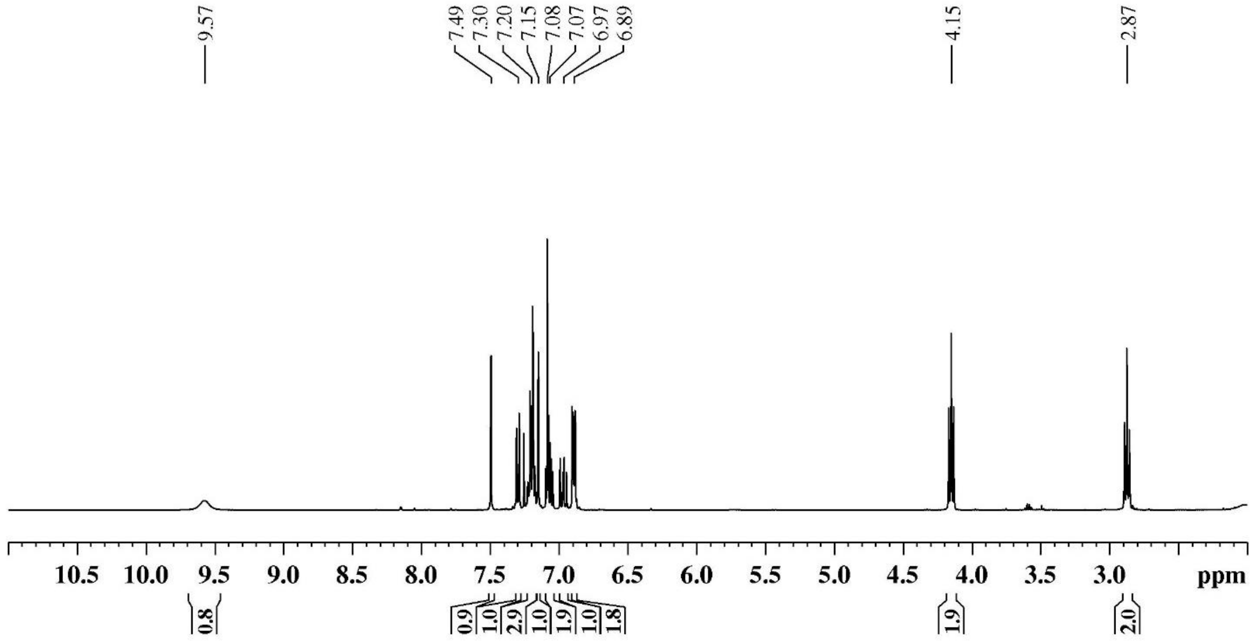

^{13}C NMR ($CDCl_3$, 100 MHz):

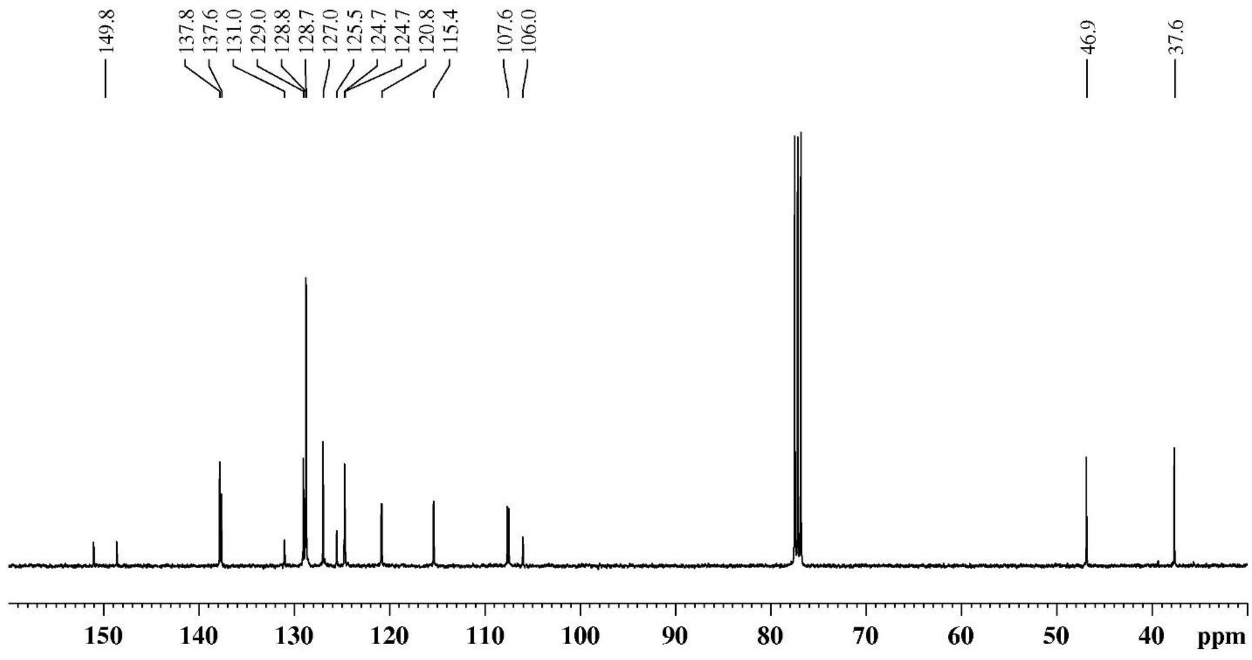

Figure S53. 4-Chloro-3-(1-phenethyl-1*H*-imidazol-5-yl)-1*H*-indole (**53**)

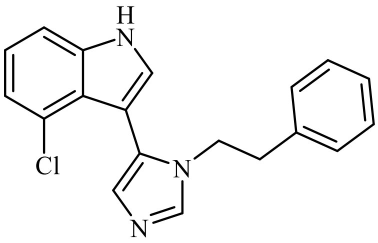

^{1}H NMR ($CDCl_3$, 400 MHz):

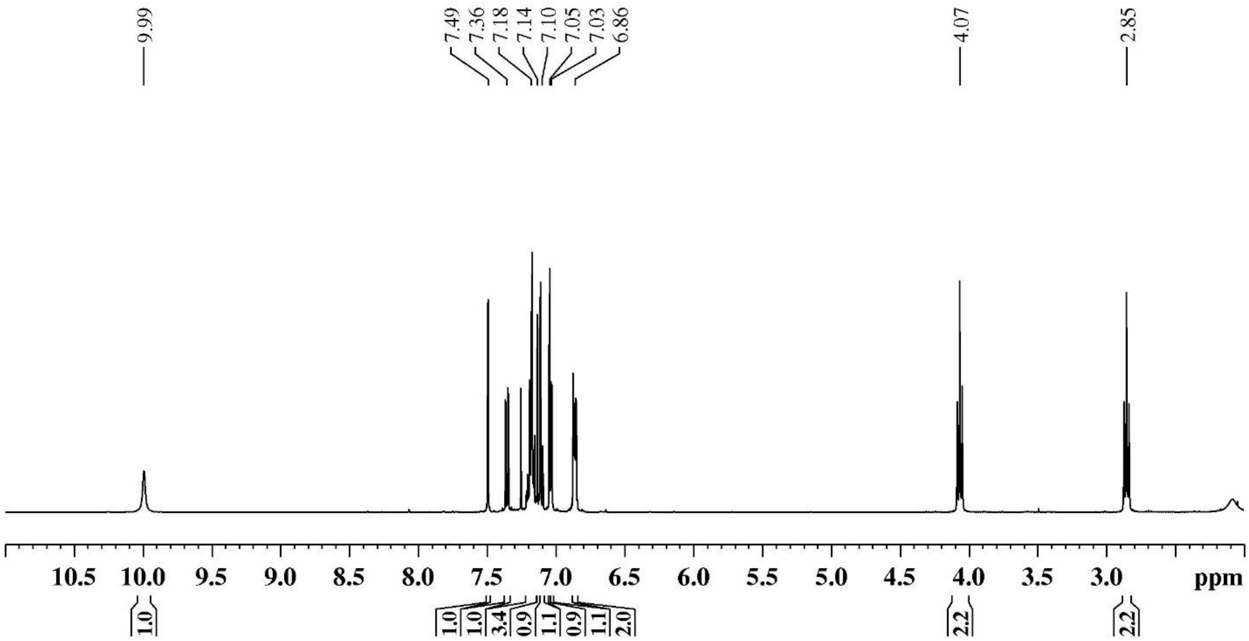

^{13}C NMR ($CDCl_3$, 100 MHz):

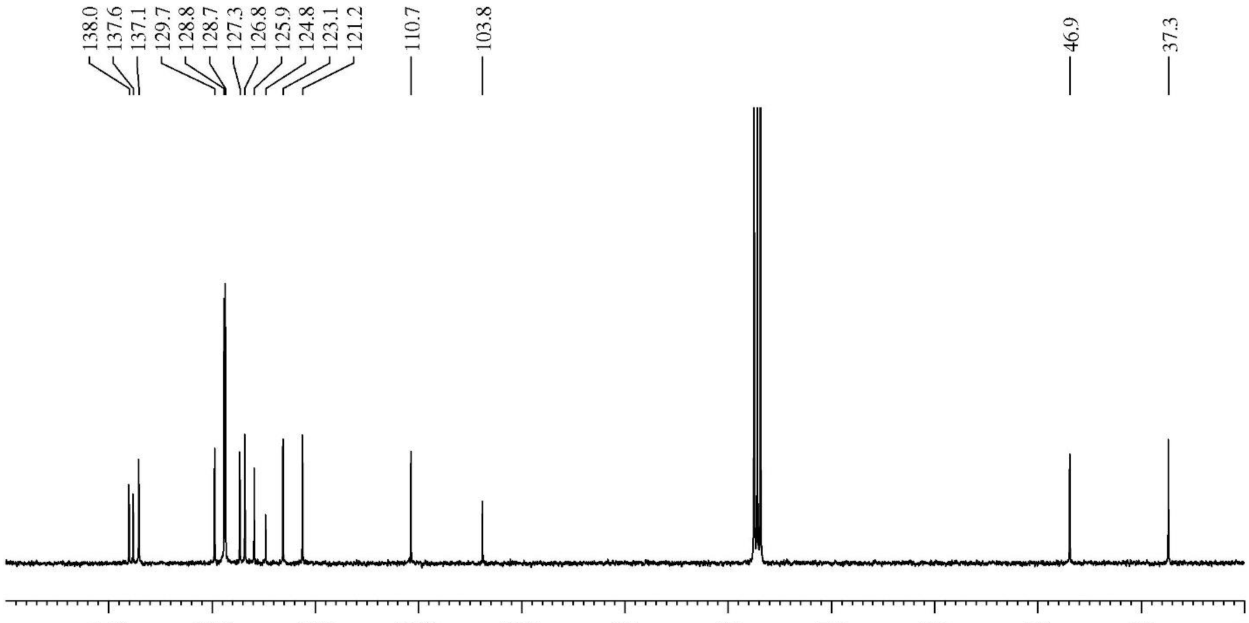

Figure S54. 7-Chloro-3-(1-phenethyl-1*H*-imidazol-5-yl)-1*H*-indole (**54**)

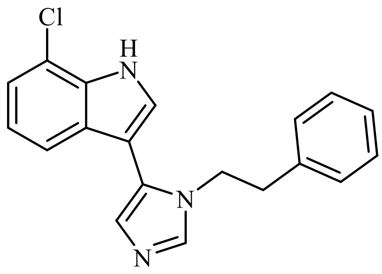

^{1}H NMR ($CDCl_3$, 400 MHz):

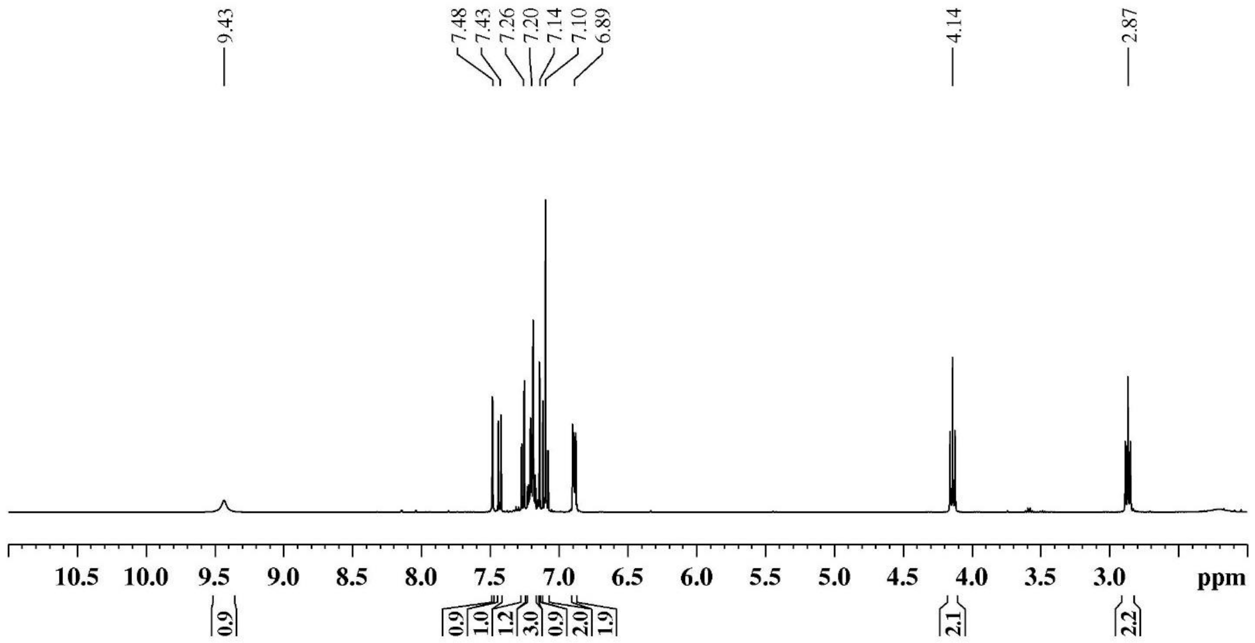

^{13}C NMR ($CDCl_3$, 100 MHz):

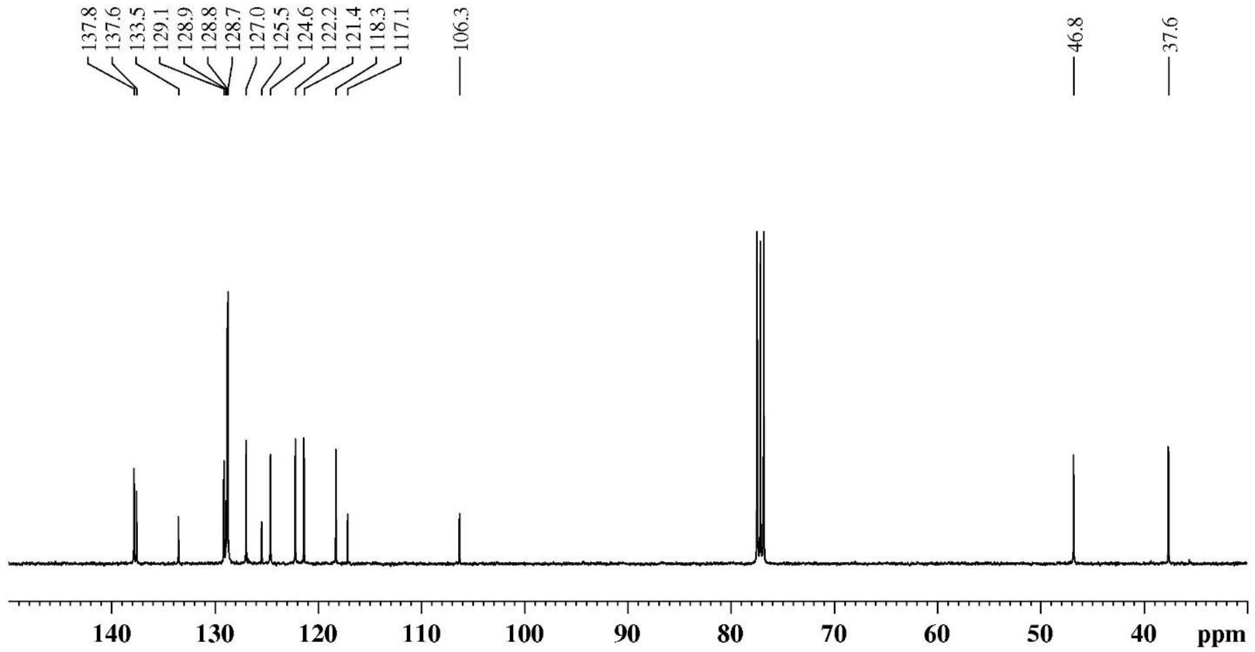

Figure S55. 4-Bromo-3-(1-phenethyl-1*H*-imidazol-5-yl)-1*H*-indole (**55**)

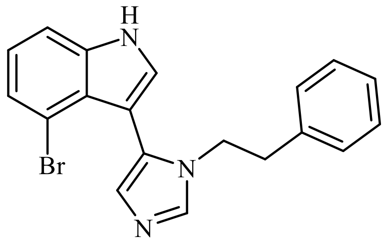

^{1}H NMR ($CDCl_3$, 400 MHz):

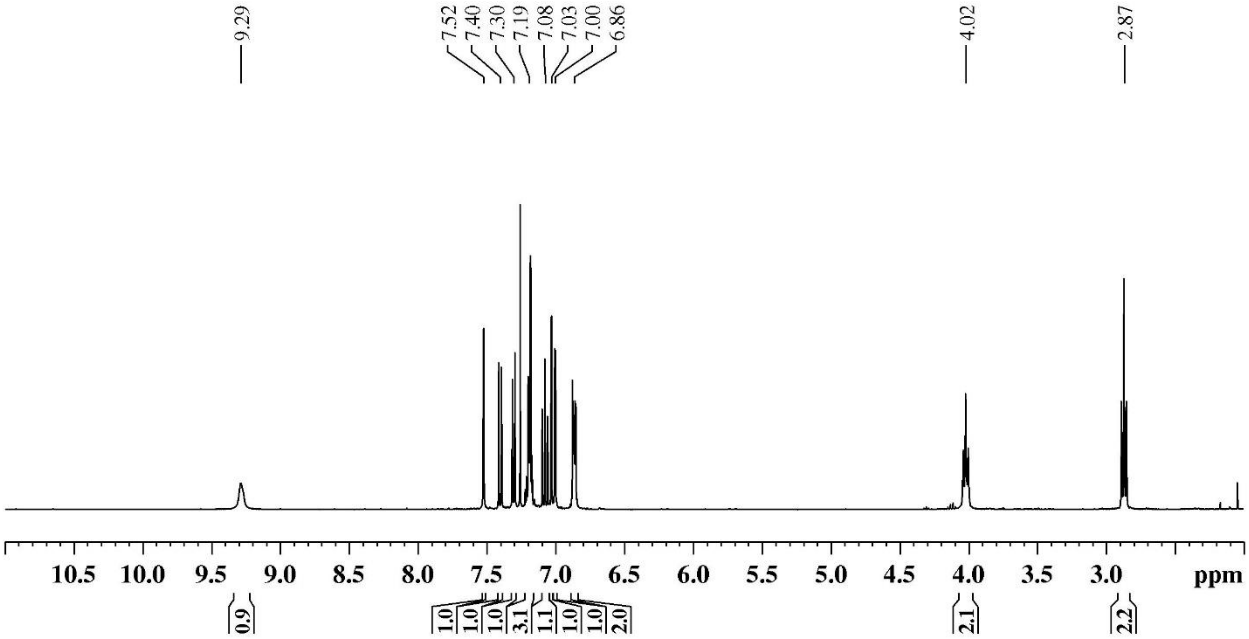

^{13}C NMR ($CDCl_3$, 100 MHz):

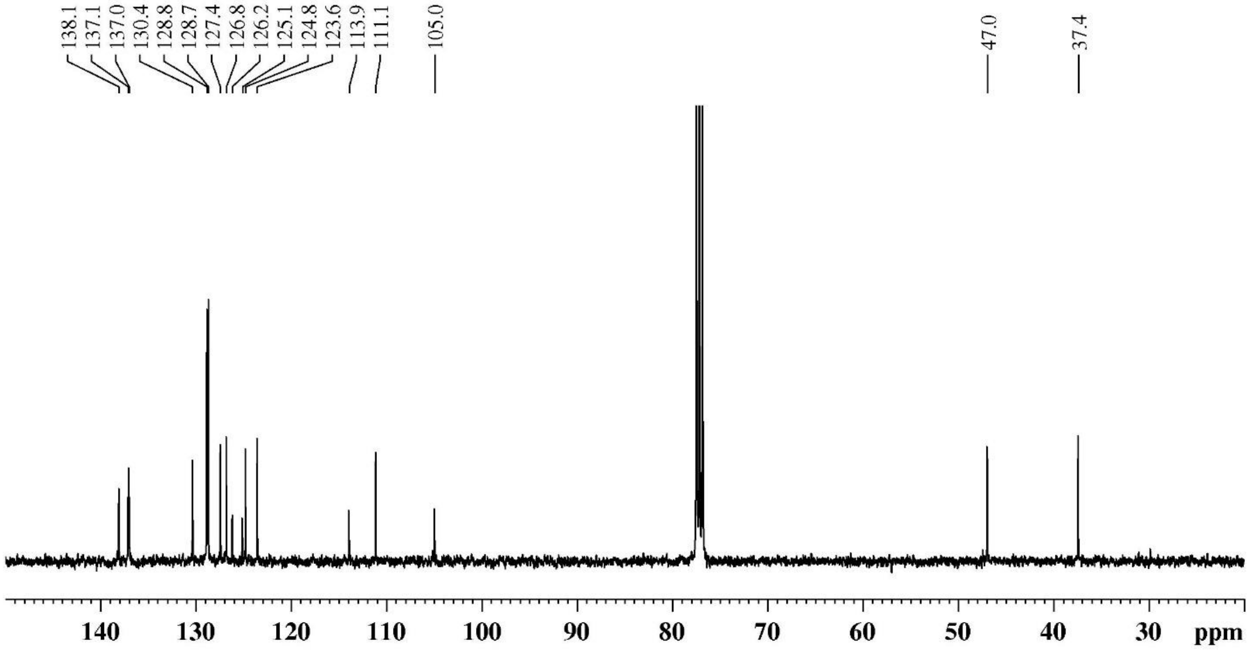

Figure S56. 5-Methoxy-3-(1-phenethyl-1*H*-imidazol-5-yl)-1*H*-indole (**56**)

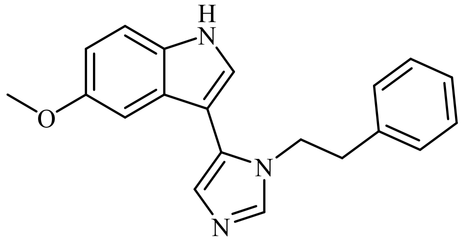

^{1}H NMR ($CDCl_3$, 400 MHz):

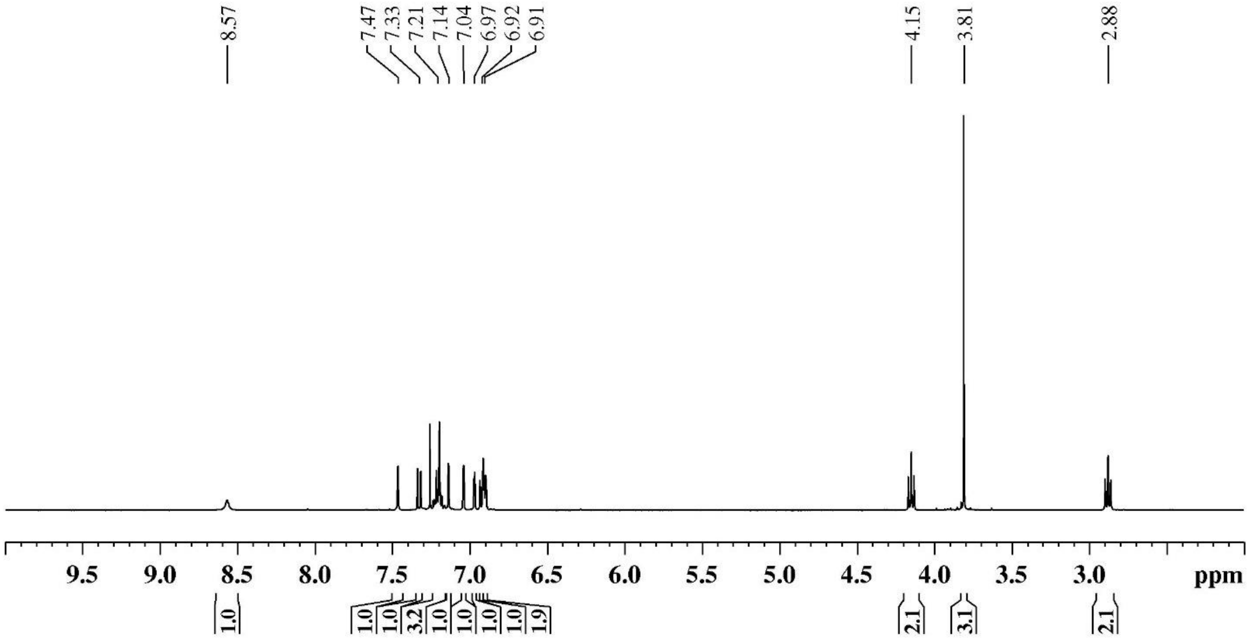

^{13}C NMR ($CDCl_3$, 100 MHz):

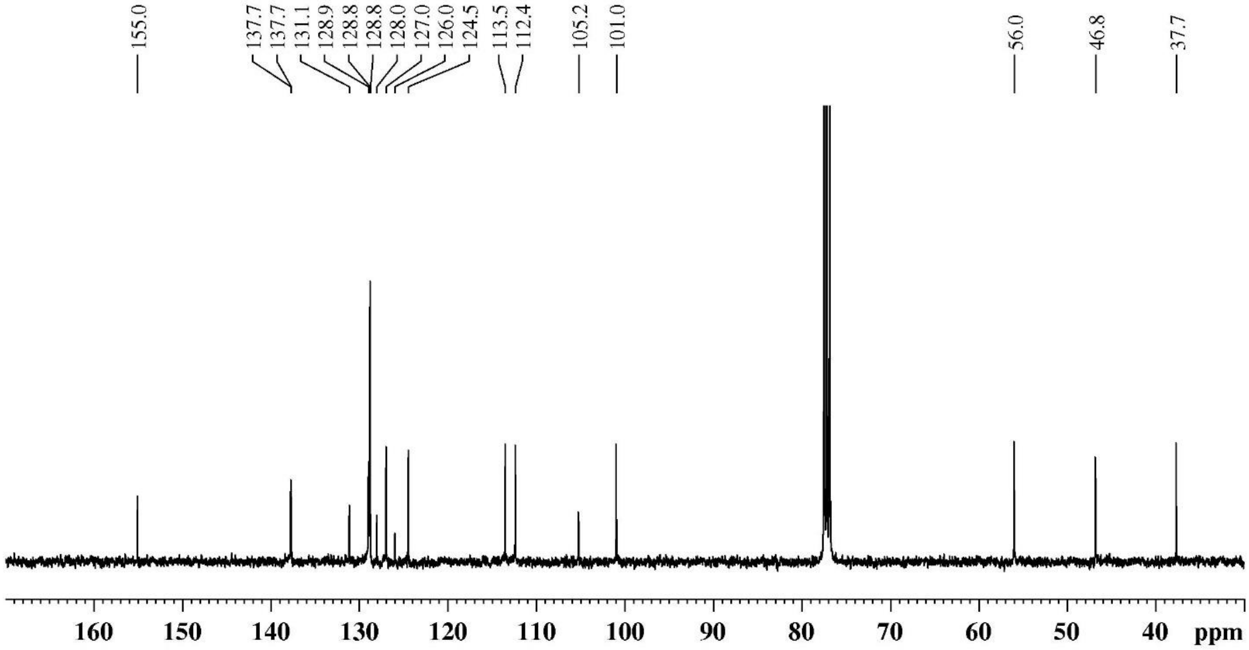

Figure S57. 6-Methoxy-3-(1-phenethyl-1*H*-imidazol-5-yl)-1*H*-indole (**57**)

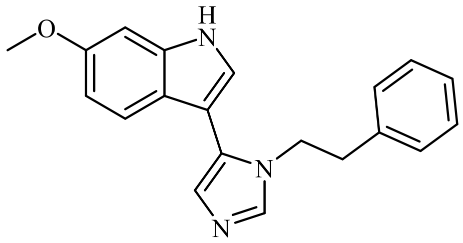

^{1}H NMR ($CDCl_3$, 400 MHz):

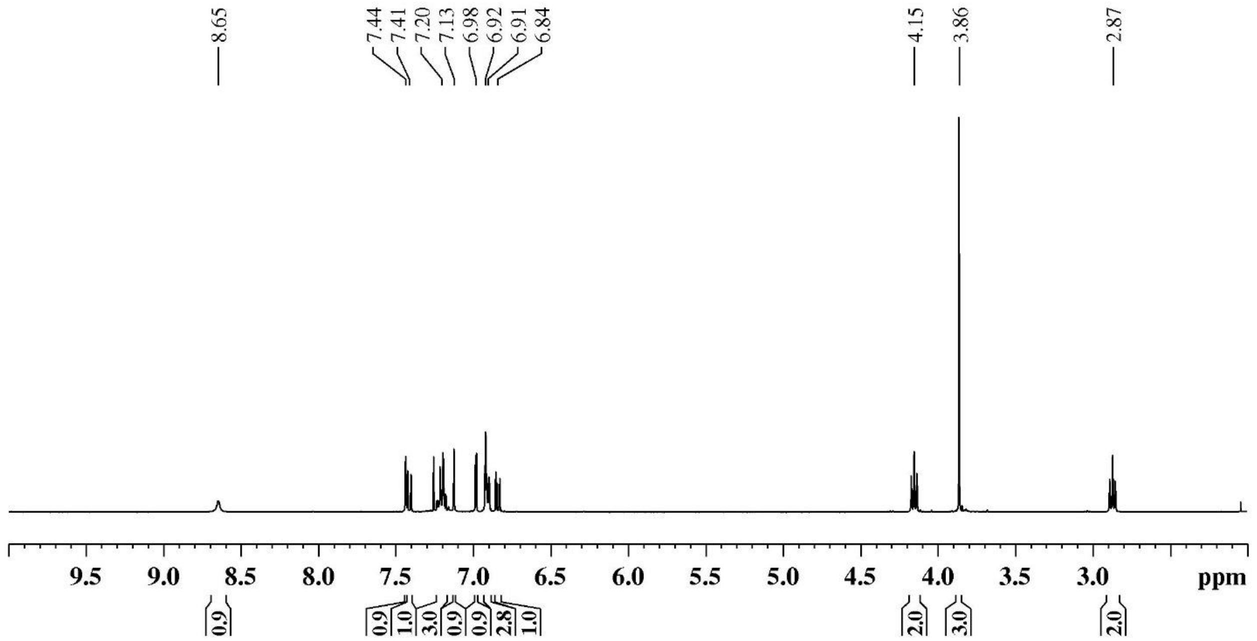

^{13}C NMR ($CDCl_3$, 100 MHz):

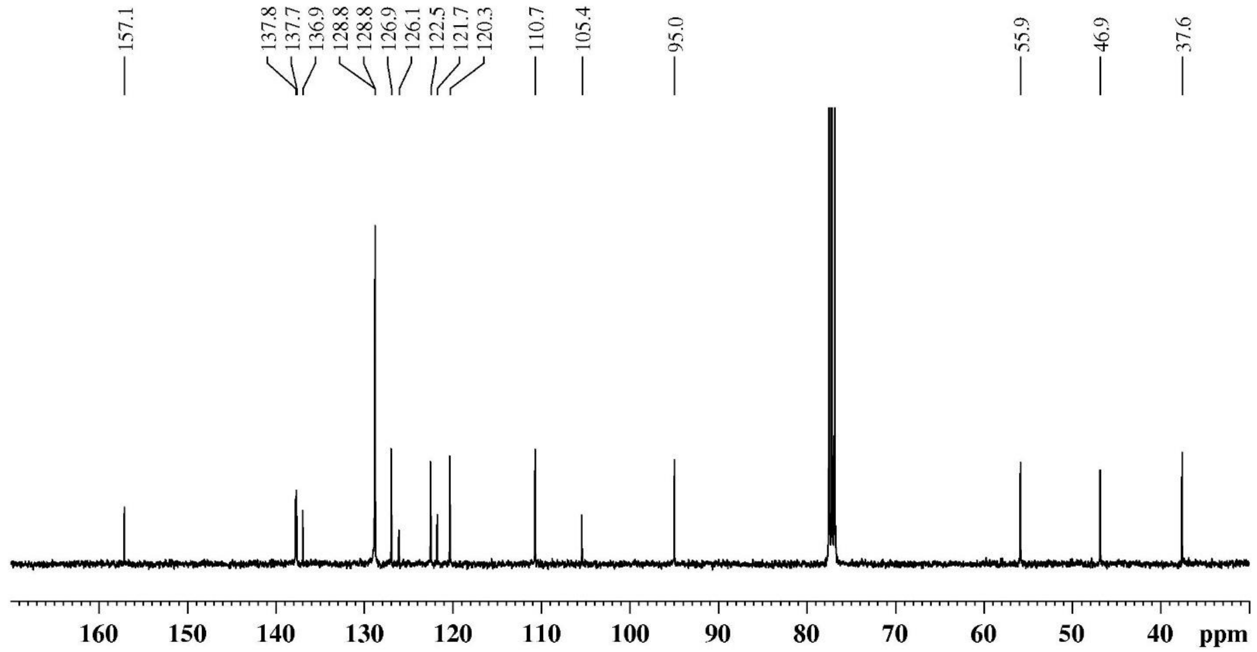

Figure S58. 5-Chloro-3-(1-(3-phenylpropyl)-1*H*-imidazol-5-yl)-1*H*-indole (**58**)

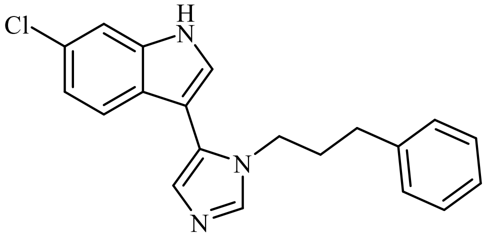

^{1}H NMR (DMSO-d_6, 400 MHz):

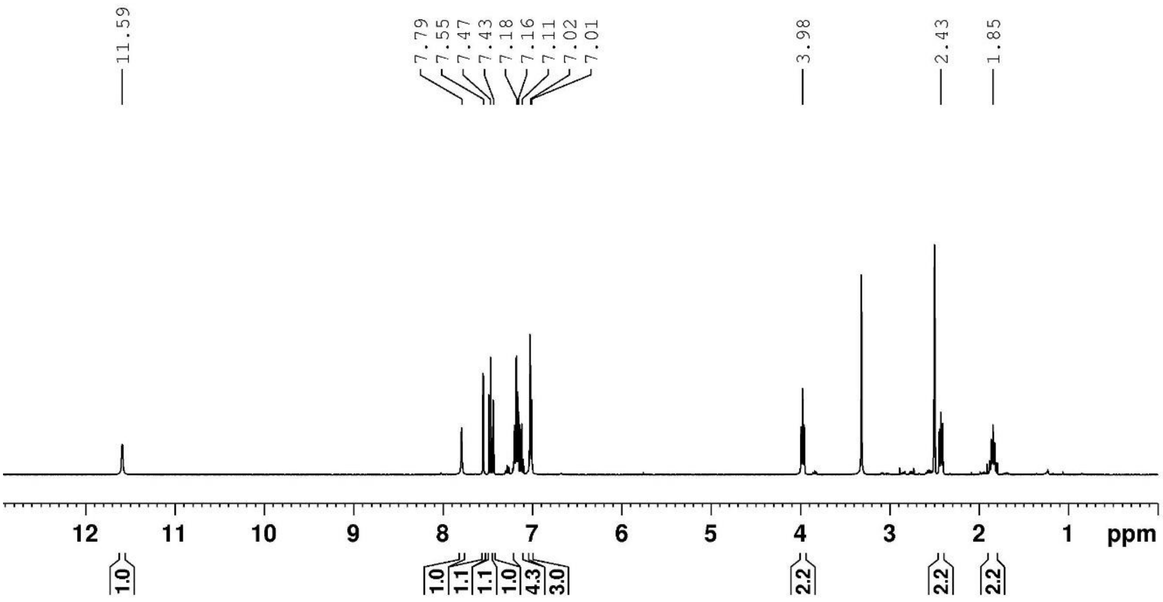

^{13}C NMR (DMSO-d_6, 100 MHz):

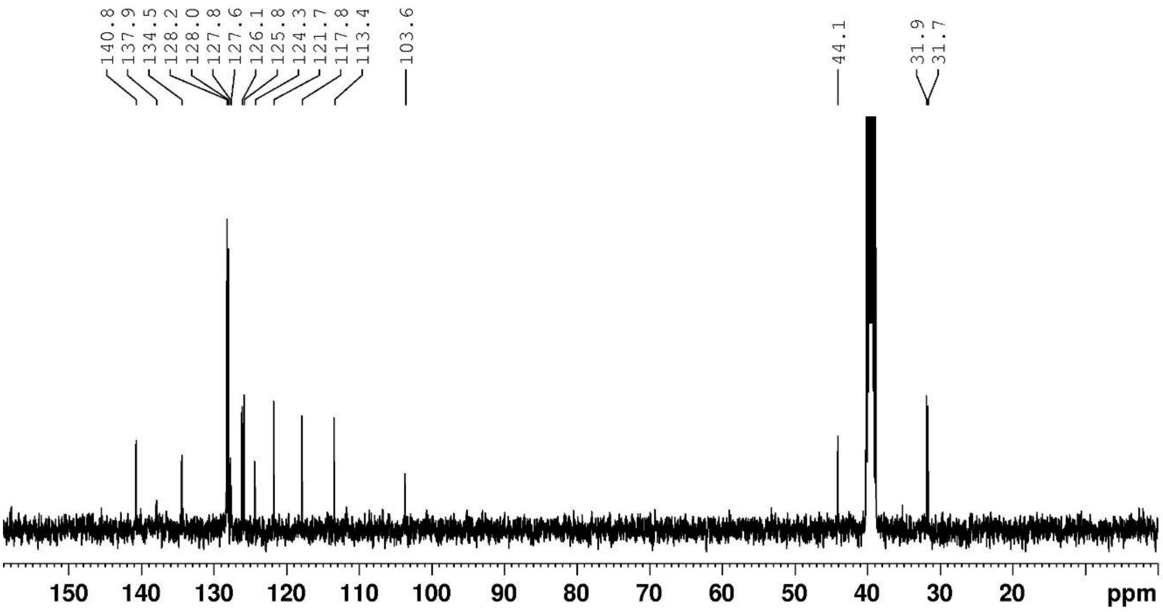

Figure S59. 4-Fluoro-3-(1-(4-methoxybenzyl)-1*H*-imidazol-5-yl)-1*H*-indole (**59**)

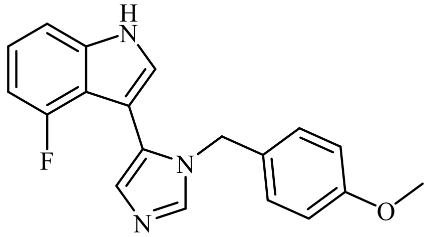

^{1}H NMR ($CDCl_3$, 400 MHz):

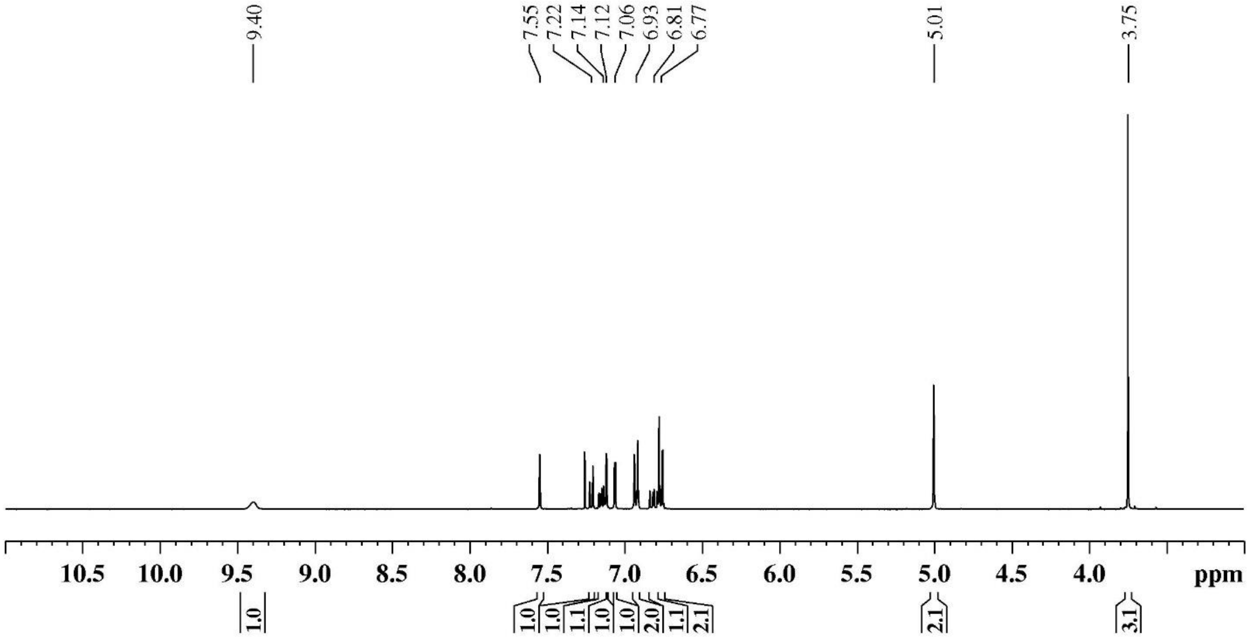

^{13}C NMR ($CDCl_3$, 100 MHz):

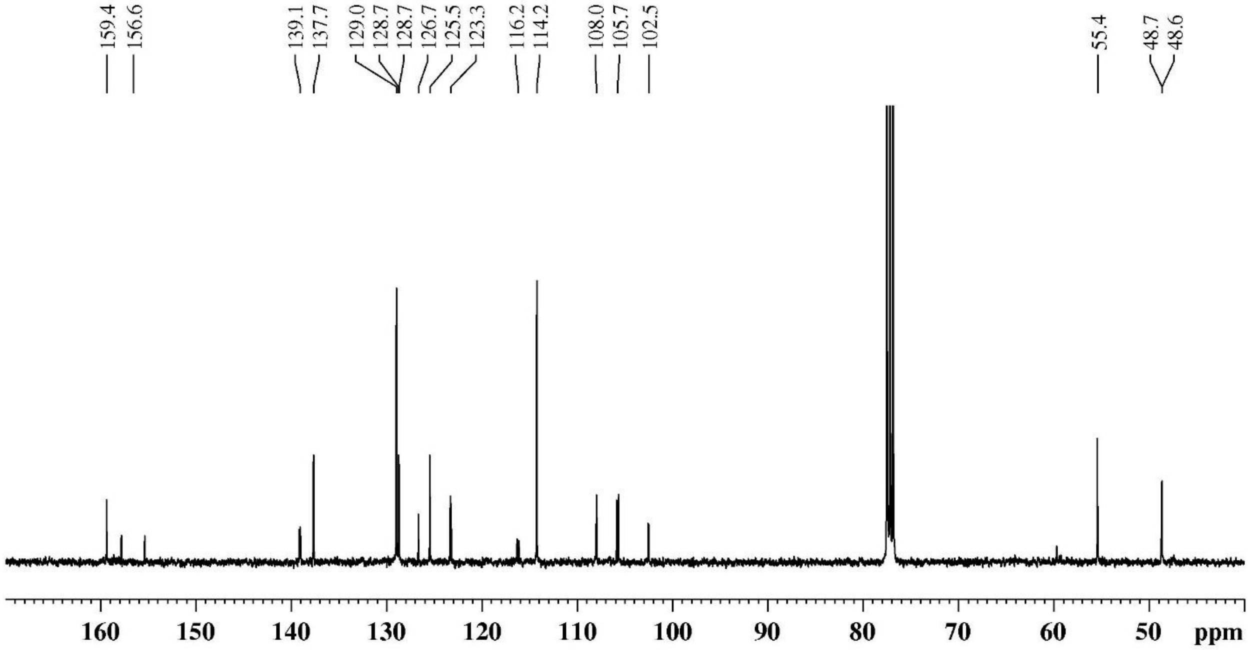

Figure S60. 7-Fluoro-3-(1-(4-methoxybenzyl)-1*H*-imidazol-5-yl)-1*H*-indole (**60**)

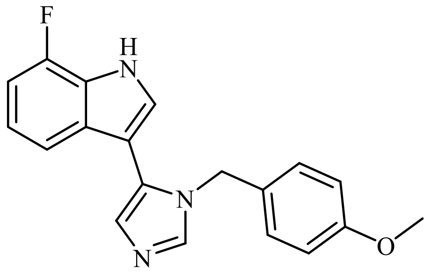

^{1}H NMR ($CDCl_3$, 400 MHz):

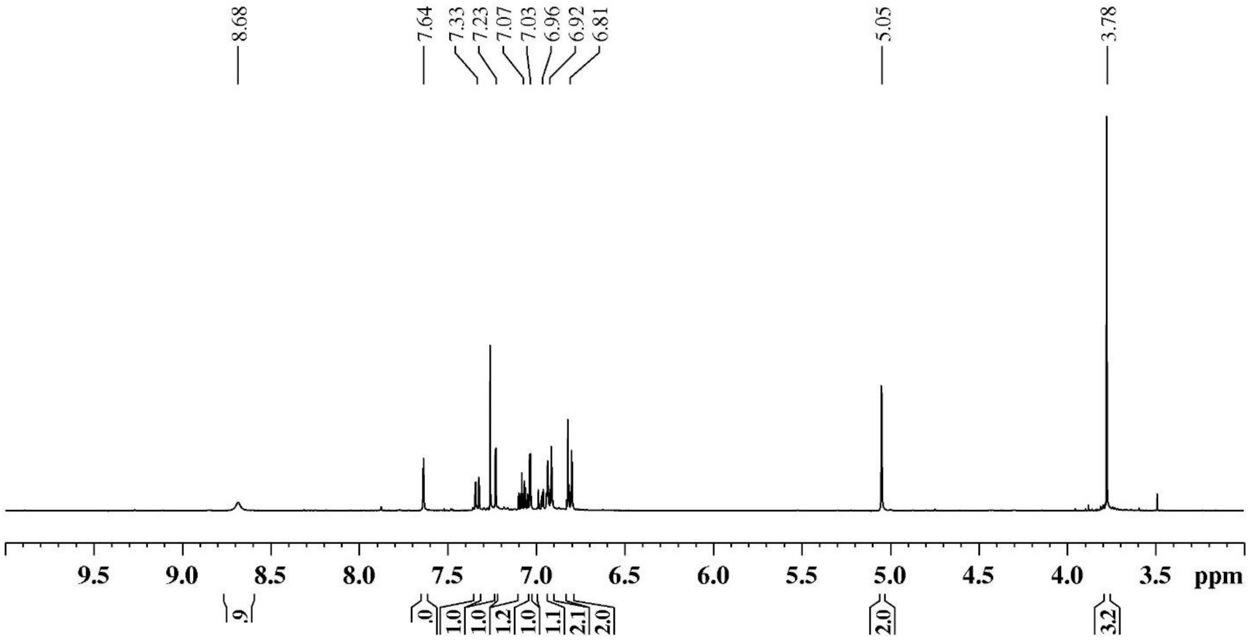

^{13}C NMR ($CDCl_3$, 100 MHz):

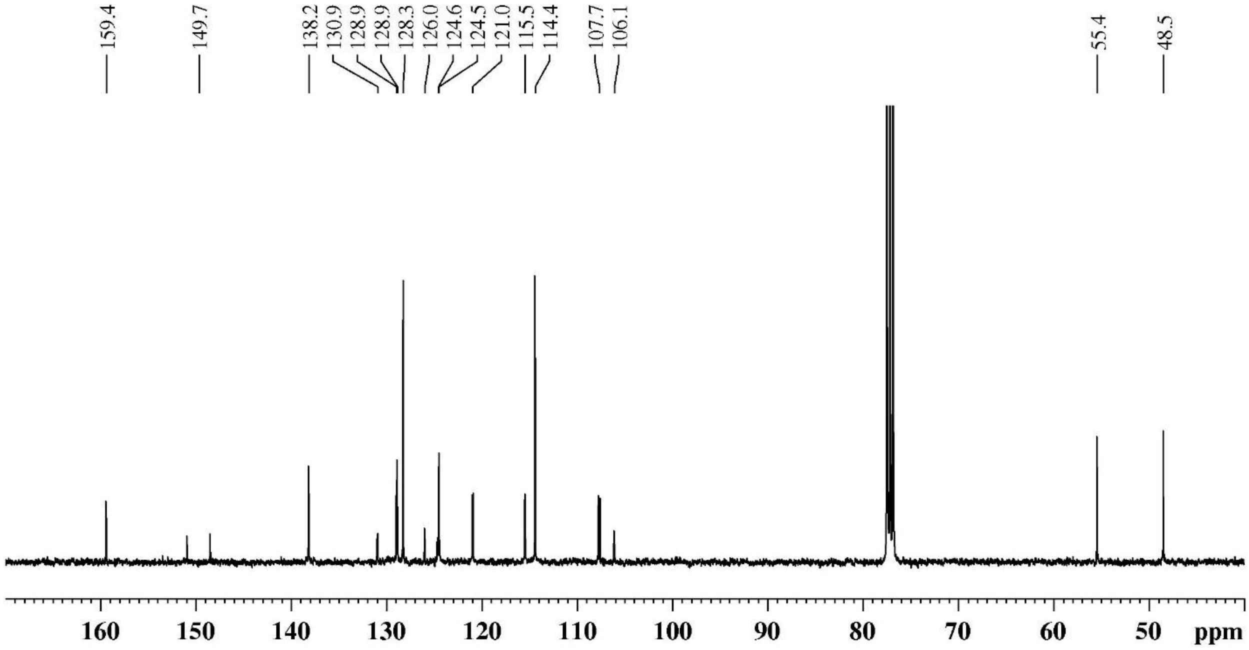

Figure S61. 4-Chloro-3-(1-(4-methoxybenzyl)-1*H*-imidazol-5-yl)-1*H*-indole (**61**)

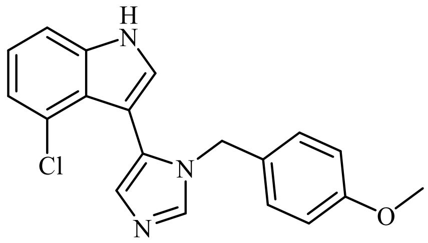

^{1}H NMR ($CDCl_3$, 400 MHz):

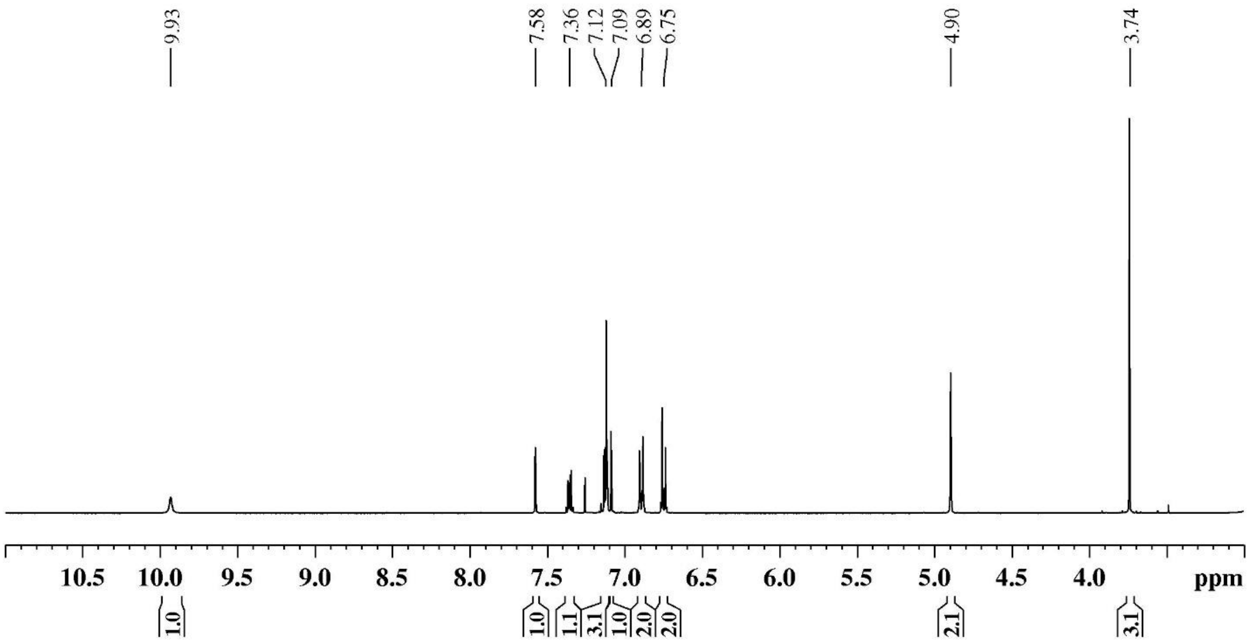

^{13}C NMR ($CDCl_3$, 100 MHz):

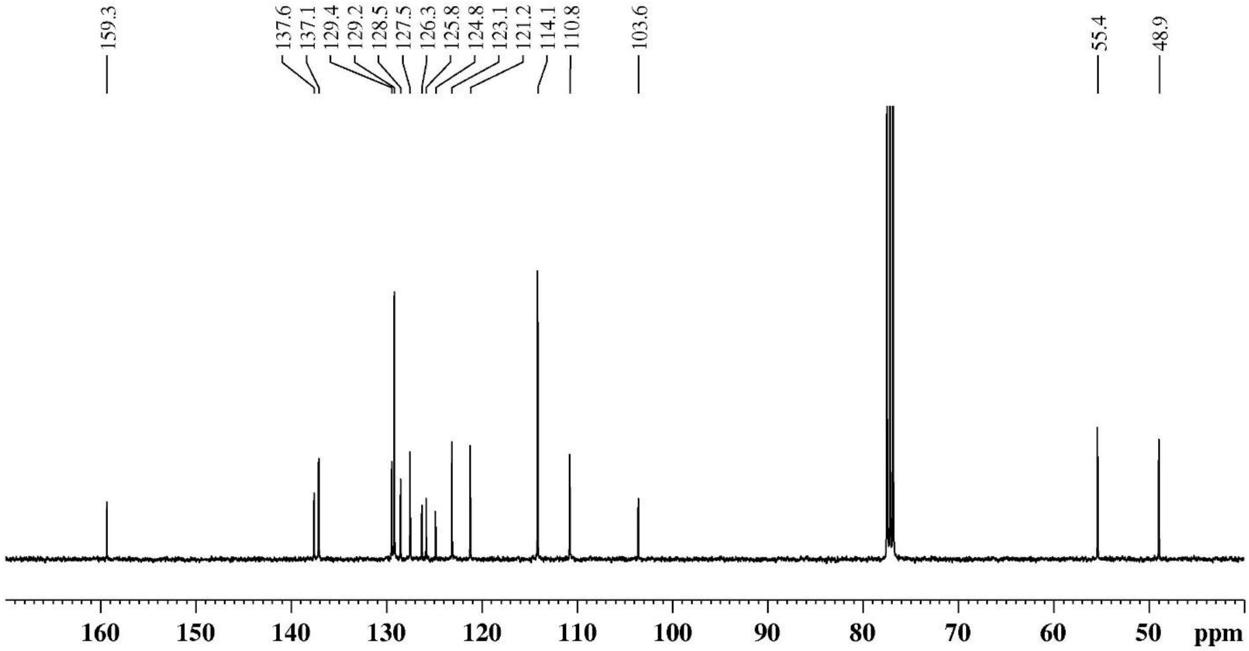

Figure S62. 7-Chloro-3-(1-(4-methoxybenzyl)-1*H*-imidazol-5-yl)-1*H*-indole (**62**)

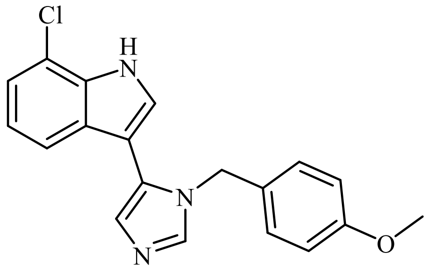

^{1}H NMR ($CDCl_3$, 400 MHz):

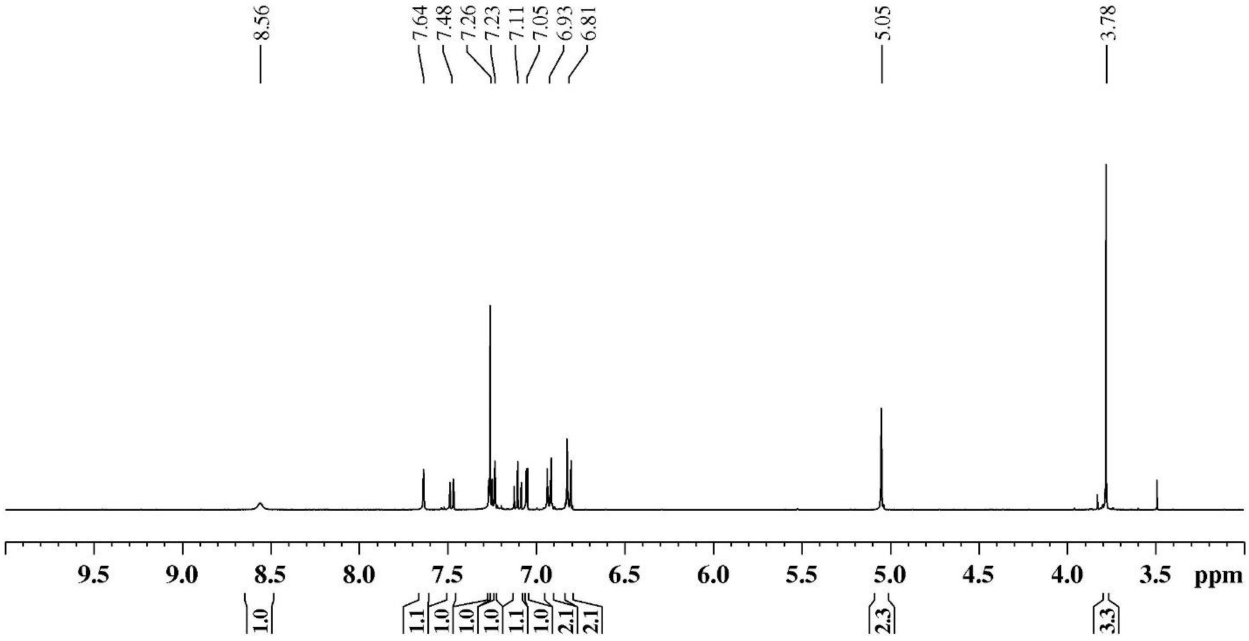

^{13}C NMR ($CDCl_3$, 100 MHz):

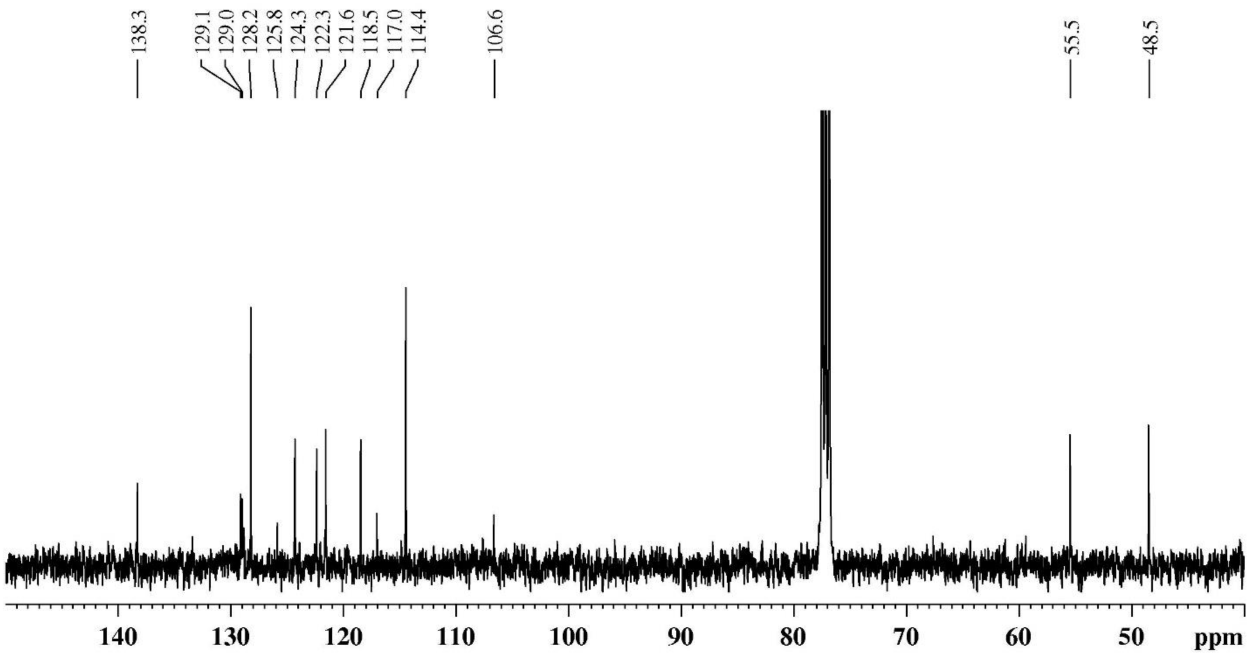

Figure S63. 4-Bromo-3-(1-(4-methoxybenzyl)-1*H*-imidazol-5-yl)-1*H*-indole (**63**)

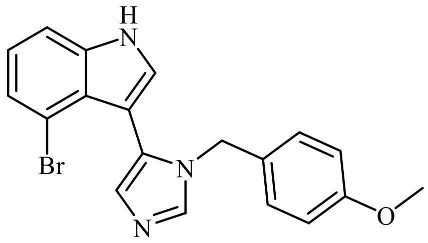

^{1}H NMR ($CDCl_3$, 400 MHz):

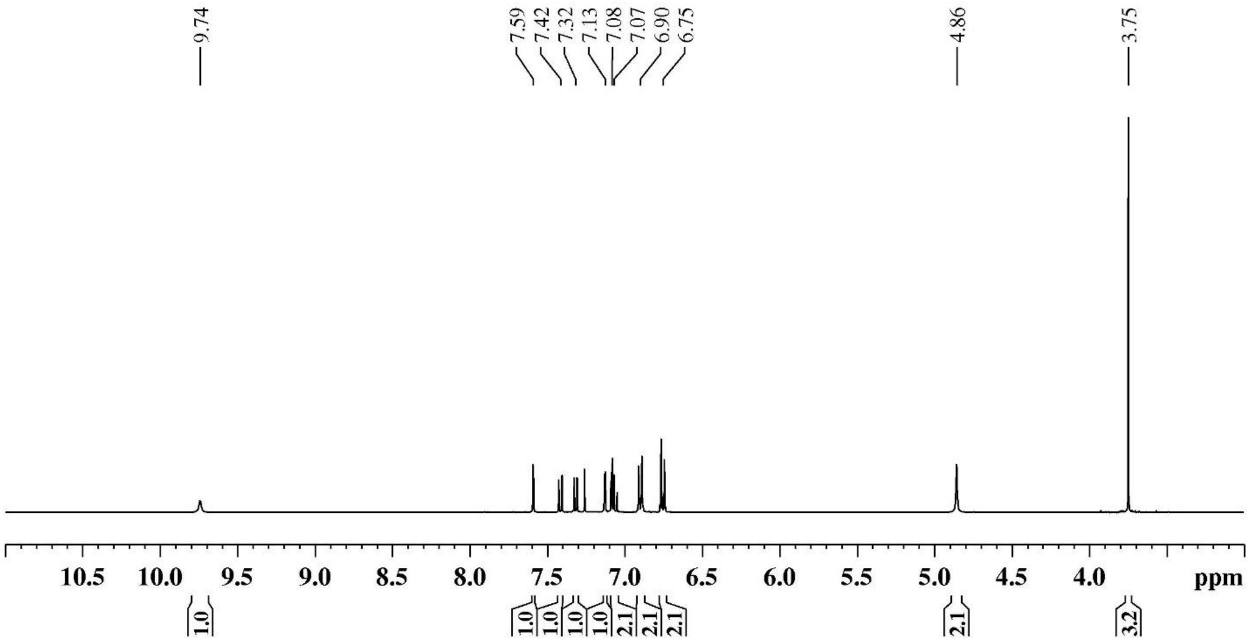

^{13}C NMR ($CDCl_3$, 100 MHz):

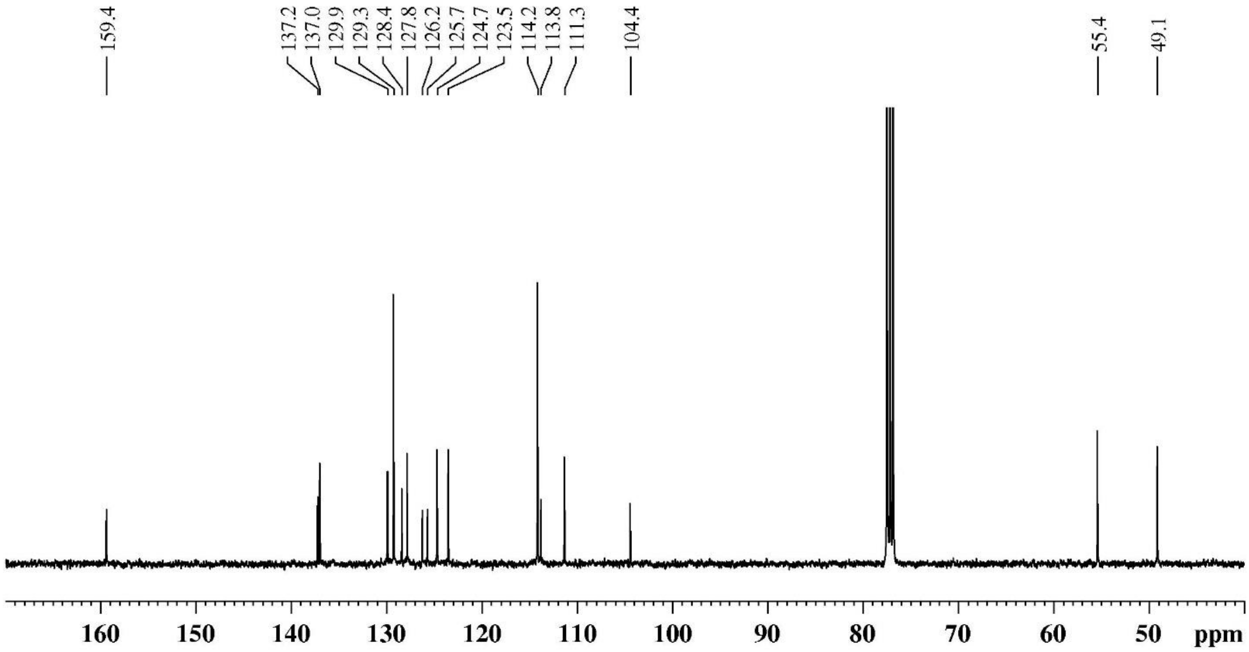

Figure S64. 5-Methoxy-3-(1-(4-methoxybenzyl)-1*H*-imidazol-5-yl)-1*H*-indole (**64**)

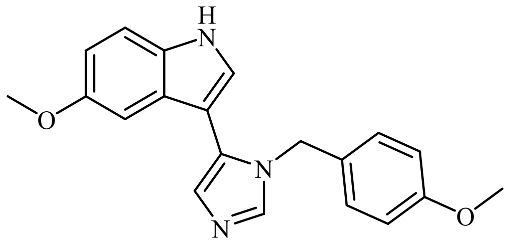

^{1}H NMR ($CDCl_3$, 400 MHz):

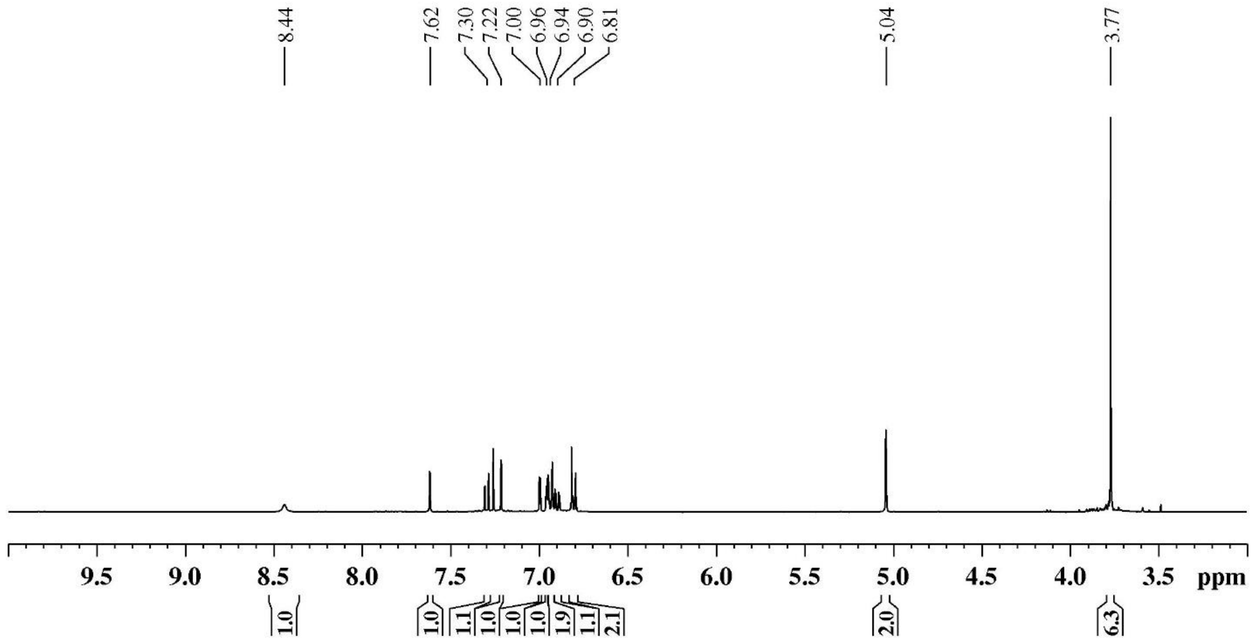

^{13}C NMR ($CDCl_3$, 100 MHz):

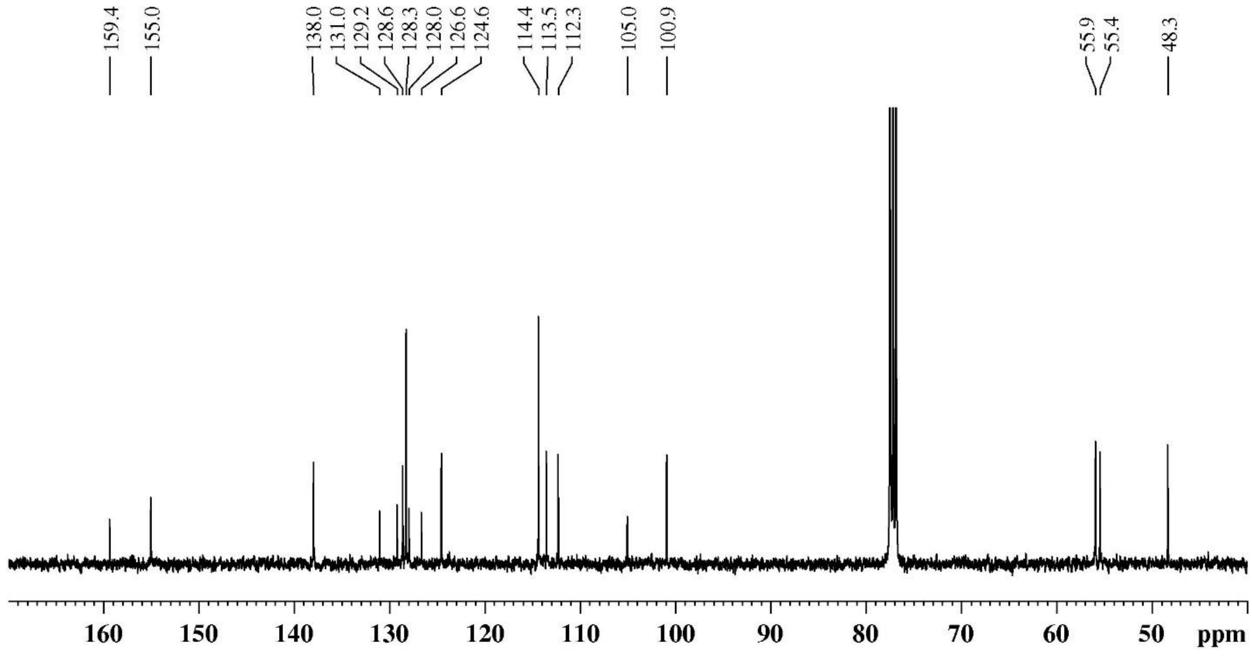

Figure S65. 6-Methoxy-3-(1-(4-methoxybenzyl)-1*H*-imidazol-5-yl)-1*H*-indole (**65**)

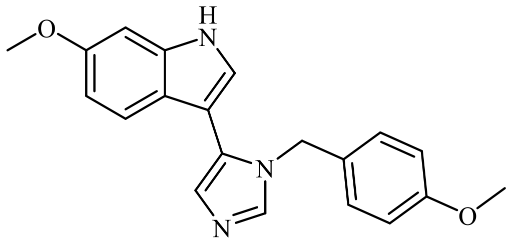

^{1}H NMR ($CDCl_3$, 400 MHz):

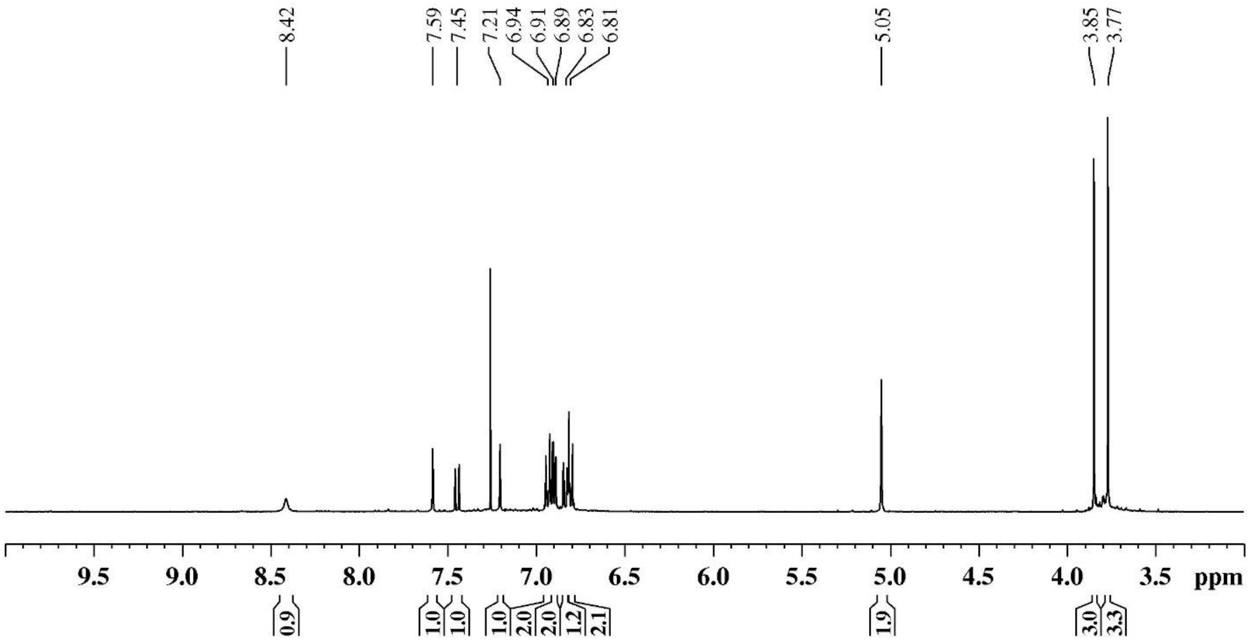

^{13}C NMR ($CDCl_3$, 100 MHz):

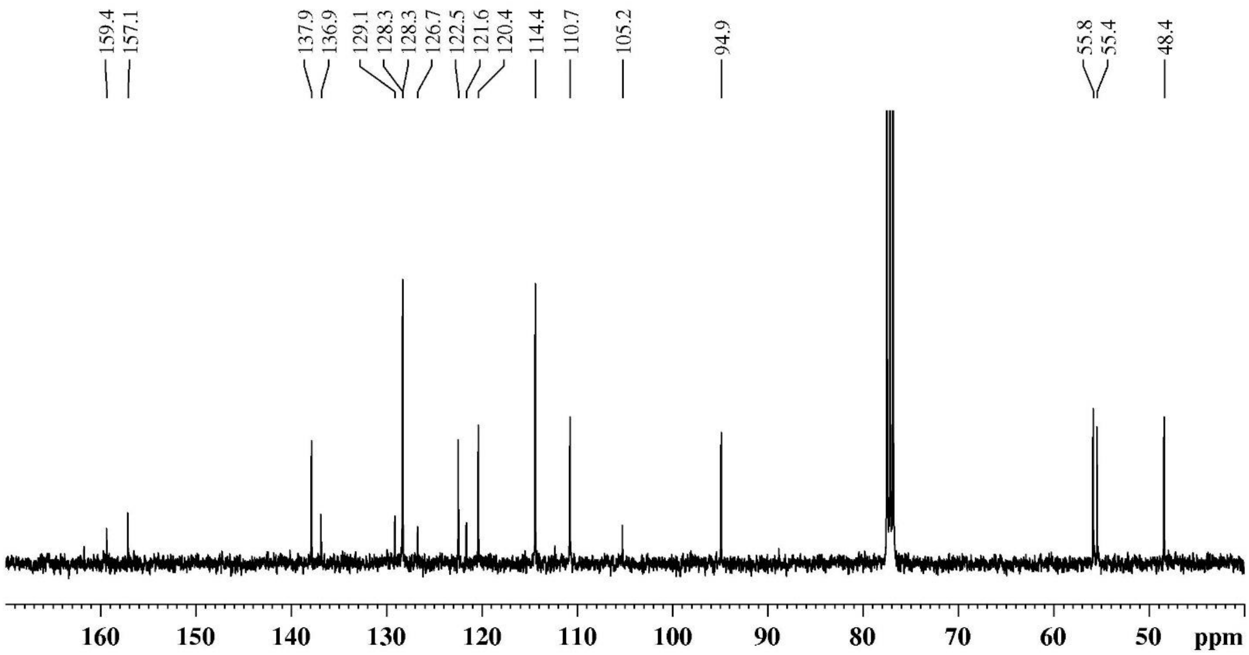

Figure S66. 4-Fluoro-3-(1-(4-methoxyphenethyl)-1*H*-imidazol-5-yl)-1*H*-indole (**66**)

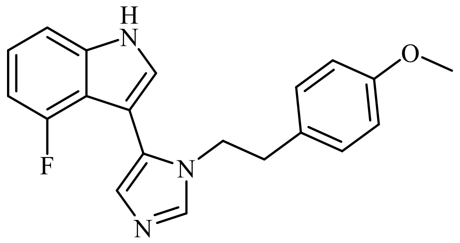

^{1}H NMR ($CDCl_3$, 400 MHz):

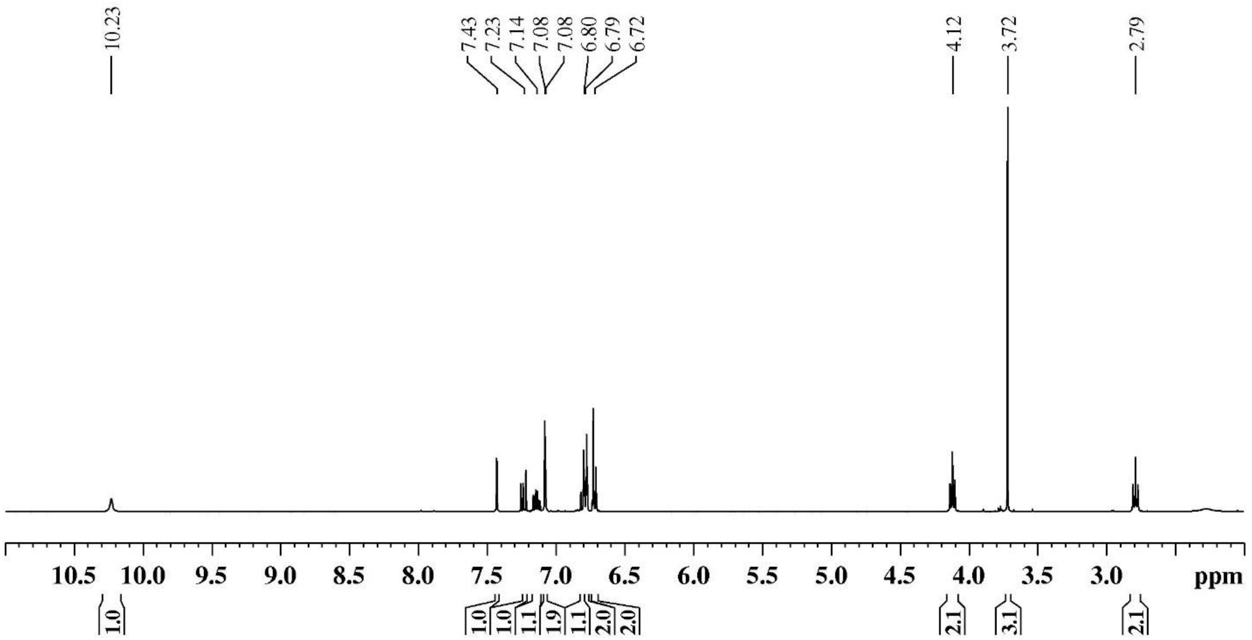

^{13}C NMR ($CDCl_3$, 100 MHz):

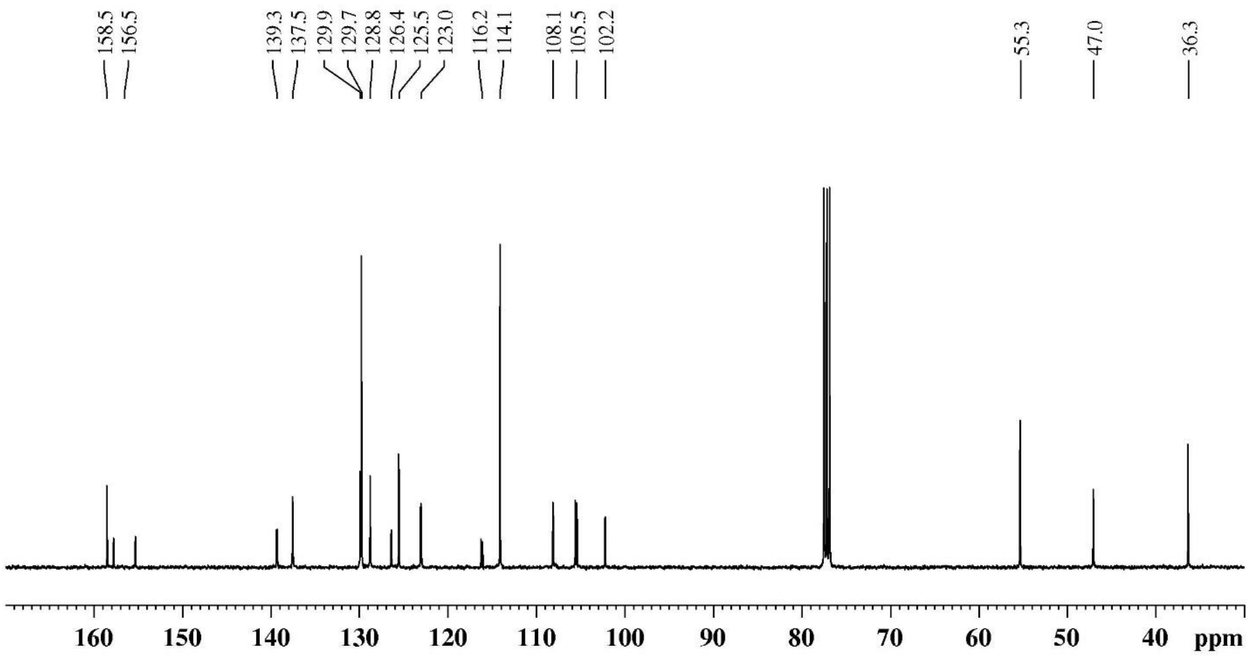

Figure S67. 7-Fluoro-3-(1-(4-methoxyphenethyl)-1*H*-imidazol-5-yl)-1*H*-indole (**67**)

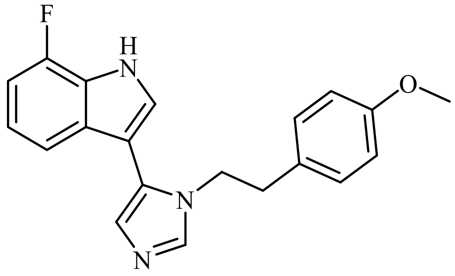

^{1}H NMR ($CDCl_3$, 400 MHz):

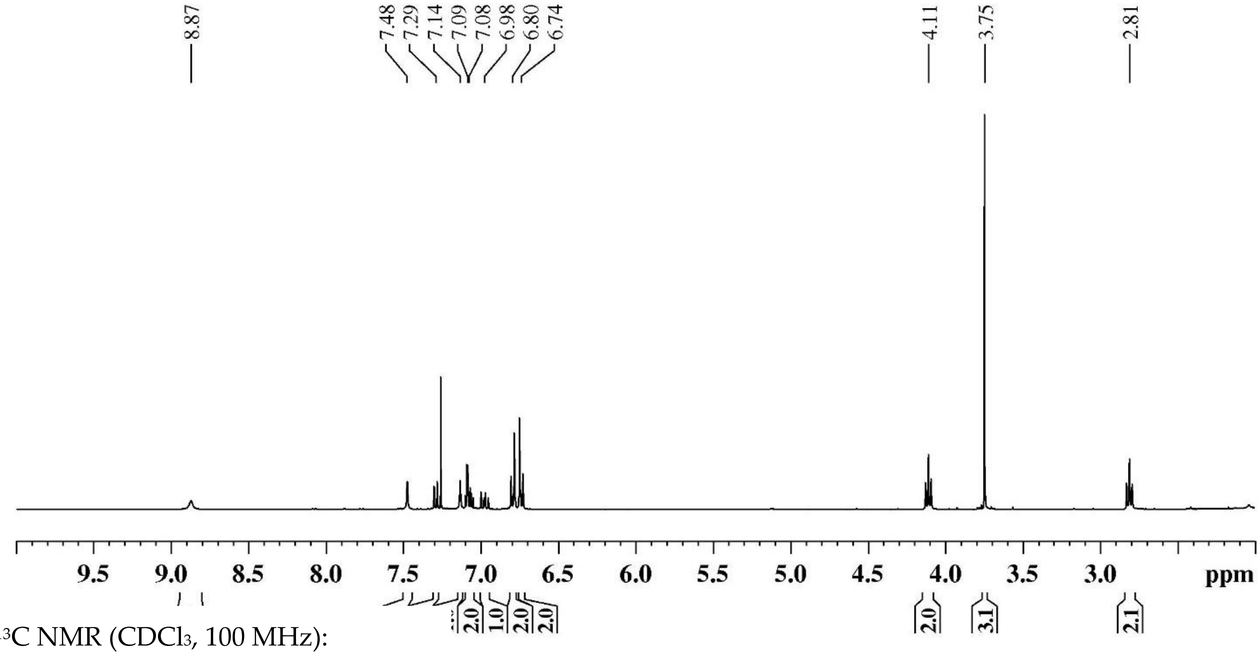

^{13}C NMR ($CDCl_3$, 100 MHz):

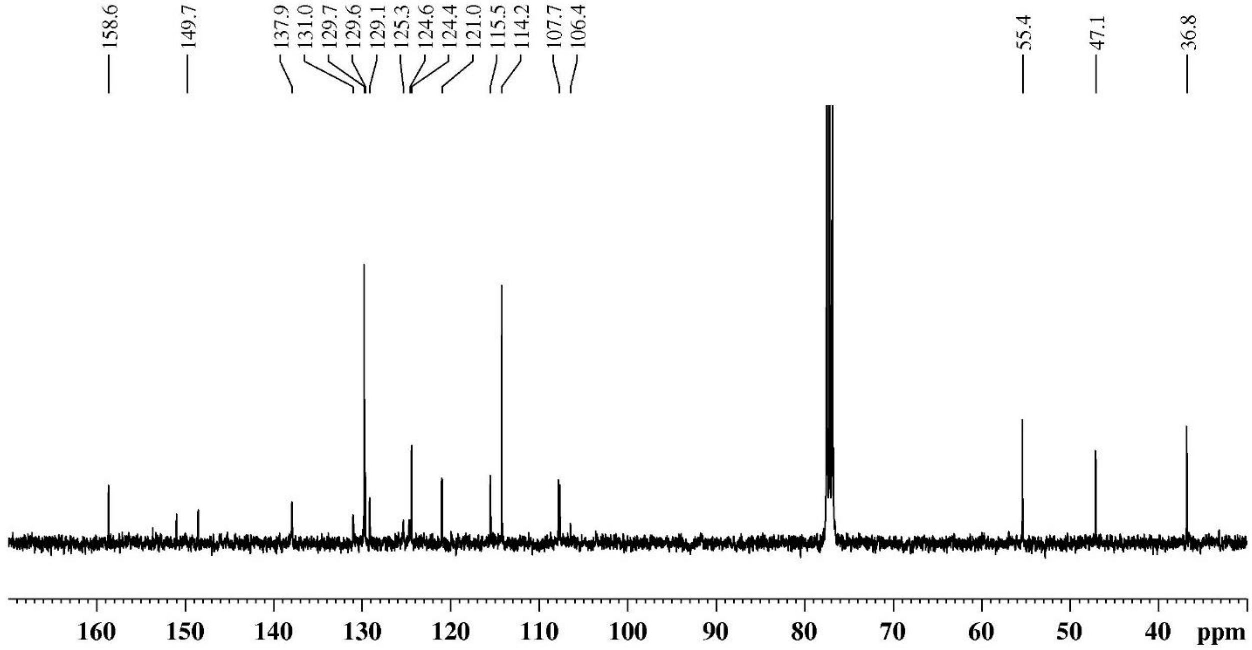

Figure S68. 4-Chloro-3-(1-(4-methoxyphenethyl)-1*H*-imidazol-5-yl)-1*H*-indole (**68**)

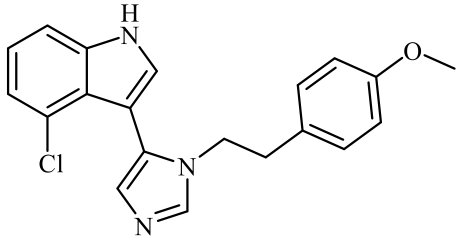

^{1}H NMR ($CDCl_3$, 400 MHz):

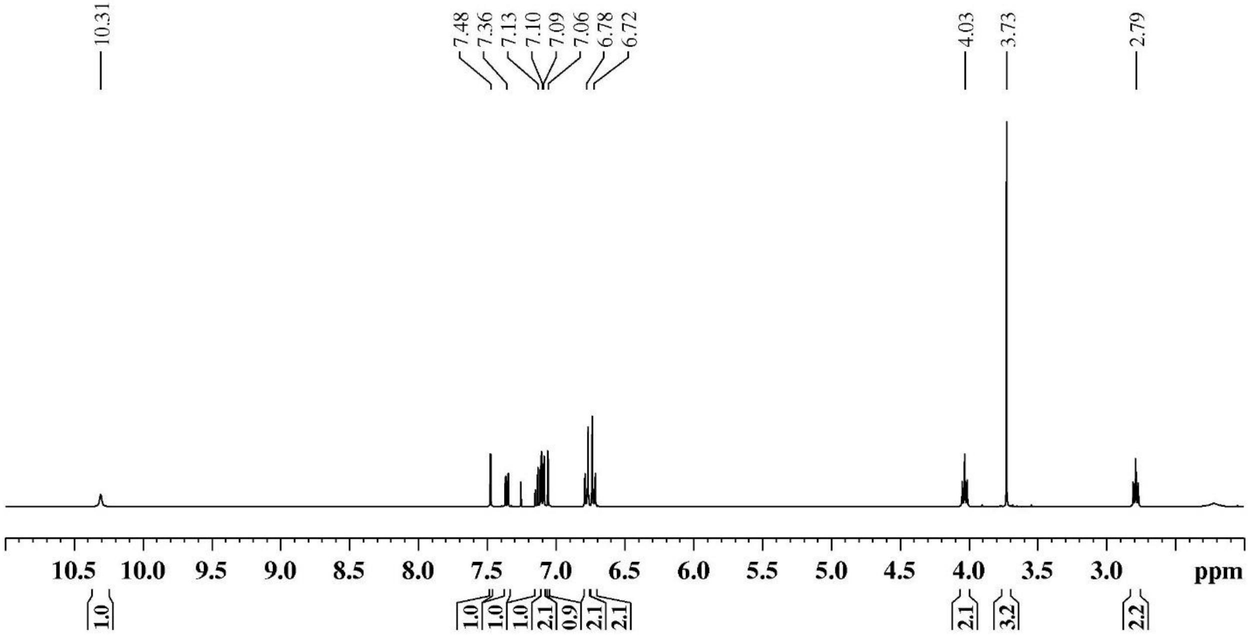

^{13}C NMR ($CDCl_3$, 100 MHz):

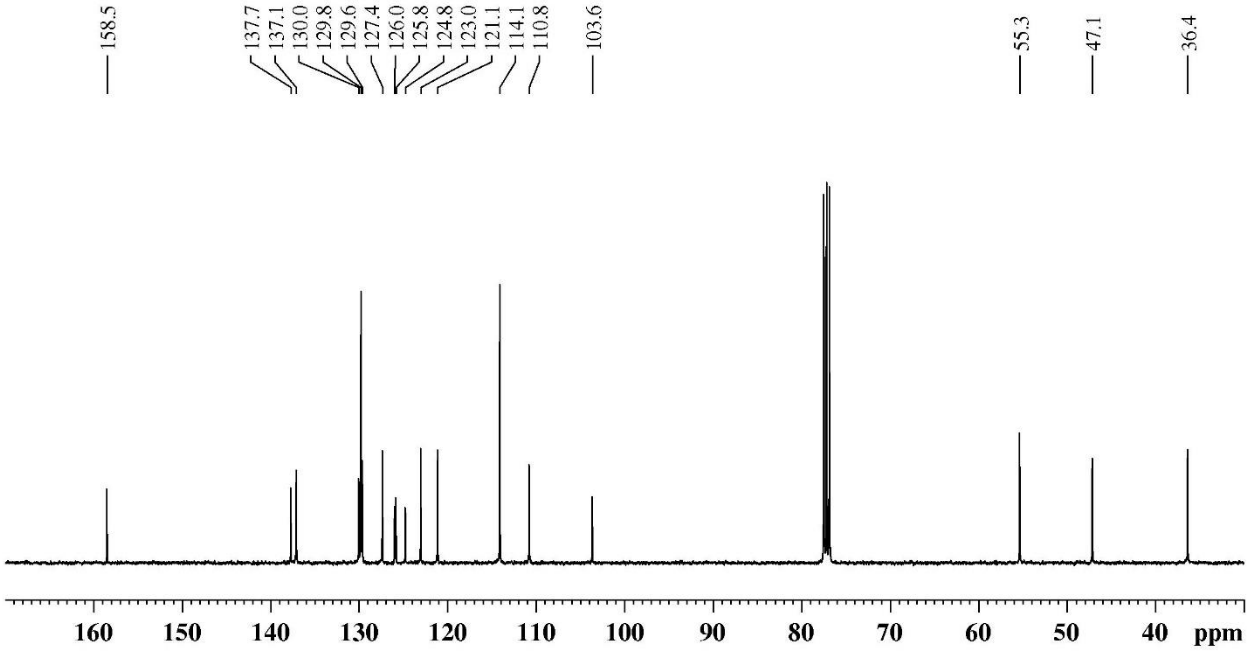

Figure S69. 7-Chloro-3-(1-(4-methoxyphenethyl)-1*H*-imidazol-5-yl)-1*H*-indole (**69**)

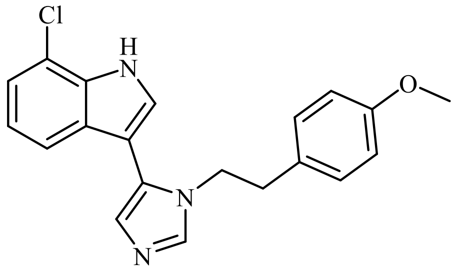

^{1}H NMR (CDCl$_3$, 400 MHz):

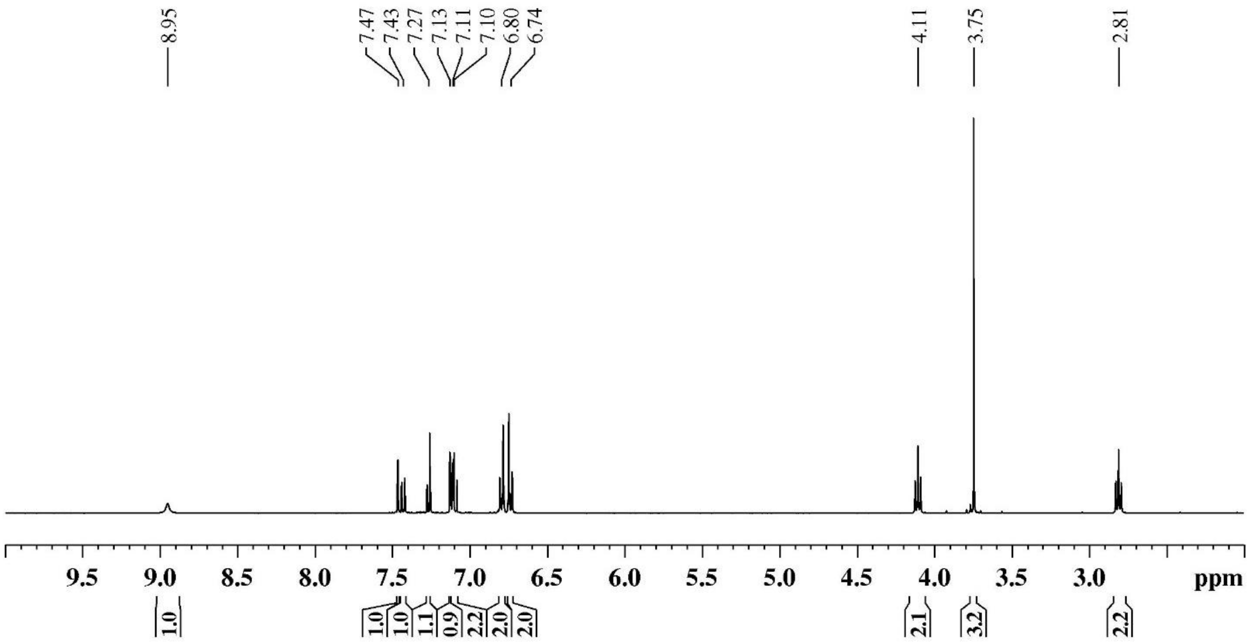

^{13}C NMR (CDCl$_3$, 100 MHz):

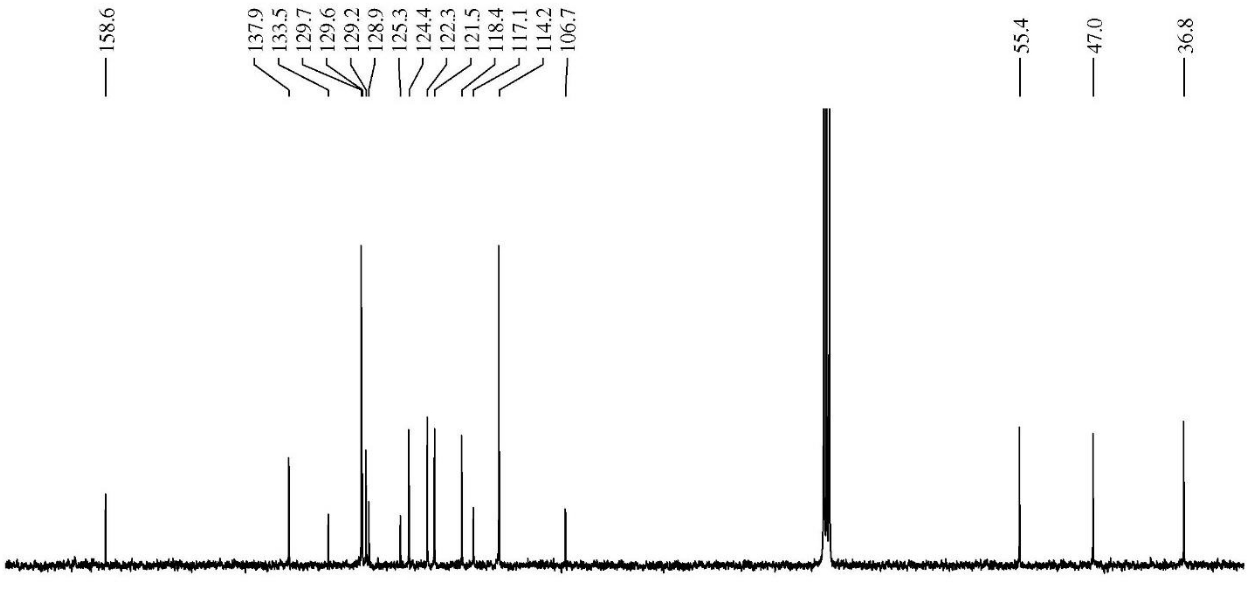

Figure S70. 4-Bromo-3-(1-(4-methoxyphenethyl)-1*H*-imidazol-5-yl)-1*H*-indole (**70**)

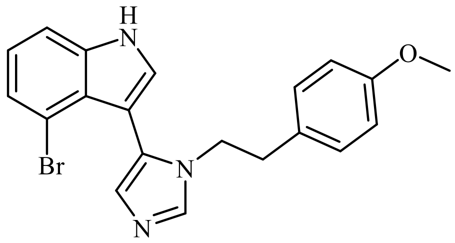

^{1}H NMR ($CDCl_3$, 400 MHz):

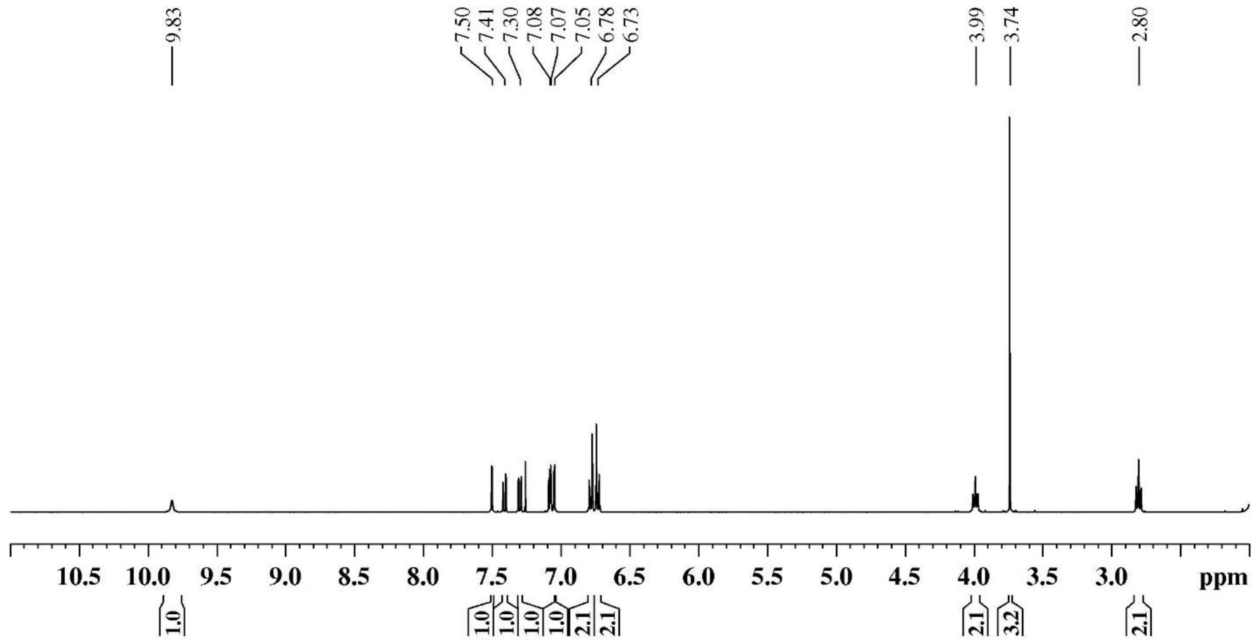

^{13}C NMR ($CDCl_3$, 100 MHz):

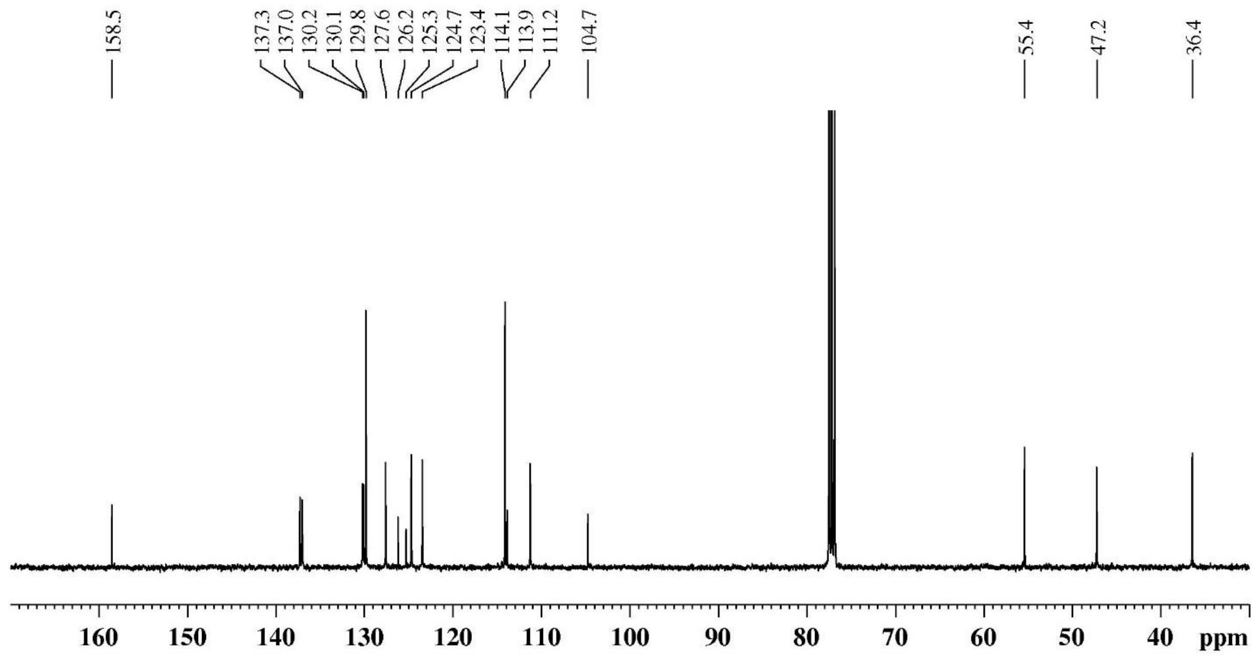

Figure S71. 5-Methoxy-3-(1-(4-methoxyphenethyl)-1*H*-imidazol-5-yl)-1*H*-indole (**71**)

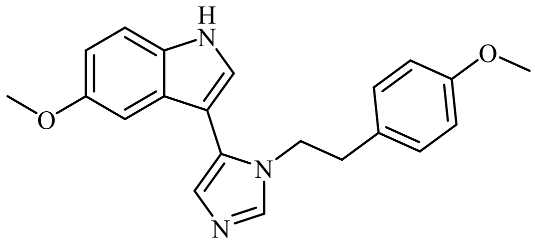

^{1}H NMR ($CDCl_3$, 400 MHz):

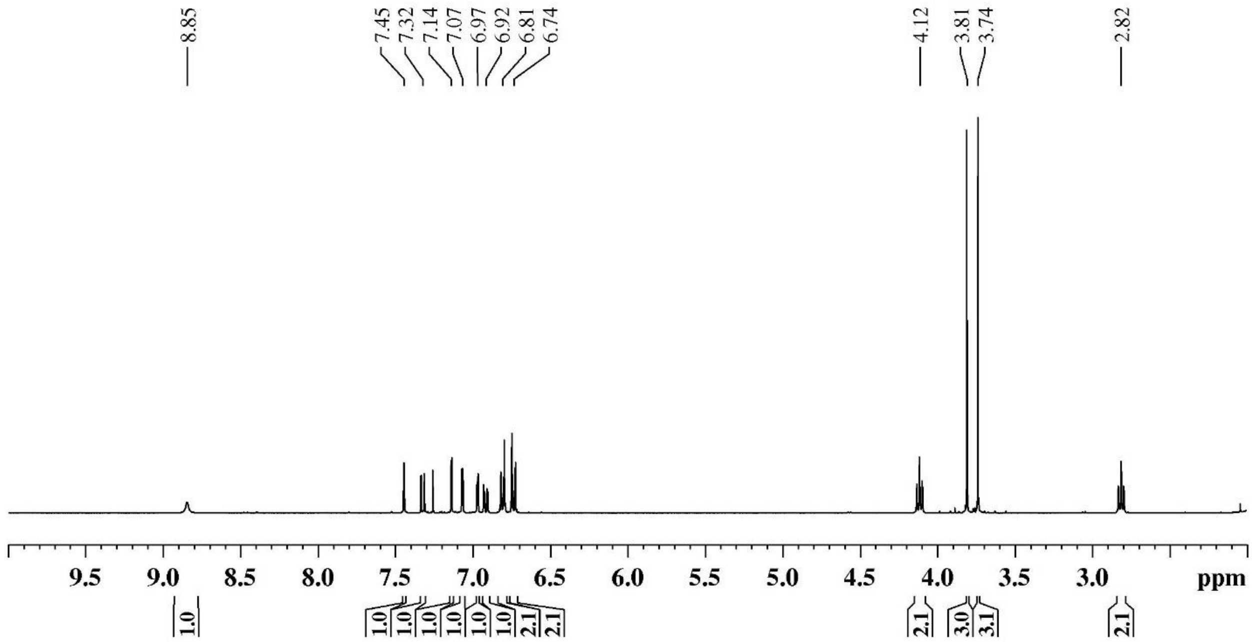

^{13}C NMR ($CDCl_3$, 100 MHz):

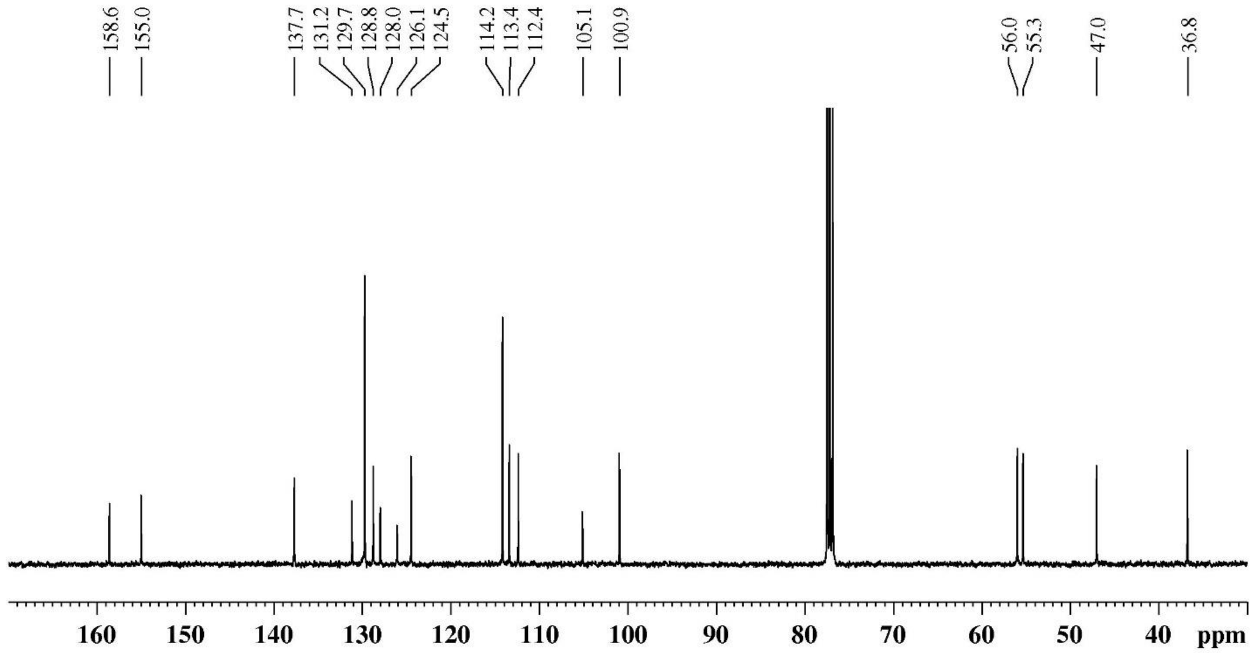

Figure S72. 6-Methoxy-3-(1-(4-methoxyphenethyl)-1*H*-imidazol-5-yl)-1*H*-indole (**72**)

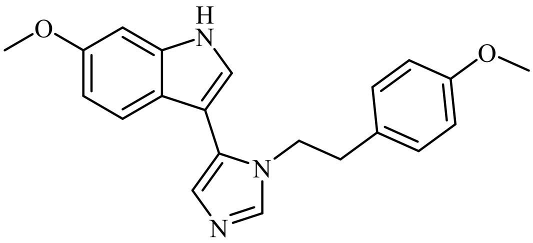

^{1}H NMR ($CDCl_3$, 400 MHz):

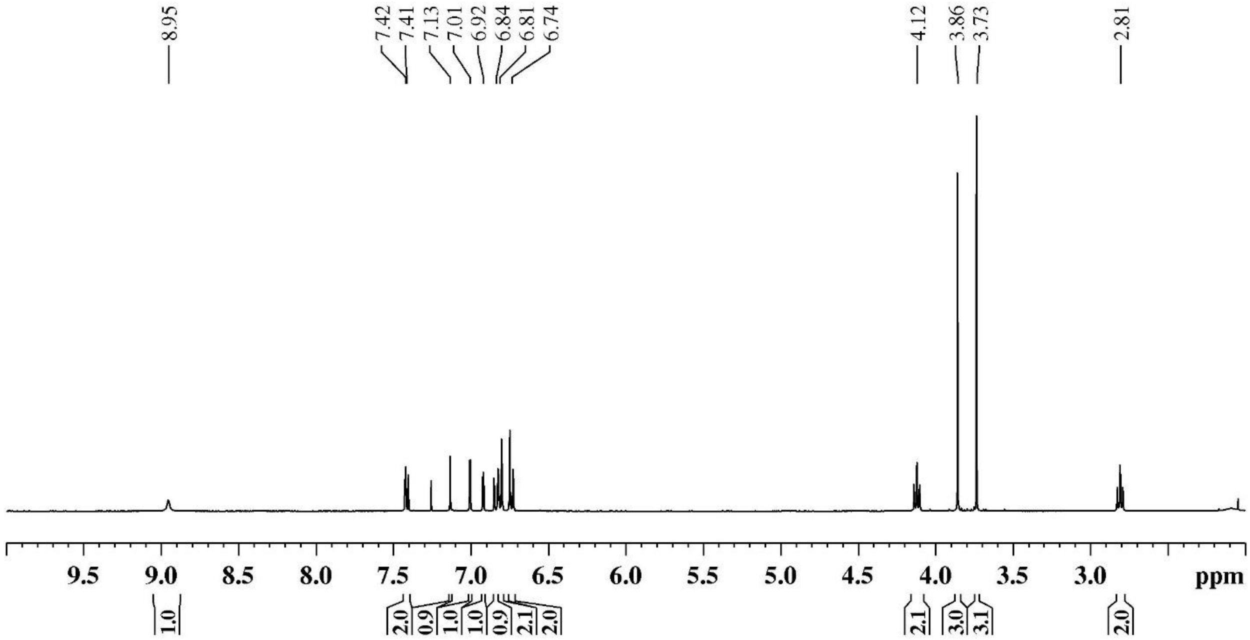

^{13}C NMR ($CDCl_3$, 100 MHz):

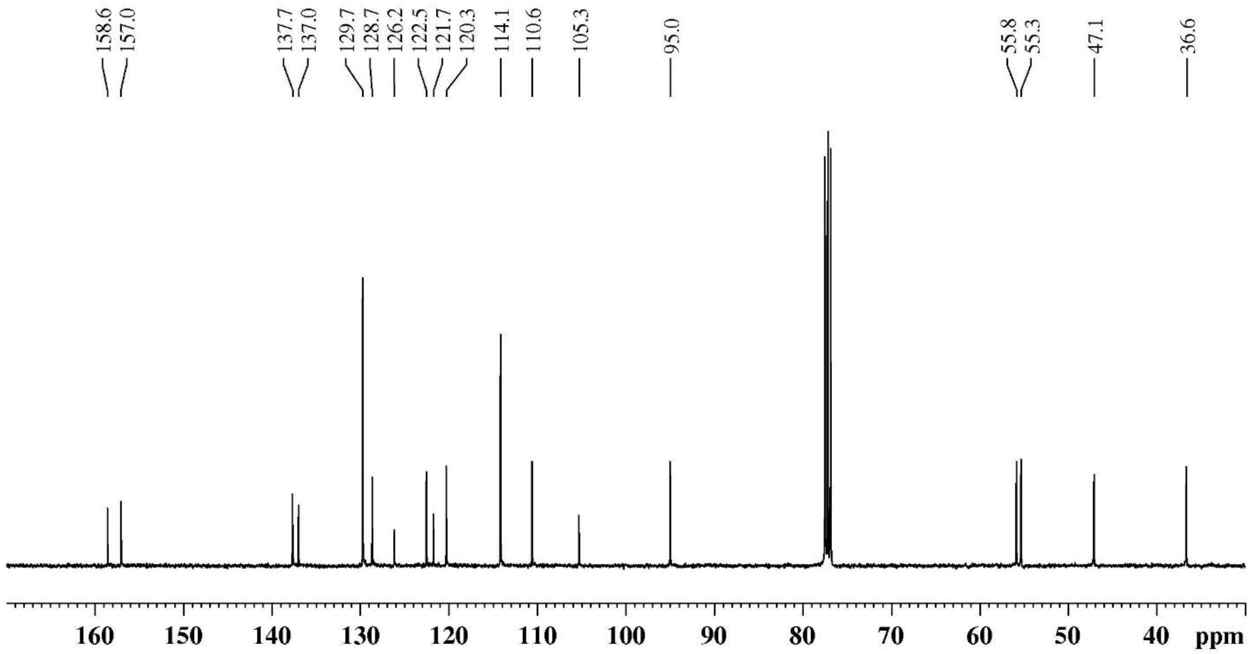

Figure S73. 3-(1-((1*H*-Indol-3-yl)methyl)-1*H*-imidazol-5-yl)-4-fluoro-1*H*-indole (**73**)

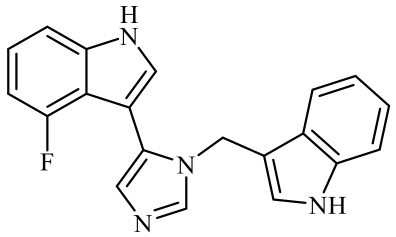

^{1}H NMR (DMSO-d_6, 400 MHz):

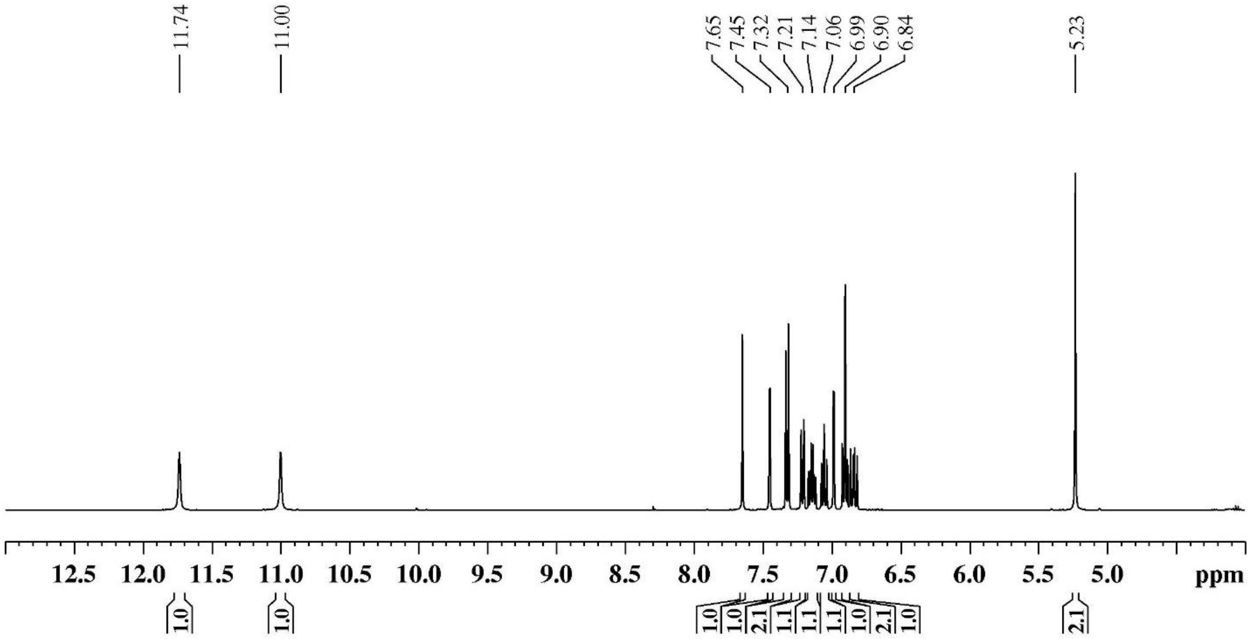

^{13}C NMR (DMSO-d_6, 100 MHz):

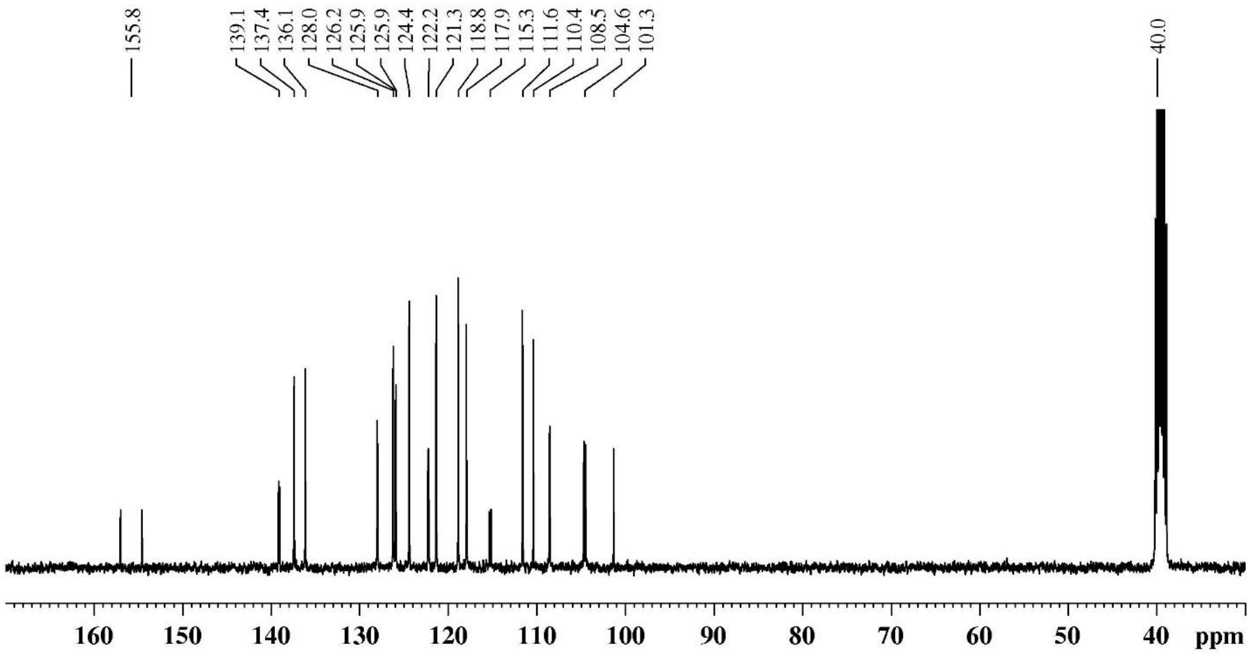

Figure S74. 3-(1-((1*H*-Indol-3-yl)methyl)-1*H*-imidazol-5-yl)-7-fluoro-1*H*-indole (**74**)

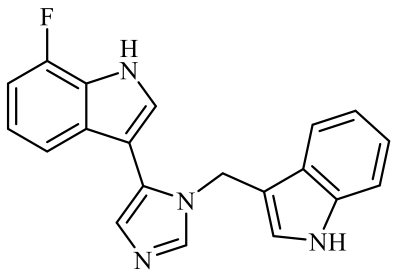

^{1}H NMR (DMSO-d_6, 400 MHz):

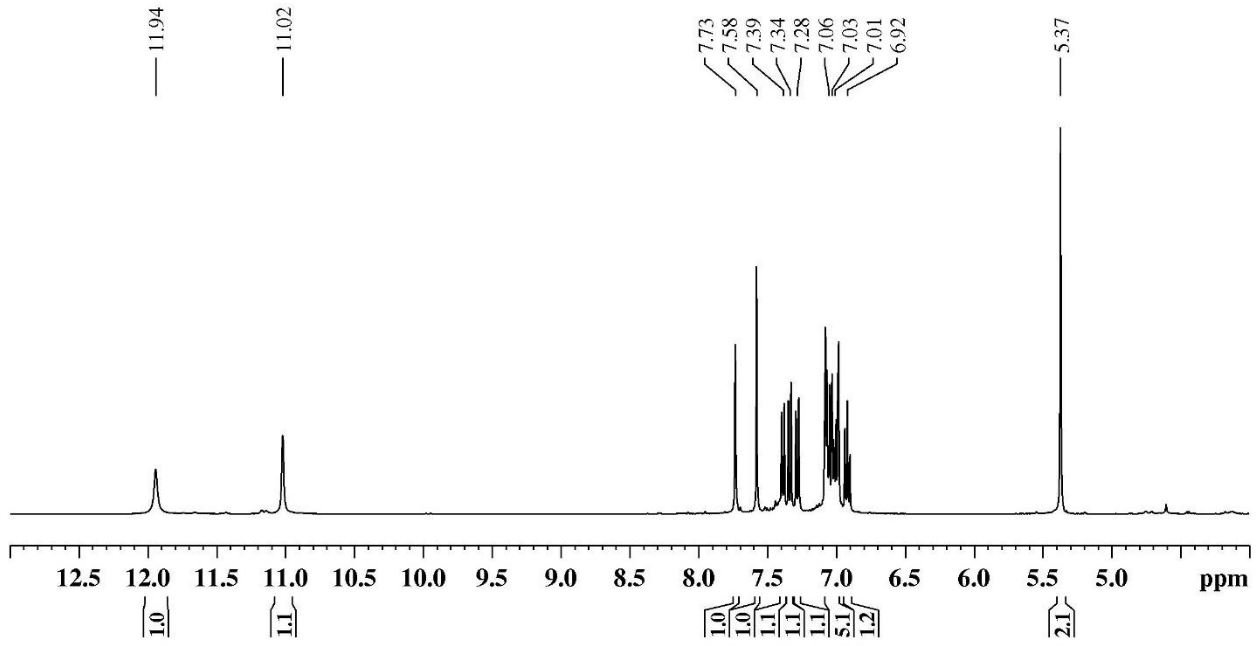

^{13}C NMR (DMSO-d_6, 100 MHz):

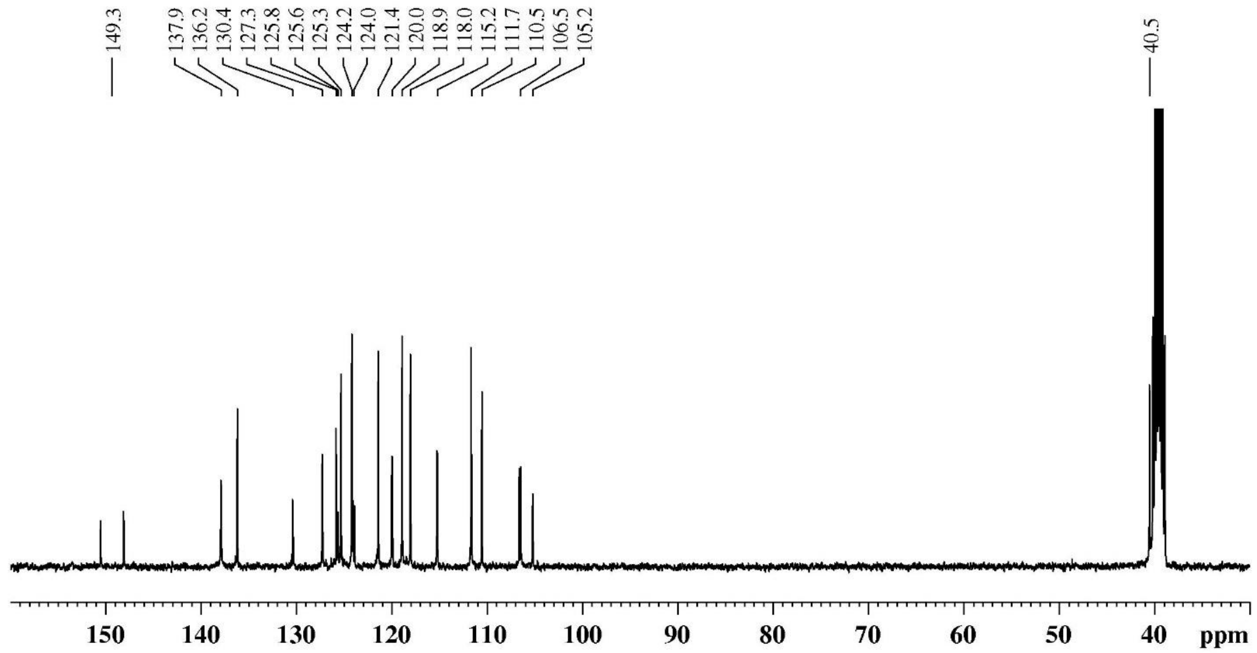

Figure S75. 3-(1-((1*H*-Indol-3-yl)methyl)-1*H*-imidazol-5-yl)-4-chloro-1*H*-indole (**75**)

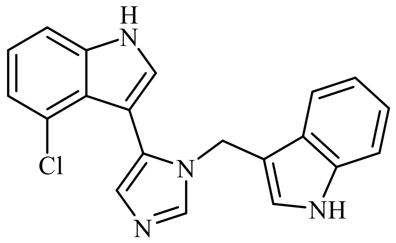

^{1}H NMR (DMSO-d_6, 400 MHz):

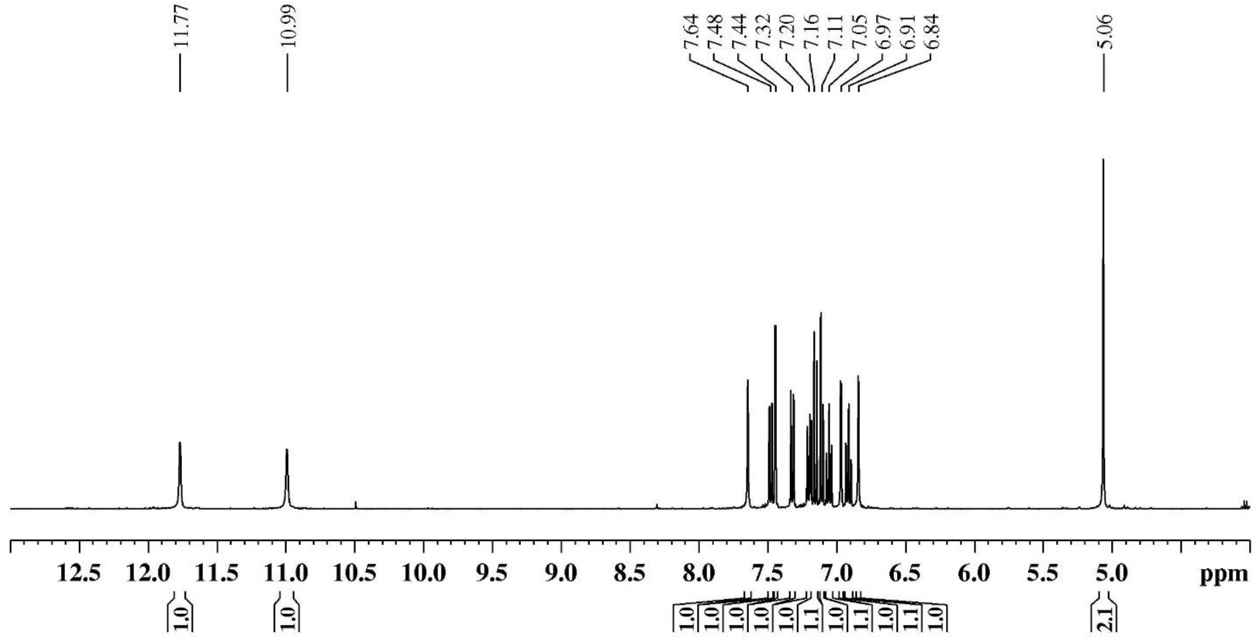

^{13}C NMR (DMSO-d_6, 100 MHz):

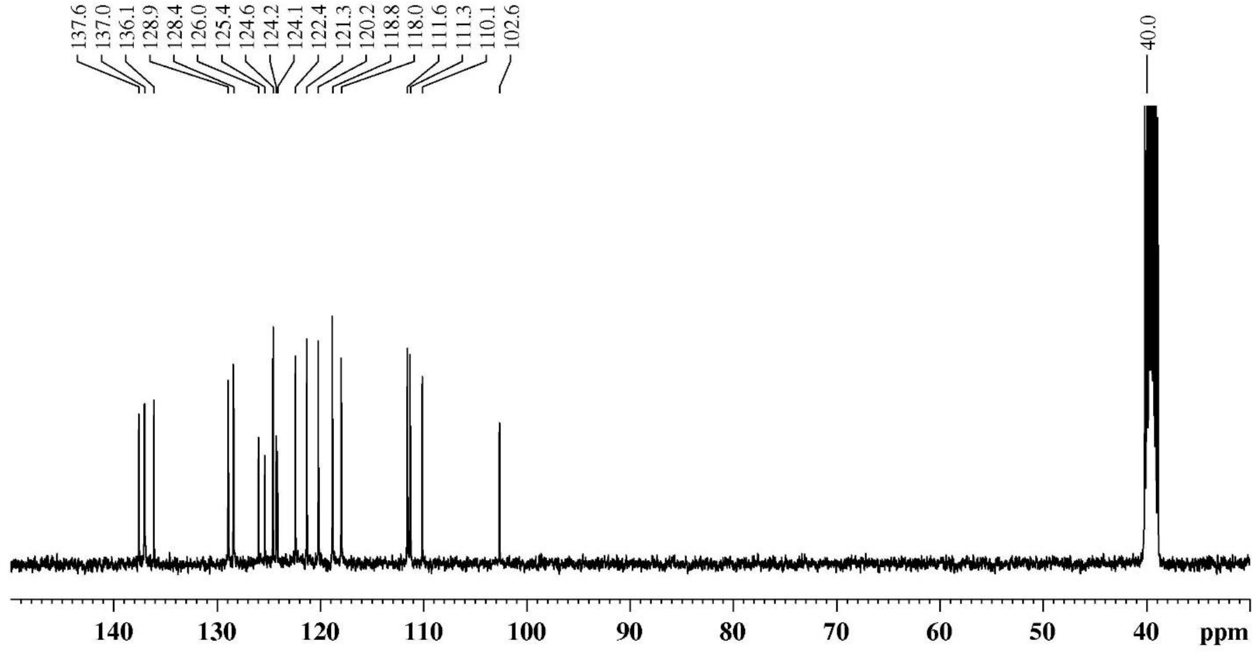

Figure S76. 3-(1-((1*H*-Indol-3-yl)methyl)-1*H*-imidazol-5-yl)-5-chloro-1*H*-indole (**76**)

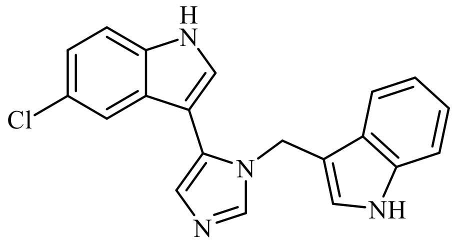

^{1}H NMR (DMSO-d_6, 400 MHz):

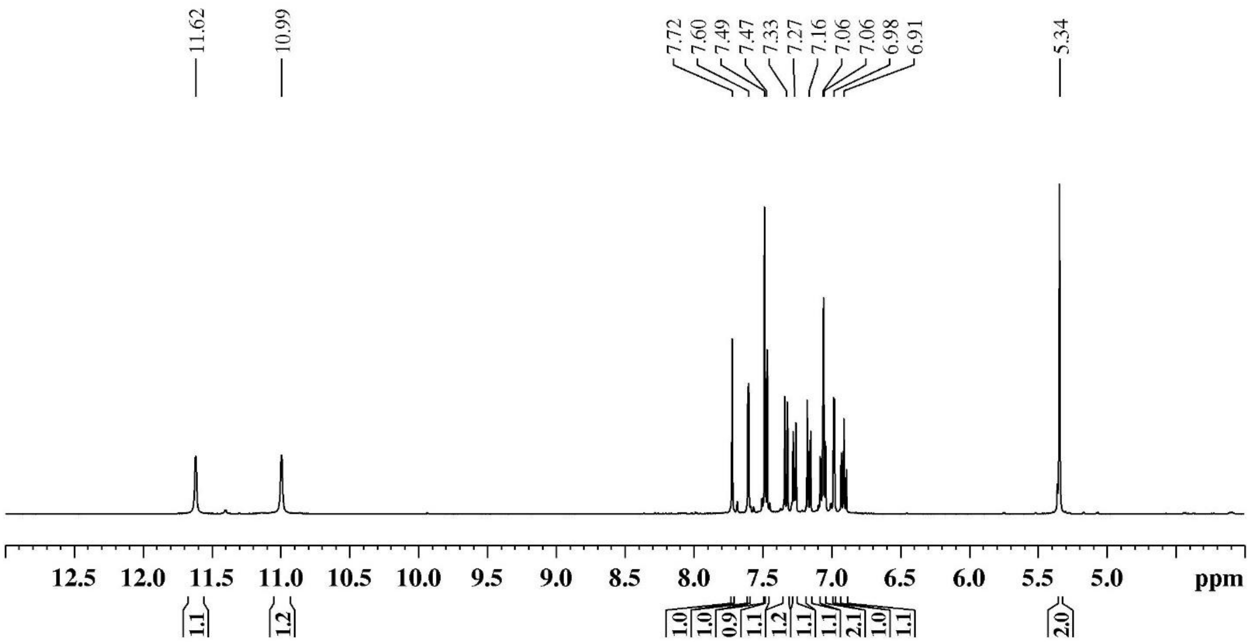

^{13}C NMR (DMSO-d_6, 100 MHz):

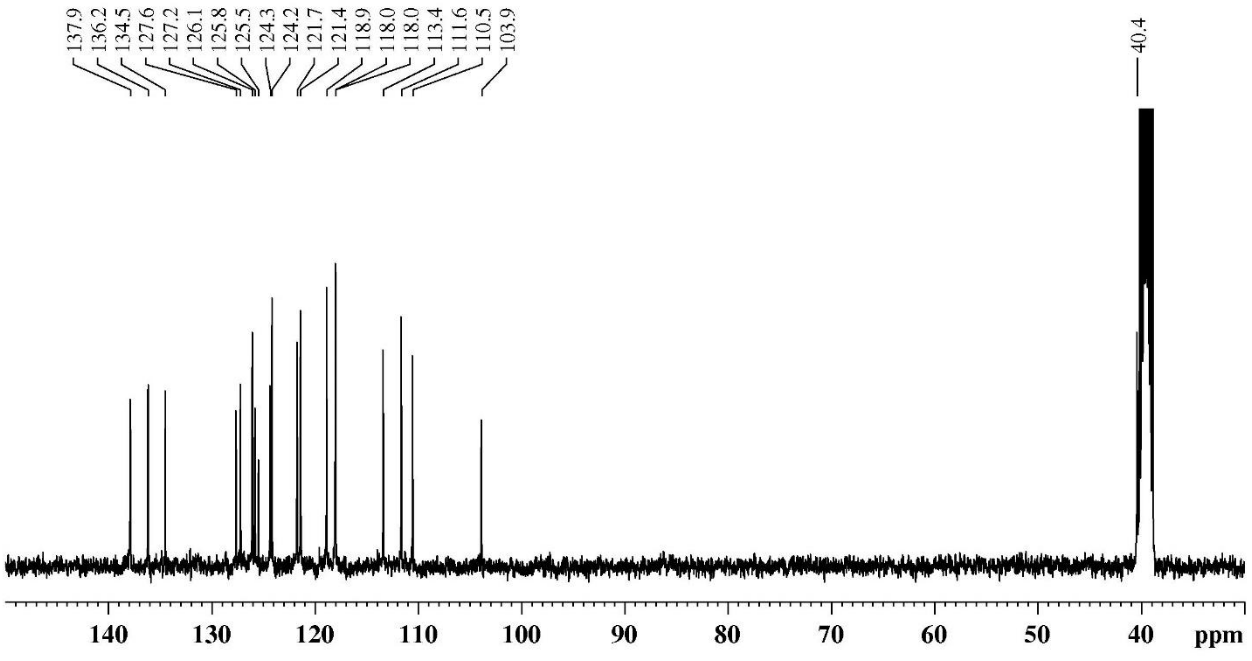

Figure S77. 3-(1-((1*H*-Indol-3-yl)methyl)-1*H*-imidazol-5-yl)-7-chloro-1*H*-indole (**77**)

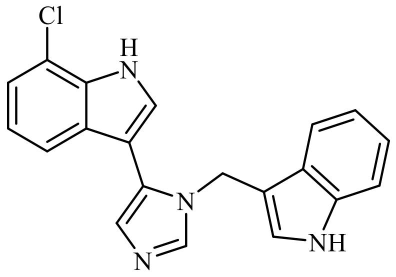

^{1}H NMR (DMSO-d_6, 400 MHz):

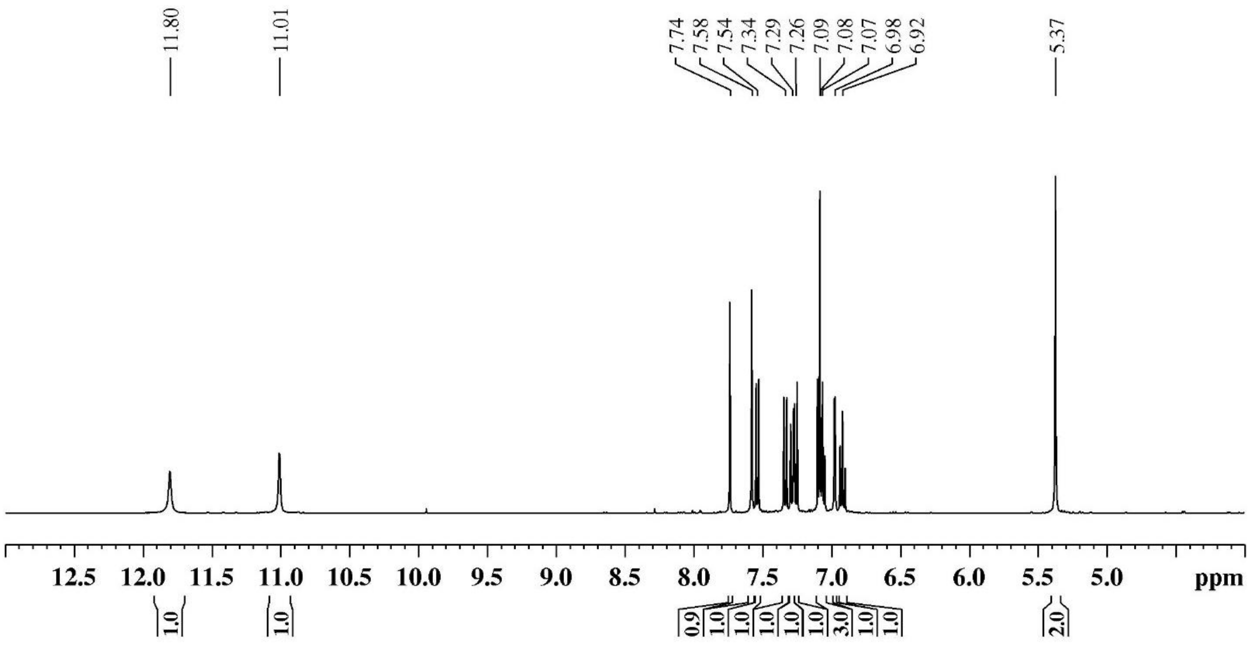

^{13}C NMR (DMSO-d_6, 100 MHz):

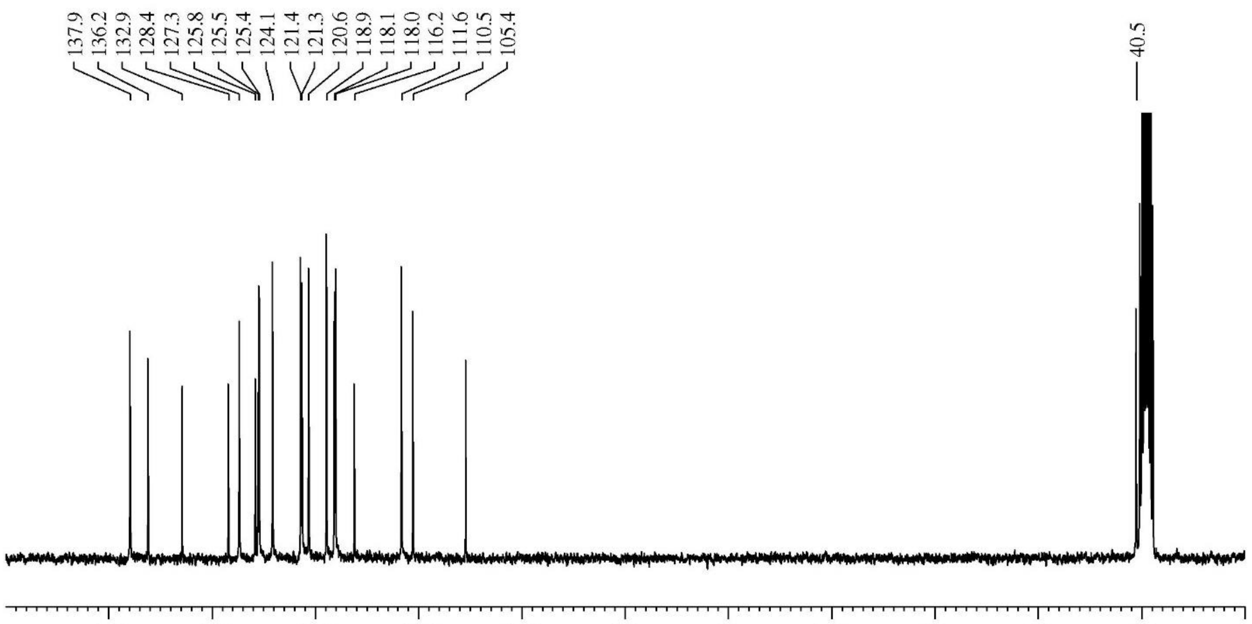

Figure S78. 3-(1-((1*H*-Indol-3-yl)methyl)-1*H*-imidazol-5-yl)-4-bromo-1*H*-indole (**78**)

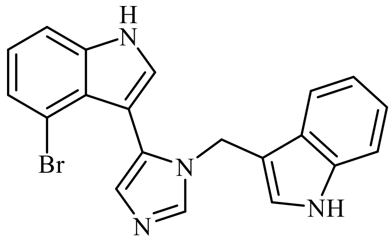

^{1}H NMR (DMSO-d_6, 400 MHz):

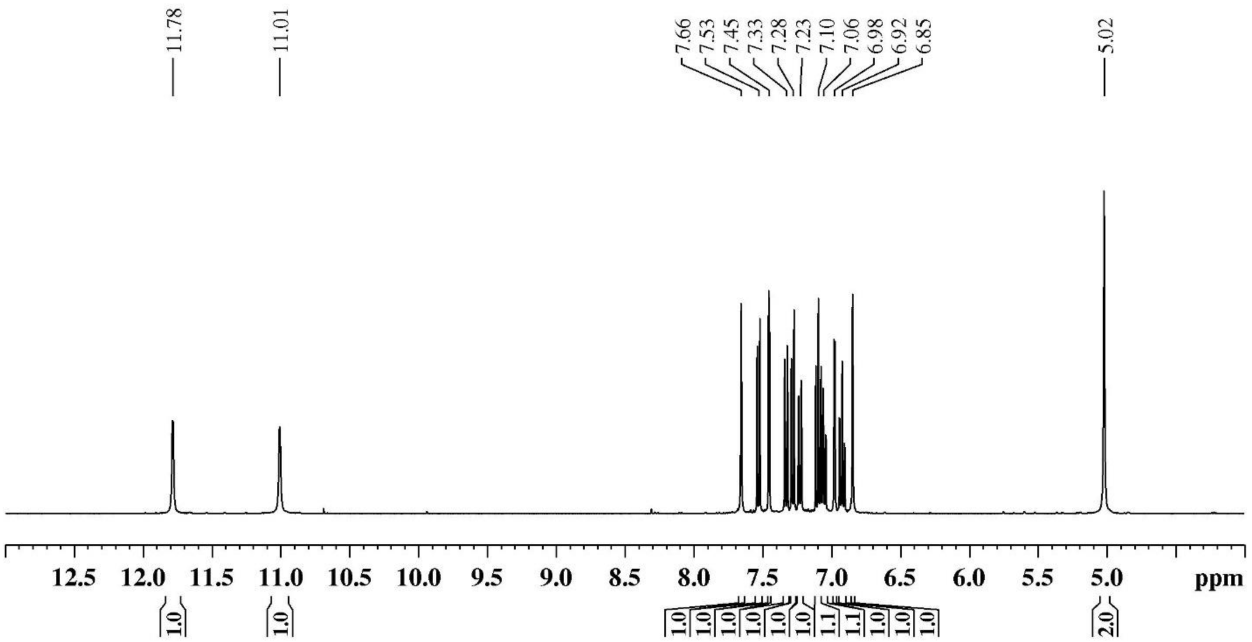

^{13}C NMR (DMSO-d_6, 100 MHz):

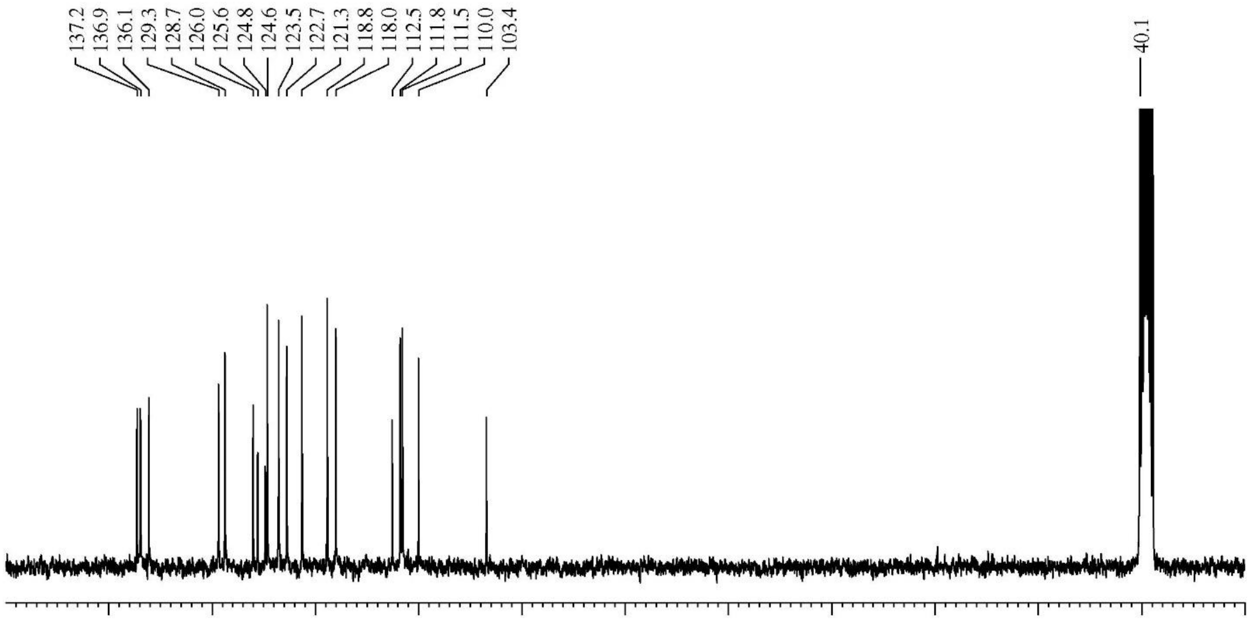

Figure S79. 3-(1-((1*H*-Indol-3-yl)methyl)-1*H*-imidazol-5-yl)-5-methoxy-1*H*-indole (**79**)

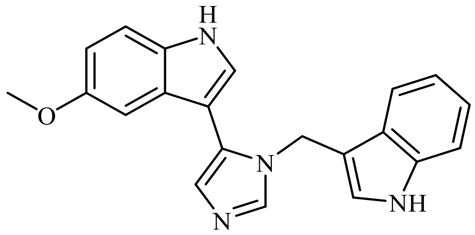

^{1}H NMR ($CDCl_3$, 400 MHz):

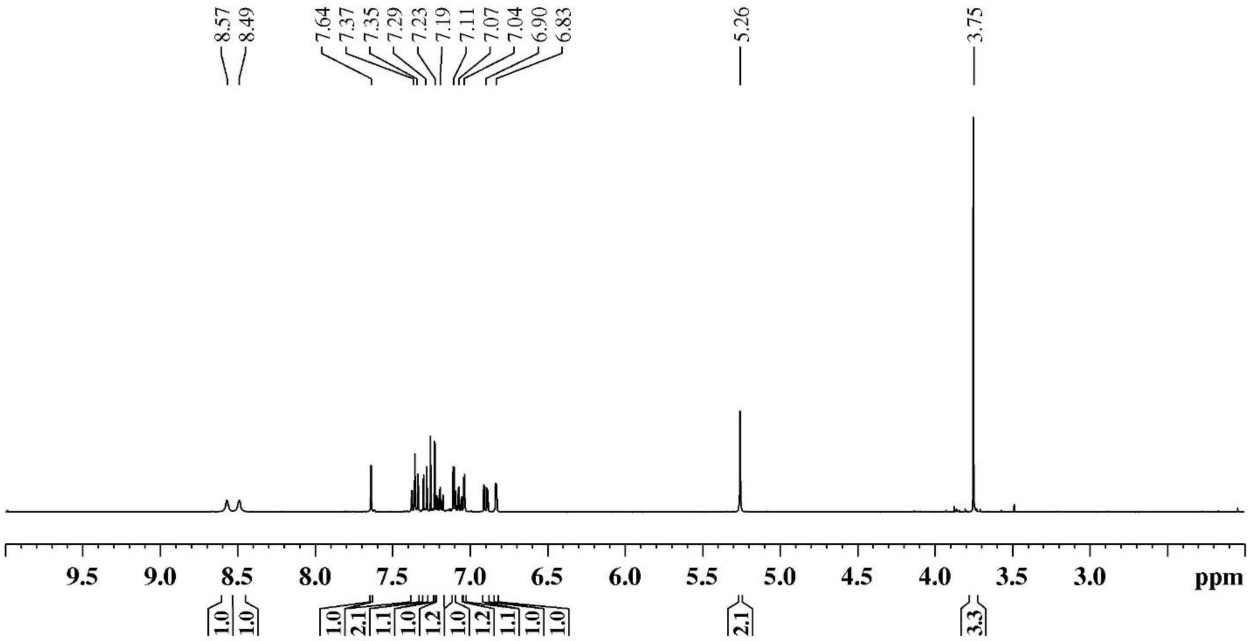

^{13}C NMR ($CDCl_3$, 100 MHz):

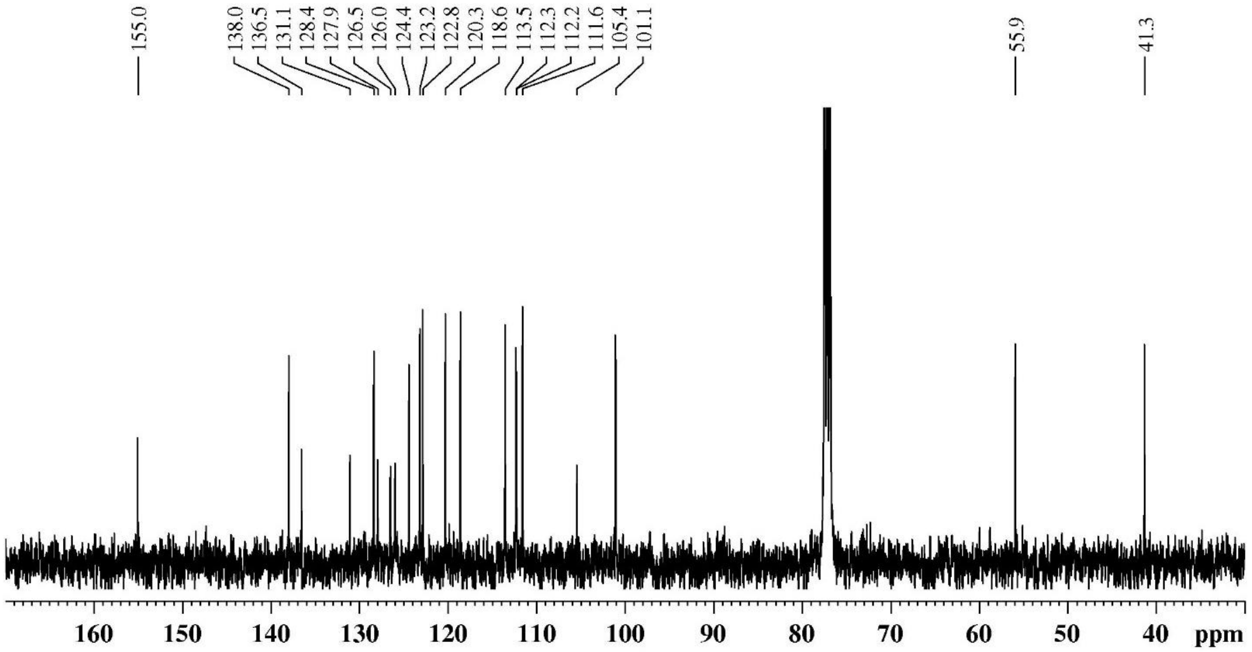

__Figure S80.__ 3-(1-((1*H*-Indol-3-yl)methyl)-1*H*-imidazol-5-yl)-6-methoxy-1*H*-indole (**80**)

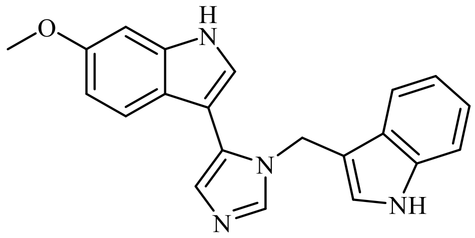

^{1}H NMR ($CDCl_3$, 400 MHz):

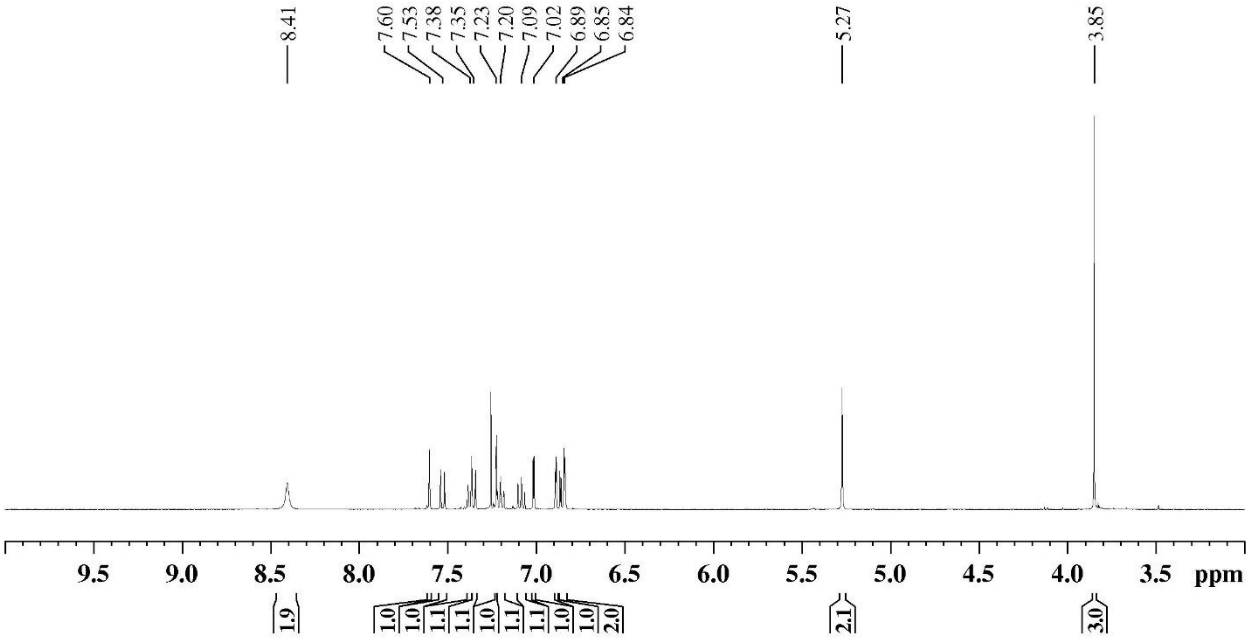

^{13}C NMR ($CDCl_3$, 100 MHz):

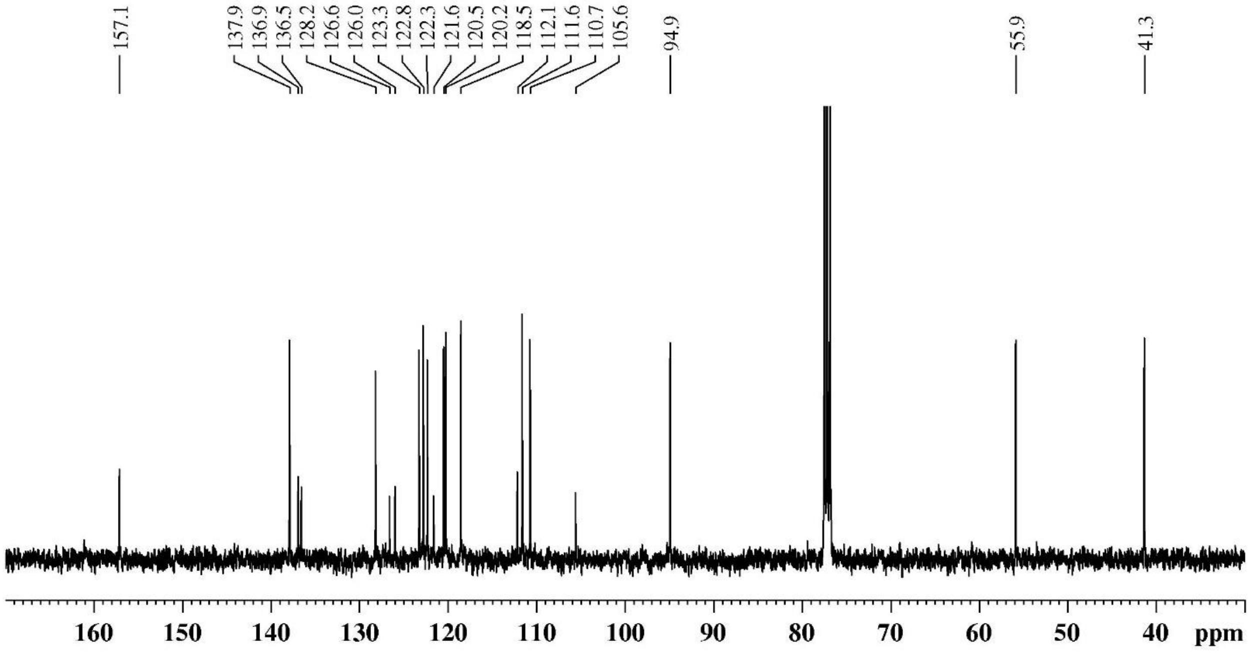

Figure S81. 3-(1-(2-(1*H*-Indol-3-yl)ethyl)-1*H*-imidazol-5-yl)-4-fluoro-1*H*-indole (**81**)

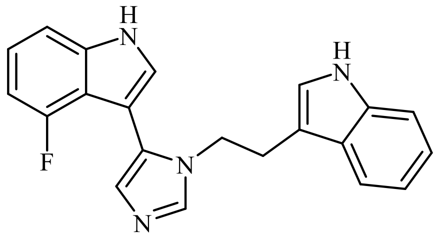

^{1}H NMR (DMSO-d_6, 400 MHz):

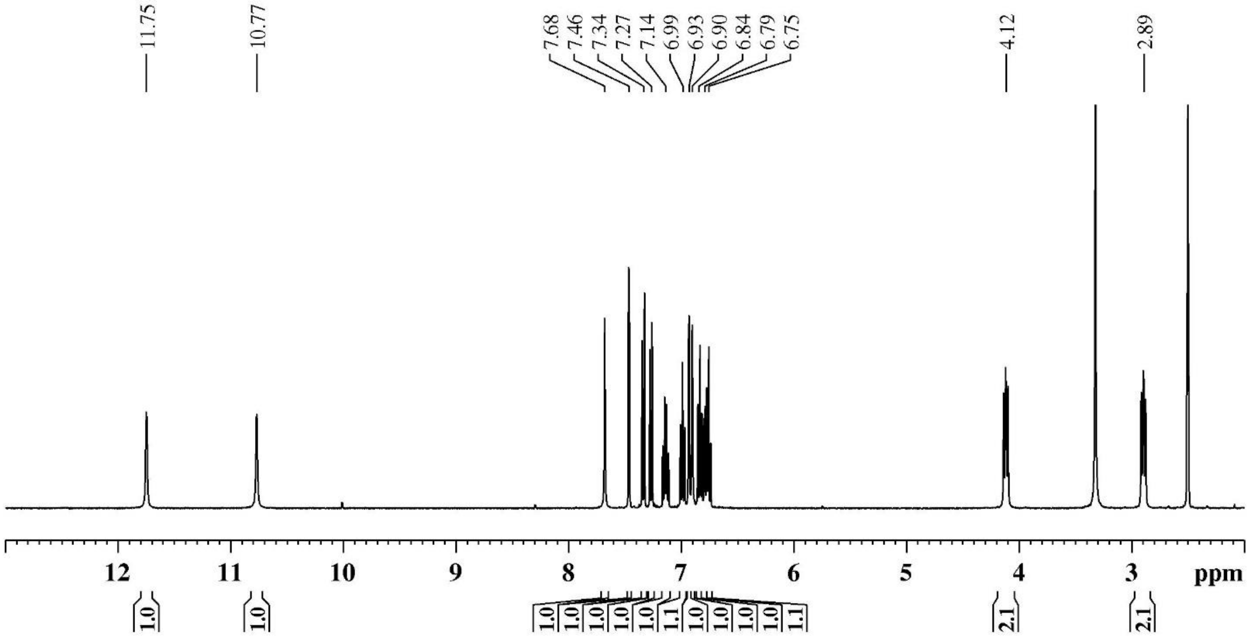

^{13}C NMR (DMSO-d_6, 100 MHz):

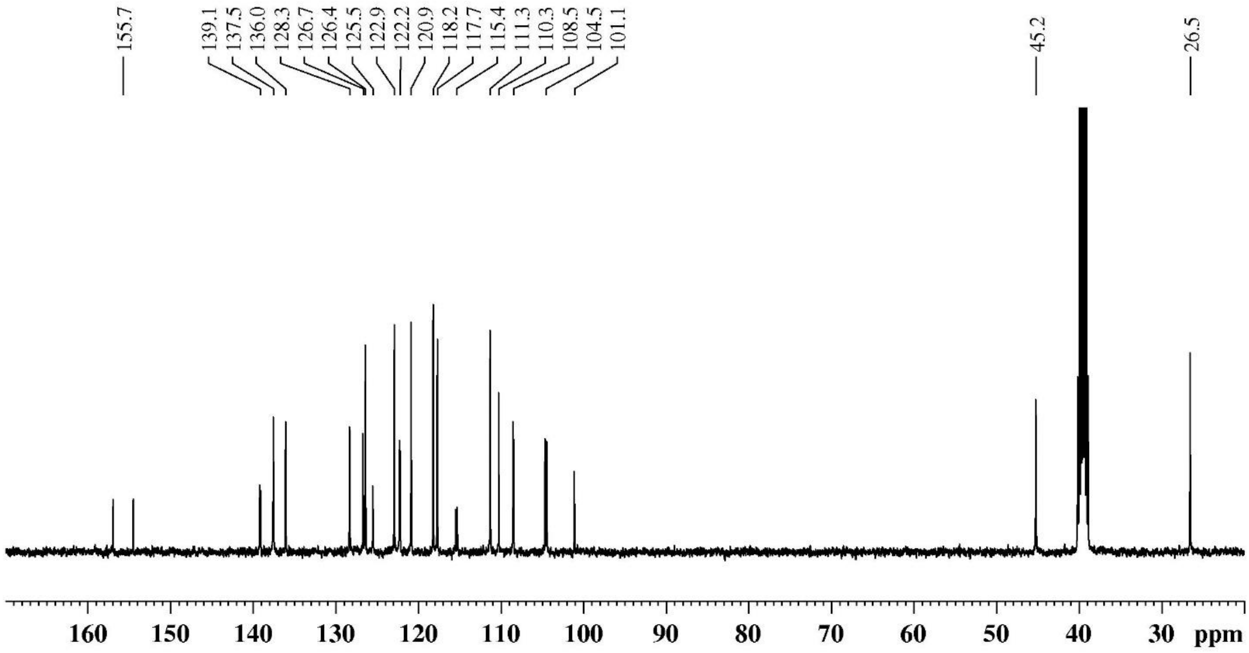

Figure S82. 3-(1-(2-(1*H*-Indol-3-yl)ethyl)-1*H*-imidazol-5-yl)-7-fluoro-1*H*-indole (**82**)

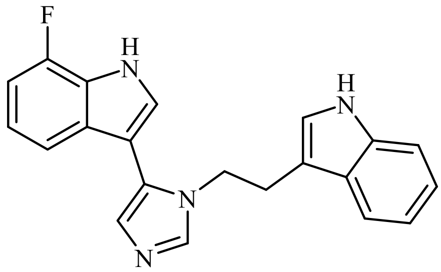

^{1}H NMR (DMSO-d_6, 400 MHz):

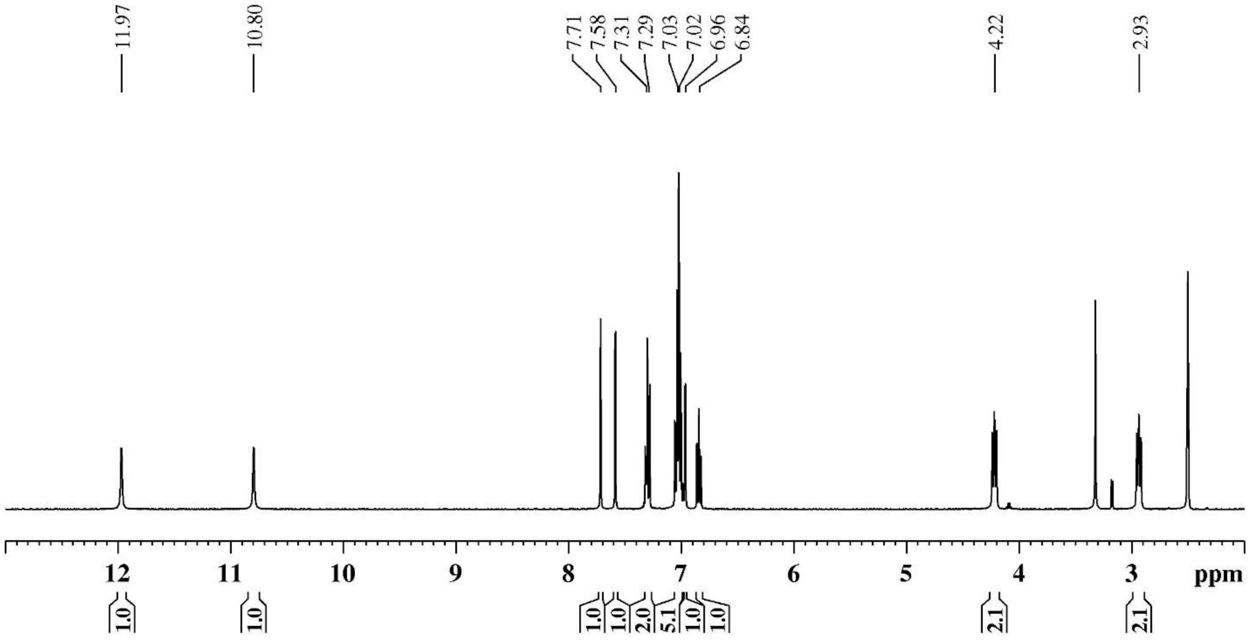

^{13}C NMR (DMSO-d_6, 100 MHz):

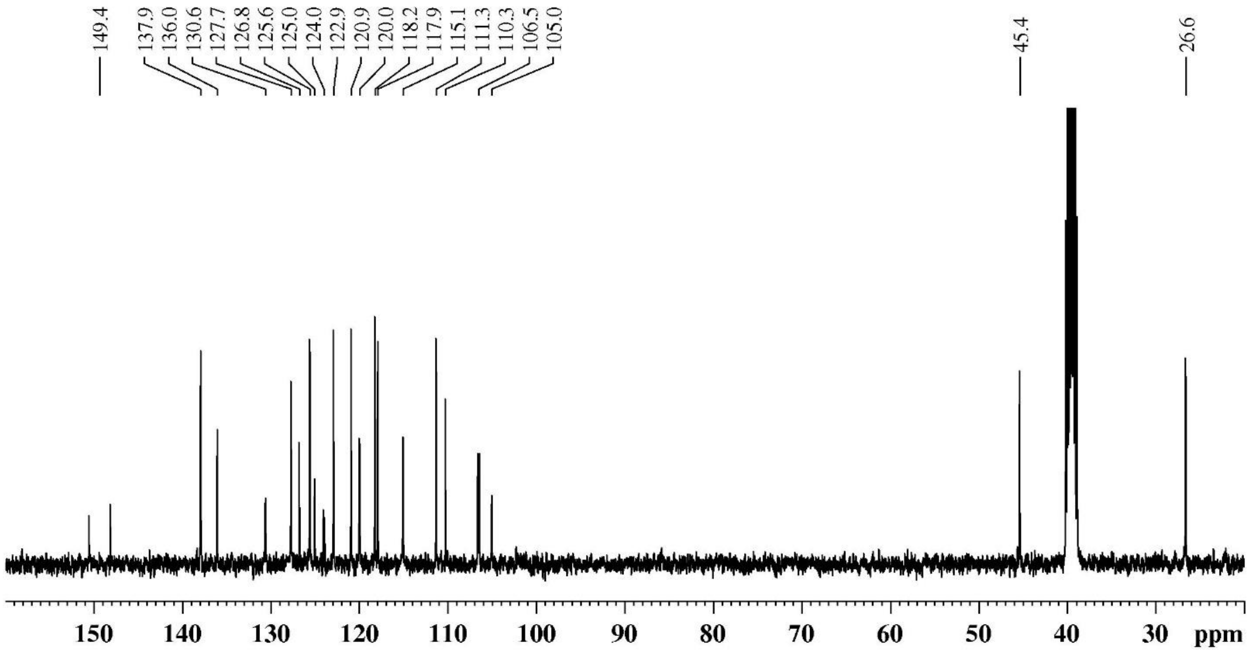

Figure S83. 3-(1-(2-(1*H*-Indol-3-yl)ethyl)-1*H*-imidazol-5-yl)-4-chloro-1*H*-indole (**83**)

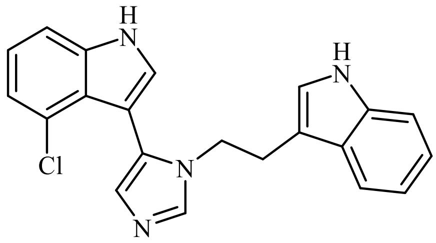

^{1}H NMR (DMSO-d_6, 400 MHz):

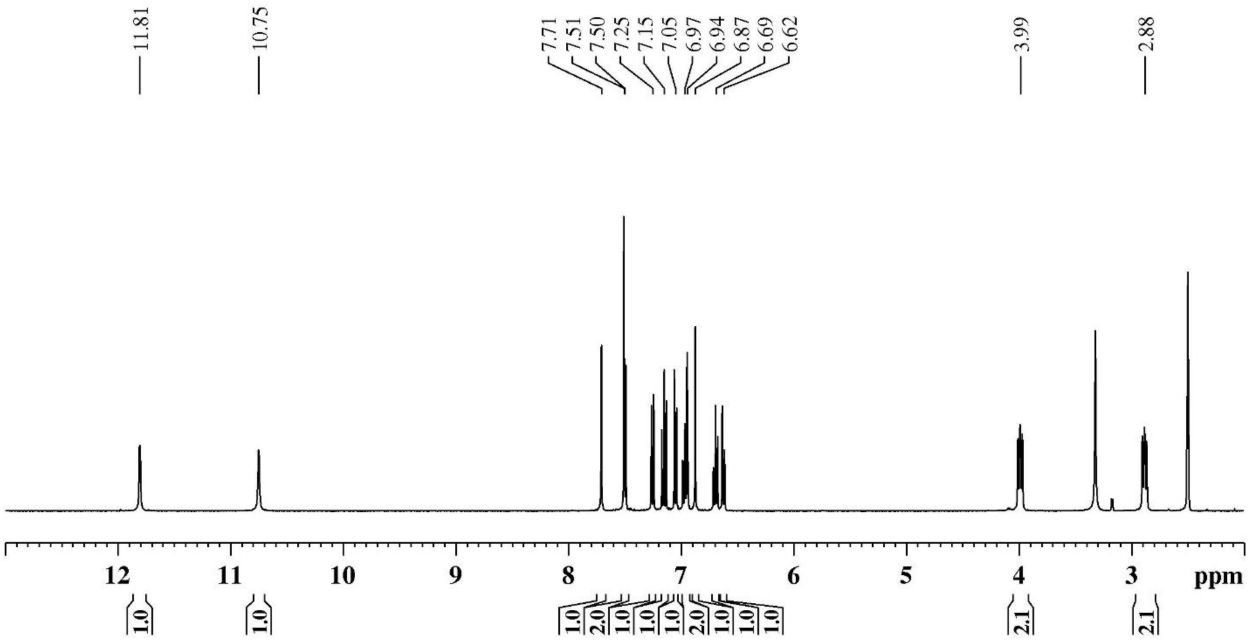

^{13}C NMR (DMSO-d_6, 100 MHz):

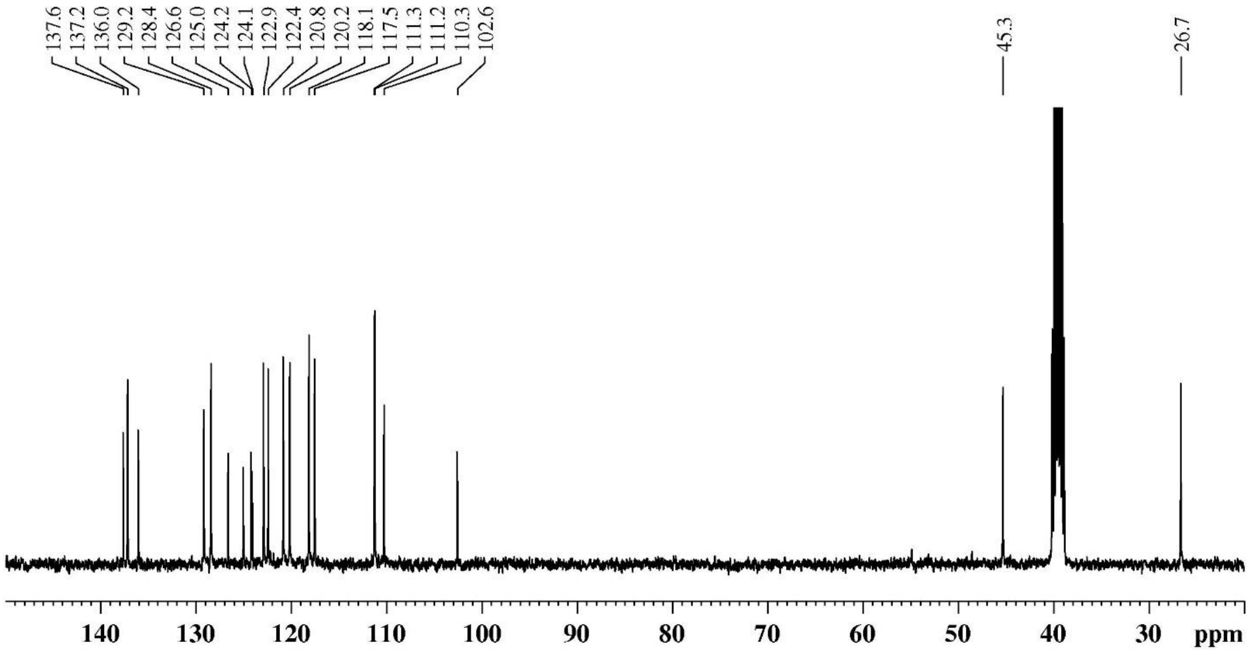

Figure S84. 3-(1-(2-(1*H*-Indol-3-yl)ethyl)-1*H*-imidazol-5-yl)-5-chloro-1*H*-indole (**84**)

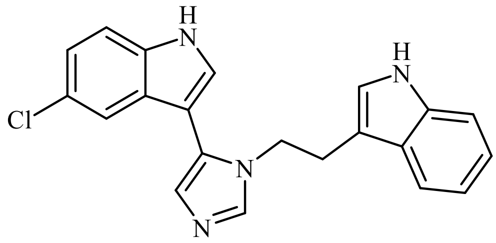

^{1}H NMR (DMSO-d_6, 400 MHz):

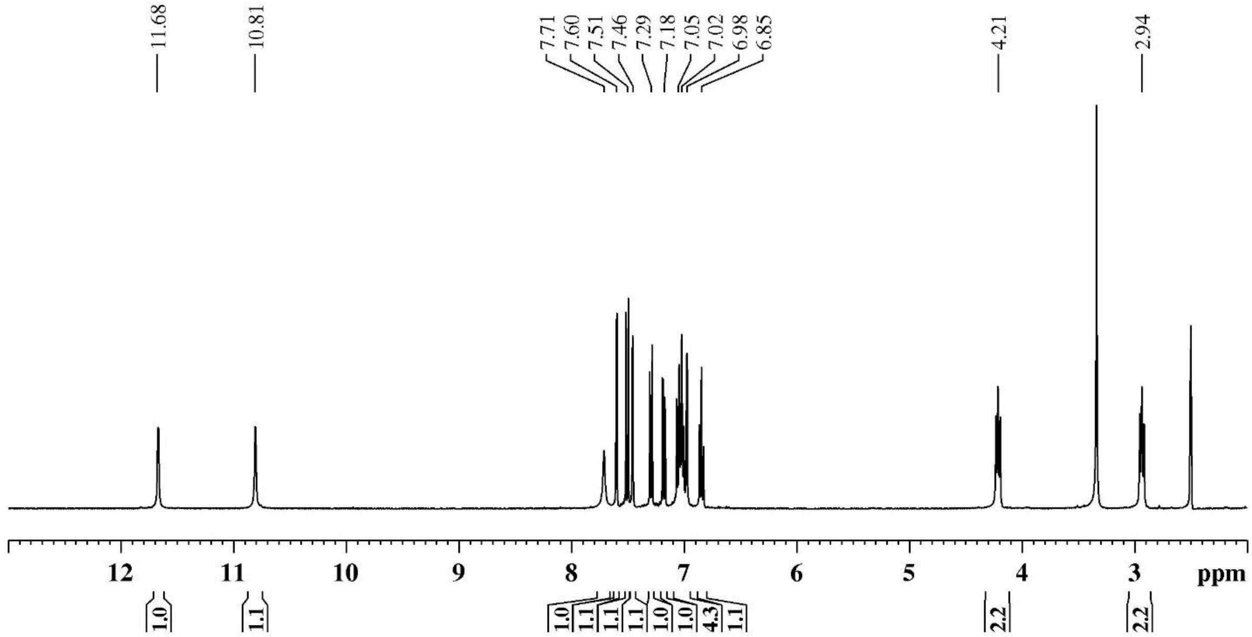

^{13}C NMR (DMSO-d_6, 100 MHz):

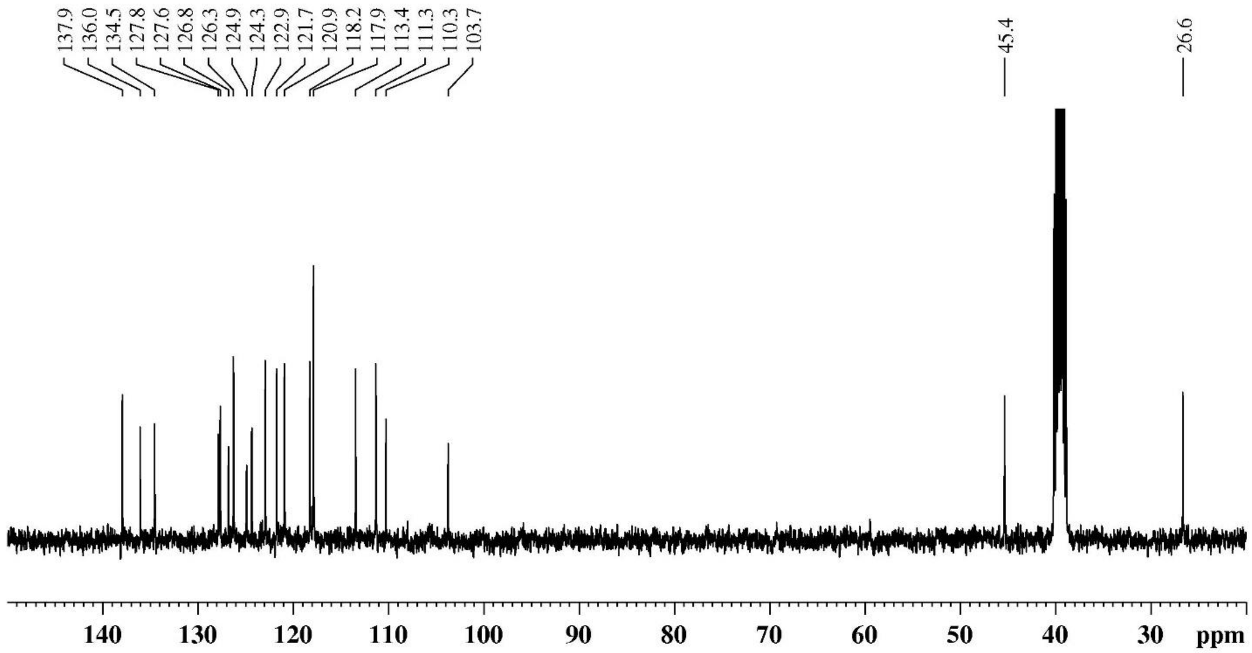

Figure S85. 3-(1-(2-(1*H*-Indol-3-yl)ethyl)-1*H*-imidazol-5-yl)-7-chloro-1*H*-indole (**85**)

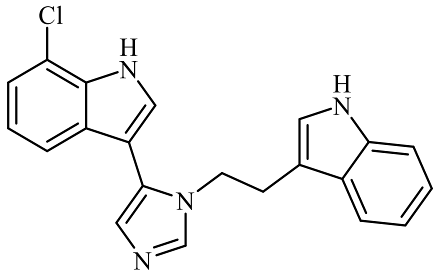

^{1}H NMR (DMSO-d_6, 400 MHz):

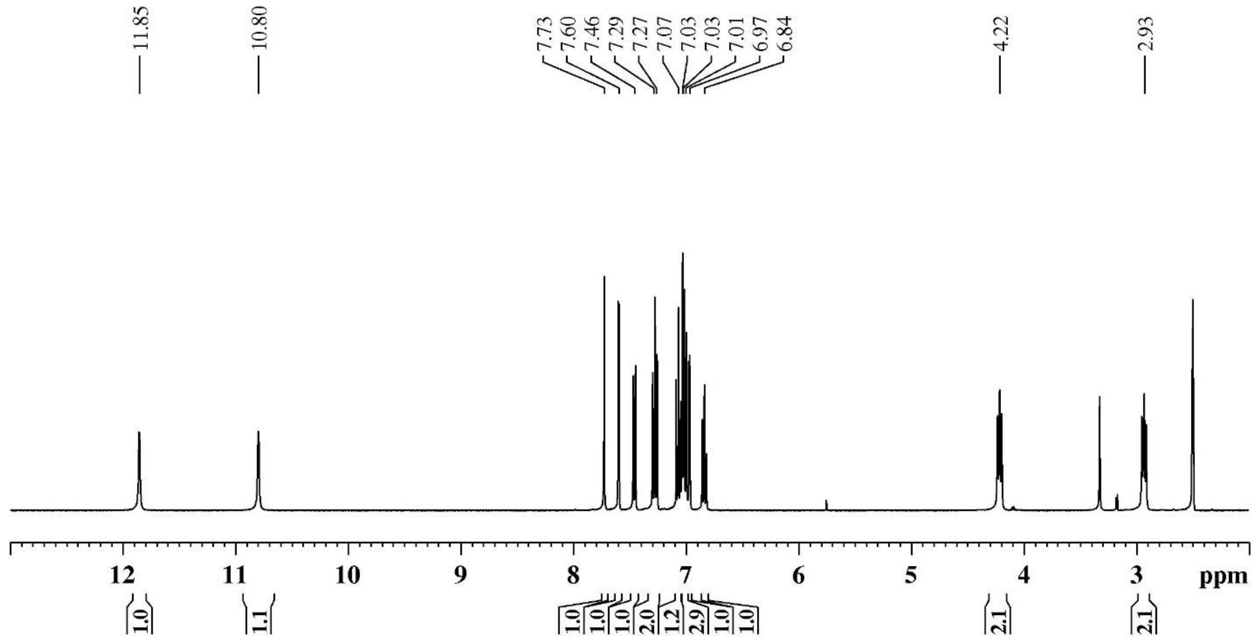

^{13}C NMR (DMSO-d_6, 100 MHz):

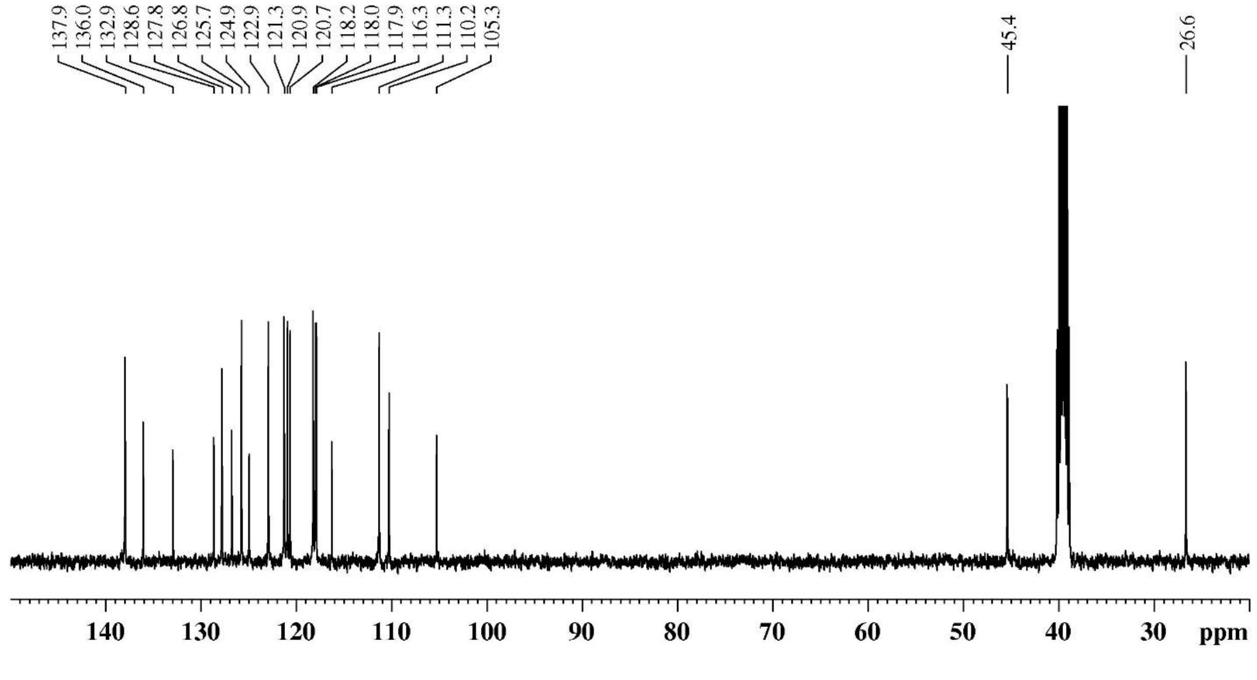

Figure S86. 3-(1-(2-(1*H*-Indol-3-yl)ethyl)-1*H*-imidazol-5-yl)-4-bromo-1*H*-indole (**86**)

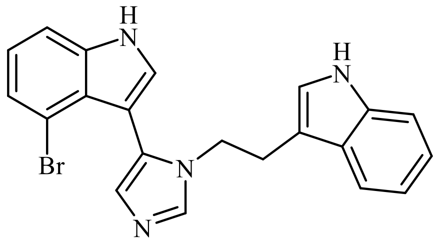

^{1}H NMR (DMSO-d_6, 400 MHz):

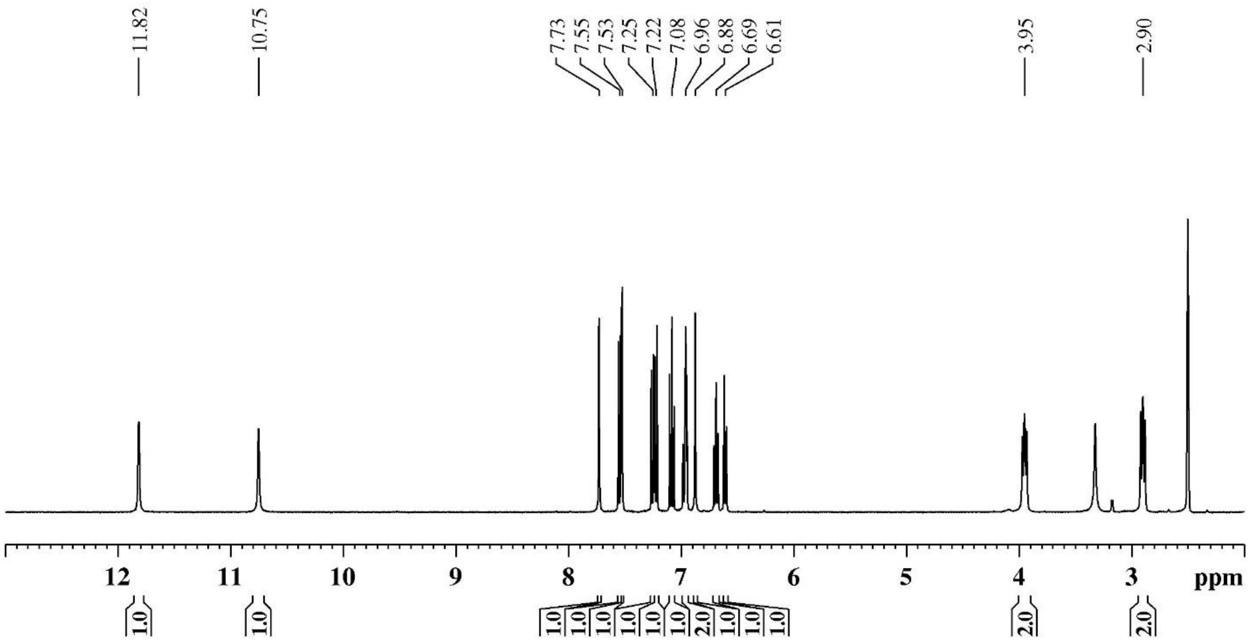

^{13}C NMR (DMSO-d_6, 100 MHz):

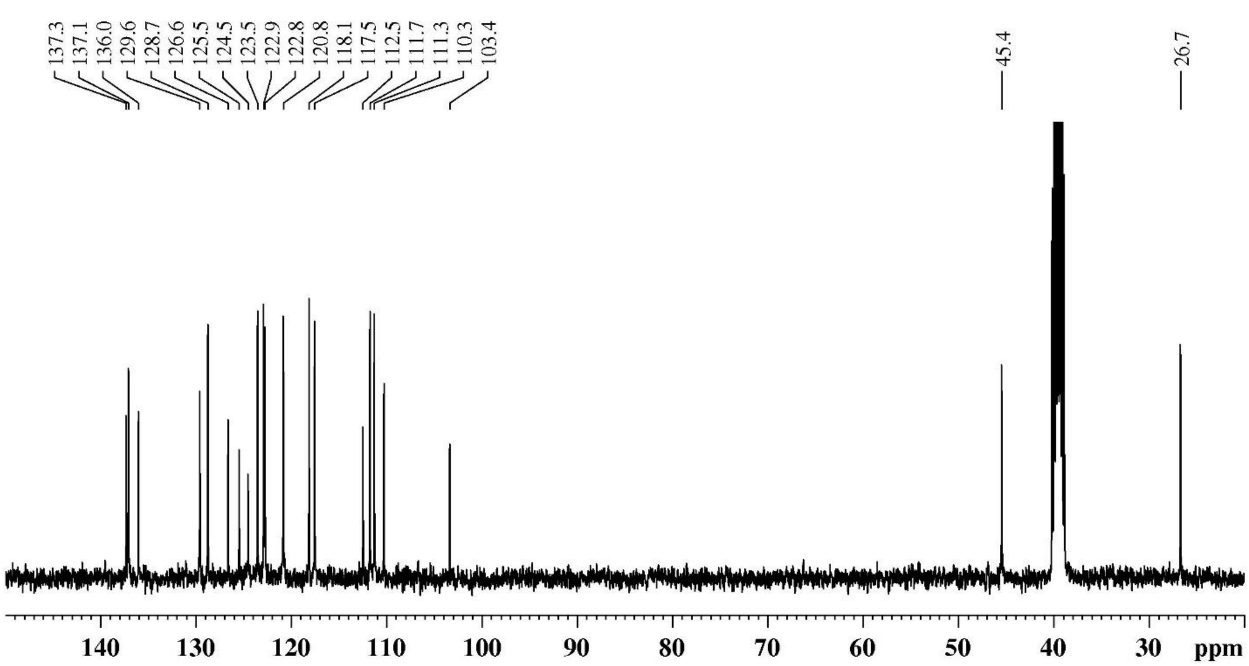

Figure S87. 3-(1-(2-(1*H*-Indol-3-yl)ethyl)-1*H*-imidazol-5-yl)-5-methoxy-1*H*-indole (**87**)

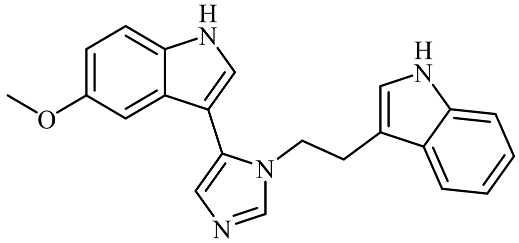

^{1}H NMR ($CDCl_3$, 400 MHz):

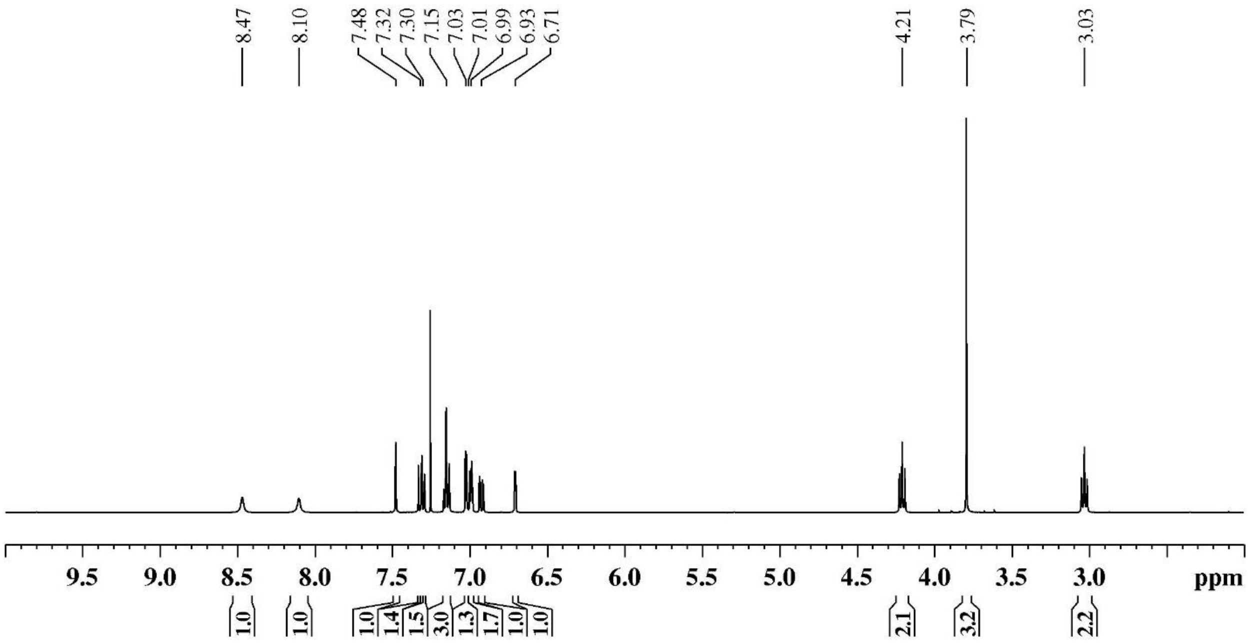

^{13}C NMR ($CDCl_3$, 100 MHz):

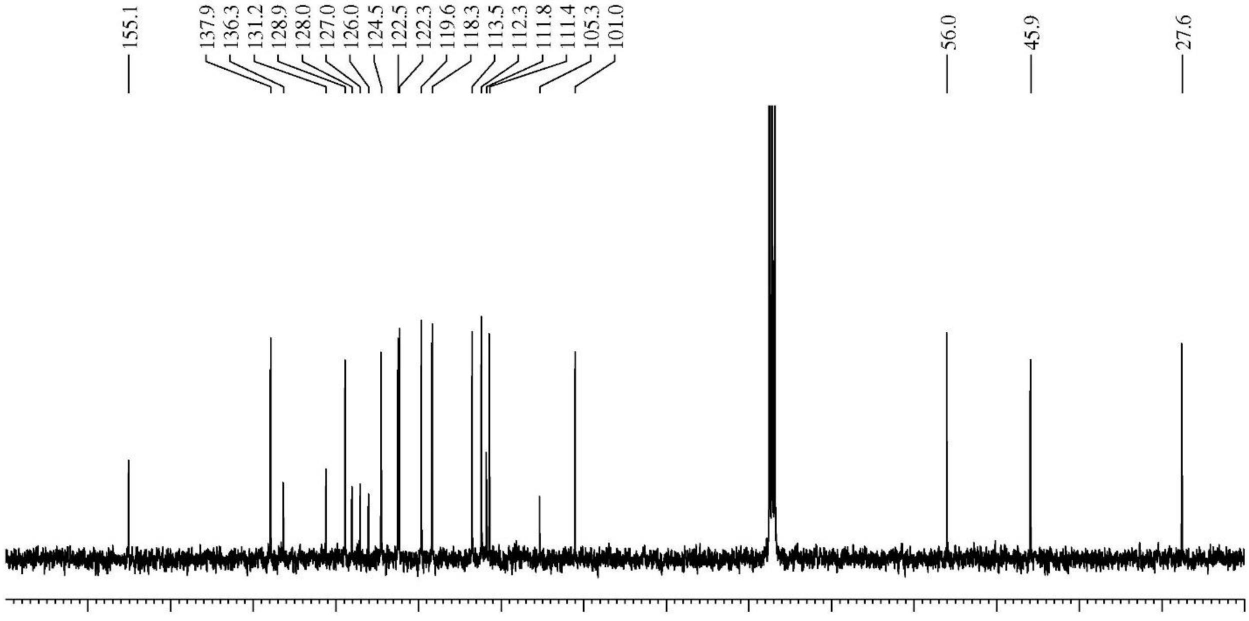

Figure S88. 3-(1-(2-(1*H*-Indol-3-yl)ethyl)-1*H*-imidazol-5-yl)-6-methoxy-1*H*-indole (**88**)

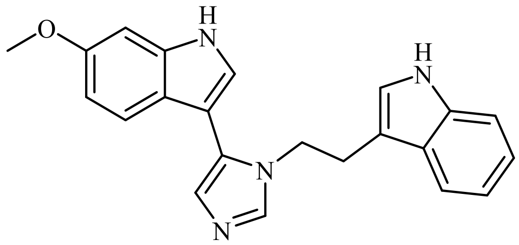

^{1}H NMR ($CDCl_3$, 400 MHz):

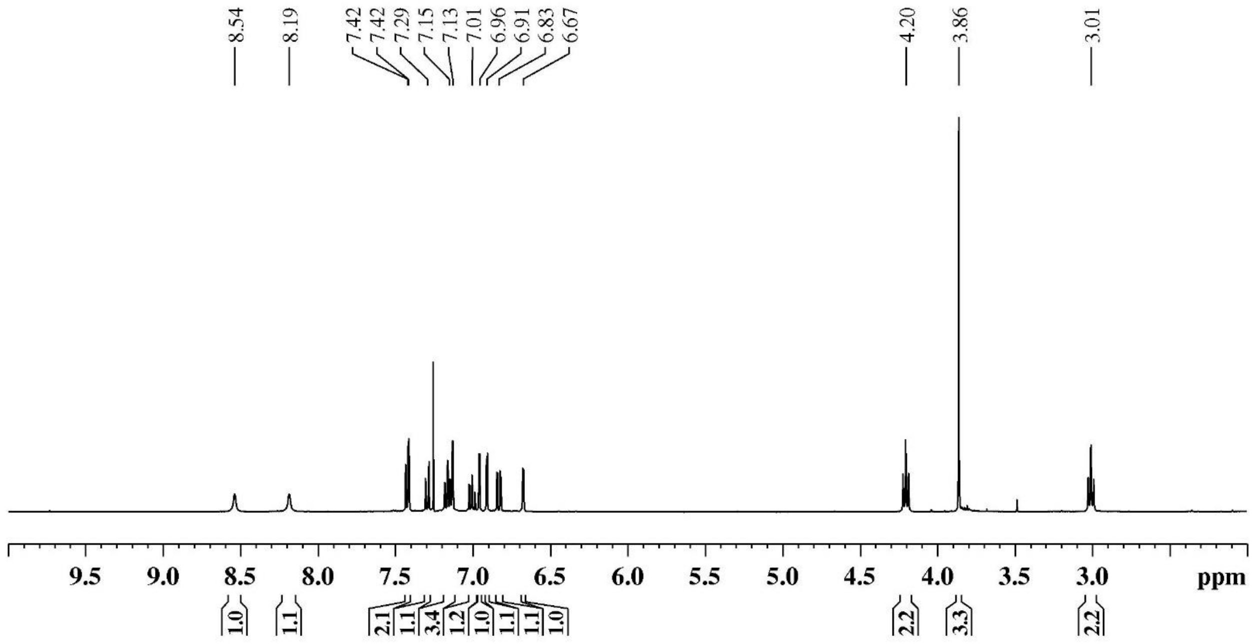

^{13}C NMR ($CDCl_3$, 100 MHz):

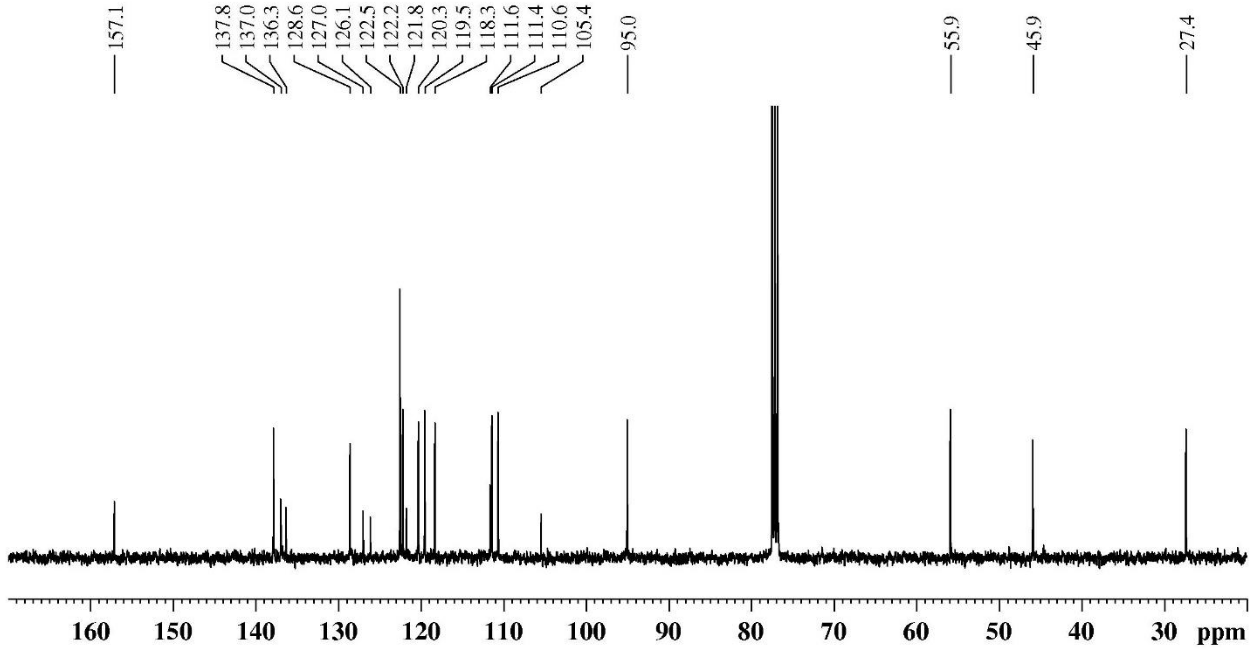

Figure S89. 4-Fluoro-3-(1-pentyl-1*H*-imidazol-5-yl)-1*H*-indole (**89**)

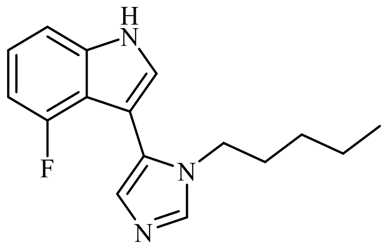

^{1}H NMR ($CDCl_3$, 400 MHz):

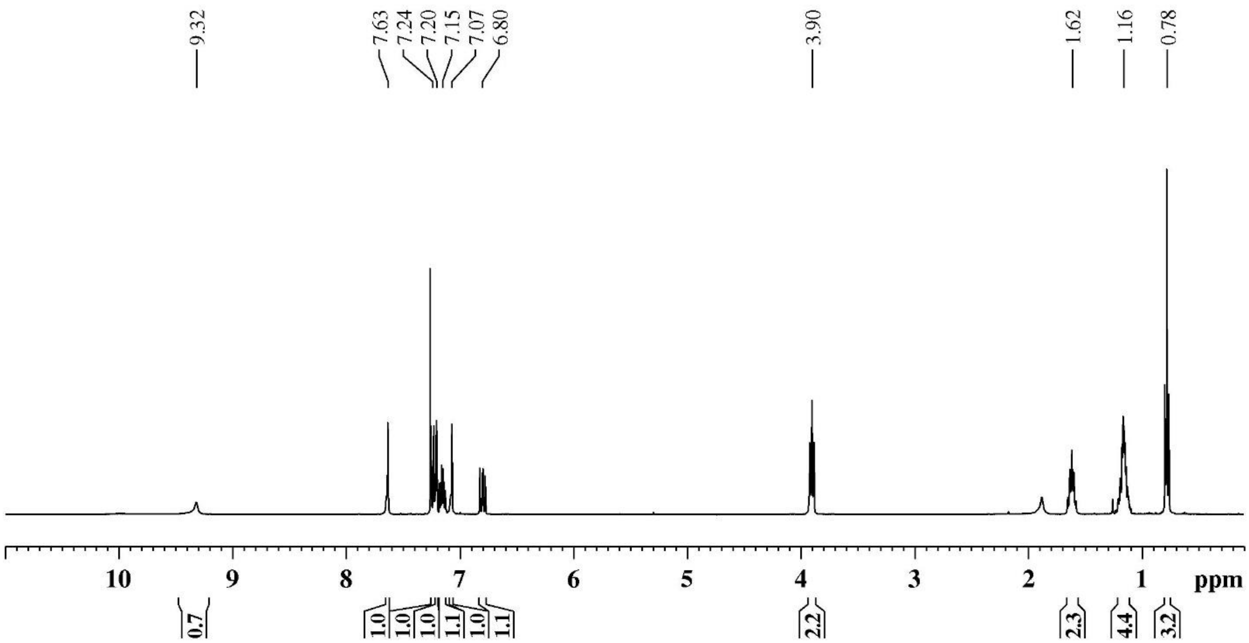

^{13}C NMR ($CDCl_3$, 100 MHz):

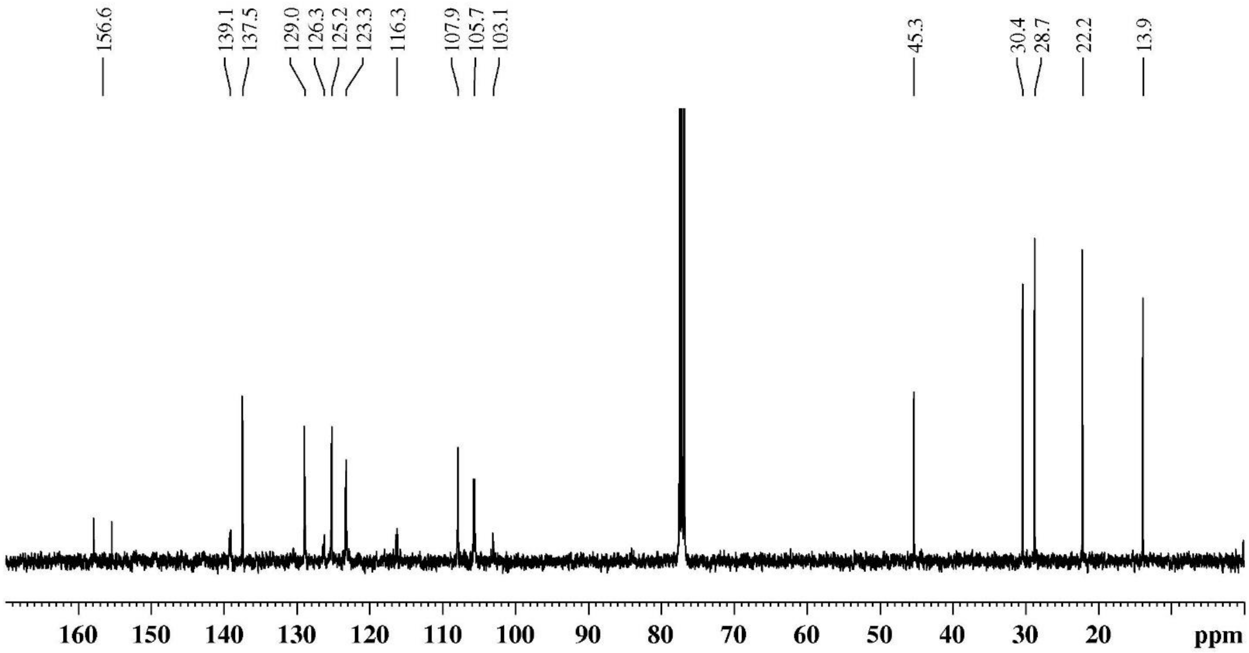

Figure S90. 7-Fluoro-3-(1-pentyl-1*H*-imidazol-5-yl)-1*H*-indole (**90**)

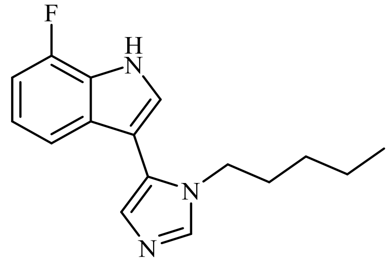

^{1}H NMR ($CDCl_3$, 400 MHz):

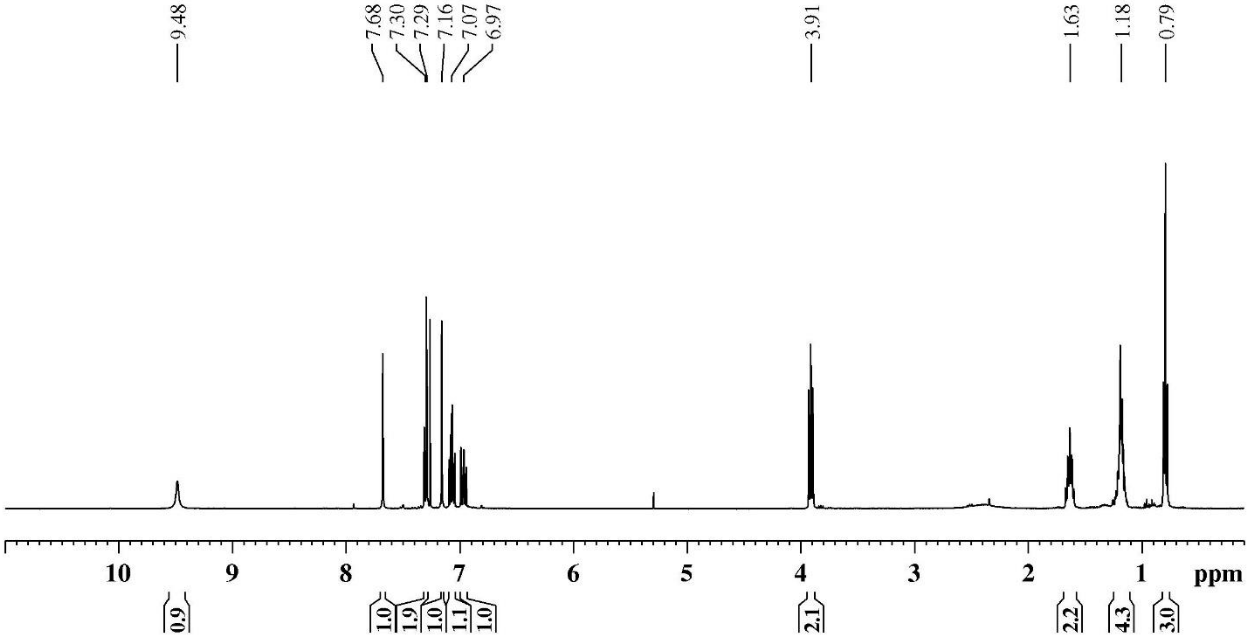

^{13}C NMR ($CDCl_3$, 100 MHz):

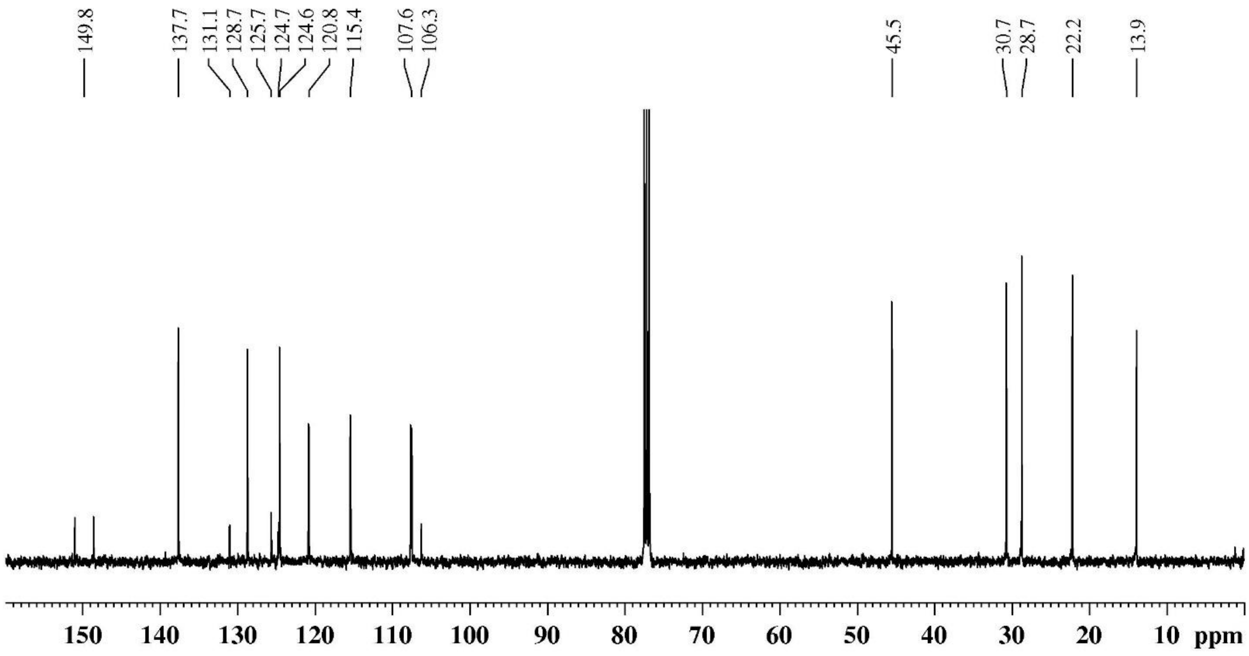

Figure S91. 4-Chloro-3-(1-pentyl-1*H*-imidazol-5-yl)-1*H*-indole (**91**)

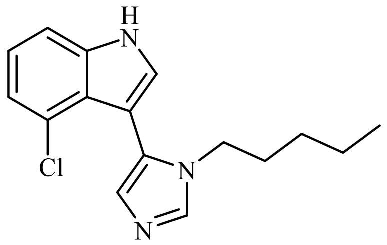

^{1}H NMR ($CDCl_3$, 400 MHz):

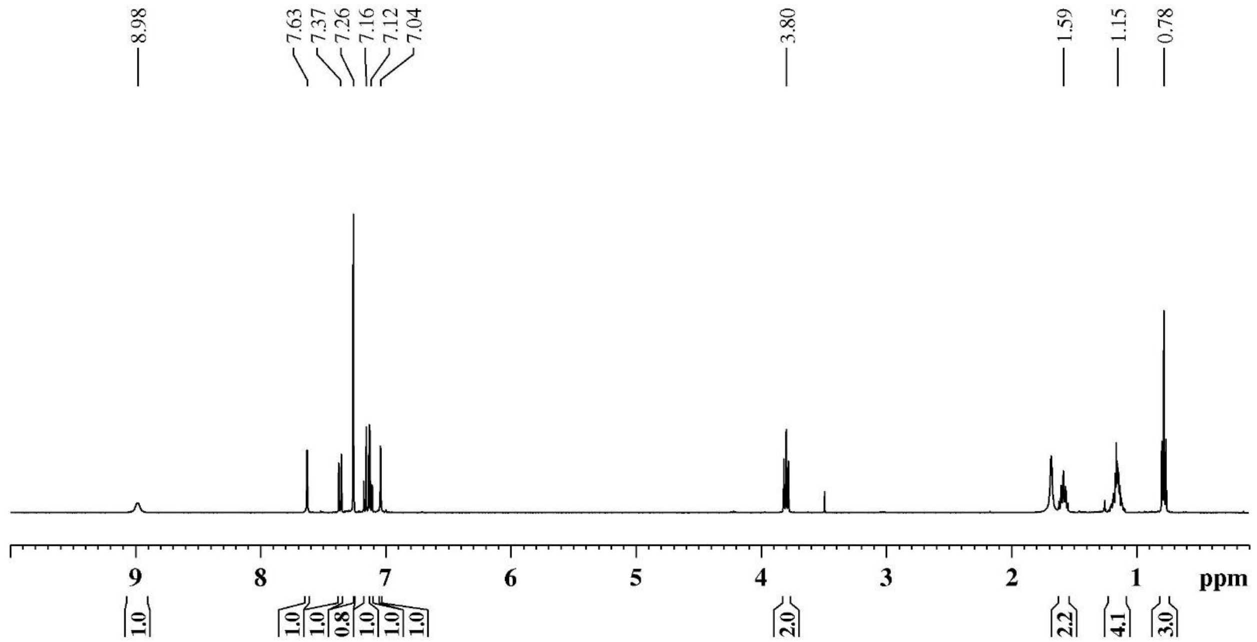

^{13}C NMR ($CDCl_3$, 100 MHz):

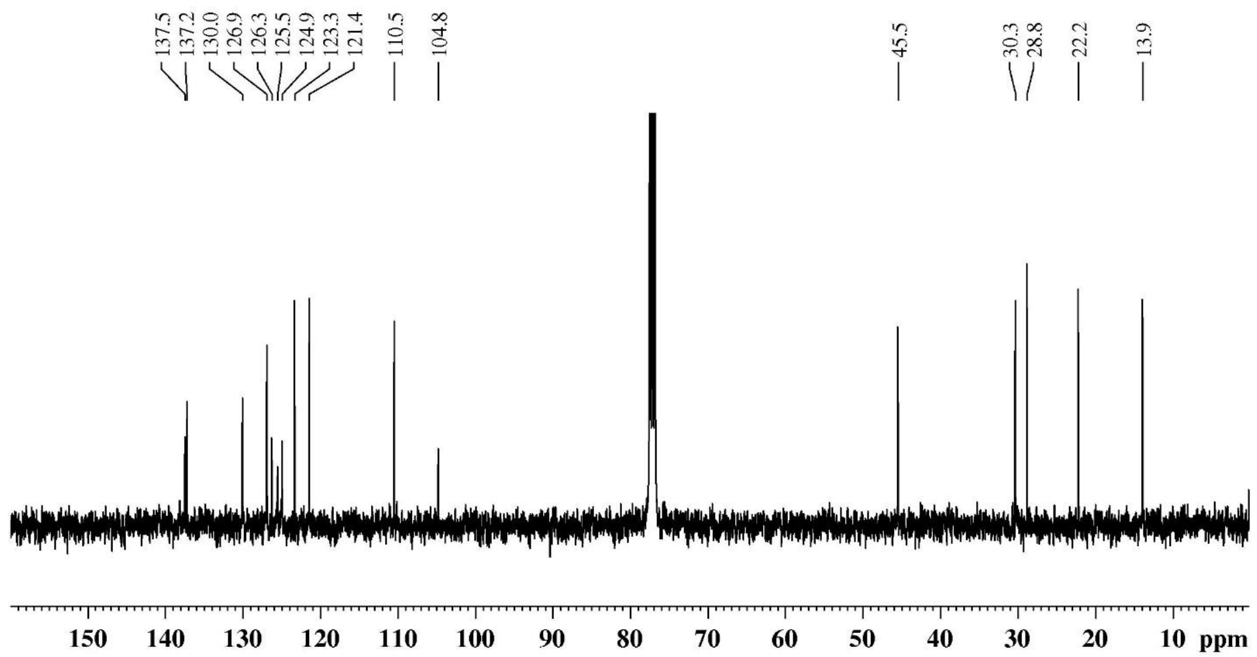

Figure S92. 5-Chloro-3-(1-pentyl-1*H*-imidazol-5-yl)-1*H*-indole (**92**)

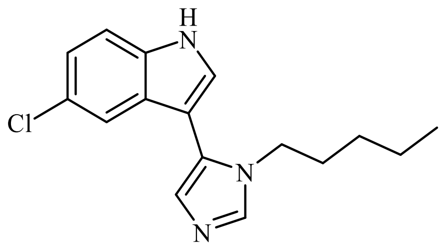

^{1}H NMR ($CDCl_3$, 400 MHz):

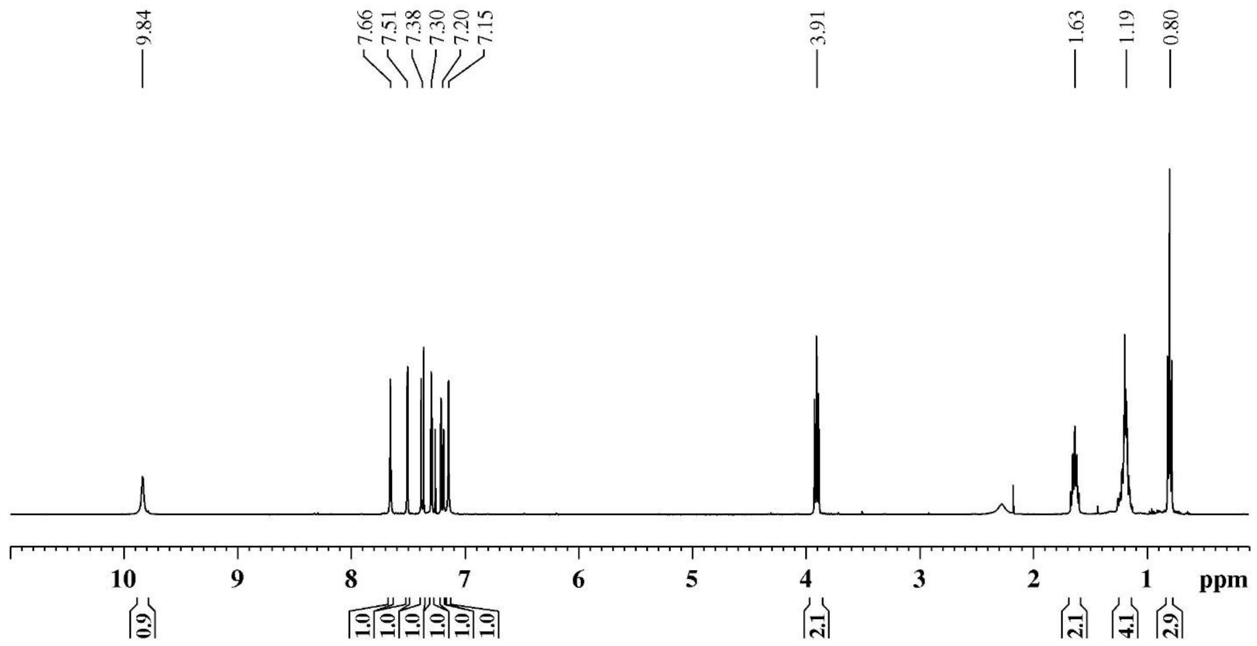

^{13}C NMR ($CDCl_3$, 100 MHz):

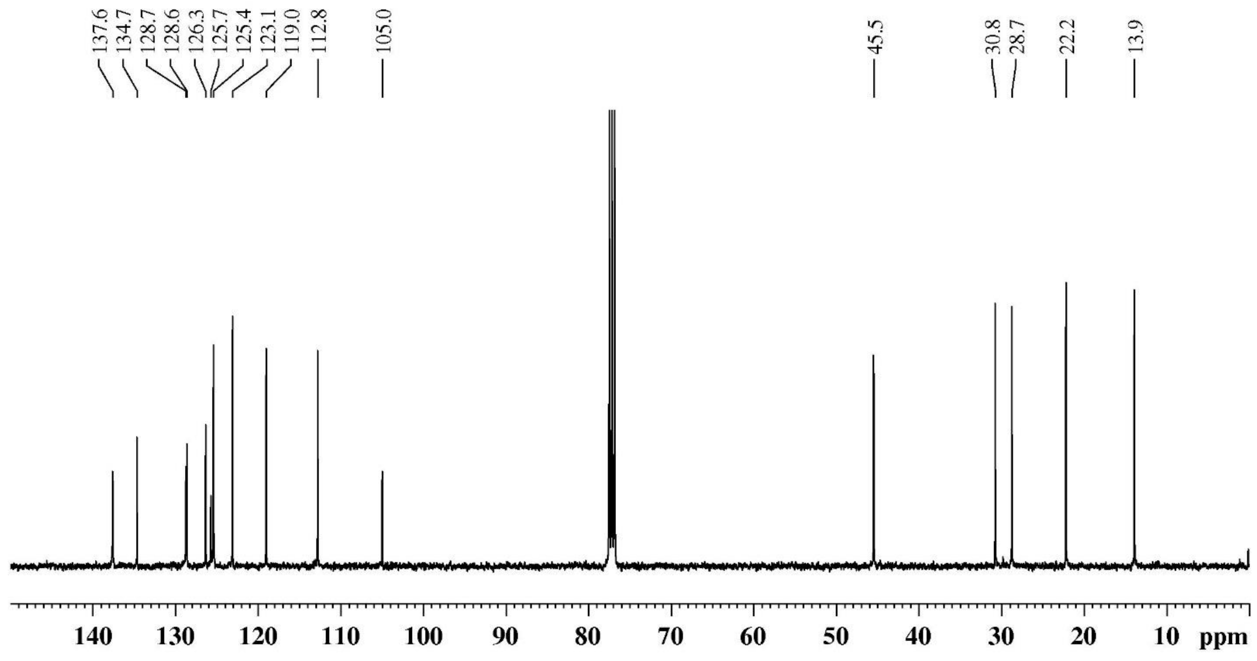

Figure S93. 7-Chloro-3-(1-pentyl-1*H*-imidazol-5-yl)-1*H*-indole (**93**)

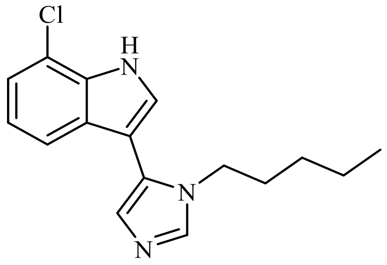

^{1}H NMR ($CDCl_3$, 400 MHz):

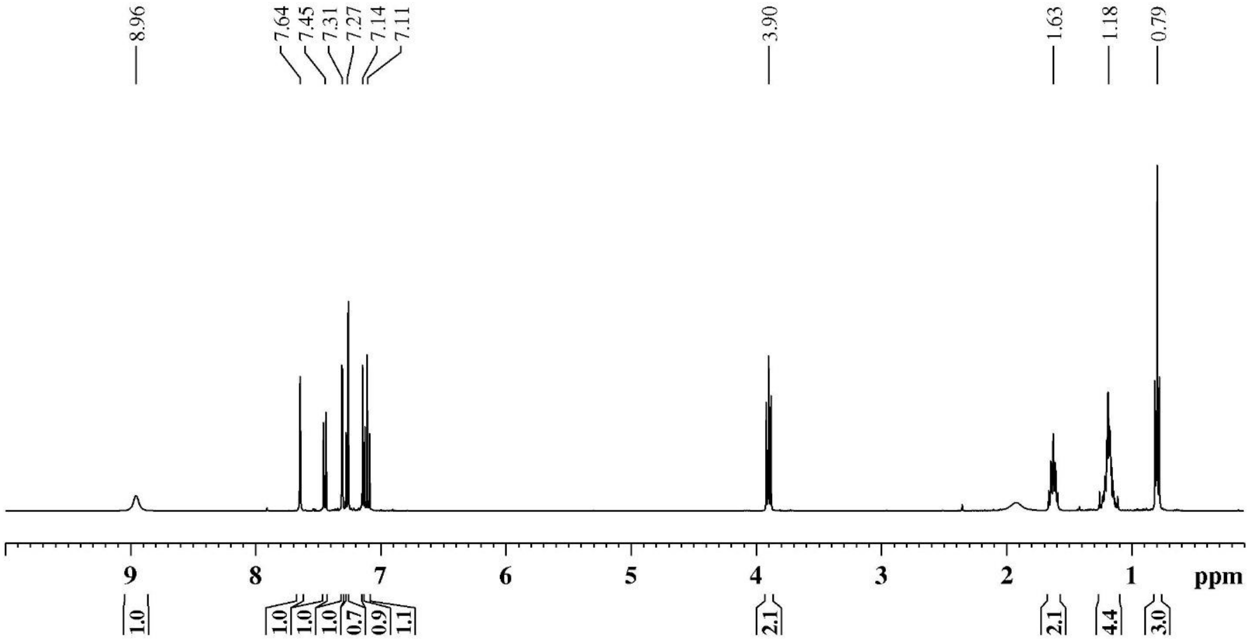

^{13}C NMR ($CDCl_3$, 100 MHz):

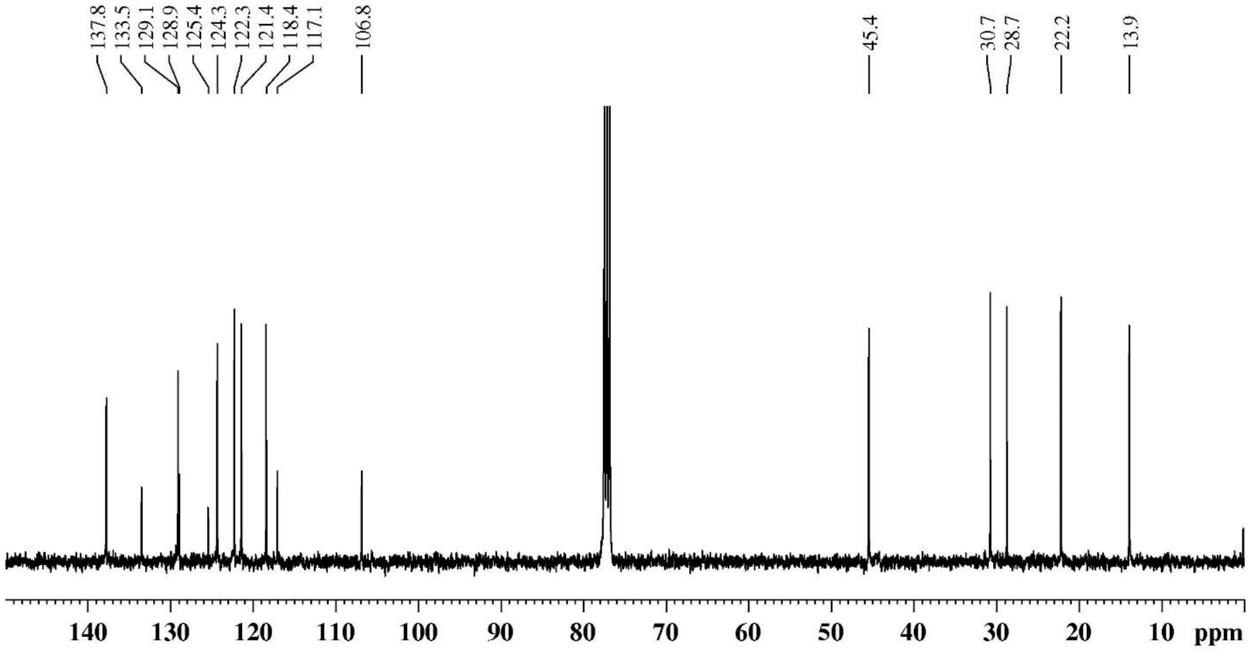

Figure S94. 4-Bromo-3-(1-pentyl-1*H*-imidazol-5-yl)-1*H*-indole (**94**)

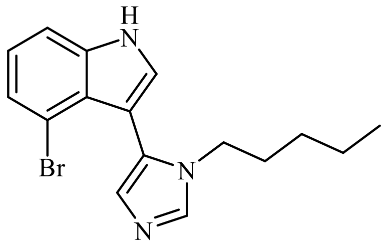

^{1}H NMR ($CDCl_3$, 400 MHz):

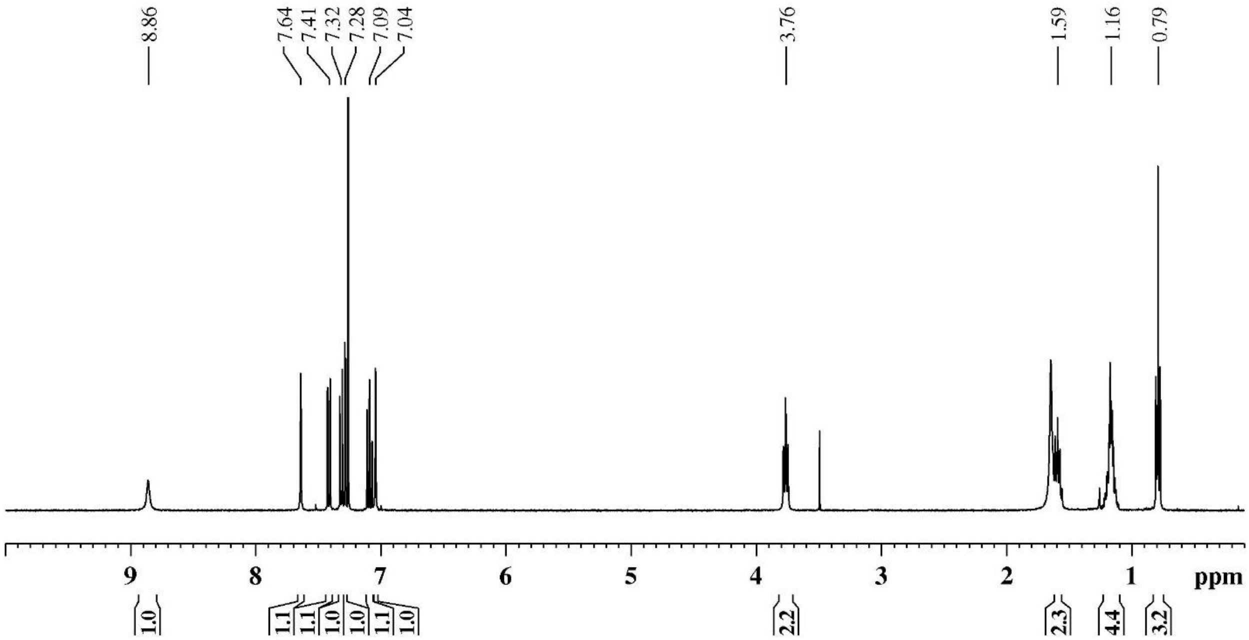

^{13}C NMR ($CDCl_3$, 100 MHz):

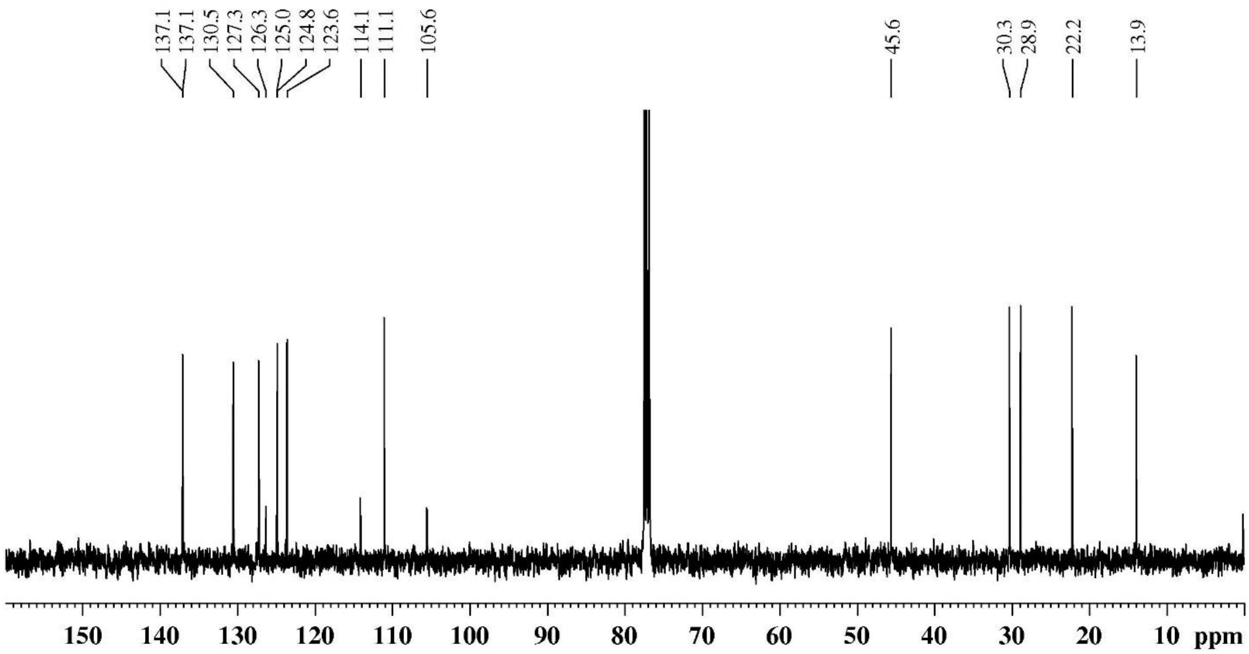

Figure S95. 5-Methoxy-3-(1-pentyl-1*H*-imidazol-5-yl)-1*H*-indole (**95**)

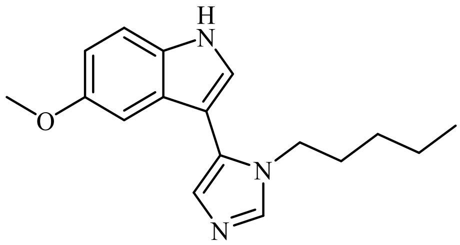

^{1}H NMR ($CDCl_3$, 400 MHz):

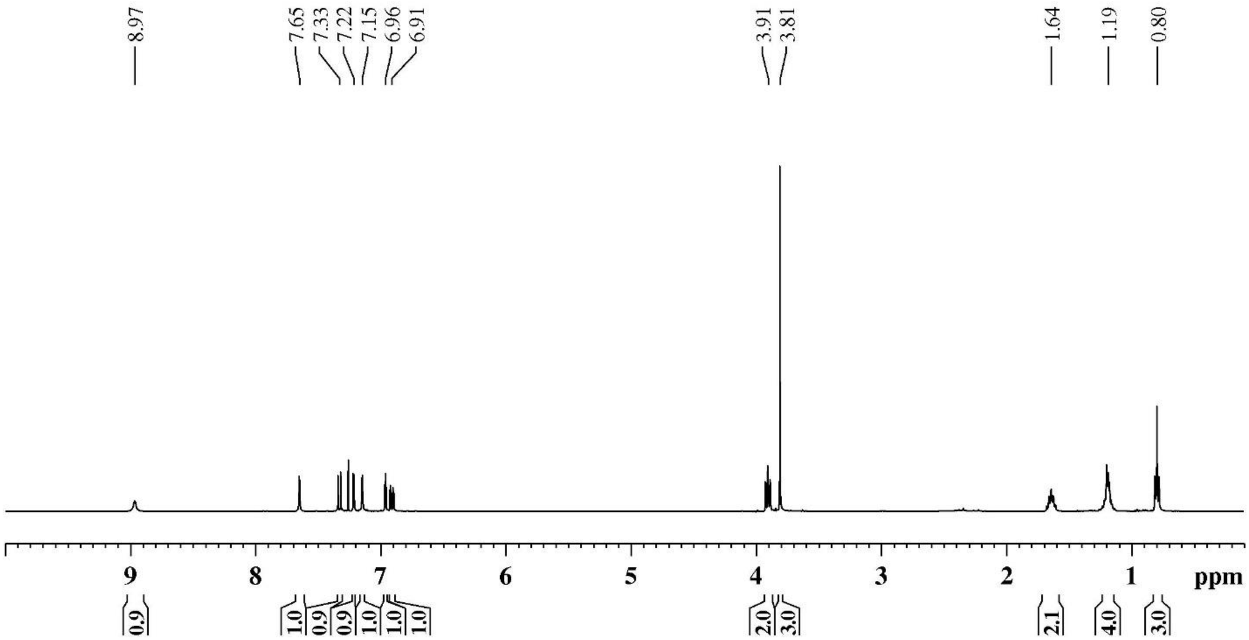

^{13}C NMR ($CDCl_3$, 100 MHz):

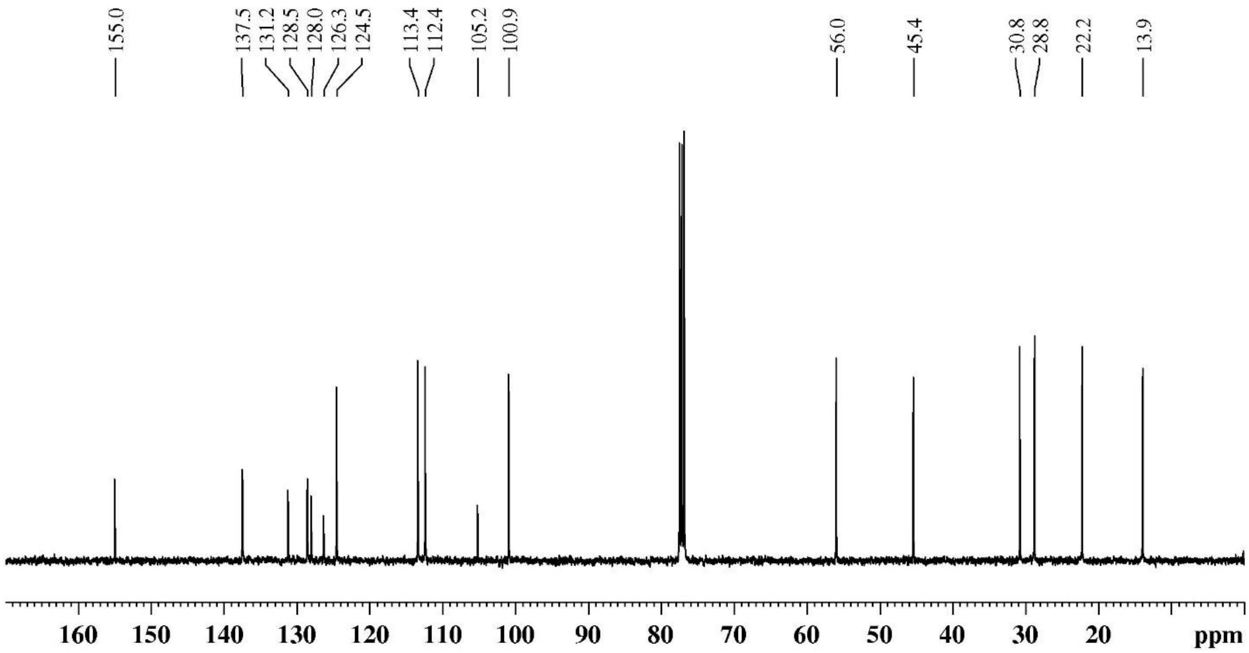

Figure S96. 6-Methoxy-3-(1-pentyl-1*H*-imidazol-5-yl)-1*H*-indole (**96**)

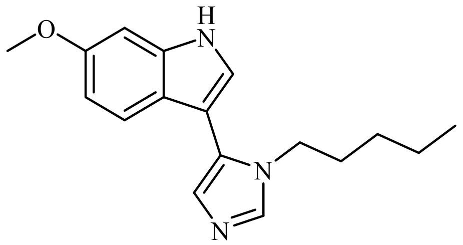

^{1}H NMR ($CDCl_3$, 400 MHz):

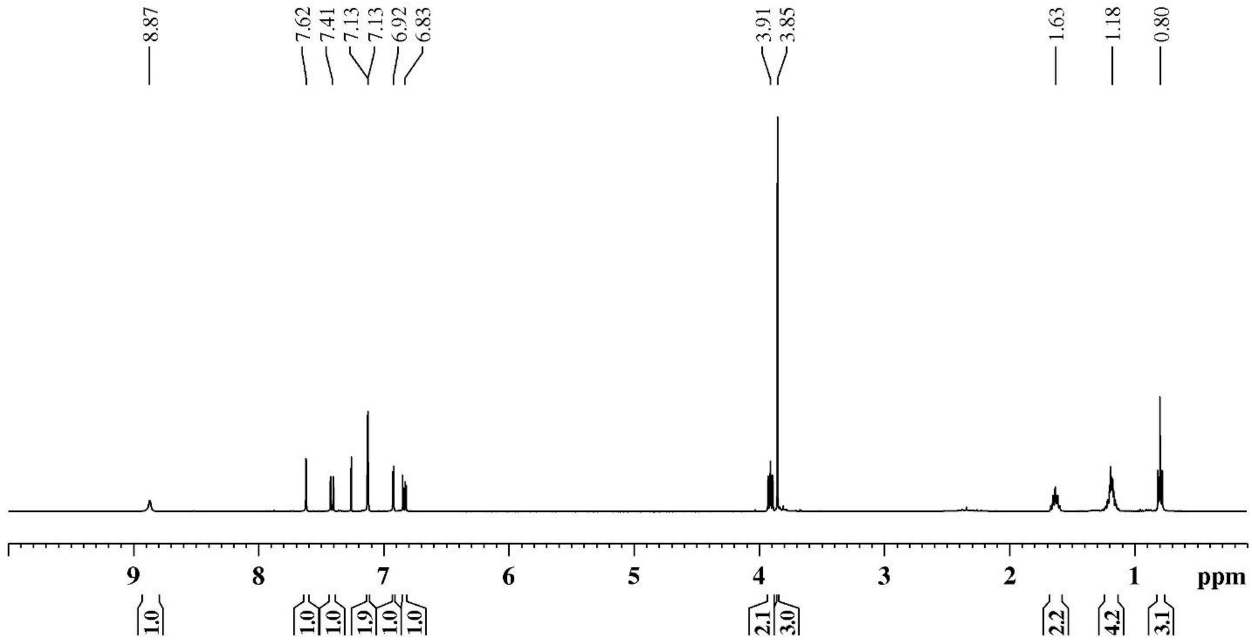

^{13}C NMR ($CDCl_3$, 100 MHz):

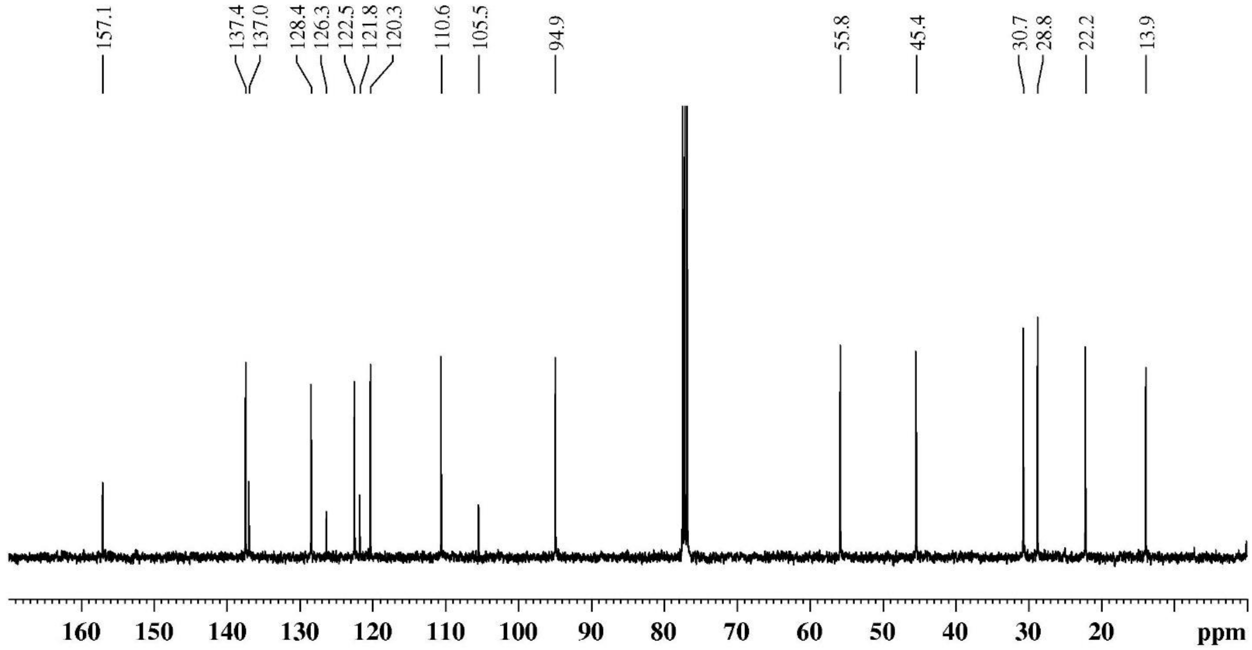

Figure S97. 3-(1-(Benzo[*d*][1,3]dioxol-5-ylmethyl)-1*H*-imidazol-5-yl)-5-chloro-1*H*-indole (**97**)

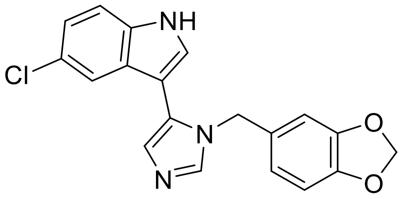

^{1}H NMR (DMSO-d_6, 400 MHz):

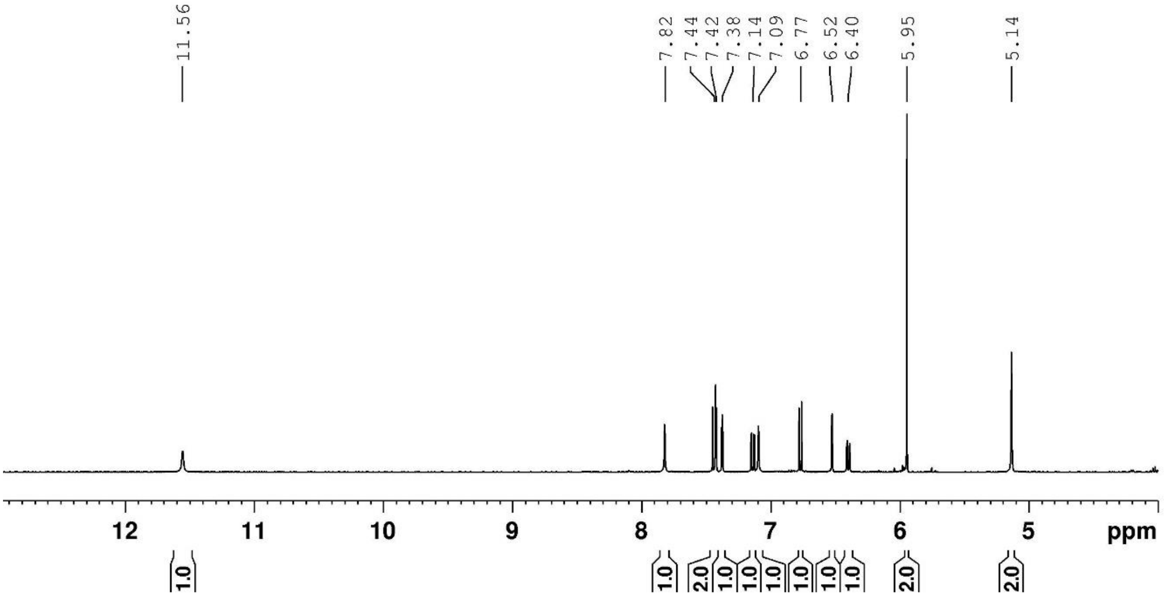

^{13}C NMR (DMSO-d_6, 100 MHz):

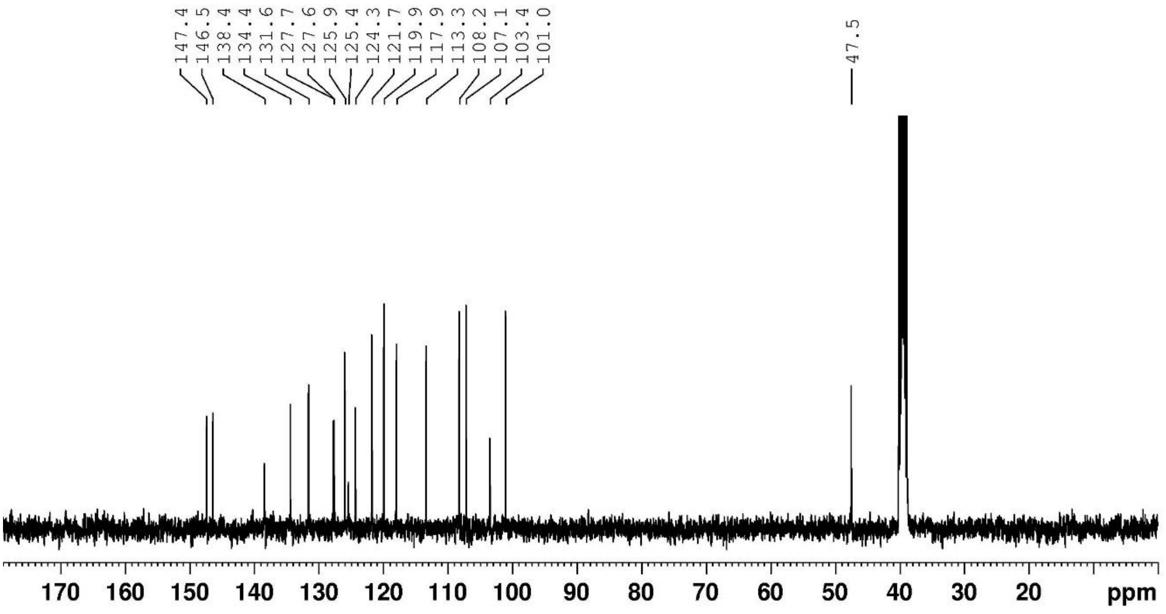

Figure S98. 5-Chloro-3-(1-(2-(pyrrolidin-1-yl)ethyl)-1*H*-imidazol-5-yl)-1*H*-indole (**98**)

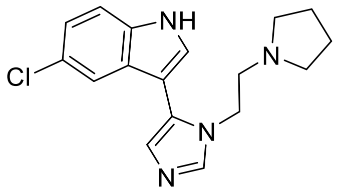

^{1}H NMR (DMSO-*d*$_6$, 400 MHz):

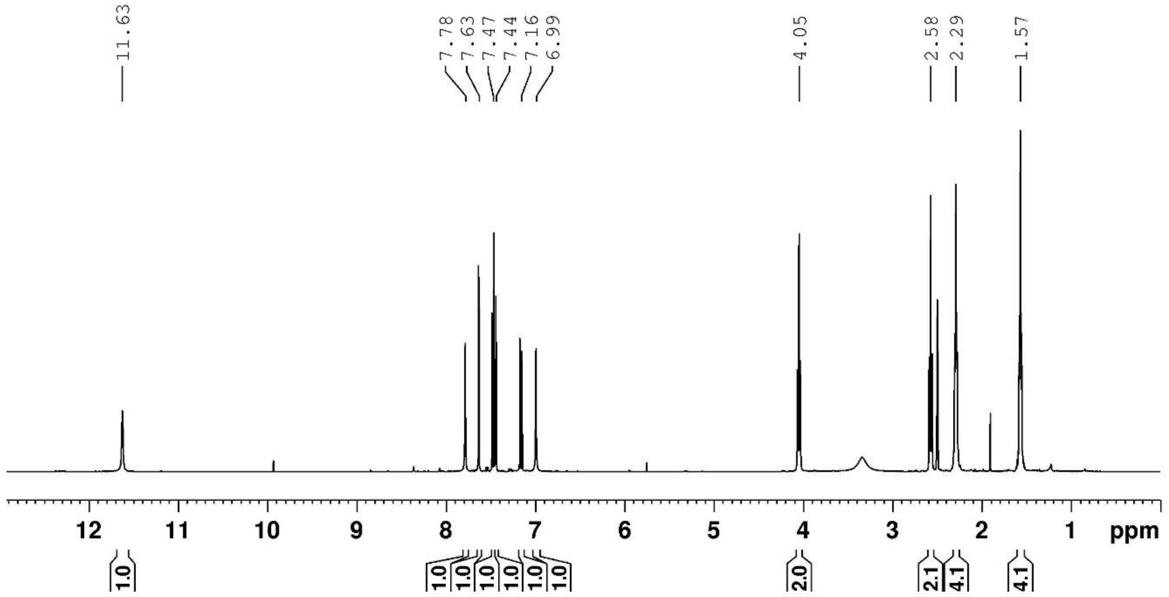

^{13}C NMR (DMSO-*d*$_6$, 100 MHz):

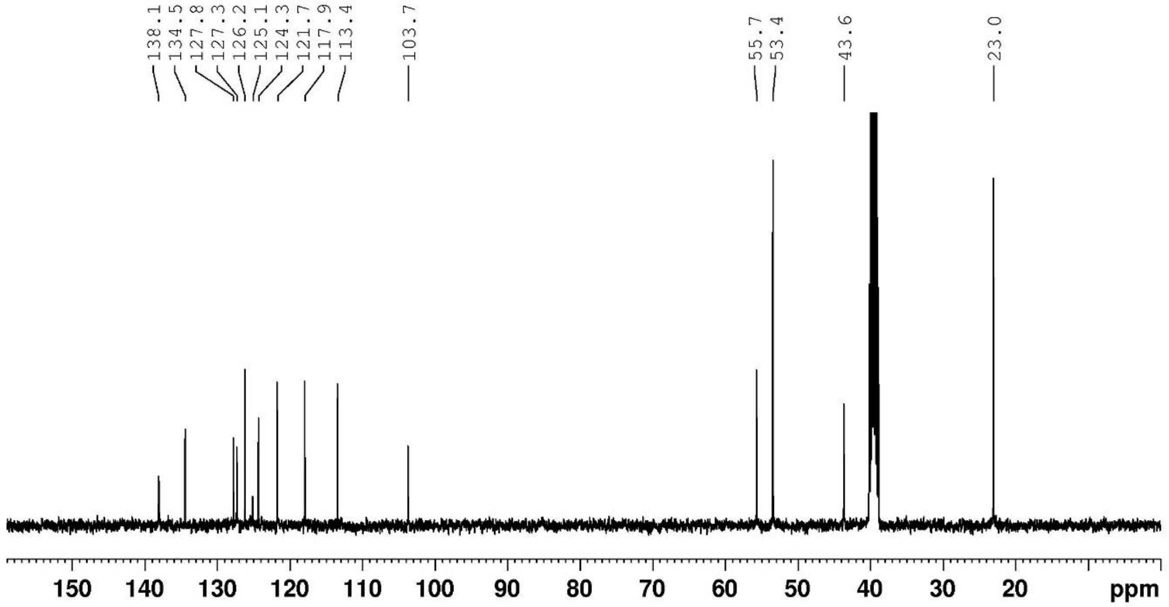

Figure S99. (*E*)-5-Chloro-3-(1-(3,7-dimethylocta-2,6-dien-1-yl)-1*H*-imidazol-5-yl)-1*H*-indole (**99**)

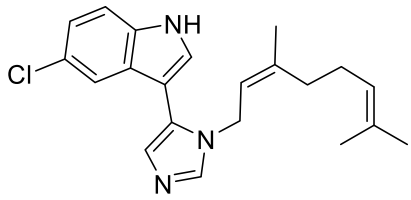

^{1}H NMR (DMSO-d_6, 400 MHz):

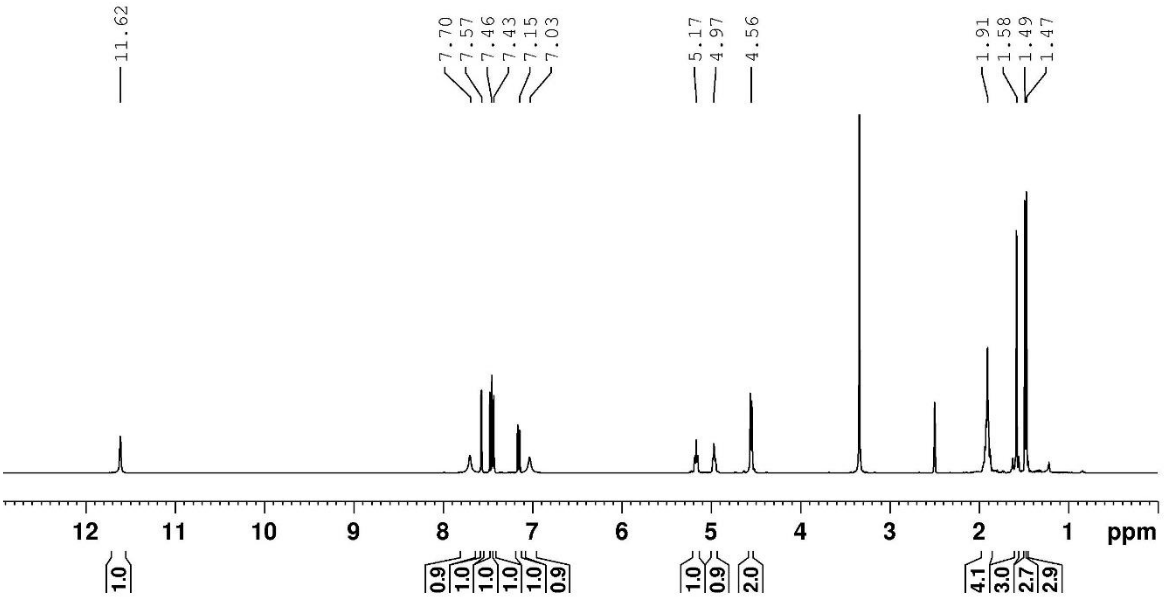

^{13}C NMR (DMSO-d_6, 100 MHz):

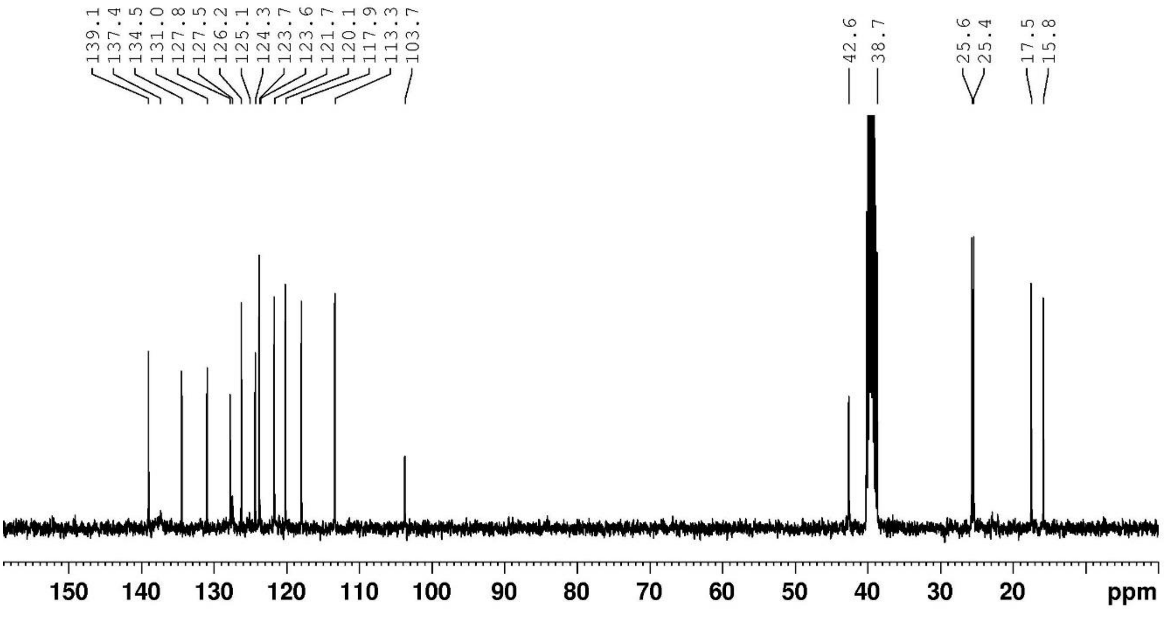

Figure S100. 6-(5-(5-Chloro-1*H*-indol-3-yl)-1*H*-imidazol-1-yl)hexan-1-ol (**100**)

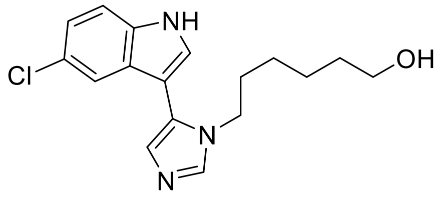

^{1}H NMR (DMSO-*d*$_6$, 400 MHz):

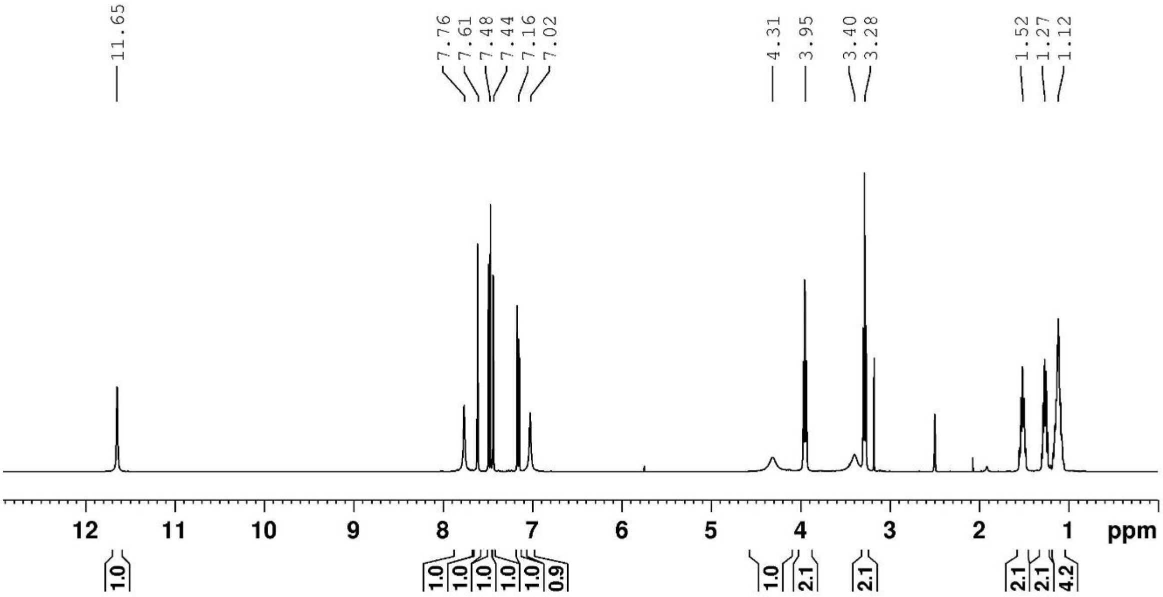

^{13}C NMR (DMSO-*d*$_6$, 100 MHz):

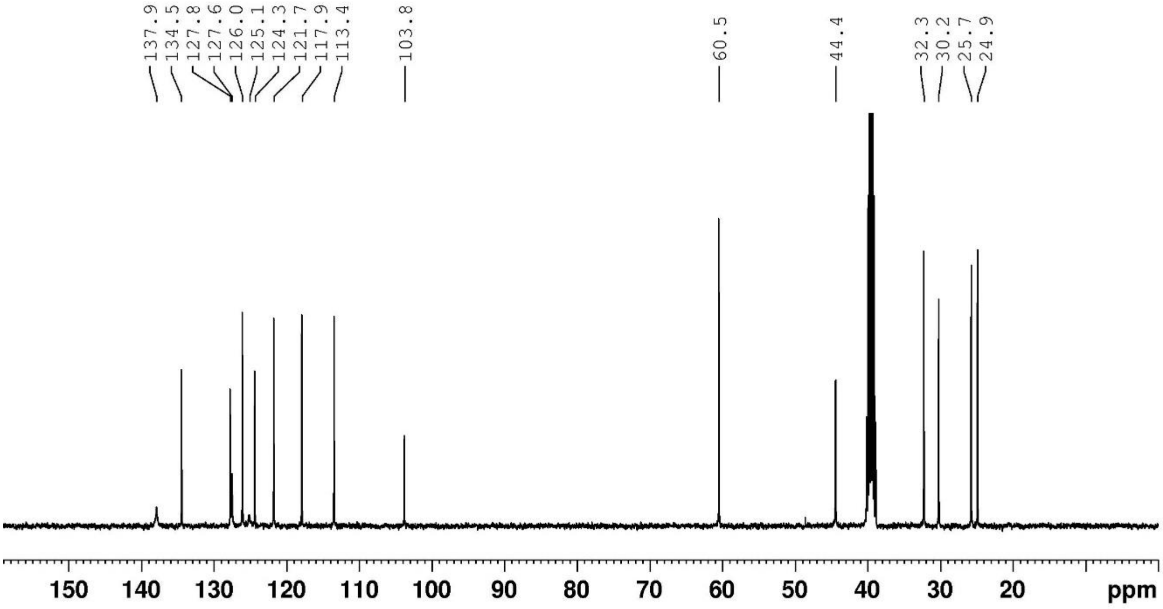

Figure S101. *tert*-Butyl (6-(5-(5-chloro-1*H*-indol-3-yl)-1*H*-imidazol-1-yl)hexyl)carbamate (**101**)

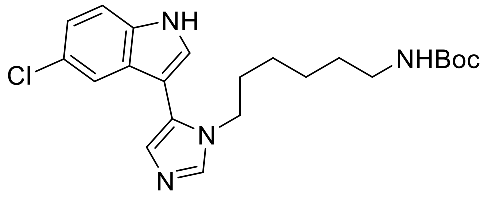

^{1}H NMR (DMSO-d_6, 400 MHz):

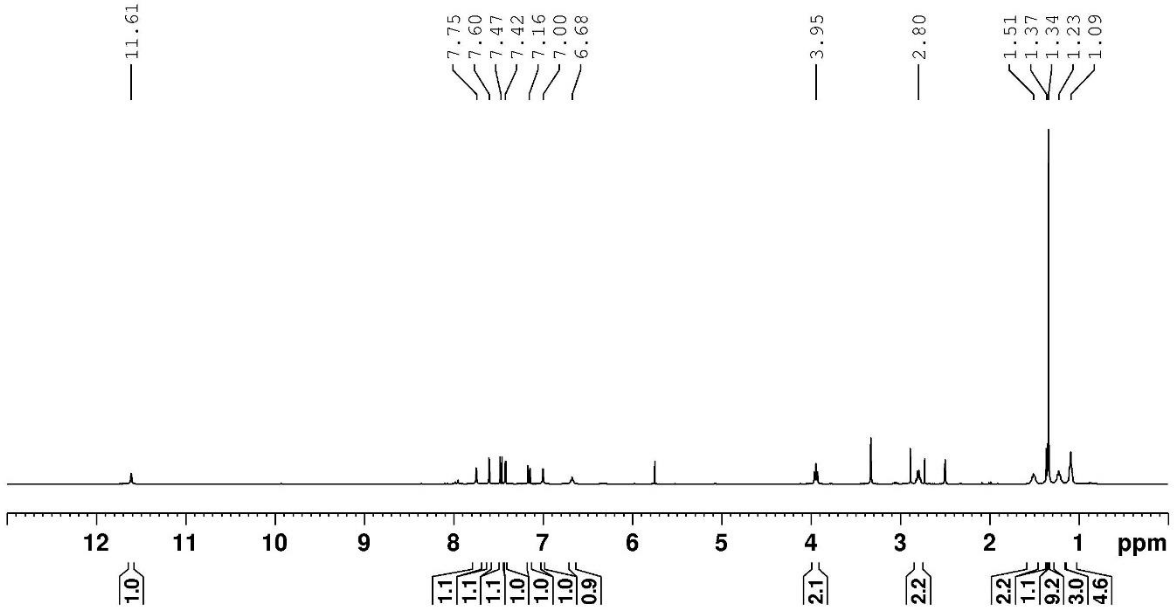

^{13}C NMR (DMSO-d_6, 100 MHz):

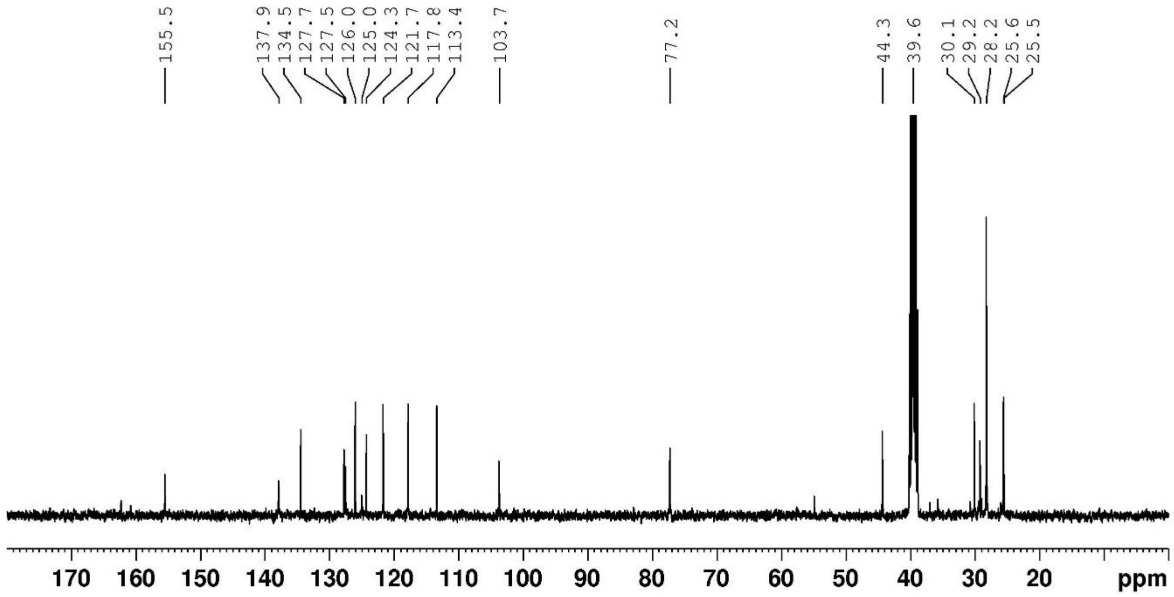

Figure S102. 6-(5-(5-Chloro-1*H*-indol-3-yl)-1*H*-imidazol-1-yl)hexan-1-aminium 2,2,2-trifluoroacetate (**102**)

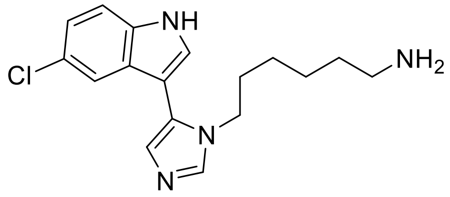

^{1}H NMR (DMSO-d_6, 400 MHz):

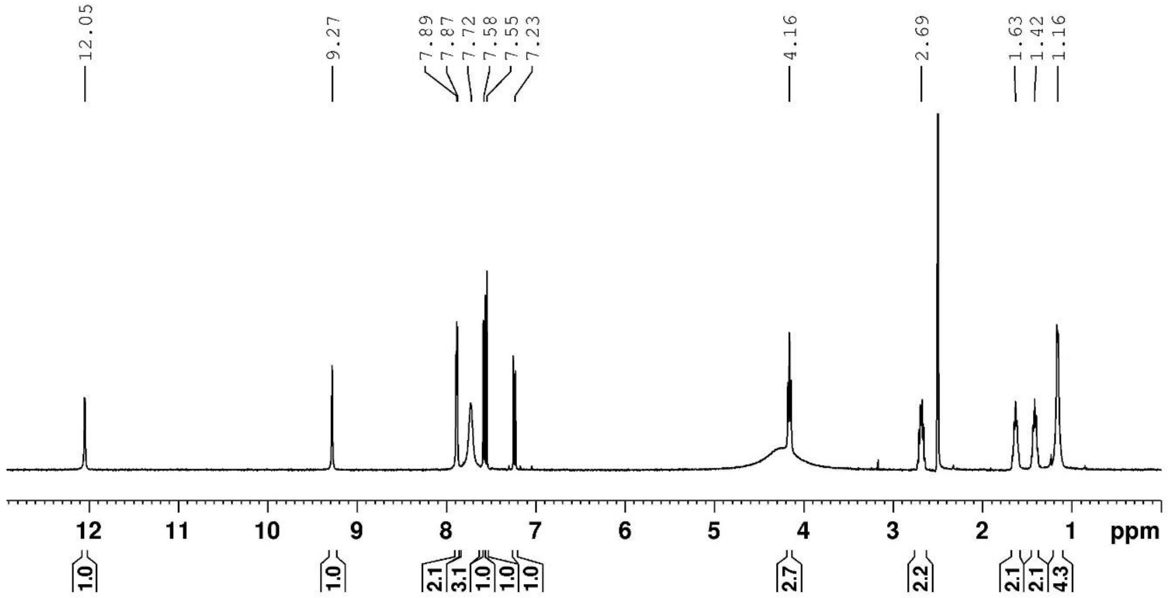

^{13}C NMR (DMSO-d_6, 100 MHz):

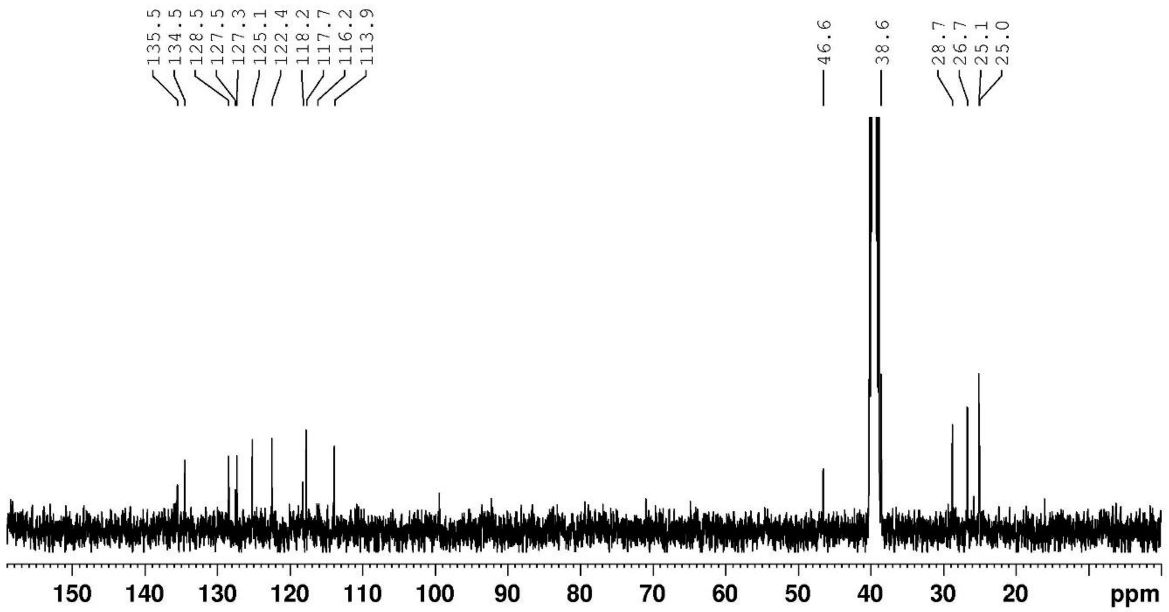

Figure S103. 1-Benzyl-5-phenyl-1*H*-imidazole (**103**)

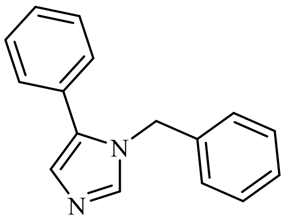

^{1}H NMR ($CDCl_3$, 400 MHz):

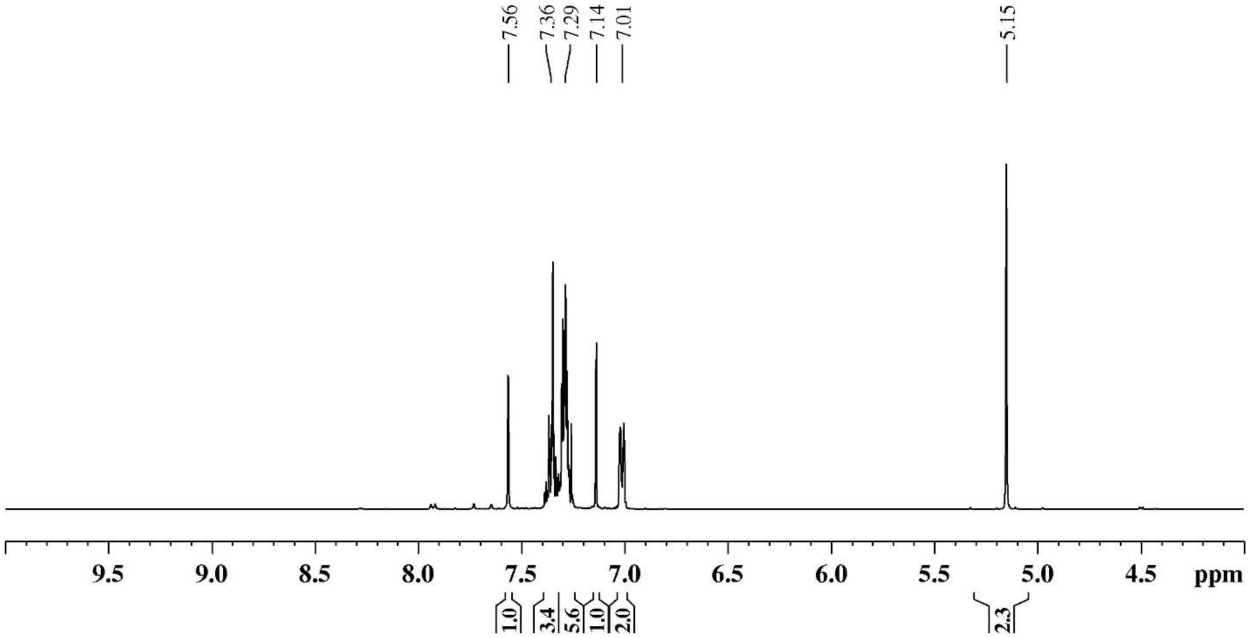

^{13}C NMR ($CDCl_3$, 100 MHz):

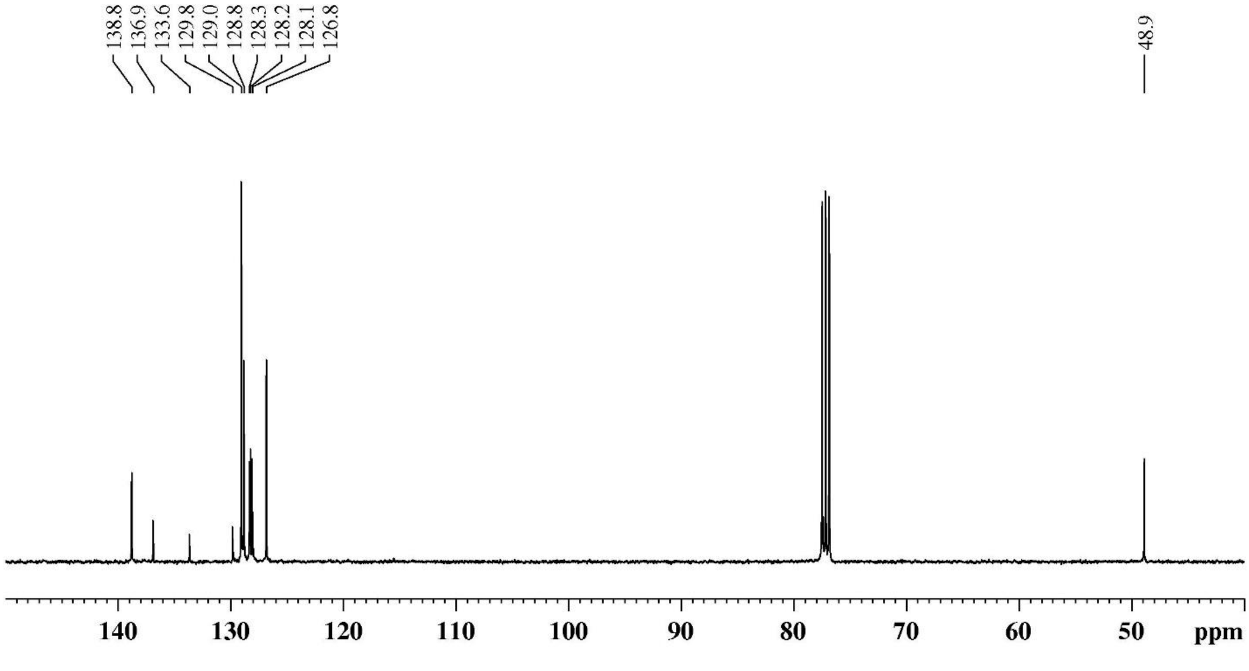

Figure S104. 1-Benzyl-5-(4-methoxyphenyl)-1*H*-imidazole (**104**)

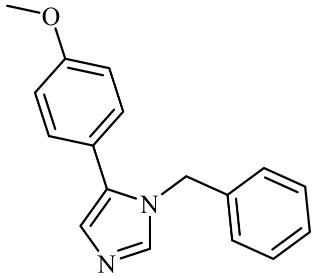

^{1}H NMR ($CDCl_3$, 400 MHz):

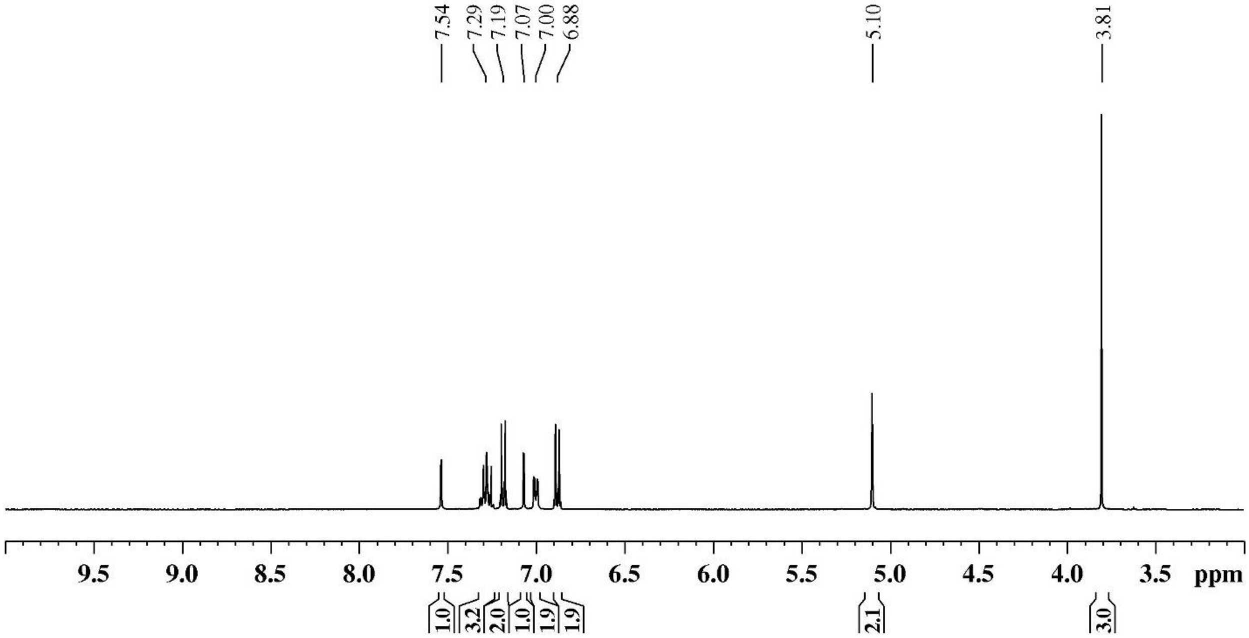

^{13}C NMR ($CDCl_3$, 100 MHz):

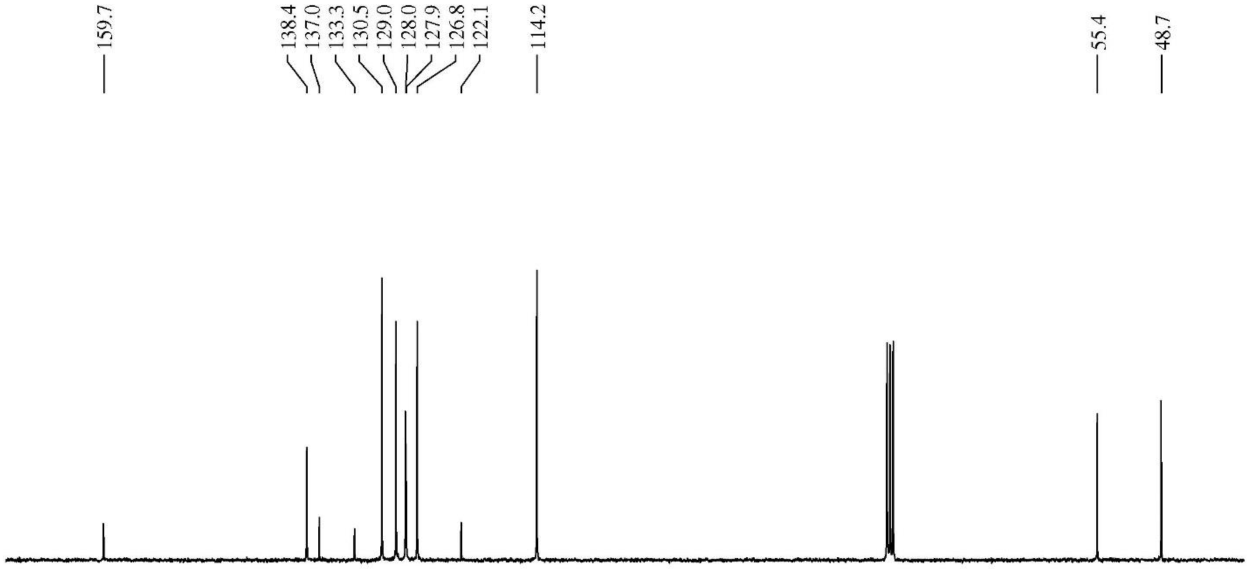

Figure S105. 1-Phenethyl-5-phenyl-1*H*-imidazole (**105**)

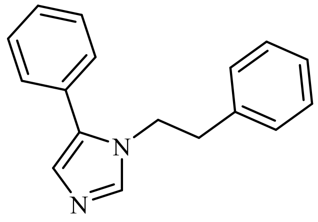

^{1}H NMR ($CDCl_3$, 400 MHz):

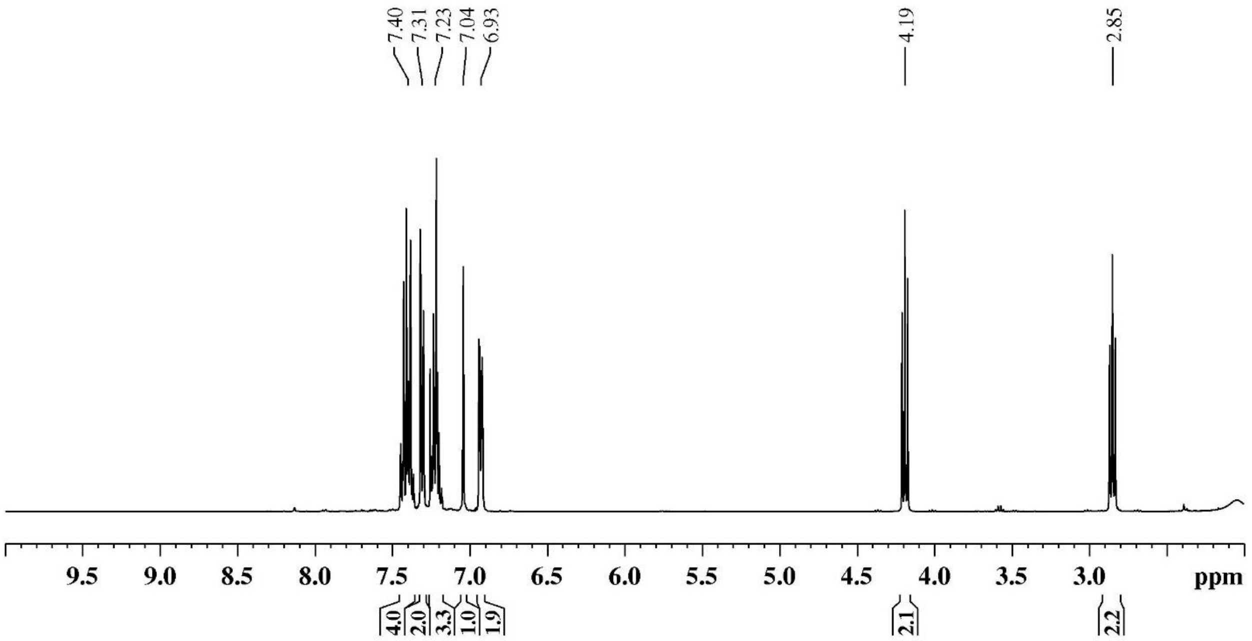

^{13}C NMR ($CDCl_3$, 100 MHz):

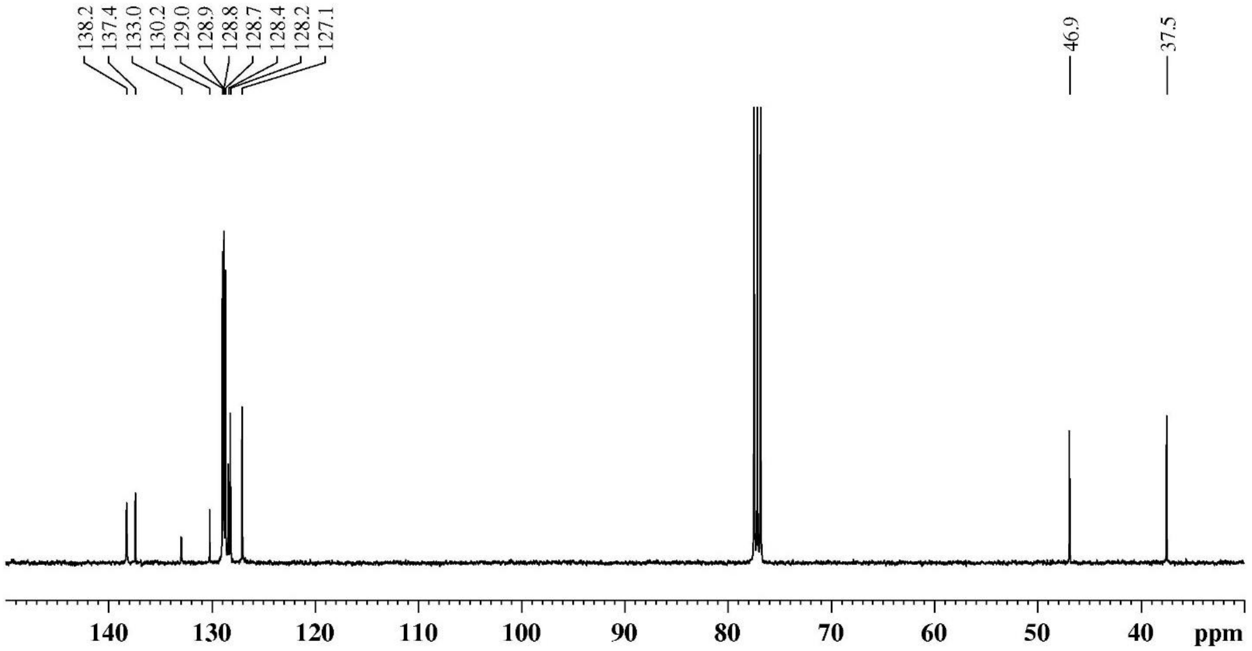

Figure S106. 5-(4-Methoxyphenyl)-1-phenethyl-1*H*-imidazole (**106**)

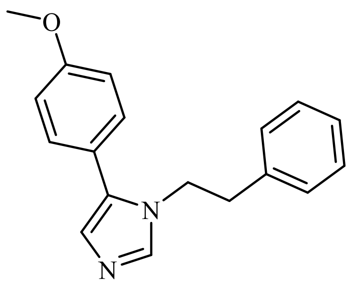

^{1}H NMR ($CDCl_3$, 400 MHz):

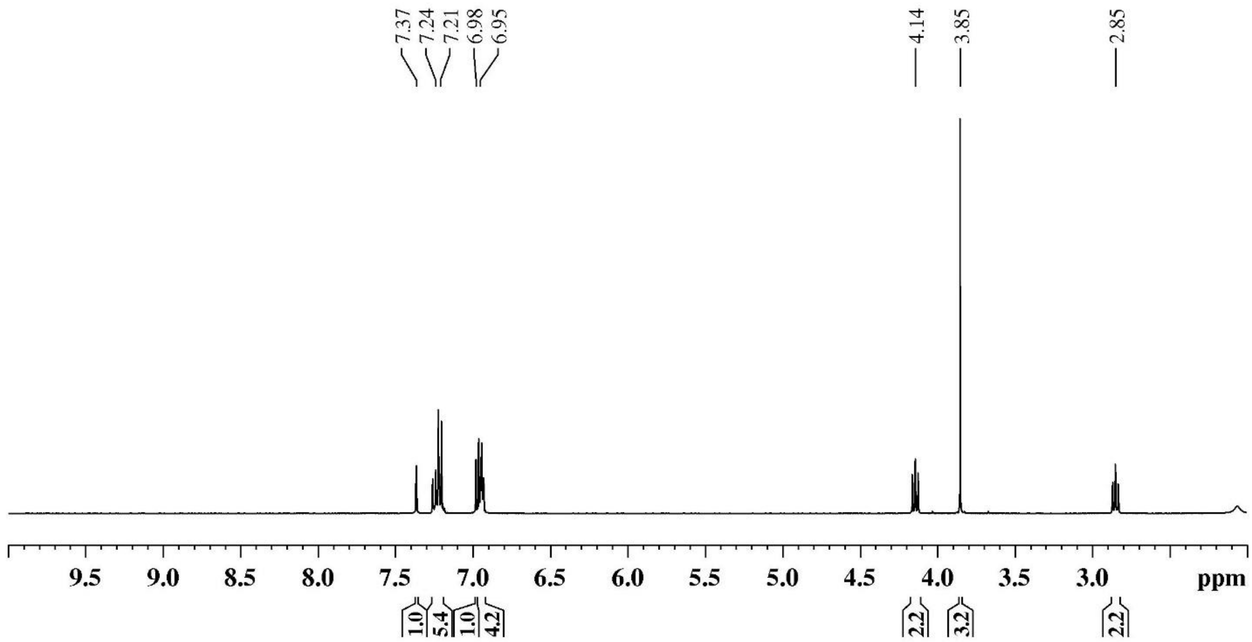

^{13}C NMR ($CDCl_3$, 100 MHz):

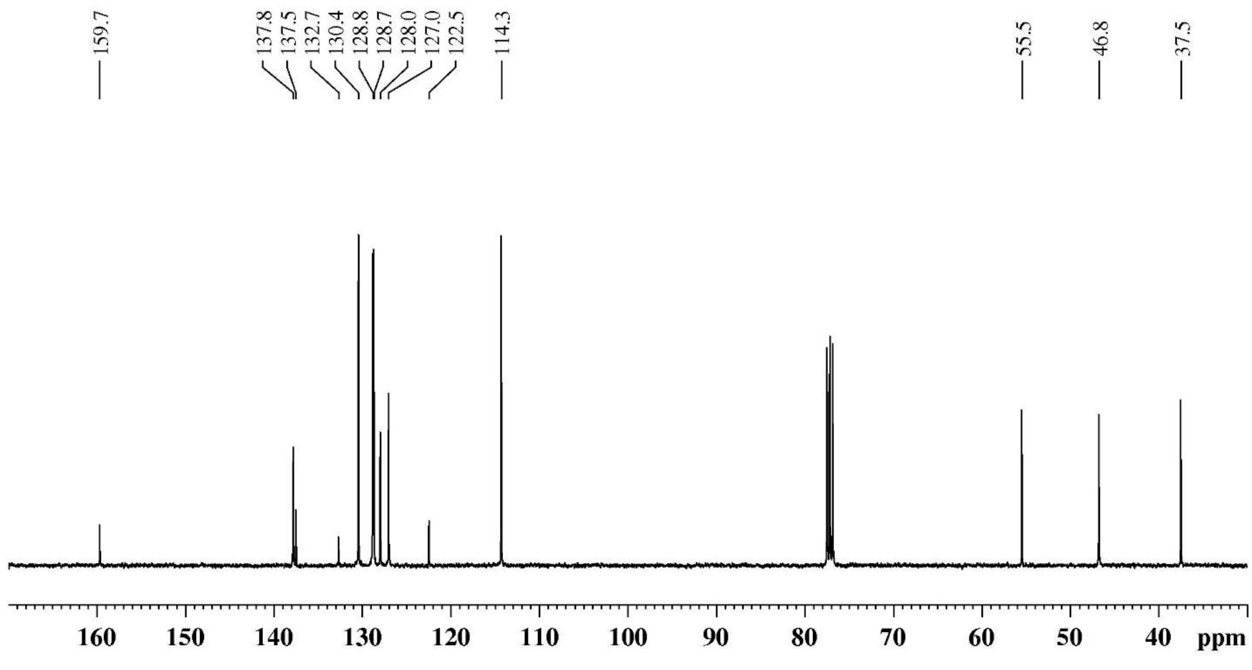

Figure S107. 1-(4-Methoxybenzyl)-5-phenyl-1*H*-imidazole (**107**)

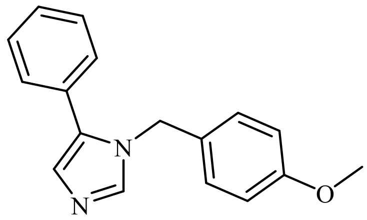

^{1}H NMR ($CDCl_3$, 400 MHz):

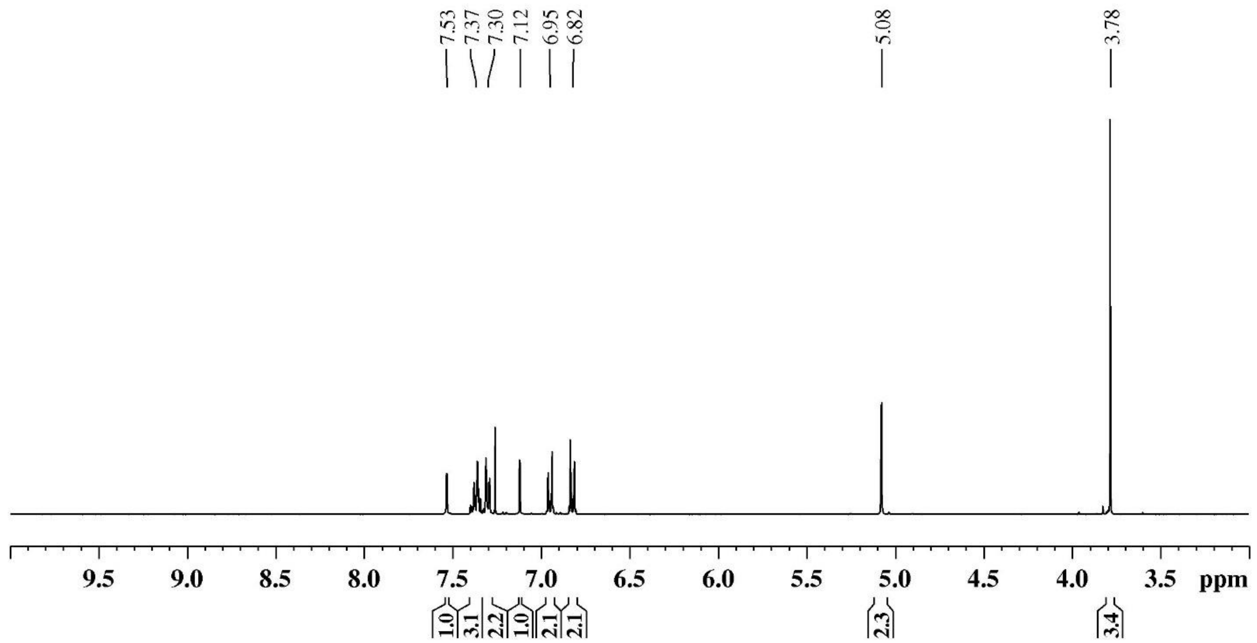

^{13}C NMR ($CDCl_3$, 100 MHz):

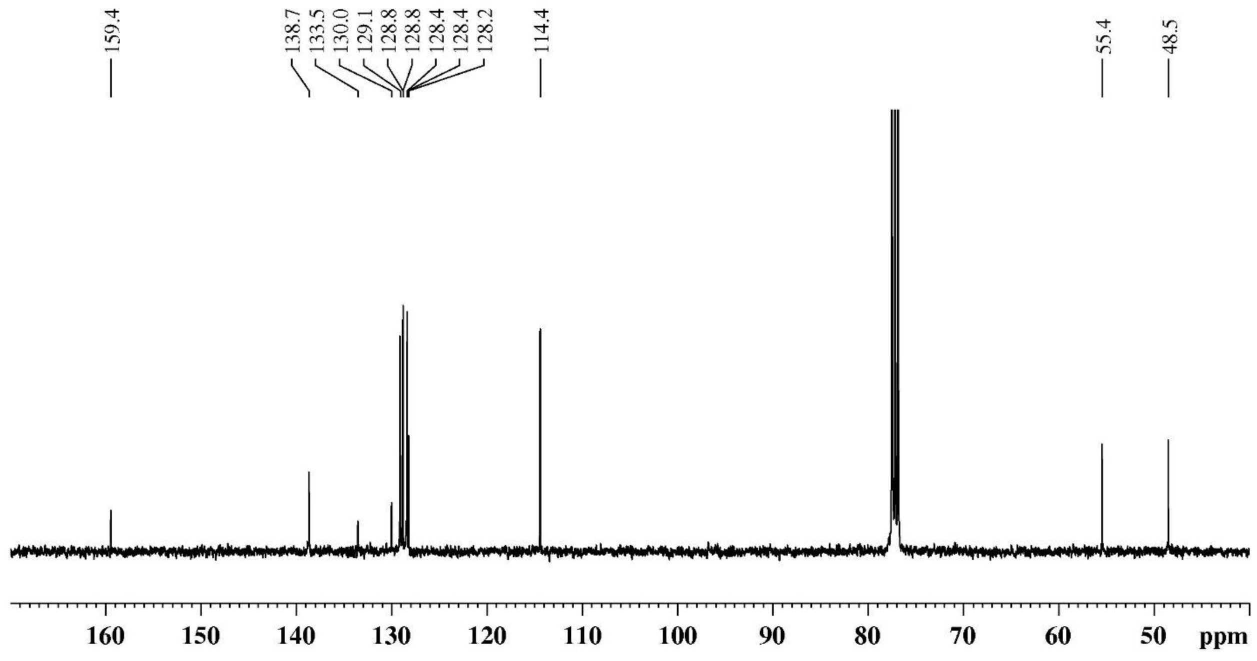

Figure S108. 1-(4-Methoxybenzyl)-5-(4-methoxyphenyl)-1*H*-imidazole (**108**)

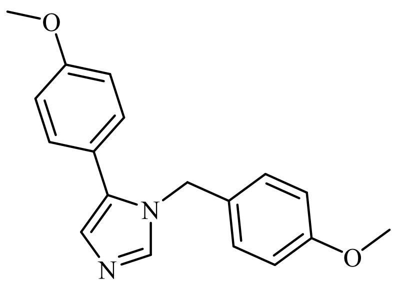

^{1}H NMR ($CDCl_3$, 400 MHz):

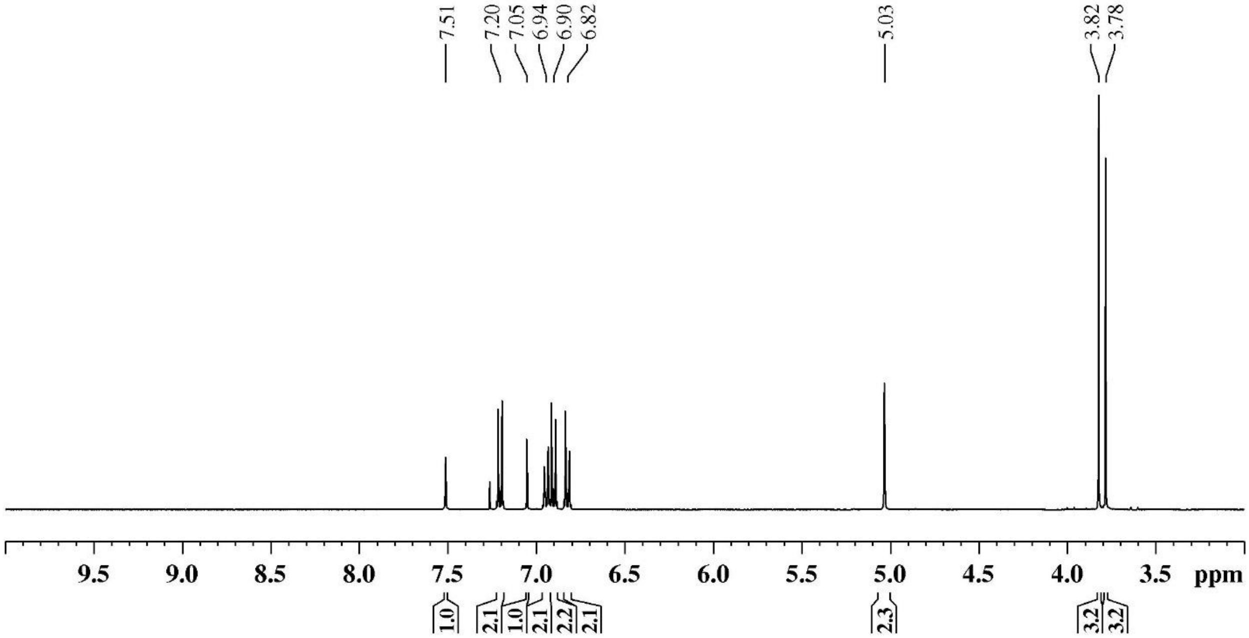

^{13}C NMR ($CDCl_3$, 100 MHz):

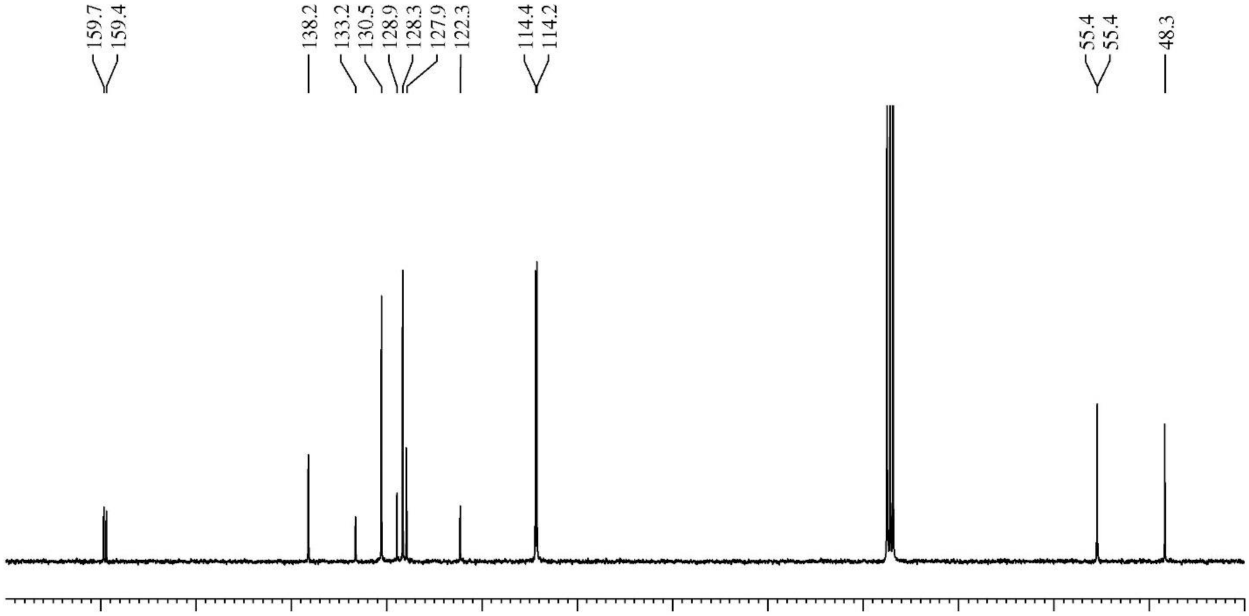

Figure S109. 1-(4-Methoxyphenethyl)-5-phenyl-1*H*-imidazole (**109**)

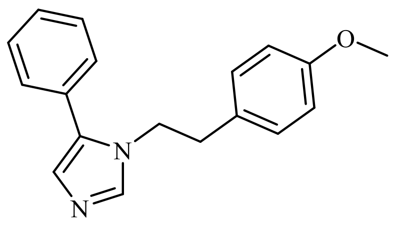

^{1}H NMR ($CDCl_3$, 400 MHz):

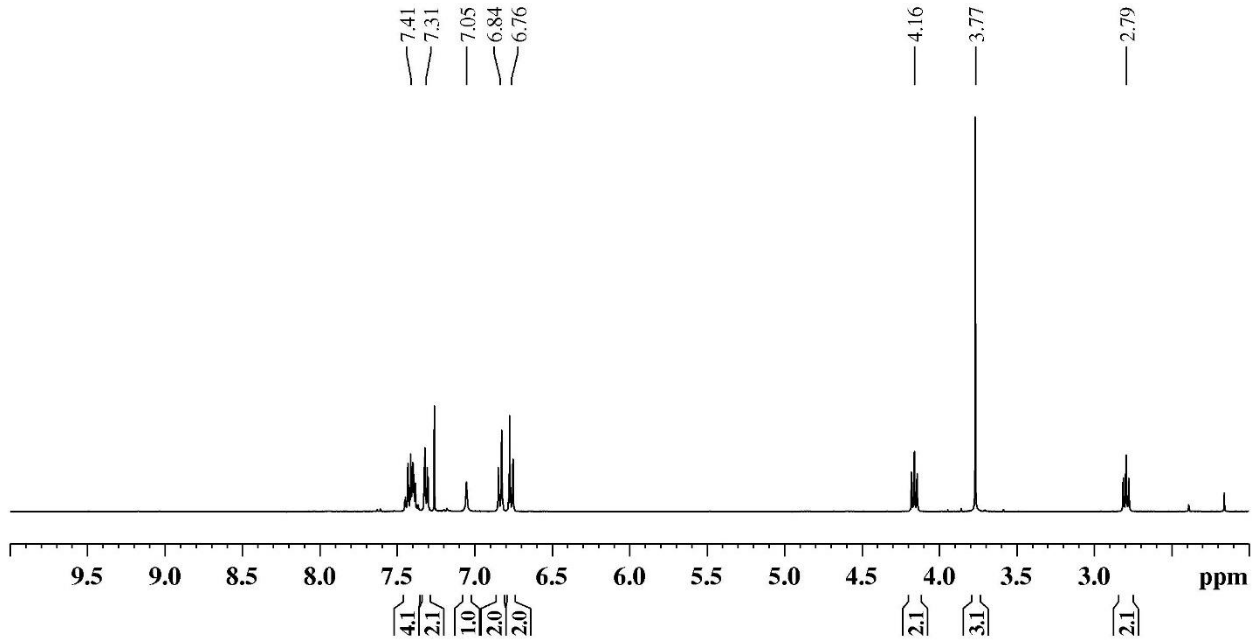

^{13}C NMR ($CDCl_3$, 100 MHz):

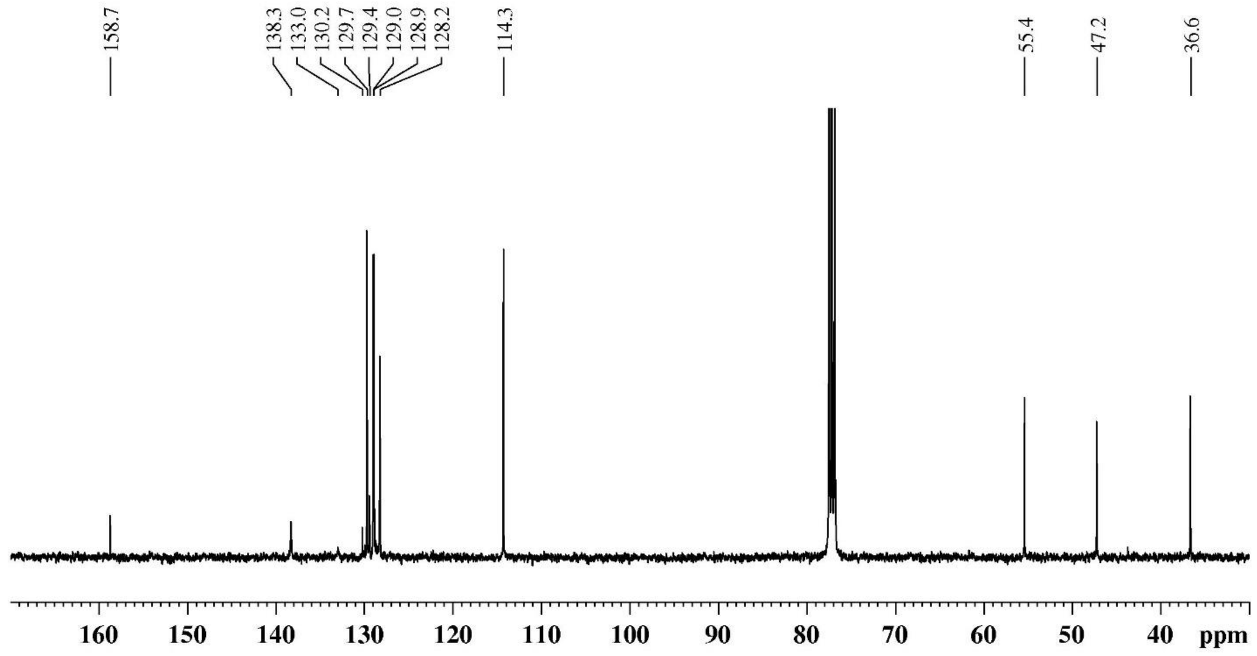

Figure S110. 1-(4-Methoxyphenethyl)-5-(4-methoxyphenyl)-1*H*-imidazole (**110**)

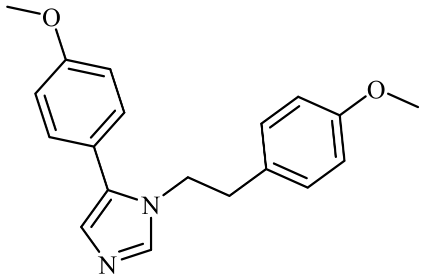

^{1}H NMR ($CDCl_3$, 400 MHz):

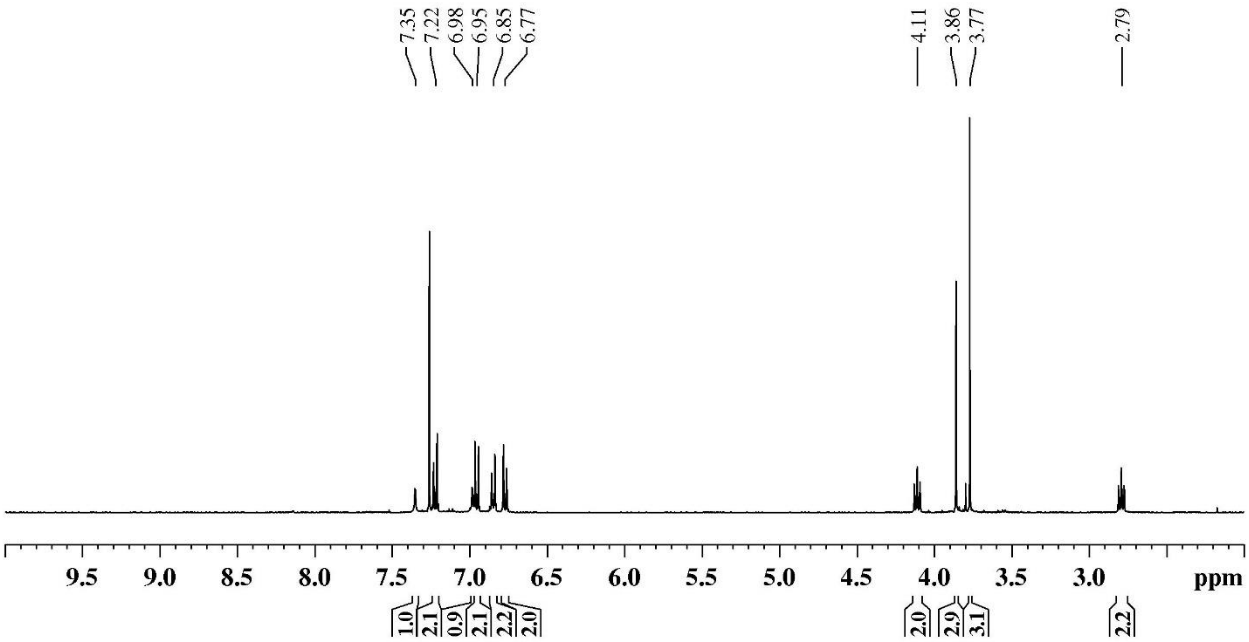

^{13}C NMR ($CDCl_3$, 100 MHz):

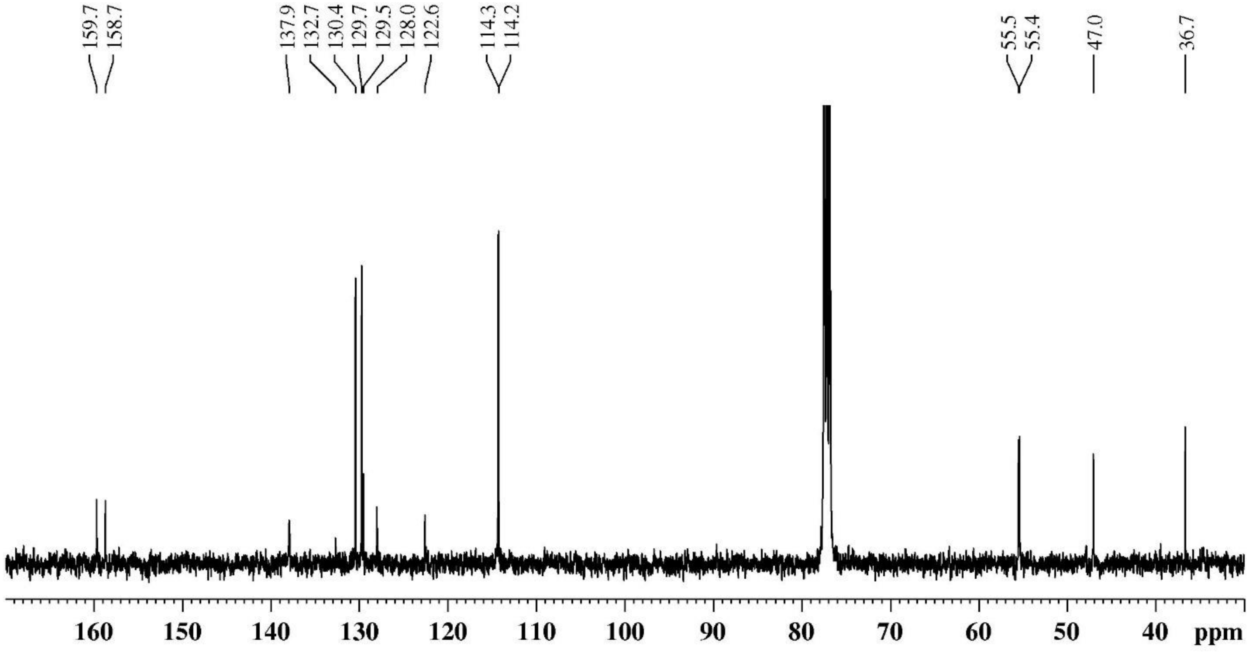

Figure S111. 3-((5-Phenyl-1*H*-imidazol-1-yl)methyl)-1*H*-indole (**111**)

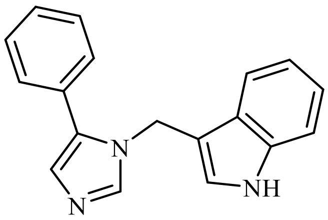

^{1}H NMR ($CDCl_3$, 400 MHz):

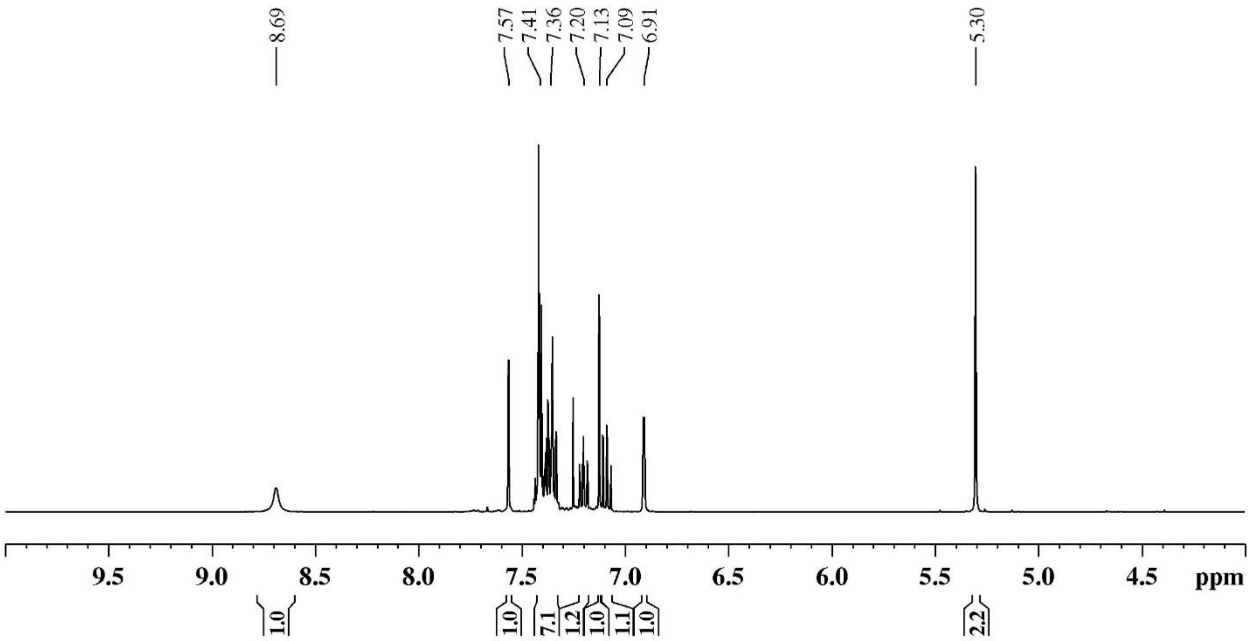

^{13}C NMR ($CDCl_3$, 100 MHz):

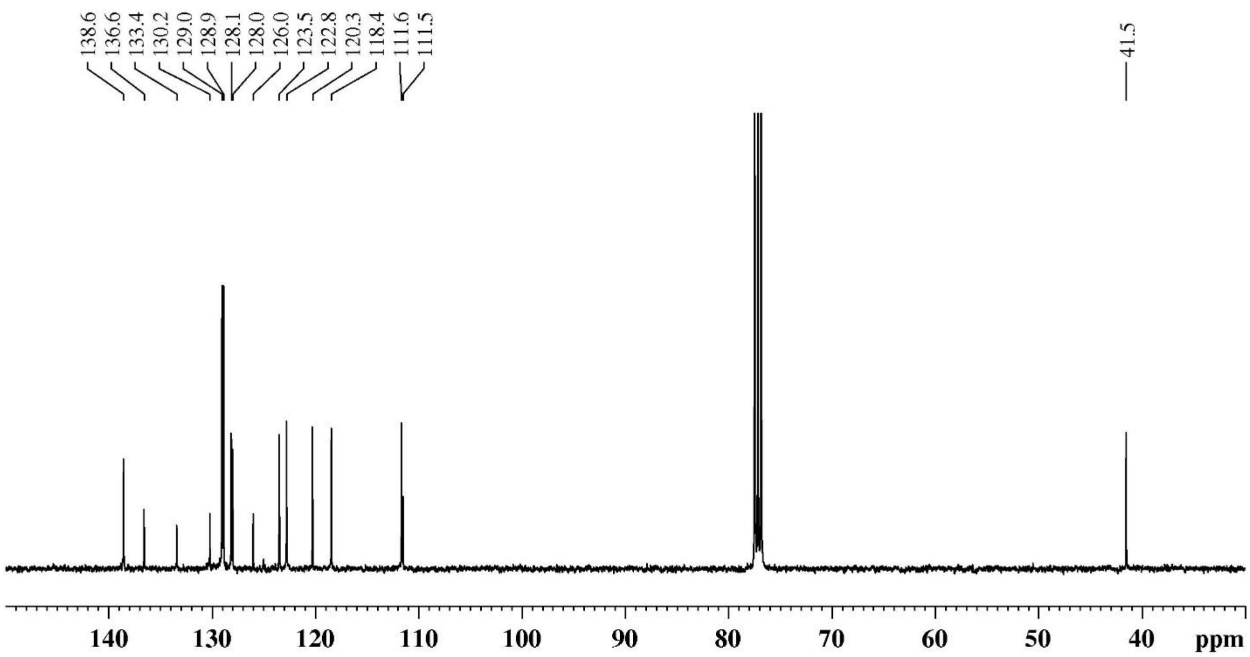

Figure S112. 3-((5-(4-Methoxyphenyl)-1*H*-imidazol-1-yl)methyl)-1*H*-indole (**112**)

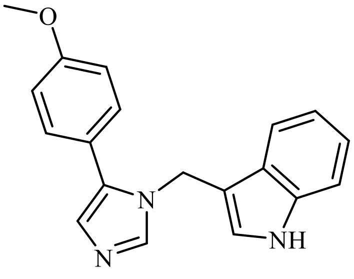

^{1}H NMR ($CDCl_3$, 400 MHz):

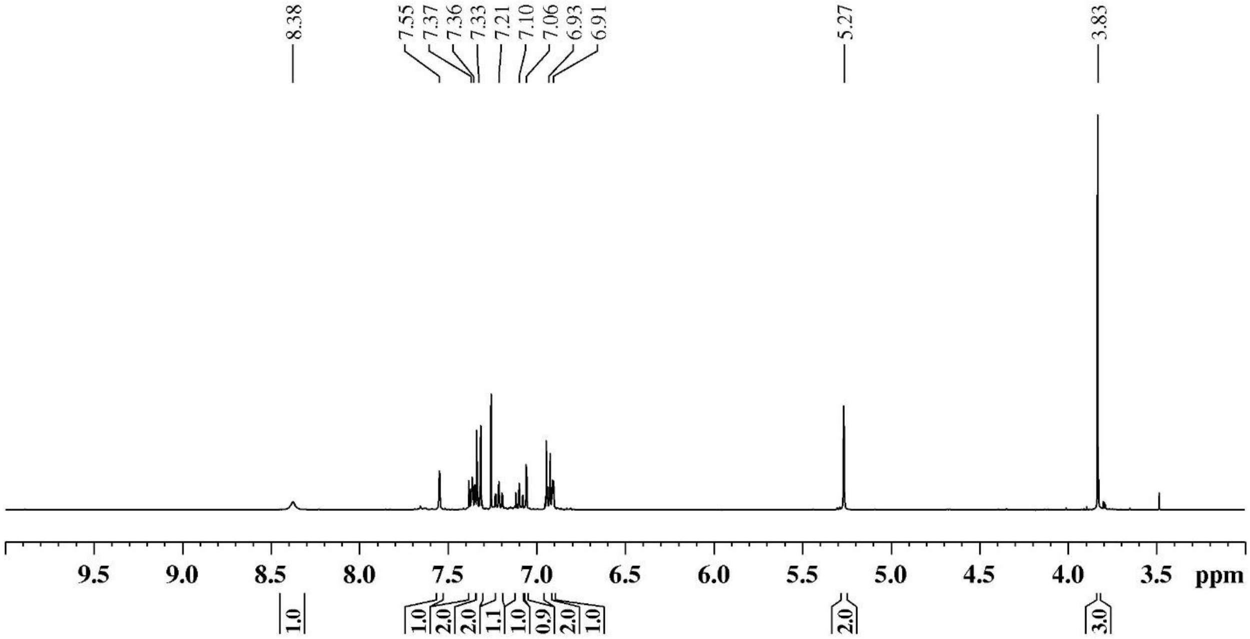

^{13}C NMR ($CDCl_3$, 100 MHz):

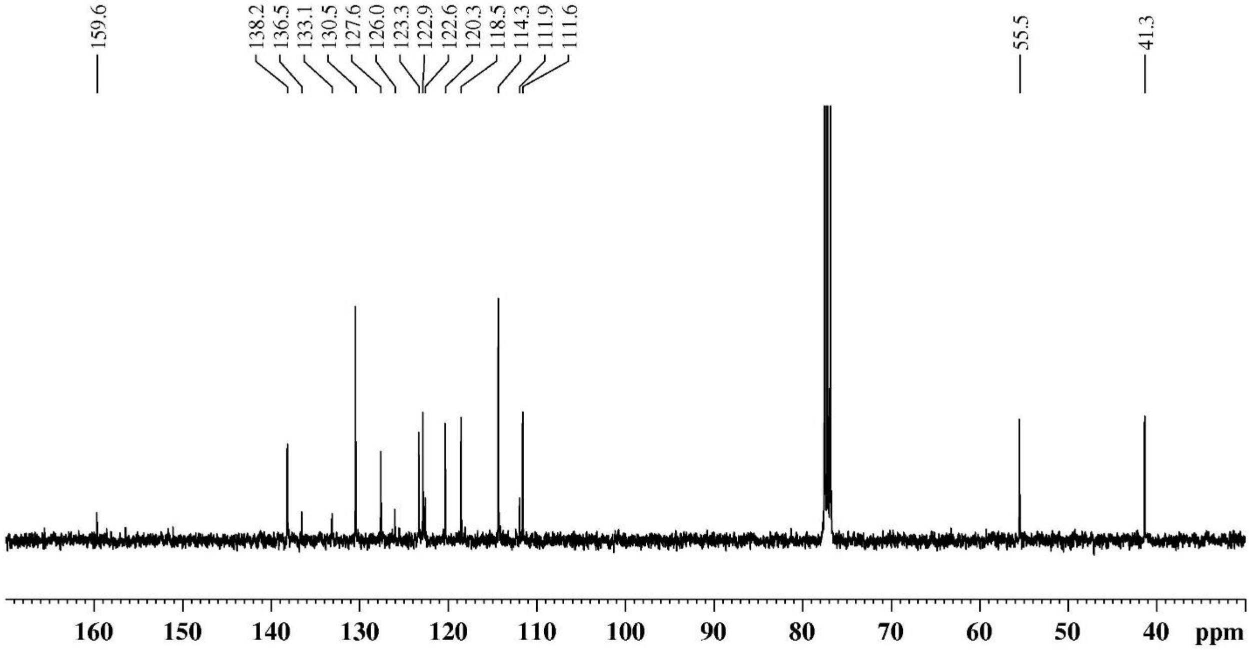

Figure S113. 3-(2-(5-Phenyl-1*H*-imidazol-1-yl)ethyl)-1*H*-indole (**113**)

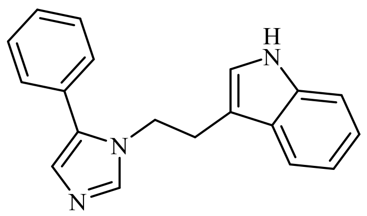

^{1}H NMR ($CDCl_3$, 400 MHz):

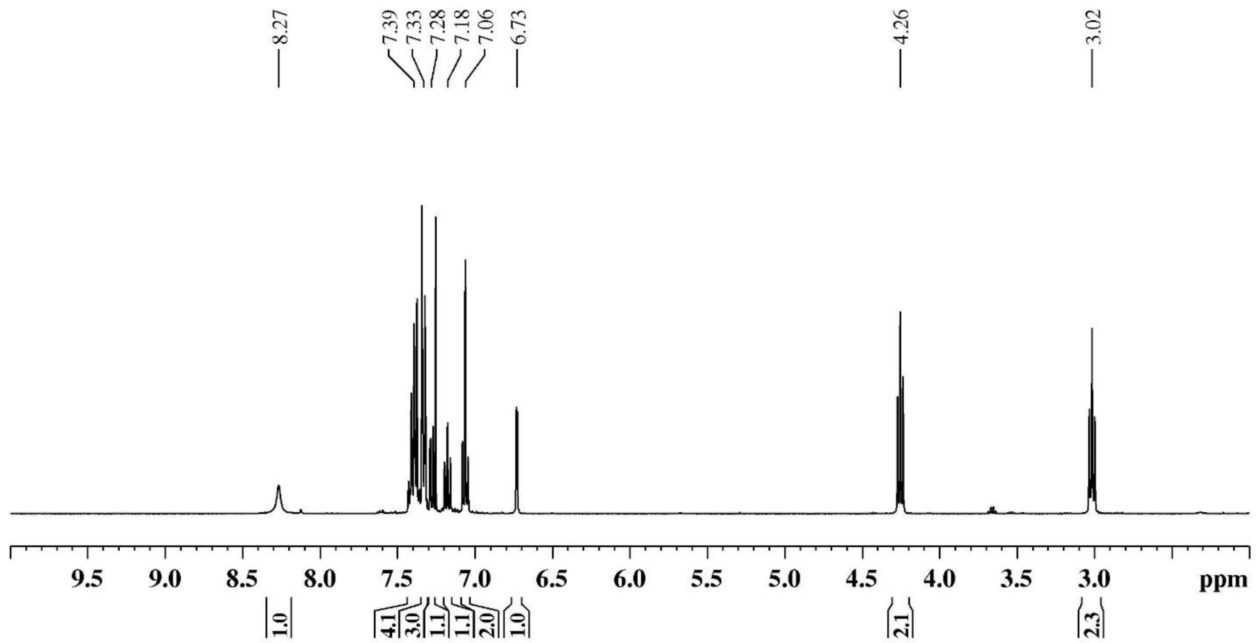

^{13}C NMR ($CDCl_3$, 100 MHz):

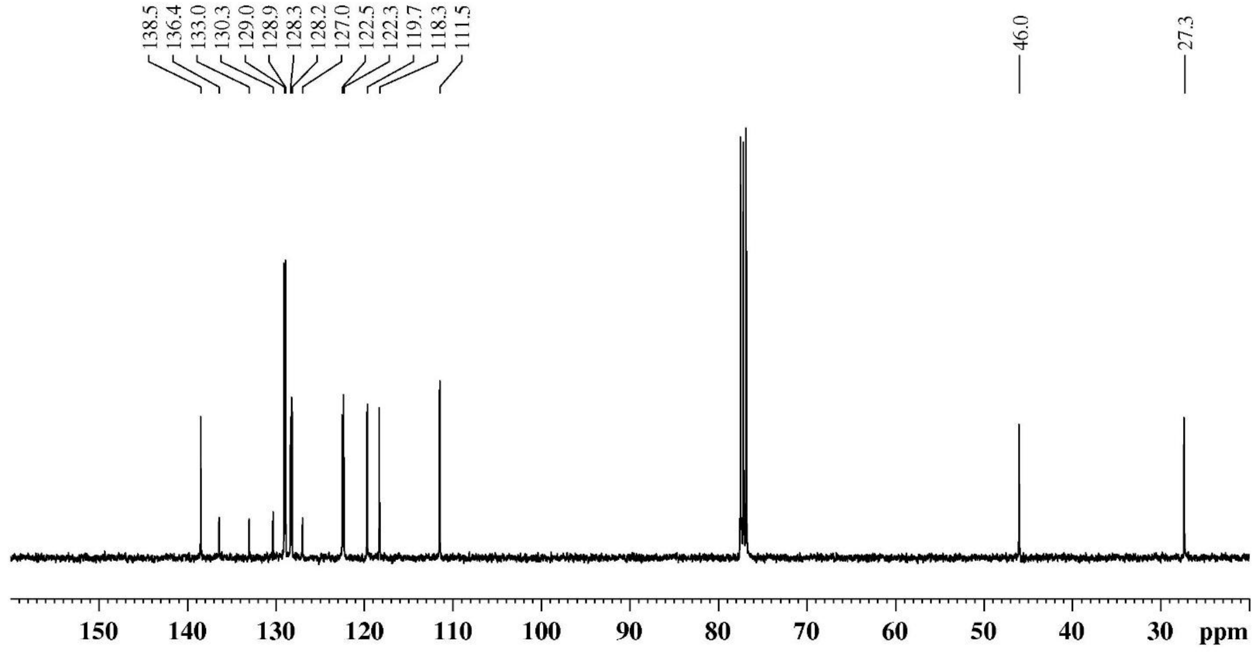

Figure S114. 3-(2-(5-(4-Methoxyphenyl)-1*H*-imidazol-1-yl)ethyl)-1*H*-indole (**114**)

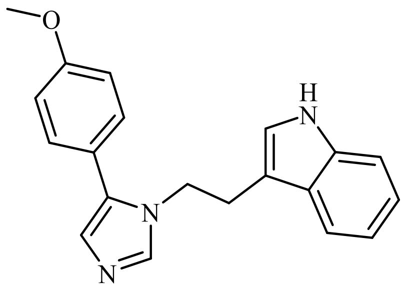

^{1}H NMR ($CDCl_3$, 400 MHz):

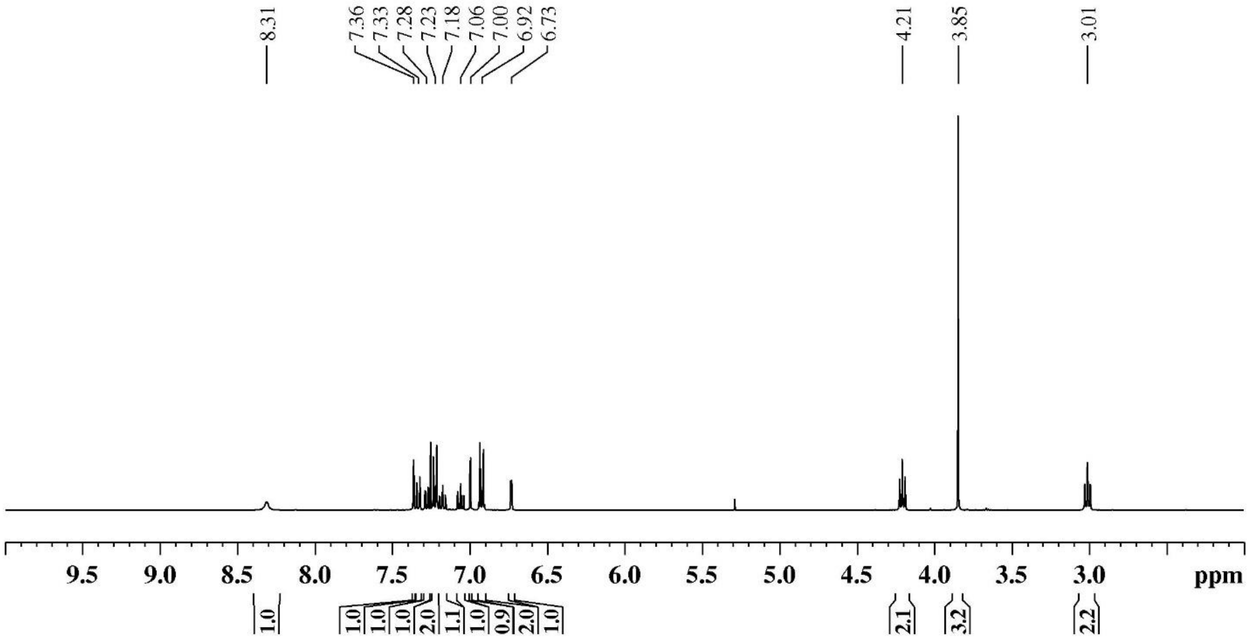

^{13}C NMR ($CDCl_3$, 100 MHz):

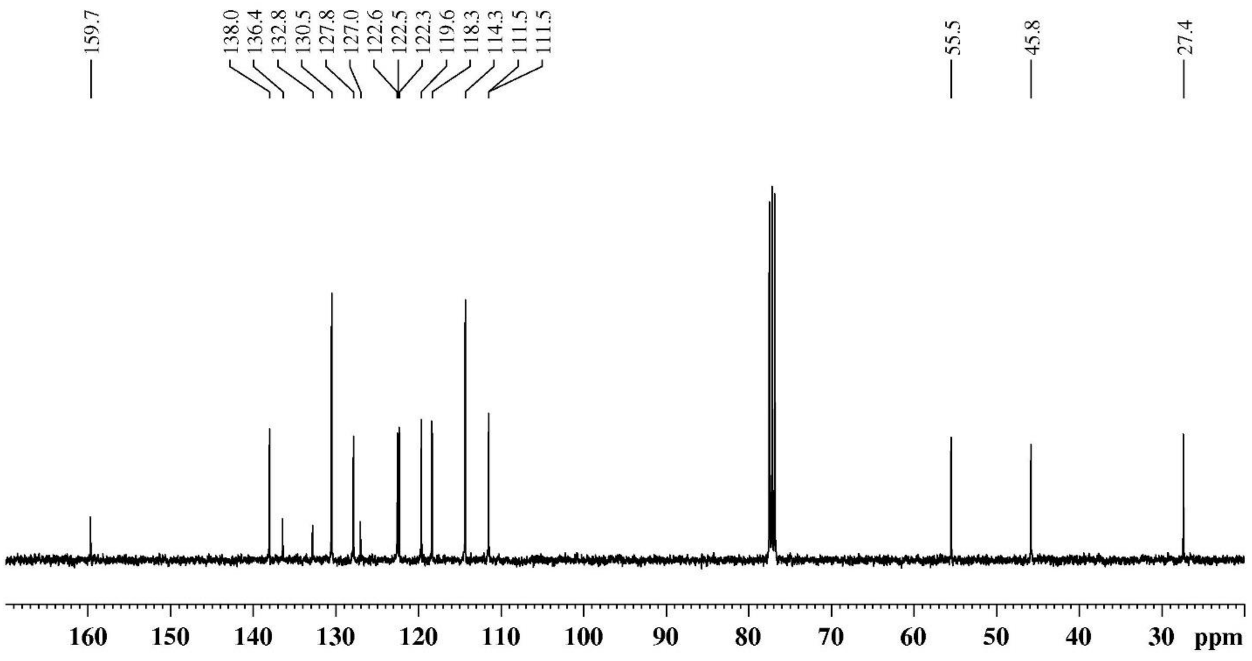

Figure S115. 1-Pentyl-5-phenyl-1*H*-imidazole (**115**)

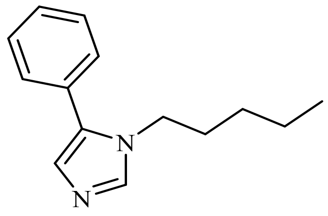

^{1}H NMR ($CDCl_3$, 400 MHz):

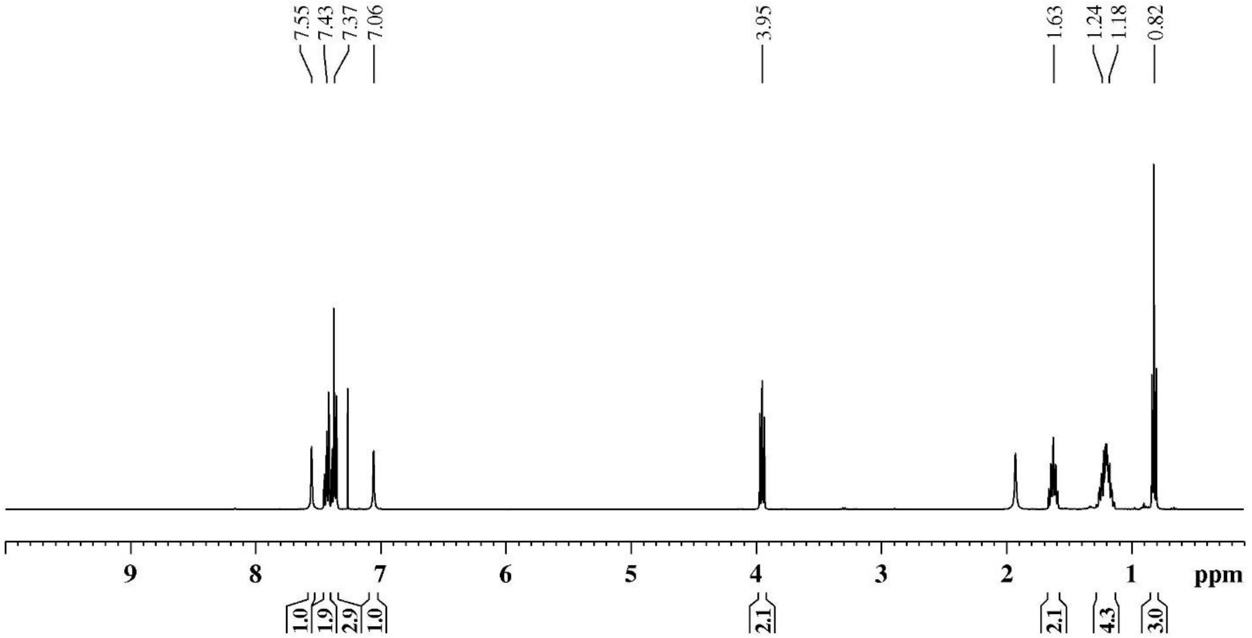

^{13}C NMR ($CDCl_3$, 100 MHz):

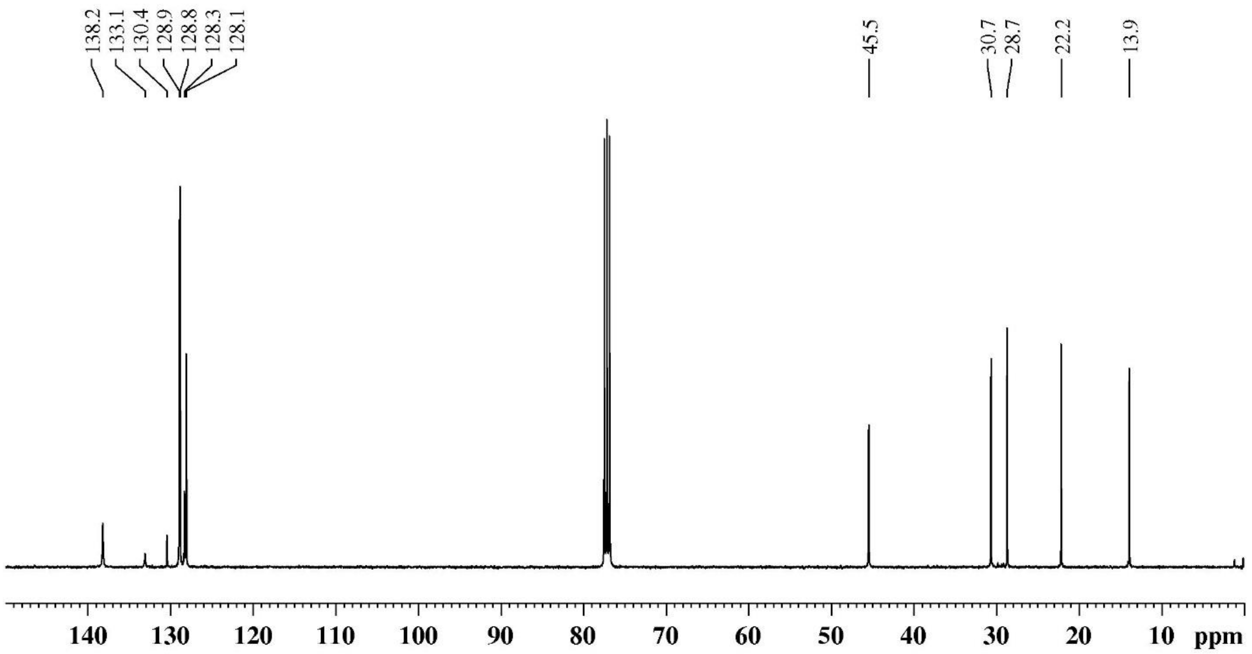

Figure S116. 5-(4-Methoxyphenyl)-1-pentyl-1*H*-imidazole (**116**)

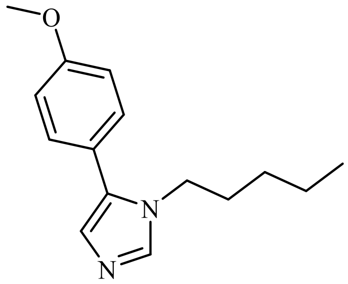

^{1}H NMR ($CDCl_3$, 400 MHz):

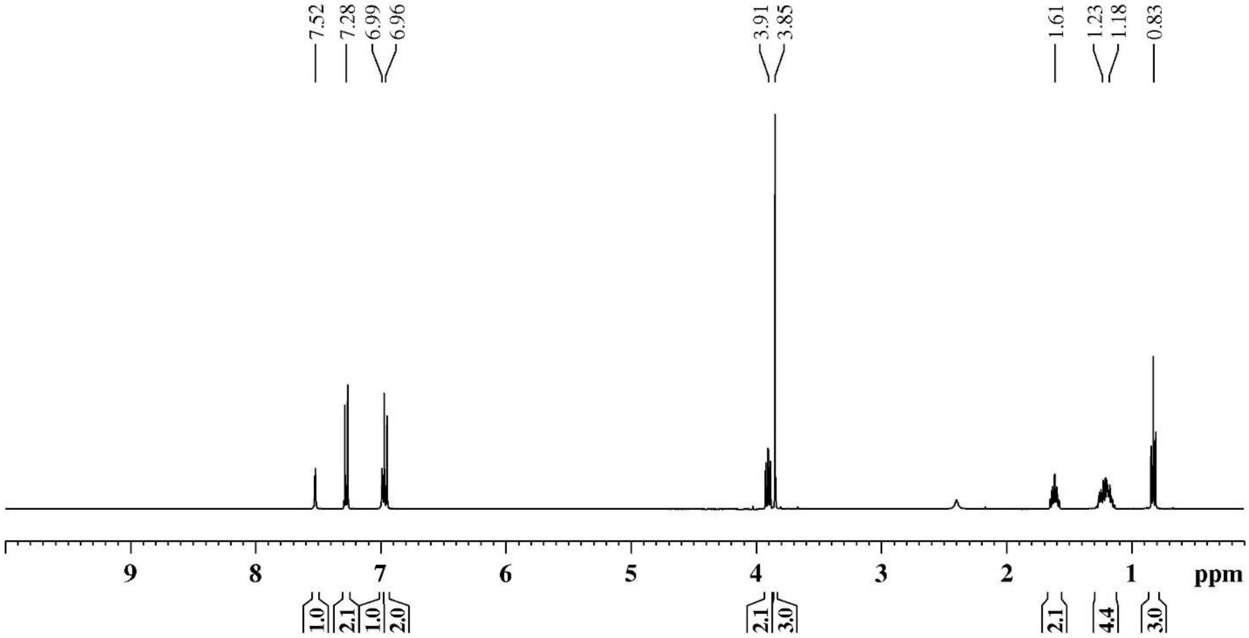

^{13}C NMR ($CDCl_3$, 100 MHz):

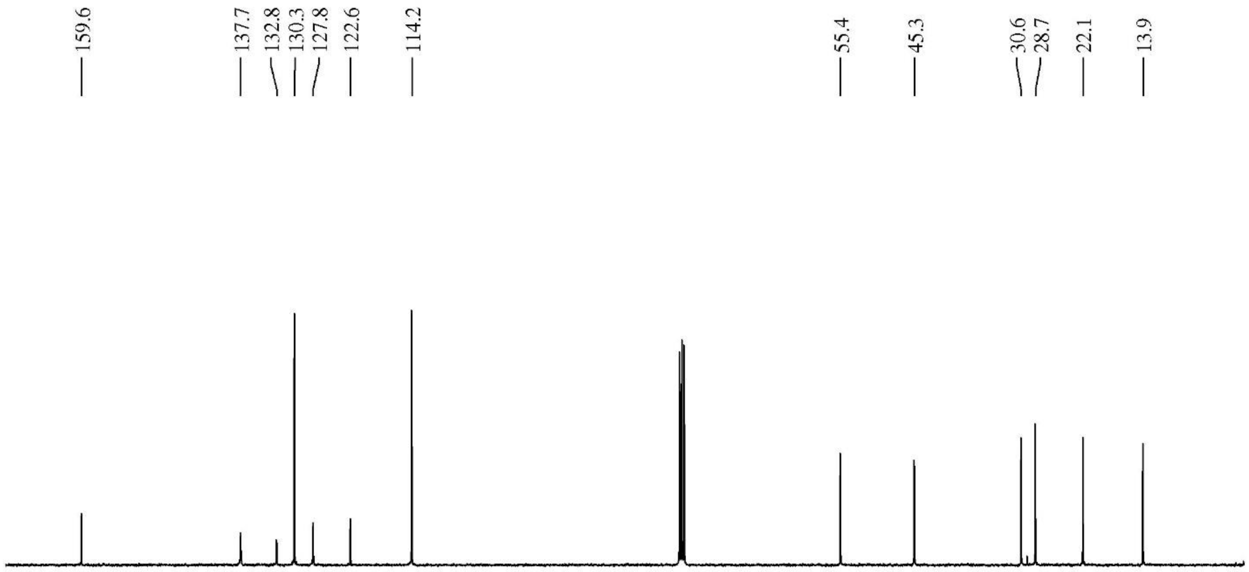

Supplement: Supplementary file 1 [file antibiotics-11-01450-s001.zip › antibiotics-1990342-supplementary.pdf]
